# Diretriz Brasileira de Hipertensão Arterial – 2025

**DOI:** 10.36660/abc.20250624

**Published:** 2025-09-18

**Authors:** Andréa Araujo Brandão, Cibele Isaac Saad Rodrigues, Luiz Aparecido Bortolotto, Anderson da Costa Armstrong, Rogério Andrade Mulinari, Audes Diógenes de Magalhães Feitosa, Marco Antonio Mota-Gomes, Eduardo Costa Duarte Barbosa, José Andrade Moura, Mario Fritsch Toros Neves, Kunz Sebba Barroso Weimar, Claudia Lucia de Moraes Forjaz, Márcia Regina Simas Torres Klein, Fernando Nobre, Paulo César Brandão Veiga Jardim, Celso Amodeo, Rogério Baumgratz de Paula, Sebastião Rodrigues Ferreira, João Roberto Gemelli, José Fernando Vilela-Martin, Elizabeth Silaid Muxfeldt, Décio Mion, Marcus Vinicius Bolivar Malachias, Carlos Alberto Machado, Fernando Antonio de Almeida, Alexandre Jorge Gomes de Lucena, Alvaro Avezum, Amaury Zatorre Amaral, Ana Flavia Moura, Ana Lúcia Rego Fleury de Camargo, Andrea Pio de Abreu, Andrei C. Sposito, Angela Maria Geraldo Pierin, Annelise Machado Gomes de Paiva, Antonio Gabriele Laurinavicius, Carlos Eduardo Poli-de-Figueiredo, Dilma do Socorro Moraes de Souza, Elizabete Viana de Freitas, Emilton Lima, Erika Maria Gonçalves Campana, Fábio Argenta, Fernanda Marciano Consolim Colombo, Fernanda Salomão Gorayeb Polacchini, Flavio Antonio de Oliveira Borelli, Frida Liane Plavnik, Giovanio Vieira da Silva, Grazia Maria Guerra, Heno Ferreira Lopes, José Augusto Soares Barreto, Juan Carlos Yugar Toledo, Leda Aparecida Daud Lotaif, Lílian Soares da Costa, Lucelia Batista Neves Cunha Magalhães, Luciano Ferreira Drager, Luis Cuadrado Martin, Luiz César Nazário Scala, Maria Eliane Campos Magalhães, Maria Eliete Pinheiro, Maria Emília Figueiredo Teixeira, Nelson Dinamarco, Osni Moreira, Oswaldo Passarelli, Otavio Rizzi Coelho, Renault Mattos Ribeiro, Roberto Dischinger Miranda, Rodrigo Bezerra, Rodrigo Pinto Pedrosa, Rogério Toshiro Passos Okawa, Rui Manuel dos Santos Povoa, Sandra Lia do Amaral Cardoso, Sayuri Inuzuka, Sergio Emanuel Kaiser, Sheyla Cristina Tonheiro Ferro da Silva, Tânia Plens Shecaira, Vera Hermina Kalika Koch, Wilson Nadruz

**Affiliations:** 1 Universidade do Estado do Rio de Janeiro Rio de Janeiro RJ Brasil Universidade do Estado do Rio de Janeiro (UERJ), Rio de Janeiro, RJ – Brasil; 2 Pontifícia Universidade Católica de São Paulo Faculdade de Ciências Médicas e da Saúde Sorocaba SP Brasil Pontifícia Universidade Católica de São Paulo, Faculdade de Ciências Médicas e da Saúde, Sorocaba, SP – Brasil; 3 Hospital das Clínicas da Faculdade de Medicina da Universidade de São Paulo Instituto do Coração São Paulo SP Brasil Instituto do Coração (Incor) do Hospital das Clínicas da Faculdade de Medicina da Universidade de São Paulo (HCFMUSP), São Paulo, SP – Brasil; 4 Universidade Federal do Vale do São Francisco Petrolina PE Brasil Universidade Federal do Vale do São Francisco, Petrolina, PE – Brasil; 5 Universidade Federal do Paraná Curitiba PR Brasil Universidade Federal do Paraná (UFPR), Curitiba, PR – Brasil; 6 Universidade Federal de Pernambuco Recife PE Brasil Universidade Federal de Pernambuco, Recife, PE – Brasil; 7 Centro Universitário CESMAC Maceió AL Brasil Centro Universitário CESMAC, Maceió, AL – Brasil; 8 Hospital do Coração de Alagoas Maceió AL Brasil Hospital do Coração de Alagoas, Maceió, AL – Brasil; 9 Centro de Pesquisas Clínicas Dr. Marco Mota Maceió AL Brasil Centro de Pesquisas Clínicas Dr. Marco Mota, Maceió, AL – Brasil; 10 Universidade FEEVALE Porto Alegre RS Brasil Universidade FEEVALE, Porto Alegre, RS – Brasil; 11 Escola Bahiana de Medicina e Saúde Pública Salvador BA Brasil Escola Bahiana de Medicina e Saúde Pública (EBMSP), Salvador, BA – Brasil; 12 Faculdade de Medicina da Universidade Federal de Goiás Goiânia GO Brasil Faculdade de Medicina da Universidade Federal de Goiás (UFG), Goiânia, GO – Brasil; 13 Escola de Educação Física e Esporte da Universidade de São Paulo São Paulo SP Brasil Escola de Educação Física e Esporte da Universidade de São Paulo (USP), São Paulo, SP – Brasil; 14 Hospital das Clínicas da Faculdade de Medicina de Ribeirão Preto da Universidade de São Paulo Ribeirão Preto SP Brasil Hospital das Clínicas da Faculdade de Medicina de Ribeirão Preto da Universidade de São Paulo, Ribeirão Preto, SP – Brasil; 15 Hcor Associação Beneficente Síria São Paulo SP Brasil Hcor, Associação Beneficente Síria, São Paulo, SP – Brasil; 16 Universidade Federal de Juiz de Fora Juiz de Fora MG Brasil Universidade Federal de Juiz de Fora, Juiz de Fora, MG – Brasil; 17 Universidade Federal de Uberlândia Uberlândia MG Brasil Universidade Federal de Uberlândia, Uberlândia, MG – Brasil; 18 Governo do Estado de Rondônia Porto Velho RO Brasil Governo do Estado de Rondônia, Porto Velho, RO – Brasil; 19 Faculdade de Medicina de São José do Rio Preto São José do Rio Preto SP Brasil Faculdade de Medicina de São José do Rio Preto (FAMERP), São José do Rio Preto, SP – Brasil; 20 Universidade Federal do Rio de Janeiro Hospital Universitário Clementino Fraga Filho Rio de Janeiro RJ Brasil Hospital Universitário Clementino Fraga Filho, Universidade Federal do Rio de Janeiro (UFRJ), Rio de Janeiro, RJ – Brasil; 21 Hospital das Clínicas da Faculdade de Medicina da Universidade de São Paulo São Paulo SP Brasil Hospital das Clínicas da Faculdade de Medicina da Universidade de São Paulo (HCFMUSP), São Paulo, SP – Brasil; 22 Faculdade Ciências Médicas de Minas Gerais Belo Horizonte MG Brasil Faculdade Ciências Médicas de Minas Gerais, Belo Horizonte, MG – Brasil; 23 Fundação Educacional Lucas Machado Belo Horizonte MG Brasil Fundação Educacional Lucas Machado (FELUMA), Belo Horizonte, MG – Brasil; 24 Instituto de Hipertensão de Minas Gerais Belo Horizonte MG Brasil Instituto de Hipertensão de Minas Gerais, Belo Horizonte, MG – Brasil; 25 Ministério da Saúde Brasília DF Brasil Ministério da Saúde, Brasília, DF – Brasil; 26 Pontifícia Universidade Católica de São Paulo Fundação São Paulo Sorocaba SP Brasil Fundação São Paulo, Pontifícia Universidade Católica de São Paulo, Sorocaba, SP – Brasil; 27 Hospital Agamenom Magalhães Recife PE Brasil Hospital Agamenom Magalhães, Recife, PE – Brasil; 28 Hospital Alemão Oswaldo Cruz São Paulo SP Brasil Hospital Alemão Oswaldo Cruz, São Paulo, SP – Brasil; 29 Universidade de São Paulo São Paulo SP Brasil Universidade de São Paulo (USP), São Paulo, SP – Brasil; 30 Universidade Federal de São Paulo São Paulo SP Brasil Universidade Federal de São Paulo (UNIFESP), São Paulo, SP – Brasil; 31 PHRI-McMaster University Ontário Canadá PHRI-McMaster University, Ontário – Canadá; 32 Secretaria Municipal de Saúde de São Paulo São Paulo SP Brasil Secretaria Municipal de Saúde de São Paulo, São Paulo, SP – Brasil; 33 Universidade Estadual de Campinas Campinas SP Brasil Universidade Estadual de Campinas (UNICAMP), Campinas, SP – Brasil; 34 Centro de Pesquisas Clínicas Dr. Marco Mota Maceió AL Brasil Centro de Pesquisas Clínicas Dr. Marco Mota, Maceió, AL – Brasil; 35 Instituto Dante Pazzanese de Cardiologia São Paulo SP Brasil Instituto Dante Pazzanese de Cardiologia, São Paulo, SP – Brasil; 36 Pontifícia Universidade Católica do Rio Grande do Sul Porto Alegre RS Brasil Pontifícia Universidade Católica do Rio Grande do Sul, Porto Alegre, RS – Brasil; 37 Universidade Federal do Pará Belém PA Brasil Universidade Federal do Pará (UFPA), Belém, PA – Brasil; 38 Fundação Pública Estadual Hospital de Clínicas Gaspar Vianna Belém PA Brasil Fundação Pública Estadual Hospital de Clínicas Gaspar Vianna, Belém, PA – Brasil; 39 Universidade Federal do Paraná Curitiba PR Brasil Universidade Federal do Paraná, Curitiba, PR – Brasil; 40 Instituto Dr. Fábio Argenta Cuiabá MT Brasil Instituto Dr. Fábio Argenta, Cuiabá, MT – Brasil; 41 Hospital de Base de São José do Rio Preto São José do Rio Preto SP Brasil Hospital de Base de São José do Rio Preto, São José do Rio Preto, SP – Brasil; 42 Rede D’Or São Luiz Rio de Janeiro RJ Brasil Rede D’Or São Luiz, Rio de Janeiro, RJ – Brasil; 43 Universidade Cruzeiro do Sul São Paulo SP Brasil Universidade Cruzeiro do Sul (UNCSUL), São Paulo, SP – Brasil; 44 Faculdade Beneficência Portuguesa São Paulo SP Brasil Faculdade Beneficência Portuguesa, São Paulo, SP – Brasil; 45 Universidade Federal de Sergipe Aracaju SE Brasil Universidade Federal de Sergipe, Aracaju, SE – Brasil; 46 Faculdade de Medicina de São José do Rio Preto São José do Rio Preto SP Brasil Faculdade de Medicina de São José do Rio Preto (FAMERP), São José do Rio Preto, SP – Brasil; 47 ENDOCOR São José do Rio Preto SP Brasil ENDOCOR, São José do Rio Preto, SP – Brasil; 48 Escola de Ciências da Saúde e Bem-estar Faculdade Souza Marques Rio de Janeiro RJ Brasil Escola de Ciências da Saúde e Bem-estar, Faculdade Souza Marques, Rio de Janeiro, RJ – Brasil; 49 Universidade Estácio de Sá Rio de Janeiro RJ Brasil Universidade Estácio de Sá, Rio de Janeiro, RJ – Brasil; 50 Instituto Estadual de Cardiologia Aloysio de Castro Rio de Janeiro RJ Brasil Instituto Estadual de Cardiologia Aloysio de Castro – IECAC, Rio de Janeiro, RJ – Brasil; 51 Faculdade Zarns Salvador BA Brasil Faculdade Zarns, Salvador, BA – Brasil; 52 Universidade Estadual Paulista São Paulo SP Brasil Universidade Estadual Paulista (UNESP), São Paulo, SP – Brasil; 53 Universidade Federal de Mato Grosso Cuiabá MT Brasil Universidade Federal de Mato Grosso, Cuiabá, MT – Brasil; 54 Universidade do Estado do Rio de Janeiro Hospital Universitário Pedro Ernesto Rio de Janeiro RJ Brasil Hospital Universitário Pedro Ernesto, Universidade do Estado do Rio de Janeiro (UERJ), Rio de Janeiro, RJ – Brasil; 55 Universidade Federal de Alagoas Maceió AL Brasil Universidade Federal de Alagoas, Maceió, AL – Brasil; 56 Universidade Estadual de Santa Cruz Ilhéus BA Brasil Universidade Estadual de Santa Cruz (UESC), Ilhéus, BA – Brasil; 57 Clínica Gapski Moreira Curitiba PR Brasil Clínica Gapski Moreira, Curitiba, PR – Brasil; 58 Cardios Vita Brasília DF Brasil Cardios Vita, Brasília, DF – Brasil; 59 Hospital Santa Lucia Brasília DF Brasil Hospital Santa Lucia, Brasília, DF – Brasil; 60 Pronto Socorro Cardiológico de Pernambuco Recife PE Brasil Pronto Socorro Cardiológico de Pernambuco (PROCAPE), Recife, PE – Brasil; 61 Universidade Estadual de Maringá Maringá PR Brasil Universidade Estadual de Maringá, Maringá, PR – Brasil; 62 Universidade Estadual Paulista Campus de Bauru São Paulo SP Brasil Universidade Estadual Paulista, Campus de Bauru, São Paulo, SP – Brasil; 63 Hospital de Acidentados Goiânia GO Brasil Hospital de Acidentados, Goiânia, GO – Brasil; 64 CEMISE Oncoclínicas Aracaju SE Brasil CEMISE Oncoclínicas, Aracaju, SE – Brasil; 65 Prefeitura Municipal de Aracaju Aracaju SE Brasil Prefeitura Municipal de Aracaju, Aracaju, SE – Brasil

## Capítulos e participações:

**1. INTRODUÇÃO**
  **Membros:** Andréa Araujo Brandão, Cibele Isaac Saad Rodrigues, Luiz Aparecido Bortolotto, Wilson Nadruz
**2. EPIDEMIOLOGIA, DEFINIÇÃO E PREVENÇÃO PRIMÁRIA**
  **Coordenadores:** Anderson da Costa Armstrong, Rogério Andrade Mulinari
  **Membros:** Alvaro Avezum Junior, Dilma do Socorro Moraes de Souza, Leda Aparecida Daud Lotaif, Lucelia Batista Neves Cunha Magalhães, Luiz César Nazário Scala
**3. MEDIDA DA PRESSÃO ARTERIAL, DIAGNÓSTICO E CLASSIFICAÇÃO**
  **Coordenadores:** Audes Diógenes de Magalhães Feitosa, Marco Antonio Mota-Gomes
  **Membros:** Angela Maria Geraldo Pierin, Annelise Machado Gomes de Paiva, Lílian Soares da Costa, Nelson Dinamarco, Rodrigo Bezerra
**4. AVALIAÇÃO CLÍNICA E COMPLEMENTAR E ESTRATIFICAÇÃO DE RISCO CARDIOVASCULAR**
  **Coordenadores:** Eduardo Costa Duarte Barbosa, José Andrade Moura-Neto
  **Membros:** Andrei C. Sposito, Antonio Gabriele Laurinavicius, Maria Eliane Campos Magalhães, Rodrigo Pinto Pedrosa, Rogério Toshiro Passos Okawa, Rui Manuel dos Santos Povoa, Sayuri Inuzuka
**5. INÍCIO DE TRATAMENTO E METAS TERAPÊUTICAS**
  **Coordenadores:** Mario Fritsch Toros Neves, Weimar Kuns Sebba Barroso de Souza
  **Membros:** Ana Flavia Moura, Frida Liane Plavnik, Luciano Ferreira Drager, Osni Moreira Filho
**6. MEDIDAS NÃO MEDICAMENTOSAS**
  **Coordenadores:** Claudia Lucia de Moraes Forjaz, Márcia Regina Simas Torres Klein
  **Membros:** Grazia Guerra, Maria Eliete Pinheiro, Maria Emília Figueiredo Teixeira, Tânia Plens Shecaira
**7. TRATAMENTO MEDICAMENTOSO**
  **Coordenadores:** Fernando Nobre, Paulo César Brandão Veiga Jardim
  **Membros:** Fernanda Marciano Consolim Colombo, Otavio Rizzi Coelho, Sergio Emanuel Kaiser
**8. HIPERTENSÃO ARTERIAL E CONDIÇÕES CLÍNICAS ASSOCIADAS: DOENÇA ARTERIAL CORONARIANA, DOENÇA RENAL CRÔNICA, DIABETES MELLITUS, OBESIDADE, COVID-19, PÓS-ACIDENTE VASCULAR CEREBRAL E INSUFICIÊNCIA CARDÍACA**
  **Coordenadores:** Celso Amodeo, Rogério Baumgratz de Paula
  **Membros:** Andrea Pio de Abreu, Emilton Lima Júnior, Heno Ferreira Lopes
**9. HIPERTENSÃO ARTERIAL EM IDOSOS, CRIANÇAS, ADOLESCENTES**
  **Coordenador:** Sebastião Rodrigues Ferreira-Filho
  **Membros:** Alexandre Jorge Gomes de Lucena, Carlos Eduardo Poli de Figueiredo, Elizabete Viana de Freitas, Roberto Dischinger Miranda, Sheyla Cristina Tonheiro Ferro da Silva, Vera Hermina Kalika Koch
**10. HIPERTENSÃO ARTERIAL EM MULHERES**
  **Coordenador:** Sebastião Rodrigues Ferreira-Filho
  **Membros:** Alexandre Jorge Gomes de Lucena, Carlos Eduardo Poli-de-Figueiredo, Elizabete Viana de Freitas, Roberto Dischinger Miranda, Sheyla Cristina Tonheiro Ferro da Silva, Vera Hermina Kalika Koch
**11. CRISE HIPERTENSIVA**
  **Coordenadores:** João Roberto Gemelli, José Fernando Vilela-Martin
  **Membros:** Fábio Argenta, Fernanda Salomão Gorayeb Polacchini, Luis Cuadrado Martin, Renault Mattos Ribeiro Junior
**12. HIPERTENSÃO ARTERIAL RESISTENTE E REFRATÁRIA**
  **Coordenador:** Elizabeth Silaid Muxfeldt
  **Membros:** Erika Maria Gonçalves Campana, Flavio Antonio de Oliveira Borelli, Juan Carlos Yugar Toledo, Oswaldo Passarelli Junior
**13. ADESÃO AO TRATAMENTO ANTI-HIPERTENSIVO**
  **Coordenadores:** Decio Mion Júnior, Marcus Vinicius Bolivar Malachias
  **Membros:** Ana Lúcia Rego Fleury de Camargo, Giovanio Vieira da Silva, Sandra Lia do Amaral Cardoso
**14. MELHORES PRÁTICAS NO CUIDADO ÀS PESSOAS COM HIERTENSÃO ARTERIAL NA ATENÇÃO PRIMÁRIA À SAÚDE DO SISTEMA ÚNICO DE SAÚDE**
  **Coordenadores:** Carlos Alberto Machado, Fernando Antonio de Almeida
  **Membros:** Amaury Zatorre Amaral, José Augusto Soares Barreto-Filho
**15. PERSPECTIVAS**
  **Membros:** Andréa Araujo Brandão, Audes Diógenes de Magalhães Feitosa, Cibele Isaac Saad Rodrigues, Weimar Kuns Sebba Barroso de Souza, Wilson Nadruz

**Table:** 

Diretriz Brasileira de Hipertensão Arterial – 2025	
O relatório abaixo lista as declarações de interesse conforme relatadas à SBC pelos especialistas durante o período de desenvolvimento deste posicionamento, 2024/2025.	
Especialista	Tipo de relacionamento com a indústria
Alexandre Jorge Gomes de Lucena	Nada a ser declarado
Alvaro Avezum	Outros relacionamentos Financiamento de atividades de educação médica continuada, incluindo viagens, hospedagens e inscrições para congressos e cursos, provenientes da indústria farmacêutica, de órteses, próteses, equipamentos e implantes, brasileiras ou estrangeiras: - EMS: Congresso ESC; Biolab: Congresso ESC.
Amaury Zatorre Amaral	Nada a ser declarado
Ana Flavia Moura	Declaração financeira A - Pagamento de qualquer espécie e desde que economicamente apreciáveis, feitos a (i) você, (ii) ao seu cônjuge/ companheiro ou a qualquer outro membro que resida com você, (iii) a qualquer pessoa jurídica em que qualquer destes seja controlador, sócio, acionista ou participante, de forma direta ou indireta, recebimento por palestras, aulas, atuação como proctor de treinamentos, remunerações, honorários pagos por participações em conselhos consultivos, de investigadores, ou outros comitês, etc. Provenientes da indústria farmacêutica, de órteses, próteses, equipamentos e implantes, brasileiras ou estrangeiras: - AstraZeneca: consultoria.
Ana Lúcia Rego Fleury de Camargo	Nada a ser declarado
Anderson da Costa Armstrong	Declaração financeira A - Pagamento de qualquer espécie e desde que economicamente apreciáveis, feitos a (i) você, (ii) ao seu cônjuge/ companheiro ou a qualquer outro membro que resida com você, (iii) a qualquer pessoa jurídica em que qualquer destes seja controlador, sócio, acionista ou participante, de forma direta ou indireta, recebimento por palestras, aulas, atuação como proctor de treinamentos, remunerações, honorários pagos por participações em conselhos consultivos, de investigadores, ou outros comitês, etc. Provenientes da indústria farmacêutica, de órteses, próteses, equipamentos e implantes, brasileiras ou estrangeiras: - Alnylam: amvuttra; Novo Nordisk: Rybelsus e wegovy. Outros relacionamentos Financiamento de atividades de educação médica continuada, incluindo viagens, hospedagens e inscrições para congressos e cursos, provenientes da indústria farmacêutica, de órteses, próteses, equipamentos e implantes, brasileiras ou estrangeiras: - Alnylam: amiloidose; Novo Nordisk: semaglutida. Participação societária de qualquer natureza e qualquer valor economicamente apreciável de empresas na área de saúde, de ensino ou em empresas concorrentes ou fornecedoras da SBC: - Clínicas de trabalho médico: Cardiovasf e Cintilo. Vínculo empregatício com a indústria farmacêutica, de órteses, próteses, equipamentos e implantes, brasileiras ou estrangeiras, assim como se tem relação vínculo empregatício com operadoras de planos de saúde ou em auditorias médicas (incluindo meio período) durante o ano para o qual você está declarando: - Médico cooperado da Unimed Vale do São Francisco.
Andréa Araujo Brandão	Declaração financeira A - Pagamento de qualquer espécie e desde que economicamente apreciáveis, feitos a (i) você, (ii) ao seu cônjuge/ companheiro ou a qualquer outro membro que resida com você, (iii) a qualquer pessoa jurídica em que qualquer destes seja controlador, sócio, acionista ou participante, de forma direta ou indireta, recebimento por palestras, aulas, atuação como proctor de treinamentos, remunerações, honorários pagos por participações em conselhos consultivos, de investigadores, ou outros comitês, etc. Provenientes da indústria farmacêutica, de órteses, próteses, equipamentos e implantes, brasileiras ou estrangeiras: - Biolab: Nebivolol; AstraZeneca: Metoprolol; Servier: Perindopril, Hypera; Mantecorp: Olmesartana e combinações; Torrent: Olmesartana e combinações; Daiichi Sankyo: Olmesartana e combinações. B - Financiamento de pesquisas sob sua responsabilidade direta/pessoal (direcionado ao departamento ou instituição) provenientes da indústria farmacêutica, de órteses, próteses, equipamentos e implantes, brasileiras ou estrangeiras: - Daiichi Sankyo: Olmesartana; Servier: Elfie; Brainfarma: Olmesartana. Outros relacionamentos Financiamento de atividades de educação médica continuada, incluindo viagens, hospedagens e inscrições para congressos e cursos, provenientes da indústria farmacêutica, de órteses, próteses, equipamentos e implantes, brasileiras ou estrangeiras: - Servier: Perindopril; Novo Nordisk: Semaglutida.
Andrea Pio de Abreu	Nada a ser declarado
Andrei C. Sposito	Declaração financeira A - Pagamento de qualquer espécie e desde que economicamente apreciáveis, feitos a (i) você, (ii) ao seu cônjuge/ companheiro ou a qualquer outro membro que resida com você, (iii) a qualquer pessoa jurídica em que qualquer destes seja controlador, sócio, acionista ou participante, de forma direta ou indireta, recebimento por palestras, aulas, atuação como proctor de treinamentos, remunerações, honorários pagos por participações em conselhos consultivos, de investigadores, ou outros comitês, etc. Provenientes da indústria farmacêutica, de órteses, próteses, equipamentos e implantes, brasileiras ou estrangeiras: - Lilly, Novo Nordisk, Daiichi Sankyo. B - Financiamento de pesquisas sob sua responsabilidade direta/pessoal (direcionado ao departamento ou instituição) provenientes da indústria farmacêutica, de órteses, próteses, equipamentos e implantes, brasileiras ou estrangeiras: - AstraZeneca. Outros relacionamentos Financiamento de atividades de educação médica continuada, incluindo viagens, hospedagens e inscrições para congressos e cursos, provenientes da indústria farmacêutica, de órteses, próteses, equipamentos e implantes, brasileiras ou estrangeiras: - Daiichi Sankyo, Novo Nordisk.
Angela Maria Geraldo Pierin	Nada a ser declarado
Annelise Machado Gomes de Paiva	Nada a ser declarado
Antonio Gabriele Laurinavicius	Declaração financeira A - Pagamento de qualquer espécie e desde que economicamente apreciáveis, feitos a (i) você, (ii) ao seu cônjuge/ companheiro ou a qualquer outro membro que resida com você, (iii) a qualquer pessoa jurídica em que qualquer destes seja controlador, sócio, acionista ou participante, de forma direta ou indireta, recebimento por palestras, aulas, atuação como proctor de treinamentos, remunerações, honorários pagos por participações em conselhos consultivos, de investigadores, ou outros comitês, etc. Provenientes da indústria farmacêutica, de órteses, próteses, equipamentos e implantes, brasileiras ou estrangeiras: - AstraZeneca; EMS; Biolab; Servier; Libbs; Merck. Outros relacionamentos Financiamento de atividades de educação médica continuada, incluindo viagens, hospedagens e inscrições para congressos e cursos, provenientes da indústria farmacêutica, de órteses, próteses, equipamentos e implantes, brasileiras ou estrangeiras: - Servier.
Audes Diógenes de Magalhães Feitosa	Declaração financeira A - Pagamento de qualquer espécie e desde que economicamente apreciáveis, feitos a (i) você, (ii) ao seu cônjuge/ companheiro ou a qualquer outro membro que resida com você, (iii) a qualquer pessoa jurídica em que qualquer destes seja controlador, sócio, acionista ou participante, de forma direta ou indireta, recebimento por palestras, aulas, atuação como proctor de treinamentos, remunerações, honorários pagos por participações em conselhos consultivos, de investigadores, ou outros comitês, etc. Provenientes da indústria farmacêutica, de órteses, próteses, equipamentos e implantes, brasileiras ou estrangeiras: - Omron: hipertensão; Servier: hipertensão; Biolab: hipertensão; Brace: hipertensão; Daichi Sankyo: hipertensão; FQM: hipertensão; Merck: hipertensão. B - Financiamento de pesquisas sob sua responsabilidade direta/pessoal (direcionado ao departamento ou instituição) provenientes da indústria farmacêutica, de órteses, próteses, equipamentos e implantes, brasileiras ou estrangeiras: - Servier: hipertensão; Brace: hipertensão; Daichi Sankyo: hipertensão; Angem: Lp(a), Lilly: Lp(a), Janssen: anticoagulante; Bayer: Finerinona. Outros relacionamentos Financiamento de atividades de educação médica continuada, incluindo viagens, hospedagens e inscrições para congressos e cursos, provenientes da indústria farmacêutica, de órteses, próteses, equipamentos e implantes, brasileiras ou estrangeiras: - Servier: hipertensão; FQM: hipertensão; Novo Nordisk: diabetes e obesidade; Sanofi: vacinas.
Carlos Alberto Machado	Nada a ser declarado
Carlos Eduardo Poli-de-Figueiredo	Declaração financeira A - Pagamento de qualquer espécie e desde que economicamente apreciáveis, feitos a (i) você, (ii) ao seu cônjuge/ companheiro ou a qualquer outro membro que resida com você, (iii) a qualquer pessoa jurídica em que qualquer destes seja controlador, sócio, acionista ou participante, de forma direta ou indireta, recebimento por palestras, aulas, atuação como proctor de treinamentos, remunerações, honorários pagos por participações em conselhos consultivos, de investigadores, ou outros comitês, etc. Provenientes da indústria farmacêutica, de órteses, próteses, equipamentos e implantes, brasileiras ou estrangeiras: - AstraZeneca: SHUA e Doença renal crônica; Organização do evento MAYOPUCRS 2023 e de outros congressos de Sociedades Médicas com apoio de empresas. B - Financiamento de pesquisas sob sua responsabilidade direta/pessoal (direcionado ao departamento ou instituição) provenientes da indústria farmacêutica, de órteses, próteses, equipamentos e implantes, brasileiras ou estrangeiras: - Pesquisador do Centro de Pesquisa Clínica da PUCRS. Sem estudos no momento. Outros relacionamentos Financiamento de atividades de educação médica continuada, incluindo viagens, hospedagens e inscrições para congressos e cursos, provenientes da indústria farmacêutica, de órteses, próteses, equipamentos e implantes, brasileiras ou estrangeiras: - Consultório médico (Nefromonitoriza). Participação em estudos financiados pelo Ministério da Saúde, CNPq e CAPES. Participação societária de qualquer natureza e qualquer valor economicamente apreciável de empresas na área de saúde, de ensino ou em empresas concorrentes ou fornecedoras da SBC: - Consultório médico (Nefromonitoriza). Participação em órgãos governamentais de regulação, ou de defesa de direitos na área de cardiologia: - Consultor FAPERGS.
Celso Amodeo	Declaração financeira A - Pagamento de qualquer espécie e desde que economicamente apreciáveis, feitos a (i) você, (ii) ao seu cônjuge/ companheiro ou a qualquer outro membro que resida com você, (iii) a qualquer pessoa jurídica em que qualquer destes seja controlador, sócio, acionista ou participante, de forma direta ou indireta, recebimento por palestras, aulas, atuação como proctor de treinamentos, remunerações, honorários pagos por participações em conselhos consultivos, de investigadores, ou outros comitês, etc. Provenientes da indústria farmacêutica, de órteses, próteses, equipamentos e implantes, brasileiras ou estrangeiras: - EMS: BRAs. Outros relacionamentos Financiamento de atividades de educação médica continuada, incluindo viagens, hospedagens e inscrições para congressos e cursos, provenientes da indústria farmacêutica, de órteses, próteses, equipamentos e implantes, brasileiras ou estrangeiras: - EMS.
Cibele Isaac Saad Rodrigues	Nada a ser declarado
Claudia Lucia de Moraes Forjaz	Nada a ser declarado
Decio Mion Júnior	Nada a ser declarado
Dilma do Socorro Moraes de Souza	Declaração financeira A - Pagamento de qualquer espécie e desde que economicamente apreciáveis, feitos a (i) você, (ii) ao seu cônjuge/ companheiro ou a qualquer outro membro que resida com você, (iii) a qualquer pessoa jurídica em que qualquer destes seja controlador, sócio, acionista ou participante, de forma direta ou indireta, recebimento por palestras, aulas, atuação como proctor de treinamentos, remunerações, honorários pagos por participações em conselhos consultivos, de investigadores, ou outros comitês, etc. Provenientes da indústria farmacêutica, de órteses, próteses, equipamentos e implantes, brasileiras ou estrangeiras: - Novartis Biociência.
Eduardo Costa Duarte Barbosa	Outros relacionamentos Financiamento de atividades de educação médica continuada, incluindo viagens, hospedagens e inscrições para congressos e cursos, provenientes da indústria farmacêutica, de órteses, próteses, equipamentos e implantes, brasileiras ou estrangeiras: - Servier; Cardios; Mantecorp; Brace Pharma.
Elizabete Viana de Freitas	Nada a ser declarado
Elizabeth Silaid Muxfeldt	Nada a ser declarado
Emilton Lima Júnior	Declaração financeira A - Pagamento de qualquer espécie e desde que economicamente apreciáveis, feitos a (i) você, (ii) ao seu cônjuge/ companheiro ou a qualquer outro membro que resida com você, (iii) a qualquer pessoa jurídica em que qualquer destes seja controlador, sócio, acionista ou participante, de forma direta ou indireta, recebimento por palestras, aulas, atuação como proctor de treinamentos, remunerações, honorários pagos por participações em conselhos consultivos, de investigadores, ou outros comitês, etc. Provenientes da indústria farmacêutica, de órteses, próteses, equipamentos e implantes, brasileiras ou estrangeiras: - Bayer: doença renal; Biolab: hipertensão e dislipidemia; Chiesi: DPOC; Daiichi Sankyo: dislipidemia; Novartis: dislipidemia; Novo Nordisk: cardiometabolismo; Servier: hipertensão e cardiometabolismo. Outros relacionamentos Financiamento de atividades de educação médica continuada, incluindo viagens, hospedagens e inscrições para congressos e cursos, provenientes da indústria farmacêutica, de órteses, próteses, equipamentos e implantes, brasileiras ou estrangeiras: - Servier: hipertensão.
Erika Maria Gonçalves Campana	Declaração financeira A - Pagamento de qualquer espécie e desde que economicamente apreciáveis, feitos a (i) você, (ii) ao seu cônjuge/ companheiro ou a qualquer outro membro que resida com você, (iii) a qualquer pessoa jurídica em que qualquer destes seja controlador, sócio, acionista ou participante, de forma direta ou indireta, recebimento por palestras, aulas, atuação como proctor de treinamentos, remunerações, honorários pagos por participações em conselhos consultivos, de investigadores, ou outros comitês, etc. Provenientes da indústria farmacêutica, de órteses, próteses, equipamentos e implantes, brasileiras ou estrangeiras: - Servier, Brace Pharma, Biolab, Momenta. Outros relacionamentos Financiamento de atividades de educação médica continuada, incluindo viagens, hospedagens e inscrições para congressos e cursos, provenientes da indústria farmacêutica, de órteses, próteses, equipamentos e implantes, brasileiras ou estrangeiras: - Servier, Biolab: hipertensão.
Fábio Argenta	Declaração financeira A - Pagamento de qualquer espécie e desde que economicamente apreciáveis, feitos a (i) você, (ii) ao seu cônjuge/ companheiro ou a qualquer outro membro que resida com você, (iii) a qualquer pessoa jurídica em que qualquer destes seja controlador, sócio, acionista ou participante, de forma direta ou indireta, recebimento por palestras, aulas, atuação como proctor de treinamentos, remunerações, honorários pagos por participações em conselhos consultivos, de investigadores, ou outros comitês, etc. Provenientes da indústria farmacêutica, de órteses, próteses, equipamentos e implantes, brasileiras ou estrangeiras: - Novartis: Sybrava; Boehringer: Jardiance; AstraZeneca: Forxiga; Servier: Triplixam; Daiichi Sankyo: Benicar; GSK: vacinas. Outros relacionamentos Financiamento de atividades de educação médica continuada, incluindo viagens, hospedagens e inscrições para congressos e cursos, provenientes da indústria farmacêutica, de órteses, próteses, equipamentos e implantes, brasileiras ou estrangeiras: - Novo Nordisk: Wegovy; Torrent: Rosucor.
Fernanda Marciano Consolim Colombo	Declaração financeira A - Pagamento de qualquer espécie e desde que economicamente apreciáveis, feitos a (i) você, (ii) ao seu cônjuge/ companheiro ou a qualquer outro membro que resida com você, (iii) a qualquer pessoa jurídica em que qualquer destes seja controlador, sócio, acionista ou participante, de forma direta ou indireta, recebimento por palestras, aulas, atuação como proctor de treinamentos, remunerações, honorários pagos por participações em conselhos consultivos, de investigadores, ou outros comitês, etc. Provenientes da indústria farmacêutica, de órteses, próteses, equipamentos e implantes, brasileiras ou estrangeiras: - Daiichi Sankyo; Merck; Servier; AstraZeneca. Outros relacionamentos Financiamento de atividades de educação médica continuada, incluindo viagens, hospedagens e inscrições para congressos e cursos, provenientes da indústria farmacêutica, de órteses, próteses, equipamentos e implantes, brasileiras ou estrangeiras: - Daiichi Sankyo; Servier.
Fernanda Salomão Gorayeb Polacchini	Declaração financeira A - Pagamento de qualquer espécie e desde que economicamente apreciáveis, feitos a (i) você, (ii) ao seu cônjuge/ companheiro ou a qualquer outro membro que resida com você, (iii) a qualquer pessoa jurídica em que qualquer destes seja controlador, sócio, acionista ou participante, de forma direta ou indireta, recebimento por palestras, aulas, atuação como proctor de treinamentos, remunerações, honorários pagos por participações em conselhos consultivos, de investigadores, ou outros comitês, etc. Provenientes da indústria farmacêutica, de órteses, próteses, equipamentos e implantes, brasileiras ou estrangeiras: - AstraZeneca: Lokelma e Forxiga.
Fernando Antonio de Almeida	Nada a ser declarado
Fernando Nobre	Declaração financeira A - Pagamento de qualquer espécie e desde que economicamente apreciáveis, feitos a (i) você, (ii) ao seu cônjuge/ companheiro ou a qualquer outro membro que resida com você, (iii) a qualquer pessoa jurídica em que qualquer destes seja controlador, sócio, acionista ou participante, de forma direta ou indireta, recebimento por palestras, aulas, atuação como proctor de treinamentos, remunerações, honorários pagos por participações em conselhos consultivos, de investigadores, ou outros comitês, etc. Provenientes da indústria farmacêutica, de órteses, próteses, equipamentos e implantes, brasileiras ou estrangeiras: - EMS; Servier; Novartis; Daiichi Sankyo; Biolab. Outros relacionamentos Financiamento de atividades de educação médica continuada, incluindo viagens, hospedagens e inscrições para congressos e cursos, provenientes da indústria farmacêutica, de órteses, próteses, equipamentos e implantes, brasileiras ou estrangeiras: - AstraZeneca.
Flavio Antonio de Oliveira Borelli	Nada a ser declarado
Frida Liane Plavnik	Declaração financeira A - Pagamento de qualquer espécie e desde que economicamente apreciáveis, feitos a (i) você, (ii) ao seu cônjuge/ companheiro ou a qualquer outro membro que resida com você, (iii) a qualquer pessoa jurídica em que qualquer destes seja controlador, sócio, acionista ou participante, de forma direta ou indireta, recebimento por palestras, aulas, atuação como proctor de treinamentos, remunerações, honorários pagos por participações em conselhos consultivos, de investigadores, ou outros comitês, etc. Provenientes da indústria farmacêutica, de órteses, próteses, equipamentos e implantes, brasileiras ou estrangeiras: - EMS; Servier; Novartis; Daiichi Sankyo; Biolab. Outros relacionamentos Financiamento de atividades de educação médica continuada, incluindo viagens, hospedagens e inscrições para congressos e cursos, provenientes da indústria farmacêutica, de órteses, próteses, equipamentos e implantes, brasileiras ou estrangeiras: - Libbs: candesartana.
Giovanio Vieira da Silva	Nada a ser declarado
Grazia Maria Guerra	Nada a ser declarado
Heno Ferreira Lopes	Nada a ser declarado
João Roberto Gemelli	Declaração financeira A - Pagamento de qualquer espécie e desde que economicamente apreciáveis, feitos a (i) você, (ii) ao seu cônjuge/ companheiro ou a qualquer outro membro que resida com você, (iii) a qualquer pessoa jurídica em que qualquer destes seja controlador, sócio, acionista ou participante, de forma direta ou indireta, recebimento por palestras, aulas, atuação como proctor de treinamentos, remunerações, honorários pagos por participações em conselhos consultivos, de investigadores, ou outros comitês, etc. Provenientes da indústria farmacêutica, de órteses, próteses, equipamentos e implantes, brasileiras ou estrangeiras: - AstraZeneca: Forxiga, Breztri; Novo Nordisk: Rybelsus; Servier: Trilpixam e Vastarel. Outros relacionamentos Financiamento de atividades de educação médica continuada, incluindo viagens, hospedagens e inscrições para congressos e cursos, provenientes da indústria farmacêutica, de órteses, próteses, equipamentos e implantes, brasileiras ou estrangeiras: - Astrazeneca: Forxiga, Breztri; Novo Nordisk: Rybelsus; Servier: Trimplixam, Vastarel.
José Andrade Moura-Neto	Declaração financeira A - Pagamento de qualquer espécie e desde que economicamente apreciáveis, feitos a (i) você, (ii) ao seu cônjuge/ companheiro ou a qualquer outro membro que resida com você, (iii) a qualquer pessoa jurídica em que qualquer destes seja controlador, sócio, acionista ou participante, de forma direta ou indireta, recebimento por palestras, aulas, atuação como proctor de treinamentos, remunerações, honorários pagos por participações em conselhos consultivos, de investigadores, ou outros comitês, etc. Provenientes da indústria farmacêutica, de órteses, próteses, equipamentos e implantes, brasileiras ou estrangeiras: - AstraZeneca.
José Augusto Soares Barreto-Filho	Declaração financeira A - Pagamento de qualquer espécie e desde que economicamente apreciáveis, feitos a (i) você, (ii) ao seu cônjuge/ companheiro ou a qualquer outro membro que resida com você, (iii) a qualquer pessoa jurídica em que qualquer destes seja controlador, sócio, acionista ou participante, de forma direta ou indireta, recebimento por palestras, aulas, atuação como proctor de treinamentos, remunerações, honorários pagos por participações em conselhos consultivos, de investigadores, ou outros comitês, etc. Provenientes da indústria farmacêutica, de órteses, próteses, equipamentos e implantes, brasileiras ou estrangeiras: - Servier: aula sobre avaliação do risco cardiovascular.
José Fernando Vilela-Martin	Nada a ser declarado
Juan Carlos Yugar Toledo	Nada a ser declarado
Leda Aparecida Daud Lotaif	Declaração financeira A - Pagamento de qualquer espécie e desde que economicamente apreciáveis, feitos a (i) você, (ii) ao seu cônjuge/ companheiro ou a qualquer outro membro que resida com você, (iii) a qualquer pessoa jurídica em que qualquer destes seja controlador, sócio, acionista ou participante, de forma direta ou indireta, recebimento por palestras, aulas, atuação como proctor de treinamentos, remunerações, honorários pagos por participações em conselhos consultivos, de investigadores, ou outros comitês, etc. Provenientes da indústria farmacêutica, de órteses, próteses, equipamentos e implantes, brasileiras ou estrangeiras: - AstraZeneca: Forxiga, Lojelma, Fresenius Kabi: Ketosteril.
Lílian Soares da Costa	Nada a ser declarado
Lucelia Batista Neves Cunha Magalhães	Nada a ser declarado
Luciano Ferreira Drager	Declaração financeira A - Pagamento de qualquer espécie e desde que economicamente apreciáveis, feitos a (i) você, (ii) ao seu cônjuge/ companheiro ou a qualquer outro membro que resida com você, (iii) a qualquer pessoa jurídica em que qualquer destes seja controlador, sócio, acionista ou participante, de forma direta ou indireta, recebimento por palestras, aulas, atuação como proctor de treinamentos, remunerações, honorários pagos por participações em conselhos consultivos, de investigadores, ou outros comitês, etc. Provenientes da indústria farmacêutica, de órteses, próteses, equipamentos e implantes, brasileiras ou estrangeiras: - Aché, AstraZeneca, Biolab, Daiichi Sankyo, Lilly e Novo Nordisk: palestras voltadas ao diagnóstico e ao tratamento da Hipertensão Arterial e comorbidades. Outros relacionamentos Financiamento de atividades de educação médica continuada, incluindo viagens, hospedagens e inscrições para congressos e cursos, provenientes da indústria farmacêutica, de órteses, próteses, equipamentos e implantes, brasileiras ou estrangeiras: - AstraZeneca, Lilly e Novo Nordisk.
Luis Cuadrado Martin	Outros relacionamentos Financiamento de atividades de educação médica continuada, incluindo viagens, hospedagens e inscrições para congressos e cursos, provenientes da indústria farmacêutica, de órteses, próteses, equipamentos e implantes, brasileiras ou estrangeiras: - Servier: anti-hipertensivos.
Luiz Aparecido Bortolotto	Declaração financeira A - Pagamento de qualquer espécie e desde que economicamente apreciáveis, feitos a (i) você, (ii) ao seu cônjuge/ companheiro ou a qualquer outro membro que resida com você, (iii) a qualquer pessoa jurídica em que qualquer destes seja controlador, sócio, acionista ou participante, de forma direta ou indireta, recebimento por palestras, aulas, atuação como proctor de treinamentos, remunerações, honorários pagos por participações em conselhos consultivos, de investigadores, ou outros comitês, etc. Provenientes da indústria farmacêutica, de órteses, próteses, equipamentos e implantes, brasileiras ou estrangeiras: - Servier: Triplixam; GSK: vacina. Outros relacionamentos Financiamento de atividades de educação médica continuada, incluindo viagens, hospedagens e inscrições para congressos e cursos, provenientes da indústria farmacêutica, de órteses, próteses, equipamentos e implantes, brasileiras ou estrangeiras: - Servier: Triplixam.
Luiz César Nazário Scala	Nada a ser declarado
Márcia Regina Simas Torres Klein	Nada a ser declarado
Marco Antonio Mota-Gomes	Declaração financeira A - Pagamento de qualquer espécie e desde que economicamente apreciáveis, feitos a (i) você, (ii) ao seu cônjuge/ companheiro ou a qualquer outro membro que resida com você, (iii) a qualquer pessoa jurídica em que qualquer destes seja controlador, sócio, acionista ou participante, de forma direta ou indireta, recebimento por palestras, aulas, atuação como proctor de treinamentos, remunerações, honorários pagos por participações em conselhos consultivos, de investigadores, ou outros comitês, etc. Provenientes da indústria farmacêutica, de órteses, próteses, equipamentos e implantes, brasileiras ou estrangeiras: - Omron: Equipamentos; Biolab: Press Plus; Daiichi Sankyo: Benicar Triplo; AstraZeneca: Forxiga; Ache: Edistride. B - Financiamento de pesquisas sob sua responsabilidade direta/pessoal (direcionado ao departamento ou instituição) provenientes da indústria farmacêutica, de órteses, próteses, equipamentos e implantes, brasileiras ou estrangeiras: - Novartis: Inclisiran; Libbs: Associação Tripla; Servier: Associação Quádrupla.
Marcus Vinicius Bolivar Malachias	Declaração financeira A - Pagamento de qualquer espécie e desde que economicamente apreciáveis, feitos a (i) você, (ii) ao seu cônjuge/ companheiro ou a qualquer outro membro que resida com você, (iii) a qualquer pessoa jurídica em que qualquer destes seja controlador, sócio, acionista ou participante, de forma direta ou indireta, recebimento por palestras, aulas, atuação como proctor de treinamentos, remunerações, honorários pagos por participações em conselhos consultivos, de investigadores, ou outros comitês, etc. Provenientes da indústria farmacêutica, de órteses, próteses, equipamentos e implantes, brasileiras ou estrangeiras: - Dapagliflozina/AstraZeneca: insuficiência cardíaca, diabetes; Finerenona/Bayer: insuficiência cardíaca, diabetes; Empagliflozina/Boehringer-Ingelheim: insuficiência cardíaca, diabetes; Olmesartana/Daiichi-Sankyo: hipertensão; Telmisartana/EMS: hipertensão; Arexvy/GSK: vacinas; Candesartana/Libbs: hipertensão, insuficiência cardíaca; Levanlodipino/Mantecorp: hipertensão; Bisoprolol/Merck: hipertensão, insuficiência cardíaca; Inclisiran/Novartis: insuficiência cardíaca, dislipidemia; Semaglutida/Novo Nordisk: diabetes; Elecsys/Roche: biomarcadores, insuficiência cardíaca, diabetes; Eplerenone/Viatris: insuficiência cardíaca, hipertensão.
Maria Eliane Campos Magalhães	Declaração financeira A - Pagamento de qualquer espécie e desde que economicamente apreciáveis, feitos a (i) você, (ii) ao seu cônjuge/ companheiro ou a qualquer outro membro que resida com você, (iii) a qualquer pessoa jurídica em que qualquer destes seja controlador, sócio, acionista ou participante, de forma direta ou indireta, recebimento por palestras, aulas, atuação como proctor de treinamentos, remunerações, honorários pagos por participações em conselhos consultivos, de investigadores, ou outros comitês, etc. Provenientes da indústria farmacêutica, de órteses, próteses, equipamentos e implantes, brasileiras ou estrangeiras: - AstraZeneca: Forxiga, Breztri, Lokelma.
Maria Eliete Pinheiro	Nada a ser declarado
Maria Emília Figueiredo Teixeira	Nada a ser declarado
Mario Fritsch Toros Neves	Nada a ser declarado
Nelson Dinamarco	Nada a ser declarado
Osni Moreira Filho	Declaração financeira A - Pagamento de qualquer espécie e desde que economicamente apreciáveis, feitos a (i) você, (ii) ao seu cônjuge/ companheiro ou a qualquer outro membro que resida com você, (iii) a qualquer pessoa jurídica em que qualquer destes seja controlador, sócio, acionista ou participante, de forma direta ou indireta, recebimento por palestras, aulas, atuação como proctor de treinamentos, remunerações, honorários pagos por participações em conselhos consultivos, de investigadores, ou outros comitês, etc. Provenientes da indústria farmacêutica, de órteses, próteses, equipamentos e implantes, brasileiras ou estrangeiras: - Servier: hipertensão arterial. Outros relacionamentos Financiamento de atividades de educação médica continuada, incluindo viagens, hospedagens e inscrições para congressos e cursos, provenientes da indústria farmacêutica, de órteses, próteses, equipamentos e implantes, brasileiras ou estrangeiras: - Torrent: hipertensão arterial.
Oswaldo Passarelli Junior	Nada a ser declarado
Otavio Rizzi Coelho	Declaração financeira A - Pagamento de qualquer espécie e desde que economicamente apreciáveis, feitos a (i) você, (ii) ao seu cônjuge/ companheiro ou a qualquer outro membro que resida com você, (iii) a qualquer pessoa jurídica em que qualquer destes seja controlador, sócio, acionista ou participante, de forma direta ou indireta, recebimento por palestras, aulas, atuação como proctor de treinamentos, remunerações, honorários pagos por participações em conselhos consultivos, de investigadores, ou outros comitês, etc. Provenientes da indústria farmacêutica, de órteses, próteses, equipamentos e implantes, brasileiras ou estrangeiras: - Bayer: Finerinone; EMS: Rivaroxabana; AstraZeneca: Dapaglifozina; Libbs: Ticagrelor. Outros relacionamentos Financiamento de atividades de educação médica continuada, incluindo viagens, hospedagens e inscrições para congressos e cursos, provenientes da indústria farmacêutica, de órteses, próteses, equipamentos e implantes, brasileiras ou estrangeiras: - Bayer; Novo Nordisk; AstraZeneca.
Paulo César Brandão Veiga Jardim	Nada a ser declarado
Renault Mattos Ribeiro Júnior	Outros relacionamentos Financiamento de atividades de educação médica continuada, incluindo viagens, hospedagens e inscrições para congressos e cursos, provenientes da indústria farmacêutica, de órteses, próteses, equipamentos e implantes, brasileiras ou estrangeiras: - Novo Nordisk, GSK.
Roberto Dischinger Miranda	Declaração financeira A - Pagamento de qualquer espécie e desde que economicamente apreciáveis, feitos a (i) você, (ii) ao seu cônjuge/ companheiro ou a qualquer outro membro que resida com você, (iii) a qualquer pessoa jurídica em que qualquer destes seja controlador, sócio, acionista ou participante, de forma direta ou indireta, recebimento por palestras, aulas, atuação como proctor de treinamentos, remunerações, honorários pagos por participações em conselhos consultivos, de investigadores, ou outros comitês, etc. Provenientes da indústria farmacêutica, de órteses, próteses, equipamentos e implantes, brasileiras ou estrangeiras: - Aché: Enoxaparina; Daiichi: Benicar; Nutricia: Nutridrink; EMS: Xaquilis; Novo Nordisk: Wegovy; Pfizer: vacinas Prevenar e Arexvy. Participação societária de qualquer natureza e qualquer valor economicamente apreciável de empresas na área de saúde, de ensino ou em empresas concorrentes ou fornecedoras da SBC: - Sócio fundador do Instituto Longevitá (área da saúde).
Rodrigo Bezerra	Nada a ser declarado
Rodrigo Pinto Pedrosa	Nada a ser declarado
Rogério Andrade Mulinari	Declaração financeira B - Financiamento de pesquisas sob sua responsabilidade direta/pessoal (direcionado ao departamento ou instituição) provenientes da indústria farmacêutica, de órteses, próteses, equipamentos e implantes, brasileiras ou estrangeiras: - Tricida, Inc: TRC101 em doença renal crônica.
Rogério Baumgratz de Paula	Nada a ser declarado
Rogério Toshiro Passos Okawa	Declaração financeira A - Pagamento de qualquer espécie e desde que economicamente apreciáveis, feitos a (i) você, (ii) ao seu cônjuge/ companheiro ou a qualquer outro membro que resida com você, (iii) a qualquer pessoa jurídica em que qualquer destes seja controlador, sócio, acionista ou participante, de forma direta ou indireta, recebimento por palestras, aulas, atuação como proctor de treinamentos, remunerações, honorários pagos por participações em conselhos consultivos, de investigadores, ou outros comitês, etc. Provenientes da indústria farmacêutica, de órteses, próteses, equipamentos e implantes, brasileiras ou estrangeiras: - EMS: hipertensão. Outros relacionamentos Financiamento de atividades de educação médica continuada, incluindo viagens, hospedagens e inscrições para congressos e cursos, provenientes da indústria farmacêutica, de órteses, próteses, equipamentos e implantes, brasileiras ou estrangeiras: - Novo Nordisk: obesidade.
Rui Manuel dos Santos Povoa	Nada a ser declarado
Sandra Lia do Amaral Cardoso	Nada a ser declarado
Sayuri Inuzuka	Outros relacionamentos Financiamento de atividades de educação médica continuada, incluindo viagens, hospedagens e inscrições para congressos e cursos, provenientes da indústria farmacêutica, de órteses, próteses, equipamentos e implantes, brasileiras ou estrangeiras: - Servier.
Sebastião Rodrigues Ferreira-Filho	Nada a ser declarado
Sergio Emanuel Kaiser	Declaração financeira A - Pagamento de qualquer espécie e desde que economicamente apreciáveis, feitos a (i) você, (ii) ao seu cônjuge/ companheiro ou a qualquer outro membro que resida com você, (iii) a qualquer pessoa jurídica em que qualquer destes seja controlador, sócio, acionista ou participante, de forma direta ou indireta, recebimento por palestras, aulas, atuação como proctor de treinamentos, remunerações, honorários pagos por participações em conselhos consultivos, de investigadores, ou outros comitês, etc. Provenientes da indústria farmacêutica, de órteses, próteses, equipamentos e implantes, brasileiras ou estrangeiras: - Bayer: Firialta; Novartis: Sybrava; Libbs: Naprix; Daiichi Sankyo: Nustendi e Benicar; Biolab: Repatha e Livalo; Astrazeneca: Selozok e Forxiga; Novo Nordisk: Ozempic e Wegovy: Boehringer Ingelheim: Jardiance. Outros relacionamentos Financiamento de atividades de educação médica continuada, incluindo viagens, hospedagens e inscrições para congressos e cursos, provenientes da indústria farmacêutica, de órteses, próteses, equipamentos e implantes, brasileiras ou estrangeiras: - Bayer: Vericiguat; Novo Nordisk: Wegovy; Daiichi Sankyo: Nustendi; Benicar: Lixiana; Novartis: Sybrava.
Sheyla Cristina Tonheiro Ferro da Silva	Outros relacionamentos Financiamento de atividades de educação médica continuada, incluindo viagens, hospedagens e inscrições para congressos e cursos, provenientes da indústria farmacêutica, de órteses, próteses, equipamentos e implantes, brasileiras ou estrangeiras: - AstraZeneca: Forxiga, Lokelma; Novartis: Entresto, Sybrava; Lilly: Jardiance, Mounjaro; Novo Nordisk: Rybelsus, Wegovy.
Tânia Plens Shecaira	Nada a ser declarado
Vera Hermina Kalika Koch	Nada a ser declarado
Weimar Kunz Sebba Barroso	Declaração financeira A - Pagamento de qualquer espécie e desde que economicamente apreciáveis, feitos a (i) você, (ii) ao seu cônjuge/ companheiro ou a qualquer outro membro que resida com você, (iii) a qualquer pessoa jurídica em que qualquer destes seja controlador, sócio, acionista ou participante, de forma direta ou indireta, recebimento por palestras, aulas, atuação como proctor de treinamentos, remunerações, honorários pagos por participações em conselhos consultivos, de investigadores, ou outros comitês, etc. Provenientes da indústria farmacêutica, de órteses, próteses, equipamentos e implantes, brasileiras ou estrangeiras: - EMS; Brace Pharma; Servier; Biolab; Omron; Cardios. B - Financiamento de pesquisas sob sua responsabilidade direta/pessoal (direcionado ao departamento ou instituição) provenientes da indústria farmacêutica, de órteses, próteses, equipamentos e implantes, brasileiras ou estrangeiras: - Ministério de Saúde; PROADI SUS; Novartis: Entresto. C - Financiamento de pesquisa (pessoal), cujas receitas tenham sido provenientes da indústria farmacêutica, de órteses, próteses, equipamentos e implantes, brasileiras ou estrangeiras. - EMS: Estudo RITMO; Brace Pharma: Estudo BRACE 23. Outros relacionamentos Financiamento de atividades de educação médica continuada, incluindo viagens, hospedagens e inscrições para congressos e cursos, provenientes da indústria farmacêutica, de órteses, próteses, equipamentos e implantes, brasileiras ou estrangeiras: - Servier; Congresso Europeu de Hipertensão Arterial.
Wilson Nadruz	Nada a ser declarado

## Sumário

**1. Introdução** 24
  **1.1. Principais Destaques** 25
**2. Epidemiologia, Definição e Prevenção Primária** 30
  **2.1. Definição, Epidemiologia e Prevenção Primária** 30
    **2.1.1. Definição de Hipertensão Arterial** 30
    **2.1.2. Dados Epidemiológicos e Impacto da Hipertensão Arterial** 30
  **2.3. Fatores de Risco para Hipertensão Arterial** 30
    **2.3.1. Genética** 30
    **2.3.2. Idade e Sexo** 30
    **2.3.3. Etnia, Urbanização e Fatores Socioeconômicos** 30
    **2.3.4. Espiritualidade e Fatores Psicossociais** 31
    **2.3.5. Sobrepeso/Obesidade e Sedentarismo** 31
    **2.3.6. Ingestão de Sódio e Potássio** 31
    **2.3.7. Álcool** 32
    **2.3.8. COVID-19** 32
    **2.3.9. Medicamentos, Drogas Ilícitas e Uso de Esteroides Anabolizantes** 32
    **2.3.10. Distúrbios do Sono** 32
    **2.3.11. Tabagismo** 32
    **2.3.12. Fatores Ambientais** 32
  **2.4. Prevenção Primária** 32
**3. Medida da Pressão Arterial, Diagnóstico e Classificação** 34
  **3.1. Introdução** 34
  **3.2. Medida da Pressão Arterial no Consultório** 34
    **3.2.1. Técnicas de Medida da Pressão Arterial** 34
    **3.2.2. Populações Especiais** 34
    **3.2.3. Classificação** 35
  **3.3. Medida da Pressão Arterial Fora do Consultório** 36
    **3.3.1. Indicações da Medida Ambulatorial da Pressão Arterial e Medida Residencial da Pressão Arterial** 36
  **3.4. Efeito do Avental Branco e Efeito de Mascaramento** 36
  **3.5. Hipertensão do Avental Branco e Hipertensão Mascarada** 37
  **3.6. Hipertensão Mascarada Não Controlada e Hipertensão do Avental Branco Não Controlada** 38
  **3.7. Recomendações para Diagnóstico e Seguiment**o 38
    **3.7.1. Automedida da Pressão Arterial** 38
    **3.7.2. Teste Ergométrico** 39
  **3.8. Pressão Arterial Central, Velocidade de Onda de Pulso e Augmentation Index** 39
  **3.9. Novas Tecnologias para Medidas da Pressão Arterial** 39
**4. Avaliação Clínica e Complementar e Estratificação de Risco Cardiovascular** 41
  **4.1. Estratificação de Risco Adicional (Condições Associadas)** 41
  **4.2. Avaliação Clínica e Complementar** 41
    **4.2.1. Anamnese** 41
    **4.2.2. Exame Físico** 41
    **4.2.3. Exames Complementares** 41
  **4.4. Lesões de Órgãos-alvo** 43
  **4.5. Avaliação de Doença Renal Crônica** 43
  **4.6. Avaliação de Causas Secundárias** 45
  **4.7. Cálculo do Risco Cardiovascular** 45
  **4.8. Aspectos Adicionais e Desafios na Avaliação do Risco Cardiovascular na Hipertensão Arterial** 45
**5. Início de Tratamento e Metas Terapêuticas** 49
  **5.1. Início de Tratamento** 49
  **5.2. Metas de Pressão Arterial** 49
  **5.3. Metas de Pressão Arterial na Medida Ambulatorial da Pressão Arterial e Medida Residencial da Pressão Arterial** 49
  **5.4. Curva em J da Pressão Arterial** 50
  **5.5. Reduções Intensas da Pressão Arterial e Risco de Eventos Adversos** 51
  **5.6. Considerações na Hipertensão Sistólica Isolada** 51
**6. Medidas Não Medicamentosas** 52
  **6.1. Cessação do Tabagismo** 52
  **6.2. Perda de Peso** 52
  **6.3. Modificações na Ingestão Alimentar** 53
    **6.3.1. Restrição na Ingestão De Sódio** 53
    **6.3.2. Aumento no Consumo De Potássio Dietético** 53
    **6.3.3. Redução no Consumo de Álcool** 54
    **6.3.4. Adoção de um Padrão Alimentar Saudável** 54
    **6.3.5. Outras Intervenções na Dieta** 54
  **6.4. Prática de Atividade Física e Exercício Físico** 54
  **6.5. Abordagem de Aspectos Psicoemocionais** 55
    **6.5.1. Técnicas de Abordagem do Estresse** 55
    **6.5.2. Respiração Lenta** 55
    **6.5.3. Espiritualidade e Religiosidade** 55
  **6.6. Equipe Multiprofissional na Adoção das Medidas Não Medicamentosas** 55
  **6.7. Considerações Finais sobre as Medidas Não Medicamentosas** 57
**7. Tratamento Medicamentoso** 57
  **7.1. Objetivos do Tratamento e Racional para o Uso de Medicamentos** 57
    **7.1.1. Insuficiência Cardíaca** 57
    **7.1.2. Acidente Vascular Cerebral** 57
    **7.1.3. Doença Arterial Coronariana** 57
    **7.1.4. Doença Renal Crônica** 58
    **7.1.5. Morte Cardiovascular** 58
  **7.2. Tratamento Medicamentoso** 58
  **7.3. Princípios Gerais do Tratamento Medicamentoso** 59
  **7.4. Classes dos Medicamentos Utilizados no Tratamento da Hipertensão Arterial** 63
  **7.5. Medicamentos Indicados para Outras Doenças e que Têm Ação sobre a Pressão Arterial e Eventos Cardiovasculares, Porém Ainda Não Consolidados Como Rotina no Tratamento da Hipertensão Arterial** 63
    **7.5.1. Sacubitril/Valsartana** 63
    **7.5.2. Inibidores de Cotransportador de Sódio-Glicose Tipo 2** 64
    **7.5.3. Agonistas de Peptídeo Semelhante a Glucagon 1 e os Novos Antagonistas dos Receptores de Mineralocorticoides Não Hormonais Seletivos** 65
  **7.6. Estratégias de Tratamento** 65
    **7.6.1. Monoterapia** 65
    **7.6.2. Associação de Medicamentos** 66
**8. Hipertensão Arterial E Condições Clínicas Associadas: Doença Arterial Coronariana, Doença Renal Crônica, Diabetes Mellitus, Obesidade, COVID-19, Pós-Acidente Vascular Cerebral E Insuficiência Cardíaca** 68
  **8.1. Hipertensão Arterial e Doença Arterial Coronariana** 68
    **8.1.1. Metas de Pressão Arterial na Doença Arterial Coronariana** 68
    **8.1.2. Tratamento da Hipertensão Arterial e Doença Arterial Coronariana** 68
  **8.2. Hipertensão Arterial na Doença Renal Crônica em Pacientes sob Tratamento Conservador e na Diálise** 69
    **8.2.1. Meta de Pressão Arterial para Doença Renal Crônica em Tratamento Conservador** 69
    **8.2.2. Meta de Pressão Arterial para Doença Renal Crônica em Diálise** 70
    **8.2.3. Tratamento da hipertensão arterial na doença renal crônica em tratamento conservador** 70
    **8.2.4. Tratamento da Hipertensão Arterial na Doença Renal Crônica em Diálise** 71
  **8.3. Hipertensão Arterial e Diabetes Mellitus** 71
    **8.3.1. Metas na Hipertensão Arterial no Paciente com Diabetes Mellitus** 72
    **8.3.2. Tratamento da Hipertensão Arterial no Paciente com Diabetes Mellitus** 72
  **8.4. Hipertensão Arterial e Obesidade** 73
    **8.4.1. Meta de Pressão Arterial em Pacientes com Obesidade** 73
    **8.4.2. Tratamento da Hipertensão Arterial na Obesidade** 73
  **8.5. Hipertensão Arterial e COVID-19** 74
  **8.6. Hipertensão Arterial, Acidente Vascular Cerebral e Déficit Cognitivo** 75
    **8.6.1. Meta de Pressão Arterial na Hipertensão Arterial do Acidente Vascular Cerebral na Fase Crônica** 75
    **8.6.2. Tratamento da Hipertensão Arterial no Acidente Vascular Cerebral na Fase Crônica** 75
  **8.7. Hipertensão Arterial e Insuficiência Cardíaca com Fração de Ejeção Preservada e com Fração de Ejeção Reduzida** 76
**9. Hipertensão Arterial em Idosos, Crianças e Adolescentes** 78
  **9.1. Hipertensão Arterial no Idoso** 78
    **9.1.1. Introdução** 78
    **9.1.2. Mecanismos Fisiopatológicos** 78
    **9.1.3. Diagnóstico e Decisão Terapêutica** 78
      **9.1.3.1. Diagnóstico** 78
      **9.1.3.2. Avaliação do Status Funcional e Fragilidade** 79
      **9.1.3.3. Avaliação da Função Cognitiva** 79
      **9.1.3.4. Decisão Terapêutica** 79
    **9.1.4. Metas Pressóricas e Tratamento** 79
      **9.1.4.1. Metas para o Controle da Hipertensão Arterial no Paciente Idoso** 79
      **9.1.4.2. Tratamento Não Medicamentoso** 80
      **9.1.4.3. Tratamento Medicamentoso** 81
      **9.1.4.4. Polifarmácia e Adesão** 81
      **9.1.4.5. Desintensificação e Desprescrição** 81
  **9.2. Hipertensão Arterial na Criança e no Adolescente** 82
**10. Hipertensão Arterial em Mulheres** 91
  **10.1. Hipertensão Arterial em Mulheres Jovens e na Menopausa** 91
    **10.1.1. Prevalência** 91
    **10.1.2. Fatores Associados a Hipertensão Arterial em Mulheres** 91
      **10.1.2.1. Mulheres Jovens e Anticoncepcionais** 91
      **10.1.2.2. Hipertensão, Função Sexual e Fertilidade na Mulher** 91
      **10.1.2.3. Menopausa** 91
      **10.1.2.4. Síndrome dos Ovários Policísticos** 92
    **10.1.3. Repercussões em Órgãos-Alvo e Comorbidades em Mulheres** 92
    **10.1.4. Tratamento da Hipertensão nas Mulheres** 92
  **10.2. Hipertensão na Gestação** 93
    **10.2.1. Classificação** 93
    **10.2.2. Predição e Profilaxia de Pré-eclâmpsia** 93
    **10.2.3. Abordagem Terapêutica da Hipertensão na Gestação** 93
    **10.2.4. Hipertensão Arterial no Puerpério** 95
    **10.2.5. Risco Cardiovascular Futuro** 95
**11. Crise Hipertensiva** 98
  **11.1. Definição e Classificação** 98
  **11.2. Classificação** 98
  **11.3. Aspectos Epidemiológicos e Prognósticos** 98
    **11.3.1. Epidemiologia** 98
    **11.3.2. Prognóstico** 98
  **11.4. Investigação Clínico-laboratorial Complementar** 98
  **11.5. Tratamento Geral da Crise Hipertensiva** 98
  **11.6. Emergências Hipertensivas em Situações Especiais** 99
    **11.6.1. Encefalopatia Hipertensiva** 9 9
    **11.6.2. Acidente Vascular Cerebral** 100
      **11.6.2.1. Acidente Vascular Cerebral Isquêmico** 102
      **11.6.2.2. Acidente Vascular Cerebral Hemorrágico** 102
    **11.6.3. Síndromes Coronarianas Agudas** 102
    **11.6.4. Edema Agudo de Pulmão** 102
    **11.6.5. Dissecção Aguda de Aorta** 103
    **11.6.6. Crises Adrenérgicas** 103
    **11.6.7. Hipertensão Acelerada/Maligna** 103
    **11.6.8. Hipertensão com Múltiplos Danos aos Órgãos-Alvo** 103
    **11.6.9. Hipertensão na Gravidez: Pré-Eclâmpsia e Eclâmpsia** 103
**12. Hipertensão Arterial Resistente e Refratária** 105
  **12.1. Definição e Classificação** 105
  **12.2. Epidemiologia da Hipertensão Arterial Resistente e Refratária** 105
  **12.3. Fisiopatologia** 105
  **12.4. Avaliação Diagnóstica** 105
    **12.4.1. Falha na Técnica de Medida da Pressão Arterial** 105
    **12.4.2. Efeito do Avental Branco** 107
    **12.4.3. Inércia Terapêutica** 107
    **12.4.4. Falta de Adesão Medicamentosa** 107
  **12.5. Abordagem Clínica** 107
  **12.6. Tratamento** 108
    **12.6.1. Tratamento Não Medicamentoso** 108
    **12.6.2. Tratamento Medicamentoso** 108
    **12.6.3. Perspectivas** 109
    **12.6.4. Denervação Renal** 109
**13. Adesão ao Tratamento Anti-hipertensivo** 111
  **13.1. Mensuração da Adesão** 111
  **13.2. Evidências** 112
  **13.3. Estratégias** 112
  **13.4 Recomendações** 114
**14. Melhores Práticas no Cuidado às Pessoas com Hipertensão Arterial na Atenção Primária à Saúde do Sistema Único de Saúde** 114
  **14.1. Sistema Único de Saúde e sua Importância no Controle da Hipertensão Arterial** 114
    **14.1.1. Atenção Primária à Saúde** 114
    **14.1.2. Atenção Especializada** 112
  **14.2. Busca Ativa por Pessoas com Hipertensão Arterial** 116
  **14.3. Protocolo para a Aferição da Pressão Arterial: Cuidados Necessários** 116
  **14.4. Diagnóstico de Hipertensão Arterial** 116
  **14.5. Avaliação Clínica e Complementar do Indivíduo com Hipertensão Arterial** 118
  **14.6. Classificação de Risco após a Avaliação Clínica e Complementar** 118
  **14.7. Metas do Controle Pressórico** 119
  **14.8. Medida da Pressão Arterial Fora do Consultório ou do Ambiente de Cuidado à Saúde** 119
  **14.9. Bases do Tratamento da Pessoa com Hipertensão Arterial** 121
  **14.10. Tratamento com Medicamentos** 122
  **14.11. Condições Clínicas que Justificam o Encaminhamento a Especialista(s)** 124
  **14.12. Contrarreferência** 124
  **14.13. Telemedicina na Linha de Cuidado da Hipertensão Arterial no Âmbito do Sistema Único de Saúde** 124
**15. Perspectivas** 124
  **15.1. Expossoma e Hipertensão Arterial** 124
  **15.2. Wearables e Telemonitoramento em Hipertensão** 128
  **15.3. Hipertensão Arterial e Complacência Intracraniana** 132
  **15.4. Combinações de Três ou Quatro Medicamentos em Doses Ultrabaixas** 132
  **15.5. Novos Medicamentos para o Tratamento da Hipertensão Arterial** 133
**Agradecimentos** 133
**Referências** 134

## 1. Introdução

A Diretriz Brasileira de Hipertensão Arterial 2025 (DBHA 2025) é uma realização conjunta da Sociedade Brasileira de Cardiologia (SBC), da Sociedade Brasileira de Nefrologia (SBN) e da Sociedade Brasileira de Hipertensão (SBH).

Este documento reúne as recomendações mais atualizadas e baseadas nas melhores evidências disponíveis para o manejo da hipertensão arterial (HA), abrangendo aspectos diagnósticos, de acompanhamento e terapêuticos, em diferentes subgrupos de pacientes e condições clínicas associadas, além de situações de maior gravidade, como as emergências hipertensivas (EHs) e os casos de HA resistente e refratária (Figura Central).

**Figure:**
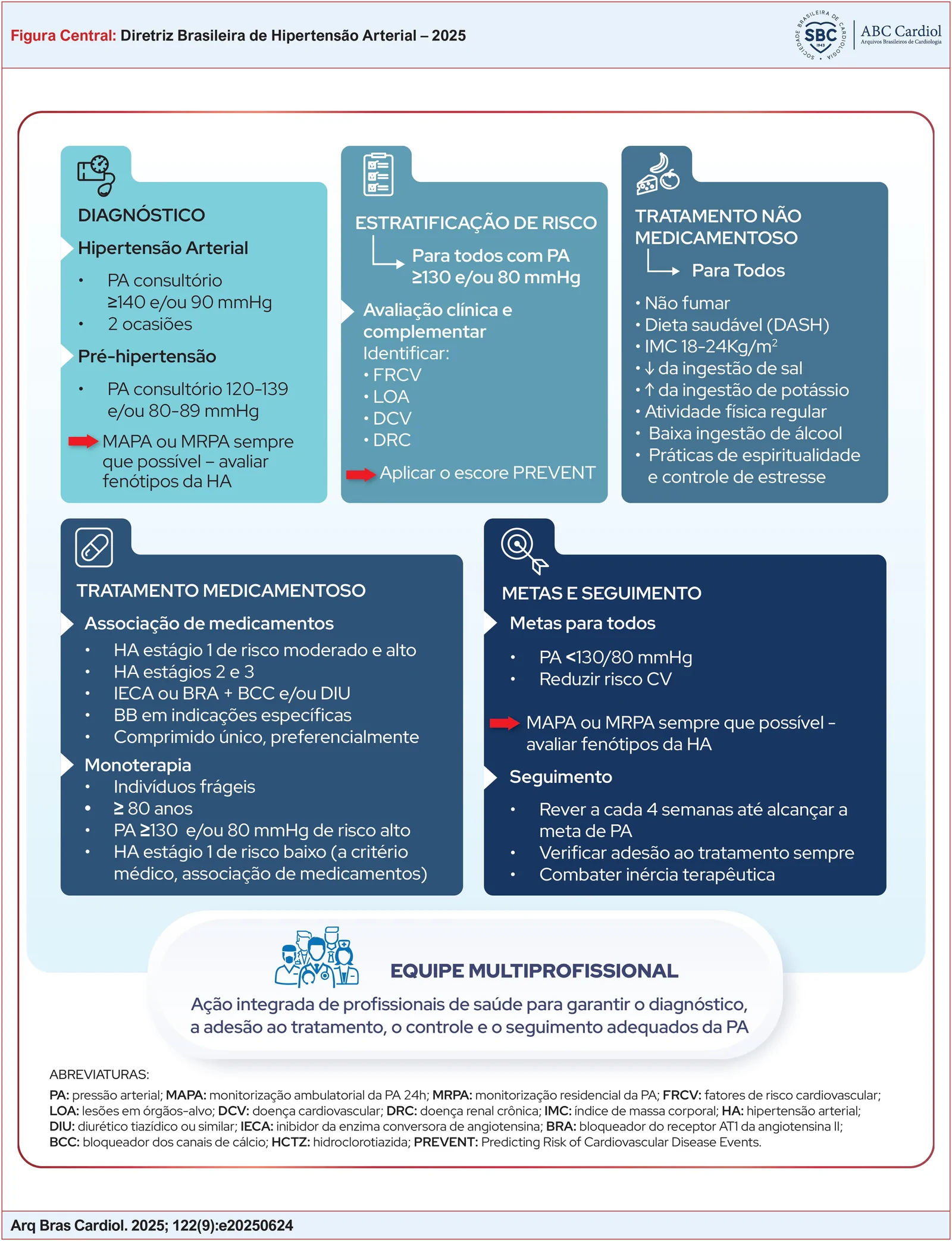


Um dos principais desafios enfrentados na elaboração desta nova diretriz foi atender às Normas para Elaboração de Diretrizes, Recomendações e Posicionamentos da SBC. Conforme essas normas, a classificação das recomendações deve basear-se na metodologia *Grading of Recommendations Assessment, Development and Evaluation* (GRADE), que estabelece a força das recomendações e o nível de certeza das evidências. Contamos, para essa análise, com o apoio incansável do metodologista e cardiologista Dr. Leonardo Luna, a quem agradecemos por seus ensinamentos e valiosas orientações ao longo de todo o processo.

Destacamos que, pela primeira vez, essa diretriz inclui um capítulo inteiramente dedicado às especificidades do manejo da hipertensão no Sistema Único de Saúde (SUS), tema de grande relevância, considerando-se que cerca de 75% dos pacientes com HA no Brasil são tratados no SUS, especialmente na atenção primária à saúde.

Um documento com o porte da DBHA 2025, com 154 páginas, só pôde ser desenvolvido graças ao envolvimento de inúmeros médicos de diferentes especialidades e profissionais de saúde, todos profundamente comprometidos com o cuidado ao paciente hipertenso. A eles, registramos nossa sincera gratidão pelas contribuições valiosas e competentes.

Em seguida, destacaremos aproximadamente 30% das recomendações da DBHA 2025 – aquelas consideradas de maior relevância ou que representam novidades em relação à edição anterior.

Esperamos que aproveitem a leitura e que compartilhem amplamente este conteúdo, para que, juntos, possamos fortalecer o cuidado de qualidade ao paciente com HA em nosso país.

**Andréa Araujo Brandão–**

Coordenadora Geral

**Wilson Nadruz**

Sociedade Brasileira de Cardiologia

**Cibele Isaac Saad Rodrigues**

Sociedade Brasileira de Nefrologia

**Luiz Aparecido Bortolotto**

Sociedade Brasileira de Hipertensão

### 1.1. Principais Destaques

**Capítulo 3:** Medida da pressão arterial, diagnóstico e classificação

Recomendações para medida da pressão arterial, diagnóstico e classificação	Forca da recomendação	Certeza da evidência
Recomenda-se classificar a pré-hipertensão abrangendo valores de PAS entre 120-139 mmHg ou PAD entre 80-89 mmHg no consultório com o objetivo de identificar precocemente indivíduos em risco e incentivar intervenções mais proativas e não medicamentosas para prevenir a progressão para HA.	FORTE	MODERADA
Recomenda-se que o diagnóstico de HA seja feito quando a PA no consultório for ≥ 140 e/ou 90 mmHg em duas ocasiões diferentes e classificada em estágios 1, 2 e 3, de acordo com o maior valor de PAS ou PAD.	FORTE	MODERADA
Recomenda-se utilizar equipamento automático de braço com o objetivo de reduzir erros e facilitar a obtenção da medida da PA.	FORTE	MODERADA
Recomenda-se usar MAPA ou MRPA para confirmar diagnóstico de HA e monitorar o tratamento.	FORTE	ALTA

HA: hipertensão arterial; MAPA: monitorização ambulatorial da pressão arterial; MRPA: monitorização residencial da pressão arterial; PA: pressão arterial; PAD: pressão arterial diastólica; PAS: pressão arterial sistólica.

**Capítulo 4:** Avaliação clínica e complementar, e estratificação de risco cardiovascular

Recomendações para avaliação clínica e complementar, e estratificação de risco cardiovascular	Forca da recomendação	Certeza da evidência
Recomenda-se pesquisar FRCV e LOA idealmente em todos os pacientes no momento do diagnóstico de HA e repetir essa avaliação pelo menos anualmente, sendo a escolha do método decidida conforme os recursos disponíveis.	FORTE	BAIXA
Recomenda-se avaliar o risco CV utilizando o escore PREVENT.	FORTE	ALTA
Recomenda-se estratificar o risco CV em indivíduos com pré-hipertensão com o objetivo de orientar o início do tratamento anti-hipertensivo e promover um controle mais adequado dos FRCV.	FORTE	ALTA
Recomenda-se estratificar o risco CV em pacientes com HA com o objetivo de promover uma abordagem mais precisa e personalizada no manejo de medicamentos e direcionamento de metas para controle dos FRCVs.	FORTE	ALTA

CV: cardiovascular; FRCVs: fatores de risco cardiovascular; HA: hipertensão arterial; LOA: lesão de órgão-alvo; PREVENT: *Predicting Risk of Cardiovascular Disease Events*.

**Capítulo 5:** Início de tratamento e metas terapêuticas

Recomendações para início do tratamento anti-hipertensivo	Força da recomendação	Certeza da evidência
Recomendam-se MNM para todos os indivíduos com PA ≥ 120/80 mmHg.	FORTE	ALTA
Recomenda-se tratamento medicamentoso após 3 meses de MNM para indivíduos com PA 130-139/80-89 mmHg e alto risco CV.	FORTE	ALTA
Recomenda-se o início de tratamento medicamentoso para indivíduos com PA ≥ 140/90 mmHg.	FORTE	ALTA

CV: cardiovascular; MNM: medidas não medicamentosas; PA: pressão arterial.

**Table:** 

Recomendações para metas terapêuticas	Força da recomendação	Certeza da evidência
Recomenda-se a meta de PA < 130/80 mmHg para pacientes com PA 130-139/80-89 mmHg e risco CV alto.	FORTE	ALTA
Recomenda-se a meta de PA < 130/80 mmHg para os pacientes com HA independentemente de o risco CV ser baixo, moderado ou alto.	FORTE	ALTA
Recomenda-se que, para pacientes que não tolerem a meta de PA < 130/80 mmHg, deve-se reduzir a PA até o valor mais baixo tolerado.	FORTE	MODERADA
Confirmar o alcance da meta de PA com a medida da PA fora do consultório por meio da MAPA ou MRPA.	FORTE	BAIXA

CV: cardiovascular; HA: hipertensão arterial; MAPA: monitorização ambulatorial da pressão arterial; MRPA: monitorização residencial da pressão arterial; PA: pressão arterial.

**Capítulo 6:** Medidas não medicamentosas

Recomendações para medidas não medicamentosas	Força da recomendação	Certeza da evidência
Recomenda-se diminuição do peso corporal para redução da PA e de mortalidade em pacientes com obesidade.	FORTE	ALTA
Recomenda-se redução da ingestão de sódio e aumento da ingestão de potássio dietético (exceto para pacientes com DRC) para redução da PA.	FORTE	ALTA
Recomendam-se a dieta DASH e a prática de AF moderada para redução da PA.	FORTE	ALTA

DASH: Dietary Approaches to Stop Hypertension; DRC: doença renal crônica; PA: pressão arterial; AF: atividade física.

**Capítulo 7:** Tratamento medicamentoso

Recomendações para o tratamento medicamentoso	Força da recomendação	Certeza da evidência
Recomenda-se a associação de medicamentos, preferencialmente em comprimido único e com as classes preferenciais, para atingir metas rigorosas de PA (< 130/80 mmHg) e reduzir eventos CV e renais.	FORTE	MODERADA
Recomenda-se monoterapia para indivíduos com PA 130-139/80-89 mmHg e alto risco CV; pacientes com HA estágio 1 de baixo risco (a critério médico, pode-se optar por associação); em indivíduos frágeis; muito idosos (≥ 80 anos) ou com hipotensão ortostática sintomática, especialmente em indivíduos idosos.	FRACA	BAIXA
Recomenda-se, para a maioria dos pacientes, o início do tratamento da HA com associação dupla de medicamentos, em doses baixas, preferencialmente em comprimido único, se disponível.	FORTE	MODERADA
Recomenda-se o uso de diuréticos tiazídicos ou similares, IECA ou BRA e BCC como classes preferenciais para o tratamento da HA e redução de eventos CV e renais importantes.	FORTE	ALTA
Recomenda-se o uso de BB para o tratamento de HA em situações específicas: IC, FA, arritmias, DAC, HA em pacientes em HD e outras (enxaqueca, tremor essencial, mulheres que desejam engravidar, varizes esofágicas).	FORTE	MODERADA
Recomenda-se o uso de espironolactona (ou eplerenona, caso ocorra intolerância à espironolactona) para atingir metas rigorosas de PA (< 130/80 mmHg) e reduzir eventos CV e renais quando não tiverem sido atingidas apenas com três classes iniciais (HAR e HARf).	FORTE	ALTA

BB: betabloqueadores; BCC: bloqueadores dos canais de cálcio; BRA: bloqueadores dos receptores de angiotensina; CV: cardiovascular; DAC: doença arterial coronariana; FA: fibrilação atrial; HA: hipertensão arterial; HAR: hipertensão arterial resistente; HARf: hipertensão arterial refratária; HD: hemodiálise; IC: insuficiência cardíaca; IECA: inibidores da enzima conversora de angiotensina; PA: pressão arterial.

**Capítulo 8:** Hipertensão arterial e condições clínicas associadas: doença arterial coronariana, doença renal crônica, diabetes mellitus, obesidade, COVID-19, pós-acidente vascular cerebral e insuficiência cardíaca

Recomendações para pacientes com hipertensão arterial e doença arterial coronariana	Força da recomendação	Certeza da evidência
Recomenda-se a meta de PA < 130/80 mmHg para indivíduos com HA e DAC. Admite-se que reduzir a PA até 120/70 mmHg não aumenta o risco CV e que pacientes com PA < 120/70 mmHg e assintomáticos não necessitam parar ou reduzir a medicação anti-hipertensiva.	FORTE	MODERADA

CV: cardiovascular; HA: hipertensão arterial; DAC: doença arterial coronariana; PA: pressão arterial.

**Table:** 

Recomendações para pacientes com hipertensão arterial e doença renal crônica em tratamento conservador	Força da recomendação	Certeza da evidência
Recomenda-se, em adultos com HA e DRC ou DM, a meta de PA < 130/80 mmHg para redução de eventos CV e falência renal.	FORTE	ALTA
Recomenda-se que a escolha de anti-hipertensivos na HA da DRC deva incluir, a menos que contraindicado, o uso de IECA ou BRA, e favorecer a introdução e manutenção de medicamentos com efeito anti-hipertensivo discreto, mas que exibam propriedades nefro e cardioprotetoras, como a finerenona e os aGLP1 em pacientes com DM e os iSGLT2, devido ao comprovado efeito benéfico na proteção cardiorrenal.	FORTE	ALTA

aGLP1: análogos dos peptídeos semelhantes ao glucagon 1 (Glucagon-like peptide-1); BRA: bloqueadores dos receptores de angiotensina; DM: diabetes mellitus; DRC: doença renal crônica; HA: hipertensão arterial; IECA: inibidores da enzima conversora de angiotensina; iSGLT2: inibidores do cotransportador de sódio-glicose 2 (sodium/glucose cotransporter 2 inhibitors); PA: pressão arterial.

**Table:** 

Recomendações para pacientes com hipertensão arterial no paciente com diabetes mellitus	Força da recomendação	Certeza da evidência
Recomenda-se que o início do tratamento medicamentoso deva ser imediato (ao diagnóstico) com dois medicamentos, IECA ou BRA, quando houver intolerância ao IECA, associado a BCC ou diurético tiazídico similar.	FORTE	ALTA

BCC: bloqueadores dos canais de cálcio; BRA: bloqueadores dos receptores de angiotensina; IECA: inibidores da enzima conversora de angiotensina.

**Table:** 

Recomendações para pacientes com hipertensão arterial no paciente com obesidade	Força da recomendação	Certeza da evidência
Recomenda-se a meta de PA < 130/80 mmHg para pacientes com obesidade.	FORTE	MODERADA
Recomenda-se o uso de medicamentos para diminuir peso, tais como os aGLP1 (liraglutida, semaglutida e dulaglutida), que apresentam bons resultados na redução de peso e do risco CV, mas os resultados na redução da PA são modestos.	FORTE	ALTA

aGLP1: análogos dos peptídeos semelhantes ao glucagon 1 (Glucagon-like peptide-1); CV: cardiovascular; PA: pressão arterial.

**Table:** 

Recomendações para pacientes com hipertensão arterial e acidente vascular cerebral e déficit cognitivo	Força da recomendação	Certeza da evidência
Recomenda-se a meta de PA < 130/80 mmHg para pacientes com AVC ou AIT prévios para redução de eventos CV maiores (IAM, recorrência AVC, morte)	FORTE	ALTA

AIT: ataque isquêmico transitório; AVC: acidente vascular cerebral; CV: cardiovascular; IAM: infarto agudo do miocárdio; PA: pressão arterial.

**Table:** 

Recomendações para pacientes com hipertensão arterial e insuficiência cardíaca	Força da recomendação	Certeza da evidência
Recomenda-se a meta de PA < 130/80 mmHg para os pacientes com HA, ICFEp e ICFEr.	FRACA	BAIXA
Recomenda-se, para pacientes com ICFEr e HA, o tratamento com medicações que tenham ação anti-hipertensiva e que melhorem o prognóstico: BB, IECA (ou BRA) e ARM.	FORTE	ALTA

ARM: antagonista dos receptores mineralocorticoides; BB: betabloqueadores; BRA: bloqueadores dos receptores de angiotensina; HA: hipertensão arterial; ICFEp: insuficiência cardíaca com fração de ejeção preservada; ICFEr: insuficiência cardíaca com fração de ejeção reduzida; IECA: inibidores da enzima conversora de angiotensina; PA: pressão arterial.

**Capítulo 9:** Hipertensão arterial em idosos, crianças e adolescentes

Recomendações para abordagem da HA em pacientes idosos	Força da recomendação	Certeza da evidência
Recomenda-se a meta de PA < 130/80 mmHg para a maioria dos pacientes idosos.	FORTE	ALTA
Recomenda-se, para idosos frágeis, muito idosos ou com condições que comprometam a expectativa de vida, que a PA seja reduzida até o menor valor tolerado.	FORTE	ALTA
Recomenda-se, nos idosos sob polifarmácia, a revisão periódica de cada um dos medicamentos em uso, a avaliação de eventos adversos e que o tratamento anti-hipertensivo possua o menor número possível de comprimidos ao dia, com a utilização de anti-hipertensivos em combinações em único comprimido de dose única diária, além da ênfase às MNM.	FORTE	MODERADA

PA: pressão arterial; MNM: medidas não medicamentosas.

**Table:** 

Recomendações para abordagem da HA em crianças e adolescentes	Força da recomendação	Certeza da evidência
Recomenda-se início de terapêutica medicamentosa em crianças e adolescentes com IECA, BRA, BCC de ação prolongada ou um diurético tiazídico para pacientes com HA sintomática, presença de LOA, HA estágio 2 sem causa modificável aparente ou HA persistente não responsiva a MNM, se a PA for ≥ percentil 95 ou ≥ 130/80 mmHg em adolescentes ≥ 13 anos de idade.	FORTE	BAIXA

BCC: bloqueadores dos canais de cálcio; BRA: bloqueadores dos receptores de angiotensina; HA: hipertensão arterial; IECA: inibidores da enzima conversora de angiotensina; LOA: lesão de órgão-alvo; MNM: medidas não medicamentosas; PA: pressão arterial.

**Capítulo 10:** Hipertensão arterial em mulheres

Recomendações para abordagem da HA em mulheres dentro e fora do período gestacional	Força da recomendação	Certeza da evidência
Recomenda-se monitorar a PA em mulheres jovens antes da prescrição de anticoncepcionais e após o início do uso, bem como reforçar a importância de medidas regulares da PA (a cada 6 meses), incluindo medidas domiciliares e o controle dos demais FRCVs.	FORTE	MODERADA
Recomenda-se, para o tratamento medicamentoso da HA na mulher durante e após a menopausa, iniciar com a combinação de bloqueador do SRAA com BCC ou diurético tiazídico.	FORTE	MODERADA
Recomendam-se metas de PA iguais para mulheres e homens, assim como a utilização das mesmas classes de anti-hipertensivos para o tratamento da HA em mulheres e homens.	FORTE	MODERADA
Recomenda-se iniciar o tratamento anti-hipertensivo em gestantes com HA com metildopa ou BCCs di-hidropiridínicos (nifedipina de longa duração ou amlodipina).	FORTE	BAIXA
Recomenda-se o acompanhamento de longo prazo de toda mulher que teve HA na gestação para a prevenção efetiva de DCV.	FORTE	BAIXA

BCCs: bloqueadores dos canais de cálcio; DCV: doença cardiovascular; FRCVs: fatores de risco cardiovascular; HA: hipertensão arterial; PA: pressão arterial; SRAA: sistema renina-angiotensina-aldosterona.

**Capítulo 11:** Crise hipertensiva

Recomendações para abordagem da crise hipertensiva	Força da recomendação	Certeza da evidência
Recomenda-se, em pacientes com elevação importante da PA sem LOA aguda (antes denominada urgência hipertensiva), reavaliação ambulatorial em 1 a 7 dias com alvo de PAS < 160 e PAD < 100 mmHg.	FRACA	BAIXA
Recomenda-se que o paciente com EH deva ser admitido em UTI, usar anti-hipertensivos IV com monitorização da PA e observação da progressão da LOA.	FORTE	MODERADA

EH: emergências hipertensivas; IV: intra-venoso; LOA: lesão de órgão-alvo; PA: pressão arterial; PAD: pressão arterial diastólica; PAS: pressão arterial sistólica; UTI: unidade de terapia intensiva.

**Capítulo 12:** Hipertensão arterial resistente e refratária

Recomendações para abordagem da hipertensão resistente e refratária	Força da recomendação	Certeza da evidência
Recomenda-se a realização preferencial de MAPA para confirmação diagnóstica de HAR e HARf, se disponível, ou MRPA.	FORTE	MODERADA
Recomenda-se a meta de PA < 130/80 mmHg para pacientes com HAR ou HARf.	FRACA	BAIXA

HAR: hipertensão arterial resistente; HARf: hipertensão arterial refratária; MAPA: monitorização ambulatorial da pressão arterial; MRPA: monitorização residencial da pressão arterial; PA: pressão arterial.

**Capítulo 13:** Adesão ao tratamento anti-hipertensivo

Recomendações para adesão ao tratamento anti-hipertensivo	Força da recomendação	Certeza da evidência
Recomendam-se estratégias envolvendo a atuação de equipe multiprofissional, subsídios, recursos de comunicação/educação e aplicativos para promover melhorias na adesão ao tratamento anti-hipertensivo.	FRACA	BAIXA

## 2. Epidemiologia, Definição e Prevenção Primária

### 2.1. Definição, Epidemiologia e Prevenção Primária

#### 2.1.1. Definição de Hipertensão Arterial

HA é uma doença crônica não transmissível definida por elevação persistente da pressão arterial (PA) sistólica (PAS) maior ou igual a 140 mmHg e/ou da PA diastólica (PAD) maior ou igual a 90 mmHg, medida com a técnica correta, em pelo menos duas ocasiões e na ausência de medicação anti-hipertensiva. Trata-se de uma condição multifatorial, dependente de fatores genéticos/epigenéticos, ambientais e psicossociais. A definição de HA tem se consolidado ao longo dos anos e foi mantida nos termos já descritos na DBHA de 2020.^1^

#### 2.1.2. Dados Epidemiológicos e Impacto da Hipertensão Arterial

Os principais levantamentos epidemiológicos de HA no Brasil são derivados da Vigilância de Fatores de Risco e Proteção para Doenças Crônicas por Inquérito Telefônic (VIGITEL) e da Pesquisa Nacional de Saúde, ambos do Ministério da Saúde. O VIGITEL iniciou em 2006 e ocorre anualmente, por meio de entrevistas telefônicas com pessoas a partir de 18 anos de idade, moradoras das capitais dos 26 estados brasileiros e do Distrito Federal.^2^ A Pesquisa Nacional de Saúde é um inquérito de saúde por questionários, de base domiciliar e âmbito nacional, realizada em 2013 e 2019.^3^ A principal limitação desses levantamentos, especialmente da Pesquisa Nacional de Saúde de 2019 e do VIGITEL, está no fato de o diagnóstico de HA ser autorreferido, o que pode subestimar sobremaneira sua prevalência. No entanto, esses inquéritos fornecem uma visão abrangente e evolutiva da HA no país.

A análise de dados do VIGITEL entre 2006 e 2019 mostrou que a prevalência de HA em adultos foi de 24,5% em média, mantendo-se estável no período.^4^ Após a pandemia de COVID-19, dados do VIGITEL 2023 mostraram tendência de crescimento na prevalência de HA, alcançando 27,9% (intervalo de confiança [IC] 95% 26,6–29,2). A capital estadual com a menor prevalência foi São Luís (19,2%; IC 95% 15,7–22,7), e a maior foi Rio de Janeiro (34,4%; IC 95% 29,8–39,0).^4^

Em 2023, a Organização Mundial de Saúde (OMS) publicou projeções globais para HA, tendo utilizado dados brasileiros referentes a 2019 bem mais alarmantes, estimando a prevalência global de 45% dos brasileiros entre 30 e 70 anos de idade, sendo 42% das mulheres e 48% dos homens. Nessa estimativa, 67% dos indivíduos com HA no país estariam diagnosticados, 62% receberiam tratamento, e apenas 33% estariam com a pressão controlada.^5^ Entretanto, nessa publicação da OMS não está especificada qual amostra da população brasileira foi avaliada. Por outro lado, análises de amostras populacionais brasileiras urbanas, em que o diagnóstico de HA incluiu a medida da PA, têm demonstrado uma prevalência média de HA em torno de 36%.^6^

A avaliação evolutiva da Pesquisa Nacional de Saúde demonstra uma melhora no autocuidado com a medida da PA. No total, 3% (IC 95% 2,7–3,2) dos adultos brasileiros referiram nunca ter aferido a PA em 2013, com redução para 2% (IC 95% 1,8–2,2) em 2019. O diagnóstico de HA pela Pesquisa Nacional de Saúde teve tendência crescente, de 21,4% (IC 95% 20,8–22) em 2013 para 23,9% (IC 95% 23,4–24,4) em 2019. Há dados favoráveis a respeito do impacto da HA sobre a saúde, com tendência a decréscimo das limitações às atividades habituais em decorrência da doença, de 12,1% (IC 95% 11,2–13,1) em 2013 para 9,8% (IC 95% 9,1–10,5) em 2019.^3^

No Brasil e no mundo, as doenças cardiovasculares (DCVs) são a principal causa de morte, e a HA é o principal fator de risco responsável por elas.^7,8^ Nesse contexto, dados do *Global Burden of Cardiovascular Diseases and Risks* para o Brasil demonstram que a HA foi o principal fator de risco responsável pela mortalidade total no país (104,8 por 100 mil) em 2019.^7^ Também em 2019, a OMS estimou que 54% das mortes por causa cardiovascular (CV) no Brasil seriam atribuídas à HA.^5^ De forma preocupante, o número de mortes por DCV associadas à HA tem mostrado uma tendência de crescimento no país (Figura 2.1).

**Figura 2.1:**
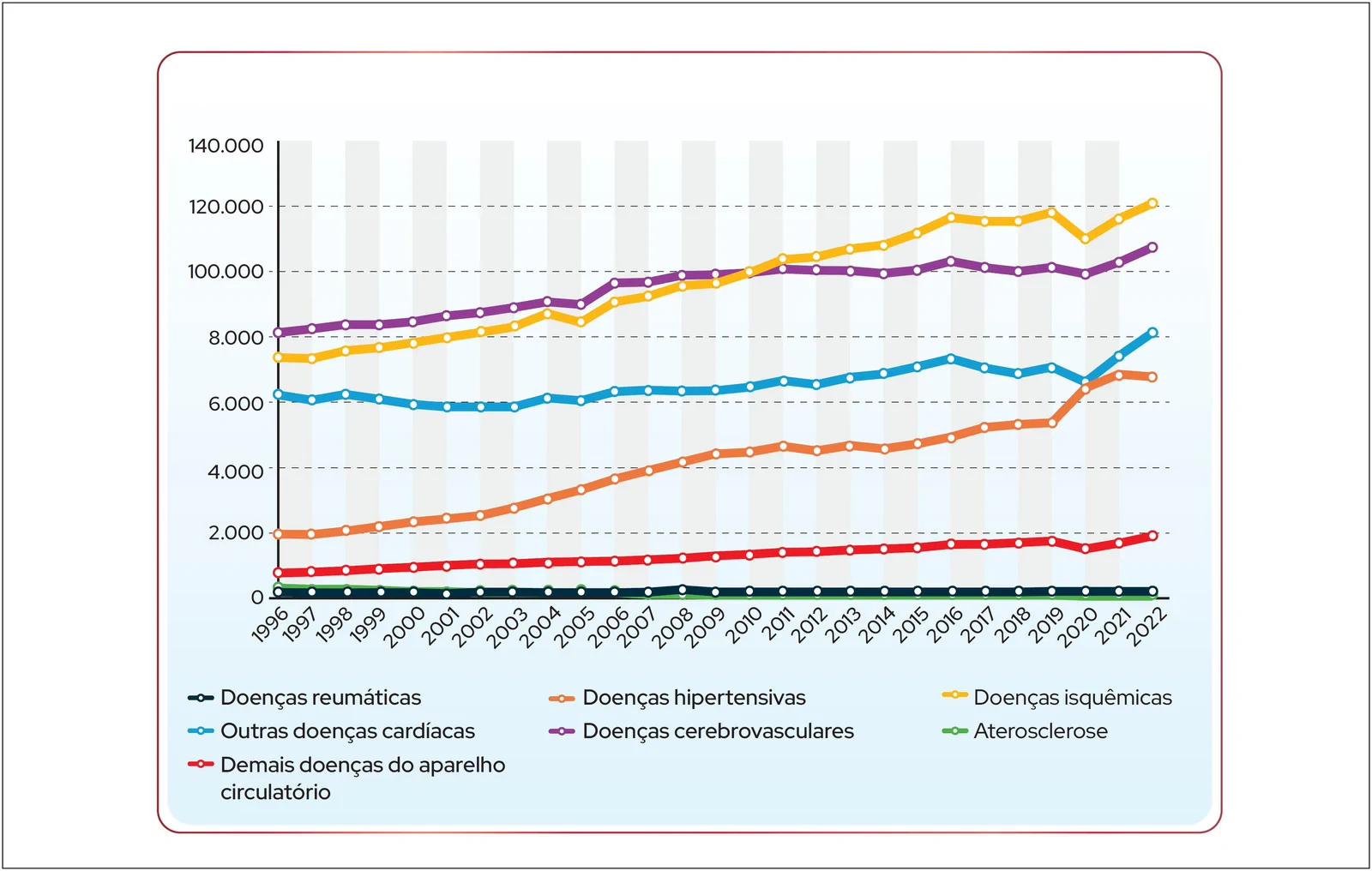
Número absoluto de mortes pelas doenças cardiovasculares entre 1996 e 2022 (DATASUS).^9^

### 2.3. Fatores de Risco para Hipertensão Arterial

#### 2.3.1. Genética

Embora existam mutações genéticas monogênicas raras associadas à HA, a herança genética associada à HA primária é tipicamente poligênica, envolvendo milhares de variantes genéticas de risco, as quais exercem, individualmente, efeitos de pequena magnitude sobre a PA. A probabilidade de ocorrência de HA primária aumenta com o número de alelos de risco presentes e é modulada por fatores ambientais.^10^

#### 2.3.2. Idade e Sexo

A prevalência de HA aumenta em proporção linear com o avançar da idade, tendo chegado a 65,1% em indivíduos acima de 65 anos em 2023.^2^ No geral, homens a partir do final da adolescência apresentam prevalência de HA significativamente maior do que as mulheres. Contudo, as mulheres tendem a apresentar um aumento mais acentuado da PA com a idade, fazendo com que apresentem prevalência de HA similar ou mesmo superior à dos homens após os 60 anos.^11^

#### 2.3.3. Etnia, Urbanização e Fatores Socioeconômicos

Esses três fatores estão intimamente conectados e, além de influenciarem diversos aspectos relevantes (estilo de vida, cosmovisão de mundo, dieta, adesão terapêutica, vulnerabilidade social, atividade física (AF), acesso ao sistema de saúde e nível educacional), são determinantes de risco para a HA.

Além desses, há o risco residual relacionado à cor da pele mesmo após ajustes para indicadores socioeconômicos, comportamentais e de saúde. No Brasil, a incidência de HA foi de 43,4/1.000 pessoas-ano, sendo mais baixa entre mulheres brancas (30,5/1.000 pessoas-ano). Considerando a taxa incidência em mulheres brancas como referência, ela foi maior em mulheres pardas (1,47; IC 95% 1,31–1,67), mulheres negras (1,85; IC 95% 1,50–2,30), homens brancos (1,76; IC 95% 1,49–2,08), homens pardos (1,89; IC 95% 1,59–2,25) e homens negros (2,25; IC 95% 1,65–3,08).^12^ Além disso, a contribuição da HA para eventos CV é habitualmente maior na população negra do que na população branca.^13,14^

Os grupos étnicos indígenas brasileiros estão tradicionalmente em vulnerabilidade social e muitos deles em rápido processo de urbanização. Uma metanálise incluindo pelo menos 33 etnias indígenas mostrou a prevalência mais alta de HA em indígenas que vivem em áreas urbanas na Região Sul (30%; IC 95% 10–50) e mais baixas nas áreas menos urbanizadas da Região Norte (1%; IC 95% 1–2) do Brasil.^15^ Quando comparados indígenas e não indígenas em um mesmo estudo, observa-se que os indígenas que vivem em áreas urbanas tendem a ter prevalência de HA similar, mas maior chance de apresentar PA não controlada do que os não indígenas.^16^

#### 2.3.4. Espiritualidade e Fatores Psicossociais

A SBC publicou um Posicionamento sobre HA e Espiritualidade em 2021, após extensa revisão da literatura, reconhecendo que a espiritualidade e a religiosidade podem agir por meio de mecanismos como redução do estresse, melhora do autocuidado e maior adesão ao tratamento, potencialmente influenciando no controle da PA.^17^

Fatores psicossociais, como estresse ocupacional, baixo *status* socioeconômico, isolamento social, discriminação racial, depressão e ansiedade têm sido identificados como potencializadores do desenvolvimento da HA e de suas complicações. Em metanálise de artigos publicados na literatura, o estresse psicossocial foi associado a um risco 2,4 (IC 95% 1,6–3,5) vezes maior de HA, e pacientes com HA tiveram 2,7 (IC 95% 2,3–3,1) vezes maior incidência de estresse psicossocial em comparação com normotensos.^18^

#### 2.3.5. Sobrepeso/Obesidade e Sedentarismo

A relação entre índice de massa corporal (IMC) e PAS ou PAD é contínua e direta.^19^ As principais consequências do sobrepeso ou obesidade incluem maior prevalência de HA e uma cascata de distúrbios metabólicos associados.^20^

O sedentarismo é um fator associado não apenas à HA, mas também à doença arterial coronariana (DAC), insuficiência cardíaca (IC), resistência à insulina, diabetes mellitus tipo 2 (DM2), dislipidemia, acidente vascular cerebral (AVC), demência e outras doenças crônicas.^21^

#### 2.3.6. Ingestão de Sódio e Potássio

A ingestão elevada de sódio é um fator de risco bem determinado para a elevação da PA e maior prevalência de HA na infância e na vida adulta.^22,23^ Além disso, reduções da PA podem ser obtidas ao se utilizar substitutos do sal tradicional, enriquecidos com potássio.^24^

#### 2.3.7. Álcool

Dados do VIGITEL de 2023 demonstram o uso abusivo crescente de álcool no Brasil (mulheres com quatro doses/dia e homens com cinco doses/dia em pelo menos 1 dos 30 dias anteriores) entre 2006 (15,7%) e 2023 (20,8%), mais às custas das mulheres (7,8% para 15,2%).^2^ Existe forte associação positiva, contínua e não linear entre consumo de álcool e PA.^25^ De fato, a ingestão de mais de 15 g/dia nas mulheres e 30 g/dia nos homens (07 e 14 doses semanais, respectivamente) têm demonstrado representar risco para o desenvolvimento de HA.^26^

#### 2.3.8. COVID-19

A COVID-19 não se apresentou de forma conclusiva como fator de risco para o desenvolvimento de HA. Um grande levantamento brasileiro entre janeiro de 2019 e dezembro de 2020 não mostrou efeitos significativos da pandemia no perfil da HA, para medidas de consultório e fora do consultório. Nesse estudo, pacientes tratados (n = 27.699), comparados aos não tratados (n = 24.227), tiveram melhora modesta e transitória no controle da PA de consultório, restrita aos primeiros meses após o surto pandêmico.^27^ No entanto, a HA parece ser um fator de pior prognóstico no paciente com COVID-19. No Brasil, houve aumento na taxa de mortalidade por DCV durante a pandemia, e a HA esteve entre os fatores de risco para complicações fatais nos que desenvolveram COVID-19.^28,29^

#### 2.3.9. Medicamentos, Drogas Ilícitas e Uso de Esteroides Anabolizantes

Algumas medicações têm potencial de elevar a PA ou dificultar seu controle, como: inibidores da monoaminoxidase, simpatomiméticos, antidepressivos tricíclicos, hormônios tireoidianos, contraceptivos orais, anti-inflamatórios não esteroidais, glicocorticoides, inibidores da calcineurina, carbexonolona, alcaçuz, eritropoietina e testosterona. Drogas ilícitas também podem elevar a PA, como cocaína, *Cannabis sativa*, anfetamina e 3,4 metilenodioximetanfetamina (MDMA).^1^

Há uma crescente utilização de testosterona por pessoas transgênero, com prevalência de HA em trans masculinos variando grandemente de 1,5 a 25,2%. A estimativa de prevalência de HA em trans femininos também tem variado acentuadamente, com valores entre 1,5 e 29,2%. No entanto, é ainda incerto se transgêneros estão sob maior risco de desenvolver HA.^30^ Por outro lado, o uso de esteroides anabolizantes para fins estéticos e de *performance* está associado à HA, bem como a outras DCV e renais.^31^

#### 2.3.10. Distúrbios do Sono

Risco aumentado para HA tem sido demonstrado com relação à privação, duração e qualidade do sono. Distúrbios do sono, como a apneia obstrutiva do sono, afetam mecanismos reguladores como ativação do sistema nervoso simpático, aumento da liberação de cortisol e inflamação crônica.^32-34^

#### 2.3.11. Tabagismo

Dados do VIGITEL 2023 mostram redução progressiva do tabagismo no Brasil entre 2006 (15,7%) e 2018 (9,3%), mantendo-se estável até 2023.^2^ A despeito de o tabagismo estar inquestionavelmente relacionado ao aumento da morbidade e mortalidade CV, seu papel direto na indução da HA ainda é controverso.^35,36^ Mais recentemente, o uso de cigarros eletrônicos, apesar de proibido no país,^37^ tem sido motivo de preocupação, principalmente entre adolescentes, já que substâncias presentes nesses dispositivos associam-se a DCV.^38^ Essas substâncias têm demonstrado efeitos agudos de elevação da PA, mas ainda com efeitos pouco conhecidos no longo prazo.^39^

#### 2.3.12. Fatores Ambientais

Os mecanismos que relacionam fatores ambientais com o risco aumentado para HA são complexos e envolvem epigenética, fatores sociais e composição de microbiota intestinal.^40-42^ A poluição sonora, por exemplo, parece relacionar-se ao aumento da PA mediado por elevação do estresse psicossocial, enquanto a poluição do ar acarreta alterações na PA por meio de poluentes físicos e químicos, gerando disfunção endotelial e aumento do estresse oxidativo. Além disso, a ausência de espaços verdes nas cidades já se mostrou relacionada à inatividade física e redução da interação social, as quais são determinantes de HA.^40^

O impacto do clima no risco de HA também é complexo, embora cada vez mais bem demonstrado. Em países temperados, aumentos da PAS são observados nas temperaturas de inverno (em que há aumento da norepinefrina) em comparação às de verão (em que há aumento do óxido nítrico e do nível de AF). Ademais, variações extremas de temperatura durante o verão ou inverno estão associadas ao aumento de eventos CV.^40^ No Brasil, regiões com temperaturas ambientais mais elevadas apresentam menor prevalência de HA, enquanto regiões com temperaturas mais baixas exibem maior prevalência de HA.^41^

Nos últimos anos, tem crescido o entendimento sobre o papel do microbioma intestinal na elevação da PA. No entanto, mais estudos ainda se fazem necessários para que se possa posicionar adequadamente a relevância clínica do microbioma intestinal na gênese da HA e de suas complicações.^42^

Na Figura 2.2, estão resumidos os principais fatores de risco para a HA.

**Figura 2.2:**
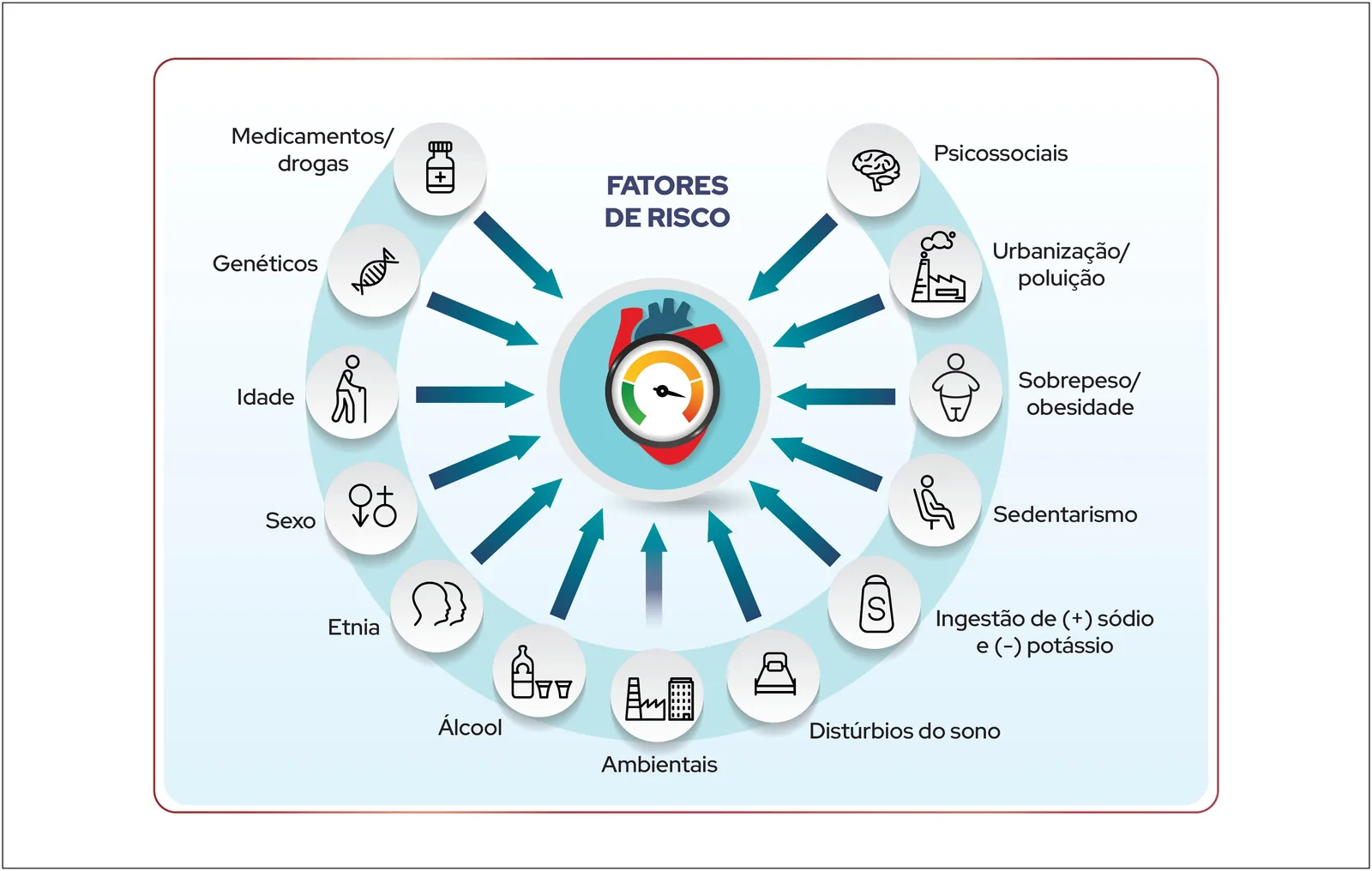
Principais fatores de risco para hipertensão arterial.

### 2.4. Prevenção Primária

A prevenção primária da HA deve ser feita por meio de uma abordagem centrada no paciente, a fim de promover um estilo de vida mais saudável (Figura 2.3).^5^ O Capítulo 6 desta diretriz revisa em detalhes as recomendações sobre medidas não medicamentosas (MNM), as quais cumprem papel importante tanto na prevenção quanto no tratamento da HA.

**Figura 2.3:**
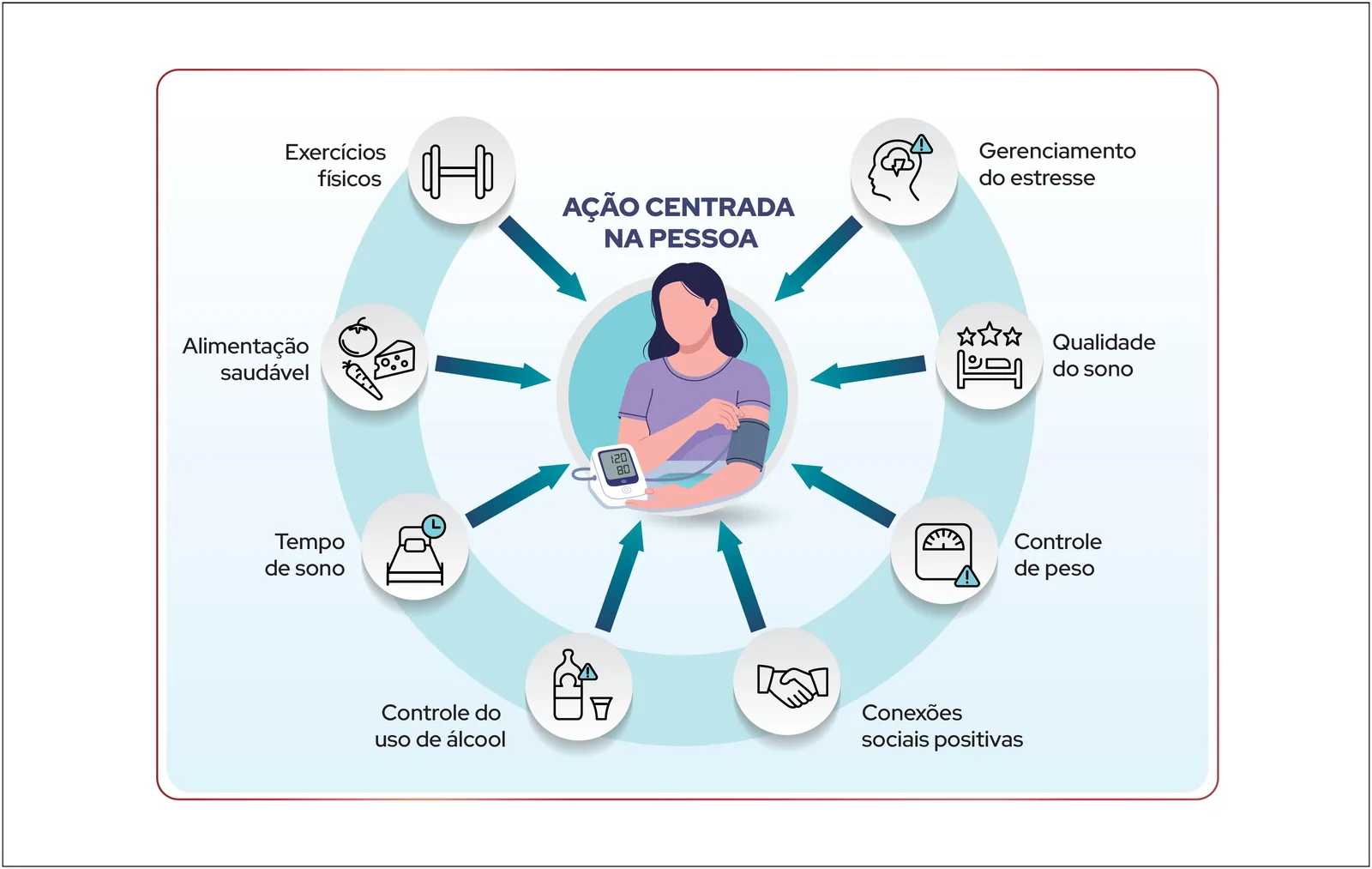
Medidas para prevenção da hipertensão arterial.

Em linhas gerais, devem fazer parte do plano de prevenção da HA: controle do peso corporal, alimentação saudável, AF regular, sono de qualidade e com duração adequada, gerenciamento do estresse, controle do consumo de álcool e o fortalecimento de conexões sociais positivas. Em relação à cessação do tabagismo, essa recomendação deve ser sempre feita pelos profissionais de saúde como medida de prevenção primária CV, independentemente do potencial benefício na redução da PA.^36,38^

A implementação dessas medidas deve prever engajamento e responsabilidade pessoal, incentivando o indivíduo a ser o protagonista de sua saúde a fim de promover mudanças sustentáveis no estilo de vida. Além disso, é central assegurar políticas públicas que promovam a cultura da prevenção e o autocuidado para HA.^43^

**Table:** 

Mensagens principais
Há elevada prevalência de HA na população adulta brasileira com baixas taxas de controle da PA.
A HA é o principal determinante de mortalidade CV no Brasil e no mundo.
A HA é multifatorial, sendo que seus fatores de risco incluem determinantes genéticos inatos, envelhecimento, obesidade, sedentarismo, fatores psicossociais de estresse, alimentação inadequada, consumo de drogas lícitas e ilícitas, distúrbios do sono, abuso de álcool e fatores ambientais complexos, com comportamento diferente conforme o sexo, diferenças étnicas e fatores socioeconômicos.
A prevenção da HA deve ser feita por meio de uma abordagem centrada no indivíduo, a fim de direcioná-lo a um estilo de vida mais saudável, que inclua controle do peso corporal, alimentação saudável, AF regular, sono de qualidade e com duração adequada, gerenciamento do estresse, controle do consumo de álcool e o fortalecimento de conexões sociais positivas, reforçando uma abordagem multiprofissional sustentável e estimulando políticas públicas de promoção à saúde.

AF: atividade física; CV: cardiovascular; HA: hipertensão arterial; PA: pressão arterial.

## 3. Medida da Pressão Arterial, Diagnóstico e Classificação

### 3.1. Introdução

A medida da PA é fundamental para a avaliação inicial de indivíduos com ou sem HA, devendo ser realizada em todas as consultas médicas e por todos os profissionais de saúde, utilizando técnicas adequadas e equipamentos validados e calibrados. A PA deve ser medida tanto no consultório quanto fora dele – por meio de medida ambulatorial da pressão arterial (MAPA) e/ou medida residencial da pressão arterial (MRPA) – para garantir um diagnóstico preciso e monitorar o controle da HA.^44-46^ A avaliação diagnóstica de HA inclui a confirmação da condição, a identificação de causas secundárias e a avaliação do risco CV, sendo fundamental investigar a possibilidade de lesão de órgãos-alvo (LOA) e doenças associadas. Esse processo deve envolver, preferencialmente, múltiplas medidas da PA em diferentes ocasiões, história médica completa, exame físico detalhado e investigação clínica e laboratorial.

### 3.2. Medida da Pressão Arterial no Consultório

A PA deve ser medida em todas as avaliações por profissionais de saúde capacitados. Entretanto, o diagnóstico de HA e seus fenótipos, assim como a conduta clínica, são de responsabilidade exclusiva dos médicos.^46^

#### 3.2.1. Técnicas de Medida da Pressão Arterial

A medida da PA deve ser realizada utilizando técnica correta e equipamento adequado.^46-48^ A medida da PA com esfigmomanômetros automáticos ou semiautomáticos usando técnica oscilométrica oferece vantagens em relação à técnica auscultatória com esfigmomanômetros aneroides, com redução dos erros provocados pelo observador e maior facilidade na execução do procedimento.^44-46^ Outra vantagem é possibilitar a realização da medida desacompanhada da PA no consultório, na qual o indivíduo realiza a medida em uma sala reservada, sem a presença do profissional de saúde, o que melhora a reprodutibilidade das medidas e reduz o efeito do avental branco.^46,49^ No entanto, é essencial que os equipamentos sejam validados de acordo com protocolos padronizados e a calibração seja avaliada pelo menos anualmente.^46^

A PA deve ser medida em ambos os braços inicialmente, utilizando o braço com a PA mais elevada para medidas subsequentes. Diferenças superiores a 15 mmHg na PAS entre os braços indicam um risco CV aumentado e podem sinalizar a presença de doença arterial obstrutiva.^50^ Além disso, essa diferença também está associada à remodelação adversa do ventrículo esquerdo.^51^

#### 3.2.2. Populações Especiais

Em idosos e nos indivíduos com DM, disautonomia e em uso de anti-hipertensivos, a PA deve ser medida também em posição supina e 1 e 3 minutos após a mudança para a posição ortostática, com o braço apoiado pelo examinador na altura do coração. Isso é importante para identificar a hipotensão ortostática, que é definida como uma redução na PAS ≥ 20 mmHg e/ou na PAD ≥ 10 mmHg.^45,46,52,53^

Para todos os pacientes, é indispensável utilizar manguitos adequados ao tamanho e à forma do braço para evitar sub ou superestimação da PA. Quando a medida no braço for difícil, em particular nos obesos, pode-se considerar a medida no punho, utilizando o pulso radial por meio de aparelhos validados.^46^ No caso de impossibilidade de medida de PA nos membros superiores, tem-se sugerido a medida de PA nos membros inferiores, especialmente nas panturrilhas, utilizando-se aparelhos oscilométricos.^54^

Medidas inadequadas da PA podem resultar em diagnósticos imprecisos e tratamentos inadequados. Assim, é fundamental seguir protocolos rigorosos para assegurar a precisão e eficácia no manejo da HA. A Figura 3.1 e o Quadro 3.1 ilustram as etapas da medida da PA. Para informação mais aprofundada sobre as técnicas recomendadas, consulte a "Diretriz de Medidas da Pressão Arterial Dentro e Fora do Consultório".^46^ A adesão a essas diretrizes garante que as medidas da PA sejam realizadas com a máxima precisão, minimizando erros e melhorando o tratamento dos pacientes com HA.

**Figura 3.1:**
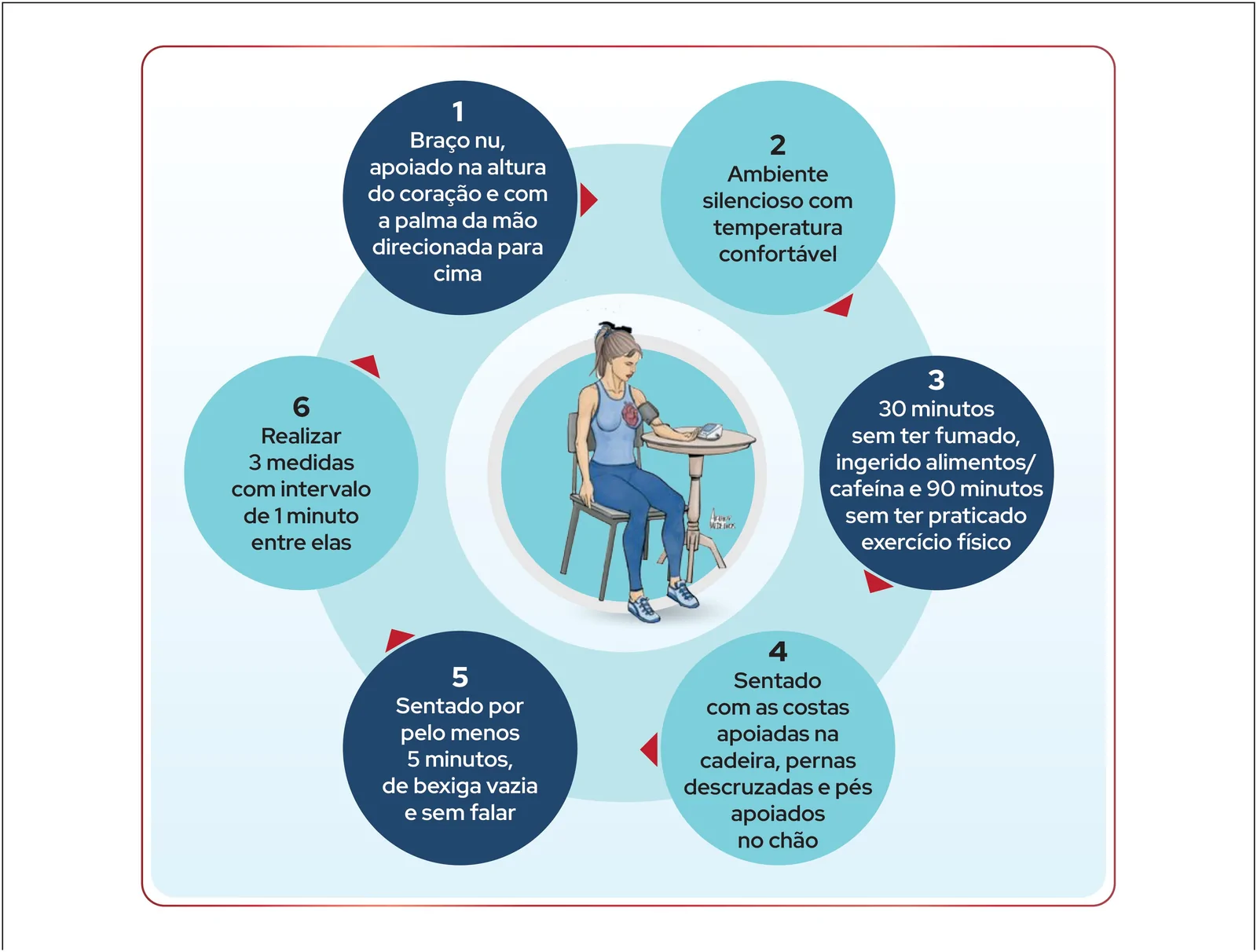
Etapas para a medida da pressão arterial em consultório.

**Quadro 3.1:** Procedimento de medida da pressão arterial em consultório^46^

Etapas de medida da pressão arterial
Colocar o manguito, sem folgas, 2 a 3 cm acima da fossa cubital, centralizar o meio da bolsa inflável sobre a artéria braquial.
Estimar o nível da PAS.*
Colocar a campânula ou o diafragma do estetoscópio, sem compressão excessiva, na fossa cubital.*
Inflar rapidamente até ultrapassar 20 a 30 mmHg o nível estimado da PAS.*
Realizar a deflação lentamente (cerca de 2 mmHg/s).*
Determinar a PAS na ausculta do primeiro som.*
Determinar a PAD desaparecimento dos sons.*
Realizar três medidas, com intervalo de 1 minuto e utilizar a média das duas últimas medidas. Se houver diferença > 10 mmHg, realizar medidas adicionais.

PAS: pressão arterial sistólica; PAD: pressão arterial diaslótica.

* Aplicados à técnica auscultatória.

#### 3.2.3. Classificação

A partir de medidas obtidas no consultório, são considerados pacientes com HA os indivíduos com PAS ≥ 140 mmHg e/ou PAD ≥ 90 mmHg. A classificação da PA é detalhada no Quadro 3.2.^46^ A nova classificação da PA proposta por esta diretriz apresenta algumas alterações importantes em relação às DBHA de 2020. As principais mudanças são:

**Quadro 3.2:** Classificação da pressão arterial de acordo com a medida no consultório a partir de 18 anos de idade

Classificação da PA	PAS (mmHg)		PAD (mmHg)
**PA normal**	< 120	e	< 80
**Pré-hipertensão**	120-139	e/ou	80-89
**HA Estágio 1**	140-159	e/ou	90-99
**HA Estágio 2**	160-179	e/ou	100-109
**HA Estágio 3**	≥ 180	e/ou	110

DBHA: Diretriz Brasileira de Hipertensão Arterial; HA: hipertensão arterial; PA: pressão arterial; PAD: pressão arterial diastólica; PAS: pressão arterial sistólica. ^*^A classificação é definida de acordo com a PA no consultório e pelo nível mais elevado de PA, sistólica ou diastólica. ^**^A HA sistólica isolada, caracterizada pela PAS ≥ 140 mmHg e PAD < 90 mmHg, é classificada em Estágio 1, 2 ou 3, de acordo com os valores da PAS nos intervalos indicados. ^***^A HA diastólica isolada, caracterizada pela PAS < 140 mmHg e PAD ≥ 90 mmHg, é classificada em Estágio 1, 2 ou 3, de acordo com os valores da PAD nos intervalos indicados.

PA ótima: a classificação "PA ótima" foi removida. Esse intervalo de PA, antes considerado ideal, agora está incluído dentro do novo termo "PA normal" para valores de PAS abaixo de 120 mmHg e PAD abaixo de 80 mmHg;

Pré-hipertensão: a nova categoria de "Pré-hipertensão" agora abrange os valores anteriormente classificados como "PA normal" e "Pré-hipertensão" nas DBHA 2020. Isso significa que valores de PAS entre 120 e 129 mmHg e/ou PAD entre 80 e 84 mmHg, antes considerados normais, passam a ser classificados como "Pré-hipertensão". Assim, essa faixa inclui PAS entre 120 e 139 mmHg e/ou PAD entre 80 e 89 mmHg. Essas alterações buscam identificar precocemente indivíduos em risco e incentivar intervenções mais proativas para prevenir a progressão para HA.

Para validar o diagnóstico de HA, é necessário realizar medidas repetidas, em duas ou mais visitas médicas, com intervalos de dias ou semanas. Alternativamente, pode-se utilizar a MAPA ou a MRPA para uma avaliação mais precisa. No entanto, em pacientes com LOA ou DCV estabelecida, a presença desses achados é suficiente para confirmar o diagnóstico de HA, dispensando a necessidade de medidas de PA fora do consultório. A classificação de HA baseia-se no valor mais elevado de PA, seja sistólica ou diastólica, aferida no consultório.^44-46^

Indivíduos com PAS ≥ 140 mmHg e/ou PAD < 90 mmHg são definidos como pacientes com HA sistólica isolada, enquanto aqueles com PAS < 140 mmHg e PAD ≥ 90 mmHg são classificados como com HA diastólica isolada.^46^

### 3.3. Medida da Pressão Arterial fora do Consultório

A PA fora do consultório pode ser obtida por meio da MAPA e/ou da MRPA. Essas modalidades de medida da PA são fundamentais para confirmar o diagnóstico de HA e monitorar o controle da PA, especialmente em pacientes com suspeita de hipertensão do avental branco (HAB) ou hipertensão mascarada (HM). O Quadro 3.3 sumariza as vantagens e desvantagens desses métodos.

**Quadro 3.3:** Principais vantagens e desvantagens da medida da pressão arterial fora do consultório^46^

Vantagens	MAPA	MRPA
Medida da PA fora do consultório e sem a presença do médico.	X	X
Correlação com prognóstico melhor do que a PA no consultório.	X	X
Pode reduzir gastos com saúde.	X	X
É considerada o padrão-ouro na avaliação da PA.	X	
Avalia o controle da PA nas 24 horas; durante o sono e atividades diárias.	X	
Avalia a elevação rápida da PA matinal.	X	
Monitora a eficácia do tratamento a longo prazo e melhora a adesão.		X
Favorece o controle da PA e adesão ao tratamento.		X
Custo menor do que o da MAPA.		X
Desvantagens		
Disponibilidade por vezes limitada.	X	X
Relutância de alguns pacientes em usar e repetir o exame.	X	X
Pode ser desconfortável, principalmente durante o sono.	X	
Frequentemente, a PA durante o sono não é calculada de acordo com os horários de sono do indivíduo.	X	
Medida da PA somente em repouso na vigília.		X
Requer orientação e treinamento; potencial para erro de medida: medida em momentos inadequados, número excessivo de medidas, indução de ansiedade, mudança da medicação pelo paciente, paciente relata valores mais baixos.		X

MAPA: monitorização ambulatorial da pressão arterial de 24 horas; MRPA: monitorização residencial da pressão arterial; PA: pressão arterial.

#### 3.3.1. Indicações da Medida Ambulatorial da Pressão Arterial e Medida Residencial da Pressão Arterial

Tanto a MAPA quanto a MRPA são indicadas em várias situações clínicas importantes, que incluem suspeita de HAB, HM, avaliação da eficácia do tratamento anti-hipertensivo e o diagnóstico e acompanhamento de HA em pacientes com variabilidade significativa da PA. A MRPA pode ser integrada à telemedicina, melhorando a adesão ao tratamento e o controle da PA, especialmente quando combinada com orientação médica. A MAPA é útil na avaliação do comportamento da PA durante o sono e, segundo um estudo recente, pode ser combinada com a polissonografia sem afetar o tempo total de sono, sua eficiência ou o número de despertares.^55^ Os valores de anormalidade da PA estão resumidos no Quadro 3.4, e as principais indicações dos métodos encontram-se no Quadro 3.5.^46,56-60^

**Quadro 3.4:** Definição de hipertensão arterial de acordo com a medida da pressão arterial de consultório, monitorização ambulatorial da pressão arterial e monitorização residencial da pressão arterial

Categoria		PAS (mmHg)		PAD (mmHg)
PA no consultório		≥ 140	e/ou	≥ 90
MAPA 24 horas		≥ 130	e/ou	≥ 80
	Vigília	≥ 135	e/ou	≥ 85
	Sono	≥ 120	e/ou	≥ 70
MRPA		≥ 130	e/ou	≥ 80

HA: hipertensão arterial; MAPA: monitorização ambulatorial da pressão arterial; MRPA: monitorização residencial da pressão arterial; PA: pressão arterial; PAD: pressão arterial diastólica; PAS: pressão arterial sistólica.

**Quadro 3.5:** Indicações da monitorização ambulatorial da pressão arterial e da monitorização residencial da pressão arterial^46^

Indicações	MAPA	MRPA
Suspeita de HAB – efeito avental branco.	X	X
HA Estágio 1 (140-159 e/ou 90-99 mmHg) no consultório.	X	X
PA ≥ 140/90 mmHg no consultório sem LOA e com baixo RCV.	X	X
Hipertensão sistólica isolada ou diastólica isolada no consultório.	X	X
Suspeita de HM – efeito de mascaramento.	X	X
PA dentro da faixa de pré-hipertensão (130-139 e/ou 85-89 mmHg).	X	X
PA < 140/90 mmHg no consultório com LOA e alto RCV.	X	X
Suspeita de efeito do avental branco.	X	X
PA elevada no consultório ou suspeita de pré-eclâmpsia em gestantes.	X	X
Identificar HA não controlada, HAR e redução excessiva da PA.	X	X
Ajustar a medicação anti-hipertensiva.	X	X
Assegurar o controle adequado da PA.	X	X
Avaliar o controle da PA nas 24 horas, durante o sono e atividades diárias.	X	
Identificar hipotensão ortostática, pós-prandial e na sesta.	X	
Avaliar as variações da PA na disautonomia.	X	
Avaliação de sintomas, principalmente de hipotensão.	X	
Monitorar a eficácia do tratamento no longo prazo e melhorar a adesão.		X

HA: hipertensão arterial; HAB: hipertensão do avental branco; HAR: hipertensão arterial resistente; HM: Hipertensão mascarada; LOA: lesões de órgãos-alvo; MAPA: monitoração ambulatorial da pressão arterial; MRPA: monitoração residencial da pressão arterial; PA: pressão arterial; RCV: risco cardiovascular.

### 3.4. Efeito do Avental Branco e Efeito de Mascaramento

O efeito do avental branco e o efeito de mascaramento referem-se às diferenças na PA entre as medidas obtidas no consultório e fora dele.

O efeito do avental branco é considerado significativo quando a PAS no consultório é ≥ 20 mmHg (MAPA – média de 24 horas) ou ≥ 15 mmHg (MRPA), ou a PAD é ≥ 15 mmHg (MAPA – média de 24 horas) ou ≥ 9 mmHg (MRPA) em comparação com as medidas fora do consultório.^61,62^ Por outro lado, o efeito de mascaramento é considerado significativo quando a PAS no consultório é ≤ 2 mmHg (MAPA – média de 24 horas) ou ≤ −1 mmHg (MRPA), ou a PAD é ≤ 2 mmHg (MAPA – média de 24 horas) ou ≤ −1 mmHg (MRPA) em comparação com as medidas fora do consultório.^61,62^

Essas variações não alteram o diagnóstico de HA, mas ajudam a identificar quais pacientes devem realizar medidas de PA fora do consultório com mais frequência, contribuindo para um manejo terapêutico mais eficaz.

### 3.5. Hipertensão do Avental Branco e Hipertensão Mascarada

A HAB ocorre quando a PA é elevada no consultório (≥ 140 mmHg e/ou 90 mmHg), mas normal fora dele (< 130/80 mmHg na MRPA ou na MAPA de 24 horas), enquanto a HM ocorre quando a PA é normal no consultório, mas elevada fora dele.

A prevalência de HAB varia entre 7 e 15% e é mais comum em pacientes com HA estágio 1 e naqueles com HA sistólica ou diastólica isolada.^46,56,57^ A prevalência de HM varia entre 8 e 22%, sendo mais comum em indivíduos com fatores de risco adicionais, como DM e obesidade, podendo chegar no indivíduo pré-hipertenso a 62% (Figura 3.2).^46^ Em indivíduos com DM, contudo, a presença de HM parece ser mais bem identificada pela MAPA do que pela MRPA^46,63^ Tanto a HAB quanto a HM estão associadas a um maior risco de desenvolver DM, LOA e eventos CV a longo prazo. Por outro lado, a presença de PA normal no consultório e fora dele define o indivíduo com normotensão verdadeira, enquanto o indivíduo com PA elevada no consultório e fora dele é definido com HA sustentada.

**Figura 3.2:**
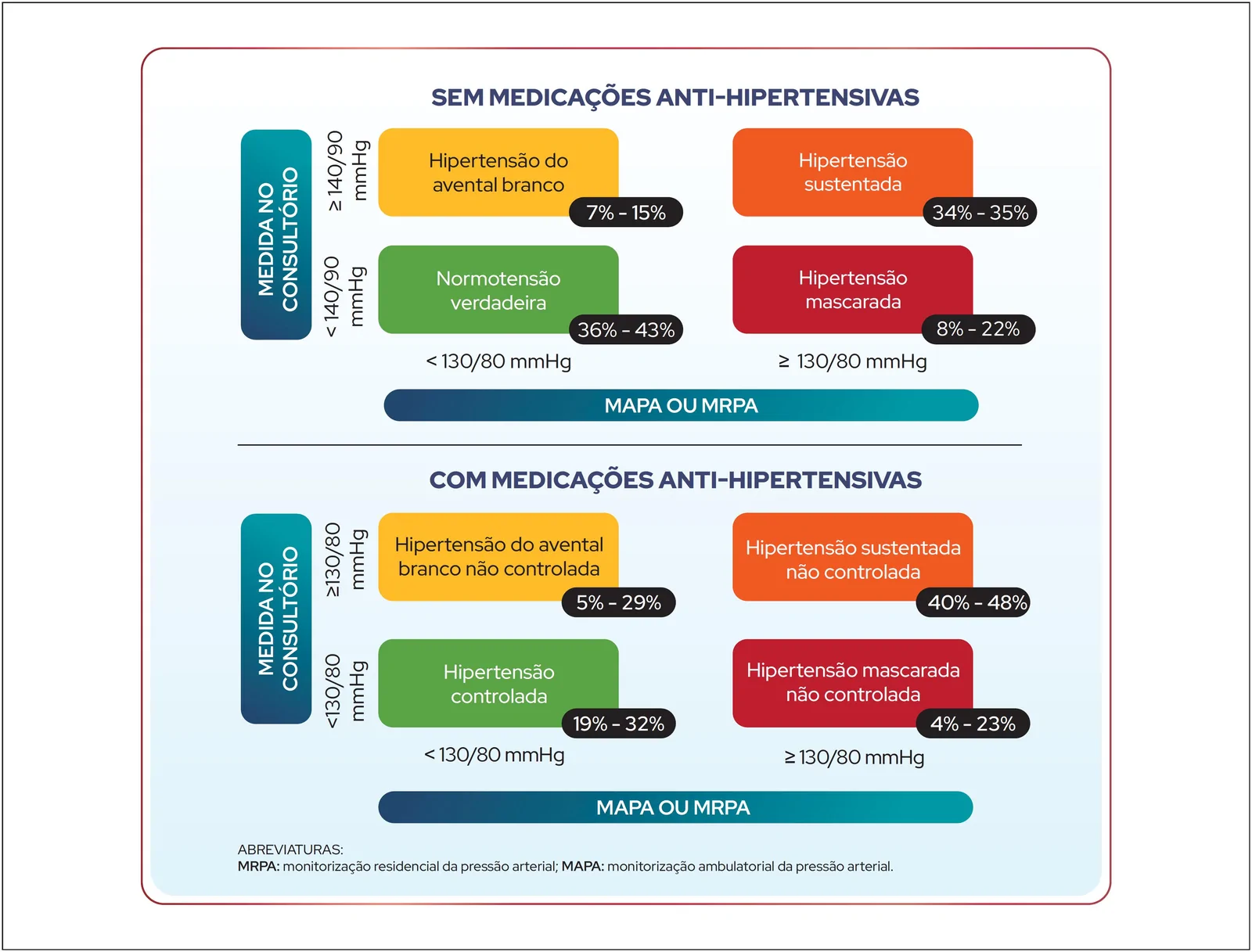
Prevalências dos fenótipos de pressão arterial entre pacientes tratados ou não com medicações anti-hipertensivas.

Para uma leitura mais completa, consulte a "Diretriz de Medidas da Pressão Arterial Dentro e Fora do Consultório".^46^

### 3.6. Hipertensão Mascarada Não Controlada e Hipertensão do Avental Branco Não Controlada

Em pacientes tratados com medicações anti-hipertensivas, a PA também pode apresentar concordância ou discrepância entre medidas no consultório e fora dele. Os termos correspondentes para normotensão verdadeira, HAB, HM e HA sustentada são HA controlada, HAB não controlada (HABNC), HM não controlada (HMNC) e HA sustentada não controlada. Nos pacientes tratados com medicações anti-hipertensivas, esta diretriz considera elevados os valores de PA no consultório ou fora do consultório (MRPA ou MAPA de 24 horas) quando ≥ 130 mmHg e/ou 80 mmHg. As prevalências desses fenótipos podem ser observadas na Figura 3.2.^64,65^ Para uma leitura mais completa, consulte a "Diretriz de Medidas da Pressão Arterial Dentro e Fora do Consultório".^46^

### 3.7. Recomendações para Diagnóstico e Seguimento

A HA é frequentemente assintomática e, por isso, sua presença deve ser avaliada em todas as consultas médicas e em programas de triagem populacional. As medidas da PA devem ser realizadas regularmente, com a frequência variando conforme a classificação da PA. A Figura 3.3 resume as orientações para diagnóstico e seguimento da HA, levando-se em consideração as medidas de PA realizadas tanto dentro quanto fora do consultório.^46^

**Figura 3.3:**
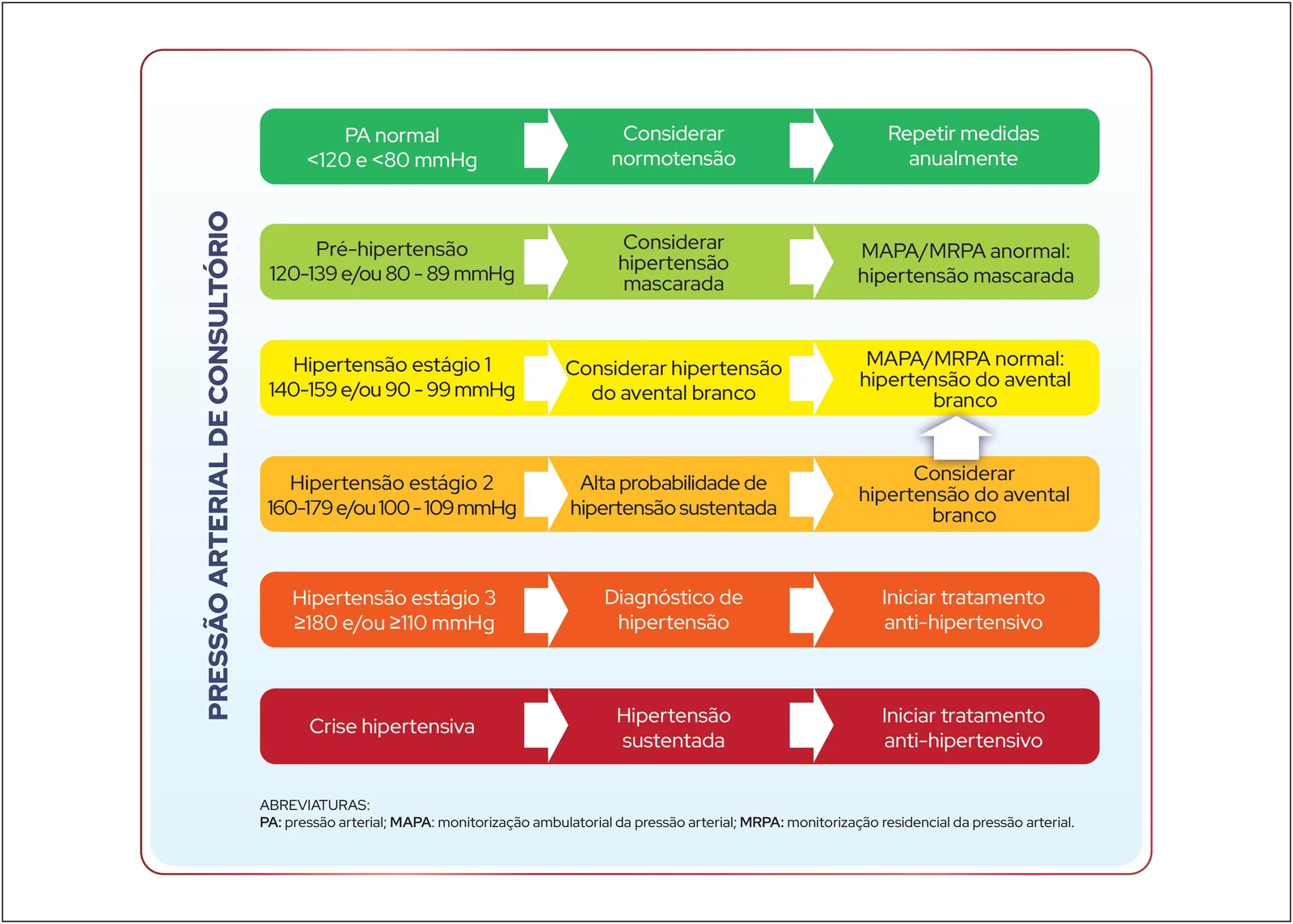
Triagem e diagnóstico de hipertensão arterial.

Devido à alta variabilidade da PA, o diagnóstico de HA não deve se basear em uma única medida no consultório, a menos que a PA esteja substancialmente elevada (HA Estágio 3) ou haja diagnóstico estabelecido de LOA ou DCV. Recomenda-se confirmar a elevação da PA com medidas repetidas em visitas subsequentes no consultório. Pacientes em Estágio 2 ou 3 requerem mais visitas, com intervalos curtos entre elas, enquanto aqueles em Estágio 1 podem ter intervalos mais longos, se houver ausência de LOA e risco CV baixo.^46^

Por outro lado, desde que viável logística e economicamente, a MAPA e MRPA devem ser realizadas para confirmar o diagnóstico de HA e monitorar o tratamento, visto que essas medidas são superiores à medida de PA no consultório para a predição de risco CV.^46,66,67^ Essa abordagem é também essencial para detectar HAB e HM, fornecendo informações clínicas adicionais.^46^

#### 3.7.1. Automedida da Pressão Arterial

A automedida da pressão arterial (AMPA) é a medida da PA realizada pelo próprio paciente ou familiar em seu domicílio, a qual não obedece a nenhum protocolo preestabelecido e é realizada com equipamento automático do próprio paciente.^46^ Ainda não há evidências conclusivas que respaldem a determinação de valores de normalidade para esse método, nem a adoção de protocolos específicos (número de medidas, assim como horários e dias de monitoração) para sua avaliação.^46^ Portanto, esta diretriz recomenda que a AMPA seja utilizada apenas como meio de triagem, para que métodos confirmatórios (MAPA ou MRPA) sejam solicitados.

#### 3.7.2. Teste Ergométrico

O teste ergométrico não é recomendado para a avaliação diagnóstica de HA devido à falta de padronização e consenso sobre a resposta normal da PA durante o exercício.^68^

### 3.8. Pressão Arterial Central, Velocidade de Onda de Pulso e *Augmentation Index*

A PA central não invasiva pode ser medida por meio de algoritmos específicos, a partir de medidas de PA obtidas no braço.^69^ Estudos indicam que a PA central pode ser um melhor preditor de eventos CV do que a PA braquial, embora mais evidências sejam necessárias para confirmar seu valor prognóstico incremental.^46^

A HA espúria, presente em jovens, principalmente homens atletas, é a principal indicação para a medida da PA central.^46,70^ Parâmetros associados, como a velocidade de onda de pulso (VOP) e o *augmentation index*, possuem valores prognósticos bem estabelecidos e podem ser úteis para melhor estratificação de risco CV do paciente. Para uma leitura mais completa sobre esse tema, consulte a "Diretriz de Medidas da Pressão Arterial Dentro e Fora do Consultório".^46^

### 3.9. Novas Tecnologias para Medidas da Pressão Arterial

Embora o método tradicional com manguito seja amplamente utilizado, ele apresenta limitações, como medições intermitentes e estáticas, além de erros devido ao tamanho ou posicionamento incorreto do manguito. Para resolver isso, surgiram novas tecnologias que permitem medir a PA de forma contínua e confortável, sem o uso de manguito. Essas tecnologias têm vantagens como a possibilidade de medições sem interferir no sono e nas atividades diárias, além de facilitar o automonitoramento e a adesão ao tratamento.^71^

Esses novos dispositivos funcionam analisando a onda de pulso, o tempo de chegada do pulso e o tempo de trânsito do pulso. No entanto, apesar de serem promissores, ainda existem dúvidas sobre a precisão desses novos métodos. Desafios relacionados à calibração, ao uso em diferentes populações e à falta de transparência sobre os algoritmos utilizados levantam preocupações.^72,73^ Por isso, recomenda-se cautela no uso clínico até que mais evidências sobre sua precisão e desempenho estejam disponíveis.

**Table:** 

Recomendação para medida da pressão arterial, diagnóstico e classificação	Forca da recomendação	Certeza da evidência
Recomenda-se classificar a pré-hipertensão abrangendo valores de PAS entre 120-139 mmHg ou PAD entre 80-89 mmHg no consultório com o objetivo de identificar precocemente indivíduos em risco e incentivar intervenções mais proativas e não medicamentosas para prevenir a progressão para HA.	FORTE	MODERADA
Recomenda-se que o diagnóstico de HA seja feito quando a PA no consultório for ≥ 140 e/ou 90 mmHg em duas ocasiões diferentes e classificada em Estágio 1, 2 e 3 de acordo com o maior valor de PAS ou PAD.	FORTE	MODERADA
Recomenda-se realizar a medida da PA utilizando técnica e equipamento adequados.	FORTE	MODERADA
Recomenda-se utilizar equipamento automático de braço com o objetivo de reduzir erros e facilitar a obtenção da medida da PA.	FORTE	MODERADA
Recomenda-se a investigação de hipotensão ortostática em grupos de risco (pacientes idosos, com diabetes, disautonomia e em uso de anti-hipertensivos).	FORTE	ALTA
Recomenda-se usar MAPA ou MRPA para confirmar diagnóstico de HA e monitorar o tratamento.	FORTE	ALTA
Recomenda-se, como principal indicação para avaliação de PA central, a investigação de HA sistólica espúria no indivíduo jovem.	FRACA	BAIXA

HA: hipertensão arterial; MAPA: medida ambulatorial da pressão arterial; MRPA: medida residencial da pressão arterial; PA: pressão arterial; PAD: pressão arterial diastólica; PAS: pressão arterial sistólica.

## 4. Avaliação Clínica e Complementar e Estratificação de Risco Cardiovascular

A avaliação clínica e complementar dos indivíduos com HA tem como objetivo não apenas definir a presença de HA, de seus determinantes e de possíveis causas secundárias, como também identificar a presença de fatores de risco CV associados e LOA.^45,74-76^

A estratificação de risco CV é um processo sistemático que visa categorizar os pacientes com base em seu risco individual de desenvolver desfechos CV e renais relevantes. Esse processo envolve a integração de múltiplos fatores de risco CV e marcadores de LOA, por meio de equações ou algoritmos que estimam o risco baseado em modelos de regressão com múltiplas variáveis e desenvolvidos a partir de estudos populacionais.^74^

### 4.1. Estratificação de Risco Adicional (Condições Associadas)

A estratificação de risco adicional em pacientes com HA envolve a identificação e o manejo de condições associadas que, isoladamente, podem aumentar o risco CV, independentemente dos níveis pressóricos. Entre essas condições, destacam-se os fatores de risco coexistentes, como DM, tabagismo, dislipidemia e obesidade, bem como as LOA, como hipertrofia ventricular esquerda (HVE), retinopatia, rigidez arterial, doença arterial obstrutiva periférica e doença renal crônica (DRC).^45,74^ Pacientes com múltiplos fatores de risco e/ou LOA apresentam um risco significativamente maior de desenvolver DCV graves, como infarto agudo do miocárdio (IAM), AVC, IC e mortalidade.^45,74^ A correlação linear entre idade e risco CV, com aumento acentuado em homens a partir dos 55 anos e em mulheres após os 65 anos,^76^ reforça a importância de intervenções precoces em populações de maior risco.

Entre os fatores de risco metabólicos, a dislipidemia, caracterizada por níveis elevados de colesterol de lipoproteína de baixa densidade (*low-density lipoprotein cholesterol* – LDLc) e triglicérides, bem como pela redução do colesterol de lipoproteína de alta densidade (*high-density lipoprotein cholesterol* – HDLc), é um dos fatores de risco que contribui significativamente para a progressão da aterosclerose.^77^ Da mesma forma, o DM, diagnosticado por meio de critérios estabelecidos como glicemia em jejum ≥ 126 mg/dL e hemoglobina glicada ≥ 6,5%, está fortemente associado ao aumento da mortalidade CV em pacientes com HA, destacando a importância do controle glicêmico rigoroso nessa população. Além desses, a obesidade, medida por meio do IMC e da circunferência abdominal, é um fator de risco bem estabelecido na HA, contribuindo para o desenvolvimento de resistência insulínica e disfunção endotelial.^78^

### 4.2. Avaliação Clínica e Complementar

A avaliação clínica e complementar do paciente com HA deve ser feita seguindo o método tradicional, constituído por anamnese, exame físico e exames complementares.

#### 4.2.1. Anamnese

Apesar de a HA ser de natureza assintomática, alterações decorrentes de LOA e complicações CV, renais e neurológicas podem gerar inúmeros sintomas. É fundamental a investigação dos determinantes de HA, descritos no Capítulo 2, os quais incluem hábitos alimentares, ganho ponderal, consumo de sal, ingestão de álcool, padrão do sono, nível de AF, assim como fatores socioeconômicos, biopsicossociais, culturais e ambientais. A história familiar também deve ser coletada para corroborar o diagnóstico de HA primária.^79^ O detalhamento do tempo de doença, do uso prévio ou atual de medicamentos e suas respectivas doses também é essencial para a avaliação e manejo do paciente com HA. Além disso, a busca por causas secundárias ou fatores contribuintes, como medicamentos ou substâncias que possam interferir na PA, deve ser sempre incluída na história clínica.

O Quadro 4.1 contempla um resumo dos objetivos a serem alcançados ao término da anamnese.

**Quadro 4.1:** Principais objetivos da anamnese

Pesquisar sintomas que possam estar relacionados à HA.
Questionar sobre história familiar de HA.
Explorar hábitos alimentares (ingestão de sal e tipo de dieta) e vícios como história de tabagismo, alcoolismo e uso de drogas ilícitas (tempo de uso e quantidade).
Investigar adesão a MNM, terapia anti-hipertensiva e demais medicamentos em uso, especialmente aqueles que podem interferir no controle da PA.
Questionar sobre história gestacional, em especial antecedentes de pré-eclâmpsia ou eclâmpsia, em mulheres.
Identificar fatores de risco CV e renal associados e aplicar escore de risco.
Pesquisar LOA.
Investigar a presença de outras doenças associadas.
Questionar sobre sedentarismo e nível de estresse.
Indagar sobre histórico do sono, ronco, apneia do sono.
Explorar história de disfunção erétil.
Rastrear indícios de HA secundária.

CV: cardiovascular; HA: hipertensão arterial; LOA: lesões em órgãos-alvo; MNM: medidas não medicamentosas; PA: pressão arterial.

#### 4.2.2. Exame Físico

O exame físico pode ajudar a avaliar a gravidade e a progressão da HA, auxiliar na detecção de formas secundárias de HA e identificar sinais de LOA e de fatores de risco associados. Embora um dos principais objetivos do exame físico seja a medida correta da PA, conforme descrito detalhadamente no Capítulo 3, a avaliação de todos os sistemas é importante. Deve haver particular ênfase no exame físico do sistema CV.^80^ O índice tornozelo-braquial (ITB), calculado pela razão da PAS do braço pela PAS do tornozelo, também faz parte do exame físico e melhora a acurácia da predição de risco CV.^81^ A avaliação antropométrica, a qual inclui a obtenção de peso, altura e medida da circunferência abdominal, permite calcular o IMC, diagnosticar a obesidade e estimar a presença de obesidade abdominal.^82^

Os principais pontos da avaliação do exame físico estão apresentados na Figura 4.1 e no Quadro 4.2.

**Figura 4.1:**
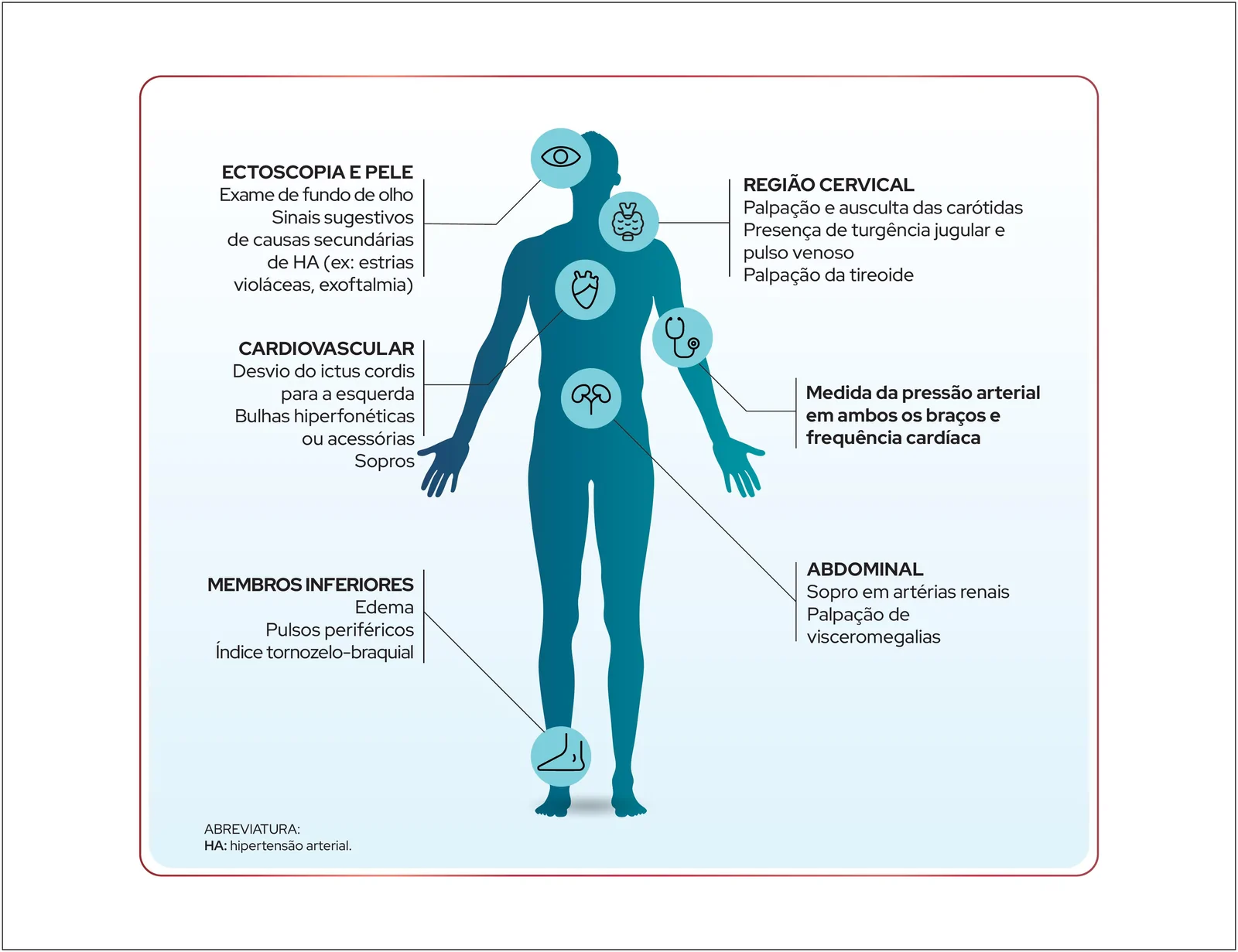
Principais pontos de avaliação do exame físico do paciente com hipertensão arterial.

**Quadro 4.2:** Parâmetros antropométricos e do índice tornozelo-braquial. CC: circunferência cintura; IMC: índice de massa corporal; ITB: índice tornozelo-braquial. Observação: a CC deve ser calculada no ponto médio entre a última costela e a crista ilíaca lateral.^83,84^

IMC (kg/m^2^)	Circunferência da cintura (cm)	ITB
Normal: < 24,9 Sobrepeso: 25-29,9 Obesidade: ≥ 30	CC normal: Mulher:< 88 cm Homem: < 102 cm	Normal: > 0,90 Obstrução leve: 0,71-0,90 Obstrução moderada: 0,41-0,70 Obstrução grave: ≤ 0,40

#### 4.2.3. Exames Complementares

O Quadro 4.3 elenca os exames complementares de rotina que devem ser solicitados para o paciente com HA na primeira consulta e no mínimo anualmente, quando estiverem dentro dos parâmetros normais. Em caso de alterações nesses exames, o controle deve ser feito na periodicidade requerida para se observar o efeito do manejo adotado.

**Quadro 4.3:** Exames complementares de rotina

Análise de urina
Potássio plasmático
Creatinina plasmática
Estimativa da TFGe pelo CKD-EPI*
Razão proteinúria/creatininúria ou albuminúria/creatininúria
Glicemia de jejum e hemoglobina glicada (se risco de DM ou síndrome metabólica)
Colesterol total, LDLc, HDLc e triglicerídeos plasmáticos**
Ácido úrico plasmático
Eletrocardiograma convencional

CKD-EPI: Chronic Kidney Disease Epidemiology Collaboration; DM: diabetes mellitus; HDLc: colesterol de lipoproteína de alta densidade (high-density lipoprotein cholesterol); LDLc: colesterol de lipoproteína de baixa densidade (low-density lipoprotein cholesterol); TFGe: taxa de filtração glomerular estimada.

* A equação do CKD-EPI utiliza sexo, idade e valor da creatinina plasmática para o cálculo da TFGe.^85^

** O LDLc pode ser calculado pela seguinte fórmula: LDLc = colesterol total – (HDLc + triglicerídeos/5) (quando a dosagem de triglicerídeos for abaixo de 400 mg/dL.^86^ O LDLc também tem sido dosado por alguns laboratórios, fazendo parte da rotina laboratorial.

### 4.4. Lesões de Órgãos-Alvo

A avaliação de LOA deveria ser realizada em todo paciente no momento do diagnóstico da HA. A escolha do(s) método(s) pode variar em função dos recursos disponíveis. Eletrocardiograma, ecocardiograma, avaliação da taxa de filtração glomerular estimada (TFGe) e albuminúria são, hoje, ferramentas amplamente disponíveis e de comprovado valor agregado na estratificação de risco e na definição de metas terapêuticas para o paciente com HA.^87^ É recomendável que a avaliação de LOA seja repetida ao longo do tempo para monitorar sua evolução (progressão, estabilização ou regressão) e, consequentemente, orientar as intervenções terapêuticas mais oportunas.^88,89^ Os critérios utilizados para detecção de LOA estão demonstrados no Quadro 4.4.^81,90-96^

**Quadro 4.4:** Critérios para lesão de órgãos-alvo

Órgão	Método	Critérios
Coração	ECG	Índice Sokolow-Lyon (onda S de V1 + onda R de V5 ou V6) > 35 mm; onda R de aVL ≥ 11 mm; Cornell índice (S de V3 + R de aVL) > 28 mm em homens e > 20 mm em mulheres.^90,91^
	ECO	IMVE ≥ 116 g/m^2^ nos homens ou ≥ 96 g/m^2^ nas mulheres (indexada por SC) ou ≥ 45 g/m^2^ nos homens ou ≥ 49 g/m^2^ nas mulheres (indexada por altura^2,7^).^92^
Grandes vasos	ITB	ITB < 0,9.^81^
	USG de carótidas	Presença de placas.^93^
	VOP	VOP carotídeo-femoral ≥ 10 m/s.^94^
Rim	TFGe	Doença renal crônica a partir do Estágio 3a (TFGe < 60 mL/min/1,73 m^2^).^95^
	Albuminúria	Albuminúria ≥ 30 mg/24 h ou relação albuminúria/creatininúria ≥ 30 mg/g.^95^
Retina	FO	Retinopatia hipertensiva (KWB).^96^

ECG: eletrocardiograma; ECO: ecocardiograma; FO: fundo de olho; KWB: Keith–Wagener–Barker; IMVE: índice de massa ventricular esquerda; ITB: índice tornozelo-braquial; SC: superfície corpórea; TFGe: taxa de filtração glomerular estimada; USG: ultrassonografia; VOP: velocidade de onda de pulso.

O tempo necessário para detectar mudanças significativas nas LOAs após ajustes terapêuticos varia de acordo com o órgão-alvo e o método utilizado. Quando presente, a albuminúria e VOP são os parâmetros que costumam responder mais rapidamente ao controle anti-hipertensivo (de semanas a poucos meses).^97^ Embora não disponhamos de estudos clínicos especificamente desenhados para avaliar a periodicidade ideal no monitoramento das LOAs, pacientes com controle da PA inadequada e/ou evidência de LOA na avaliação inicial deveriam ser reavaliados com maior frequência (a cada 6 meses a 1 ano) até que a meta pressórica seja atingida e a LOA seja estabilizada ou revertida.^88,98^

### 4.5. Avaliação de Doença Renal Crônica

A DRC é definida como anormalidades da estrutura ou da função renal presentes por no mínimo 3 meses e com implicações para a saúde.^95^ Possui cinco estágios evolutivos de acordo com a TFGe, listados na Figura 4.2, conforme recomendado pelo KDIGO (*Kidney Disease Improving Global Outcomes*).^95^ Em qualquer um desses estágios, especialmente a partir do G3a (TFGe < 60 mL/min/1,73 m^2^), há aumento do risco de DCV, desfechos renais específicos e de mortalidade CV e por todas as causas.^95,99^

**Figura 4.2:**
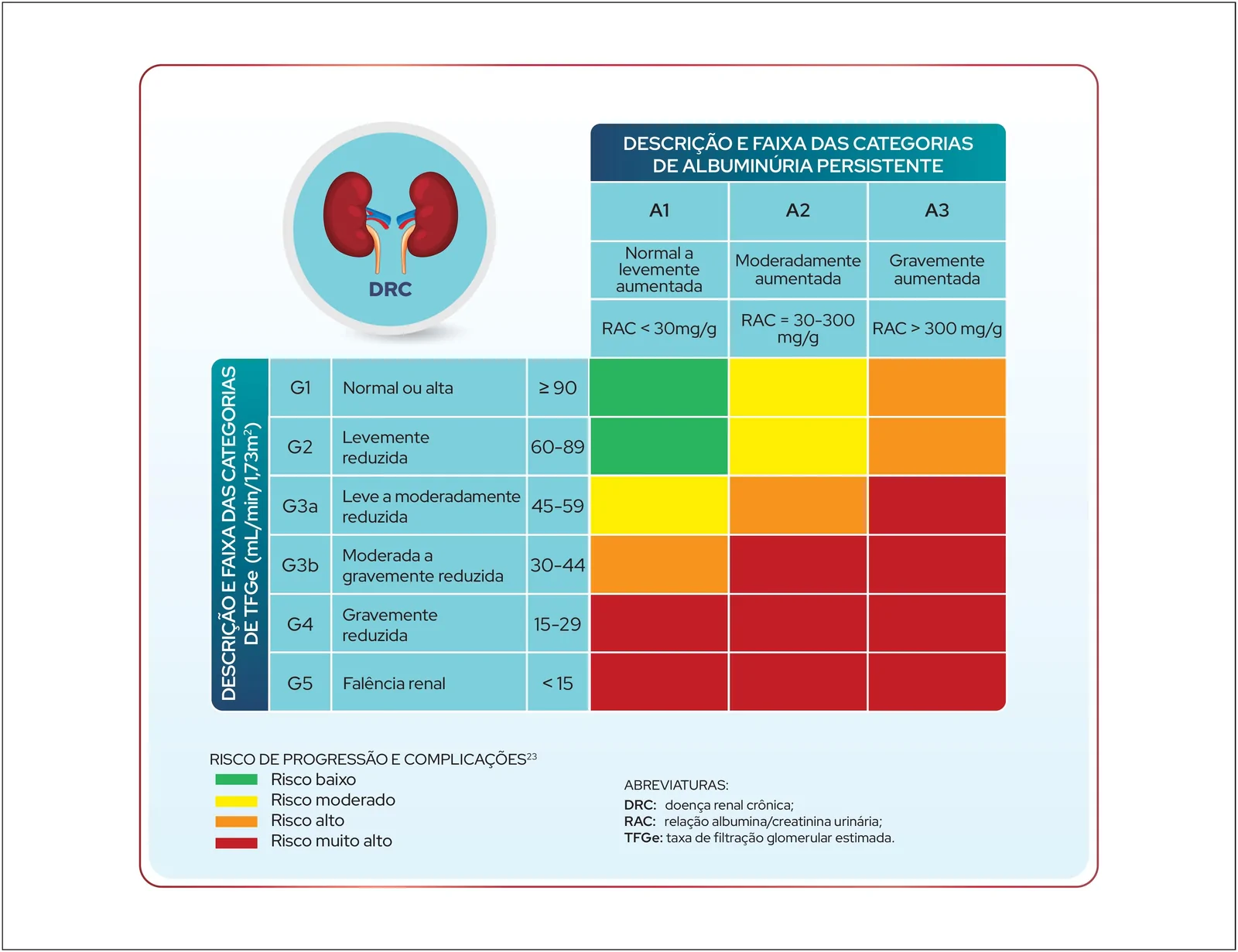
Avaliação e prognóstico da doença renal crônica. Adaptado de KDIGO 2024 Clinical Practice Guideline for the Evaluation and Management of Chronic Kidney Disease.^95^

Vale ressaltar que a relação albuminúria/creatininúria (RAC) ≥ 30 mg/g está associada a maior mortalidade e maior taxa de eventos CV, mesmo em pacientes com função renal preservada (Figura 4.2).^95^ Para avaliar o risco CV em pacientes com DRC, é fundamental utilizar calculadoras que levem em consideração a função renal do paciente, uma vez que calculadoras que excluam a TFGe podem gerar estimativas de risco CV subestimadas para pacientes com DRC.^95^

### 4.6. Avaliação de Causas Secundárias

A HA secundária é a forma de HA decorrente de uma causa identificável, que pode ser tratada com uma intervenção específica, a qual determina a cura ou a melhora do controle pressórico. A real prevalência de HA secundária é desconhecida, mas é estimada entre 5 e 10%.^100^ Ela deve ser investigada diante de indícios (história clínica, exame físico ou exames de rotina) que levem à sua suspeita clínica (Quadro 4.5). Além disso, os pacientes devem ser questionados sobre o uso de hormônios, medicamentos e demais substâncias que possam elevar a PA ou dificultar seu controle, o que está descrito no Capítulo 2.

**Quadro 4.5:** Principais causas de hipertensão arterial secundária, não endócrinas e endócrinas, sinais indicativos e rastreamento diagnóstico

Suspeita diagnóstica	Achados clínicos	Achados adicionais
Causas não endócrinas		
Doença renal parenquimatosa	Edema, anemia, anorexia, fadiga, creatinina e ureia elevadas e alterações do sedimento urinário ou nos exames de imagem.	Creatinina e cálculo da TFGe, USG renal, exame sumário de urina para pesquisa de proteinúria e/ou hematúria dismórfica. Dosagem de albuminúria ou relação proteinúria/creatininúria.
Estenose de artéria renal	HA de aparecimento súbito ou com piora sem causa aparente antes dos 30 ou após os 55 anos, HAR ou HARf ou HAM, sopro abdominal, EAP súbito, alteração da função renal inexplicável ou por medicamentos que bloqueiam o SRAA, assimetria entre o tamanho dos rins > 1,5 cm.	USG com Doppler renal com medida de velocidade de fluxo e índice de resistividade (rastreio, mas observador dependente), angiografia por RNM ou TC espiral. Padrão-ouro: arteriografia renal convencional.
Apneia obstrutiva do sono	Maior frequência em homens ou mulheres pós-menopausa, ronco na maioria das noites, fragmentação do sono com pausas ou engasgos, sonolência excessiva diurna, sono não reparador, fadiga, nictúria, cefaleia matinal, síndrome metabólica.	Questionários apresentam baixa precisão para triagem. Padrão-ouro: polissonografia ou poligrafia (para pacientes com probabilidade pré-teste alta). IAH < 5 eventos/h: sem AOS; IAH 5-14,9 eventos/h: AOS leve; IAH 15-29,9 eventos/h: AOS moderada; IAH ≥ 30 eventos/h: AOS importante (ou grave).
Coarctação de aorta	Fraqueza em MMII, pulsos em MMII ausentes ou de amplitude diminuída, HA com PAS 10 mmHg > em MMSS com relação aos MMII, sopro sistólico interescapular e no tórax.	Radiografia de tórax, ecocardiograma para rastreio. Angiografia de tórax por TC ou, preferencialmente, RNM aorta (padrão-ouro). Angiografia invasiva, apenas quando forem necessários dados adicionais.
Causas endócrinas		
Hiperaldosteronismo primário (hiperplasia ou adenoma)	HAR ou HARf e/ou com hipopotassemia (não obrigatória) e/ou com nódulo adrenal.	Determinações de aldosterona e atividade/concentração de renina plasmática e testes confirmatórios. (Teste da infusão salina, Teste do Captopril, Teste da fludrocortisona, Teste da furosemida intravenosa). Exames de imagem: TC com cortes finos ou RNM. Coleta seletiva de aldosterona e cortisol em veias adrenais, para identificar subtipo, quando indicado.
Feocromocitoma e paragangliomas	HA paroxística com a tríade composta por cefaleia, sudorese e palpitações.	Metanefrinas plasmáticas livres e metanefrinas fracionadas urinárias (rastreamento), RNM e cintilografia nos casos indicados
Hipotireoidismo	Fadiga, ganho de peso, perda de cabelo, HA diastólica, fraqueza muscular, sonolência.	Rastreamento: TSH e T4 livre.
Hipertireoidismo	Intolerância ao calor, perda de peso, taquicardia/palpitações, exoftalmia, hipertermia, reflexos exaltados, tremores, bócio.	Rastreamento: TSH e T4 livre.
Hiperparatireoidismo (hiperplasia ou adenoma)	Litíase urinária, osteoporose, depressão, letargia, fraqueza ou espasmos musculares, sede, poliúria, polidipsia, constipação	Cálcio total e/ou iônico, fósforo, PTH, calciúria de 24 h e dosagem de vitamina D
Síndrome de Cushing (hiperplasia, adenoma e excesso de produção de ACTH)	Ganho de peso, diminuição da libido, fadiga, hirsutismo, amenorreia, "fácies em lua cheia", "giba dorsal", estrias purpúreas, obesidade central, hipopotassemia.	Cortisol basal, cortisol salivar à meia-noite, cortisol urinário livre de 24 h e teste de supressão com dexametasona ou betametasona: tomar dexametasona 1 mg às 23-24 h e dosar cortisol matinal (7-8 h) do dia seguinte. Exames de imagem: TC, RNM.
Obesidade Classe 1: IMC de 30 a < 35 kg/m^2^ Classe 2: IMC de 35 a < 40 kg/m^2^ Classe 3: IMC ≥ 40 kg/m^2^	Aumento da gordura central ou generalizada.	IMC (peso em kg/altura em m^2^) e da circunferência abdominal (> 102 cm homem e 88 cm mulher) Exames de imagem: DEXA (padrão-ouro), TC, RNM (estudos clínicos).
Acromegalia	HA em até 30% dos casos, além de diabetes, HVE e AOS. Outros sintomas: defeitos visuais, paralisia de nervos cranianos, cefaleia, macrognatia, crescimento de pés e mãos, hipertrofia de tecidos moles, macroglossia, complicações musculoesqueléticas.	Dosagem de IGF-1, nível sérico de GH e GH pós-sobrecarga oral de glicose. Localização: RNM de sela túrcica (preferencial) ou TC de sela túrcica.

ACTH: adrenocorticotropina; AOS: apneia obstrutiva do sono; DEXA: dual-energy x-ray absorptiometry scanning; EAP: edema agudo de pulmão; GH: hormônio do crescimento; HA: hipertensão arterial; HAM: hipertensão arterial maligna; HAR: hipertensão arterial resistente; HARf: hipertensão arterial refratária; HVE: hipertrofia ventricular esquerda; IAH: índice de apneia e hipopneia; IGF-1: fator de crescimento insulina-símile tipo 1; IMC: índice de massa corporal; MMII: membros inferiores; MMSS: membros superiores; PAS: pressão arterial sistólica; PTH: paratormônio; RNM: ressonância nuclear magnética; SRAA: sistema renina-angiotensina-aldosterona; TC: tomografia computadorizada; TFGe: taxa de filtração glomerular estimada; TSH: hormônio tireoestimulante; USG: ultrassonografia.

Os pacientes com HA secundária estão, habitualmente, sob maior risco CV e renal e apresentam maior impacto nos órgãos-alvo, especialmente devido a níveis mais elevados e sustentados de PA. Informações mais aprofundadas sobre diagnóstico e tratamento das causas secundárias de HA estão disponíveis nas "Diretrizes Brasileiras de Hipertensão Arterial de 2020".^1^

### 4.7. Cálculo do Risco Cardiovascular

Entre os escores mais utilizados para a estratificação de risco CV, estão o SCORE2 (*Systematic Coronary Risk Evaluation 2*), desenvolvido pela Sociedade Europeia de Cardiologia (European Society of Cardiology – ESC) em conjunto com a Sociedade Europeia de Hipertensão (European Society of Hypertension – ESH),^101^ e o PREVENT (*Predicting Risk Of Cardiovascular Disease Events*), recentemente validado pela American Heart Association (AHA).^74^ Esses escores avaliam o risco CV individual e podem ser facilmente acessados por meio de calculadoras digitais.

O SCORE2, uma evolução do escore de risco SCORE, incorpora variáveis como idade, sexo, níveis de colesterol total, PAS, tabagismo e a presença de DM. Ele é ajustado de acordo com o nível de risco CV basal de diferentes populações europeias, reconhecendo as disparidades regionais na epidemiologia das DCV.^101^ Já o escore PREVENT, além de considerar variáveis tradicionais, como idade, PAS, níveis de colesterol, tabagismo e DM, inclui marcadores metabólicos e renais adicionais, como TFGe, RAC e a hemoglobina glicada, refletindo um avanço na identificação precoce de lesões subclínicas e risco CV aumentado.^74^ Essa calculadora foi desenvolvida a partir de estudos com mais de 6 milhões de indivíduos de diferentes etnias, regiões geográficas e condições socioeconômicas.^102^

A presente diretriz sugere o uso do escore PREVENT, proposto pela AHA, por ser a única a incluir critérios considerando a existência de uma síndrome cardiorrenal e metabólica. Além disso, a calculadora de risco PREVENT estima o risco de desenvolver DCV em 10 e em 30 anos para pacientes entre 30 e 79 anos de idade. Portanto, o PREVENT faz uma estimativa do risco a longo prazo, identificando de forma mais acurada o risco CV, especialmente para os pacientes mais jovens.

A calculadora PREVENT está disponível no site da American Heart Association (AHA): https://professional.heart.org/en/guidelines-and-statements/prevent-calculator. A estratificação de risco utilizando a calculadora PREVENT para estimativa do risco de doença cardiovascular aterosclerótica (ou ASCVD em inglês) em 10 anos é realizada com base nos seguintes pontos de corte percentuais: baixo risco: <5,0%; risco limítrofe: de 5,0% até <7,5%; risco intermediário: de 7,5% até <20%; alto risco: ≥20%.

Embora o escore PREVENT derive de dados de população mais diversa e miscigenada que o SCORE2, deve ser ressaltada a ausência de dados populacionais brasileiros nesses modelos de estimativa de risco, o que os pode tornar menos precisos na avaliação do risco CV em nossa população. Em outras palavras, o risco aferido pelos escores internacionais pode estar subestimado por deixar de considerar os fatores de risco mais prevalentes ou relevantes em nossa população.

Por outro lado, deve-se reconhecer que o acesso à calculadora PREVENT pode não ser universal em alguns cenários clínicos. Nessas circunstâncias, a análise de risco pode ser realizada conforme sugerido no Quadro 4.6, muito embora a acurácia dessa estratégia seja limitada e não validada.

**Quadro 4.6:** Classificação dos estágios de hipertensão arterial de acordo com o nível de pressão arterial, presença de fatores de risco cardiovascular, lesão de órgão-alvo ou doença cardiovascular estabelecida

	PA (mmHg)			
Fatores de risco CV, presença de LOA ou DCV	PAS 130-139 PAD 80-89	Estágio 1 PAS 140-159 PAD 90-99	Estágio 2 PAS 160-179 PAD 100-109	Estágio 3 PAS ≥ 180 PAD ≥ 110
Sem fatores de risco CV	RISCO BAIXO	RISCO BAIXO	RISCO MODERADO	RISCO ALTO
1 ou 2 fatores de risco CV	RISCO BAIXO	RISCO MODERADO	RISCO ALTO	RISCO ALTO
≥ 3 fatores de risco CV	RISCO MODERADO	RISCO ALTO	RISCO ALTO	RISCO ALTO
LOA, DRC Estágio 3 ou DM	RISCO ALTO	RISCO ALTO	RISCO ALTO	RISCO MUITO ALTO
DCV estabelecida ou DRC Estágio ≥ 4	RISCO MUITO ALTO	RISCO MUITO ALTO	RISCO MUITO ALTO	RISCO MUITO ALTO

CV: cardiovascular; DCV: doença cardiovascular; DM: diabetes mellitus; DRC: doença renal crônica; LOA: lesão em órgão-alvo; PA: pressão arterial; PAD: pressão arterial diastólica; PAS: pressão arterial sistólica. Observação: são considerados fatores de risco CV para essa análise: sexo masculino; idade: > 55 anos no homem e > 65 anos na mulher; DCV prematura em parentes de 1^o^ grau (homens < 55 anos e mulheres < 65 anos); tabagismo; dislipidemia: colesterol de lipoproteína de baixa densidade (*low-density lipoprotein cholesterol* – LDLc) ≥ 100 mg/dL e/ou não colesterol de lipoproteína de alta densidade (*high-density lipoprotein cholesterol* – HDLc) 130 mg/dL e/ou HDLc ≤ 40 mg/dL no homem e ≤ 50 mg/dL na mulher e/ou triglicerídeos (TG) > 150 mg/dL; obesidade (IMC ≥ 30 kg/m^2^).

### 4.8. Aspectos Adicionais e Desafios na Avaliação do Risco Cardiovascular na Hipertensão Arterial

Várias condições clínicas podem interferir na estratificação do risco CV do paciente com HA e, entre elas, destaca-se a idade. Em curto prazo, enquanto os pacientes idosos têm maior risco absoluto, os jovens apresentam menor risco absoluto, mesmo com perfil de risco desfavorável.^45^ No longo prazo ou ao longo da vida, a influência da idade sobre o risco CV inverte-se, passando a ter maior perda de longevidade aqueles que manifestam os fatores de risco em idade jovem.

Portanto, uma limitação relevante na avaliação do risco CV envolve a duração da exposição à HA ou aos fatores de risco. A duração da HA e do tratamento anti-hipertensivo, assim como dos valores de PA aos quais os pacientes foram expostos durante longos períodos, podem interferir na estimativa de risco.^103^ Além disso, escores que utilizam a opção binária (sim/não) para condições clínicas como DM e tabagismo na avaliação do risco CV não consideram a duração dessas condições. Portanto, pacientes com maior tempo de exposição terão maior risco CV quando comparados com indivíduos com menor tempo de exposição aos mesmos fatores.^45^ Por isso, as incorporações de novas ferramentas para a avaliação do risco relativo (RR) vs. risco vitalício (*lifetime risk*) e períodos de aumento do risco parecem ser necessárias, especialmente nos jovens com baixo risco absoluto, mas com alto RR para DCV.^102,104^ O conceito de "idade vascular" (calculada pela VOP) e "idade cardiometabólica" podem auxiliar nessa estratégia.^105,106^

Medidas de PA realizadas fora do consultório por meio de MAPA e MRPA apresentam melhor valor prognóstico do que a medida de PA no consultório.^107^ Entretanto, medidas de PA obtidas pela MAPA ou MRPA, assim como a duração do tratamento anti-hipertensivo e o histórico dos valores de PA, ainda não estão incorporadas nas calculadoras de risco atuais. Por outro lado, os fenótipos de PA derivados de medidas obtidas dentro e fora do consultório, como HM, HMNC, HAB e HABNC, podem ter valor prognóstico e são úteis na avaliação de risco e manejo dos pacientes.^108-110^

Em comparação com a normotensão, a HM apresenta pior prognóstico CV, o qual é similar à HA sustentada, enquanto a HMNC apresenta pior prognóstico em comparação com a HA controlada, tendendo a ser similar à HA sustentada não controlada.^108^ Portanto, é recomendado que os pacientes com HM e HMNC sejam rotineiramente estratificados do ponto de vista CV da mesma forma que os pacientes com HA sustentada e HA sustentada não controlada, incluindo a avaliação de LOA.

No que se refere à HAB, diversas evidências apontam que esse fenótipo apresenta risco CV intermediário entre normotensão e HA sustentada, muito embora os mecanismos que medeiam esse aumento de risco não estejam bem estabelecidos.^109^ Portanto, sugere-se que os pacientes com HAB sejam regularmente avaliados do ponto de vista de estratificação de risco CV e LOA. Por outro lado, a HABNC parece não apresentar maior risco de eventos CV quando comparada à HA controlada, embora algumas evidências apontem para uma maior presença de LOA.^109,110^

A incorporação de novos marcadores para a estratificação do risco CV também pode ser útil para uma abordagem mais precisa e personalizada no manejo do paciente com HA. Nesse contexto, o uso de biomarcadores como lipoproteína(a),^111^ troponina ultrassensível e peptídeos natriuréticos (NT-proBNP e BNP),^112^ bem como métodos de imagem mais avançados, como o escore de cálcio coronariano,^113^ parecem estar emergindo como ferramentas adicionais na estratificação do risco CV.

Por fim, é importante destacar que a estratificação de risco CV em indivíduos com pré-hipertensão é fundamental para a orientação do início do tratamento anti-hipertensivo e para o controle mais adequado dos fatores de risco CV. Por outro lado, a avaliação do risco CV nos indivíduos com HA permite uma abordagem mais precisa e personalizada no manejo de medicamentos e direcionamento de metas para controle dos fatores de risco CV.

Fluxogramas para estratificação de risco CV em indivíduos com pré-hipertensão e HA estão apresentados na Figura 4.3.

**Figura 4.3:**
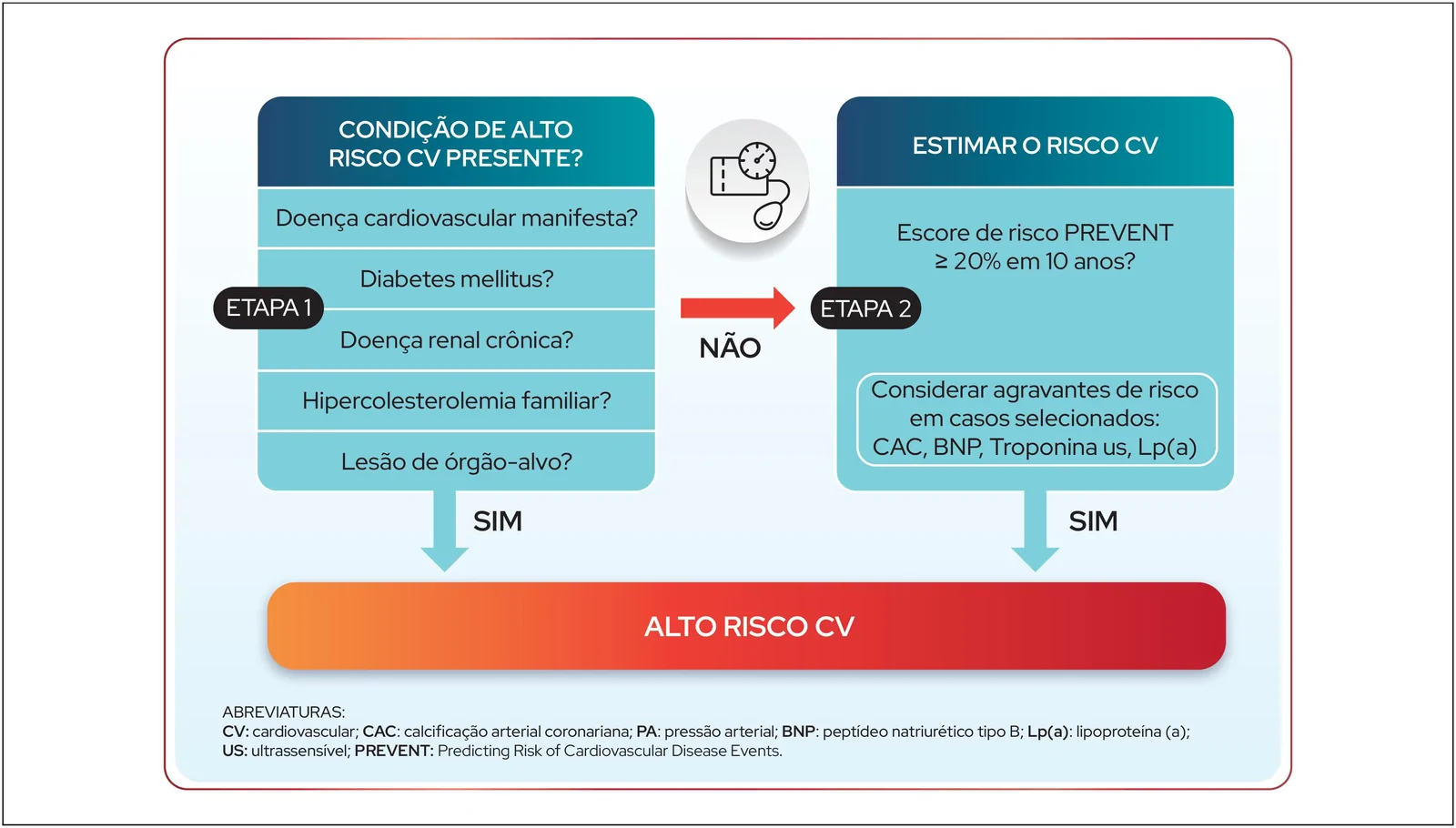
Estratificação de risco cardiovascular dos indivíduos com pré-hipertensão e hipertensão arterial.

**Table:** 

Recomendações para avaliação clínica e complementar, e estratificação de risco cardiovascular	Forca da recomendação	Certeza da evidência
Recomenda-se realizar anamnese e exame físico com o objetivo de se obter um diagnóstico mais preciso e ajudar na identificação de causas secundárias de HA e LOA.	FORTE	BAIXA
Recomenda-se pesquisar FRCV e LOA idealmente em todos os pacientes no momento do diagnóstico de HA e repetir essa avaliação pelo menos anualmente, sendo a escolha do método decidida conforme os recursos disponíveis.	FORTE	BAIXA
Recomenda-se verificar a presença de doença renal e classificá-la de acordo com o KDIGO 2024.	FORTE	MODERADA
Recomenda-se avaliar o risco CV utilizando o escore PREVENT.	FORTE	ALTA
Recomenda-se estratificar o risco CV em indivíduos com pré-hipertensão com o objetivo de orientar o início do tratamento anti-hipertensivo e promover controle mais adequado dos FRCVs.	FORTE	ALTA
Recomenda-se estratificar o risco CV em pacientes com HA com o objetivo de promover uma abordagem mais precisa e personalizada no manejo de medicamentos e direcionamento de metas para controle dos FRCVs.	FORTE	ALTA

CV: cardiovascular; FRCVs: fatores de risco cardiovascular; HA: hipertensão arterial; KDIGO: Kidney Disease Improving Global Outcomes; LOA: lesão de órgãos-alvo; PREVENT: Predicting Risk of Cardiovascular Disease Events.

## 5. Início de Tratamento e Metas Terapêuticas

### 5.1. Início de Tratamento

É discutível se a tomada de decisão para o início do tratamento deve se basear nos níveis pressóricos ou no risco CV associado. Uma metanálise publicad a em 2021 pelo *The Blood Pressure Lowering Treatment Trialists’ Collaboration*^114^ sugere que a redução da PA em valor fixo é igualmente eficaz na prevenção primária e secundária para a redução de eventos CV, mesmo em níveis pressóricos considerados "normais" e geralmente não considerados para o tratamento medicamentoso. Quanto maior for o risco individual, maior será o efeito em redução de desfechos para aquele indivíduo.

Em adição a essa informação, uma metanálise publicada em 2018 por Karmali *et al*.^115^ compara a estratégia baseada em metas de PA com aquela baseada no risco CV, e os resultados sugerem que a decisão de iniciar o tratamento baseada no risco CV é mais eficaz. No entanto, mesmo considerando que essa metanálise é de boa qualidade, implicando uma alta certeza da evidência, recomenda-se que a decisão para o início do tratamento considere a combinação de ambos.

Indivíduos com PA ≥ 120/80 mmHg devem adotar MNM com o objetivo de manter a PA < 130/80 mmHg e reduzir o risco CV (ver Capítulo 6). Para a decisão de início de tratamento, a estimativa de risco CV é importante para os pacientes com PA 130-139 e/ou 80-89 mmHg. Uma metanálise com 24 ensaios clínicos randomizados (ECRs) e 47.991 indivíduos investigou o efeito da redução da PA sobre desfechos CV em indivíduos com PA na faixa de pré-hipertensão e demonstrou redução significativa do risco de AVC (60%) para reduções de PAS/PAD de 10/5 mmHg em pacientes de alto e muito alto risco CV.^116^ No entanto, a metanálise de Karmali *et al*. questiona esses efeitos protetores.^115^

Para os pacientes com HA, a estratificação do risco CV não deve interferir na decisão de iniciar o tratamento. Os benefícios de tratar os pacientes com HA sem outros fatores de risco associados e que apresentem valores persistentes de PA significativamente elevados já estão bem estabelecidos e vêm sendo sistematicamente recomendados por diretrizes nacionais e internacionais.^1,44,45,117^

Assim, esta diretriz utiliza dados de metanálises com dados individuais de participantes de estudos randomizados conduzidos em indivíduos com HA Estágio 1 sem DCV prévia para a definição da melhor conduta.^118,119^ Um desses estudos evidenciou que o tratamento do paciente com HA de baixo risco não reduziu os desfechos de DAC, os eventos CV ou a mortalidade CV, em um seguimento de 4 a 5 anos.^118^ No entanto, houve uma tendência para a redução de AVC e mortalidade total, sendo que tais diminuições seriam provavelmente obtidas com um tempo de seguimento maior e/ou mais participantes incluídos nos estudos.

Uma segunda publicação demonstrou que, com o tratamento, havia redução de DCV e de mortalidade quando a PA inicial era ≥ 140/90 mmHg.^119^ No Estágio 1, houve redução significativa da mortalidade, mas a redução de eventos CV maiores foi menos relevante. Todos esses achados foram reforçados pelos resultados de uma análise de subgrupo do estudo HOPE-3 (*Heart Outcomes Prevention Evaluation 3*).^114^ Nesse estudo, naqueles pacientes classificados como Estágio 1 e risco CV intermediário, o tratamento anti-hipertensivo promoveu redução média de 6 mmHg na PAS e redução de 27% na taxa de eventos CV combinados em pacientes com PAS no consultório maior que 143 mmHg.^114^ Com base em tais dados, o tratamento medicamentoso deve ser iniciado junto com o tratamento não medicamentoso quando a PA no consultório for ≥ 140/90 mmHg.

As indicações para o início do tratamento não medicamentoso e medicamentoso estão apresentadas Tabela 5.1 e Figura 5.1.^114,116,118-120^

**Tabela 5.1:** Indicações de medidas não medicamentosas e medicamentosas de acordo com a pressão arterial e a idade

Tratamento	População-alvo	Início do tratamento
Medidas não medicamentosas	PA ≥ 120/80 mmHg	No diagnóstico
Tratamento medicamentoso	PA ≥ 140/90 mmHg	No diagnóstico
	PA 130-139/80-89 mmHg e alto risco CV	Quando não há controle da PA após 3 meses de MNM
	Idosos ≥ 60 anos	Quando PAS ≥ 140 mmHg
	Idosos ≥ 80 anos	Quando PAS ≥ 140 mmHg*

CV: cardiovascular; MNM: medidas não medicamentosas; PA: pressão arterial; PAS: pressão arterial sistólica.

* Avaliar com cautela e individualizar a estratégia em pacientes com história prévia de hipotensão ortostática, fragilidade moderada a grave e expectativa de vida menor que 3 anos.^114,116,118-120^

**Figura 5.1:**
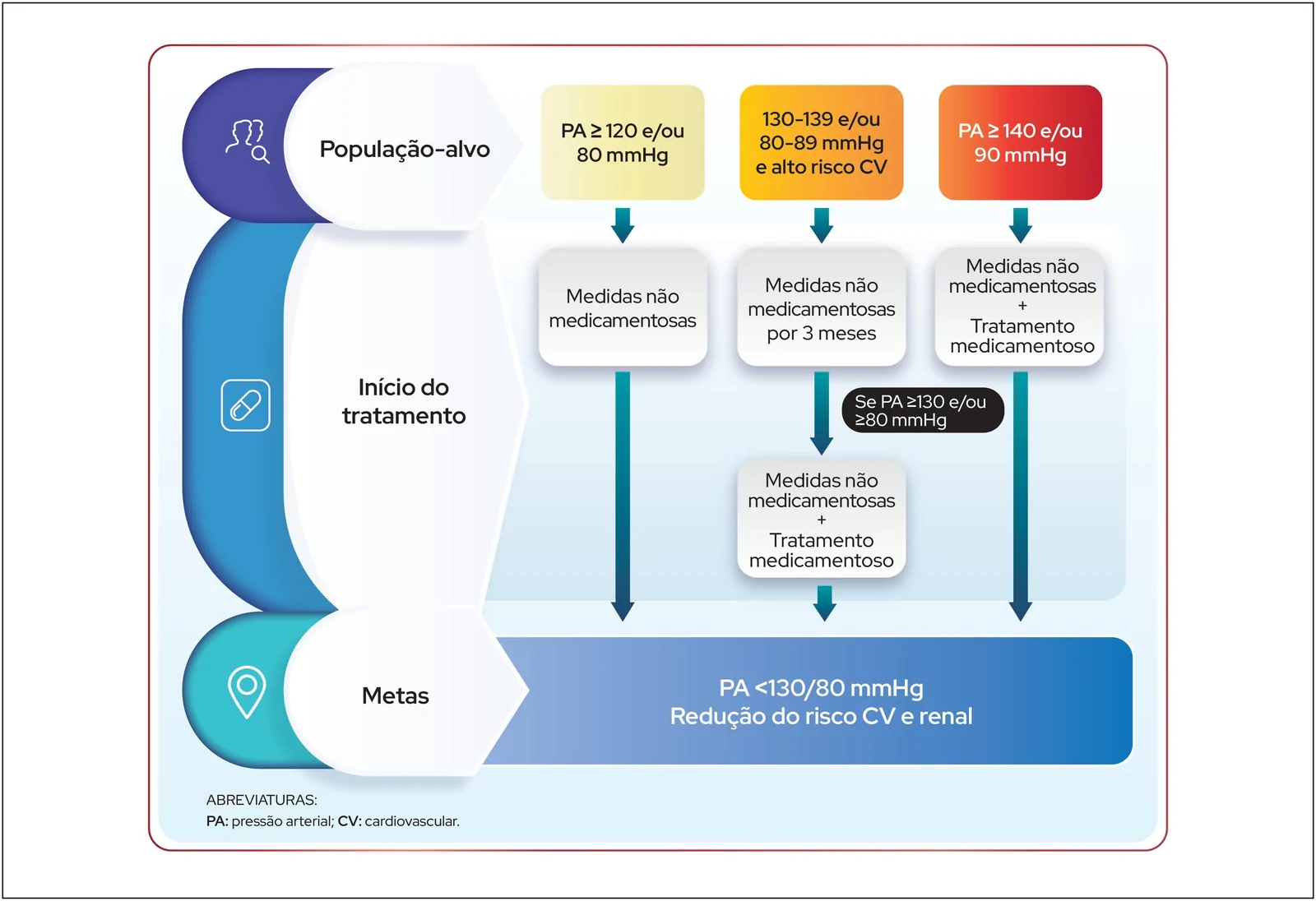
Início de tratamento e metas de pressão arterial.

### 5.2. Metas de Pressão Arterial

Com relação à meta de PA no paciente com HA de baixo risco CV, uma metanálise e dados de um grande estudo observacional sugerem que o alcance de PA < 140/90 mmHg é acompanhado por reduções significativas do risco de mortalidade, AVC e DRC em estágio final; no entanto, as maiores reduções do risco de mortalidade total foram observadas com valores de PAS menores que 130 mmHg.^121,122^ De acordo com revisão sistemática (RS) e metanálise publicada pela SBC, a recomendação desta diretriz é alcançar meta de PA < 130 × 80 mmHg mesmo em pacientes de risco baixo ou intermediário (Figura 5.1).^123^

Independentemente do nível pressórico, se estiver dentro da faixa considerada pré-hipertensão ou nos diferentes estágios de HA, a presença de DCV, de DM ou DRC, de hipercolesterolemia familiar, de LOA ou estimativa de risco ≥ 20% em 10 anos pelo escore de risco PREVENT são condições de alto ou muito alto risco CV.^124^

No estudo SPRINT (*Systolic Blood Pressure Intervention Trial*), realizado com pacientes sem diabetes de alto risco cardiovascular, a PAS média foi de 134,6 mmHg no grupo de tratamento padrão e de 121,5 mmHg no grupo de tratamento intensivo ao longo dos 3,26 anos de acompanhamento, resultando em taxas mais baixas de eventos CV fatais e não fatais e morte por qualquer causa.^125^ Alguns eventos adversos foram mais observados no grupo de tratamento intensivo. Assim, de uma forma geral, a meta de PA a ser atingida em pacientes de alto risco CV pode ser uniformizada para valores < 130/80 mmHg.^123^

A análise conjunta dos estudos SPRINT e ACCORD (*Action to Control Cardiovascular Risk in Diabetes*) mostrou que, quanto maior for o tempo na faixa de controle da PA, menores serão os riscos de eventos adversos renais e CV,^46^ destacando a importância da adesão e persistência ao tratamento.

### 5.3. Metas de Pressão Arterial na Medida Ambulatorial da Pressão Arterial e Medida Residencial da Pressão Arterial

Vale ainda considerar que, idealmente, devemos ter a medida da PA realizada tanto no consultório quanto no domicílio por meio da MAPA ou MRPA. Entre as várias vantagens das medidas dentro e fora do consultório, uma das mais importantes é identificar os diferentes fenótipos do comportamento da PA, o que implica em estratificações de risco e estratégias de tratamento distintas, assim como, em alguns cenários, utilizar metas guiadas pela MAPA ou MRPA.^126^

Nos fenótipos de HAB e HM, é recomendável sempre orientar as MNM e a terapia medicamentosa, quando indicada; nessas condições, as metas de controle da PA devem ser guiadas pelas medidas dentro e fora do consultório, de acordo com os valores de referência apresentados no Tabela 5.2.

**Tabela 5.2:** Metas de pressão arterial na medida ambulatorial da pressão arterial (nas 24 horas, vigília e durante o sono) e na medida residencial da pressão arterial

	PAS (mmHg)	PAD (mmHg)
MAPA 24 horas	< 130	< 80
MAPA vigília	< 135	< 85
MAPA sono	< 120	< 70
MRPA	< 130	< 80

MAPA: monitoração ambulatorial da pressão arterial; MRPA: monitoração residencial da pressão arterial; PAD: pressão arterial diastólica; PAS: pressão arterial sistólica.^126^

### 5.4. Curva em J da Pressão Arterial

No tratamento da HA, a redução excessiva da PA pode estar associada a um aumento no risco de eventos CV. Esse fenômeno sugere uma relação em forma de J ou U, em que tanto pressões muito altas quanto muito baixas estão associadas a riscos aumentados. Ou seja, haveria um limite abaixo do qual uma redução adicional poderia aumentar o risco de eventos adversos.^127,128^

A curva J é mais observada em relação à PAD, mais comumente em pacientes com DAC estabelecida. A principal hipótese é que valores muito baixos de PAD resultam em diminuição de fluxo na circulação coronariana. No entanto, dados observacionais nem sempre demonstram consistentemente uma curva J da PA em relação ao risco CV.^127,128^

Dados observacionais mais recentes demonstraram que a curva em J da PA não parece ser um processo causal, podendo haver diversos fatores de confusão.^128-130^ Um estudo de randomização mendeliana não linear não encontrou evidências de que a redução da PAD aumente o risco de eventos CV, sugerindo que o risco de IAM diminui consistentemente com a redução da PAD, mesmo em valores baixos.^114^ Em resumo, não há evidências que indiquem que uma PA baixa seja a causa de um estado de alto risco CV, mas simplesmente um marcador desse risco, especialmente entre adultos mais idosos e aqueles com comorbidades.

Revisões sistemáticas e metanálises recentes que avaliaram populações com DCV prévia e reduções da PAS além de 120 mmHg encontraram evidências robustas que sugerem haver benefício adicional na redução de desfechos duros.^131^

### 5.5. Reduções Intensas da Pressão Arterial e Risco de Eventos Adversos

A redução mais intensa dos valores da PA sempre suscita a discussão sobre possível aumento na incidência de efeitos colaterais graves, especialmente nos pacientes idosos e/ou frágeis. Uma metanálise recente que avaliou 20.895 indivíduos idosos tratados para a obtenção de metas mais rigorosas observou redução de 29% nos principais eventos CV, sem aumento nas reações adversas graves.^132^

Esses e outros achados reforçam a recomendação de metas mais intensas na redução da PA para os indivíduos com HA, independentemente da idade. Devemos estar atentos, no entanto, aos pacientes frágeis, aos muito idosos (≥ 80 anos), aos com expectativa de vida reduzida ou com características que possam aumentar o risco de efeitos adversos.^131,133^ Quando não for tolerada a meta < 130/80 mmHg, a PA deve ser reduzida até o menor valor tolerado.^117^

Uma pergunta importante e objeto de ensaios clínicos nos últimos anos é se reduções da PA abaixo de 120/70 mmHg, especialmente em pacientes com risco CV alto, adicionam maior proteção CV. Uma metanálise publicada em 2021 demonstrou que, para qualquer faixa inicial de PA (mesmo em indivíduos com valores sistólicos menores do que 120 mmHg), a cada redução de 5 mmHg, observou-se igual redução de 11% nos desfechos CV sem aumento relevante na incidência de eventos adversos.^131^

### 5.6. Considerações na Hipertensão Sistólica Isolada

Em um paciente com pressão de pulso (PP) ampla, PAS ≥ 140 mmHg e PAD < 90 mmHg, as evidências sugerem que a terapia anti-hipertensiva deve ser iniciada e intensificada, enquanto estiver bem tolerada, para reduzir o risco CV.^134,135^ Na HA sistólica isolada, obviamente, a meta de controle fica restrita à PAS. Na prática, esse controle pode ser limitado devido à redução concomitante e progressiva da PAD que já se encontra em níveis abaixo de 90 mmHg, podendo chegar a valores inferiores a 60 mmHg. No entanto, não existem evidências definitivas de um limite mínimo para a PAD nesse contexto, considerando que esses indivíduos são geralmente idosos e apresentam diferentes comorbidades. Como citado anteriormente, ainda não foi confirmada uma relação de causa-efeito entre uma PAD baixa e um maior risco CV.^128^ Por isso, a meta recomendada para controle da PA deve sempre ser buscada de forma individualizada, e o acompanhamento deve ser mais frequente, especialmente nos mais idosos com outras comorbidades.^134^

Alguns pacientes que conseguem atingir uma meta mais intensiva de PA, com valores abaixo de 120/70 mmHg, podem apresentar maior risco de hipotensão sintomática, síncope, injúria renal aguda e hipercalemia. Apesar de uma grande heterogeneidade nas métricas definidoras desses efeitos adversos, o controle mais rigoroso da PA deve ser mantido naqueles com boa tolerância ao tratamento anti-hipertensivo.^136^

A recomendação atual é que o tratamento da HA seja individualizado, considerando fatores como idade, fragilidade, polifarmácia e a presença de outras condições de saúde.^136^

**Table:** 

Mensagens principais sobre o início do tratamento
A adoção de medidas de MNM tem por objetivo a redução do risco CV. Todos os indivíduos com PA ≥ 120/80 mmHg devem iniciar e manter MNM, mesmo após o início do tratamento medicamentoso, quando indicado.
A estimativa de risco CV é importante para os indivíduos com PA 130-139 e/ou 80-89 mmHg, estando indicado o início do tratamento medicamentoso para indivíduos de alto risco CV.
Para os pacientes com HA, o início do tratamento medicamentoso independe da estratificação do risco CV.

CV: cardiovascular; HA: hipertensão arterial; MNM: medidas não medicamentosas; PA: pressão arterial.

**Table:** 

Recomendações para início de tratamento	Força da recomendação	Certeza da evidência
Recomendam-se MNM para todos os indivíduos com PA ≥ 120/80 mmHg.	FORTE	ALTA
Recomenda-se tratamento medicamentoso após 3 meses de MNM para indivíduos com PA 130-139/80-89 mmHg e alto risco CV.	FORTE	ALTA
Recomenda-se o início de tratamento medicamentoso para indivíduos com PA ≥ 140/90 mmHg.	FORTE	ALTA

CV: cardiovascular; MNM: medidas não medicamentosas; PA: pressão arterial. *Avaliar com cautela e individualizar a estratégia em pacientes com história prévia de hipotensão ortostática, fragilidade moderada a grave e expectativa de vida menor que 3 anos.^114,116,118-121^

**Table:** 

Mensagens principais sobre as metas de pressão arterial
Não há evidências consistentes da existência de curva em J da PA para o risco CV.
Considerar e monitorar a tolerabilidade dos pacientes para reduções intensas da PA, em especial nos indivíduos frágeis, com idade ≥ 80 anos, com hipotensão ortostática sintomática, ou com expectativa de vida reduzida.
Quando não for tolerada a meta de PA < 130/80 mmHg, a PA deve ser reduzida até o menor valor tolerado.
Na HAB e HM, avaliar o alcance das metas de PA pela MAPA ou MRPA.
Na HA sistólica isolada, a meta de PAS < 130 mmHg deve ser alcançada, e a redução da PAD seve ser monitorada em relação a possíveis eventos adversos.

CV: cardiovascular; HA: hipertensão arterial; HAB: hipertensão do avental branco; HM: hipertensão mascarada; MAPA: medida ambulatorial da pressão arterial; MRPA: medida residencial da pressão arterial; PA: pressão arterial; PAD: pressão arterial diastólica; PAS: pressão arterial sistólica.

**Table:** 

Recomendação para metas de pressão arterial	Força da recomendação	Certeza da evidência
Recomenda-se a meta de PA < 130/80 mmHg para pacientes com PA 130-139/80-89 mmHg e risco CV alto.	FORTE	ALTA
Recomenda-se a meta de PA < 130/80 mmHg para os pacientes com HA, independentemente de o risco CV ser baixo, moderado ou alto.	FORTE	ALTA
Recomenda-se que, para pacientes que não tolerem a meta de PA < 130/80 mmHg, deve-se reduzir a PA até o valor mais baixo tolerado.	FORTE	MODERADA
Recomenda-se confirmar o alcance da meta de PA com a medida da PA fora do consultório por meio da MAPA ou MRPA.	FORTE	BAIXA

CV: cardiovascular; HA: hipertensão arterial; MAPA: monitorização ambulatorial da pressão arterial; MRPA: monitorização residencial da pressão arterial; PA: pressão arterial.

## 6. Medidas Não Medicamentosas

Diversas intervenções não medicamentosas são sugeridas para a promoção da saúde, prevenção e controle da PA. Este capítulo apresenta as evidências mais recentes e o grau de recomendação de cada uma delas na abordagem da HA. Devido à abrangência e diversidade das MNM, apenas algumas condutas apresentam estudos sobre seus efeitos em desfechos como mortalidade e eventos CV. Dessa forma, este capítulo e suas recomendações focam os efeitos das MNM sobre a PA, e os efeitos sobre mortalidade são apresentados apenas quando existem evidências específicas. O capítulo tem um objetivo prático, trazendo os resultados da magnitude de efeito de cada MNM e a recomendação de como aplicar cada uma delas. Além disso, em cada intervenção, novas abordagens com evidências promissoras de benefício são mostradas. Para completar, o capítulo aborda o papel fundamental da equipe multidisciplinar para o sucesso das MNM que impactam o controle da HA.

### 6.1. Cessação do Tabagismo

O tabagismo, considerado como o uso de produtos derivados do tabaco, narguilê e cigarros eletrônicos, é um dos mais relevantes fatores de risco CV, de modo que, parar de fumar se associa à redução de mortalidade geral e CV.^137,138^ A relação entre tabagismo e PA é controversa, com alguns estudos não demonstrando associação entre a intensidade do fumo e a PA,^139^ e com outros demonstrando aumento da PA com o início do hábito de fumar^26^ e redução da prevalência de HA nos ex-fumantes de longo prazo.^140^ Considerando a redução do risco CV global, parar de fumar deve ser recomendado por todos os profissionais da saúde para todos os pacientes, e as diversas estratégias e medicamentos disponíveis para auxiliar na adoção dessa conduta devem ser usadas a partir de orientação especializada.^141^

### 6.2. Perda de Peso

A HA está fortemente relacionada ao sobrepeso e à obesidade, visto que 60 a 77% dos obesos têm HA.^142,143^ Mesmo sem alcançar o peso corporal desejável, a perda ponderal reduz a PA e o risco CV proporcionalmente à magnitude e duração da perda de peso.^143,144^ Em uma metanálise, a redução de 1 kg de peso corporal promoveu redução de 1,05 mmHg na PAS e de 0,92 mmHg na PAD.^145^ Em pacientes obesos, a perda de peso se associa à redução de 15% na mortalidade.^143,146^ Assim, para pacientes com HA e que tenham sobrepeso ou obesidade, a perda de peso é uma recomendação essencial. Em adultos, o ideal é alcançar e manter um peso saudável, representado pelo IMC < 25 kg/m^2^.^1,139^ Em idosos, o IMC entre 22 e < 27 kg/m^2^.^1,139^

A avaliação não deve se limitar à análise do IMC, devendo-se incluir parâmetros de adiposidade central, como a circunferência abdominal (CA), que deve ser mantida < 102 cm em homens e < 88 cm em mulheres.^1^ A manutenção do peso corporal depende da adoção de hábitos saudáveis complementados por condutas medicamentosas e cirúrgicas, quando necessárias. Há evidências de que a cirurgia bariátrica associada a MNM seja mais efetiva em diminuir a HA e a DCV aterosclerótica na obesidade grave.^143,144^

### 6.3. Modificações na Ingestão Alimentar

Evidências consistentes indicam que algumas modificações na ingestão alimentar reduzem a PA e devem ser recomendadas aos pacientes com HA (Tabela 6.1). Essas recomendações e suas evidências são apresentadas a seguir.

**Tabela 6.1:** Medidas nutricionais para prevenção e tratamento da hipertensão arterial

Modificação	Meta
Redução na ingestão de sódio	< 2 g/dia = 5 g de sal/dia = uma colher de chá/dia^11^
Aumento na ingestão dietética de potássio	≥ 3,5 g/dia^18^*
Diminuição do consumo de álcool	Limitar a ingestão de bebida alcoólica: Homens – duas doses**/dia Mulheres – uma dose**/dia^14^ Deve-se evitar consumo episódico abusivo de álcool (*binge drinking*)^14^
Adoção de padrão alimentar saudável	Adotar, preferencialmente, a dieta DASH^14,23^

DASH: *Dietary Approaches to Stop Hypertension*.

* Exceto em pacientes com doença renal crônica ou com hiperpotassemia.

** Existem diferentes definições para a quantidade de álcool presente em uma dose^17^, variando em média de 10 a 15 g. Recomendações recentes para a hipertensão arterial consideram que uma dose contém 10 a 12 g de álcool^14^, correspondendo a aproximadamente 200 mL de cerveja (5% de álcool), 100 mL de vinho (12% de álcool) e 25 mL de bebida destilada, como uísque, vodca e aguardente (40% de álcool).

#### 6.3.1. Restrição na Ingestão de Sódio

O consumo elevado de sódio está associado ao aumento da PA, podendo ser responsável por cerca de 30% da prevalência de HA.^147^ A restrição na ingestão de sódio promove redução proporcional da PA, que é mais pronunciada em indivíduos com HA, negros e idosos.^148^ A redução da ingestão de cada 1,15 g/dia de sódio reduz a PAS e a PAD em 2,8 e 1,4 mmHg, respectivamente, em pacientes com HA.^148^ Assim, a restrição da ingestão de sódio para no máximo 2 g/dia é recomendada, o que corresponde a 5 g de sal de cozinha ou 1 colher de chá/dia.^149^

No Brasil e no mundo, a ingestão habitual de sódio é aproximadamente o dobro dessa recomendação.^149^ Alimentos *in natura* contribuem com uma parcela muito pequena dessa ingestão de sódio. Nos países de alta renda, a maior parte do sódio ingerido (70 a 80%) provém dos alimentos industrializados (processados e ultraprocessados) ou consumidos em *fast-food* e restaurantes. Nos países de renda baixa ou média, a principal fonte de sódio é a adição de sal durante a cocção (cozimento) ou à mesa, além do uso de condimentos ricos em sódio. A expansão da indústria de alimentos tem aumentado a exposição desses países aos alimentos processados.^147,149^

Estratégias para a redução da ingestão de sódio incluem a conscientização da população, a reformulação de alimentos industrializados, o uso de temperos, como ervas e pimentas, e a utilização de substitutos do sal nos quais parte do sódio é substituída por potássio.^149,150^ Esses substitutos do sal reduzem a PAS e a PAD em 5,9 e 2,2 mmHg, respectivamente, em pacientes com HA e podem diminuir o risco de eventos cardiovasculares, além da mortalidade CV e por todas as causas.^24^ Entretanto, os substitutos do sal contendo potássio podem elevar esse íon sérico em indivíduos com risco de hipercalemia, como pacientes com DRC.^151^ O sal hipossódico ou *light* pode ser considerado um substituto do sal, pois possui teor de sódio pelo menos 50% menor que o sal de cozinha.^152^ O sal de ervas (mistura do sal de cozinha com ervas secas como alecrim, manjericão, orégano e salsinha) é mais uma opção de sal com menor teor de sódio. Porém, outros tipos de sal, como o sal rosa do Himalaia e o sal marinho, apresentam o mesmo teor de sódio que o sal de cozinha.^153,154^

#### 6.3.2. Aumento no Consumo de Potássio Dietético

O aumento da ingestão de potássio reduz a PAS e a PAD em cerca de 4,8 e 3,0 mmHg, respectivamente, nos pacientes com HA, sendo esse efeito mais acentuado quando a ingestão de sódio é elevada.^150,155^ Apesar da maioria dos estudos ter utilizado cápsulas de cloreto de potássio, a resposta da PA é similar quando a modificação dietética é usada.

Como dietas ricas em potássio refletem um padrão alimentar saudável, elas são a forma recomendada para aumentar a ingestão desse mineral,^156^ que deve ser de pelo menos 3,5 g/dia.^156^ Boas fontes de potássio incluem frutas, verduras, hortaliças, oleaginosas, leguminosas e laticínios pobres em gordura.^151^ Quatro a cinco porções de frutas (uma porção equivale a uma unidade média ou ½ xícara fruta fresca cortada) e hortaliças (uma porção equivale a uma xícara de hortaliças folhosas cruas ou ½ xícara de hortaliças cozidas) fornecem, em geral, de 1,5 a 3,0 g de potássio.^45,157-159^

#### 6.3.3. Redução no Consumo de Álcool

O consumo de álcool tem associação direta com a PA e a incidência de HA, especialmente quando a ingestão é superior a duas doses/dia (10 a 12 g/dia).^150,151,160^ A redução na ingestão de álcool não diminui a PA em indivíduos com ingestão de até duas doses/dia, mas diminui naqueles com ingestão de três ou mais doses/dia, sendo a redução mais acentuada nos que ingerem seis ou mais doses/dia e reduzem essa ingestão em cerca de 50%, obtendo uma diminuição de 5,5 e 4,0 mmHg na PAS e PAD, respectivamente.^161^

Apesar de o consumo de álcool em quantidades pequenas a moderadas já ter sido associado a efeito cardioprotetor, evidências recentes indicam que essa associação pode ser atribuída a fatores de confusão e que, de fato, a ingestão de álcool tem relação com maior risco de DCV, havendo aumento exponencial com níveis mais elevados de ingestão.^25^ Portanto, mesmo em baixas doses, o consumo de álcool pode trazer prejuízos à saúde, e o maior risco está associado com o consumo elevado, sendo ele contínuo ou episódico.^162^ Assim, para indivíduos que consomem bebidas alcoólicas, a redução/moderação nessa ingestão é recomendada, associada à adoção de alguns dias na semana sem a ingestão de álcool para auxiliar no controle da PA.^45,150,161^

#### 6.3.4. Adoção de um Padrão Alimentar Saudável

A avaliação do padrão alimentar, definido como a combinação de todos os alimentos habitualmente consumidos, permite estudar os nutrientes e alimentos de forma conjunta, além do sinergismo entre eles. Entre esses padrões, a dieta DASH (*Dietary Approaches to Stop Hypertension*) enfatiza o consumo de frutas, hortaliças, laticínios com baixo teor de gordura, cereais integrais, carnes magras e frutas oleaginosas, enquanto restringe o consumo de gorduras saturadas, carnes gordurosas, grãos refinados e açúcar de adição. Devido às suas características, é uma dieta rica em potássio, cálcio, magnésio e fibras, contendo quantidades reduzidas de sódio, colesterol, gordura total e saturada.^163^ A dieta DASH reduz a PAS/PAD em 8,7/4,5 mmHg em pacientes com HA,^164^ e sua combinação com a restrição adicional de sódio produz maior redução.^163^

Outro padrão alimentar com efeito na PA é a dieta do Mediterrâneo, que é rica em frutas, hortaliças e cereais integrais, pobre em carne vermelha e que se caracteriza pelo consumo de peixe, oleaginosas e quantidades generosas de azeite de oliva e moderadas de vinho tinto.^165^ Essa dieta promove redução modesta da PAS/PAD de 1,5/0,9 mmHg.^165^ Assim, a adoção de um padrão alimentar saudável, como a dieta DASH ou Mediterrânea, é recomendada na HA. Os efeitos benéficos da dieta DASH e de outras dietas saudáveis dependem da adesão em longo prazo, o que, normalmente, é um desafio.^151,166,167^ As barreiras para essa adesão incluem a falta de motivação e o custo dos alimentos.

#### 6.3.5. Outras Intervenções na Dieta

Outros componentes da alimentação também podem afetar a PA, porém as evidências atuais não são conclusivas/robustas. Alguns desses componentes são abordados a seguir:

- Café e produtos com cafeína: estudos recentes sugerem que o consumo habitual moderado de café não eleva a PA e não aumenta o risco de HA.^168^ Assim, recomenda-se que o consumo de café seja moderado, chegando até três a quatro xícaras/dia.^151^ Quantidades mais elevadas devem ser evitadas devido ao elevado teor de cafeína que é, em média, de 80 mg/200 mL de café filtrado. A cafeína promove elevação aguda da PA, em especial, nos indivíduos que não têm o hábito de ingeri-la, sendo considerada segura a ingestão de até 400 mg/dia em adultos saudáveis.^169^ Assim, o consumo de outras bebidas ricas em cafeína, como os energéticos, também deve ser controlado;
- Probióticos: nos últimos anos, surgiram evidências de que a suplementação de probióticos pode produzir redução modesta da PA em pacientes com HA;^170^
- Suplementação de vitamina D: apesar de não haver consenso, a suplementação de vitamina D parece reduzir a PA especificamente em pacientes com HA que tenham hipovitaminose D.^171^

### 6.4. Prática de Atividade Física e Exercício Físico

AF refere-se a qualquer movimento corporal que aumente o gasto energético acima do repouso, como locomoção, atividades laborais, domésticas e de lazer. O exercício físico (EF), por sua vez, refere-se à AF estruturada, realizada com um objetivo específico, como melhorar a saúde e/ou a aptidão física. O comportamento sedentário é o tempo gasto em atividades de baixo gasto energético (≤ 1,5 MET [*metabolic equivalente of task*]), como aquelas realizadas na posição sentada, reclinada ou deitada (por exemplo, assistir à TV, usar o computador, jogar *videogame* etc.).^172^

Estima-se que cada hora passada em comportamento sedentário aumenta em 5% o risco CV. Assim, recomenda-se a interrupção do tempo em comportamento sedentário com a execução de AFs leves.^173^

A prática regular de AF diminui a PAS entre 2 e 5 mmHg e a PAD entre 1 e 4 mmHg, com essa redução sendo maior quanto maior for o nível inicial da PA.^174^ A prática de AF, principalmente de lazer, reduz em até 19% a incidência de HA e, nos pacientes com HA, diminui em cerca de 30% o risco de mortalidade.^174,175^ Assim, a prática regular de AF é recomendada para a prevenção e tratamento da HA.^173,174^

Em pacientes com HA, os maiores benefícios são obtidos com o EF estruturado. Tanto o treinamento aeróbico quanto o resistido dinâmico, o resistido isométrico e o combinado (aeróbico + resistido dinâmico) reduzem a PA de consultório (Tabela 6.2).^176^ Porém, apenas o treinamento aeróbico reduz a PA ambulatorial.^177^ Assim, na HA, recomenda-se o treinamento aeróbico, que pode ser associado ao resistido dinâmico para se obterem benefícios complementares à saúde.

**Tabela 6.2:** Redução da pressão arterial sistólica e da pressão arterial diastólica com os diferentes tipos de treinamento

Treinamento	PAS/PAD de consultório (mmHg)^33^	PAS/PAD de 24 horas (mmHg)^34^
Aeróbico	-7,6/-4,7	-5,5/-3,8
Resistido dinâmico	-2,6/-2,1	---
Resistido isométrico	-4,3/-5,0	---
Combinado	-5,3/-5,6	---

PAD: pressão arterial diastólica; PAS: pressão arterial sistólica. --- sem efeito comprovado.

Outras modalidades de EF, como as realizadas em meio líquido, as práticas alternativas neuromotoras (ioga, *tai chi chuan*, pilates etc.) e o treinamento intervalado de alta intensidade, reduzem a PA de consultório em pacientes com HA, mas os poucos estudos que embasam esses efeitos têm qualidade limitada, não havendo evidências de redução da PA ambulatorial nem avaliação dos potenciais riscos.^174^ Assim, essas práticas não são recomendadas como tratamento da HA, mas podem ser usadas em situações pontuais.

A Tabela 6.3 apresenta as recomendações de AF e EF para pacientes com HA. Alguns cuidados devem ser tomados antes e durante essa prática.^172,178^ Para a prescrição de prática de AFs leves a moderadas em indivíduos sem doença cardíaca, renal ou cerebrovascular, a avaliação médica prévia não é obrigatória. Caso surjam sintomas durante a prática, deve-se interromper a atividade e procurar o médico. Indivíduos com HA que tenham comorbidades, sintomas cardiovasculares (angina, cansaço, dispneia, tontura, edema etc.), múltiplos fatores de risco ou que pretendem fazer atividades de alta intensidade ou competitivas devem se submeter à avaliação médica prévia. Recomenda-se o teste ergométrico para avaliar a aptidão física e prescrever o treinamento, pois ele possibilita avaliar a resposta da PA ao esforço e suspeitar da presença de DAC nos indivíduos sintomáticos ou com múltiplos fatores de risco. A sessão de treinamento não deve ser realizada se a PA estiver acima de 160/105 mmHg, e a intensidade deve ser reduzida se, durante o exercício aeróbico, a PA estiver acima de 180/105 mmHg.^172,179-182^

**Tabela 6.3:** Orientações para a prescrição de atividade física e exercício físico

**Interrupção do comportamento sedentário**		
– Levantar e se movimentar por 5 min a cada 30 min sentado.		
**Atividade física**		
– Realizar, pelo menos, 150 min/semana de AF aeróbica moderada ou 75 min/semana de AF aeróbica vigorosa, ou ainda, uma combinação equivalente de AF moderada e vigorosa.		
– Para maior benefício, executar exercícios de fortalecimento muscular duas vezes/semana.		
**Treinamento físico**		
– Aeróbico complementado pelo resistido.		
**Prescrição do treinamento aeróbico – Obrigatório**		
– Modalidades diversas: andar, correr, dançar, nadar, entre outras.		
– Volume: somar, pelo menos, 150 min/semana.		
	– Frequência: três a cinco vezes por semana (mais vezes – maior efeito);
	– Duração: 30 a 60 min por sessão (mais tempo – maior efeito).
– Intensidade moderada definida por:		
	1) Maior intensidade conseguindo conversar (sem ficar ofegante);
	2) Sentir-se "ligeiramente cansado" (12 a 13 na escala de Borg 20);
	3) Manter a FC de treino na faixa calculada por:	
		FC treino = (FC máxima – FC repouso) x % + FC repouso
Em que: FC máxima: deve ser obtida num teste ergométrico máximo, feito em uso dos medicamentos regulares, ou pelo cálculo da FC máxima prevista pela idade (ou seja, 220 – idade). Essa fórmula não pode ser usada em paciente com HA que tenham cardiopatias, estejam em uso de BB ou de bloqueadores de canais de cálcio não di-hidropiridínicos; FC repouso: deve ser medida após 5 min de repouso deitado; %: utilizar 40% como limite inferior e 60% como superior.		
**Prescrição do treinamento resistido – Complementar**		
– Frequência: duas a três vezes/semana		
– 8 a 10 exercícios para os principais grupos musculares, priorizando a execução unilateral, quando possível		
– Uma a três séries		
– 10 a 15 repetições até a fadiga moderada (ou seja, repetição na qual há redução da velocidade de movimento) – aproximadamente 60% de 1RM		
– Pausas longas passivas: 90 a 120 s		

1RM: repetição máxima; AF: atividade física; FC: frequência cardíaca; HA: hipertensão arterial; BB: betabloqueadores. Adaptado das referências.^1,172,173,178^

### 6.5. Abordagem de Aspectos Psicoemocionais

#### 6.5.1. Técnicas de Abordagem do Estresse

O estresse mental está associado a um risco aumentado de HA e possui maior incidência em pacientes com HA do que em normotensos.^18^ Intervenções usuais para a redução do estresse podem diminuir a PAS e a PAD, porém apresentam menor certeza de evidência do que as demais intervenções de mudanças de estilo de vida.^164^ De modo geral, as terapias comportamentais de abordagem do estresse não apresentam evidências robustas de eficácia na diminuição da PA, exceto a meditação.^164^ Nesse sentido, uma metanálise demonstrou que programas de redução de estresse baseados em *mindfulness*, que consistiam em pelo menos oito sessões de meditação intensiva, reduziram a PAS e a PAD em 6,6/2,5 mmHg, respectivamente.^183^ Além disso, a meditação transcendental reduziu em 5,6 mmHg a PAS e em 2,9 mmHg na PAD, enquanto outras técnicas de meditação reduziram a PAS em 5,1 mmHg e a PAD em 2,6 mmHg.^184-186^

#### 6.5.2. Respiração Lenta

A respiração lenta ou guiada se refere à redução da frequência respiratória para 6 a 10 respirações/min durante 15 a 20 min/dia. Em comparação com a respiração natural, a respiração lenta promove redução de 6,4 mmHg na PAS e na PAD.^187^ Em pacientes com HA e DCV, exercícios voluntários de respiração lenta também reduzem a PAS e a PAD.^187^

Considerando-se a respiração lenta guiada por equipamento (*device guided breathing; Resperate device®*), embora uma metanálise tenha evidenciado redução da PA, esse efeito desapareceu quando os estudos relacionados à indústria de equipamentos foram excluídos.^188^ Assim, recomenda-se a respiração lenta como terapia coadjuvante de baixo risco para a HA.

#### 6.5.3. Espiritualidade e Religiosidade

Espiritualidade é definida como um conjunto de valores morais, mentais e emocionais que norteiam pensamentos, comportamentos e atitudes nas circunstâncias da vida de relacionamento intra e interpessoal, sendo passível de mensuração. Assim, ela difere de religiosidade, que pode ser conceituada como o quanto um indivíduo acredita, segue e pratica uma religião, definida, por sua vez, como um construto multidimensional que inclui crenças, comportamentos, dogmas, rituais e cerimônias, que podem ser realizados ou praticados em contextos privados ou públicos.^189^

O bem-estar espiritual e o cultivo de sentimentos edificantes podem ter efeitos benéficos na saúde CV, com redução do risco CV e menor prevalência de HA.^190^ Recentemente, o estudo FEEL demonstrou redução de 7,6 mmHg da PAS com o estímulo dos sentimentos de perdão, gratidão, otimismo e propósito de vida.^191^ Recomenda-se o treinamento dos profissionais de saúde para a abordagem de aspectos de espiritualidade e religiosidade, com respeito e sem interferências nas crenças do paciente.^192^

### 6.6. Equipe Multiprofissional na Adoção das Medidas Não Medicamentosas

Estudos evidenciam melhor controle da PA com a abordagem multiprofissional, promovendo maior controle dos fatores de risco CV e redução de morbidade e mortalidade CV.^193^ Alguns estudos descritivos mostram que cuidados dispensados por equipes e decisões compartilhadas entre profissionais com a coparticipação do paciente estão associados à redução de custos e ao melhor resultado na HA.^194^ A atuação multiprofissional foi empregada com sucesso em alguns serviços de atenção primária, secundária e terciária, tendo resultado numa taxa de controle da HA de 68%.^195^ Assim, o atendimento multiprofissional amplo é recomendado na prevenção e tratamento da HA.

A equipe deve incluir profissionais de diversas formações (médicos da atenção primária à saúde, cardiologistas, endocrinologistas, nefrologistas, geriatras, enfermeiros, farmacêuticos, nutricionistas, profissionais de educação física, psicólogos, assistentes sociais, profissionais da saúde comunitária, entre outros), pois eles se complementam, integrando competências, habilidades e conhecimentos, dividindo responsabilidades e compartilhando decisões.^196^ O foco deve ser no paciente e deve-se permitir o protagonismo dos diversos atores, desenvolvendo um campo de pesquisa em serviço e proporcionando o crescimento profissional. Ações educativas e terapêuticas devem ser estendidas a todos os pacientes, seus familiares e à comunidade, considerando-se, sempre, as particularidades socioculturais, locais e regionais. Estratégias modernas envolvendo o uso de mídias sociais, técnicas de educação à distância e recursos digitais educacionais podem facilitar a adoção e adesão às MNM de tratamento da HA.^197^

### 6.7. Considerações Finais sobre as Medidas não Medicamentosas

Diversas condutas com diferentes graus de evidência e recomendação constituem MNM de prevenção e tratamento da HA. A Figura 6.1 resume as principais recomendações que, além de efeito na HA, trazem benefícios à saúde em geral. Assim, exceto nas situações especificadas nos quadros, elas devem ser recomendadas por todos os profissionais de saúde para todos os adultos com PA ≥ 120/80 mmHg e, em especial, para os pacientes com HA.

**Figura 6.1:**
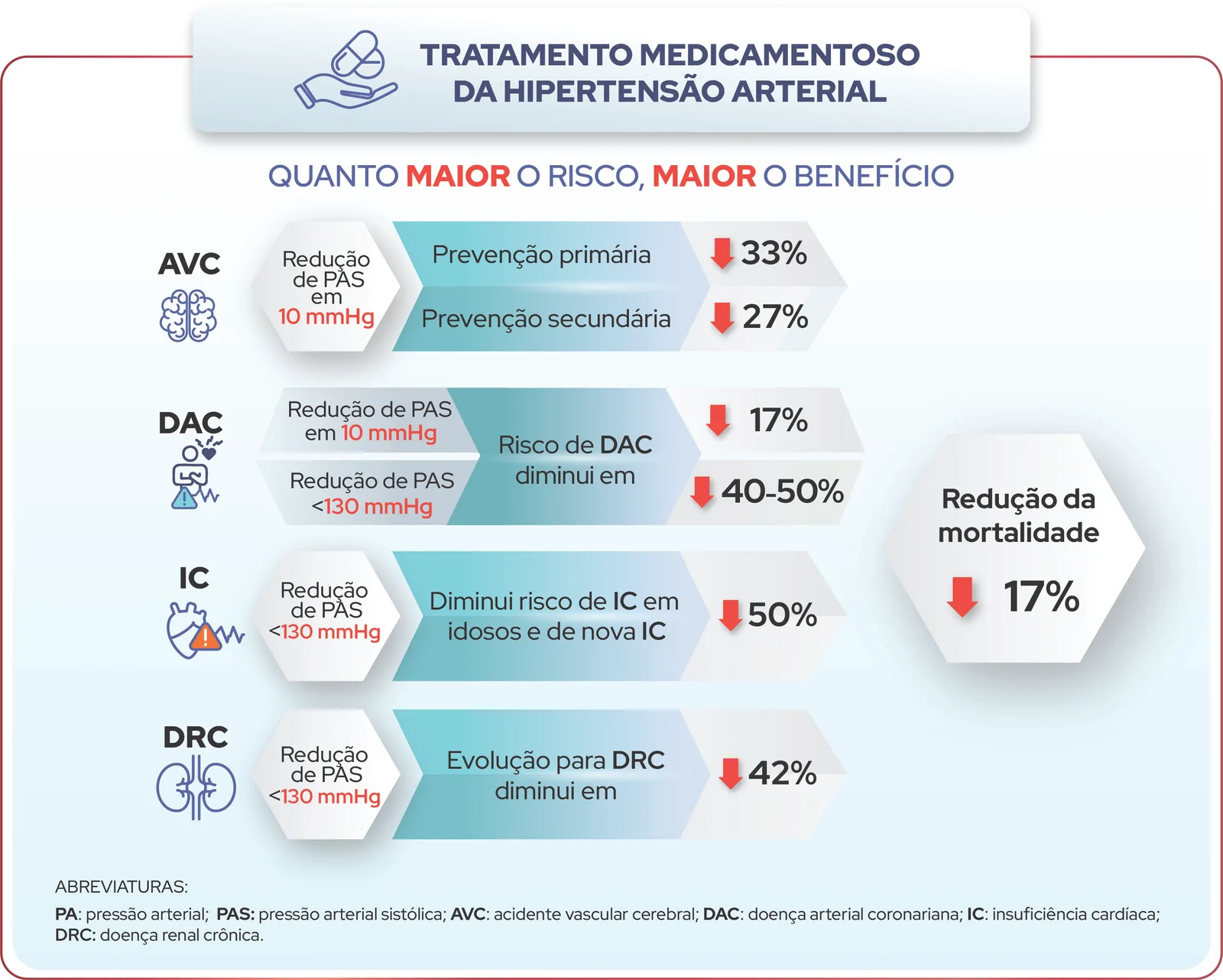
Medidas não medicamentosas para a prevenção e o tratamento da hipertensão arterial

**Table:** 

Recomendações para medidas não medicamentosas de abordagem da PA	Força da recomendação	Certeza da evidência
Recomenda-se a cessação do tabagismo para a redução de eventos cardiovasculares e morte.	FORTE	ALTA
Recomenda-se a diminuição do peso corporal para a redução da PA e de mortalidade em pacientes com obesidade.	FORTE	ALTA
Recomenda-se a redução da ingestão de sódio e aumento da ingestão de potássio (exceto para pacientes com DRC) dietético para a redução da PA.	FORTE	ALTA
Recomenda-se limitação do consumo máximo diário de álcool para a redução da PA.	FORTE	MODERADA
Recomendam-se a dieta DASH e a prática regular de AF moderada para a redução da PA e mortalidade.	FORTE	ALTA
Recomenda-se treinamento aeróbico para a redução da PA.	FORTE	ALTA
Recomenda-se meditação para a redução da PA.	FORTE	MODERADA
Recomendação a favor da prática de respiração lenta para a redução de PA.	NEUTRA	BAIXA
Recomenda-se a abordagem de aspectos de espiritualidade e religiosidade no tratamento da HA.	NEUTRA	BAIXA
Recomenda-se a atuação de equipe multiprofissional para se atingir um melhor controle da PA.	FORTE	BAIXA

DASH: Dietary Approaches to Stop Hypertension; DRC: doença renal crônica; HA: hipertensão arterial; PA: pressão arterial; AF: atividade física.

## 7. Tratamento Medicamentoso

### 7.1. Objetivos do Tratamento e Racional para o Uso de Medicamentos

A diminuição da morbimortalidade é o objetivo primordial do tratamento anti-hipertensivo. A redução da PA é a primeira meta, com o objetivo maior de reduzir desfechos CV, renais, e mortalidade associados à HA, independentemente da idade do indivíduo.^114,198-200^

Uma evidência de alta qualidade, obtida por meio do projeto colaborativo dos Trialists’, analisou os dados individuais de cerca de 350.000 participantes de 48 ECRs, cujo objetivo era avaliar os desfechos CV com a redução da PA em resposta ao tratamento anti-hipertensivo. Durante média de acompanhamento de 4,2 anos, uma redução de 5 mmHg na PAS reduziu o RR de eventos CV maiores em 10%. Os riscos de AVC, IC, DAC e morte por DCV foram reduzidos em 13, 13, 8 e 5%, respectivamente. Observou-se que os benefícios foram maiores quanto maior era o risco CV e maior a redução da PA.^114^

#### 7.1.1. Insuficiência Cardíaca

Com o tratamento da HA, o risco de desenvolver IC é reduzido em 30% nas populações mais jovens, em 50% nos idosos e em quase 80% entre os idosos com história de IAM. Além disso, o controle da PA diminui o risco de nova IC em aproximadamente 50%.^201,202^

#### 7.1.2. Acidente Vascular Cerebral

A terapia anti-hipertensiva reduz significativamente o risco de AVC, tanto em prevenção primária quanto secundária. Um estudo de metanálise demonstrou que a redução da PAS em 10 mmHg está associada a uma redução de 33% no risco de AVC.^203^ Além disso, a análise do estudo HOPE-3 mostrou que a combinação de terapia anti-hipertensiva com estatinas reduziu significativamente o risco de acidente vascular cerebral isquêmico (AVCI) em 59% em pessoas com risco CV intermediário.^204^ Isso também ocorre em prevenção secundária. Uma metanálise de ECRs mostrou que o tratamento anti-hipertensivo reduziu o risco de AVC recorrente em 27%.^205^

#### 7.1.3. Doença Arterial Coronariana

A redução da PAS em 10 mmHg está associada a uma diminuição de 17% no risco de DAC.^120^ Uma redução da PA em pacientes com HA à meta pode levar a uma diminuição de 40 a 50% no risco de morte por DAC em pessoas de meia-idade. No entanto, é importante notar que a eficácia do tratamento pode variar dependendo de fatores como a presença de comorbidades e as classes de medicamentos utilizadas.^120,206^

#### 7.1.4. Doença Renal Crônica

De acordo com o estudo FROM-J (*Frontier of Renal Outcome Modifications in Japan*), uma meta de PAS < 130 mmHg está associada à redução de 42% no risco de desfechos renais em pacientes que inicialmente apresentavam PAS ≥ 130 mmHg.^207^ Além disso, o estudo ARIC (*Atherosclerosis Risk in Communities*) sugere que uma redução modesta na PAS em toda a população pode prevenir novos casos de DRC, com uma redução estimada de 11,7 a 13,4 eventos de DRC por 100.000 pessoas-ano para cada diminuição de 1 mmHg na PAS.^208^

#### 7.1.5. Morte Cardiovascular

A metanálise de Ettehad *et al*. demonstrou que uma redução de 10 mmHg na PAS resulta em uma redução de 13% na mortalidade por todas as causas, o que inclui a morte CV.^120^

Um resumo desses benefícios do tratamento medicamentoso da HA pode ser visualizado na Figura 7.1.^114,120,198-208^

**Figura 7.1:**
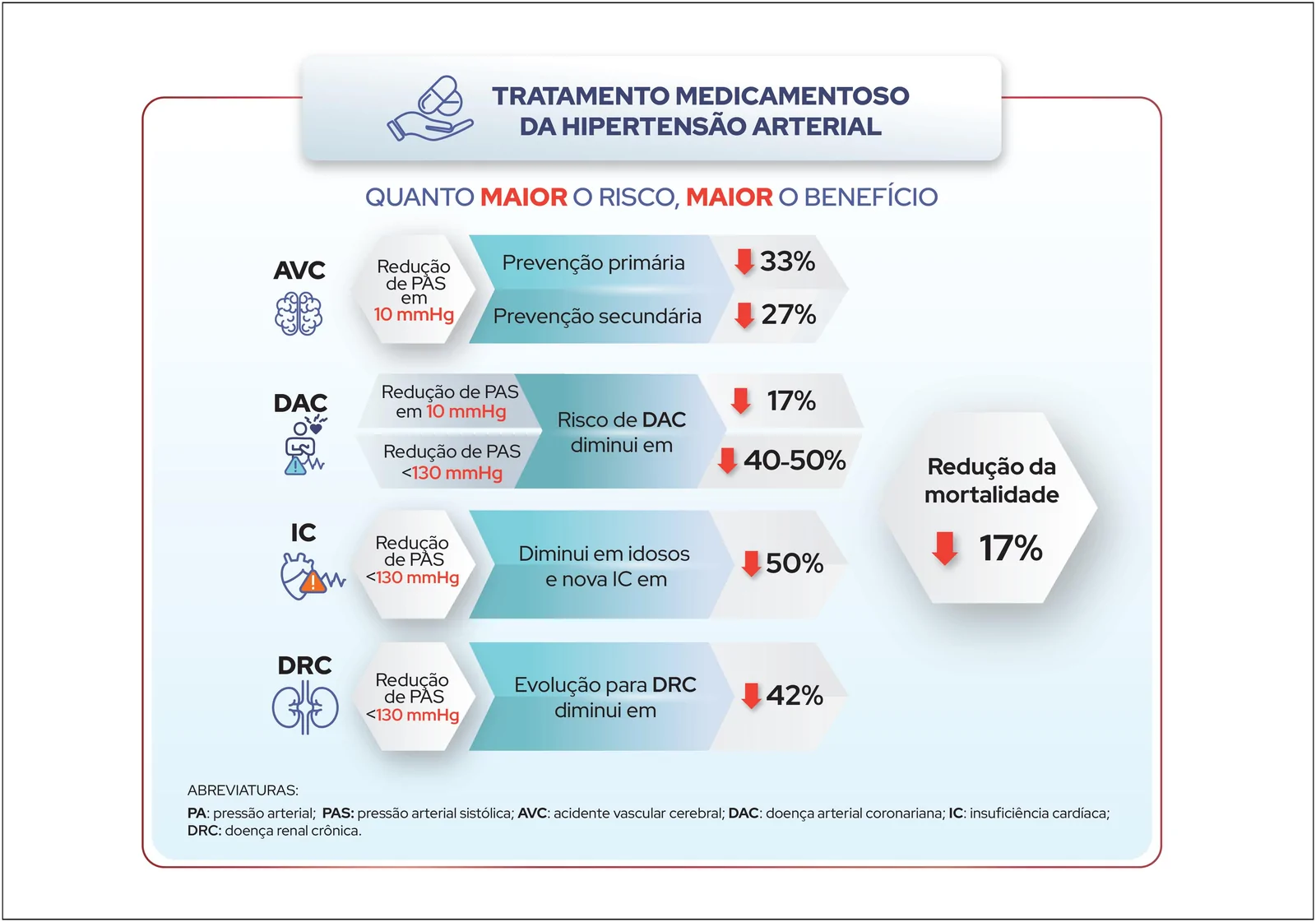
Benefícios do tratamento medicamentoso da hipertensão arterial.^114,120,198-208^

### 7.2. Tratamento Medicamentoso

O tratamento medicamentoso da HA é baseado em diretrizes que recomendam o uso de classes específicas de medicamentos, com base principalmente em evidências robustas de ECR e controlados que demonstraram a eficácia na redução da PA e na diminuição do risco de eventos CV e renais.^1,117,209^

As classes de medicamentos mais recomendadas incluem diuréticos (DIUs) tiazídicos ou tiazídicos similares, inibidores da enzima conversora da angiotensina (IECAs), bloqueadores dos receptores da angiotensina II (BRAs) e bloqueadores dos canais de cálcio (BCCs). Há controvérsias sobre se os betabloqueadores (BBs) poderiam ser incluídos na lista de medicamentos preferenciais^45,117,150,210^ e, por esse motivo, foi elaborada uma RS com metanálise especificamente para essa diretriz, visando encontrar uma resposta baseada em evidências robustas, a qual será discutida neste capítulo. Por outro lado, o uso dos BBs está consagrado quando há condições clínicas associadas como: síndromes coronarianas (pós-IAM e angina);^211^ em fibrilação atrial (FA); insuficiência cardíacada com fração de ejeção reduzida (ICFEr); para o controle da frequência cardícada (FC); em mulheres com chance ou planejando engravidar,^212^ e na DRC em hemodiálise.^213^

Há grandes disparidades globais entre países de alta e baixa renda no que se refere ao diagnóstico, tratamento e controle da HA, apesar dos reconhecidos benefícios sobre a morbidade e mortalidade CV e renal, ou seja, há muito o que se planejar e implementar para melhores desfechos.^214^

### 7.3. Princípios Gerais do Tratamento Medicamentoso

A maioria dos pacientes com HA necessitará, além de MNM, de medicamentos em associação para alcançar a meta de PA recomendada (ver Capítulo 5).

O Quadro 7.1 mostra as cinco principais classes de medicamentos anti-hipertensivos que demonstraram reduções da PA comparadas com placebo, acompanhadas de benefícios consideráveis sobre desfechos CV e renais fatais ou não, destacando-se que esses benefícios estiveram relacionados fundamentalmente à redução da PA.^215-219^

**Quadro 7.1:** Principais classes de medicamentos anti-hipertensivos utilizados no tratamento da hipertensão arterial^215-219^

Diuréticos (DIUs)
Bloqueadores dos canais de cálcio (BCCs)
Inibidores da enzima conversora da angiotensina (IECAs)
Bloqueadores dos receptores AT1 da angiotensina II (BRAs)
Betabloqueadores (BBs), com indicações específicas

Informações específicas sobre cada classe de medicamentos serão abordadas nos Quadros 7.2 a 7.6.

**Quadro 7.2:** Diuréticos, suas doses diárias habituais (mg/dia), frequência de tomadas (número de doses/dia) e informações adicionais

DIURÉTICOS
Inibem a reabsorção de sódio em diferentes locais do néfron, determinando natriurese. No longo prazo, diminuem a resistência periférica. Podem ser associados sinergicamente com outros anti-hipertensivos.

Classe e medicamento	Dose diária habitual (mg)	Frequência (nº de doses/dia)	Informações adicionais
**DIURÉTICOS TIAZÍDICOS E SÍMILES**			
Hidroclorotiazida	– 50	1	– Ação no túbulo contornado distal. – Doses maiores aumentam o efeito diurético, mas também os efeitos colaterais metabólicos, sem acrescentar efeito anti-hipertensivo. – Contraindicação em gota ativa.
Clortalidona	12,5 – 25	1	
Indapamida (*slow release*)	1,5 – 3,0	1	
**DIURÉTICOS DE ALÇA**			
Furosemida	20 – 240	1 a 3	– Ação no ramo ascendente espesso da alça de Henle. – Utilizada na DRC, IC e estados edematosos (retenção de sódio e água).
**DIURÉTICOS POUPADORES DE POTÁSSIO**			
Espironolactona	25 – 100	1 a 2	– Antagonista dos receptores mineralocorticoides –hormonal não seletivo. – Uso em IC, *status* de hiperaldosteronismo, HA resistente e refratária (4º medicamento), HA com comorbidades, diminui proteinúria. – Pode provocar hiperpotassemia, particularmente na DRC e associado à IECA ou BRA. – Efeitos colaterais: ginecomastia, diminuição da libido, impotência.
Eplerenona	25 – 100	1	– Antagonista dos receptores mineralocorticoides – não hormonal seletivo. – Mais seletiva para receptores de aldosterona (↓ efeitos adversos). – Hiperpotassemia similar à espironolactona. – Mesmas indicações que a espironolactona.
Amilorida	2,5 – 5	1	– Ação nos canais de sódio do túbulo distal e coletor. – Disponível em associação com diurético tiazídico ou similar.

BRA: bloqueadores dos receptores AT1 da angiotensina II; DRC: doença renal crônica; HA: hipertensão arterial; IECA: Inibidores da enzima conversora da angiotensina; IC: insuficiência cardíaca.

**Quadro 7.3:** Bloqueadores dos canais de cálcio, suas doses diárias habituais (mg/dia), frequência de tomadas (número de doses/dia) e informações adicionais

BLOQUEADORES DE CANAIS DE CÁLCIO (BCC)
Bloqueiam os canais lentos de cálcio, diminuindo o influxo de cálcio nas células musculares lisas vasculares e a resistência vascular. Todos têm demonstrado reduções similares da pressão arterial em doses equipotentes. Podem ser associados sinergicamente com outros anti-hipertensivos.

Classe e medicamento	Dose diária habitual (mg)	Frequência (nº de doses/dia)	Informações adicionais
**Bloqueadores de canais de cálcio di-hidropiridínicos**			
Anlodipino	2,5 – 10	1	– Evitar uso em pacientes com ICFEr e em taquiarritmias. – Pode provocar cefaleia, rubor facial, edema de membros inferiores. – Edema dose-dependente que melhora com uso de bloqueadores do SRAA e não responde bem a diuréticos.
Felodipino	2,5 – 10	1	
Nifedipino	10 – 60	1 – 3	
Nitrendipino	10 – 30	1	
Manidipino	10 – 20	1	
Lacidipino	2 – 6	1	
Lercanidipino	10 – 20	1	
Levanlodipino	2,5 – 5	1	
**Bloqueadores de canais de cálcio não di-hidropiridínicos** Pouco utilizados no tratamento da hipertensão arterial atualmente			
Fenilalquilamina (Verapamil)	120 – 360	1 a 2	– Evitar uso em pacientes com ICFEr. – Evitar associação com BBs ou pacientes com bradicardia (FC < 50 bpm).
Benzotiazepina (Diltiazem)	80 – 240	1 a 2	– Mesmo cuidado com bradicardia e associação com BBs.

*bpm: batimentos por minuto; FC: frequência cardíaca; ICFEr: insuficiência cardíaca com fração de ejeção reduzida; SRAA: sistema renina angiotensina aldosterona; BBs=: betabloqueador; BCC: bloqueadores de canais de cálcio.*

**Quadro 7.4:** Inibidores da enzima conversora da angiotensina, suas doses diárias habituais (mg/dia), frequência de tomadas (número de doses/dia) e informações adicionais

Classe e medicamento	Dose diária habitual (mg)	Frequência (nº de doses/dia)	Informações adicionais e recomendações
**Inibidores da enzima conversora da angiotensina II**			
Bloqueiam a ECA, reduzindo a produção de angiotensina II e a degradação da bradicinina; têm propriedades anti-ateroscleróticas e anti-inflamatórias. Eficazes na ICFEr e pós-IAM; diminuem progressão da DRC, proteinúria e HVE. Associam-se sinergicamente a outros anti-hipertensivos.			
Captopril	25 – 150	2 a 3	– Geralmente, bem tolerados. – Evitar uso em mulheres em idade fértil e contraindicado na gravidez. – Contraindicação em associação com BRA. – Pode ser usado com espironolactona na IC e DRC com proteinúria. – Contraindicado na estenose de artéria renal bilateral ou em rim único. – Risco de hiperpotassemia (especialmente em DRC avançada ou uso de potássio/espironolactona). – Pode piorar a função renal no início, com estabilização posterior. – Tosse é efeito colateral comum (por acúmulo de bradicinina).
Enalapril	5 – 40	1 a 2	
Benazepril	10 – 40	1 a 2	
Lisinopril	10 – 40	1	
Fosinopril	10 – 40	1	
Ramipril	2,5 – 20	1 a 2	
Perindopril	4 – 16	1	

BRA: bloqueadores dos receptores AT1 da angiotensina II; DRC: doença renal crônica; ECA: enzima conversora da angiotensina; HVE: hipertrofia ventricular esquerda; IAM: infarto agudo do miocárdio; ICFEr: insuficiência cardíaca com fração de ejeção reduzida; IC: insuficiência cardíaca; SRAA: sistema renina angiotensina aldosterona.

**Quadro 7.5:** Bloqueadores dos receptores AT1 da angiotensina II, suas doses diárias habituais (mg/dia), frequência de tomadas (número de doses/dia) e informações adicionais

**Bloqueadores dos receptores AT**1 **da angiotensina II** Impedem ação da angiotensina II por bloqueio do receptor AT1; propriedades anti-ateroscleróticas e anti-inflamatórias. Eficazes na ICFEr, pós-IAM; reduzem progressão de DRC, proteinúria e HVE. Associam-se sinergicamente a outros anti-hipertensivos.			
Medicamento	Dose diária habitual (mg)	Frequência (nº de doses/dia)	Informações adicionais e recomendações
Losartana	50 – 100	1 a 2	Mesmas informações, recomendações e efeitos colaterais dos IECAs, com exceção de tosse
Valsartana	80 – 320	1	
Irbesartana	150 – 300	1	
Candesartana	8 – 32	1	
Olmesartana	20 – 40	1	
Telmisartana	20 – 80	1	

DRC: doença renal crônica; HVE: hipertrofia ventricular esquerda; IAM: infarto agudo do miocárdio; ICFEr: insuficiência cardíaca com fração de ejeção reduzida; IECAs: inibidores da enzima conversora da angiotensina.

**Quadro 7.6:** Betabloqueadores, suas doses diárias habituais (mg/dia), frequência de tomadas (número de doses/dia) e informações adicionais

Classe e Medicamento	Dose diária habitual (mg)	Frequência (número de doses/dia)	Informações adicionais e recomendações
**Betabloqueadores** Indicações: ICFEr, Síndromes coronarianas agudas, no pós-IAM e em pacientes com angina, e fibrilação atrial. Hipertensão associada a arritmias, tremor essencial, prolapso de válvula mitral, enxaqueca, ansiedade, hipertensão portal, síndromes hipercinéticas como tireotoxicose, apneia obstrutiva do sono. Contraindicações: broncopatia grave ou descompensada (se indispensável, usar os cardiosseletivos ou com ação vasodilatadora, com cautela), bloqueio sinoatrial ou atrioventricular, bradicardia (FC < 50 bpm), doença arterial obstrutiva periférica (exceto para os betabloqueadores com ação vasodilatadora).			
**1ª GERAÇÃO E NÃO CARDIOSSELETIVO**			
Propranolol	80 – 320	2 a 3	– Bloqueiam os receptores β1 (principalmente no miocárdio) e β2 (músculo liso, pulmões, vasos sanguíneos etc.). – Efeitos periféricos mais acentuados, como aumento da resistência arterial periférica e broncoconstrição. – Pindolol possui atividade simpatomimética intrínseca que proporciona menor bradicardia.
Nadolol	40 – 160	1	
Pindolol	10 – 60	1	
**2ª GERAÇÃO E CARDIOSSELETIVO**			
Atenolol	50 – 100	1 a 2	– Bloqueiam apenas os receptores β1 adrenérgicos, presentes em maior parte no coração, no sistema nervoso e nos rins e, portanto, sem os efeitos de bloqueio periférico indesejáveis. – Doses muito altas podem ter ação nos receptores β2.
Metoprolol	50 – 200	1	
Bisoprolol	5 – 20	1	
**3ª GERAÇÃO**			
Nebivolol	2,5 – 10	1	– Cardiosseletivo. – Ação vasodilatadora por maior disponibilidade do óxido nítrico.
Carvedilol	6,25 – 50	1 a 2	– Não cardiosseletivo. – Efeito alfa e betabloqueador, produz menor bradicardia e vasodilatação. Indicado na ICFEr.

bpm: batimentos por minuto; FC: frequência cardíaca; IAM: infarto agudo do miocárdio; ICFEr: insuficiência cardíaca com fração de ejeção reduzida.

Outras classes de medicamentos, como antagonistas dos receptores mineralocorticoides (ARMs) não seletivos, alfabloqueadores, simpatolíticos de ação central e periférica e vasodilatadores de ação direta, não foram amplamente estudadas em ECR e se associam a maior taxa de eventos adversos, devendo ser utilizadas quando não houver controle da PA em pacientes que já estão em uso de associações de medicamentos com as principais classes já mencionadas (Quadro 7.1).

As características desejáveis em um medicamento anti-hipertensivo são: ser capaz de reduzir a morbidade e a mortalidade CV e renal; demonstrar eficácia por via oral e boa tolerabilidade; ter ação prolongada, possibilitando sua utilização em dose única diária; apresentar sinergismo farmacológico com outras classes medicamentosas, permitindo seu uso em associação; passar por controle de qualidade em sua produção, não devendo ser utilizados medicamentos manipulados.^1^

Além de se considerarem as características dos medicamentos, é importante:

- Fazer os ajustes de tratamento geralmente após 4 semanas de uso, salvo em situações específicas;
- Orientar pacientes e eventuais cuidadores que, para o sucesso do controle da PA, é absolutamente necessária a adesão ao tratamento;
- Considerar que podem ser precisos ajustes de doses, trocas ou associações de outros medicamentos;
- Alertar sobre o eventual aparecimento de efeitos adversos e como lidar com eles.

Os medicamentos anti-hipertensivos em monoterapia ou em associação podem ser administrados pela manhã ou à noite, com eficácia similar.^220^

### 7.4. Classes dos Medicamentos Utilizados no Tratamento da Hipertensão Arterial

A Figura 7.2 resume o octeto do tratamento da HA, de tal modo que as classes preferenciais estão na base da pirâmide, e os medicamentos menos utilizados e com menores evidências disponíveis na literatura estão no topo.

**Figura 7.2:**
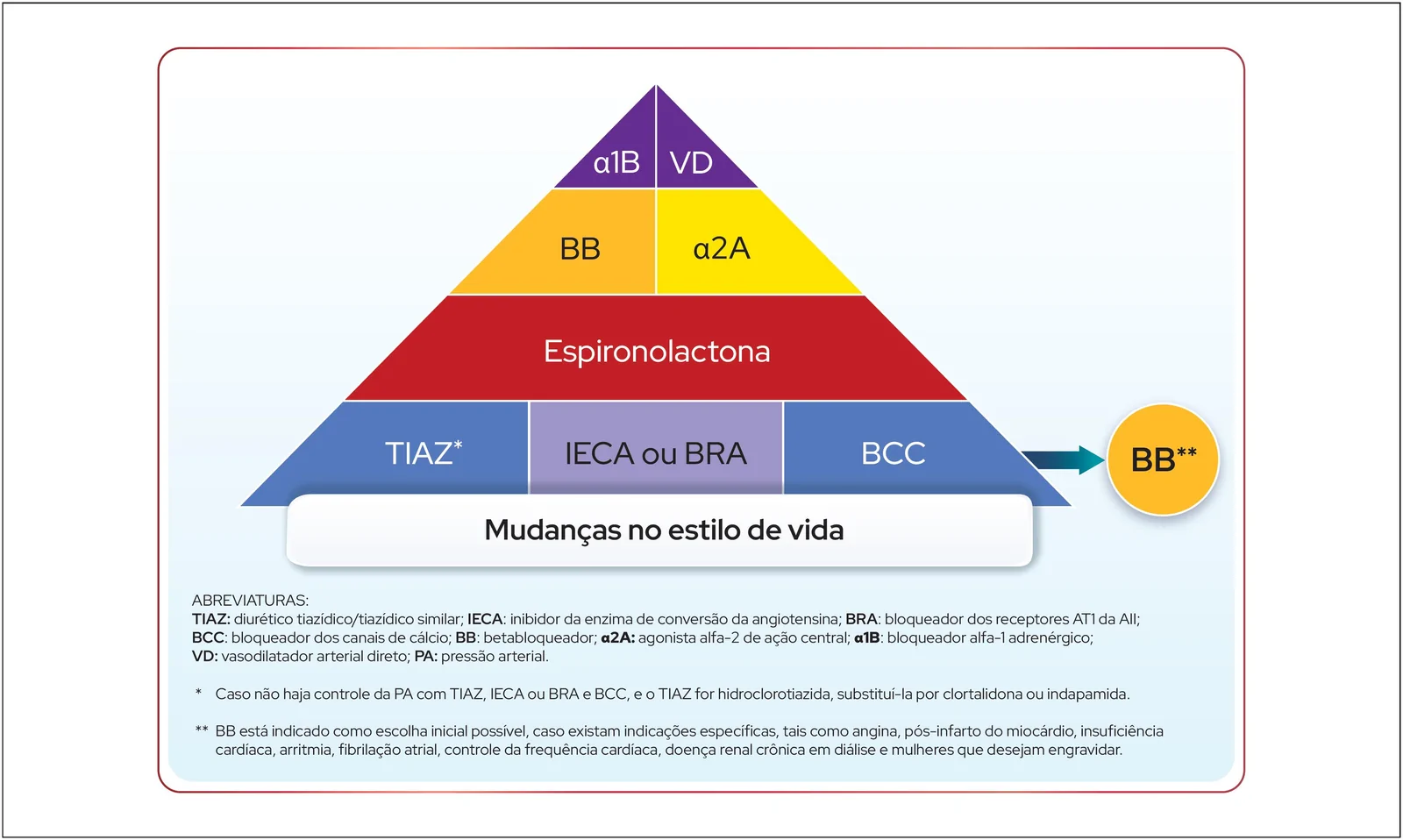
Octeto do tratamento da hipertensão arterial. Adaptada de Feitosa et al.^221^

A inclusão dos BBs na base do octeto do tratamento da HA, com indicações específicas, foi baseada em RS com metanálise desenvolvida pela SBC, visando sanar as controvérsias existentes entre diretrizes sobre sua recomendação.^117,219,221^

Foram incluídos sete ECRs que preencheram os critérios de inclusão, com o objetivo de avaliar a eficácia e segurança do atenolol no tratamento de HA primária em comparação a outros medicamentos anti-hipertensivos sabidamente eficazes, modificadores de desfechos e bem tolerados.^211^

A pergunta PICO (paciente/problema, intervenção, comparador, desfecho – *patient/problem, intervention, comparison, outcome*) limitou-se ao atenolol, porque é o único entre os BBs que tem ECRs que pudessem ser incluídos na análise e, portanto, essa recomendação não pode ser estendida a outros BBs não avaliados.^211^

O atenolol, em comparação a anlodipino e losartana, apresentou uma incidência pouco maior de eventos CV, com certeza de evidência baixa e moderada, respectivamente.^211^

Nessa análise, a combinação de hidroclorotiazida (HCTZ) e amilorida foi a intervenção que apresentou maior redução de eventos CV, embora com certeza de evidência muito baixa. A redução da PA, no entanto, foi semelhante entre todas as comparações medicamentosas, podendo ser constatada pequena diferença em relação à redução a desfechos CV a favor de diuréticos tiazídicos, anlodipino e losartana. Note-se que essa é uma recomendação fraca, com baixa ou muito baixa certeza da evidência, à exceção da comparação de atenolol com losartana, que apresentou moderada certeza da evidência (entre os resultados em que houve diferença no efeito das estratégias comparadas).^211^

Assim, com os resultados obtidos, o uso de atenolol é uma opção para o tratamento de HA primária em associação a outros anti-hipertensivos considerados de primeira escolha (diuréticos tiazídicos ou tiazídicos similares em baixas doses, bloqueadores do sistema renina-angiotensina-aldosterona (SRAA) e BCCs. Deve-se individualizar essa prescrição de acordo com as melhores indicações, comorbidades, características clínicas e preferências de cada indivíduo.^211^

Os Quadros 7.2 a 7.7 resumem todos os medicamentos disponíveis do Brasil, de acordo com suas características farmacológicas, doses e frequência de administração, principais indicações e informações adicionais, que incluem os principais efeitos colaterais.

**Quadro 7.7:** Outros medicamentos utilizados no tratamento da hipertensão arterial: simpatolíticos e vasodilatadores de ação direta, suas doses diárias habituais (mg/dia), frequência de tomadas (número de doses/dia) e informações adicionais

Classe e medicamento	Dose diária habitual (mg)	Frequência (número de doses/dia)	Informações adicionais e recomendações
**SIMPATOLÍTICOS DE AÇÃO CENTRAL**			
Metildopa	500 – 2000	2	– Medicamento de escolha na HA durante a gestação, praticamente sua única indicação.
Clonidina	0,2 – 0,9	2 a 3	– A retirada abrupta da clonidina pode provocar hipertensão rebote (crise hipertensiva) por liberação de catecolaminas na terminação sináptica. – Pode ser utilizada em elevação aguda da PA e na HA resistente e refratária como um dos medicamentos, especialmente quando ocorrem efeitos adversos intoleráveis com espironolactona ou eplerenona.
**SIMPATOLÍTICOS DE AÇÃO PERIFÉRICA**			
Prazosina	1 – 20	2 a 3	– Iniciar com dose baixa antes de deitar-se, pois pode provocar hipotensão ortostática. Aumentar progressivamente a dose a cada 2 dias. – Pode induzir taquifilaxia e efeito de primeira passagem.
Doxazosina	1 – 16	1	– Outros alfabloqueadores indicados para hiperplasia benigna de próstata, com uso eventual em HA: tansulosina, alfuzosina, silodosina.
Terazosina	1 – 20	1	
**VASODILATADORES DE AÇÃO DIRETA**			
Hidralazina	50 – 200	2 a 3	– Pode provocar retenção de sódio e água, hipervolemia e taquicardia reflexa. – Pode ser necessária a associação com diuréticos de alça e/ou betabloqueadores. – Não utilizar isoladamente. – Possível uso durante a gestação.
Minoxidil	5 – 40	1	– Indicado em hipertensão resistente ou refratária, hipertensão maligna, ou para a retirada de medicamentos vasodilatadores intravenosos – Mesmos efeitos (adversos ou não) da hidralazina, exceto não poder ser utilizado na gestação e causar hirsutismo.

HA: hipertensão arterial; PA: pressão arterial

### 7.5. Medicamentos Indicados para outras Doenças e que têm Ação sobre a Pressão Arterial e Eventos Cardiovasculares, porém ainda Não Consolidados como Rotina no Tratamento da Hipertensão Arterial

#### 7.5.1. Sacubitril/Valsartana

Inicialmente desenvolvido para tratar HA, mostrou benefícios em mortalidade e morbidade em pacientes com ICFEr, porém faltam dados para indicá-lo no tratamento da HA isoladamente.^45,114,199,211,222^

Uma metanálise mostrou que sacubitril/valsartana foi mais eficaz em diminuir a PA quando comparada à olmesartana,^223,224^ e outra metanálise em rede, sobre uso dessa medicação em HA primária, concluiu que a melhor dose em termos de eficácia anti-hipertensiva avaliada pela MAPA foi de 200 mg/dia.^225,226^ De qualquer forma, mais ECRs são necessários para determinar o valor incremental do sacubitril/valsartana como agente anti-hipertensivo em relação às classes de anti-hipertensivos já recomendadas, pois não há RS e metanálise que tenha abordado desfechos duros e efeitos adversos, como hipotensão. Tosse e angioedema são efeitos colaterais que podem ser observados. Lembrar que a associação de IECA ou BRA com sacubitril/valsartana não é recomendada.

#### 7.5.2. Inibidores de Cotransportador de Sódio-Glicose Tipo 2

Esses medicamentos mostraram efeitos positivos em eventos CV e na função renal, tanto em pacientes com diabetes quanto em pacientes com DRC de outras causas e na IC. No entanto, têm ação pouco significante sobre a diminuição da PA.^213-215^

Os mecanismos anti-hipertensivos dos inibidores do cotransportador de sódio-glicose 2 (*sodium/glucose cotransporter 2 inhibitors* – iSGLT2) envolvem natriurese e diurese osmótica, levando à redução do volume intravascular e da resistência vascular periférica (RVP). Além disso, há ativação do feedback tubuloglomerular e a perda de peso associada ao uso desses agentes pode contribuir adicionalmente para a redução pressórica. A presença de receptores no sistema nervoso central (SNC) deve ter importância nessa ação modesta sobre a PA.^227^

Uma metanálise demonstrou que os iSGLT2 reduzem significativamente a PAS e a PAD pela MAPA, em pacientes com DM2 e HA, com uma diferença média de −5,08 mmHg e −2,73 mmHg, respectivamente.^228^

Há estudos consistentes na literatura de que os iSGLT2 promovem redução modesta, porém consistente, da PA em pacientes com DM2, com menor magnitude em outras populações. Esses efeitos são clinicamente relevantes, especialmente considerando o perfil de segurança e os benefícios cardiorrenais adicionais desses medicamentos.^228-231^

#### 7.5.3. Agonistas de Peptídeo Semelhante a Glucagon 1^216^ e os Novos Antagonistas dos Receptores de Mineralocorticoides Não Hormonais Seletivos^218,232^

A finerenona, um antagonista dos receptores mineralocorticoides (ARM) não hormonal seletivo, disponível no Brasil, e os agonistas de peptídeo semelhante a glucagon 1 (*Glucagon-like peptide-1* – GLP-1), no geral, parecem ter ação discreta sobre a PA, mas ainda carecem de mais evidências científicas para serem recomendados como tratamento anti-hipertensivo, embora devam ser considerados nas condições clínicas estudadas em que demonstraram nefro e cardioproteção.

Uma metanálise mostrou que os agonistas de GLP-1 (aGLP-1) reduziram a PAS em média em 3,37 mmHg e a PAD em 1,05 mmHg em comparação com o placebo.^233^ Outro estudo corroborou esses achados, relatando reduções modestas na PAS com diferentes aGLP-1, como semaglutida, liraglutida e dulaglutida.^233^

Revisões em rede, que comparam diferentes aGLP-1, identificam que moléculas como a semaglutida subcutânea apresentam maior eficácia na redução da PAS e na PAD entre os agentes da classe.^234^

Quanto à finerenona, a análise de MAPA no estudo ARTS-DN (*Mineralocorticoid Receptor Antagonist Tolerability Study–Diabetic Nephropathy*) revelou que esse ARM diminuiu a PAS de 24 horas, diurna e noturna, com uma redução ajustada para o placebo de até −11,2 mmHg em doses de 15 mg.^235^

A maioria das metanálises e RS com finerenona concentra-se em desfechos renais e CV em pacientes com DRC associada ao DM2, sendo a redução da PA um desfecho secundário ou exploratório.^236-238^

### 7.6. Estratégias de Tratamento

Observa-se que o tratamento pode ser iniciado com monoterapia em casos selecionados (Figura 7.3) ou em associação, sendo que a maioria dos pacientes terá indicação para iniciar o tratamento com dois medicamentos, preferencialmente em um único comprimido.

**Figura 7.3:**
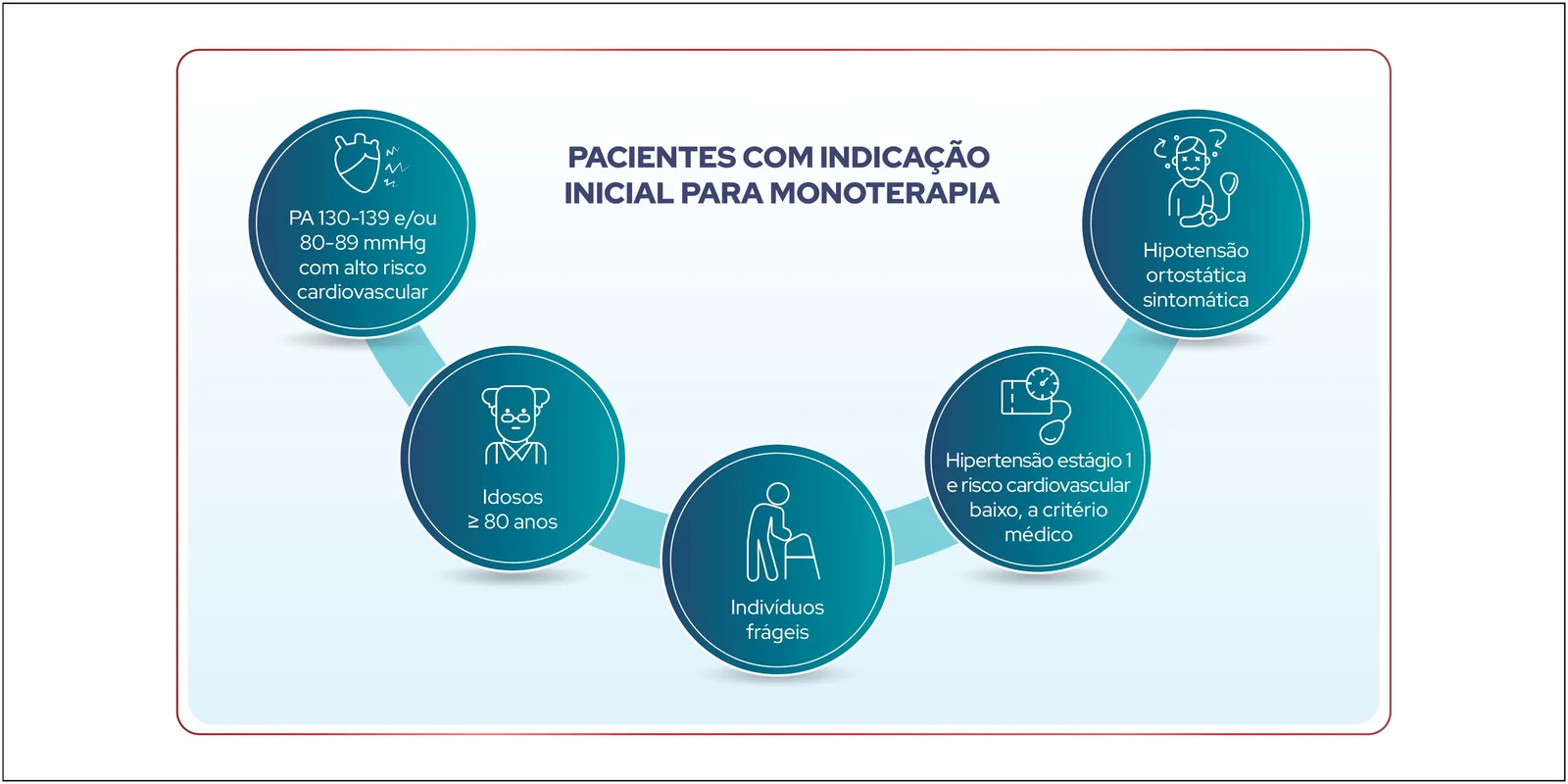
Pacientes com indicação inicial para monoterapia.

O fluxograma do tratamento medicamentoso está exposto na Figura 7.4.

**Figura 7.4:**
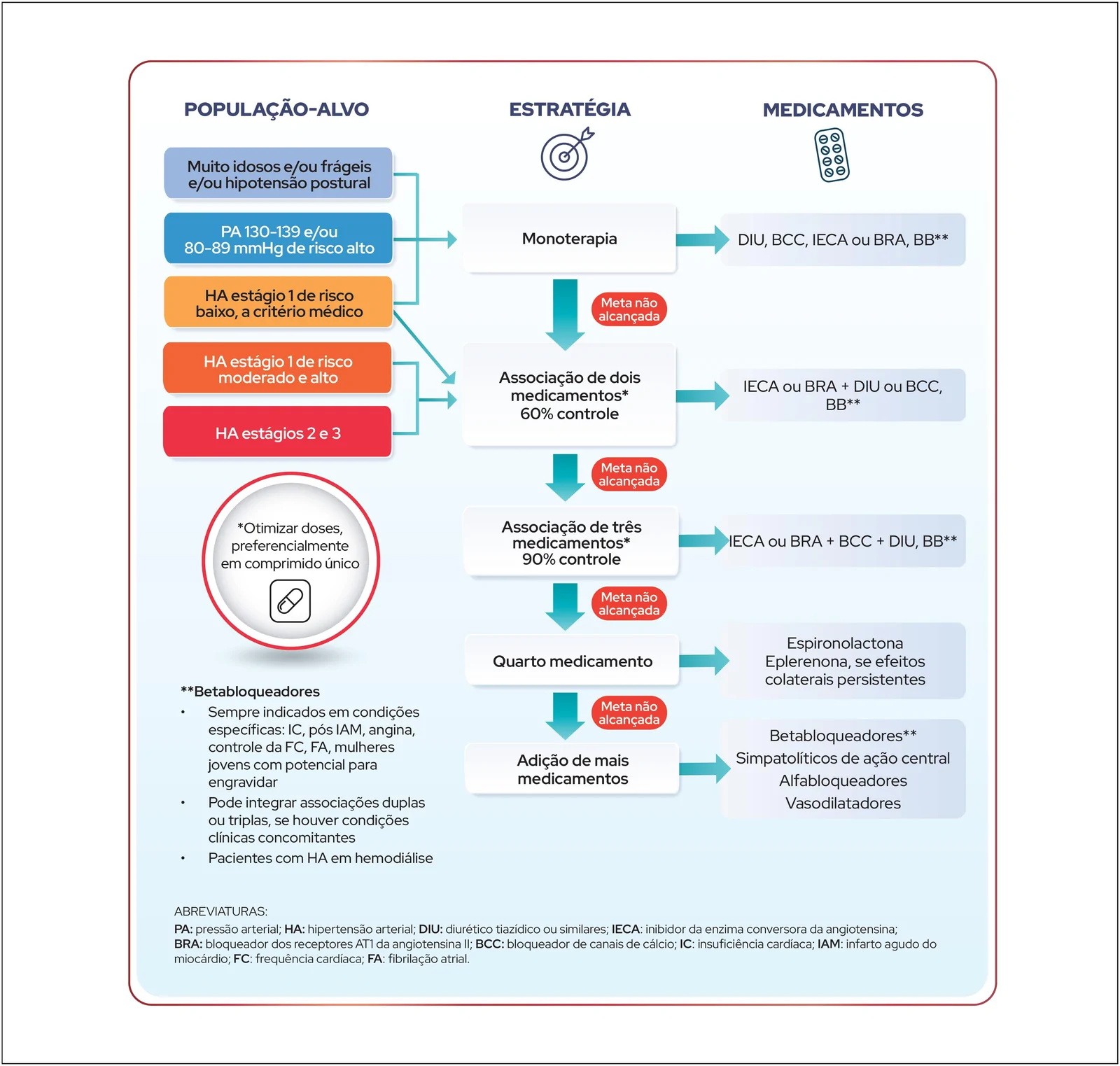
Fluxograma do tratamento medicamentoso da hipertensão arterial.

#### 7.6.1. Monoterapia

A monoterapia está indicada como estratégia anti-hipertensiva inicial e excepcional para pacientes como estabelecido na Figura 7.3.

Nesses grupos de pacientes, a redução da PA desejada é pequena e/ou a redução da PA deve ser gradual, de modo a se evitar eventos adversos graves.

O tratamento deve ser individualizado, e a escolha do medicamento inicial deverá ser baseada nas características dos anti-hipertensivos, nas particularidades de cada paciente, na presença de doenças associadas e LOA, considerando, também, as condições socioeconômicas e a disponibilidade dos medicamentos no SUS para as pessoas a serem tratadas.^239-245^

Os BBs podem ser considerados como o medicamento inicial em situações específicas, conforme já descrito anteriormente; no entanto, são mais frequentemente usados em associação com outros medicamentos.^211,213,246,247^

#### 7.6.2. Associação de Medicamentos

Para a maioria dos pacientes, é recomendado o início do tratamento da HA com associação dupla de medicamentos, preferencialmente em um comprimido único, se disponível.^239-243^ A critério médico, hipertensos estágio 1, com baixo risco CV, podem ser tratados como monoterapia (Figura 7.3) ou associação de medicamentos.

As associações preferidas devem incluir um bloqueador do SRAA (IECA ou BRA) com um BCC ou com um diurético tiazídico ou similar. Essas estratégias são apoiadas por evidências que mostraram que a terapia combinada, preferencialmente em comprimido único, produz maiores reduções da PA do que a monoterapia, causa menos efeitos colaterais que os componentes individuais, melhora a adesão terapêutica e, a persistência do tratamento a longo prazo, permite um controle mais precoce da PA^114,198-200,225-233,248^ e reduz a inércia terapêutica e o risco de mortalidade total e hospitalizações por DCV.^245,248,250-252^

Nos programas do Ministério da Saúde de distribuição de medicamentos, não há associações disponíveis em um único comprimido e, portanto, para os usuários do SUS, devem ser utilizados os medicamentos disponíveis em associação em múltiplos comprimidos, com os ajustes nas doses individuais toleradas dos medicamentos constantes na Relação Nacional de Medicamentos Essenciais (RENAME), conforme descrito no Capítulo 14, para que as metas de PA sejam atingidas.

Caso a meta de PA não seja alcançada com dois medicamentos, ajustes de doses até as máximas preconizadas e toleradas deverão ser utilizados de acordo com as particularidades de cada paciente. Na sequência, outras classes de medicamentos anti-hipertensivos deverão ser acrescentadas até ser alcançado o controle da PA, seguindo o fluxograma da Figura 7.4. Destaca-se que combinações triplas também devem ser preferencialmente administradas em comprimido único, quando possível.

É importante ressaltar que a associação de BRA com IECA é contraindicada, por promover aumento de efeitos adversos, sem redução de desfechos.^235^ Essa contraindicação é baseada principalmente nos desfechos desfavoráveis observados no estudo ONTARGET (*Ongoing Telmisartan Alone and in combination with Ramipril Global Endpoint Trial*),^253^ mas há controvérsias advindas de duas revisões sistemáticas,^254,255^ o que confere uma fraca força de recomendação com baixa certeza da evidência.^256-259^

**Table:** 

Mensagens principais sobre o tratamento da hipertensão arterial
O tratamento medicamentoso deve obrigatoriamente associar-se às mudanças do estilo de vida (ver Capítulo 6).
A associação de dois medicamentos em doses baixas, em um único comprimido ou não, é a estratégia inicial recomendada para a maioria dos hipertensos, utilizada pela manhã ou à noite, permitindo controle da PA em mais de 60% dos pacientes.
Quando não se consegue o controle da PA com a associação dupla, mesmo com o aumento de doses individuais até as máximas toleradas, a mudança para a associação tripla deve ser a opção, em comprimido único quando possível. A associação tripla permite o controle da PA em cerca de 90% dos indivíduos sob tratamento.
A espironolactona deve ser a quarta opção medicamentosa. Se houver eventos adversos persistentes, deverá ser substituída por eplerenona. A adição de mais classes de medicamentos deve ser realizada progressivamente, conforme recomendações desta diretriz, de acordo com o fluxograma do tratamento apresentado, a fim de se obter a meta de PA.
Se, mesmo assim, não for obtida a meta de PA, caracteriza-se como paciente com HAR ou HARf e deverá ser encaminhado a um centro especializado para investigações adicionais (ver Capítulo 12).

HAR: hipertensão arterial resistente; HARf: hipertensão arterial refratária; PA: pressão arterial.

**Table:** 

Recomendações para o tratamento medicamentoso	Força de recomendação	Certeza da evidência
Recomenda-se a associação de medicamentos, preferencialmente em comprimido único e com as classes preferenciais, para atingir metas rigorosas de PA (< 130/80 mmHg) e reduzir eventos CV e renais.	FORTE	MODERADA
Recomenda-se monoterapia para indivíduos com PA 130-139/80-89 mmHg e alto risco CV; pacientes com HA Estágio 1 de baixo risco (a critério médico, pode-se optar por associação); em indivíduos frágeis; muito idosos (≥ 80 anos) ou com hipotensão ortostática sintomática, especialmente em indivíduos idosos.	FRACA	BAIXA
Recomenda-se, para a maioria dos pacientes, o início do tratamento da HA com associação dupla de medicamentos, em doses baixas, preferencialmente em um comprimido único, se disponível.	FORTE	MODERADA
Recomenda-se o uso de diuréticos tiazídicos ou similares, IECA ou BRA e BCC como classes preferenciais para o tratamento da HA e redução de eventos CV e renais importantes.	FORTE	ALTA
Recomenda-se o uso de BB para o tratamento de HA em situações específicas: IC, FA, arritmias, DAC, HA em pacientes em HD e outras (enxaqueca, tremor essencial, mulheres que desejam engravidar, varizes esofágicas).	FORTE	MODERADA
Recomenda-se o uso de espironolactona (ou eplerenona, caso ocorra intolerância à espironolactona) para atingir metas rigorosas de PA (< 130/80 mmHg) e reduzir eventos CV e renais, quando não se tenha atingido apenas com três classes iniciais (HAR e HARf).	FORTE	ALTA
Recomenda-se o uso de clonidina para atingir metas rigorosas de PA (< 130/80 mmHg) e reduzir eventos CV quando não se tenham atingido as metas de PA apenas com as classes iniciais (HAR e HARf) e intolerantes à espironolactona/eplerenona, ou ainda, sem controle após sua associação.	FRACA	BAIXA
Não se recomenda o uso concomitante de IECA e BRA.	FORTE	ALTA

BCC: bloqueador dos canais de cálcio; BRA: bloqueador dos receptores da angiotensina; CV: cardiovascular; FA: fibrilação atrial; DAC: doença arterial coronariana; HA: hipertensão arterial; HAR: hipertensão arterial resistente; HARf: hipertensão arterial refratária; HD: hemodiálise; IC: insuficiência cardíaca; IECA: inibidor da enzima conversora da angiotensina; PA: pressão arterial.

## 8. Hipertensão Arterial e Condições Clínicas Associadas: Doença Arterial Coronariana, Doença Renal Crônica, diabetes mellitus, Obesidade, COVID-19, Pós-Acidente Vascular Cerebral e Insuficiência Cardíaca

### 8.1. Hipertensão Arterial e Doença Arterial Coronariana

O paciente com HA e DAC é considerado de alto ou muito alto risco CV.^260^ O envelhecimento desempenha papel importante na influência da relação dos parâmetros de PA com o risco de DAC. Em pacientes com menos de 50 anos de idade, a PAD é um preditor mais forte do risco do que a PAS ou a PP, sugerindo que o aumento da RVP e a amplificação alterada da onda de pulso são dominantes na determinação do risco de DAC. Entre as idades de 50 e 59 anos, os três parâmetros de PA são preditores semelhantes do risco, sugerindo um equilíbrio entre a resistência dos pequenos vasos e a rigidez das grandes artérias. A partir dos 60 anos, há uma mudança em favor da PP e da PAS como preditores do risco de DAC, sugerindo que a rigidez das grandes artérias com reflexão precoce da onda de pulso é o determinante hemodinâmico dominante do risco. Embora a PAD predomine sobre a PAS em adultos jovens, a maior carga de DCV ocorre em indivíduos mais velhos com HA sistólica isolada e PP ampla.^261^

A diminuição da PA está associada a redução do risco CV nos pacientes com DAC. No entanto, estudos iniciais sugeriram que uma PAD muito baixa poderia comprometer a perfusão coronariana. Isso é particularmente relevante em pacientes com DAC, nos quais uma redução excessiva da pressão diastólica poderia precipitar isquemia miocárdica.^262^ O estudo TNT (*Treating to New Targets*) mostrou que PAS muito baixas, menores que 110-120/60-70 mmHg, estão associadas a aumento no risco de eventos CV, exceto para AVC, em que pressões mais baixas são benéficas.^262^

Há, no entanto, uma discussão sobre a possível existência de curva em J, já abordada no Capítulo 5. Em coorte retrospectiva de pacientes coreanos com DAC e submetidos à intervenção coronariana percutânea, verificou-se curva em J para complicações maiores, com nadir em 119 mmHg para PAS e 74 mmHg para PAD.^263^ Já uma análise *post hoc* de estudo em população nipônica sobre o efeito de anti-hipertensivos em doentes com DAC não identificou curva em J para pressões sistólicas e nem tampouco em pacientes japoneses com DAC submetidos à intervenção coronariana percutânea.^264^

Evidências da curva J da PA são derivadas de estudos observacionais, com baixa certeza da evidência e com alta probabilidade de não refletir uma relação causal entre redução da PA e desfechos CV. Mais provavelmente, estão relacionadas à presença de fatores de confusão residuais e/ou causalidade reversa.^128,130,265^

#### 8.1.1. Metas de Pressão Arterial na Doença Arterial Coronariana

A presente diretriz recomenda metas de PA < 130/80 mmHg para indivíduos com HA e DAC. Obviamente, há um limite a partir do qual a redução da PA atingida pode causar malefício CV, mas não há evidências que respaldem qual é esse valor de PA e o quanto ele pode diferir, de acordo com comorbidades presentes. Admite-se que reduzir a PA até 120/70 mmHg não aumenta o risco CV, e pacientes com PA < 120/70 mmHg e assintomáticos não necessitam parar ou reduzir a medicação anti-hipertensiva em uso. A redução da PA deve ser feita de forma gradual e com cautela, de acordo com a tolerância, particularmente nos pacientes com evidência de isquemia miocárdica, nos indivíduos com diabetes e nos muito idosos.^266,267^

Recomenda-se atenção para eventos adversos como a hipotensão ortostática, síncope, alterações eletrolíticas e injúria renal aguda.^266,267^ Na presença de PP elevada, a redução de PA deve igualmente ser gradual e cuidadosa, pelo possível aparecimento ou surgimento de isquemia miocárdica.^206^

#### 8.1.2. Tratamento da Hipertensão Arterial e Doença Arterial Coronariana

Os BB e os BCC di-hidropiridínicos e não di-hidropiridínicos são preferenciais para o tratamento da HA em pacientes sintomáticos com DAC. Na presença de IAM recente, os BB também melhoram o prognóstico e devem ser prescritos, a menos que contraindicados.^268-270^ A duração dos benefícios relacionados aos BB é incerta. No entanto, na ausência de inconvenientes específicos, não há razão para interromper a terapia com BB.

É importante mencionar que o aumento da FC se correlaciona linearmente com eventos CV, e o benefício da redução da FC como meta de tratamento em pacientes com DAC foi demonstrado por vários medicamentos, incluindo os BB.^271^ Os IECAs demonstraram reduzir os desfechos CV em pacientes de alto risco CV. Os BRA podem substituir os IECAs em pacientes com HA e DAC que sejam intolerantes a essa classe de medicamentos.^271^

**Table:** 

Mensagens principais sobre hipertensão arterial no paciente com doença arterial coronariana
O controle da PA diminui o risco de eventos coronarianos.
Há controvérsias em relação à existência de curva em J na DAC, pois as evidências favoráveis são de baixa qualidade.

PA: pressão arterial; DAC: doença arterial coronariana.

**Table:** 

Recomendações para o paciente com hipertensão arterial e doença arterial coronariana	Forca da recomendação	Certeza da evidência
Recomenda-se metas de PA < 130/80 mmHg para indivíduos com HA e DAC. Admite-se que reduzir a PA até 120/70 mmHg não aumenta o risco CV e que pacientes com PA < 120/70 mmHg e assintomáticos não necessitam parar ou reduzir a medicação anti-hipertensiva.	FORTE	MODERADA
Recomenda-se que a redução da PA em pacientes hipertensos com DAC deva ser feita de forma gradual e com cautela, de acordo com a tolerância, nos pacientes com evidência de isquemia miocárdica, nos pacientes com diabetes, muito idosos e naqueles com PP elevada, com atenção aos EAs como a hipotensão ortostática, síncope, alterações eletrolíticas e IRA.	FRACA	BAIXA
Recomenda-se o uso de BB para o tratamento da HA em pacientes sintomáticos com DAC para a redução de eventos CV importantes e sintomas anginosos.	FORTE	ALTA
Recomenda-se o uso de IECA para a redução de desfechos CV em pacientes com DAC, sendo que os BRAs podem substituir os IECAs nos pacientes que sejam intolerantes a essa classe de medicamentos.	FORTE	ALTA
Recomenda-se a utilização dos BCCs para o tratamento de HA e DAC concomitante, especialmente em pacientes com angina, para redução de eventos CV importantes e sintomas anginosos.	FRACA	MODERADA

BB: betabloqueador; BCC: bloqueador dos canais de cálcio; BRA: bloqueador dos receptores da angiotensina; CV: cardiovascular; DAC: doença arterial coronariana; EAs: efeitos adversos; HA: hipertensão arterial; IECA: inibidor da enzima conversora da angiotensina; IRA: injúria renal aguda; PA: pressão arterial; PP: pressão de pulso.

### 8.2. Hipertensão Arterial na Doença Renal Crônica em Pacientes Sob Tratamento Conservador e na Diálise

#### 8.2.1. Meta de Pressão Arterial para Doença Renal Crônica em Tratamento Conservador

A HA é o principal fator de risco para DRC no Brasil e, portanto, precisa ser tratada de modo eficaz e precoce, evitando-se a inércia terapêutica.^273^ Trata-se de uma população complexa, que frequentemente apresenta hipertensão arterial resistente (HAR) ou hipertensão arterial refratária (HARf), acompanhada de outras comorbidades e elevado risco de evolução para falência renal e mortalidade CV quando não controlada.^274^ A meta de PA para esses pacientes ainda suscita muitas controvérsias na literatura.^275,276^

Em pacientes com HA e DRC em tratamento conservador, o estudo SPRINT é utilizado como parâmetro, propondo uma meta de PAS < 120 mmHg, comparativamente à PAS < 140 mmHg,^256^ como medida redutora do risco CV e da mortalidade em adultos que não têm diabetes e com elevado risco CV.^277^ Apesar de ter sido a base para a atualização do KDIGO 2024^95^ – principal diretriz internacional para o manejo da DRC e suas complicações – essa meta proposta é um desafio em contextos clínicos reais de rotina, especialmente fora de ambientes especializados.^276^

O estudo SPRINT possui algumas características que minimizam a extrapolação de suas recomendações para todos os pacientes com HA e DRC. Um importante ponto a se considerar foram os critérios de exclusão: idade inferior a 50 anos, TFGe < 20 mL/min/1,73 m^2^, proteinúria > 1 g/dia, pacientes com DM, rins policísticos ou com antecedente de AVC, critérios esses que incluem parte expressiva dos pacientes com diagnóstico de DRC no Brasil.^278,279^ Além disso, a PA foi medida com esfigmomanômetros profissionais automatizados, que não estão disponíveis comercialmente no Brasil (modelo 907XL, Omron®), e foi realizada frequentemente desacompanhada, o que geralmente resulta em valores inferiores aos medidos em consultório e, portanto, não podem ser extrapolados para medidas não padronizadas.^49^

Finalmente, a avaliação de desfechos renais não foi o objetivo primário do estudo SPRINT.^280^ Assim, não é possível afirmar, a partir dos seus resultados, que o controle intensivo da PA seja capaz de retardar a progressão da DRC ou mesmo que não acelere tal progressão, uma vez que pode aumentar significativamente o risco de hipotensão, hipofluxo renal com injúria renal aguda (IRA), anormalidades eletrolíticas, síncope e outros efeitos colaterais.^256^

De acordo com uma RS da Cochrane (2024), que incluiu seis ECRs com 7.348 participantes, metas de PA mais baixas (≤ 130/80 mmHg) em comparação com metas consideradas padrão (140-160/90-100 mmHg) não mostraram diferenças significativas em mortalidade total, eventos CV totais ou progressão para DRC dialítica. No entanto, os participantes que seguiram metas mais baixas alcançaram valores de PAS e PAD mais baixos e necessitaram de um maior número de medicamentos anti-hipertensivos.^281^

Especificamente em pacientes com DM, o controle da PA determina: redução da albuminúria, melhora da retinopatia e redução de AVC, com metas mais intensivas, porém sem efeitos em outros desfechos CV.^282^ Não foi observada redução de eventos CV em pacientes com DM no estudo ACCORD,^283^ com níveis de PAS < 120 mmHg, fato atribuível ao reduzido poder estatístico do desenho fatorial do estudo. Por outro lado, em análise *post hoc* de longo prazo do estudo IDNT (*Irbesartan Diabetic Nephropathy Trial*), a redução da PAS até 120 mmHg melhorou a sobrevida renal e dos pacientes.^95^

Pelo exposto, a meta de PA recomendada por esta diretriz para pacientes com DRC em tratamento conservador é < 130/80 mmHg. A meta de PAS < 120 mmHg pode ser almejada em casos selecionados, particularmente em pacientes mais jovens, com menos comorbidades, sem fragilidade, com a PA medida por método automático, preferencialmente desacompanhados, e sob vigilância.

O debate sobre a melhor meta de PA para prevenção de desfechos CV e renais precisa ser normalizado para que se tenha uma mensagem única em todas as diretrizes de HA e de DM, mas enquanto mais ECRs não estiverem disponíveis, com aplicabilidade global, mesmo em países com recursos limitados, há que se ter cautela, pois haverá discordâncias que passam inclusive por instituições de alta confiabilidade como o KDIGO e a Cochrane.^117,245,276,281,284-289^

#### 8.2.2. Meta de Pressão Arterial para Doença Renal Crônica em Diálise

Não há publicação de ECR de longo prazo, desenhado com a finalidade específica de definir os alvos ideais de PA em pacientes com HA em diálise. Diversos estudos observacionais demonstraram um comportamento dos níveis de PA desses pacientes em relação à mortalidade, como uma curva em U (ou J).^290-292^

O estudo CRIC (*The Chronic Renal Insufficiency Cohort Study*) também demonstrou uma relação entre mortalidade por todas as causas e PAS em pacientes em hemodiálise (HD) (n = 326) em forma de U para as PAS medidas no centro de diálise. Porém, para as medidas de PAS fora do centro de diálise, esse mesmo estudo encontrou uma associação linear com a mortalidade. Esses resultados sugerem que a avaliação da PA em pacientes em diálise deve ser determinada, idealmente, no período interdialítico, preferencialmente pela MAPA de 44 h, ou MRPA, se a MAPA estiver indisponível.^293^

Diante da ausência de dados robustos em mais de um ECR, e considerando o maior risco de hipotensão, pior adesão e possível agravamento das lesões renais em pacientes com controle intensivo da PA, consideramos razoável preconizar uma meta de PA < 130/80 mmHg para pacientes em tratamento conservador ou transplantados renais.^281^ Para pacientes em HD ou hemofiltração, recomenda-se uma meta de PA pré-diálise < 140/90 mmHg e PA pós-diálise < 130/80 mmHg. A medida da PA pós-diálise deve ser realizada após a selagem do cateter ou hemostasia da fístula arteriovenosa.^294^ Finalmente, para a diálise peritoneal, uma meta de PA < 140/90 mmHg é sugerida, preferencialmente aferida pela MAPA de 24 h.^295^

#### 8.2.3. Tratamento da Hipertensão Arterial na Doença Renal Crônica em Tratamento Conservador

Em associação ao tratamento medicamentoso, a implementação de MNM é fundamental^95^ (ver Capítulo 6).

Os IECAs ou os BRAs são medicamentos de primeira escolha para pacientes com HA e DRC, com ou sem albuminúria, por seus efeitos na redução de eventos renais e CV, não sendo recomendada a sua associação. Diuréticos tiazídicos ou tiazídicos similares e de alça e os BCC são eficazes e adequados para associações. Os BB estão indicados na DAC e na IC. Os ARMs (espironolactona e eplerenone) possuem ação cardioprotetora e são eficazes em HARf, porém o risco de hiperpotassemia é elevado, particularmente em pacientes com DRC Estágios 3b-5, o que não contraindica seu uso e sim o seu controle.^95^

Recentemente, medicamentos com ação anti-hipertensiva discreta, mas com eficácia comprovada em proteção renal e cardíaca, têm sido integrados ao tratamento de pacientes com DRC, especialmente nos pacientes com diabetes. Os iSGLT2 se destacam, reduzindo o risco de desfechos renais e CV em pacientes com e sem DM e com TFG-e > 20 mL/min.^231,296^ A finerenona, um ARM não esteroide, também oferece proteção renal e CV, comprovada em pacientes com DM e DRC.^297,298^ Adicionalmente, a semaglutida, um aGLP-1, reduziu eventos renais e CV graves em pacientes com DRC e DM2.^276^

**Table:** 

Mensagens principais sobre hipertensão arterial no paciente com doença renal crônica em tratamento conservador
A HA é a principal causa de DRC no Brasil e, portanto, precisa ser tratada de modo eficaz e precoce.
Frequentemente, a HA na DRC é resistente ou refratária ao tratamento.
A meta de PA nos pacientes com DRC em tratamento conservador é controversa, com RS e metanálises divergentes quanto ao alvo de PA, mesmo entre instituições de alta confiabilidade como a Cochrane e o KDIGO.

HA: hipertensão arterial; DRC: doença renal crônica; PA: pressão arterial; RS: revisão sistemática.

#### 8.2.4. Tratamento da Hipertensão Arterial na Doença Renal Crônica em Diálise

Por se tratar de assunto específico do nefrologista, recomendamos que sejam seguidas as orientações da I Diretriz Brasileira de Hipertensão na Diálise da SBN para as MNM e para o tratamento medicamentoso, que estão sintetizadas abaixo.^295^

Recomenda-se aos pacientes em diálise o uso de:

- Diurético de alça para todos os pacientes em diálise com diurese residual;
- IECA ou BRA em pacientes com HA em diálise, especialmente por seus efeitos pleiotrópicos: redução HVE, IC, fibrose peritoneal em diálise peritoneal (DP) e mortalidade CV;
- BBs são os medicamentos anti-hipertensivos preferíveis em HD e DP, especialmente atenolol, exceto se contraindicado;
- Recomenda-se uso de BCC em pacientes em HD ou DP, por seus efeitos hipotensores e de proteção CV;
- Recomenda-se uso de ARM em pacientes em HD ou DP por seus efeitos anti-hipertensivos, na redução da mortalidade e de proteção CV, se tolerados.

**Table:** 

Recomendações para o paciente com hipertensão arterial e doença renal crônica em tratamento conservador^231,299,300^	Forca da recomendação	Certeza da evidência
Recomenda-se, em adultos com HA e DRC, a meta de PA < 130/80 mmHg para a redução de eventos CV e falência renal.	FORTE	ALTA
Recomenda-se, para pacientes em tratamento conservador de DRC, a meta de PAS < 120 mmHg, em casos selecionados, particularmente em pacientes mais jovens, com menos comorbidades, sem fragilidade, com a PA medida por método automático, preferencialmente desacompanhada e sob vigilância.	FORTE	MODERADA
Recomenda-se como meta para pacientes em diálise: • HD: uma meta de PA pré-sessão < 140/90 mmHg ou PA < 130/80 mmHg na pós-HD; • Hemodiafiltração: < 130/80 mmHg; • DP: < 140/90 mmHg.	FRACA	MUITO BAIXA
Recomenda-se para pacientes em HD ou DP que a avaliação da PA deve ser feita preferencialmente pela MAPA de 44 h ou de 24 h, respectivamente. MRPA é a opção, se a MAPA estiver indisponível.	FRACA	BAIXA
Recomenda-se que a escolha de anti-hipertensivos na HA da DRC deva incluir, a menos que contraindicado, o uso de IECA ou BRA, e favorecer a introdução e manutenção de medicamentos com efeito anti-hipertensivo discreto, mas que exibem propriedades nefro e cardioprotetoras, como a finerenona e os aGLP1 em pacientes com DM, além dos iSGLT2, devido ao comprovado efeito benéfico na proteção cardiorrenal.	FORTE	ALTA
Recomenda-se a monitorização do potássio e da redução da função renal durante o tratamento da HA na DRC em tratamento conservador.	FORTE	ALTA

aGLP1: análogos dos peptídeos semelhantes ao glucagon 1 (*Glucagon-like peptide*-1); BRA: bloqueador dos receptores de angiotensina; CV: cardiovascular; DP: diálise peritoneal; DRC: doença renal crônica; HA: hipertensão arterial; HD: hemodiálise; IECA: inibidor da enzima conversora da angiotensina; iSGLT2: inibidores do cotransportador de sódio-glicose 2 (*sodium/glucose cotransporter 2 inhibitors*); MAPA: medida ambulatorial da pressão arterial; MRPA: medida residencial da pressão arterial; PA: pressão arterial; PAS: pressão arterial sistólica; DM: diabetes mellitus.

### 8.3. Hipertensão Arterial e Diabetes Mellitus

A associação de HA e DM é frequente e parece se relacionar de maneira bidirecional, isto é, a HA pode contribuir para o desenvolvimento e agravamento do DM, como o contrário também pode ser verdadeiro. Aproximadamente 80 a 90% dos pacientes com DM desenvolverão HA, tornando complexo o tratamento da doença e aumentando o risco de complicações micro e macrovasculares, como doença renal, retinopatia, doença arterial obstrutiva periférica (DAOP) e doenças CV, o que torna essencial o controle rigoroso da PA.^278^ As avaliações de natureza clínica e complementar devem sempre ser feitas conforme exposto no Capítulo 4.

Destacam-se a pesquisa e classificação de albuminúria em amostra isolada ou preferencialmente, RAC para a avaliação do comprometimento renal, não sendo recomendada a utilização das nomenclaturas micro ou macroalbuminúria^289^ (ver Capítulo 4). Ressalta-se que a presença de albuminúria deve ser comprovada em duas ou três amostras de urina, visto que exercícios, febre e refeições ricas em proteínas podem interferir no resultado e não necessariamente significar dano renal.

Recomenda-se monitorar regularmente a PA fora do consultório. MAPA e MRPA são recomendadas para identificar HM e HAB, situações frequentes em pacientes com DM,^301^ tanto para o diagnóstico como para a avaliação do tratamento.

Pacientes com DM bem controlado, com duração inferior a 10 anos, sem evidência de LOA e sem fatores de risco CV adicionais são categorizados como de risco moderado. No entanto, para pacientes que têm risco mais elevado de DCV, como aqueles com histórico de eventos CV ou evidência de dano a órgãos-alvo, o risco é alto.^255,302-304^

#### 8.3.1. Metas na Hipertensão Arterial no Paciente com Diabetes Mellitus

A associação entre HA e DM aumenta o risco de complicações micro e macrovasculares, tornando essencial o controle rigoroso da PA. Por outro lado, não há dados disponíveis em pacientes com DM de início recente, sem complicações e, portanto, com risco CV relativamente baixo. De forma geral, o controle da PA é mais difícil nos indivíduos com DM.

O estudo ACCORD^283^ não foi capaz de confirmar que uma meta de PAS abaixo de 120 mmHg resulta em diminuição nas taxas de eventos CV nos indivíduos com DM. Todavia, nesse estudo, a incidência de AVC foi reduzida em 41% com o tratamento intensivo. O estudo ADVANCE (*Action in Diabetes and Vascular Disease: Preterax and Diamicron MR Controlled Evaluation*)^305^ revelou que uma PAS de 135 mmHg diminuiu significativamente as taxas de eventos CV em comparação com uma PAS de 140 mmHg.

Um estudo recente denominado BPROAD (*Intensive Blood-Pressure Control in Patients with Type 2 Diabetes*) foi desenhado especificamente para avaliar os benefícios do controle intensivo da PA em pacientes com DM2.^306^ Foram avaliados 12.821 pacientes com diabetes, com idade superior a 50 anos, PAS elevada e alto risco de doenças CV. Durante o seguimento médio de 4,2 anos, os pacientes no grupo de tratamento intensivo (meta PAS < 120 mmHg) apresentaram uma redução significativa de 21% na ocorrência de eventos CV maiores em comparação ao grupo de tratamento padrão (meta PAS < 140 mmHg), o que indica um benefício do controle intensivo da PA para redução de eventos como AVC, IAM e insuficiência cardíaca crônica (ICC). Albuminúria ocorreu com menos frequência em pacientes do tratamento intensivo. A incidência de efeitos adversos sérios foi similar nos dois grupos, mas hipotensão sintomática e hiperpotassemia ocorreram mais frequentemente no grupo de tratamento intensivo. A inclusão unicamente de pacientes chineses pode limitar a generalização dos resultados para outras populações.^306^

Uma das maiores metanálises sobre redução da PA no DM2 incluiu mais de 70 mil participantes. Comparado com um controle menos rígido da PA, a alocação para um controle da PA mais rigoroso reduziu significativamente o risco de AVC em 31%, mas não reduziu o risco de IAM.^282^

Com base nas evidências atuais, esta diretriz recomenda que pessoas com DM e HA sejam tratadas para atingir metas abaixo de 130/80 mmHg.

#### 8.3.2. Tratamento da Hipertensão Arterial no Paciente com Diabetes Mellitus

O tratamento anti-hipertensivo no indivíduo com DM deve sempre ser individualizado. Os potenciais efeitos adversos da terapia anti-hipertensiva também devem ser levados em consideração. Indivíduos mais idosos, com DRC e com fragilidade apresentam maior risco de efeito adverso do controle intensivo da PA. Além disso, indivíduos com hipotensão ortostática, comorbidades significativas, limitações funcionais ou polifarmácia podem estar em alto risco de efeito adverso, e em alguns pacientes, alvos de PA menos intensivos podem ser preferidos em prol de uma melhor qualidade de vida.^307^

O tratamento da HA se baseia em MNM,^308^ já descritas no Capítulo 6 desta diretriz, e no tratamento medicamentoso. Por se tratar de um paciente de alto risco CV, o tratamento deve ser iniciado com duas classes diferentes de anti-hipertensivos, preferencialmente em um único comprimido. Diferentes ECRs recomendam medicamentos que bloqueiam o SRAA como tratamento de primeira escolha, por meio do uso de um IECA ou um BRA, em pacientes intolerantes ao IECA.^309^ Essa recomendação é apoiada pelo maior corpo de evidências na redução de desfechos micro e macrovasculares dos IECAs em pacientes com DM.^304,309^

Outros medicamentos anti-hipertensivos, como BCCs e diuréticos,^310^ compõem a base inicial de tratamento, levando-se em consideração as características e necessidades individuais do paciente. Quando a escolha for pelo diurético como segundo medicamento, deve-se dar atenção aos seus efeitos metabólicos.^311^ Os diuréticos tiazídicos similares (clortalidona [CTD] e indapamida) têm vantagens em comparação à HCTZ. A indapamida é o diurético que apresenta evidências de menores efeitos metabólicos indesejados quando comparada a CTD.^312^ Ao estabelecer a estratégia de tratamento para o controle da HA no paciente com DM, deve ser considerado o impacto da introdução ou retirada de iSGLT2 e de aGLP-1. Essas duas classes medicamentosas têm demonstrado efeitos muito discretos de redução da PA, mas de diminuição significativa de eventos CV.^296,299,313^

Em pacientes com doença renal do DM, a finerenona, um ARM não esteroidal seletivo, demonstrou proteção cardíaca e renal, além de reduções pequenas da PA.^297^

Deve ser enfatizado que educar os pacientes sobre técnicas de autogerenciamento, incluindo o monitoramento domiciliar da PA e o uso de recursos tecnológicos, contribui para adesão ao tratamento e a adoção de um estilo de vida saudável. Isso favorece o controle eficaz e sustentado da doença.^298,308^

**Table:** 

Mensagens principais sobre hipertensão arterial no paciente com diabetes mellitus
A associação de HA e DM é frequente. A HA pode contribuir para o desenvolvimento e agravamento do DM, como o contrário também pode ser verdadeiro.
Aproximadamente 80 a 90% dos pacientes com DM desenvolverão HA, aumentando o risco de complicações micro e macrovasculares, como doença renal, retinopatia, DAOP e doenças CV, o que torna essencial o controle rigoroso da PA.
MNM devem ser prescritas para todos os pacientes com HA e DM.

CV: cardiovascular; DAOP: doença arterial obstrutiva periférica; DM: diabetes mellitus; HA: hipertensão arterial; MNM: medidas não medicamentosas; PA: pressão arterial.

**Table:** 

Recomendações para pacientes com hipertensão arterial e diabetes mellitus	Forca da recomendação	Certeza da evidência
Recomenda-se, para a avaliação da PA, a utilização de medida da PA fora do consultório, pela MAPA e/ou MRPA.	FORTE	MODERADA
Recomenda-se a realização da dosagem de albuminúria em duas ou três amostras ao diagnóstico e, a seguir, de acordo com o julgamento clínico.	FORTE	ALTA
Recomenda-se a meta de PA < 130/80 mmHg para pacientes com HA e DM.	FORTE	ALTA
Recomenda-se que o início do tratamento medicamentoso deva ser imediato (ao diagnóstico), com dois medicamentos, IECA ou BRA, quando houver intolerância ao IECA, associado a BCC ou diurético tiazídico similar.	FORTE	ALTA

BCC: bloqueador dos canais de cálcio; BRA: bloqueador dos receptores da angiotensina; DM: diabetes mellitus; IECA: inibidor da enzima conversora da angiotensina; MAPA: medida ambulatorial da pressão arterial; MRPA: medida residencial da pressão arterial; PA: pressão arterial.

### 8.4. Hipertensão Arterial e Obesidade

A obesidade está intimamente relacionada à HA por meio de diversos mecanismos fisiopatológicos. Estudos epidemiológicos, como o *Framingham Heart Study*^314^ e o *Nurses’ Health Study*,^315^ demonstraram uma relação direta, contínua e quase linear entre o IMC e a PA. A obesidade, especialmente a obesidade abdominal, interfere nos sistemas endócrino e imunológico, aumentando o risco de resistência à insulina, DM, HA e DCV.^316^

As diferentes formas de distribuição da gordura corporal têm papel importante em relação à associação da HA com a obesidade. A gordura corporal pode ser classificada como central e periférica. A gordura central ou abdominal é chamada de visceral. Porém, indivíduos com uma mesma CA podem ter concentração de gordura predominantemente no subcutâneo ou intra-abdominal (visceral). A gordura visceral é diferente do ponto de vista anatômico e funcional e tem expressão genética diferente da gordura do subcutâneo.^317^ Estudos recentes têm demonstrado forte associação da gordura visceral com a HA.^318^

A obesidade visceral aumenta o risco de HA primária em 65 a 75%.^319-312^ Os mecanismos fisiopatológicos para essa associação são complexos e ainda não estão bem estabelecidos. Entre eles, podem-se destacar: a) o desbalanço simpático-vagal, com predomínio da atividade simpática sobre a parassimpática; b) a produção parácrina de angiotensinogênio, angiotensina II e aldosterona pelos adipócitos; c) a compressão renal pelo aumento da gordura intra-abdominal e retroperitoneal, que vai resultar na redução da excreção de sódio e ativação do SRAA.^319^ Além dos mecanismos citados, níveis reduzidos de peptídeos natriuréticos na obesidade e a produção de várias adipocitocinas parecem ter papel importante na patogênese da HA associada à obesidade.^322^

#### 8.4.1. Meta de Pressão Arterial em Pacientes com Obesidade

A meta pressórica recomendada para pacientes com obesidade é PA < 130/80 mmHg, à semelhança de outras situações de alto risco CV e renal.

#### 8.4.2. Tratamento da Hipertensão Arterial na Obesidade

O foco principal do tratamento da HA na pessoa com obesidade é a redução do peso para valores de IMC entre 20 e < 25 kg/m^2^ de modo estável por meio de MNM, tratamento medicamentoso e cirúrgico da obesidade, se indicado.^144^

As MNM devem ter como objetivo a redução de pelo menos 5 a 10% do peso corpóreo por meio de mudanças já descritas no Capítulo 6. Essas medidas podem resultar na perda ponderal e melhor controle da PA e de parâmetros metabólicos.^144^

Um grande número de medicamentos antiobesidade, com diferentes mecanismos de ação, já foi testado na literatura.^323^ A maioria deles apresentou resultados pouco expressivos na redução de peso e da PA. Recentemente, os aGLP1 (liraglutida, semaglutida e dulaglutida) e a tirzepatida, que é um agonista do GLP-1 e do GIP (*glucose-dependent insulinotropic polypeptide*), vêm sendo utilizados com bons resultados na redução de peso e do risco CV,^234^ mas os resultados na redução da PA são modestos.^305^

A cirurgia bariátrica tem mostrado bons resultados no controle do peso e da PA em pacientes com HA, porém, a recomendação desses procedimentos deve seguir uma boa avaliação clínica e protocolos bem estabelecidos.^282,325-329^

Em relação aos medicamentos preferenciais no tratamento da HA no paciente com obesidade, várias classes, com diferentes mecanismos de ação, são eficazes no sentido de reduzir a PA, porém podem ter efeitos metabólicos favoráveis ou desfavoráveis^330^ (Quadro 8.1).

**Quadro 8.1:** Anti-hipertensivos mais utilizados para o tratamento da hipertensão arterial em pacientes com obesidade

Classe	Ação principal	Efeitos na insulina
Diuréticos	Natriurese, ↓RVP	↓Sensibilidade à insulina
IECA ou BRA	Natriurese, ↓Aldosterona, ↓RVP	↓Resistência à insulina
BCC	↓RVP	↓Insulina plasmática
β-bloqueadores	↓Produção de renina, ↓RVP	↓Sensibilidade à insulina

BCC: bloqueadores de canais de cálcio; BRA: bloqueadores dos receptores AT1 da angiotensina II; IECA: inibidores da enzima conversora da angiotensina; RVP: resistência vascular periférica.

**Table:** 

Mensagens principais para pacientes com hipertensão arterial no paciente com obesidade
A obesidade está intimamente relacionada à HA por meio de diversos mecanismos fisiopatológicos.
Há forte associação da gordura visceral com a HA, sendo que a obesidade visceral aumenta o risco de HA primária em 65 a 75% devido a mecanismos fisiopatológicos complexos.

HA: hipertensão arterial.

**Table:** 

Recomendações para pacientes com hipertensão arterial e obesidade	Forca da recomendação	Certeza da evidência
Recomenda-se a meta de PA < 130/80 mmHg para pacientes com obesidade.	FORTE	MODERADA
Recomenda-se, além de MNM para o tratamento de pessoas com obesidade e HA, o uso de diuréticos, bloqueadores do SRAA, BCC ou BB (em condições específicas), observando-se que cada uma dessas classes de medicamentos pode interferir com parâmetros metabólicos.	FORTE	BAIXA
Recomenda-se o uso de medicamentos para diminuir peso, tais como os aGLP1 (liraglutida, semaglutida e dulaglutida) que apresentam bons resultados na redução de peso e do risco CV, mas os resultados na redução da PA são modestos.	FORTE	ALTA

aGLP1: análogos dos peptídeos semelhantes ao glucagon 1 (*Glucagon-like peptide-1*); BB: betabloqueador; BCC: bloqueador dos canais de cálcio; CV: cardiovascular; HA: hipertensão arterial; PA: pressão arterial; SRAA: sistema renina-angiotensina-aldosterona.

### 8.5. Hipertensão Arterial e COVID-19

Desde o início da pandemia de COVID-19, em 2019, observou-se que as doenças crônicas não transmissíveis (DCNT) estavam associadas a maior gravidade e letalidade da doença^331^ e, entre elas, a HA foi uma das comorbidades mais presentes, sendo consistentemente relatada como um marcador de mau prognóstico,^304,309^ dada à sua associação ao maior risco de COVID-19 grave.^96,97,332-334^

Estudos demonstraram que a HA se manteve como fator de risco para maior gravidade da COVID-19, mesmo após o ajuste para a idade;^333,335^ em pacientes com HA tratados, a PAS elevada mostrou uma relação dose-resposta com a COVID-19 grave, mesmo quando ajustada para idade e para comorbidades CV.^335^

Uma possível explicação para sua relação com maior gravidade é que a HA provoca, frequentemente, lesões subclínicas em órgãos vitais,^87^ o que pode reduzir as defesas do organismo contra infecções graves. Outra hipótese é a desregulação imunológica presente na HA.^336^ Estudos têm mostrado a associação entre COVID-19 grave e a presença de telômeros mais curtos nos cromossomos.^337^ Estes, por sua vez, também estão associados a diversas comorbidades, incluindo a HA.^338^

A HA também está presente de forma importante na COVID-19 longa, que ocorre 3 meses após o início da doença, com sintomas que duram pelo menos por 2 meses. Evidências sugerem que o curso clínico dos distúrbios relacionados à HA, como DCV e doenças renais, é afetado pela COVID longa. No entanto, são necessárias mais investigações.^339^

Em relação à vacinação contra o SARS-COV-2, esta é recomendada para pacientes com HA; nenhum sinal de aumento da PA ou alteração consistente emergiu dos ECRs que avaliaram a eficácia e segurança das vacinas.^340^

O coronavírus SARS-CoV-2 usa o receptor da enzima conversora da angiotensina 2 (ECA-2) para entrar nas células. Alguns estudos experimentais sugerem que o tratamento com IECA ou BRA pode aumentar a expressão de ECA-2, gerando a preocupante hipótese de que o tratamento com esses anti-hipertensivos poderia aumentar a infectividade e a gravidade do vírus. No entanto, uma RS demonstrou que a regulação positiva da ECA-2 pelos bloqueadores do SRAA foi um evento raro.^341^ Estudos observacionais e metanálises mostraram que o tratamento com inibidores do SRAA não afetou o risco de infecção pela COVID-19, de doença grave ou de mortalidade.^342,343^

**Table:** 

Mensagens principais sobre pacientes com hipertensão arterial em pacientes com COVID-19
Pacientes com HA apresentam maior risco de morbidade e mortalidade por COVID-19.
A vacinação para SARS-CoV-2 está indicada em pacientes com HA.
A HA está presente de forma importante na COVID-19 longa, que ocorre 3 meses após o início da doença, com sintomas que duram pelo menos por 2 meses. O curso clínico dos distúrbios relacionados à HA, como DCV e doenças renais, é afetado pela COVID longa.

HA: hipertensão arterial; DCV: doença cardiovascular.

**Table:** 

Recomendações para pacientes com hipertensão arterial e COVID-19	Forca da recomendação	Certeza da evidência
Recomenda-se que pacientes tratados com bloqueadores do SRAA para HA ou para outras condições clínicas devam manter o tratamento na presença de COVID-19.	FORTE	ALTA

HA: hipertensão arterial; SRAA: sistema renina-angiotensina-aldosterona.

### 8.6. Hipertensão Arterial, Acidente Vascular Cerebral e Déficit Cognitivo

A HA é um dos fatores de risco mais importantes para AVC e déficit cognitivo e contribui para aproximadamente 60% do risco de doença cerebrovascular crônica em todas as populações, pois tem ação direta sobre a estrutura e a microvasculatura cerebrais.^344^ O objetivo do tratamento da HA em pacientes que sofreram um AVC ou ataque isquêmico transitório (AIT) é reduzir o risco de recorrência e outras complicações CV, além de prevenir a incidência de demência ou reduzir a progressão do déficit cognitivo.^345,346^

No estudo InterStroke,^347^ os indivíduos com HA tinham duas vezes mais probabilidades de sofrer um AVC em contexto de prevenção primária. Assim, o controle da PA após um AVC é crítico para a prevenção secundária, para reduzir o risco de eventos cerebrovasculares recorrentes e déficit cognitivo.

#### 8.6.1. Meta de Pressão Arterial na Hipertensão Arterial do Acidente Vascular Cerebral na Fase Crônica

A redução da PA na fase crónica do AVC é altamente eficaz na prevenção de AVC recorrentes, protegendo contra eventos CV, preservando a função cognitiva e reduzindo a mortalidade geral. No estudo RESPECT (*Randomized Evaluation of Recurrent Stroke Comparing PFO Closure to Established Current Standard of Care Treatment*),^348^ os autores concluíram que as reduções nas taxas observadas para AVC recorrente no grupo de tratamento intensivo (PAS < 120 mmHg) em comparação com o grupo padrão (PAS < 140 mmHg) não foram significativas. Entretanto, a metanálise dos resultados, juntamente com os de três ensaios clínicos anteriores, mostra redução de 22% da recorrência de eventos cerebrovasculares com reduções mais intensivas da PA.^348^

O estudo ESPRIT (*Effects of Intensive Systolic Blood Pressure Lowering Treatment in Reducing Risk of Vascular Events*) incluiu pacientes com diabetes e pacientes com AVC prévio e confirmou que uma estratégia de terapêutica intensiva (PAS < 120 mmHg) para reduzir a PAS é capaz de prevenir eventos CV. Síncope como evento adverso sério ocorreu mais frequentemente no grupo de tratamento intensivo.^132^

Considerando os resultados dos estudos clínicos, a meta pressórica recomendada por esta diretriz é < 130/80 mmHg.

Essa mesma meta é preconizada pelas diretrizes da AHA/American Stroke Association^349,350^ e pela European Stroke Organisation (ESO) Guidelines^351^ para pacientes com AVC ou AIT para prevenção secundária de AVC. Achados similares foram encontrados por uma metanálise padrão e uma metarregressão de 10 ECRs, compreendendo 40.710 pacientes, que foi conduzida para avaliar a associação da magnitude da redução diferencial da PA e AVC em pacientes com AVC ou AIT. Após 2,8 anos, os resultados agrupados mostraram que o tratamento mais intensivo em comparação com o menos intensivo foi associado a um risco reduzido de AVC recorrente (risco absoluto, 8,4% vs. 10,1%; RR, 0,83; IC 95% 0,78–0,88). A metarregressão mostrou que a magnitude da redução diferencial da PAS e da PAD estava associada a um menor risco de AVC recorrente em pacientes com AVC ou AIT de forma log-linear. Resultados semelhantes foram encontrados na associação entre redução diferencial da PA e eventos CV maiores.^352^

Os casos de AVC agudos, isquêmicos ou hemorrágicos encontram-se discutidos no Capítulo 10.

#### 8.6.2. Tratamento da Hipertensão Arterial no Acidente Vascular Cerebral na Fase Crônica

Quanto ao tratamento, RS com metanálise de oito estudos, que incluiu 33.774 pacientes com AVC ou AIT, acompanhados por 25 meses, indicaram que um AVC subsequente ocorreu em 7,9% (AVCI em 7,4% ou acidente vascular cerebral hemorrágico [AVCH] em 0,6%) dos pacientes que tomaram qualquer tipo de medicamento para baixar a PA, em comparação com 9,7% dos pacientes que tomaram placebo (*odds ratio*, 0,79 [IC 95% 0,66–0,94]; diferença de risco absoluto, −1,9% [IC 95% −3,1% a −0,5%]). Evidências de qualidade moderada indicaram que a mortalidade ocorreu de forma semelhante em pacientes que receberam qualquer tipo de tratamento para redução da PA vs. placebo, com um risco absoluto de 7,3% e 7,9%, respectivamente (*odds ratio*, 1,01 [IC 95% 0,92–1,10]; diferença de risco absoluto, 0,1% [IC 95% −0,6% a 0,7%]).^353^

As evidências são escassas para a recomendação de medicamentos no tratamento dos pacientes com AVC ou AIT. Geralmente, ele é realizado com diuréticos tiazídicos ou similares e bloqueadores do SRAA (IECA ou BRA), visando a prevenção secundária.^349,354,355^ BCCs parecem ser eficazes, mas aumentam o risco de IC. Uma revisão da Cochrane também encontrou essas duas classes de medicamentos como aquelas mais utilizadas, em 11 ECRs que incluíram 38.742 pacientes.^356^ No entanto, ainda são necessários estudos adicionais para determinar as melhores associações, especialmente na prevenção secundária de AVC. A associação de três medicamentos está sendo testada atualmente após hemorragia intracerebral no estudo TRIDENT (*Triple Therapy Prevention of Recurrent Intracerebral Disease Events Trial* – NCT02699645), ainda sem resultados.^357^

Assim, nossa recomendação é que sejam utilizados diuréticos tiazídicos ou seus similares, como a indapamida utilizada no estudo PROGRESS (*Perindopril Protection Against Recurrent Stroke Study*), associada com um bloqueador do SRAA.^358^ Caso a meta não seja alcançada, o próximo passo seria um BCC di-hidropiridínico.

O tratamento da HA é fundamental para reduzir a carga global de demência ou déficit cognitivo em nível populacional. Metanálises recentes apoiam de forma convincente a eficácia da redução da PA na diminuição do risco de demência ou déficit cognitivo.^345,346^ Análise do estudo SPRINT-MIND (*Systolic Blood Pressure Intervention Trial – Memory and Cognition in Decreased Hypertension*) concluiu que o controle intensivo da PA não tem efeito deletério sobre a função cognitiva nos pacientes com PAD baixa.^359^ Embora o estudo SYST-EUR (*Systolic Hypertension in Europe*)^360^ tenha sugerido superioridade dos BCC de ação prolongada, não está definido se alguma classe anti-hipertensiva é preferível para prevenir demência e comprometimento cognitivo.^361-363^

O papel de mecanismos de risco concorrentes, incluindo hipotensão ortostática^364^ e variabilidade da PA,^363^ pode ser fator importante nas decisões de tratamento para pessoas com fragilidade, multimorbidade e/ou doença cerebrovascular crônica.

**Table:** 

Mensagens principais para pacientes com hipertensão arterial e acidente vascular cerebral e déficit cognitivo
A HA é o fator de risco mais importante para AVC e déficit cognitivo (60%).
As evidências são escassas para a recomendação de medicamentos anti-hipertensivos específicos nos pacientes com AVC ou AIT pregressos.
Não está definido se alguma classe de medicamentos anti-hipertensivos é preferível para prevenir demência e déficit cognitivo.

AIT: ataque isquêmico transitório; AVC: acidente vascular cerebral; HA: hipertensão arterial.

**Table:** 

Recomendações para pacientes com hipertensão arterial e acidente vascular cerebral e déficit cognitivo	Forca da recomendação	Certeza da evidência
Recomenda-se a meta de PA < 130/80 mmHg para pacientes com AVC ou AIT prévios para redução de eventos CV maiores (IAM, recorrência AVC, morte).	FORTE	ALTA
Recomenda-se a meta de PA < 130/80 mmHg para prevenção de déficit cognitivo e demência.	FRACA	MODERADA

AIT: ataque isquêmico transitório; AVC: acidente vascular cerebral; CV: cardiovascular; IAM: infarto agudo do miocárdio; PA: pressão arterial.

### 8.7. Hipertensão Arterial e Insuficiência Cardíaca com Fração de Ejeção Preservada e com Fração de Ejeção Reduzida

Mais de 90% dos pacientes que desenvolvem IC têm HA, e as condições concomitantes mais frequentes são DM2, obesidade, FA e DAC.^365^ A HA é considerada fator de risco para os dois tipos de apresentação da IC: com fração de ejeção reduzida (ICFEr) ou com fração de ejeção preservada (ICFEp). O tratamento adequado da HA reduz a incidência de IC e deve ser implementado de forma eficaz para todos os pacientes com PA ≥ 140/90 mmHg.^1,117,366^ Por outro lado, ainda não houve ECRs controlados e de longo prazo direcionados para a população com IC comparando diferentes metas de tratamento.^367,368^

Por essa razão, as recomendações de metas de PA para os pacientes com ICFEp e ICFEr são extrapoladas de estudos incluindo populações de alto risco, devendo ser < 130/80 mmHg.^367,368^

É importante ressaltar que boa parte dos pacientes com ICFEr tende a ser hipotensa, mas precisa de diversas medicações com ação anti-hipertensiva e que melhoram o prognóstico, tais como BB, IECA (ou BRA), ARM, diuréticos, sacubitril/valsartana, inibidores de SGLT2 e vasodilatadores de ação direta (hidralazina em combinação com nitrato). Sob essa perspectiva, níveis baixos de PA não devem limitar a otimização das doses terapêuticas dessas medicações supracitadas nos pacientes com ICFEr, desde que esses indivíduos os tolerem, sem apresentar eventos adversos.^368,369^

Nos pacientes com ICFEp, ainda não está claro se há uma faixa preferencial de PA a ser atingida. Um estudo observacional com 3.417 pacientes com ICFEp comparou a frequência de eventos CV e morte em pacientes com controle intensivo vs. controle padrão da PAD e concluiu que o controle intensivo da PAD aumentou o risco desses eventos adversos.^370^ Já a análise *post hoc* de um ECR avaliou mortalidade e outros desfechos negativos em 3.915 pacientes hospitalizados com ICFEp e mostrou que PAS < 120 mmHg esteve significativamente associada a desfechos negativos.^371^ Entretanto, por conta dos desenhos desses estudos, não está claro se esses achados se deveram ao tratamento anti-hipertensivo *per se* ou se os pacientes com valores de PA mais baixos tinham pior *performance* clínica e cardíaca e, consequentemente, pior prognóstico. Portanto, ainda não há evidências suficientes que respaldem qual o valor mínimo de PA que deva ser atingido nos pacientes com ICFEp.

Quanto ao tratamento da ICFEp, o tratamento medicamentoso parece ser o mesmo, independentemente da fração de ejeção, devendo ser direcionado pelos sintomas (relacionados ao grau de retenção de sódio e água), sinais (taquicardia), gravidade (classe funcional) e doenças e condições concomitantes. Todos os pacientes com IC devem receber todas as classes de medicamentos mencionadas para ICFEr, se bem toleradas (Figura 8.1).^372^

**Figura 8.1:**
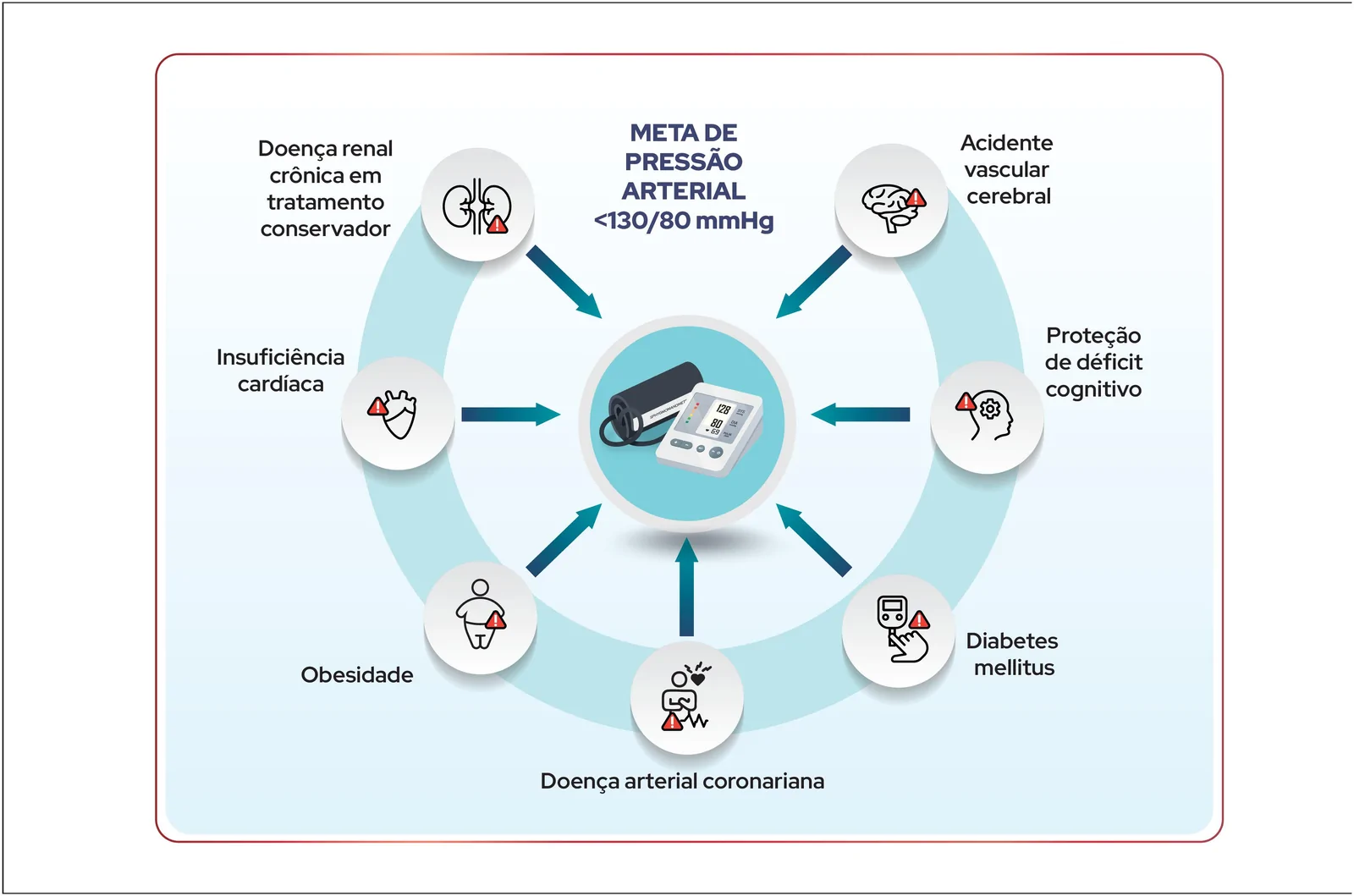
Meta de pressão arterial única para a hipertensão arterial em situações especiais de alto risco cardiovascular e renal.

**Table:** 

Mensagens principais sobre hipertensão arterial em pacientes com insuficiência cardíaca
Mais de 90% dos pacientes que desenvolvem IC têm HA, e as condições concomitantes mais frequentes são DM, obesidade, FA e DAC.
A HA é considerada fator de risco para os dois tipos de apresentação da IC (ICFEr ou ICFEp).
O tratamento adequado da HA reduz a incidência de ICFEp ou ICFEr e deve ser implementado de forma eficaz para todos os pacientes com PA ≥ 140/90 mmHg.
Níveis baixos de PA não devem limitar a otimização das doses terapêuticas dessas medicações (BB, IECA – ou BRA –, ARM, diuréticos, sacubitril/valsartana, inibidores de SGLT2 e vasodilatadores de ação direta (hidralazina em combinação com nitrato) nos pacientes com ICFEr ou ICFEP, desde que esses indivíduos as tolerem, sem apresentar efeito adverso.
O tratamento medicamentoso da HA na IC parece ser o mesmo, independentemente da fração de ejeção, devendo ser direcionado pelos sintomas predominantes.

DAC: doença arterial coronariana; DM: diabetes mellitus; FA: fibrilação atrial; HA: hipertensão arterial; IC: insuficiência cardíaca; ICFEp: insuficiência cardíaca com fração de ejeção preservada; ICFEr: insuficiência cardíaca com fração de ejeção reduzida; PA: pressão arterial.

**Table:** 

Recomendações para o paciente com hipertensão arterial e insuficiência cardíaca	Forca da recomendação	Certeza da evidência
Recomenda-se a meta de PA < 130/80 mmHg para os pacientes com HA e ICFEp e ICFEr.	FRACA	BAIXA
Recomenda-se, para pacientes com ICFEr e HA, o tratamento com medicamentos que tenham ação anti-hipertensiva e que melhorem o prognóstico: BB, IECA (ou BRA) e ARM.	FORTE	ALTA
Recomenda-se a utilização de diuréticos no tratamento de pacientes com HA e IC, se houver sinais de hipervolemia.	FORTE	ALTA
Recomenda-se o uso de sacubitril/valsartana para pacientes com ICFEr e ICFEp e HA.	FORTE	MODERADA
Recomenda-se o uso de inibidores de SGLT2 para pacientes com ICFEr e ICFEp e HA, para diminuição de hospitalização e morte CV.	FRACA	ALTA

ARM: antagonista dos receptores mineralocorticoides; BB: betabloqueador; BRA: bloqueador dos receptores da angiotensina; CV: cardiovascular; HA: hipertensão arterial; IC: insuficiência cardíaca; ICFEp: insuficiência cardíaca com fração de ejeção preservada; ICFEr: insuficiência cardíaca com fração de ejeção reduzida; IECA: inibidor da enzima conversora da angiotensina; PA: pressão arterial; SGLT2: cotransportador de sódio-glicose 2 (sodium/glucose cotransporter 2).

## 9. Hipertensão Arterial em Idosos, Crianças e Adolescentes

### 9.1. Hipertensão Arterial no Idoso

#### 9.1.1. Introdução

O Brasil está experimentando um processo envelhecimento populacional acelerado, sendo que os muito idosos (≥ 80 anos) são o estrato que mais cresce.^373^ Mais de 60% dos idosos apresentam hipertensão, com alta prevalência de HA sistólica isolada e baixa taxa de controle.^374^ A HA é um dos principais fatores de risco modificáveis para morbidade e mortalidade CV, declínio cognitivo, demência e perda de funcionalidade em idosos.^375,376^

A condição funcional do indivíduo é muito mais importante que a idade cronológica *per se* e, assim, nenhuma intervenção terapêutica deve ser negada ou retirada apenas com base na idade.^377,378^

#### 9.1.2. Mecanismos Fisiopatológicos

O enrijecimento arterial, em decorrência do envelhecimento vascular, aumenta as velocidades de propagação da onda de pulso arterial (VOP) em direção à circulação periférica (centrífuga) e das ondas reflexas que retornam ao coração (centrípeta). A superposição dessas duas ondas na fase protomesossistólica causa o aumento da PAS e o alargamento da pressão de pulso (PP = PAS – PAD), um indicador hemodinâmico útil de rigidez arterial, observado nos idosos.^379^ A rigidez arterial tem grande importância com impacto prognóstico para desfechos cardiovasculares.^380^

#### 9.1.3. Diagnóstico e Decisão Terapêutica

##### 9.1.3.1. Diagnóstico

A investigação da HA nos idosos pode ser dificultada pela presença de múltiplas comorbidades e a polifarmácia. As orientações dos Capítulos 3 e 4 desta diretriz, referentes à medida da PA, ao exame físico e avaliação laboratorial, devem ser seguidas também para essa faixa etária. No entanto, algumas características específicas do idoso devem ser levadas em conta na avaliação clínica e laboratorial desses indivíduos.

A avaliação clínica dos pacientes idosos e, especialmente, dos muito idosos difere da tradicional. Primeiramente, o médico deve reconhecer que a consulta exigirá um tempo maior, devido a vários fatores, tais como: complexidade das múltiplas condições associadas, a lentidão física e cognitiva do paciente, a presença de familiares e cuidadores, com os quais o médico deverá discutir pontos inerentes às condições clínicas e terapêuticas propostas, enquanto os muito frágeis podem necessitar de consultas adicionais, devido à exaustão do paciente.^381^

Em face da maior variabilidade pressórica e de algumas peculiaridades, a medida da PA pode resultar em valores inexatos. Os principais fatores que interferem na medida da PA em idosos são: 1) hiato auscultatório; 2) pseudo-hipertensão; e 3) variações posturais e pós-prandiais^382^ (ver Capítulo 3).

Devido à maior rigidez arterial, as variações de volume interferem de forma significativa no controle da HA. Os idosos têm menor resposta dos barorreceptores à hipotensão e, por isso, são mais propensos a hipotensão ortostática (HO) e pós-prandial (HPP). Associa-se a isso, a maior frequência de doenças neurodegenerativas. Em torno de 20% dos idosos apresentam HO, e aproximadamente 30% dos idosos têm hipotensão após as refeições. Por isso, os idosos devem ser cuidadosamente monitorados para HO e HPP. Nesses pacientes, a MAPA é particularmente útil para caracterizar os eventos, assim como os sintomas associados, frequentemente atípicos.^383,384^

A HA mal controlada e determinados anti-hipertensivos, como alfabloqueadores, podem provocar ou piorar a HO. A melhor opção para seu controle é o uso de MNM, como hidratação adequada, dieta normossódica, mudança lenta de decúbito, elevação da cabeceira e uso de meias elásticas.

Nos casos de HPP, o idoso deve também evitar refeições copiosas, grande consumo de carboidratos e de álcool e não realizar exercícios após as refeições.^385^

A monitorização da PA fora do consultório, ambulatorial (MAPA) ou residencial (MRPA) é cada vez mais valorizada e indicada no diagnóstico da HA no idoso. A automedida da PA, apesar das limitações, também deve ser considerada (ver Capítulo 3).^46^

Na solicitação de exames complementares, a investigação de causas secundárias de HA deve ser conduzida com cuidado, avaliando os riscos e os benefícios de cada procedimento (ver Capítulo 4).^386^

A avaliação clínica do idoso inclui o *status* funcional e fragilidade, além da função cognitiva, parâmetros importantes para a decisão terapêutica e escolha das medicações.

##### 9.1.3.2. Avaliação do Status Funcional e Fragilidade

Em idosos e, especialmente nos muito idosos, deve-se ter atenção especial ao *status* funcional e fragilidade. A avaliação geriátrica ampla (AGA), utilizando escalas e testes de forma sistematizada, permite identificar com precisão a condição global do idoso e montar estratégias para a abordagem terapêutica.^387,388^ Apesar de ser a forma ideal de avaliação, pode ser necessária a presença de um geriatra ou gerontólogo.

Recomenda-se o uso rotineiro de testes funcionais, como, por exemplo, o da velocidade de marcha (VM) por ser facilmente aplicado na rotina de atendimento e por ter demonstrado clara discriminação prognóstica de sobrevida.^389^ São considerados como frágeis ou em risco de fragilidade aqueles com VM < 0,8 m/s (ou incapacidade de caminhar 6 metros em menos de 8 segundos), devendo-se aprofundar a investigação.^390^ Para complementação, sugerimos utilizar a "Escala Clínica de Fragilidade" já traduzida e validada para o Brasil (Figura 9.1),^391^ a partir da *Clinical Frailty Scale* do Canadá, amplamente testada e utilizada, por sua simplicidade, confiabilidade, visão global do paciente, além de determinar prognóstico.^392^

**Figura 9.1:**
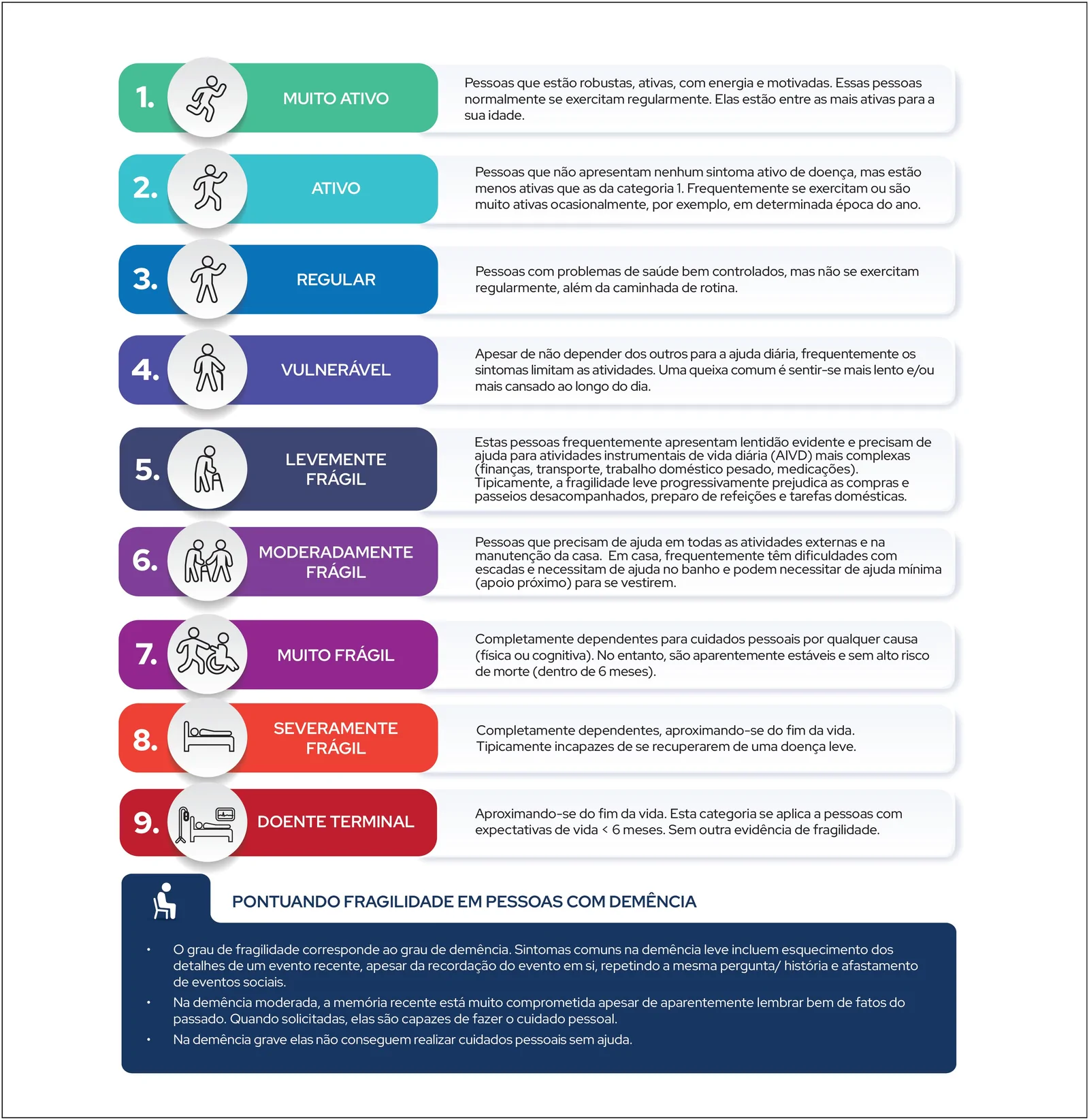
Escala clínica de fragilidade.^391^

A fragilidade está associada a maior risco de HA, doença subclínica, eventos CV e mortalidade.^393-395^ O controle adequado da HA pode potencialmente influenciar a trajetória da fragilidade. Por outro lado, graus avançados de fragilidade estão associados a menores valores de PA, menor IMC, menor massa muscular, pior cognição e maior mortalidade.^395^

##### 9.1.3.3. Avaliação da Função Cognitiva

Além de bem estabelecida como principal causa de AVC, a HA também é implicada como fator patogênico no comprometimento cognitivo, tanto de origem vascular como da doença de Alzheimer, que são as principais causas de demência no idoso, de forma mais marcante no longo prazo.^396^

Nos ECRs, a redução da PA com anti-hipertensivos mostrou redução de lesão de substância branca, do declínio cognitivo e, em menor magnitude, de demência, sendo o tratamento intensivo ainda mais eficiente.^346,397,398^ Existem limitações nos ECRs para demonstrar redução de quadros demenciais, tais como: cognição não era o desfecho primário, falta de uniformidade na definição de demência e nos testes utilizados, e curta duração dos estudos.

##### 9.1.3.4. Decisão Terapêutica

Apesar de algumas divergências entre diferentes diretrizes, todas, assim como esta diretriz, consideram fundamental a abordagem individualizada, ponderando a condição funcional, cognição, grau de fragilidade, expectativas do paciente, comorbidades, LOA e risco CV global, polifarmácia e tolerabilidade ao tratamento.^45,117,399^

Para o início do tratamento, deve-se levar em conta a fragilidade, na qual, para indivíduos hígidos, os valores para iniciar tratamento são semelhantes aos adultos (≥ 140/≥ 90 mmHg), enquanto, para os idosos frágeis, o início de tratamento deve ser individualizado de acordo com a tolerabilidade do paciente.^1,45^

#### 9.1.4. Metas Pressóricas e Tratamento

A estratégia terapêutica no idoso, especialmente naqueles com mais de 80 anos, não pode ser única. Por isso, mais importante que a idade, deve-se considerar: a presença de comorbidades, a autonomia, o *status* funcional e o grau de fragilidade para planejar o tratamento.^377,378^ Nenhuma intervenção terapêutica deve ser negada ou retirada apenas com base na idade.

##### 9.1.4.1. Metas para o Controle da Hipertensão Arterial no Paciente idoso

O estudo SHEP (*Systolic Hypertension in the Elderly Program*)^400^ com idosos com HA sistólica isolada e o estudo HYVET (*Hypertension in the Very Elderly Trial*)^401,402^ com pacientes muito idosos (idade ≥ 80 anos) demonstraram claramente o benefício da redução da PA, com diminuição significativa do risco de desfechos CV e mortalidade, mesmo em pacientes com fragilidade.

O estudo SPRINT Senior avaliou a coorte de 2.636 hipertensos com 75 anos ou mais, encontrando no braço de redução mais intensiva da PAS < 120 mmHg a redução de 34% no desfecho primário composto.^403^ Ainda no SPRINT-MIND, os pacientes alocados no braço de redução mais intensa da PAS (< 120 mmHg) tiveram progressão mais lenta no depósito de substância branca cerebral, assim como menor incidência de declínio cognitivo.^404^

Finalmente, o estudo STEP (*Semaglutide Treatment Effect in People with Obesity*) avaliou idosos com hipertensão na China com metas de redução da PAS entre 130 e 150 mmHg vs. entre 110 e 130 mmHg. Ao final de 3,3 anos de seguimento, o estudo foi interrompido pela superioridade da estratégia de redução mais intensa da PAS na redução dos desfechos CV.^133^ Metanálises recentes também demonstram que o controle mais intenso da PA nos idosos com hipertensão reduz a ocorrência de desfechos CV, sem aumento da taxa de efeitos adversos.^131,405^

Diante das evidências expostas, esta diretriz recomenda a meta < 130/80 mmHg para a grande maioria dos idosos hipertensos. Para idosos frágeis, muito idosos ou com condições que comprometam a expectativa de vida, a PA deve ser reduzida ao valor máximo tolerado.

##### 9.1.4.2. Tratamento Não Medicamentoso

Todas as MNM que se aplicam no indivíduo jovem (ver Capítulo 6) são válidas para o idoso, porém, é necessário um cuidado maior e considerar o real benefício – e potencial risco – de cada uma.

Os idosos são mais sal-sensíveis, sendo a restrição salina mais eficaz nessa faixa etária.^148^ A diminuição excessiva da ingestão de sal pode induzir hiponatremia e perda de apetite, levando a desnutrição.

As dietas ricas em potássio devem ser incentivadas,^406^ porém, deve-se ter maior cautela com o risco de hipercalemia, devido à presença frequente de DRC e do uso de medicamentos que reduzem a excreção de potássio.

Exercícios físicos, aeróbicos e resistidos são fundamentais nos idosos e devem ser orientados.^407,408^ Em idosos, especialmente nos frágeis ou sarcopênicos, a redução do peso sem EF e sem consumo adequado de proteínas pode reduzir a massa muscular e piorar a funcionalidade.

O acompanhamento por equipe multidisciplinar (ver Capítulos 6 e 13), a educação em saúde e o envolvimento de seus familiares/cuidadores são ainda mais importantes nos pacientes idosos.

##### 9.1.4.3. Tratamento Medicamentoso

Na escolha do(s) anti-hipertensivo(s) para idosos, deve-se considerar a elevada prevalência de comorbidades, contraindicações específicas, prováveis interações medicamentosas, custo, bem como a disponibilidade do medicamento e a experiência clínica. É prudente iniciar com monoterapia ou combinação em doses baixas e, se necessário, realizar aumento ou combinação gradual de anti-hipertensivos, com intervalo mínimo de 2 semanas.^409^

No Capítulo 7, estão descritos os detalhes sobre quando dar preferência ou evitar determinados anti-hipertensivos e sobre suas combinações. Destacamos, aqui, algumas peculiaridades para o idoso.

Classes de anti-hipertensivos tais como medicamentos de ação central, antagonistas da aldosterona e vasodilatadores diretos, bem como outras modalidades de tratamento invasivo no sistema simpático, devem ser vistas como exceção e não como usuais para o tratamento do idoso.

O risco de quedas em idosos pode aumentar nas primeiras semanas de tratamento com uso dos DIU e no primeiro dia com as demais classes.^410^

Em ECR, o controle da HA mostrou diminuição de eventos CV sem aumento do risco de HO ou quedas com lesão.^257,411^ Os benefícios do tratamento anti-hipertensivo foram semelhantes mesmo naqueles com HO no início do seguimento.^384^

##### 9.1.4.4. Polifarmácia e Adesão

A polifarmácia, definida pelo uso regular de cinco ou mais medicações, é mais frequente quanto maior for a idade.^411^ Ela está associada a maior chance de eventos adversos, interações medicamentosas e piora na adesão ao tratamento.^412^

Nesse sentido, recomendamos, especialmente nos idosos sob polifarmácia, a revisão periódica de cada um dos medicamentos em uso, avaliação de efeito adverso^413^ e que o tratamento anti-hipertensivo possua o menor número possível de comprimidos ao dia, com a utilização de anti-hipertensivos em combinações fixas de dose única diária, além da ênfase às MNM.

Atenção especial deve ser dada para medicamentos que possam estar contribuindo para a HO ou HPP, como diuréticos, simpatolíticos, nitratos e antidepressivos tricíclicos.

##### 9.1.4.5. Desintensificação e Desprescrição

Em diferentes situações clínicas, pode ser necessária a diminuição gradativa da dose ou até a suspensão de anti-hipertensivos, tais como: hipotensão sintomática, reações adversas, PAS muito abaixo da meta de forma persistente, detectada dentro e fora do consultório, alteração da meta pressórica para valores menos rígidos (lembrando ainda que a PA tende diminuir na idade muito avançada, resultante da diminuição progressiva da reserva orgânica e maior fragilidade) e nos cuidados paliativos no fim de vida.^414,415^

Um ponto-chave no tratamento da HA em idosos e, especialmente, em muito idosos, é o monitoramento criterioso dos efeitos adversos e da tolerabilidade, com atenção aos sinais e sintomas atípicos. A descontinuação de anti-hipertensivos parece ser segura em curto prazo, porém sem benefícios demonstrados na cognição ou funcionalidade para atividades da vida diária.^414,415^

**Table:** 

Mensagens principais sobre hipertensão arterial no paciente idoso
Mais de 60% dos pacientes idosos têm hipertensão e possuem múltiplas doenças crônicas, sendo comum a polifarmácia, com aumento de custo e risco de efeito adverso e interações.^373,374^
A polifarmácia está associada a maior mortalidade e maior taxa de hospitalização em pacientes idosos.^416,417^
A maioria dos idosos apresenta elevação isolada ou predominante da PAS.
O diagnóstico da HA no idoso requer conhecimento de suas peculiaridades, tais como o hiato auscultatório e a pseudo-hipertensão, sendo fundamental a avaliação periódica de hipotensão ortostática e a hipotensão pós-prandial.^379,380^
MNM têm bons resultados, mas exigem maior cuidado.^388^
O tratamento medicamentoso é semelhante aos de adultos mais jovens, com maior atenção à tolerabilidade e comorbidades, hipotensão ortostática ou pós-prandial e polifarmácia, mais frequentes nos idosos.^389^
A avaliação clínica deve incluir uma equipe multidisciplinar e seus cuidadores/familiares, o que contribui para aumentar as taxas de adesão e as chances de sucesso com o tratamento.
A perda de reserva orgânica nas idades avançadas está associada à diminuição gradativa da PA e implica na desintensificação e até desprescrição de um ou mais medicamentos,^390^ o que não aumenta mortalidade e pode reduzir custos.^418^
Em idosos em cuidados paliativos por doenças avançadas sem prognóstico ou fragilidade grave, o objetivo principal do tratamento é o controle de sintomas. A desprescrição de anti-hipertensivos deve ser considerada.^391^

HA: hipertensão arterial; PA: pressão arterial; PAS: pressão arterial sistólica; MNM: medidas não medicamentosas.

**Table:** 

Recomendações para abordagem da HA em pacientes idosos	Força da recomendação	Certeza da evidência
Recomenda-se que o monitoramento fora do consultório (MAPA/MRPA) deva ser realizado no paciente idoso, sempre que possível, no diagnóstico e para a avaliação do tratamento anualmente ou quando houver mudança na estratégia terapêutica.	FRACA	BAIXA
Recomenda-se que o *status* funcional, cognitivo, nutricional e social deva ser considerado para definição da capacidade intrínseca e/ou o grau de fragilidade no paciente idoso.	FRACA	BAIXA
Recomenda-se a meta de PA < 130/80 mmHg para a maioria dos pacientes idosos.	FORTE	ALTA
Recomenda-se, para idosos frágeis, muito idosos ou com condições que comprometam a expectativa de vida, que a PA seja reduzida até o menor valor tolerado.	FORTE	ALTA
Não se recomenda que BB sejam utilizados como monoterapia inicial em idosos, exceto na presença de algumas comorbidades, em que podem ter, inclusive, indicação mandatória, como IC ou nas síndromes coronarianas.	FORTE	MODERADA
Recomenda-se, para a escolha do(s) anti-hipertensivo(s) em idosos, considerar a elevada prevalência de comorbidades, as contraindicações específicas, prováveis interações medicamentosas, custo, bem como a disponibilidade do medicmaento e a experiência clínica.	FORTE	MODERADA
Recomenda-se, nos idosos sob polifarmácia, a revisão periódica de cada um dos medicamentos em uso, a avaliação de eventos adversos e que o tratamento anti-hipertensivo possua o menor número possível de comprimidos ao dia, com a utilização de anti-hipertensivos em combinações em único comprimido de dose única diária, além da ênfase às MNM.	FORTE	MODERADA

IC: insuficiência cardíaca; MAPA: monitorização. ambulatorial da pressão arterial; MRPA: medida residencial da pressão arterial; PA: pressão arterial; MNM: medidas não medicamentosas; BB: betabloqueador.

### 9.2. Hipertensão Arterial na Criança e no Adolescente

Dados de metanálise recente, incluindo 47 estudos em indivíduos ≤ 19 anos, mostram prevalência agrupada para HA de 4,0%, para HA Estágio 1 de 4,0%, para HA Estágio 2 de 0,95% e 9,67% para o grupo previamente atribuído como PA elevada, que, por corresponder à categoria de pré-hipertensão utilizada para adultos, receberá, doravante, a denominação de pré-hipertensão também em crianças e adolescentes.^419^

Em 2017, o estadiamento da PA, em crianças e adolescentes, realizado por meio de percentis de PA, de acordo com sexo, idade e percentil de estatura, foi redefinido, sendo mantido para indivíduos < 13 anos e com utilização de valores de adulto, a partir de 13 anos.^44,420^ Essas alterações racionalizaram o manejo inicial dos pacientes, com o aumento da prevalência de pré-hipertensão e HA, aumentaram a sensibilidade para detecção precoce de LOA em idade pediátrica,^421^ facilitaram a transição de cuidado do adolescente para a clínica de adultos e introduziram a confirmação diagnóstica e monitorização terapêutica da HA pediátrica pela MAPA.^420^ Mudanças no estadiamento da PA pela MAPA na idade pediátrica, utilizando a mesma base conceitual, seguiram-se em 2022.^422^ Os fatores de risco que determinam a necessidade de medida rotineira de PA em crianças < 3 anos de idade são prematuridade, muito baixo peso ao nascer, restrição de crescimento intrauterino, antecedente de internação em unidade de terapia intensiva (UTI) neonatal ou de cateterização umbilical pós-natal, cardiopatias congênitas operadas ou não, DRC, infecção do trato urinário de repetição, hematúria ou proteinúria, nefrouropatias, transplante de órgãos sólidos, doença oncológica ou transplante de medula óssea, DM, uso crônico de medicamentos com efeito de elevação de PA, doenças sistêmicas associadas a HA (neurofibromatose, esclerose tuberosa, anemia falciforme, entre outras) e evidência de hipertensão intracraniana.^420^

A medida da PA deve ser realizada pelo método auscultatório.^420^ Contudo, os dispositivos oscilométricos validados para a idade pediátrica são úteis em recém-nascidos e crianças pequenas até que possam cooperar para a adequada obtenção da medida auscultatória e como recurso de triagem para crianças maiores e adolescentes. A medida oscilométrica de PA segue a mesma orientação técnica da metodologia auscultatória.^420^ A lista de aparelhos oscilométricos validados para uso pediátrico está disponível em: https://www.stridebp.org/children-pdf/ (BHS); https://www.validatebp.org (US/AMA); https://hypertension.ca/bpdevices (CANADÁ); https://www.stridebp.org/bp-monitors (STRIDE BP), uma iniciativa conjunta da ESH, International Society of Hypertension (ISH) e World Hypertension League.

As Tabelas 9.1, 9.2, 9.3 e 9.4 apresentam, respectivamente, as definições atualizadas de PA normal, pré-hipertensão, Estágios 1 e 2 de HA na criança e no adolescente, de acordo com idade, sexo e percentil de altura, valores de PA para meninos e meninas de acordo com a idade e o percentil de estatura^420^ e definições atualizadas de PA normal, HAB, HM e HA, considerando-se a medida da PA de consultório e pela MAPA.^422,423^

**Tabela 9.1:** Definição atualizada da pressão arterial de acordo com a faixa etária

Crianças de 1 a 12 anos de idade	Crianças com idade ≥ 13 anos
PA normal: < P90 para idade, sexo e altura	PA normal: < 120/< 80 mmHg
PA elevada (pré-hipertensão): PA ≥ P90 e < 95 percentis para idade, sexo e altura ou PA 120/80 mmHg, mas < P95 (o que for menor)	Pré-hipertensão: PAS 120 a 129/< 80 mmHg
HA Estágio 1: PA ≥ P95 para idade, sexo e altura até < P95 + 12 mmHg ou PA entre 130/80 até 139/89 mmHg (o que for menor)	HA Estágio 1: PA 130/80 até 139/89 mmHg
HA Estágio 2: PA ≥ P95 + 12 mmHg para idade, sexo e altura ou PA ≥ 140/90 mmHg (o que for menor)	HA Estágio 2: PA ≥ 140/90 mmHg

HA: hipertensão arterial; P: percentil; PA: pressão arterial; PAS: pressão arterial sistólica. Adaptado de Flynn *et al*.^420^

**Tabelas 9.2:** Valores de pressão arterial para meninos, de acordo com a idade e o percentil de estatura

Idade (anos)	Porcentis de PA	Pressão Arterial Sistólica (mmHg) Percentis da Estatura ou Medida da Estatura (cm)							Pressão Arterial Diastólica (mmHg) Percentis da Estatura ou Medida da Estatura (cm)						
		5%	10%	25%	50%	75%	90%	95%	5%	10%	25%	50%	75%	90%	95%
1	Estatura (cm)	77,2	78,3	80,2	82,4	84,6	86,7	87,9	77,2	78.3	80,2	82,4	84,6	86,7	87,9
	P50	85	85	86	86	87	88	88	40	40	40	41	41	42	42
	P90	98	99	99	100	100	101	101	52	52	53	53	54	54	54
	P95	102	102	103	103	104	105	105	54	54	55	55	56	57	57
	P95 + 12 mmHg	114	114	115	115	116	117	117	66	66	67	67	68	69	69
2	Estatura (cm)	86,1	87,4	89,6	92,1	94,7	97,1	98,5	86,1	87,4	89,6	92,1	94,7	97,1	98,5
	P50	87	87	88	89	89	90	91	43	43	44	44	45	46	46
	P90	100	100	101	102	103	103	104	55	55	56	56	57	58	58
	P95	104	105	105	106	107	107	108	57	58	58	59	60	61	61
	P95 + 12 mmHg	116	117	117	118	119	119	120	69	70	70	71	72	73	73
3	Estatura (cm)	92,5	93,9	96,3	89	101,6	104,3	105,8	92,5	93,9	96,3	99	101,8	104,3	105,8
	P50	88	89	89	90	91	92	92	45	46	46	47	48	49	49
	P90	101	102	102	103	104	105	105	58	58	59	59	60	61	61
	P95	106	106	107	107	108	109	109	60	61	61	62	63	64	64
	P95 + 12 mmHg	118	118	119	119	120	121	121	72	73	73	74	75	76	76
4	Estatura (cm)	98,5	100,2	102,9	105,9	108,9	111,5	113,2	98,5	100,2	102,9	105,9	108,9	111,5	113,2
	P50	90	90	91	92	93	94	94	48	49	49	50	51	52	52
	P90	102	103	104	105	105	106	107	60	61	62	62	63	64	64
	P95	107	107	108	108	109	110	110	63	64	65	66	67	67	68
	P95 + 12 mmHg	119	119	120	120	121	122	122	75	76	77	78	79	79	80
5	Estatura (cm)	104,4	106,2	109,1	112,4	115,7	118,6	120,3	104,4	106,2	109,1	112,4	115,7	118,6	120,3
	P50	91	92	93	94	95	96	96	51	51	52	53	54	55	55
	P90	103	104	105	106	107	108	108	63	64	65	65	66	67	67
	P95	107	108	109	109	110	111	112	66	67	68	69	70	70	71
	P95 + 12 mmHg	119	120	121	121	122	123	124	78	79	80	81	82	82	83
6	Estatura (cm)	110,3	112,2	115,3	118,9	122,4	125,6	127,5	110,3	112,2	115,3	118,9	122,4	125,6	127,5
	P50	93	93	94	95	96	97	98	54	54	55	56	57	57	58
	P90	105	105	106	107	109	110	110	66	66	67	68	68	69	69
	P95	108	109	110	111	112	113	114	69	70	70	71	72	72	73
	P95 + 12 mmHg	120	121	122	123	124	125	126	81	82	82	83	84	84	85
7	Estatura (cm)	116,1	118	121,4	125,1	128,9	132,4	134,5	116,1	118	121,4	125,1	128,9	132,4	134,5
	P50	94	94	95	97	98	98	99	56	56	57	58	58	59	59
	P90	106	107	108	109	110	111	111	68	68	69	70	70	71	71
	P95	110	110	111	112	114	115	116	71	71	72	73	73	74	74
	P95 + 12 mmHg	122	122	123	124	126	127	128	83	83	84	85	85	86	86
8	Estatura (cm)	121,4	123,5	127	131	135,1	138,8	141	121,4	123,5	127	131	135,1	138,8	141
	P50	95	96	97	98	99	99	100	57	57	58	59	59	60	60
	P90	107	108	109	110	111	112	112	67	70	70	71	72	72	73
	P95	111	112	112	114	115	116	117	72	73	73	74	75	75	75
	P95 + 12 mmHg	123	124	124	126	127	128	129	84	85	85	86	87	87	87
9	Estatura (cm)	126	128,3	132,1	136,3	140,7	144,7	147,1	126	128,3	132,1	136,3	140,7	144,7	147,1
	P50	96	97	98	99	100	101	101	57	58	59	60	61	62	62
	P90	107	108	109	110	112	113	114	70	71	72	73	74	74	74
	P95	112	112	113	115	116	118	119	74	74	75	76	76	77	77
	P95 + 12 mmHg	124	124	125	127	128	130	131	86	86	87	88	88	89	89
10	Estatura (cm)	130,2	132,7	136,7	141,3	145,9	150,1	152,7	130,2	132,7	136,7	141,3	145,9	150,1	152,7
	P50	97	98	99	100	100	102	103	59	60	61	62	63	63	64
	P90	108	109	111	112	113	115	116	72	73	74	74	75	75	76
	P95	112	113	114	116	118	120	121	76	76	77	77	78	78	78
	P95 + 12 mmHg	124	125	126	128	130	132	133	88	88	89	89	90	90	90
11	Estatura (cm)	134,7	137,3	141,5	146,4	151,3	155,8	158,6	134,7	137,3	141,5	146,4	151,3	155,8	158,6
	P50	99	99	101	102	103	104	106	61	61	62	63	63	63	63
	P90	110	111	112	114	116	117	118	74	74	75	75	75	76	76
	P95	114	114	116	118	120	123	124	77	78	78	78	78	78	78
	P95 + 12 mmHg	126	126	128	130	132	135	136	89	90	90	90	90	90	90
12	Estatura (cm)	140,3	143	147,5	152,7	157,9	162,6	165,5	140,3	143	147,5	152,7	157,9	162,6	165,5
	P50	101	101	102	104	106	108	109	61	62	62	62	62	63	63
	P90	113	114	115	117	119	121	122	75	75	75	75	75	76	76
	P95	116	117	118	121	124	126	128	78	78	78	78	78	79	79
	P95 + 12 mmHg	128	129	130	133	136	138	140	90	90	90	90	90	91	91
13	Estatura (cm)	147	150	154,9	160,3	165,7	170,5	173,4	147	150	154,9	160,3	165,7	170,5	173,4
	P50	103	104	105	108	110	111	112	61	60	61	62	63	64	65
	P90	115	116	118	121	124	126	126	74	74	74	75	76	77	77
	P95	119	120	122	125	128	130	131	78	78	78	78	80	81	81
	P95 + 12 mmHg	131	132	134	137	140	142	143	90	90	90	90	92	93	93
14	Estatura (cm)	153,8	156,9	162	167,5	172,7	177,4	180,1	153,8	156,9	162	167,5	172,7	177,4	180,1
	P50	105	106	109	111	112	113	113	60	60	62	64	65	66	67
	P90	119	120	123	126	127	128	129	74	74	75	77	78	79	80
	P95	123	125	127	130	132	133	134	77	78	79	81	82	83	84
	P95 + 12 mmHg	135	137	139	142	144	145	146	89	89	91	93	94	95	96
15	Estatura (cm)	159	162	166,9	172,2	177,2	181,6	184,2	159	162	166,9	172,2	177,2	181,6	184,2
	P50	108	110	112	113	114	114	114	61	62	64	65	66	67	68
	P90	123	124	126	128	129	130	130	75	76	78	79	80	81	81
	P95	127	129	131	132	134	135	135	78	79	81	83	84	85	85
	P95 + 12 mmHg	139	141	143	144	146	147	147	90	91	93	95	96	97	97
16	Estatura (cm)	162,1	165	169,6	174,6	179,5	183,8	186,4	162,1	165	169,6	174,6	179,5	183,8	186,4
	P50	111	112	114	115	115	116	116	63	64	66	67	68	69	69
	P90	126	127	128	129	131	131	132	77	78	79	80	81	82	82
	P95	130	131	133	134	135	136	137	80	81	83	84	85	86	86
	P95 + 12 mmHg	142	143	145	146	147	148	149	92	93	95	96	97	98	98
17	Estatura (cm)	163,8	166,5	170,9	175,8	180,7	184,9	187,5	163,8	166,5	170,9	175,8	180,7	184,9	187,5
	P50	114	115	116	117	117	118	118	65	66	67	68	69	70	70
	P90	128	129	130	131	132	133	134	78	79	80	81	82	82	83
	P95	132	133	134	135	137	138	138	81	82	84	85	86	86	87
	P95 + 12 mmHg	144	145	146	147	149	150	150	93	94	96	97	98	98	99

PA: pressão arterial.

**Tabelas 9.3:** Valores de pressão arterial para meninas, de acordo com a idade e o percentil de estatura

Idade (anos)	Porcentis de PA	Pressão Arterial Sistólica (mmHg) Percentis da Estatura ou Medida da Estatura (cm)							Pressão Arterial Diastólica (mmHg) Percentis da Estatura ou Medida da Estatura (cm)						
		5%	10%	25%	50%	75%	90%	95%	5%	10%	25%	50%	75%	90%	95%
1	Estatura (cm)	75,4	76,6	78,6	80,8	83	84,9	86,1	75,4	76,6	78,6	80,8	83	84,9	86,1
	P50	84	85	86	86	87	88	88	41	42	42	43	44	45	46
	P90	98	99	99	100	101	102	102	54	55	56	56	57	58	58
	P95	101	102	102	103	104	105	105	59	59	60	60	61	62	62
	P95 + 12 mmHg	113	114	114	115	116	117	117	71	71	72	72	73	74	74
2	Estatura (cm)	84,9	86,3	88,6	91,1	93,7	96	97,4	84,3	86,3	88,6	91,1	93,7	96	97,4
	P50	84	87	88	89	90	91	91	45	46	47	48	49	50	51
	P90	101	101	102	103	104	105	106	58	58	59	60	61	62	62
	P95	104	105	106	106	107	108	109	62	63	63	64	65	66	66
	P95 + 12 mmHg	116	117	118	118	119	120	121	75	75	75	76	77	78	78
3	Estatura (cm)	91	92,4	94,9	97,6	100,5	103,1	104,6	91	92,4	94,9	97,6	100,5	103,1	104,6
	P50	88	89	89	90	91	92	93	48	48	49	50	51	53	53
	P90	102	103	104	104	105	106	107	60	61	61	62	63	64	65
	P95	106	106	107	108	109	110	110	64	65	65	66	67	68	69
	P95 + 12 mmHg	118	118	119	120	121	122	122	76	77	77	78	79	80	81
4	Estatura (cm)	97,2	98,8	101,4	104,5	107,6	110,5	112,2	97,2	98,8	101,4	104,5	107,6	110,5	112,2
	P50	89	90	91	92	93	94	94	50	51	51	53	54	55	55
	P90	103	104	105	106	107	108	108	62	63	64	65	66	67	67
	P95	107	108	109	109	110	111	112	66	67	68	69	70	70	71
	P95 + 12 mmHg	119	120	121	121	122	123	124	78	79	80	81	82	82	83
5	Estatura (cm)	103,6	105,3	108,2	111,5	114,9	118,1	120	103,6	105,3	108,2	111,5	114,9	118,1	120
	P50	90	91	92	93	94	95	96	52	52	53	55	56	57	57
	P90	104	105	106	107	108	109	110	64	65	66	67	68	69	70
	P95	108	109	109	110	111	112	113	68	69	70	71	72	73	73
	P95 + 12 mmHg	120	121	121	122	123	124	125	80	81	82	83	84	85	85
6	Estatura (cm)	110	111,8	114	118,4	122,1	125,6	127,7	110	111,8	114,9	118,4	122,1	125,6	127,7
	P50	92	92	93	94	96	97	97	54	54	55	56	57	58	59
	P90	105	106	107	108	109	110	111	67	67	68	69	70	71	71
	P95	109	109	110	111	112	113	114	70	71	72	72	73	74	74
	P95 + 12 mmHg	121	121	122	123	124	125	126	82	83	84	84	85	86	86
7	Estatura (cm)	115,9	117,8	121,1	124,9	128,8	132,5	134,7	115,9	117,8	121,1	124,9	128,8	132,5	134,7
	P50	92	93	94	95	97	98	99	55	54	56	57	58	59	60
	P90	106	106	107	109	110	111	112	68	68	69	70	71	72	72
	P95	109	110	111	112	113	114	115	72	72	73	73	74	74	75
	P95 + 12 mmHg	121	122	123	124	125	126	127	84	84	85	85	86	86	87
8	Estatura (cm)	121	123	126,5	130,6	134,7	138,8	140,9	121	123	126,5	130,6	134,7	138,5	140,9
	P50	93	94	95	97	98	99	100	56	56	57	59	60	61	61
	P90	107	107	108	110	111	112	113	69	70	71	72	72	73	73
	P95	110	111	112	113	115	116	117	72	73	74	74	75	75	75
	P95 + 12 mmHg	122	123	124	125	127	128	129	84	85	86	86	87	87	87
9	Estatura (cm)	125,3	127,6	131,3	135,6	140,1	144,1	146,6	125,3	127,6	131,3	135,6	140,1	144,1	146,6
	P50	95	95	97	98	99	100	101	57	58	59	60	60	61	61
	P90	108	108	109	111	112	113	114	71	71	72	73	73	73	73
	P95	112	112	113	114	116	117	118	74	74	75	75	75	75	75
	P95 + 12 mmHg	124	124	125	126	128	129	130	86	86	87	87	87	87	87
10	Estatura (cm)	129,7	132,2	136,3	141	145,8	150,2	152,8	129,7	132,2	136,3	141	145,8	150,2	152,8
	P50	96	97	98	99	101	102	103	58	59	59	60	61	61	61
	P90	109	110	111	112	113	115	116	72	73	73	73	73	73	73
	P95	113	114	114	116	117	119	120	75	75	76	76	76	76	76
	P95 + 12 mmHg	125	126	126	128	129	131	132	87	87	88	88	88	88	88
11	Estatura (cm)	135,6	138,3	142,8	147,8	152,8	157,3	160	135,6	138,3	142,8	147,8	152,8	157,3	160
	P50	98	99	101	102	104	105	106	60	60	60	61	62	63	64
	P90	111	112	113	114	116	118	120	74	74	74	74	74	75	75
	P95	115	116	117	118	120	123	124	76	77	77	77	77	77	77
	P95 + 12 mmHg	127	128	129	130	132	135	136	88	89	89	89	89	89	89
12	Estatura (cm)	142,8	145,5	149,9	154,8	159,6	163,8	166,4	142,8	145,5	149,9	154,8	159,6	163,8	166,4
	P50	102	102	104	105	107	108	108	61	61	61	62	64	65	65
	P90	114	115	116	118	120	122	122	75	75	75	75	76	76	76
	P95	118	119	120	122	124	125	126	78	78	78	78	79	79	79
	P95 + 12 mmHg	130	131	132	134	136	137	138	90	90	90	90	91	91	91
13	Estatura (cm)	148,1	150,6	154,7	159,2	163,7	167,8	170,2	148,1	150,6	154,7	159,2	163,7	167,8	170,2
	P50	104	105	106	107	108	108	109	62	62	63	64	65	65	65
	P90	116	117	119	121	122	123	123	75	75	75	76	76	76	76
	P95	121	122	123	124	126	126	127	79	79	79	79	80	80	81
	P95 + 12 mmHg	133	134	135	136	138	138	139	91	91	91	91	92	92	93
14	Estatura (cm)	150,6	153	156,9	161,3	165,7	169,7	172,1	150,6	153	156,9	161,3	165,7	169,7	172,1
	P50	105	106	107	108	109	109	109	63	63	64	65	66	66	66
	P90	118	118	120	122	123	123	123	76	76	76	76	77	77	77
	P95	123	123	124	125	126	127	127	80	80	80	80	81	81	82
	P95 + 12 mmHg	135	135	136	137	139	139	139	92	92	92	92	93	93	94
15	Estatura (cm)	151,7	154	157,9	162,3	166,7	170,6	173	151,7	154	157,9	162,3	166,7	170,6	173
	P50	105	106	107	108	109	109	109	64	64	64	65	66	67	67
	P90	118	119	121	122	123	123	124	76	76	76	77	77	78	78
	P95	124	124	125	126	127	127	128	80	80	80	81	82	82	82
	P95 + 12 mmHg	136	136	137	138	139	139	140	92	92	92	93	94	94	94
16	Estatura (cm)	152,1	154,5	158,4	162,8	167,1	171,1	173,4	152,1	154,5	158,4	162,8	167,1	171,1	173,4
	P50	106	107	108	109	109	110	110	64	64	65	66	66	67	67
	P90	119	120	122	123	124	124	124	76	76	76	77	78	78	78
	P95	124	125	125	127	127	128	128	80	80	80	81	82	82	82
	P95 + 12 mmHg	136	137	137	139	139	140	140	92	92	92	93	94	94	94
17	Estatura (cm)	152,4	154,7	158,7	163	167,4	171,3	173,7	152,4	154,7	158,7	163	167,4	171,3	173,7
	P50	107	108	109	110	110	110	111	64	64	65	66	66	66	67
	P90	120	121	123	124	124	125	125	76	76	77	77	78	78	78
	P95	125	125	126	127	128	128	128	80	80	80	81	82	82	82
	P95 + 12 mmHg	137	137	138	139	140	140	140	92	92	92	93	94	94	94

PA: pressão arterial. Adaptado de Flynn et al.^420^

**Tabela 9.4:** Valores de pressão arterial no consultório e na medida ambulatorial da pressão arterial para caracterização dos fenótipos de hipertensão arterial em crianças e adolescentes^422^

	Pressão arterial sistólica e pressão arterial diastólica de consultório		Monitorização ambulatorial da pressão arterial	
	< 13 anos	≥ 13 anos	< 13 anos	≥ 13 anos
Pressão arterial normal	< percentil 95	< 130/80	< percentil 95 OU valores de corte para adolescentes*	< 125/75 mmHg 24 h E< 130/80 mmHg vigília E< 110/65 mmHg sono
Hipertensão do avental branco	≥ percentil 95	≥ 130/80		
Hipertensão mascarada	< percentil 95	< 130/80	≥ percentil 95 OU valores de corte para adolescentes*	≥ 125/75 mmHg 24 h OU ≥ 130/80 mmHg vigília OU≥ 110/65 mmHg sono
Hipertensão ambulatorial	≥ percentil 95	≥ 130/80		

* 24 h, vigília e sono

A PA do neonato aumenta com o peso ao nascer, a idade gestacional e a idade pós-natal. Pequenos estudos observacionais metodologicamente distintos originaram propostas incongruentes de normatização da PA no recém-nascido.^424^ Sugere-se a utilização dos valores estimados de PA após duas semanas de vida em neonatos de 26 a 44 semanas de idade pós-concepção, demonstrados na Tabela 9.5^425^ e dos valores de PA para o primeiro ano de vida apresentados nas Tabelas 9.2 e 9.3.^426^

**Tabela 9.5:** Valores estimados de pressão arterial sistólica, diastólica e média (em mmHg) após 2 semanas de vida em neonatos de 26 a 44 semanas de idade pós-concepção425

Idade pós-concepção	Percentil 50	Percentil 95	Percentil 99
**44 semanas**			
PAS	88	105	110
PAD	50	68	73
PAM	63	80	85
**42 semanas**			
PAS	85	98	102
PAD	50	65	70
PAM	62	76	81
**40 semanas**			
PAS	80	95	100
PAD	50	65	70
PAM	60	75	80
**38 semanas**			
PAS	77	92	97
PAD	50	65	70
PAM	59	74	79
**36 semanas**			
PAS	72	87	92
PAD	50	65	70
PAM	57	72	71
**34 semanas**			
PAS	70	85	90
PAD	40	55	60
PAM	50	65	70
**32 semanas**			
PAS	68	83	88
PAD	40	55	60
PAM	50	65	70
**30 semanas**			
PAS	65	80	85
PAD	40	55	60
PAM	48	65	68
**28 semanas**			
PAS	60	75	80
PAD	38	50	54
PAM	45	58	63
**26 semanas**			
PAS	55	72	77
PAD	30	50	56
PAM	38	57	63

PAD: pressão arterial diastólica; PAM: pressão arterial média; PAS: pressão arterial sistólica. Adaptado de Dionne *et al*.^425^

O diagnóstico de HA pediátrica baseia-se na confirmação de valores de PA ≥ percentil 95 em três atendimentos diferentes utilizando metodologia auscultatória.^398^

Anamnese e exame clínico detalhados, abrangendo o período perinatal e ambiente biopsicossocial são fundamentais para a indicação de exames complementares e confirmação etiológica da HA, que pode ser primária ou secundária. A HA secundária é mais provável nos mais jovens e naqueles com valores mais elevados de PA. A HA primária prevalece no adolescente e nos casos de sobrepeso/obesidade.^420^

Avaliação extensa para HA secundária é dispensável em pacientes ≥ 6 anos com sobrepeso ou obesidade, histórico familiar positivo de HA e/ou com anamnese e exame clínico sem achados de risco.^420^ A pesquisa de proteinúria é obrigatória para casos de DRC e HA.^420^

A MAPA está indicada para a confirmação de HA em crianças e adolescentes com medidas de consultório compatíveis com pré-hipertensão por pelo menos 1 ano ou com valores de PA compatíveis com HA Estágio 1 em três consultas ambulatoriais.^420^

O procedimento deve seguir técnicas padronizadas, utilizar monitores validados para uso pediátrico e dados normativos pediátricos.^420,422^

A HA pediátrica não controlada, mesmo nos assintomáticos, pode promover aumento da espessura da camada íntima-média da carótida, redução da distensibilidade arterial, estreitamento arteriolar da retina^427^ e HVE, presente em até 40% dos casos de HA pediátrica no momento do diagnóstico inicial.^428^ Coortes prospectivas de longa duração demonstram associação de fatores de risco CV na criança com LOA no adulto. A redução do peso e do consumo de álcool, o aumento no consumo de vegetais e o estímulo à prática de AF com redução do comportamento sedentário entre a infância e a idade adulta, conjuntamente, podem contribuir para a resolução da HA na transição infância-idade adulta e para a minimização dos efeitos adversos cardiometabólicos nos indivíduos adultos não obesos,^429-432^ sugerindo que a HA pediátrica deve ser considerada uma manifestação precoce de DCV.

O tratamento da HA pediátrica visa o controle da PA, a minimização do risco de LOA, a redução do risco de HA no adulto e consequente dano CV. Seu planejamento depende da etiologia da HA, do risco CV, associado ou não a outras doenças de base e à presença de LOA.^420^ O alvo terapêutico é a redução da PAS e PAD para < percentil 90 e < 130/80 mmHg em adolescentes ≥ 13 anos. Na DRC, visa-se a redução < percentil 50 da média de PA de 24 horas na MAPA.^420^

Inicia-se terapêutica não medicamentosa para todos os pacientes com PA ≥ percentil 90 ou ≥ 130/80 mmHg (≥ 13 anos de idade)^420^ visando redução de peso, EF regular, intervenção dietética e controle de estresse. A associação entre essas quatro medidas tem efeito potencializado.^433^ Recomenda-se a dieta DASH, especialmente na HA associada à obesidade,^420,434^ além de medidas para controle de estresse.^420^

A terapêutica medicamentosa deve ser iniciada com IECA, BRA, BCC de ação prolongada ou um diurético tiazídico para casos com HA sintomática, presença de LOA, HA Estágio 2 sem causa modificável aparente e HA persistente não responsiva à MNM se a PA for ≥ percentil 95 ou ≥ 130/80 mmHg em adolescentes ≥ 13 anos de idade.^420^ Em casos de HA secundária a DRC ou DM e/ou proteinúria, recomenda-se iniciar IECA ou BRA.

O ecocardiograma, utilizando critérios definidos para a faixa etária,^398^ é sugerido previamente ao início do tratamento medicamentoso, podendo ser repetido em 6 a 12 meses, a depender da necessidade.^420^

Inicia-se o tratamento medicamentoso com agente anti-hipertensivo único, em dose baixa, com aumento a cada 2 a 4 semanas até o alvo terapêutico. Na HA secundária, sugere-se escolha do anti-hipertensivo em consonância com o princípio fisiopatológico e as comorbidades de cada caso.^420^ A frequência do acompanhamento clínico dependerá das peculiaridades de cada caso. A solicitação da MAPA no seguimento está indicada quando não houver controle da HA ou nos casos de risco de HM, como no pós-operatório tardio de coarctação de aorta. Na DRC, o controle anual da PA pela MAPA é obrigatório para afastar HM. A MRPA pode ser utilizada como adjuvante na avaliação do controle pressórico.^420^

**Table:** 

Mensagens principais sobre hipertensão arterial em crianças e adolescentes
A prevalência global agrupada de HA em indivíduos ≤ 19 anos é de 4,0%.
A HA secundária é mais provável em crianças mais jovens e naquelas com valores mais elevados de PA. A HA primária é mais prevalente no adolescente e nos casos de sobrepeso/obesidade.
A redução do peso e do consumo de álcool, o aumento no consumo de vegetais e o estímulo à prática de AF com redução do comportamento sedentário entre a infância e a idade adulta, conjuntamente, podem contribuir para a resolução da HA na transição infância-idade adulta e para a minimização dos efeitos adversos cardiometabólicos nos indivíduos adultos não obesos.

HA: hipertensão arterial; PA: pressão arterial; AF: atividade física.

**Table:** 

Recomendações para abordagem da HA em crianças e adolescentes	Força da recomendação	Certeza da evidência
Recomenda-se que a PA da criança seja medida anualmente a partir de 3 anos de idade.	FORTE	BAIXA
Recomenda-se a investigação de suspeita de HAB e HA secundária nas crianças nascidas prematuras e naquelas com DRC, DM, AOS, obesidade, pós-operatório de coarctação de aorta, TX de órgãos sólidos e HAR.	FORTE	MODERADA
Recomenda-se que o diagnóstico de HA na criança ou adolescente até 13 anos seja baseado na confirmação de valores de PA ≥ percentil 95 de acordo com o percentil de altura e quando ≥ 13 anos deve ser baseado na confirmação de valores de PA ≥ 130/80 mmHg em três atendimentos diferentes utilizando metodologia auscultatória.	FORTE	BAIXA
Recomenda-se a MAPA para a confirmação de HA em crianças e adolescentes com medidas de consultório compatíveis com pré-hipertensão por pelo menos 1 ano ou com valores de PA compatíveis com HA Estágio 1 em três consultas ambulatoriais.	FORTE	MODERADA
Recomenda-se, em crianças com DRC, a MAPA para o controle anual da PA para afastar HM.	FRACA	BAIXA
Recomenda-se a pesquisa de proteinúria nos casos de crianças com DRC e HA.	FORTE	MODERADA
Recomenda-se alvo terapêutico de redução da PAS e PAD para < percentil 90 em crianças e < 130/80 mmHg em adolescentes ≥ 13 anos.	FRACA	BAIXA
Recomenda-se a dieta DASH para crianças e adolescentes, especialmente na HA associada à obesidade, além de medidas para controle de estresse.	NEUTRA	BAIXA
Recomenda-se início de terapêutica medicamentosa em crianças e adolescentes com IECA, BRA, BCC de ação prolongada, ou um diurético tiazídico para casos com HA sintomática, presença de LOA, HA Estágio 2 sem causa modificável aparente ou HA persistente não responsiva à MNM se a PA for ≥ percentil 95 em crianças ou ≥ 130/80 mmHg em adolescentes ≥ 13 anos de idade.	FORTE	BAIXA
Recomenda-se iniciar IECA ou BRA em crianças e adolescentes com HA secundária a DRC ou DM, e/ou proteinúria.	FORTE	MODERADA

AOS: apneia obstrutiva do sono; BCC: bloqueador dos canais de cálcio; BRA: bloqueador dos receptores da angiotensina; DASH: Dietary Approaches to Stop Hypertension; DM: diabetes mellitus; DRC: doença renal crônica; HA: hipertensão arterial; HAB: hipertensão do avental branco; HAR: hipertensão arterial resistente; IECA: inibidores da enzima conversora de angiotensina; LOA: lesão de órgãos-alvo; MAPA: medida ambulatorial da pressão arterial; MNM: medidas não medicamentosas; PA: pressão arterial; PAD: pressão arterial diastólica; PAS: pressão arterial sistólica; TX: transplante.

## 10. Hipertensão Arterial em Mulheres

### 10.1. Hipertensão Arterial em Mulheres Jovens e na Menopausa

#### 10.1.1. Prevalência

Apesar de prevalência similar,^7,45^ o comportamento da PAS e PAD durante a vida em homens e mulheres é diferente, pois o aumento da PA com a idade progride mais rapidamente em mulheres e se inicia desde a fase adulta jovem.^435^ Essas diferenças entre homens e mulheres na evolução da PA ao longo da vida e no desenvolvimento da HA relacionada à idade podem ser explicadas por diferenças nos mecanismos reguladores da PA nessas pessoas, provavelmente devido a fatores hormonais específicos de gênero.^45^ A partir dos 60 anos de idade, a PA entre as mulheres costuma ser mais elevada, e a prevalência de HA é maior.^1^ A frequência aumenta com idade, sendo 61,5% em homens e 68,0% em mulheres na faixa etária de 65 anos ou mais.^45^

#### 10.1.2. Fatores Associados a Hipertensão Arterial em Mulheres

Mulheres apresentam fatores de risco específicos associados com HA, incluindo aqueles relacionados a determinantes sociais e fatores biológicos.^436^ Destacam-se, entre esses fatores, a menopausa, a síndrome do ovário policístico, o uso de contraceptivos orais e doenças hipertensivas específicas da gestação. Os fatores convencionais também têm impactos diferentes nas mulheres, como o envelhecimento, obesidade, sedentarismo, além de outros emergentes, como doenças autoimunes, estresse crônico e fatores ambientais e psicossociais. Em mulheres jovens, algumas causas secundárias, tais como estenose de artéria renal por displasia fibromuscular, são mais prevalentes do que em homens.^437^

##### 10.1.2.1. Mulheres Jovens e Anticoncepcionais

A associação entre o uso de anticoncepcional hormonal combinado oral (AHCO) e HA tem sido estudada, e uma metanálise contendo 24 estudos com 270.284 participantes mostrou RR de 1,47 (IC 95%, 1,25–1,73) de hipertensão para as usuárias com mais longa X mais curta duração de uso de AHCO e uma relação dose-resposta linear de aumento do risco de 13% (RR 1,13; IC 95% 1,03–1,25) de novos casos de HA para cada cinco anos de uso dos AHCOs.^438^

Os AHCOs contendo estrogênio e progesterona são mais propensos a induzir hipertensão, enquanto os contraceptivos que contêm apenas progestinas e formulações com baixa dosagem estrogênica são associados com menores aumentos da PA.^436,439,440^ O uso de drosperinona, uma progestina com propriedades mineralocorticoides, combinada a etinil-estradiol, promoveu redução da PAS (1-4 mmHg), mas aumentou o risco de tromboembolismo venoso.^436^

Recomenda-se a monitorização da PA em mulheres jovens antes da prescrição de AHCO e, após o início do uso, reforçar a importância de medidas regulares da PA (a cada 6 meses), incluindo medidas domiciliares e o controle dos demais fatores de risco CV.^436^ Quando houver elevação persistente da PAS ≥ 160 mmHg e/ou PAD ≥ 100 mmHg, considerar outros métodos contraceptivos ou a descontinuação dos AHCOs.^436^

A investigação de causas secundárias de HA em mulheres jovens é muito importante, destacando-se entre as principais: obesidade, síndrome do ovário policístico, apneia obstrutiva do sono, coarctação de aorta, doenças autoimunes, doenças endócrinas e nefropatias primárias.^437^

Para o tratamento da HA, IECA ou BRA não devem ser prescritos em mulheres com potencial para engravidar devido aos efeitos teratogênicos das classes no caso de uma gestação, exceto em indicações compulsórias (por exemplo, IC) e com medidas de contracepção.^436^ Preferencialmente, nessa fase, iniciar tratamento com BCCs ou BBs, preferencialmente aqueles com propriedades vasodilatadoras associadas.^1^

##### 10.1.2.2. Hipertensão, Função Sexual e Fertilidade na Mulher

A HA afeta a capacidade reprodutiva das mulheres, mas os mecanismos pelos quais a HA afeta a saúde e a função reprodutiva ainda precisam ser elucidados. A HA pode afetar a saúde dos vasos sanguíneos no útero e ovários, prejudicando a fertilidade.^441^

Além disso, mulheres com HA podem apresentar disfunção sexual devido a alterações na vasculatura clitoriana e vaginal, redução do fluxo sanguíneo na região pélvica e afinamento da parede vaginal e da musculatura lisa do clitóris, resultando em secura vaginal.^441^ Outras complicações subsequentes incluem dor durante a relação sexual, falta de orgasmo, efeitos adversos da fase de excitação e relutância sexual na mulher hipertensa.^441^ A HA pode levar à fibrose do clitóris e da parede vaginal devido a níveis mais baixos de óxido nítrico e à redução do fluxo sanguíneo na região pélvica.^441^

##### 10.1.2.3. Menopausa

A menopausa está associada a uma diminuição do estradiol e a uma alteração na relação estrogênio/androgênio.^439^ Essas alterações hormonais podem induzir ao aumento de peso, com consequente aumento da atividade simpática e do SRAA, culminando com aumento do estresse oxidativo, disfunção endotelial e consequente diminuição do óxido nítrico e aumento da endotelina.^435,439,442^ Em conjunto, esses mecanismos aumentam a sensibilidade ao sal e produzem vasoconstrição renal e HA.^442^ Além disso, em mulheres na pós-menopausa, ocorre maior rigidez aórtica devido ao aumento de células de músculo liso, colágeno e dos níveis de moléculas vasoconstritoras na parede arterial, consequente à falta do efeito protetor do estrogênio, o que pode causar HA sistólica isolada.^439^

Mulheres com HA na pós-menopausa apresentam uma transição cardiometabólica com maior incidência de HVE e maior risco de desenvolver disfunção diastólica em comparação com as mulheres adultas jovens.^439^

Para o tratamento medicamentoso durante e após a menopausa na mulher, recomenda-se iniciar com a combinação de inibidor do SRAA com BCC ou diurético tiazídico, sendo esses últimos potencialmente mais úteis por prevenir osteoporose nas mulheres.^436^

Quando indicada a terapia hormonal da menopausa, será necessário monitorar a PA desde o início e, caso não haja controle adequado, essa terapia deve ser interrompida. Mulheres com HA controlada e sintomas vasomotores (SVM) moderados a intensos podem utilizar terapia hormonal da menopausa por qualquer via, sendo preferível, porém, a terapia estrogênica transdérmica (por meio de gel ou adesivos) na presença de obesidade, dislipidemia, DM e síndrome metabólica.^439^

##### 10.1.2.4. Síndrome dos Ovários Policísticos

A síndrome dos ovários policísticos (SOP), uma condição endócrina e metabólica prevalente em mulheres em idade reprodutiva, é um distúrbio heterogêneo que está associado ao aumento do risco CV.^437,443^ Estudos anteriores demonstraram que quase todos os fatores de risco CV, incluindo HA, obesidade, resistência à insulina, DM, dislipidemia aterogênica e síndrome metabólica, são prevalentes em pacientes com SOP.^443^ Esses fatores combinados presentes em mulheres jovens com SOP aumentam o risco precoce de eventos CV. O excesso de andrógeno na SOP está claramente associado com o aumento dos distúrbios cardiometabólicos nessas mulheres.^437^ Os estudos que avaliam a associação de SOP com HA têm resultados conflitantes, mas metanálises recentes mostram forte associação de SOP com HA em mulheres jovens.^443,444^

Em RS que incluiu dados de três estudos retrospectivos e dois estudos transversais, observou-se que as mulheres com SOP tinham maior risco de desenvolver distúrbios metabólicos, incluindo HA, obesidade, dislipidemia, resistência à insulina e DM.^444^ A hiperandrogenemia foi associada com PA elevada independentemente da idade da mulher, da resistência à insulina, obesidade e dislipidemia. Em outra metanálise, a razão de risco de HA em mulheres jovens com SOP foi 1,7, enquanto, após a menopausa, a presença de SOP não aumentou o risco de HA.^443^

#### 10.1.3. Repercussões em Órgãos-Alvo e Comorbidades em Mulheres

As mulheres têm dimensões menores da raiz da aorta do que os homens, mesmo após o ajuste para o tamanho do corpo, e maior rigidez arterial tem sido identificada em mulheres, particularmente na aorta ascendente. A rigidez arterial é menos modificável pela terapia anti-hipertensiva em mulheres do que em homens.^442^

Em relação ao coração, a HVE hipertensiva é mais prevalente e menos modificável pelo tratamento anti-hipertensivo em mulheres do que em homens. As mulheres com HA desenvolvem mais frequentemente FA e IC com fração de ejeção preservada.^442^ Em relação ao rim, a prevalência global de DRC é maior em mulheres (51,7%) do que em homens, de acordo com o estudo NHANES (*National Health and Nutrition Examination Survey*), sendo 20 vezes maior que em homens em idades superiores a 65 anos.^445^ No entanto, em recente metanálise, incluindo mais de 2 milhões de indivíduos, a incidência de DRC por HA foi menor nas mulheres do que em homens.^446^

Recomenda-se que as mulheres com HA devam realizar monitoramento da função renal, incluindo albuminúria a cada ano, quando possível.^436^ Além disso, mulheres hipertensas em uso de terapias de reposição hormonal apresentam aumento significativo da albuminúria.

Mulheres com DRC e HA são mais vulneráveis ao efeito da sobrecarga hídrica no ventrículo esquerdo, por isso, a meta de peso seco deve ser mantida rigorosamente.^436^

A HA é o fator de risco mais importante para IAM, IC com fração de ejeção preservada, AVC, declínio cognitivo e doença arterial periférica em mulheres. O risco de DCV se inicia em níveis ≈ 10 mmHg mais baixos de PAS braquial em mulheres em comparação com homens.^447^

#### 10.1.4. Tratamento da Hipertensão nas Mulheres

Em primeiro lugar, as MNM são altamente recomendadas para o controle da PA em mulheres.^442^ A HA em mulh*e*res na pós-menopausa tende a ser sensível ao sal, portanto, a redução da ingestão de sódio pode ter benefícios na diminuição da PA.

No estudo DASH, a restrição dietética de sódio induziu a reduções da PA significativas apenas em mulheres.^448,449^ Quanto à AF, uma metanálise de 93 ensaios que avaliou o impacto de exercício aeróbico sobre a PA evidenciou que o exercício induziu uma redução maior da PA em participantes do sexo masculino que em mulheres.^450^

Em relação ao tratamento medicamentoso, a absorção, distribuição, metabolismo e excreção de medicamentos anti-hipertensivos são diferentes entre mulheres e homens, provavelmente devido à influência dos hormônios sexuais nos transportadores de absorção (glicoproteína P), volume de distribuição, atividade do citocromo P450 e depuração renal.^440^ No entanto, essas diferenças não justificam dosagens específicas para homens e mulheres, mas podem explicar a maior frequência de efeitos adversos dos anti-hipertensivos observadas nas mulheres, em especial na menopausa,^439^ os mais frequentes são: tosse induzida por IECA, edema de tornozelo com BCC, além de hipocalemia e hiponatremia com diuréticos. Esses efeitos podem explicar menor adesão das mulheres na menopausa ao tratamento da HA.^439,451^

Além disso, mulheres atingem pior controle da PA do que homens, e em um estudo realizado na Suécia, mulheres com HA apresentaram PA mais elevada, menos tratamento anti-hipertensivo e pior controle quando comparadas aos homens, e o sexo feminino foi um preditor de terapia anti-hipertensiva menos intensa em relação ao masculino.^452^

As mulheres e os aspectos específicos de gênero na HA são pouco estudados, e as evidências existentes são mal traduzidas em diretrizes clínicas.^453^ Algumas metanálises mostraram que reduções da PA com o tratamento são tão importantes (em termos de benefícios) em mulheres quanto em homens, e as reduções de eventos ocorreram de forma similar em homens e mulheres.^454,455^ Uma metanálise incluindo 31 ensaios randomizados e um total de 103.268 homens e 87.349 mulheres encontrou reduções comparáveis na PA e na incidência de eventos CV em ambos os sexos para tratamentos baseados em IECA, BRA, BCC, diuréticos ou BB.^454^ Da mesma forma, outra metanálise baseada em 40 ensaios clínicos e 152.379 pacientes documentou que nenhuma classe de medicamento (IECA, BRA, BCC ou BB) foi significativamente melhor do que diuréticos tiazídicos como terapia de primeira linha para qualquer desfecho (mortalidade por todas as causas, mortalidade CV, IC ou AVC) em mulheres ou homens analisados separadamente.^455^

As metas de PA recomendadas são iguais para mulheres e homens, e as classes de anti-hipertensivos podem ser utilizadas igualmente para o tratamento da HA em mulheres e homens.^453^

### 10.2. Hipertensão na Gestação

A HA na gestação é uma das principais causas de morbidade e mortalidade materna e perinatal em todo o mundo, com predominância em países pobres da América Latina e Caribe, incluindo o Brasil.^456-458^ Considera-se HA na gestação quando a gestante apresenta PAS ≥ 140 mmHg e/ou PAD ≥ 90 mmHg, com base em uma média de pelo menos duas medidas com intervalo de 4 horas ou em duas visitas ambulatoriais diferentes.^456^ Deve-se ressaltar que a medida da PA na gestação deve ser realizada na posição sentada e utilizando o quinto som de Korotkoff para obtenção da PAD na medida de PA auscultatória.

#### 10.2.1. Classificação

As definições e classificação dos distúrbios hipertensivos na gestação estão apresentadas no Quadro 10.1. Em relação às Diretrizes Brasileiras de HA de 2020, foram acrescentados os conceitos de HAB e HM e a Síndrome da encefalopatia posterior reversível (*posterior reversible encephalopathy syndrome* – PRES).^456,459,460^

**Quadro 10.1:** Definições e classificação dos distúrbios hipertensivos na gestação

**DEFINIÇÕES**	
Hipertensão do avental branco	PAS ≥ 140 mmHg e/ou PAD ≥ 90 mmHg quando medida no consultório ou clínica e PA < 135/85 mmHg quando medida com MAPA na vigília ou MRPA.
Hipertensão mascarada	PAS < 140 mmHg e/ou PAD < 90 mmHg quando medida no consultório ou clínica e PA ≥ 135/85 mmHg quando medida com MAPA na vigília ou MRPA.
Hipertensão na gestação	PAS ≥ 140 mmHg e/ou PAD ≥ 90 mmHg, medida em duas ocasiões com pelo menos 4 horas de intervalo.
Hipertensão grave da gestação	PAS ≥ 160 mmHg e/ou PAD ≥ 110 mmHg, medida em duas ocasiões com pelo menos 4 horas de intervalo.
Proteinúria	Proteinúria ≥ 300 mg em medida de 24 horas, razão proteinúria/creatinina na urina de 0,3 g/g em amostra isolada ou ++ em fitas reagentes (idealmente quantificar).
**CLASSIFICAÇÃO**	
Pré-eclâmpsia (com ou sem sinais de gravidade)	PAS ≥ 140 mmHg e/ou PAD ≥ 90 mmHg em geral após 20 semanas de gestação e frequentemente acompanhado de proteinúria. Na ausência de proteinúria, pode-se considerar o diagnóstico na presença de sinais de gravidade: trombocitopenia (< 100.000.10^9^/L), creatinina > 1,1 mg/dL ou duas vezes a creatinina basal, elevação de duas vezes das transaminases hepáticas, edema de pulmão, sintomas visuais ou cefaleia sem outros diagnósticos alternativos.
Hipertensão crônica	HA diagnosticada ou presente antes da gestação ou antes das 20 semanas de gestação; ou HA que é diagnosticada pela primeira vez durante a gravidez e que não se resolve no período pós-parto.
Hipertensão crônica com pré-eclâmpsia sobreposta	Pré-eclâmpsia em uma mulher com histórico de HA antes da gravidez ou antes de 20 semanas de gestação.
Hipertensão gestacional	PAS ≥ 140 mmHg e/ou PAD ≥ 90 mmHg em mulher com PA previamente normal, após 20 semanas de gestação, medida em duas ocasiões com pelo menos 4 horas de intervalo, sem proteinúria ou sinais de gravidade, e que retorna ao normal no período pós-parto.
**OUTRAS DEFINIÇÕES DIAGNÓSTICAS**	
Eclâmpsia	Convulsões tônico-clônicas na ausência de outras condições causais em paciente com pré-eclâmpsia.
Síndrome HELLP	Hemólise, elevação de enzimas hepáticas e trombocitopenia, associadas ou não à HA.
PRES	Sintomas neurológicos, como cefaleia, convulsões, confusão mental, vômitos e distúrbios visuais. Ressonância magnética mostra edema vasogênico nas regiões parieto-occipitais.

HA: hipertensão arterial; HELLP: hemólise, elevação de enzimas hepáticas e plaquetopenia (Hemolysis, elevated liver enzymes and low platelets); MAPA: medida ambulatorial da pressão arterial; MRPA: medida residencial da pressão arterial; PA: pressão arterial; PAD: pressão arterial diastólica; PAS: pressão arterial sistólica; PRES: Síndrome da encefalopatia posterior reversível (Posterior reversible encephalopathy syndrome).

#### 10.2.2. Predição e Profilaxia de Pré-Eclâmpsia

A predição de pré-eclâmpsia (PE) deve ser feita preferencialmente no 1° trimestre para oportunizar o uso das medicações profiláticas. O modelo de predição da Fetal Medicine Foundation (FMF) pode ser acessado no *link* https://fetalmedicine.org/research/assess/preeclampsia/first-trimester e consiste na combinação de fatores de risco maternos (Quadro 10.2) e medidas de PA média, índice de pulsatilidade das artérias uterinas (ultrassonografia [USG] Doppler de artérias uterinas realizado entre 11 e 14 semanas de gestação) e a dosagem sérica do fator de crescimento derivado da placenta (*placental growth factor* – PLGF). Esse modelo é validado para gestantes no 1° trimestre, tem taxas de detecção de 90% para a predição de PE precoce (< 34 semanas) e de 75% para pré-termo (< 37 semanas) e é superior ao uso dos fatores de risco maternos isolados para definir o alto risco.^461^

**Quadro 10.2:** Fatores de risco para pré-eclâmpsia

	Fator de alto risco (apenas um critério)	Fatores de risco moderados (dois ou mais critérios)
História de gestação anterior	PE prévia	Descolamento de placenta prévio Retardo de crescimento intrauterino prévio Natimorto prévio
Dados demográficos	IMC > 30 kg/m^2^ antes da gestação	Idade materna > 40 anos
Antecedentes clínicos	HA crônica DM antes da gestação Doença renal crônica Lúpus eritematoso sistêmico/síndrome de anticorpos antifosfolípides	
Gestação atual	Reprodução assistida	Nuliparidade Gestação multifetal

DM: diabetes mellitus; HA: hipertensão arterial; IMC: índice de massa corporal; PE: pré-eclâmpsia. Adaptado de Magee *et al*.^456^

Para prevenção de PE, todas as mulheres devem ser incentivadas a realizar AF, com início preferencialmente antes da gestação.^462^ O uso de ácido acetilsalicílico (AAS) na dose noturna de 100 a 150 mg, iniciado antes de 16 semanas até 36 semanas, reduz o risco de PE, principalmente a PE precoce em pacientes de maior risco.^463,464^

A suplementação de cálcio acima de 500 mg, especialmente nas pacientes com baixa ingesta (< 900 mg/dia), iniciada antes da gestação ou até 20 semanas também pode reduzir o risco de PE.^456,465,466^

#### 10.2.3. Abordagem Terapêutica da Hipertensão na Gestação

Não há diferença nos desfechos entre repouso rígido e repouso relativo em mulheres com HA e proteinúria.^467^ Repouso relativo no hospital, em comparação com atividade rotineira em casa, reduz o risco de HA grave.^468^ Não há indicação rotineira de repouso para HA na gestação.^441^ Desfechos clínicos semelhantes ocorrem para mães e recém-nascidos entre unidades de cuidados pré-natais ou admissão hospitalar, mas as mulheres podem preferir acompanhamento em hospital dia.^469^

Não há indicações específicas para os cuidados durante a internação, mas é necessário monitoramento da condição materna e do feto. A PA deve ser medida pelo menos quatro vezes ao dia, com avaliação de peso e diurese diários e orientação sobre sinais premonitórios. Deve-se realizar exames laboratoriais (hemograma com plaquetas, enzimas hepáticas, ácido úrico, creatinina e proteinúria uma a duas vezes por semana). O desenvolvimento do feto pode ser acompanhado pela avaliação do crescimento e dos movimentos, do bem-estar fetal e perfil biofísico fetais e ultrassonografia.^469^

O início de medicação para gestantes com HA não grave (abaixo de 160/110 mmHg) é ainda controverso, exceto em gestantes com LOA. Uma RS de Cochrane mostrou que tratar HA leve a moderada com meta mais intensiva não reduz significativamente a morbidade materna, fetal e do recém-nascido.^470^ Em situações de HA grave persistente por ≥ 15 min, indica-se o tratamento medicamentoso além de MNM para evitar lesão neurológica irreversível. Para evitar mortes maternas, PAS > 150-160 mmHg deve ser considerada para tratamento urgente. Em alinhamento com as demais diretrizes internacionais e nacionais, recomenda-se o ponto de corte de 160 mmHg.^45,456,459^

Tratamento agressivo (PAD até 85 mmHg) vs. tratamento não agressivo (PAD até 100 mmHg) resulta em redução de HA grave e de desfechos fetais desfavoráveis. A meta do controle da HA deve ser a PAS > 120 e < 160 mmHg e PAD > 80 e < 110 mmHg, já que tanto a HA quanto a hipotensão induzida podem prejudicar a perfusão placentária e, consequentemente, o crescimento fetal.^470^ O objetivo do tratamento é prevenir a progressão de LOA, complicações cardíacas e cerebrovasculares, além das complicações obstétricas e fetais.^471^

A recomendação de terapia medicamentosa em mulheres com hipertensão durante a gestação é baseada em estudos observacionais, visto a dificuldade de ECRs nessa população. Dessa forma, as recomendações terão baixa certeza de evidência. A terapia medicamentosa deve ser iniciada com monoterapia pelas medicações consideradas de primeira linha (metildopa, nifedipina de ação prolongada ou amlodipina, ou BB – exceto atenolol);^472^ caso não haja o controle adequado, deve-se associar outra medicação de primeira linha ou de segunda linha (diurético tiazídico, clonidina ou hidralazina). IECAs e BRAs são formalmente contraindicados na gestação pelo risco de malformação fetal que pode levar a injúria renal intrauterina,^471,473^ assim como atenolol, pelo alto risco de restrição de crescimento fetal relacionado ao seu uso.^471,473^ O uso de ARM também está contraindicado na gestação pelo bloqueio hormonal, e há falta de dados de segurança do uso da medicação, sendo considerada categoria C pela *Food and Drug Administration* (FDA). Evitar também o uso de diuréticos em pacientes com PE pela possibilidade de piorar a depleção do volume intravascular.^471^ Estudo comparando a eficácia de labetalol, nifedipina de ação prolongada e metildopa para o manejo de HA grave na gestação sugeriu que todas as medicações eram opções viáveis, porém a nifedipina de ação prolongada foi mais efetiva do que labetalol e metildopa.^474^

Nas formas graves de hipertensão na gestação, duas recentes revisões sistemáticas e metanálises analisaram as melhores estratégias terapêuticas para esses quadros. Uma delas, envolvendo 41 estudos na RS e 46 estudos na metanálise, sugeriu eficácia similar entre nifedipina, hidralazina e labetalol no tratamento de hipertensão grave na gestação.^475,476^ Na outra, incluindo 19 estudos randomizados, tanto labetalol quanto hidralazina foram eficientes para os distúrbios hipertensivos da gestação, mas o labetalol cursou com menos hipotensão materna.^476^ Em diretrizes internacionais, recomenda-se labetalol intravenoso (IV) para tratamento de EH na eclâmpsia ou PE grave, mas esse medicamento não está disponível no Brasil.^117,477^ Assim, o tratamento para EH na gestante pode ser feito com hidralazina IV na dose de 5 mg a cada 20 a 30 minutos até a dose de 15 mg. Quando a hidralazina não estiver disponível, pode-se utilizar nifedipina 10 mg via oral, que pode ser repetida na dose de 10 a 20 mg a cada 20 a 30 minutos.^477^ Há advertência para não usar nifedipina sublingual em nenhuma situação de elevação aguda da PA.^1^

Em situações excepcionais, como a presença de edema agudo de pulmão (EAP), HA grave e refratária, o uso de nitroglicerina IV^448^ ou nitroprussiato de sódio^478^ são opções preferenciais para controle urgente da PA, sendo este último usado por no máximo 4 horas, pelo risco de impregnação fetal pelo cianeto.

Recomenda-se o uso de sulfato de magnésio para prevenção da eclâmpsia em gestantes com HA grave sintomática e o tratamento da eclâmpsia.^456,479^

#### 10.2.4. Hipertensão Arterial no Puerpério

Nas pacientes sem HA crônica, a HA costuma se resolver dentro da 1ª semana (5 a 6 dias) após o parto, mas, nesse período, ainda há risco de complicações relacionadas à HA como eclâmpsia, AVC, EAP e IR aguda.^480^

Um ECR brasileiro mostrou que metildopa e captopril foram igualmente eficazes para o controle da PA no puerpério imediato.^481^

As puérperas podem receber qualquer medicação anti-hipertensiva, incluindo os IECAs, sendo a escolha limitada pelo aleitamento, devendo-se priorizar as medicações anti-hipertensivas que passem em quantidade menor pelo leite materno.

O Quadro 10.3 mostra as principais medicações anti-hipertensivas disponíveis no Brasil e sua relação com o aleitamento.^1^

**Quadro 10.3:** Medicamentos anti-hipertensivos e aleitamento materno

Drogas	Eliminação no leite	Aleitamento
Nifedipina	Pouca excreção	Permitido
Anlodipina	Estudos insuficientes	Incerto (aparentemente seguro)
Diltiazem, verapamil	Estudos insuficientes	Incerto (usar outra opção)
Clonidina	Excreção aumentada	Evitar
Enalapril, Captopril	Pouca excreção	Permitido sem restrição
Lisinopril, ramipril	Estudos insuficientes	Incerto (aparentemente seguro)
Losartana, valsartana candesartana, olmesartana, telmisartana	Estudos insuficientes	Incerto (usar outra opção)
Hidroclorotiazida	Pouca excreção	Usar dose baixa (< 50 mg/dia)
Clortalidona	Pouca excreção	Evitar – eliminação lenta no recém-nascido
Furosemida	Estudos insuficientes	Pode diminuir o leite
Espironolactona	Pouca excreção	Permitido
Atenolol	Excreção aumentada	Evitar
Metoprolol	Pouca excreção	Permitido
Carvedilol	Estudos insuficientes	Incerto
Propranolol	Pouca excreção	Permitido
Bisoprolol	Estudos insuficientes	Incerto (aparentemente seguro)
Hidralazina	Pouca excreção	Permitido
Metildopa	Pouca excreção	Permitido

#### 10.2.5. Risco Cardiovascular Futuro

As síndromes hipertensivas da gravidez são um marcador de risco CV futuro, e as respectivas mulheres devem ser abordadas de forma mais atenta e integral para prevenção efetiva de DCV e doença renal.^1,45^

**Table:** 

Mensagens principais sobre cuidados com a hipertensão arterial nas mulheres dentro e fora do período gestacional
A partir dos 60 anos de idade, a PA entre as mulheres costuma ser mais elevada, e a prevalência de HA é maior.
Existem diferenças no desenvolvimento da HA em mulheres devido aos mecanismos reguladores da PA específicos do gênero.
A investigação de causas secundárias de HA em mulheres jovens é importante, destacando-se entre as principais: obesidade, síndrome do ovário policístico, apneia obstrutiva do sono, coarctação de aorta, doenças autoimunes, doenças endócrinas e nefropatias primárias.
Mulheres apresentam fatores de risco específicos associados com HA, incluindo aqueles relacionados a determinantes sociais e fatores biológicos específicos. Destacam-se entre esses fatores específicos: menopausa, síndrome do ovário policístico, uso de contraceptivos orais e doenças hipertensivas específicas da gestação.
A associação entre HA e uso de AHCO mostrou aumento de 13% do risco de novos casos, e a relação dose resposta é linear para cada 5 anos de uso.
Efeitos adversos dos anti-hipertensivos são relatados com mais frequência em mulheres, em especial na menopausa, e podem explicar menor adesão das mulheres ao tratamento da HA.
Há evidências de que o sexo feminino foi um preditor de terapia anti-hipertensiva menos agressiva em relação ao masculino.
Classificar HA na gestação em pré-eclâmpsia, HA crônica, HA crônica com pré-eclâmpsia sobreposta e HA gestacional. Devem ser considerados os diagnósticos de eclâmpsia, PRES, síndrome HELLP, HAB e HM.

AHCO: anticoncepcional hormonal combinado oral; HA: hipertensão arterial; HAB: hipertensão do avental branco; HM: hipertensão mascarada; HELLP: hemólise, elevação de enzimas hepáticas e plaquetopenia (Hemolysis, elevated liver enzymes and low platelets); PA: pressão arterial; PRES: Síndrome da encefalopatia posterior reversível (Posterior reversible encephalopathy syndrome).

**Table:** 

Recomendações para abordagem da HA em mulheres dentro e fora do período gestacional	Força da recomendação	Certeza da evidência
Recomenda-se a monitorização da PA em mulheres jovens antes da prescrição de AHCO e após o início do uso; reforçar a importância de medidas regulares da PA (a cada 6 meses), incluindo medidas domiciliares e o controle dos demais FRCVs.	FORTE	MODERADA
Recomenda-se considerar outros métodos contraceptivos ou a descontinuação dos AHCOS para mulheres quando houver elevação persistente da PAS ≥ 160 mmHg e/ou PAD ≥ 100 mmHg com o uso de AHCO.	FORTE	BAIXA
Recomenda-se, para o tratamento medicamentoso da HA na mulher durante e após a menopausa, iniciar com a combinação de inibidor do SRAA com BCC ou diurético tiazídico ou similar.	FORTE	MODERADA
Recomendam-se metas de PA iguais para mulheres e homens, assim como a utilização das mesmas classes de anti-hipertensivos para o tratamento da HA em mulheres e homens.	FORTE	MODERADA
Recomenda-se avaliar o risco de PE entre 11 e 14 semanas de gestação utilizando no mínimo os fatores de risco maternos, preferencialmente associado à análise da pulsatilidade das artérias uterinas no USG com Doppler e dosagem do fator de crescimento derivado da placenta.	FORTE	BAIXA
Recomenda-se, em todas as mulheres gestantes, exceto com contraindicações, a prática de atividades físicas para reduzir a probabilidade de HA gestacional e PE.	FORTE	MODERADA
Recomenda-se a reposição de cálcio nas gestantes de alto risco com baixa ingesta (< 900 mg/dia) para reduzir risco de PE.	FORTE	MODERADA
Recomenda-se o uso de AAS iniciado antes de 16 semanas nas gestantes de alto risco para reduzir a PE abaixo de 34 semanas.	FORTE	MODERADA
Não se recomenda utilizar o tratamento não medicamentoso isoladamente em situações de PAS acima de 160 mmHg persistente por mais de 15 minutos.	FORTE	BAIXA
Recomenda-se o repouso relativo em hospital-dia com monitoramento para gestantes com PE, e a internação hospitalar deve ser indicada em gestantes com HA grave.	FORTE	MODERADA
Recomenda-se, na gestante, tratamento medicamentoso urgente em HA grave e na presença de sinais clínicos de emergência.	FORTE	MODERADA
Recomenda-se iniciar tratamento medicamentoso na gestante quando a PA estiver acima de 150-160/100-110 mmHg, com o objetivo de manter a PA entre 120-160/80-100 mmHg.	FRACA	BAIXA
Recomenda-se iniciar o tratamento anti-hipertensivo em gestantes com HA com metildopa ou BCCs di-hidropiridínicos (nifedipina de longa duração ou amlodipina).	FORTE	BAIXA
Não se recomenda fazer uso de IECA ou BRA na gestação pelo risco de malformação fetal.	FORTE	MODERADA
Não se recomenda o uso de atenolol, prazosina e ARM durante a gestação devido a possíveis riscos associados.	FORTE	BAIXA
Recomenda-se o uso de sulfato de magnésio para a prevenção da eclâmpsia em gestantes com HA grave sintomática e para o tratamento da eclâmpsia.	FORTE	MODERADA
Recomenda-se o acompanhamento de longo prazo de toda a mulher que teve HA na gestação para a prevenção efetiva de DCV.	FORTE	BAIXA

AAS: ácido acetilsalicílico; AHCO: anticoncepcional hormonal combinado oral; ARM: antagonista dos receptores mineralocorticoides; BCCs: bloqueadores dos canais de cálcio; BRA: bloqueador dos receptores da angiotensina; DCV: doença cardiovascular; FRCV: fator de risco cardiovascular; HA: hipertensão arterial; IECA: inibidor da enzima conversora da angiotensina; PA: pressão arterial; PAD: pressão arterial diastólica; PAS: pressão arterial sistólica; PE: pré-eclâmpsia; SRAA: sistema renina-angiotensina-aldosterona; USG: ultrassonografia.

## 11. Crise Hipertensiva

### 11.1. Definição e Classificação

Crise hipertensiva caracteriza-se por elevação aguda e importante da PA, acompanhada ou não por LOAs agudas.^1,44,45,482,483^ EHs são situações clínicas sintomáticas em que há elevação acentuada da PA (definida arbitrariamente como PAS ≥ 180 e/ou PAD ≥ 110 mmHg ou, dependendo da situação, até mesmo com valores menores) com LOA aguda e progressiva.^1,44,45,482,483^ Atualmente, discute-se o uso do termo "urgência hipertensiva" e recomenda-se que a nomenclatura apropriada seria "elevação importante da PA sem lesão progressiva de órgãos-alvo".^1,44,45,482,483^ O Quadro 11.1 mostra as principais diferenças entre EH e a elevação importante da PA sem LOA aguda (antes da urgência hipertensiv).

**Quadro 11.1:** Diagnóstico, prognóstico e conduta nas crises hipertensivas

Elevação importante da PA sem LOA aguda	Emergência
Nível de pressão arterial elevado acentuado;	Nível de pressão arterial elevado acentuado;
Sem LOA aguda e progressiva;	Com LOA aguda e progressiva;
Combinação medicamentosa oral;	Medicamentos parenterais;
Sem risco iminente de morte;	Com risco iminente de morte;
Acompanhamento ambulatorial precoce (1 a 7 dias).	Internação em UTI.

LOA: lesão em órgãos-alvo; PA: pressão arterial; UTI: unidade de tratamento intensivo.

Uma condição comum no atendimento em setores de emergência, que faz parte do diagnóstico diferencial da crise hipertensiva, é a pseudocrise hipertensiva. Nesta, há elevação da PA sem LOA e sem risco imediato de morte. Geralmente, ocorre em pacientes com HA não controlada, com medidas de PA muito elevadas, mas oligossintomáticos ou assintomáticos, ou ainda provocadas por algum evento emocional, doloroso ou com desconforto, como enxaqueca, tontura rotatória, cefaleias de origem musculoesquelética e síndrome do pânico.^1,482^

### 11.2. Classificação

A EH não é definida apenas pelo nível da PA, apesar de elevada, mas predominantemente pelo *status* clínico do paciente. Pode se manifestar como um evento CV, cerebrovascular, renal ou na forma de PE ou eclâmpsia durante a gestação. O Quadro 11.2 mostra a classificação das EHs.

**Quadro 11.2:** Classificação das emergências hipertensivas

EMERGÊNCIAS HIPERTENSIVAS
**Cerebrovasculares** – Encefalopatia hipertensiva – Hemorragia intracerebral – Hemorragia subaracnoide – AVC Isquêmico **Cardiocirculatórias** – Dissecção aguda de aorta – Edema agudo de pulmão com insuficiência ventricular esquerda – Infarto agudo do miocárdio – Angina instável **Sistêmicas** – Hipertensão acelerada/maligna – Hipertensão MDO **Crises adrenérgicas graves** – Crise do feocromocitoma – Dose excessiva de drogas ilícitas (cocaína, *crack*, LSD) **Hipertensão na gestação** – Eclâmpsia – Pré-eclâmpsia grave – Síndrome HELLP – Hipertensão grave em final de gestação

AVC: acidente vascular cerebral; HELLP: hemólise, elevação de enzimas hepáticas e plaquetopenia (Hemolysis, elevated liver enzymes and low platelets); LSD: dietilamida do ácido lisérgico (lysergic acid diethylamide); MDO: múltiplos danos aos órgãos-alvo.

### 11.3. Aspectos epidemiológicos e prognósticos

#### 11.3.1. Epidemiologia

A crise hipertensiva tem uma prevalência de 0,45 a 0,59% de todos os atendimentos de emergência hospitalar, e a EH responde por 25% de todos os casos de crise hipertensiva. O AVCI e o EAP são as situações mais frequentemente encontradas nas EH.^1,482^

#### 11.3.2. Prognóstico

A letalidade da EH não tratada pode atingir aproximadamente 80% ao final de 1 ano, dependendo do tipo de EH, e o tratamento anti-hipertensivo melhora substancialmente o prognóstico.^1,482^ A sobrevida de até 5 anos é maior em indivíduos com elevação importante da PA sem LOA aguda do que com EH.^1,482^

### 11.4. Investigação Clínico-Laboratorial Complementar

É essencial a realização de história clínica e exame físico direcionados para o diagnóstico correto da crise hipertensiva. Se houver uma condição com risco de morte, a conduta deverá ser iniciada concomitantemente à investigação.^1,44,45,482,483^Na história clínica, são essenciais as informações sobre a PA usual do paciente e situações que possam ter desencadeado o seu aumento (ansiedade, estresse, dor, abuso de sal). O uso de anti-hipertensivos (dose e adesão) ou sua descontinuação (inibidores adrenérgicos) ou o uso de substâncias que possam aumentar a PA devem ser considerados, incluindo o uso de drogas ilícitas.^1,44,45,482,483^ É importante a abordagem dos sintomas mais frequentes, como dispneia, palpitações, cefaleia, dor torácica ou sintomas neurológicos.^1,44,45,482-484^ A PA deve ser aferida em ambos os membros superiores, preferencialmente em ambiente tranquilo. A investigação sistematizada clínico-laboratorial e de exames de imagem auxilia no diagnóstico de LOA aguda (Quadro 11.3).^1,44,45,482-485^

**Quadro 11.3:** Investigação clínico-laboratorial complementar de acordo com as lesões de órgãos-alvo nas emergências hipertensivas^1,44,45,482,483^

Órgãos-alvo	Emergência hipertensiva	Sintomas	Alterações do exame físico	Investigação complementar
Coração e artérias	• IC, EAP • Síndrome coronariana • Dissecção aguda de aorta	• Dor torácica, abdominal, dorsal ou lombar • Dispneia, ortopneia • Palpitações • Edema	• FC, ritmo de galope • Estase jugular, congestão pulmonar, abdominal e periférica • Sopros cardíacos e vasculares • Assimetria ou ausência de pulsos periféricos • Diferença de PA entre os membros	• ECG, radiografia de tórax • Ecocardiograma • Saturação de O2 • Marcadores de necrose miocárdica • BNP e NT-proBNP • Angio TC e RNM de tórax
Cérebro	• AVC • Encefalopatia hipertensiva	• Vertigem, cefaleia • Alteração visual, auditiva ou na fala • Paresia ou parestesias • Confusão mental, agitação, sonolência	• Alteração do nível de consciência • Convulsão, déficits focais, rigidez de nuca	• TC ou RNM de crânio • Punção lombar para coleta de líquor
Rim	• IRA • Microangiopatia trombótica	• Alteração no volume ou frequência miccional • Alteração no aspecto da urina • Edema • Anorexia, perda de peso, náuseas, vômitos, fadiga	• Edema • Hematúria ou urina espumosa • Massas e sopros abdominais • Palidez cutaneomucosa	• Exame de sedimento urinário com hematúria dismórfica e/ou proteinúria, RAC, hemograma, creatinina, ureia, sódio, potássio, cloro, gasometria venosa, desidrogenase lática USG renal e de vias urinárias Doppler de artérias renais TC ou RNM de abdômen
Retina	• Retinopatia • Grau KW III e IV	• Turvação visual • Fosfenas, escotomas • Amaurose	• Espasmos, cruzamentos arteriovenosos patológicos • Papiledema, hemorragias retinianas, exsudatos algodonosos	
Suspeita de EH	• ECG, radiografia de tórax, hemograma, troponina, creatinina, urina I e potássio; • Excluir gravidez em mulheres em idade fértil.			

AVC: acidente vascular cerebral; BNP e NT-proBNP: peptídeos natriuréticos tipo B; EAP: edema agudo de pulmão; ECG: eletrocardiograma; EH: emergência hipertensiva; FC: frequência cardíaca; HA: hipertensão arterial; IC: insuficiência cardíaca; IRA: injúria renal aguda; HELLP: hemólise, elevação de enzimas hepáticas e plaquetopenia; KWB: classificação de Keith-Wagener-Barker; PA: pressão arterial; PE: pré-eclâmpsia; O2: oxigênio; RAC: relação albuminúria/creatininúria; RNM: ressonância nuclear magnética; TC: tomografia computadorizada; USG: ultrassonografia.

### 11.5. Tratamento Geral da Crise Hipertensiva

A presença e o tipo de LOA aguda devem orientar o tratamento da crise hipertensiva (Figura 11.1 e Quadro 11.4). É importante considerar os riscos da redução rápida e excessiva da PA, com resultante isquemia irreversível de órgãos-alvo (devido à perda da autorregulação da PA), e avaliar os benefícios do tratamento para a minimização da progressão da LOA identificada.^486^ Não há evidências de que o paciente com elevação importante da PA sem LOA aguda deva ser tratado em unidade de emergência, portanto recomenda-se a condução ambulatorial, tal qual um paciente com HA não controlada e assintomática.^1,482,486^ A primeira medida a ser tomada em um paciente com elevação importante da PA com sintomas sem relação com LOA aguda é a observação clínica por 30 minutos em ambiente calmo, avaliando a resolução dos sintomas e a redução da PA.^1,482^ Não se recomenda a redução aguda e abrupta da PA > 25 a 30% nas primeiras 2 a 4 horas devido ao risco de isquemia tecidual, o que pode acontecer com o uso de nifedipina de liberação rápida, o que é proscrito.^1,482,487^

**Figura 11.1:**
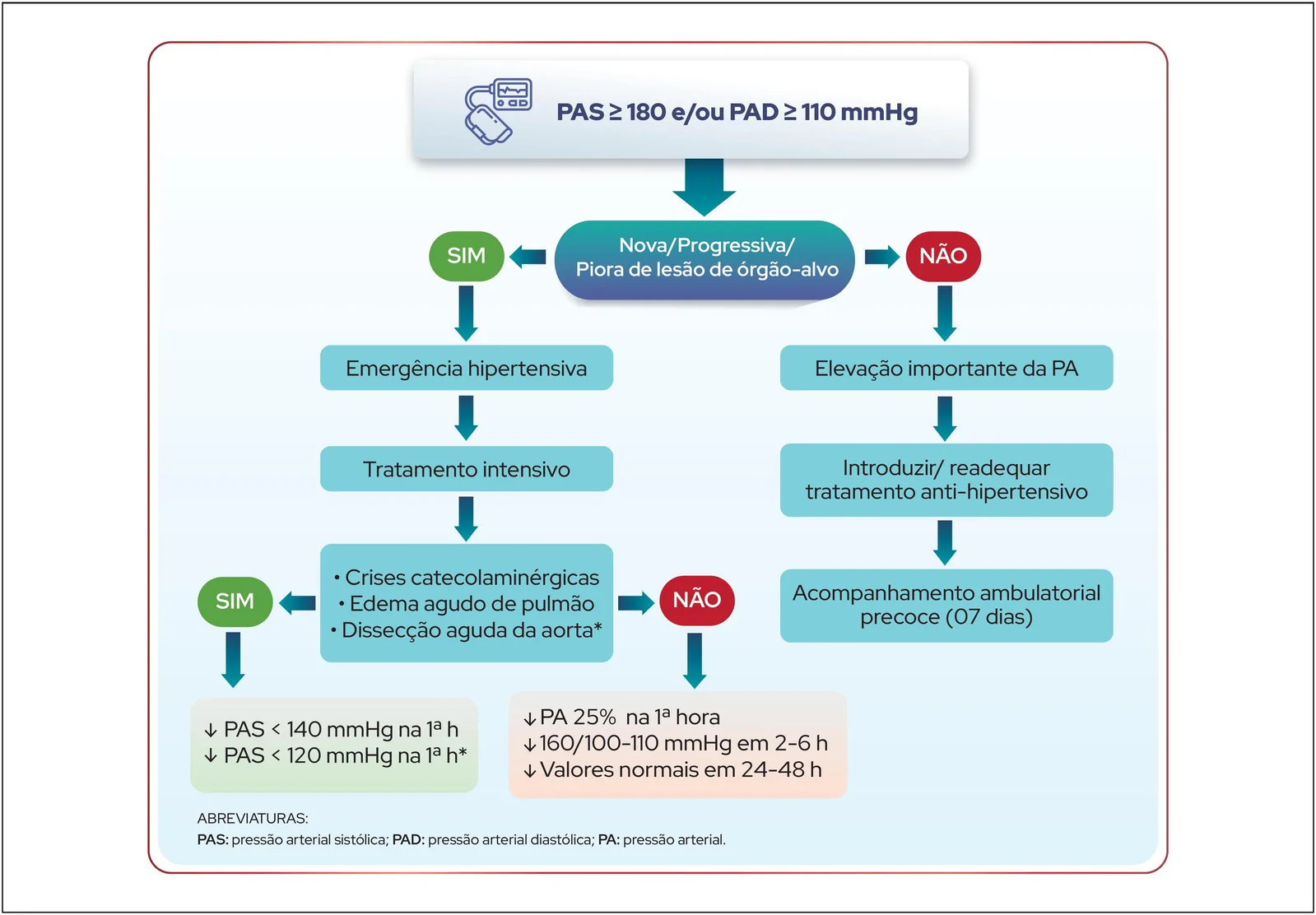
Fluxograma de atendimento da crise hipertensiva.^1,44^

**Quadro 11.4:** Medicamentos usados por via intravenosa para o tratamento das emergências hipertensivas

Medicamentos	Modo de administração e dosagem	Início	Duração	Indicações	Eventos adversos e precauções
**Nitroprussiato de sódio** (vasodilatador arterial/venoso estimula a formação de GMPc)	Infusão contínua 0,25-10 mg/kg/min EV	Imediato	1-2 min	Maioria das emergências hipertensivas	Intoxicação por cianeto, hipotensão grave, náuseas, vômitos. Cuidado na IRA e doença hepática. Proteger da luz.
**Nitroglicerina** (vasodilatador arterial/venoso)	Infusão contínua EV 5-15 mg/h	2-5 min	3-5 min	Insuficiência coronariana, EAP	Cefaleia, taquicardia reflexa, taquifilaxia, rubor, meta-hemoglobinemia
**Metoprolol** (BB seletivo)	5 mg EV (repetir 10/10 min, se necessário até 20 mg)	5-10 min	3-4 h	Insuficiência coronariana, dissecção aguda de aorta	Bradicardia, BAV avançado, IC, broncoespasmo
**Esmolol** (bloqueador β-adrenérgico seletivo de ação ultrarrápida)	Ataque: 500 μg/kg Infusão intermitente 25-50 μg/kg/min ↑25 μg/kg/min 10-20 min. Máximo 300 μg/kg/min	1-2 min	1-20 min	Dissecção aguda de aorta (em combinação com NPS), hipertensão pós-operatória grave	Náuseas, vômitos, BAV 1º grau, broncoespasmo, hipotensão
*Fentolamina (bloqueador α-adrenérgico)	Infusão contínua: 1-5 mg Máximo 15 mg	1-2 min	3-5 min	Excesso de catecolaminas	Taquicardia reflexa, rubor, tontura, náuseas, vômitos
*Trimetafano (bloqueador ganglionar do SNS e SNPS)	Infusão contínua: 0,5-1,0 mg/min. ↑0,5 mg/min até o máximo de 15 mg/min	1-5 min	10 min	Excesso de catecolaminas, dissecção aguda de aorta	Taquifilaxia
**Hidralazina** (vasodilatador de ação direta)	10-20 mg EV ou 10-40 mg IM 6/6 h	10-30 min	3-12 h	Eclâmpsia	Taquicardia, cefaleia, vômitos. Piora angina e IAM. Cuidado com pressão intracraniana
*Diazóxido (vasodilatador)	Infusão 10-15 min 1-3 mg/kg. Máximo 150 mg	1-10 min	3-18 h	Encefalopatia hipertensiva	Retenção de sódio e água, hiperglicemia e hiperuricemia
*Fenoldopam (agonista dopaminérgico)	Infusão contínua 0,1-1,6 μg/kg/min	5-10 min	10-15 min	IRA	Cefaleia, náuseas, rubor
*Nicardipina (BCC)	Infusão contínua 5-15 mg/h	5-10 min	1-4 h	AVC, encefalopatia hipertensiva, EAP	Taquicardia reflexa, evitar em pacientes com IC ou isquemia miocárdica
*Labetalol (bloqueador α/β-adrenérgico)	Ataque: 20-80 mg 10-10 min Infusão contínua 2 mg/min (máximo 300 mg/24 h)	5-10 min	2-6 h	AVC, dissecção aguda de aorta (em combinação com NPS)	Náuseas, vômitos, BAV, broncoespasmo, hipotensão ortostática
*Enalaprilato (IECA)	Infusão intermitente 1,25-5,0 mg 6/6 h	15 min	4-6 h	Insuficiência ventricular esquerda com EAP	Hipotensão, IRA
**Furosemida** (diurético de alça)	20-60 mg (repetir após 30 min)	2-5 min	30-90 min	EAP, situações de hipervolemia	Hipopotassemia

AVC: acidente vascular cerebral; BAV: bloqueio atrioventricular; BB: betabloqueador; BCC: bloqueador dos canais de cálcio; EAP: edema agudo de pulmão; EV: endovenosa; GMPc: monofosfato cíclico de guanosina; IAM: infarto agudo do miocárdio; IC: insuficiência cardíaca; IECA: inibidor da enzima de conversão da angiotensina; IM: intramuscular; IRA: injúria renal aguda; NPS: nitroprussiato de sódio; SNPS: sistema nervoso parassimpático; SNS: sistema nervoso simpático.

* Não disponíveis no Brasil.

Em estudo de coorte retrospectivo, a recidiva de novas idas à unidade de emergência e a mortalidade foram semelhantes para pacientes com PA acentuadamente elevada, mas sem LOA aguda, independentemente de terem sido tratados com terapia anti-hipertensiva aguda ou não. Se não houver redução da PA ou resolução dos sintomas após a observação clínica, recomenda-se o uso de anti-hipertensivos orais de rápido início de ação e curta duração (geralmente clonidina ou captopril), ajuste dos anti-hipertensivos de uso crônico ou início de tratamento anti-hipertensivo (ver Capítulo 7 de tratamento medicamentoso).^1,482,488^

### 11.6. Emergências Hipertensivas em Situações Especiais

#### 11.6.1. Encefalopatia Hipertensiva^1,482,485^

Encefalopatia hipertensiva é uma LOA neurológica caracterizada por edema cerebral secundário à elevação aguda da PA. Geralmente reversível, pode ocorrer em pacientes com HA crônica que desenvolvem hipertensão maligna (a ser detalhado posteriormente neste capítulo) ou nos indivíduos com normotensão que apresentam elevações agudas excessivas da PA. A falha da autorregulação da perfusão cerebral resulta em aumento da pressão intracraniana. O início é insidioso, podendo evoluir com cefaleia holocraniana, náuseas e/ou vômitos, alterações visuais, confusão mental, coma, crises convulsivas generalizadas, hiperreflexia e sinais de hipertensão intracraniana. Na presença de déficit localizado, deve-se pensar em lesão neurológica focal, e ressonância nuclear magnética (RNM) ou tomografia computadorizada (TC) de crânio devem ser realizadas.^489^

O objetivo do tratamento é restaurar a autorregulação cerebral com redução da PA de forma lenta (reduzir a PA inicialmente em 20 a 25% em 1 h), pois reduções rápidas podem provocar hipoperfusão cerebral devido à perda da autorregulação da PA. Usar medicamentos IV tais como nitroprussiato de sódio (NPS), nitroglicerina (NTG) ou esmolol (disponíveis no Brasil), devendo-se ter cautela com NTG ou NPS, pela demonstração de diminuição do fluxo sanguíneo cerebral observado com seu uso em indivíduos normotensos.^490^ Em uma RS comparando tratamento intravenoso de EH neurológicas, incluindo encefalopatia hipertensiva, devido à muito baixa qualidade de evidência, não foi possível determinar qual é o melhor medicamento para essas situações.^491^ Anti-hipertensivos orais também devem ser iniciados para melhor controle da PA (meta de PA 160/100 mmHg em até 48 h).

#### 11.6.2. Acidente Vascular Cerebral

A HA é o principal fator de risco para AVC, principalmente hemorrágico. O diagnóstico é baseado em exame neurológico completo e, para avaliar a gravidade do quadro, deve ser utilizada a escala do *National Institutes of Health Stroke Scale* (NIHSS). A TC de crânio e a RNM permitem definir o tipo do AVC (isquêmico 85% ou hemorrágico 15%) e o território envolvido.^1,482,483,485^ A RNM é mais sensível do que a TC para infartos incipientes.^492^ O tratamento pré-hospitalar com reduções rápidas e expressivas da PA não mostrou benefícios no AVCI ou no AVCH, exceto diminuição de déficit motor em pacientes com AVCH.^493^

##### 11.6.2.1. Acidente Vascular Cerebral Isquêmico

A PA elevada é comum durante o AVCI, ocorrendo em cerca de 80% dos pacientes, especialmente naqueles com história de HA. A PA frequentemente diminui espontaneamente em 90 a 120 minutos durante a fase aguda do AVC.^494-497^

As recomendações principais para o tratamento da EH com AVC isquêmico estão descritas na tabela no final do capítulo.

##### 11.6.2.2. Acidente Vascular Cerebral Hemorrágico

O AVCH representa 15% dos casos de AVC, com incidência anual de 13,2 a cada 100.000 indivíduos em recente estudo suíço.^498^ A PA elevada aumenta o risco de expansão do hematoma, risco de morte e piora o prognóstico. O edema cerebral ocorre em 30% dos casos e, normalmente, ocorre nas primeiras 24 horas. Assim, a craniectomia descompressiva e a transferência para centros especializados devem ser realizadas.^1,482,492,499^

As recomendações principais para o tratamento da EH com AVC hemorrágico estão descritas na tabela no final do capítulo.

#### 11.6.3. Síndromes Coronarianas Agudas

As síndromes coronarianas agudas (SCAs) podem ser acompanhadas de elevação da PA, devido a um reflexo da isquemia miocárdica. O aumento da RVP eleva a demanda de oxigênio pelo miocárdio, por aumento da tensão parietal do ventrículo esquerdo.^500-503^

Em idosos e indivíduos frágeis, o alvo da PA pode ser menos restrito. Hidralazina, nifedipina e NPS não estão indicados.^1,482,485^ Nitratos IV reduzem a RVP, melhoram a perfusão coronariana e possuem efeito venodilatador sistêmico, reduzindo a pré-carga e o consumo de oxigênio pelo miocárdio.

#### 11.6.4. Edema Agudo de Pulmão

Cerca de 40% dos pacientes admitidos com EAP e EH têm função preservada do ventrículo esquerdo.^504^ Isquemia miocárdica também pode estar envolvida na fisiopatologia do EAP associado à EH.^1,482,485^ O EAP deve ser tratado em UTI, com medicação IV, monitoramento e diminuição gradativa da PA. NTG e NPS reduzem a pré- e a pós-carga. DIU de alça também diminui a sobrecarga de volume e, consequentemente, a PA. Em alguns casos, o uso de ventilação não invasiva (*Continuous Positive Airway Pressure/Bilevel Positive Airway Pressure* [CPAP/BiPAP]) pode ser indicado para reduzir o edema pulmonar e o retorno venoso.^1,482,485^

#### 11.6.5. Dissecção Aguda de Aorta

Concomitantemente, deve-se promover controle da dor com morfina. Os anti-hipertensivos de escolha são BBs IV de ação rápida e titulável (metoprolol ou esmolol), que diminuem a FC e a velocidade de ejeção do ventrículo esquerdo. Em asmáticos, BCCs não di-hidropiridínicos podem ser usados. NPS deve ser iniciado após o uso de BBs para não elevar a FC e a velocidade de ejeção aórtica.^1,485,485,505^

#### 11.6.6. Crises Adrenérgicas

As crises adrenérgicas podem ser causadas pela suspensão súbita de simpatolíticos e de BBs, ou por feocromocitomas (FEO) ou pelo uso de substâncias ilícitas. Nessas crises, a PAS deve ser reduzida a < 140 mmHg na 1ª hora.^1,482,483^

Nos casos de suspensão abrupta de medicamentos com ação no sistema nervoso simpático, recomenda-se a sua reintrodução imediata, devido ao risco de complicações graves.^1,482,483^ Paroxismos recorrentes da tríade de palpitação, sudorese e cefaleia, sem relato de uso de drogas ilícitas, devem alertar para investigação de FEO.^1,482,506^

Recomenda-se tratamento com doxazosina,^507^ pois a fentolamina não está disponível no Brasil. O β-bloqueio só pode ser iniciado após o α-bloqueio, por causa da elevação paradoxal da PA com o bloqueio da vasodilatação β-adrenérgica.^507^

Anfetaminas, *ecstasy* e cocaína elevam a PA por ação simpaticomimética.^508,509^ As anfetaminas aumentam a PA e causam taquicardia, palpitações, sudorese e arritmias.^508^ A síndrome serotoninérgica (rabdomiólise e IRA) pode ocorrer na intoxicação por *ecstasy*.^508^ O aumento agudo da PA induzido por cocaína é mais intenso em hipertensos do que em normotensos e é potencializado por cafeína, nicotina ou, particularmente, por álcool.^1,482,509^ Nesta condição, ocorre vasoconstrição, formação de trombos, aumento da demanda de O2 miocárdico, defeitos de condução e arritmias.^1,482^

#### 11.6.7. Hipertensão Acelerada/Maligna

A HA maligna é uma EH com elevação excessiva da PA e progressão acelerada da doença, caracterizada por lesão microvascular aguda, dano endotelial sistêmico e perda dos mecanismos de autorregulação, afetando retina, cérebro, coração, rins e sistema vascular. Os achados característicos incluem retinopatia Grau 4 de Keith-Wagener-Barker, com ou sem IRA, e necrose fibrinoide de arteríolas renais, que refletem dano endotelial sistêmico, mas pode-se considerar a presença da condição clínica mesmo na ausência desses achados se houver mais de uma lesão concomitante e grave de órgãos-alvo.^510,511^ Pode apresentar evolução rapidamente progressiva e fatal, com mortalidade de 80% em 2 anos, se não tratada.^511^ Geralmente, mas não obrigatoriamente, cursa com HA Estágio 3. A elevação da PA, na presença de hemorragias retinianas e exsudatos ao fundo de olho, mas sem papiledema, é denominada HA acelerada. Os termos "maligna" e "acelerada" são considerados intercambiáveis, sendo usado o termo hipertensão acelerada-maligna (HAA/M).^512^ Quando há papiledema, é necessário realizar neuroimagem para descartar outras causas neurológicas.^1,482,485^

Ainda que não seja capaz de reverter totalmente o elevado risco renal e cardíaco, o tratamento da HAA/M reduz drasticamente a mortalidade.^1,482^ Os pacientes devem ser internados e receber medidas terapêuticas intensivas para controle da PA com vasodilatadores de ação imediata (NPS).^1,482,483,485,513^ Em seguida, instituir anti-hipertensivos por via oral, incluindo IECA ou BRA e quaisquer outras classes que se façam necessárias.^1,482,483,485,513^

#### 11.6.8. Hipertensão com Múltiplos Danos aos Órgãos-Alvo

A HA com múltiplos danos aos órgãos-alvo (DOA) é definida pelo envolvimento de pelo menos três dos quatro sistemas a seguir:^514^ renal (rápida deterioração da função ou proteinúria), cardíaco (HVE importante ou disfunção sistólica, ou anormalidades da repolarização ventricular, ou aumento de troponina), neurológico (AVC ou encefalopatia hipertensiva) e hematológico (hemólise microangiopática).^515^

#### 11.6.9. Hipertensão na Gravidez: Pré-eclâmpsia e Eclâmpsia

Encontram-se discutidos os principais aspectos de diagnóstico, meta pressórica e tratamento no Capítulo 10.

**Table:** 

Mensagens principais sobre cuidados da crise hipertensiva
A elevação importante da PA (≥ 180/110 mmHg) sem LOA aguda e sem risco de morte iminente permite redução da PA em maior período de tempo com anti-hipertensivos orais, e a reavaliação ambulatorial deve ser precoce em período de 1 a 7 dias.
EH: elevação da PA (≥ 180/110 mmHg) com LOA aguda e risco imediato de morte. Requer redução rápida e gradual da PA em minutos a horas, dependendo da LOA presente. Geralmente, são usados medicamentos endovenosos.
A gravidade da condição clínica não é determinada pelo nível absoluto da PA e, sim, pela magnitude da sua elevação. A definição numérica serve como parâmetro de conduta, mas não deve ser usada como critério absoluto.
O alvo de redução da PA varia de acordo com o tipo de apresentação da EH. A redução da PA deve ser gradual e cuidadosa, em 10 a 15% na 1ª hora de tratamento e de 25% nas 2 horas seguintes ao início do tratamento na maioria das situações clínicas. Alguns alvos de redução da PA são específicos de acordo com a LOA envolvida: • Crises catecolaminérgicas e EAP: PAS < 140 mmHg na 1ª hora. • Dissecção aguda de aorta: PAS < 120 mmHg na 1ª hora.
O tratamento anti-hipertensivo varia de acordo com o diagnóstico da EH. Os medicamentos mais utilizados são nitroprussiato de sódio, nitroglicerina, BB, bloqueadores dos canais de cálcio e diuréticos de alça.

EAP: edema agudo de pulmão; EH: emergência hipertensiva; LOA: lesão de órgãos-alvo; PA: pressão arterial; PAS: pressão arterial sistólica; BB: betabloqueador.

**Table:** 

Recomendações principais para as emergências hipertensivas e para elevação importante da pressão arterial sem lesão de órgão-alvo aguda	Força da recomendação	Certeza da evidência
Recomenda-se, em pacientes com elevação importante da PA sem LOA aguda (antes denominada urgência hipertensiva), reavaliação ambulatorial em 1 a 7 dias com alvo de PAS < 160 e PAD < 100 mmHg.	FRACA	BAIXA
Recomenda-se que o paciente com EH deva ser admitido em UTI, usar anti-hipertensivos IV com monitorização da PA e a observação da progressão da LOA.	FORTE	MODERADA
Recomenda-se que, no paciente com EH, a PA seja reduzida em até 25% na 1ª h; se estável, até 160/100 mmHg nas próximas 2 a 6 h; e, para níveis normais, nas próximas 24 a 48 h, exceto em LOA específicas (AVC, dissecção aguda de aorta, PE, eclâmpsia e FEO).	FRACA	MODERADA
Recomenda-se, em pacientes com AVCI e indicação de trombólise, reduzir PA < 185/110 mmHg com manutenção < 180/105 mmHg nas primeiras 24 h após trombólise e não realizar a infusão de trombolítico ou trombectomia se a PA permanecer ≥ 185/110 mmHg.	FORTE	MODERADA
Recomenda-se, em pacientes com AVCI e PA ≥ 220/120 mmHg, associado a comorbidades (dissecção de aorta, eventos coronarianos agudos, eclâmpsia, pós-trombólise e/ou EAP), uma redução inicial da PA em 15%.	FRACA	BAIXA
Recomenda-se, em pacientes com AVCI e PA ≥ 220/120 mmHg que não receberam trombolítico e não tenham comorbidades, reduzir a PA em 15% durante as primeiras 24 h após início do AVCI.	FRACA	BAIXA
Recomenda-se iniciar ou reiniciar anti-hipertensivos com segurança em indivíduos neurologicamente estáveis com AVCI e PA > 140/90 mmHg.	FORTE	MODERADA
Recomenda-se, em pacientes com HA e AVCI, que a PAS esteja entre 120 e 130 mmHg e a PAD entre 70 e 79 mmHg em 7 a 10 dias após o AVCI.	FORTE	ALTA
Recomenda-se, em pacientes com AVCH e PAS > 220 mmHg, a redução e o monitoramento frequente da PA com infusão IV contínua, com o objetivo de meta de PAS < 180 mmHg.	FORTE	MODERADA
Recomenda-se, em pacientes com AVCH de leve a moderada gravidade e PAS entre 150 e 220 mmHg, reduzir a PA < 140 mmHg com medicamentos IV (esmolol e BCC – nicardipina e clevidipina).	FRACA	MODERADA
Recomenda-se, em pacientes com hemorragia subaracnoidea e PAS > 180 mmHg, a redução gradual e lenta da PAS em período de 24 a 72 h.	FRACA	MODERADA
Recomenda-se, em pacientes com EAP por EH, o uso de NTG IV nas primeiras 48 h, desde que não haja hipotensão, infarto do ventrículo direito ou uso de inibidores da fosfodiesterase tipo 5 (sildenafila, tadalafila) nas 48 h anteriores, podendo ser utilizado em associação a BB ou IECA que reduzam mortalidade (desde que não haja contraindicações).	FRACA	MODERADA
Recomenda-se, em pacientes com SCA por EH, a meta de PAS < 140 mmHg (evitar < 120 mmHg) e PAD entre 70 e 80 mmHg, utilizando esmolol, metoprolol ou NTG em período de 24 a 72 h.	FORTE	ALTA
Recomenda-se, em pacientes com crise adrenérgica, o uso de benzodiazepínicos e nitrato sublingual nos casos mais leves; para os demais casos, os agentes de escolha são NTG e NPS.	FRACA	BAIXA
Não se recomenda usar BB isoladamente na crise adrenérgica por FEO, pois a estimulação do receptor α-adrenérgico, com a vasodilatação β–adrenérgica bloqueada, pode piorar o espasmo de coronária, que pode ser aliviado com BCC.	FRACA	BAIXA
Recomenda-se, em pacientes com dissecção aguda de aorta por EH, atingir os seguintes alvos: FC < 60 bpm e PAS entre 90 e 120 mmHg, que devem ser alcançados em 20 min.	FORTE	BAIXA

AVC: acidente vascular cerebral; AVCI: acidente vascular cerebral isquêmico; BB: betabloqueador; BCC: bloqueador dos canais de cálcio; bpm: batimentos por minuto; EAP: edema agudo de pulmão; EH: emergência hipertensiva; FC: frequência cardíaca; FEO: feocromocitoma; IECA: inibidor da enzima conversora da angiotensina; IV: intravenoso; LOA: lesão de órgãos-alvo; NPS: nitroprussiato de sódio; NTG: nitroglicerina; PA: pressão arterial; PAD: pressão arterial diastólica; PAS: pressão arterial sistólica; PE: pré-eclâmpsia; SCA: síndrome coronariana aguda; UTI: unidade de terapia intensiva.

## 12. Hipertensão Arterial Resistente e Refratária

### 12.1. Definição e classificação

Define-se a HAR como a PA de consultório que permanece com valores acima das metas propostas para o paciente, com o uso de três ou mais classes de medicamentos anti-hipertensivos com ações sinérgicas, em doses máximas preconizadas ou toleradas, sendo eles, preferencialmente, um diurético tiazídico ou tiazídico similar, um inibidor do SRAA (IECA ou BRA) e BCC de ação prolongada, salvo em condições especiais, por pelo menos 30 a 60 dias; e tendo sido afastadas as causas de pseudorresistência. Também são considerados resistentes, os pacientes em uso de quatro ou mais anti-hipertensivos e que alcançaram o controle da PA (Figura 12.1).^516,517^

**Figura 12.1:**
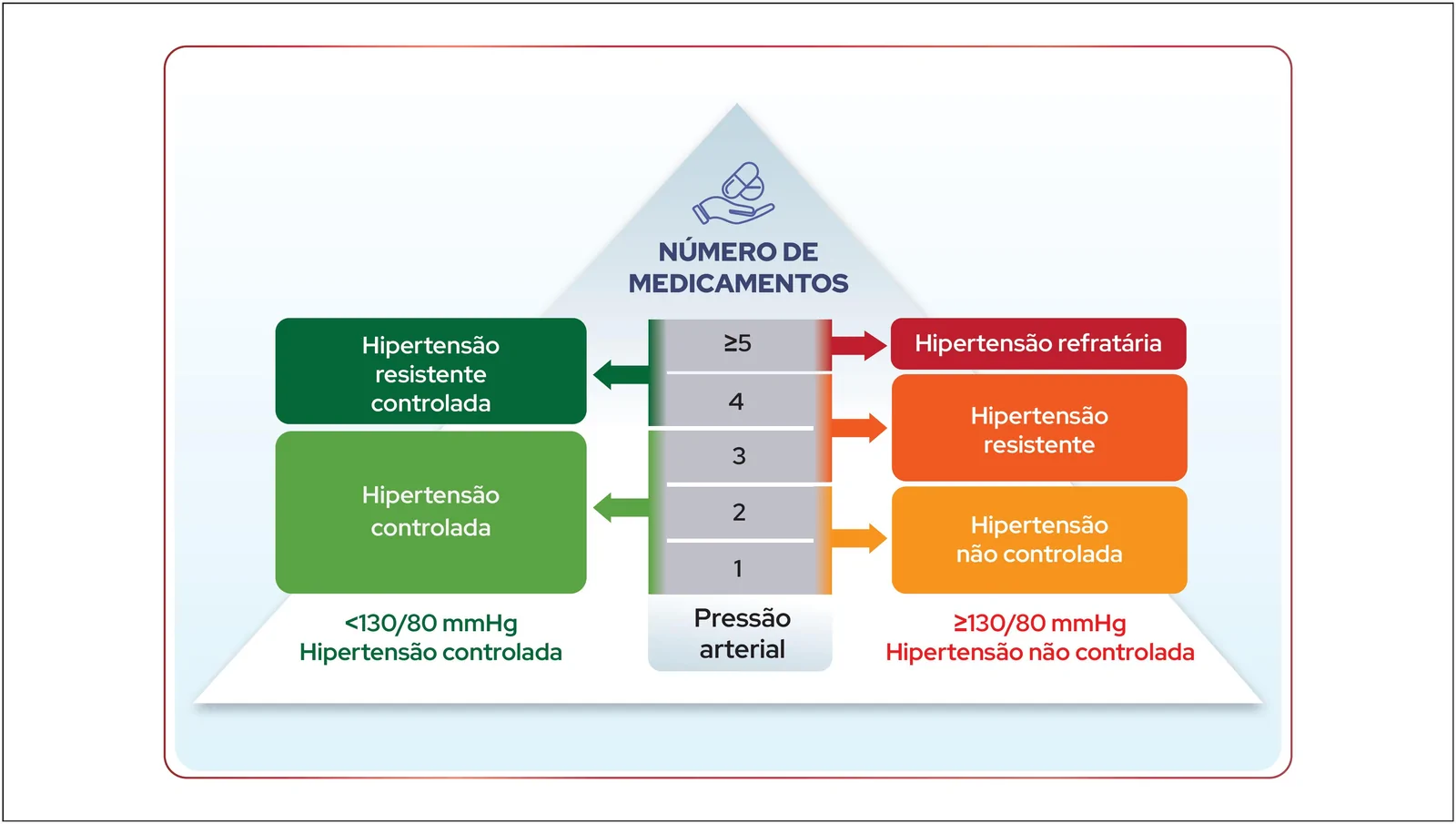
Classificação da hipertensão arterial resistente e refratária baseada no número de medicamentos e controle da pressão arterial.

A HARf é definida como um subgrupo de pacientes com HAR verdadeira que mantém a PA não controlada (acima das metas propostas individuais), mesmo estando em uso de cinco ou mais anti-hipertensivos, incluindo, preferencialmente, um ARM (preferencialmente a espironolactona ou a eplerenona, em caso de efeitos colaterais hormonais) e um diurético tiazídico similar de ação prolongada (CTD ou indapamida) (Figura 12.1).^517-519^

### 12.2. Epidemiologia da Hipertensão Arterial Resistente e Refratária

A prevalência de HAR é variável por uma série de fatores: (1) o ambiente clínico (estudo populacional, centro de referência terciário etc.); (2) classes e doses otimizadas dos anti-hipertensivos prescritos; (3) avaliação prévia da adesão terapêutica; (4) medida correta da PA; e (5) alvo terapêutico das diferentes diretrizes.

Em estudos populacionais, a prevalência de HAR está estimada entre 6 e 18% da população hipertensa.^1,517,520^ Uma grande metanálise mostrou uma prevalência de 10,3% (IC 95% 7,6–13,2%) de HAR verdadeira e 10,3% (IC 95% 6,0–15,5%) de HA pseudorresistente.^520^ No Brasil, o estudo multicêntrico ReHOT (*Resistant Hypertension Optimal Treatment*) encontrou prevalência de 11,7% de HAR.^259^ A prevalência de HARf entre os pacientes com HAR varia de 3,6 a 17,6% em diferentes estudos, já que a sua definição vem sendo construída nos últimos 12 anos.^518,521-523^

### 12.3. Fisiopatologia

Evidências acumuladas nos últimos anos sugerem que o desenvolvimento da HAR é multifatorial e envolve interação entre o SNC, rins, glândulas suprarrenais, coração e barorreceptores. Há intrincada interação envolvendo hiperatividade simpática e SRAA, controle renal de sódio, volemia, remodelamento arterial, disfunção endotelial, sistema adrenocortical, fatores genéticos, ambientais, psicossociais e ancestralidade. Contudo, a compreensão integral desses mecanismos fisiopatológicos permanece incompleta.^516,517,524,525^

A HAR é caracterizada, principalmente, pela hiperatividade do SRAA com consequente retenção hidrossalina em resposta ao *status* de hiperaldosteronismo, que se reflete nos níveis elevados de aldosterona com atividade de renina suprimida, mais exacerbado em indivíduos da raça negra, obesos e com maior consumo de sal.^516,526-528^ Por sua vez, a HARf é menos volume-dependente, já que, por definição, esses pacientes estão em uso de um duplo bloqueio diurético potente.^525^ Assim, a HARf está mais fortemente associada à hiperatividade simpática evidenciada pelo aumento de vários marcadores como FC, excreção de norepinefrina de 24 horas e RVP, conforme a Figura 12.2.^516^

**Figura 12.2:**
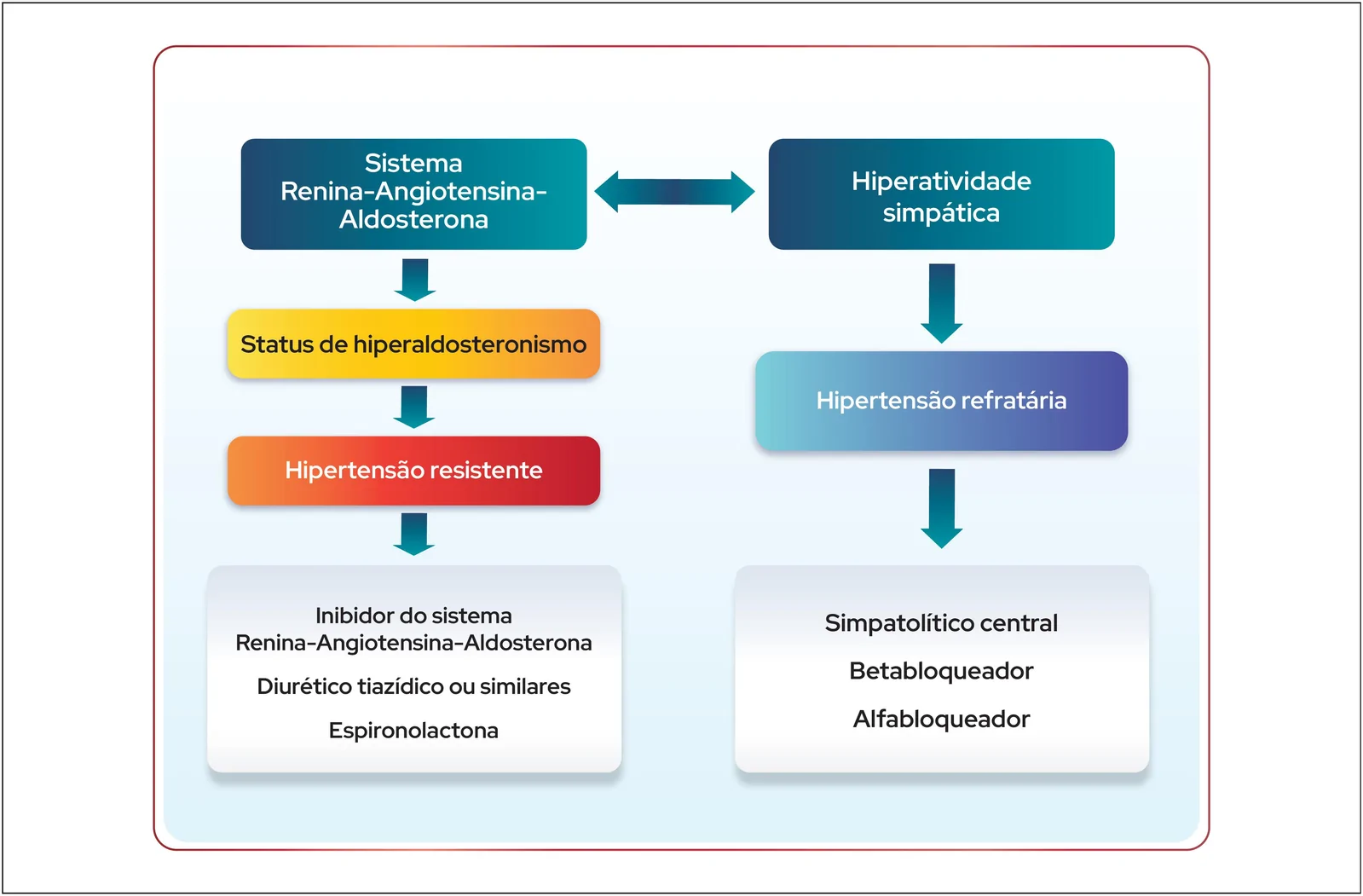
Fisiopatologia da hipertensão arterial resistente e da hipertensão arterial refratária e sua relação com a terapêutica medicamentosa proposta.

### 12.4. Avaliação Diagnóstica

O diagnóstico de HAR requer a exclusão de situações que envolvem a pseudorresistência, estimada em 50% dos pacientes com diagnóstico inicial de HAR (Figura 12.3).^516,517,529,530^

**Figura 12.3:**
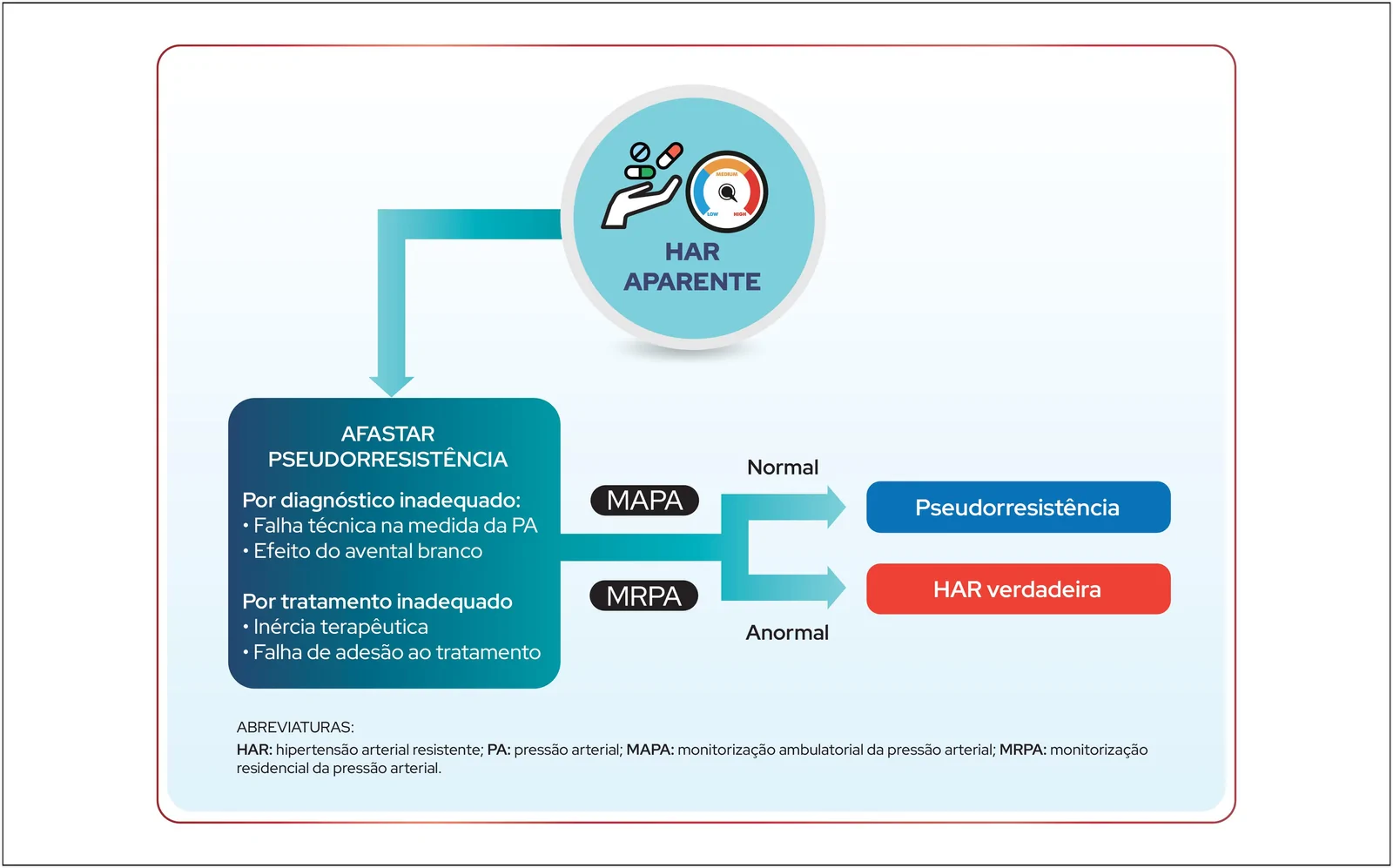
Causas de pseudorresistência.

#### 12.4.1. Falha na Técnica de Medida da Pressão Arterial

Utilização do manguito com tamanho adequado à circunferência do braço, especialmente em obesos, e cuidado com a medida em idosos com rigidez arterial expressiva, evitando superestimar os valores da PA.^516,517,530,531^

#### 12.4.2. Efeito do Avental Branco

O efeito do avental branco pode induzir erroneamente ao diagnóstico de HAR. A medida da PA fora do consultório, MRPA ou MAPA, é ferramenta mandatória para o diagnóstico e o acompanhamento de pacientes com HAR. A MAPA é preferencial em relação à MRPA, pelas medidas da PA no sono, importantes para o diagnóstico, para um controle mais rigoroso da PA, além de ser um forte marcador prognóstico.^530-534^

#### 12.4.3. Inércia Terapêutica

Inércia terapêutica é o início tardio do tratamento, a prescrição de subdoses, esquemas terapêuticos inadequados e o atraso na reavaliação das metas com consequente falta de ajuste na prescrição.

#### 12.4.4. Falta de Adesão Medicamentosa

O tratamento medicamentoso da HAR requer, por definição, uma polifarmácia, e essa necessidade diminui a chance de adesão, pois ela é inversamente proporcional ao número de comprimidos prescritos, sendo a principal causa de pseudorresistência. Dessa forma, as combinações de doses fixas estão indicadas, sempre que possível, visando a melhor adesão.^516,517,535^

### 12.5. Abordagem Clínica

A HAR é um fenótipo associado a maior risco de eventos CV e morte.^532,536^ Confirmada a HAR verdadeira, com um esquema anti-hipertensivo adequado por no mínimo 30 dias, recomenda-se a seguinte investigação diagnóstica (Figura 12.4):^1,517,530-532,537^

- Investigação de fatores de risco: sexo e idade, história familiar de DCV prematura, obesidade, obesidade central, tabagismo, alterações do perfil glicídico e lipídico, determinantes sociais de saúde, transtornos emocionais;
- Avaliação de lesões subclínicas de órgãos-alvo: ITB, rigidez aórtica (pressão central e VOP), HVE, albuminúria, DRC Estágio 3 e doença carotídea (USG de carótidas);
- Avaliação da presença de DCV e DRC estabelecidas: DAC, doença cerebrovascular, IC, DAOP, retinopatia hipertensiva e DRC Estágios 4 ou 5;
- Investigação de hipertensão secundária: hiperaldosteronismo, doença renovascular, apneia obstrutiva do sono (AOS). A investigação da AOS é recomendada para os pacientes com hipertensão resistente, devido à sua alta prevalência nesses pacientes, que pode chegar a 80%.^538,539^

**Figura 12.4:**
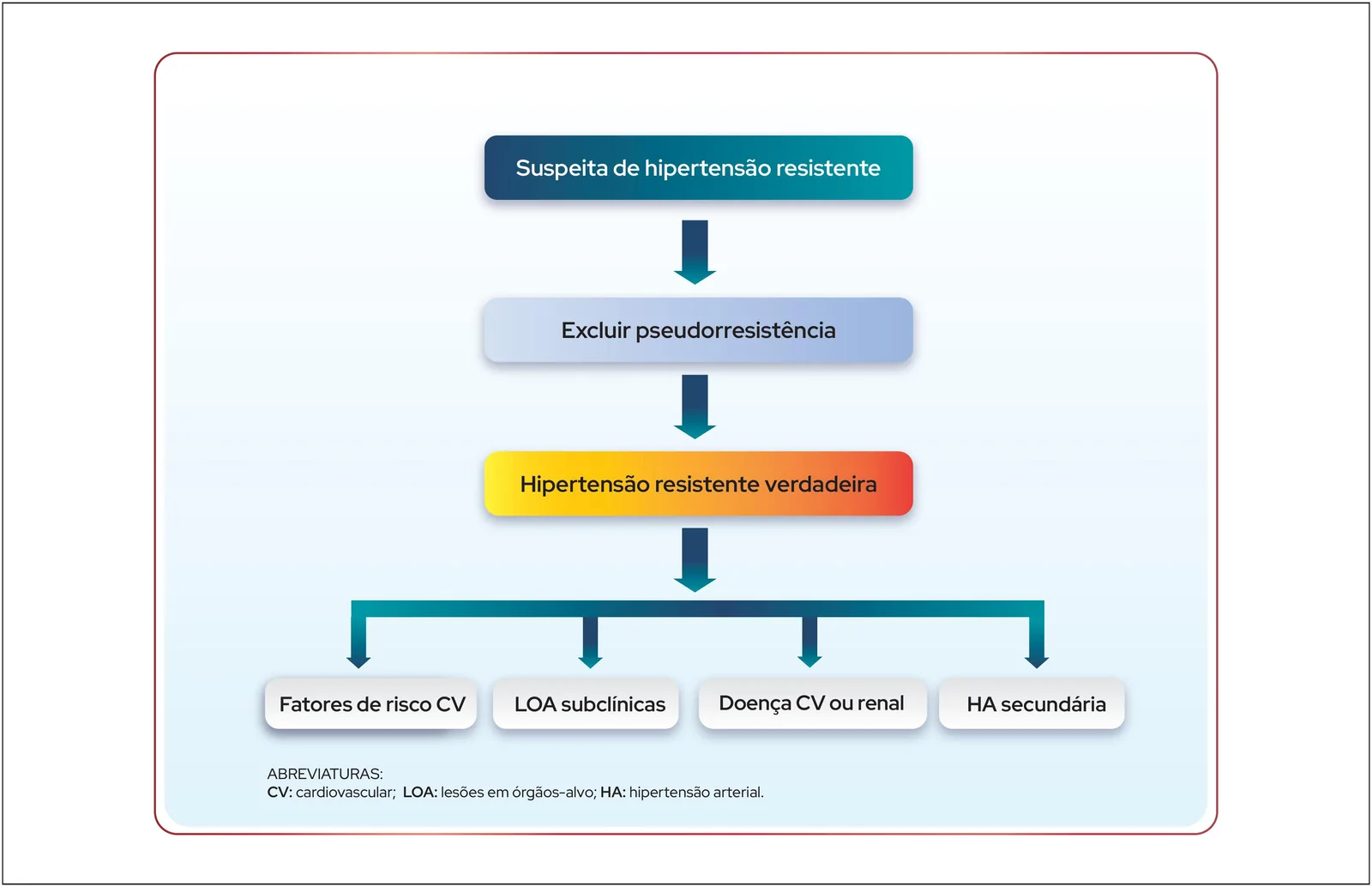
Abordagem diagnóstica da hipertensão arterial resistente

A verificação de um estilo de vida saudável deve fazer parte da abordagem clínica, porque sua implementação reduz a necessidade de tratamento medicamentoso e diminui o risco CV. Ênfase deve ser dada em relação ao consumo de sódio e álcool e ao combate à obesidade, sedentarismo, tabagismo e ao uso de drogas ilícitas.^517,532^

A abordagem clínica de pacientes com HAR é dinâmica e necessita de uma vigilância constante, com o manejo clínico individualizado e ciclos de avaliação-intervenção-reavaliação frequentes em relação à adesão medicamentosa, estilo de vida, LOAs e otimização medicamentosa.^519,536^ É fundamental, no processo de triagem e identificação desses pacientes, a confirmação do binômio que define a HAR: a falta de controle da PA e o tratamento adequado. Esses pacientes devem ser acompanhados em serviços especializados em HA capazes de oferecer uma abordagem multidisciplinar com profissionais capacitados e recursos diagnósticos necessários.^531,540^

### 12.6. Tratamento

#### 12.6.1. Tratamento Não Medicamentoso

Assim como em todos os pacientes com HA, as MNM precisam ser implementadas, com destaque para a redução do consumo de sódio,^541^ alcance de peso corporal normal, cessação do tabagismo, prática regular de exercícios físicos e controle de estresse.^1,532^

#### 12.6.2. Tratamento Medicamentoso

O uso de pelo menos três diferentes classes de anti-hipertensivos com mecanismos farmacológicos complementares está recomendado. Habitualmente, é preferida a combinação de um bloqueador do SRAA (IECA ou BRA), associado a um BCC di-hidropiridínico de ação prolongada e um diurético tiazídico, se possível, em associações fixas.^45^ A substituição do diurético tiazídico de curta duração (HCTZ) por um diurético tiazídico similar de longa ação, como a CTD ou a indapamida, pode ser considerada, de acordo com ECRs que mostraram maior eficácia anti-hipertensiva e menor incidência de efeitos adversos sobre o perfil lipídico, ácido úrico e potássio sérico observados em estudos retrospectivos.^1,542^ No entanto, o tema exibe ainda alguma controvérsia, após estudo de comparação direta entre HCTZ e CTD.^543^

Após 30 a 60 dias de uso regular desse esquema terapêutico, o paciente deve ser submetido à MAPA (preferencial) ou MRPA.^533,534^ A MAPA/MRPA não controlada define o diagnóstico de HAR verdadeira, quando está indicada a prescrição do 4º medicamento, preferencialmente a espironolactona, de 25 a 50 mg/dia (*status* de hiperaldosteronismo).^517,259,544^ O efeito adverso mais comum é a ginecomastia, podendo ser reduzida a dose da espironolactona para 12,5 mg/dia ou substituída pela eplerenona, que não tem efeitos hormonais, porém tem menor potência anti-hipertensiva.^545^ Outros efeitos adversos são a hipercalemia e a deterioração da função renal, particularmente em pacientes com DRC em uso de bloqueadores do SRAA.

A espironolactona deve ser usada de forma cautelosa em indivíduos com TFGe < 30 mL/min/1,73 m^2^ e concentração plasmática de potássio > 5,5 mEq/L, algumas vezes, sendo necessário substituir por outro anti-hipertensivo em hiperpotassemia refratária ao tratamento. O potássio plasmático e a TFGe devem ser monitorados, em intervalos de pelo menos 3 a 6 meses.^45,517^ Quando a espironolactona e outros ARM não são tolerados ou estão contraindicados, as alternativas incluem os simpaticolíticos^259,544^ ou a amilorida na dose de 10 a 20 mg/dia, que foi considerada tão eficaz quanto a espironolactona.^1,516,517^ O ECR de Lee *et al*.^546^ mostrou que a amilorida (até 10 mg/dia) foi não inferior à espironolactona (até 25 mg/dia) na redução da PAS domiciliar em pacientes com HAR já em uso de tripla terapia padrão. As taxas de controle da PA (< 130 mmHg) foram semelhantes entre os grupos, tanto em medidas domiciliares quanto em consultório. Contudo, em nosso meio, existe apenas a amilorida nas doses de 2,5 e 5 mg em associação com HCTZ ou CTD.

Com base na fisiopatologia, pacientes em uso de quatro classes de medicamentos, incluindo o duplo bloqueio diurético com um tiazídico e um ARM, teoricamente não apresentam mais o componente de hipervolemia e o *status* de hiperaldosteronismo como fatores causais do descontrole pressórico. Se a PA ainda se mantiver acima das metas, o próximo passo é reduzir a hiperatividade simpática com o uso de simpaticolíticos, como um alfa agonista central, como a clonidina, alfabloqueadores, como a doxazosina, ou BBs, preferencialmente o bisoprolol, o carvedilol ou nebivolol. Bisoprolol e nebivolol são bastante seletivos para receptor β1. Carvedilol e nebivolol têm efeito vasodilatador e não causam alterações no perfil metabólico^1,259,517,544,545^ (Figura 12.5).

**Figura 12.5:**
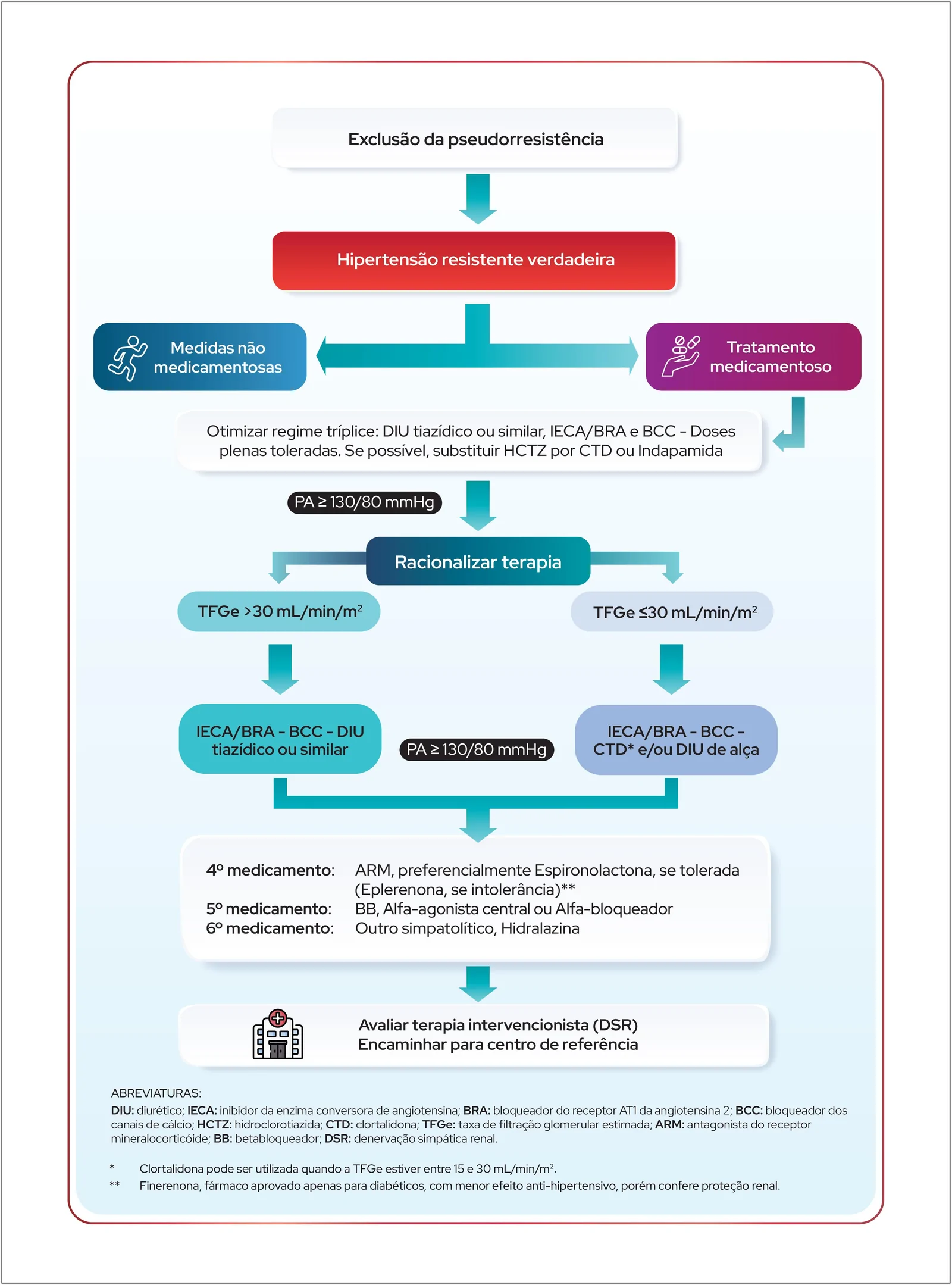
Fluxograma do tratamento.

Em alguns casos individualizados, algumas modificações no esquema terapêutico devem ser consideradas:^547^

- Considerar a substituição do diurético tiazídico por um diurético de alça para pacientes com TFGe < 30 mL/min/1,73 m^2^, sendo possível também incluir o CTD quando a TFGe se encontra entre 15 e 30 mL/min/1,73 m^2^);^548^
- Avaliar criteriosamente situações clínicas específicas, como FA, IC ou DRC, em que a terapia tripla inicial pode demandar o uso de classes de medicamentos diferentes. Em alguns casos, poderá existir indicação para introdução do BB no esquema tríplice inicial (ver Capítulo 7);^1,45,517^
- Nos casos de intolerância aos BCC por efeitos colaterais, como edema de membros inferiores (MMII), que pode ser uma causa de abandono do tratamento e falha no controle da PA, pode ser feita a prescrição de BCC como manidipina, lercanidipina ou levanlodipina ou, em casos criteriosamente selecionados, um BCC não di-hidropiridínico, como diltiazem e verapamil;^1,45,517^
- Na impossibilidade de uso de um BCC, pode ser considerada a introdução de um BB, preferencialmente com ação vasodilatadora e que não altera o perfil metabólico, como nebivolol ou carvedilol;^1,45,517^
- Baixas doses de furosemida em associação a CTD e espironolactona (bloqueio sequencial do néfron) em pacientes com TFGe > 30 mL/min/1,73 m^2^ foram testados em estudos randomizados com resultados promissores.^549^

#### 12.6.3. Perspectivas

Novos medicamentos vêm sendo pesquisados e desenvolvidos e podem ter um papel no tratamento da HAR e HARf. Eles incluem antagonistas da endotelina, inibidores da aldosterona sintase, novos ARM e inibidores da produção de angiotensinogênio pelo fígado (agente terapêutico de interferência de RNA), bem como peptídeos natriuréticos atriais e inibidores da aminopeptidase A, conforme pode-se observar no Quadro 15.2 do Capítulo 15,^236,530,550-557^ mas certamente, algumas lacunas permanecem a serem respondidas no cenário da HAR e HARf.

#### 12.6.4. Denervação Renal

A denervação simpática renal (DSR) é a ablação por radiofrequência ou por ultrassom da inervação simpática da artéria renal, com consequente aumento do fluxo sanguíneo renal, inibição do SRAA, inibição da ação simpática sobre o coração e os vasos através dos sinais cerebrais.^558^ Os resultados são bastante controversos, o que é atribuído à falta de protocolos homogêneos e bem definidos para o procedimento.

Os dois grandes estudos que incluíram pacientes com hipertensão resistente foram o Radiance-HTN Trio (ablação por ultrassom) e o Simplicity HTN-3 (ablação por radiofrequência). Este último incluiu 535 participantes (171 no grupo *sham*) e não encontrou redução da PA de 24 horas na comparação com o grupo-controle (-2,0 mmHg; IC 95% –5,0 a 1,1, p = 0,98).^559^ Por sua vez, o Radiance-HTN Trio encontrou uma redução de −6,3 mmHg (IC 95% –9,3 a –3,2, p < 0,0001).^560^ Uma metanálise recente que avaliou pacientes com HA não controlada (não obrigatoriamente pacientes com HAR) incluiu 15 ensaios clínicos com um total de 2.581 pacientes (1.723 para denervação e 858 no grupo *sham*), encontrou uma redução da PAS de 24 horas de –2,23 mmHg (IC 95% –3,56 a –0,90; p = 0,001), de consultório de −6,39 (IC 95% −11,49 a −1,30; p = 0,01) e residencial de −6,08 mmHg (IC 95% −11,54 a −0,61; p = 0,03) entre aqueles em uso de anti-hipertensivos.^561^

Dessa forma, a DSR pode ser considerada um procedimento auxiliar para atingir a PA em pacientes com HAR e HARf, de forma individualizada e compartilhada com os pacientes.^45^

**Table:** 

Mensagens principais sobre hipertensão arterial resistente e refratária
HAR e HARf são fenótipos que conferem maior risco de eventos CV, renais e de morte.
Para o diagnóstico de HAR e HARf, deve-se obter a medida da PA de consultório com técnica e equipamentos corretos, além da exclusão obrigatória de pseudorresistência por MAPA (preferencialmente) ou MRPA.
Na HAR e HARf, deve-se tratar o paciente com associações de três ou mais classes sinérgicas de anti-hipertensivos, nas doses máximas toleradas.
A HAR é caracterizada, principalmente, pela hiperatividade do SRAA e status de hiperaldosteronismo. A HARf, por sua vez, tem como principal componente da fisiopatologia a hiperatividade simpática. Conhecer a fisiopatologia implica em melhores escolhas terapêuticas.
Novos medicamentos estão sendo testados com resultados promissores (ver Capítulo 15), e o uso de DSR para atingimento de meta de PA ainda é controverso.

HAR: hipertensão arterial resistente; HARf: hipertensão arterial refratária; CV: cardiovascular; MAPA: medida ambulatorialda da pressão arterial; MRPA: medida residencial da pressão arterial; SRAA: renina-angiotensina-aldosterona; DSR: denervação simpática renal; PA: pressão arterial.

**Table:** 

Recomendações para abordagem da hipertensão arterial resistente e refratária	Força de recomendação	Certeza da evidência
Recomenda-se, para pacientes com HAR e HARf, o acompanhamento em serviços especializados em HA capazes de oferecer uma abordagem multidisciplinar com profissionais capacitados e recursos diagnósticos necessários.	FRACA	BAIXA
Recomenda-se a investigação de FRCV, LOA e causas secundárias de HA em pacientes com HAR e HARf.	FORTE	ALTA
Recomenda-se a realização preferencial de MAPA para a confirmação diagnóstica de HAR e HARf, se disponível, ou MRPA.	FORTE	MODERADA
Recomenda-se a meta de PA < 130/80 mmHg para pacientes com HAR ou HARf.	FRACA	BAIXA
Recomenda-se a associação de medicamentos com um bloqueador do SRAA (IECA ou BRA), um BCC e um diurético tiazídico, preferencialmente em comprimido único.	FORTE	ALTA
Recomenda-se a substituição do diurético tiazídico de curta duração (hidroclorotiazida) por um diurético tiazídico-símile de longa ação, como a clortalidona ou a indapamida.	FRACA	BAIXA
Recomenda-se que, havendo efeitos colaterais hormonais com a espironolactona, a substituição possa ser realizada por eplerenona.	FRACA	ALTA
Recomenda-se que, em caso de manutenção de níveis de PA acima da meta com quatro medicamentos, o quinto objetive a redução da hiperatividade simpática: simpatolíticos (alfa agonista central como a clonidina; alfabloqueadores como a doxazosina; ou BB, preferencialmente o bisoprolol, o carvedilol ou o nebivolol).	FRACA	BAIXA
Recomenda-se avaliar criteriosamente situações clínicas específicas, como FA, IC ou DRC, em que a terapia tripla inicial pode demandar o uso de classes de medicamentos diferentes, como as indicações específicas para introdução do BB no esquema tríplice inicial.	FORTE	MODERADA
Recomenda-se a DSR como procedimento auxiliar para atingir a meta pressórica em pacientes com HAR e HARf, de forma individualizada e compartilhada com os pacientes.	FRACA	BAIXA

BB: betabloqueador; BCC: bloqueador dos canais de cálcio; BRA: bloqueador dos receptores da angiotensina; DRC: doença renal crônica; DSR: denervação simpática renal; FA: fibrilação atrial; FRCV: fator de risco cardiovascular; HA: hipertensão arterial; HAR: hipertensão arterial resistente; HARf: hipertensão arterial refratária; IC: insuficiência cardíaca; IECA: inibidor da enzima conversora da angiotensina; LOA: lesão de órgãos-alvo; MAPA: medida ambulatorial da pressão arterial; MRPA: medida residencial da pressão arterial; PA: pressão arterial; SRAA: sistema renina-angiotensina-aldosterona.

## 13. Adesão ao Tratamento Anti-Hipertensivo

A adesão ao tratamento e a persistência às recomendações terapêuticas são questões críticas no manejo da HA, afetando os desfechos clínicos e os custos em saúde.^8^ A adesão ao tratamento anti-hipertensivo engloba o cumprimento pelo paciente das prescrições e recomendações dos médicos e demais profissionais de saúde quanto às medidas terapêuticas não medicamentosas e medicamentosas.^8,562^ Nesse contexto, deve-se também considerar a persistência no tratamento, definida como a duração do tempo desde o início do uso até a descontinuação da terapia.^563^

Estudos indicam que uma proporção substancial de pacientes com doenças crônicas cardiovasculares não adere aos tratamentos prescritos, quer sejam medicamentosos ou não. Uma publicação da OMS revelou que aproximadamente 50% dos pacientes com doenças crônicas não fazem uso de seus medicamentos conforme prescritos, levando ao aumento de hospitalizações, morbidade e mortalidade.^43^ Um estudo que avaliou especificamente pacientes em tratamento de DCVs revelou que a baixa adesão poderia atingir 40 a 60% deles, particularmente entre aqueles com regimes de polifarmácia.^564^

### 13.1. Mensuração da Adesão

A mensuração da adesão às recomendações de tratamento tanto medicamentoso quanto não medicamentoso é complexa, envolvendo abordagens diretas e indiretas. Os métodos diretos envolvem, principalmente, a quantificação dos níveis do(s) medicamentos(s) ou seus metabólitos no sangue ou na urina, fornecendo evidências concretas da ingestão, mas são, muitas vezes, impraticáveis para o monitoramento de rotina.^565^ Os métodos indiretos são mais comumente utilizados e incluem autorrelatos dos pacientes, contagem de medicamentos, dados de reabastecimento de farmácias e sistemas de monitoramento eletrônico, sendo estes últimos capazes de registrar a data e a hora em que o recipiente do medicamento é aberto.^566^

Cada método tem vantagens e limitações. Por exemplo, os autorrelatos são simples e de baixo custo, mas podem ser tendenciosos devido ao desejo dos pacientes de se mostrarem aderentes. A contagem de medicamentos também é fácil de implementar, mas pode ser manipulada pelos pacientes. Os registros de reabastecimento de farmácias fornecem uma medida objetiva da continuidade do fornecimento do medicamento, mas não confirmam a ingestão real. O monitoramento eletrônico é considerado o padrão-ouro entre os métodos indiretos devido à sua maior precisão em capturar padrões de dosagem ao longo do tempo, mas é mais oneroso, sujeito a vieses caso a embalagem seja aberta sem que o medicamento tenha sido efetivamente tomado, podendo ainda ser interpretado como intrusivo. A combinação desses métodos é frequentemente recomendada para fornecer uma avaliação mais abrangente da adesão tanto em ambientes clínicos quanto na pesquisa.^566^

A adesão ao tratamento medicamentoso é frequentemente quantificada usando vários pontos de corte e categorizada em níveis distintos, favorecendo o entendimento do comportamento do paciente e a eficácia do tratamento. Comumente, a "alta adesão" é definida como aderir em 80% ou mais às recomendações, a "adesão média" é quando esse comportamento está entre 50 e 79%, e a "baixa adesão" é quando o cumprimento às prescrições é de menos de 50%.^566^

A OMS indica que níveis de adesão acima de 80% são geralmente necessários para alcançar resultados terapêuticos ideais na maioria das condições crônicas.^43^ No entanto, a grande variabilidade individual e fenotípica dos pacientes com HA pode determinar diferentes necessidades de adesão mínima ao tratamento para a efetiva prevenção de desfechos clínicos.

### 13.2. Evidências

Apesar das robustas evidências de que o controle da HA promove significativas reduções de mortes e eventos CV e renais, tanto em prevenção primária quanto secundária, há ainda demonstrações das relações entre a adesão ao tratamento anti-hipertensivo e a diminuição de desfechos clínicos relevantes.^120,567-570^

Liu *et al*. avaliaram a associação entre a adesão às principais medicações de ação CV e o risco de eventos CV, AVC e mortalidade por todas as causas, tanto em prevenção primária quanto secundária.^571^ Mais de 4 milhões de pacientes distribuídos em 46 estudos observacionais foram incluídos na análise, com a avaliação da qualidade usando a pontuação média da Escala Newcastle–Ottawa de 7,9 (máximo de 9) e um período médio de acompanhamento de 4,6 anos.^569^ Quanto à medicação anti-hipertensiva, a análise de dose-resposta indicou que um aumento de 20% na adesão foi associado a uma redução de 17% (0,83 [0,78–0,89]) no RR de AVC e de 12% no RR de mortes (0,88 [0,82–0,94]). Com base na qualidade geralmente satisfatória dos estudos primários, e sendo o único documento que avaliou a relação dose-dependente entre adesão e a redução de eventos CV, essa revisão promoveu uma certeza moderada de evidência de acordo com o sistema GRADE).^571^

Lee *et al*., em outra RS, estimaram a prevalência global e as consequências da baixa adesão aos medicamentos anti-hipertensivos entre pacientes adultos com HA. A análise incluiu vários métodos de mensuração da adesão ao tratamento e envolveu 161 estudos observacionais. Os resultados indicaram que a baixa adesão aos anti-hipertensivos estava associada a um aumento da razão de chance (OR) de morte de 1,38 (IC 95% 1,35–1,41). Uma melhor adesão à medicação anti-hipertensiva foi associada a uma redução de 25% (0,75 [0,73–0,76]) no RR de morte. No entanto, esses resultados foram baseados em apenas dois estudos com 1.653.763 pacientes e um seguimento médio de 4,5 anos.^572^

### 13.3. Estratégias

Uma publicação de uma visão geral de várias revisões sistemática avaliou as evidências de efetividade das mais diferentes estratégias empregadas para aumentar a adesão ao tratamento da HA (Tabela 13.1). Entre as estratégias analisadas, o estudo revelou significativos efeitos positivos de intervenções relativas à atenção farmacêutica, automonitoramento e uso de aplicativos de celular e SMS, que podem ser aplicadas no contexto da atenção primária à saúde para melhorar a adesão ao tratamento de adultos com HA. Em revisão da Cochrane, há evidências de baixa certeza sobre os efeitos das intervenções realizadas por telefone celular para aumentar a adesão aos medicamentos prescritos para a prevenção primária de DCV e há evidências de certeza moderada de que essas intervenções não resultam em danos.^573^

**Tabela 13.1:** Resultados alcançados na adesão aos medicamentos para hipertensão arterial sistêmica por meio de estratégias envolvendo profissionais farmacêuticos, outros profissionais ou aplicativos e subsídios

Intervenção	Resultado
Atuação de farmacêuticos	
Farmacêutico clínico na equipe	Aumento da adesão em 45,4
Serviços de cuidados farmacêuticos	Melhora na continuidade e adesão
Aconselhamento e lembretes telefônicos	Aumento da adesão
Ações combinadas de gerenciamento de medicamentos, educação, consultas/contatos de acompanhamento	Aumento da adesão em 67%
Atuação de outros profissionais	
Relaxamento em grupo; cartilhas de educação + contratos com metas comportamentais e recompensa	Melhor adesão; menos interrupção de tratamento
Intervenções intensivas/ aconselhamento por ACS	Aumento da adesão em 26% e 17%, respectivamente
Treinamento grupal, educação e telefonemas	• Aumento de 16% na adesão com treinamento, e de 26,4% com ações educativas• Redução de 3% na interrupção de medicamentos, com telefonemas
Embalagem blister, gestão de caso, educação presencial, educação com apoio comportamental	Benefício com a intervenção e cuidado colaborativo
Automonitoramento, aplicativos de celular e mensagens de texto	
Automonitoramento da pressão arterial	• Efeito na adesão (DMP = 0,21; IC 95% 0,08 a 0,34; I^2^= 43%) • Sem efeito significativo na adesão de pacientes com uso de dados de registro de farmáci
*eHealth,* aplicativos, telefonemas	Melhor adesão • *eHealth* (RR = 0,79; IC 95%: 0,48; 1,01; *I*^2^= 91,3%) • Aplicativo (RR = 0,55; IC 95%: 0,33; 0,93; *P* = 0,004) • Telefonemas (RR= 0,44; IC 95%: 0,09; 2,13; *I*^2^= 90,7) • Telemonitoramento da pressão arterial (RR = 0,99; IC 95%: 0,92; 1,05; *I*^2^ = 44,2) • e-mails (RR = 0,26; IC 95%: 0,11; 0,61) • Website não teve efeito na adesão (RR= 1,01; IC 95%: 0,84; 1,22; *I*^2^= 0)
*mHealth*	Melhor adesão, embora sem significância estatística
App	Melhor adesão
App	Melhora da pressão arterial e adesão à medicação
Monitorização domiciliar da pressão arterial	Adesão similar entre os grupos em 8 semanas
SMS semanal com lembretes, relatório de automonitoramento, registro de compromissos	Não mostraram benefício
Subsídios para aquisição de medicamentos	
Subsídios	Oferta de cobertura completa de anti-hipertensivos mostrou aumento absoluto de 9% na adesão (p = 0,0340)

ACS: agente comunitário de saúde; app: aplicativos de celular; DMP: diferença de média padronizada; RR: risco relativo. Adaptado de: da Silva et al.^576^

Em outra revisão, a redução do número de doses diárias parece ser eficaz no aumento da adesão à medicação para baixar a PA e deve ser tentada como uma estratégia de primeira linha, embora haja menos evidências de um efeito na redução da PA.^574^ É fundamental a atuação de equipes multiprofissionais para a melhora da adesão ao tratamento da HA.^575^ Essas intervenções devem ser implementadas de forma única ou combinada, de acordo com os contextos locais.

As barreiras à implementação das intervenções identificadas como significativas incluíram a baixa literacia digital, o acesso limitado às tecnologias, o custo dos medicamentos e a condição de saúde dos pacientes; e, quanto aos profissionais de saúde, incluíram a falta de integração ao fluxo de trabalho, a falta de recursos humanos, o treinamento incipiente e sistema eletrônico de difícil manuseio. Os significativos facilitadores da adesão foram relacionados a fatores socioeconômicos, boa relação entre paciente e serviços/profissionais de saúde e o bom nível dos trabalhadores da saúde. Quanto ao sistema de saúde, o principal facilitador foi a melhoria do acesso aos serviços de saúde.^576^

### 13.4. Recomendações

A OMS elenca cinco grupos de fatores que impactam na adesão ao tratamento da HA, sendo estes relacionados ao paciente, à doença, à terapia, ao sistema de saúde e socioeconômicos.^43^ As recomendações de estratégias para melhorar a adesão ao tratamento da HA, assim como as dificuldades para a sua implementação, adaptadas e modificadas da OMS, estão resumidas na Figura 13.1.

**Figura 13.1:**
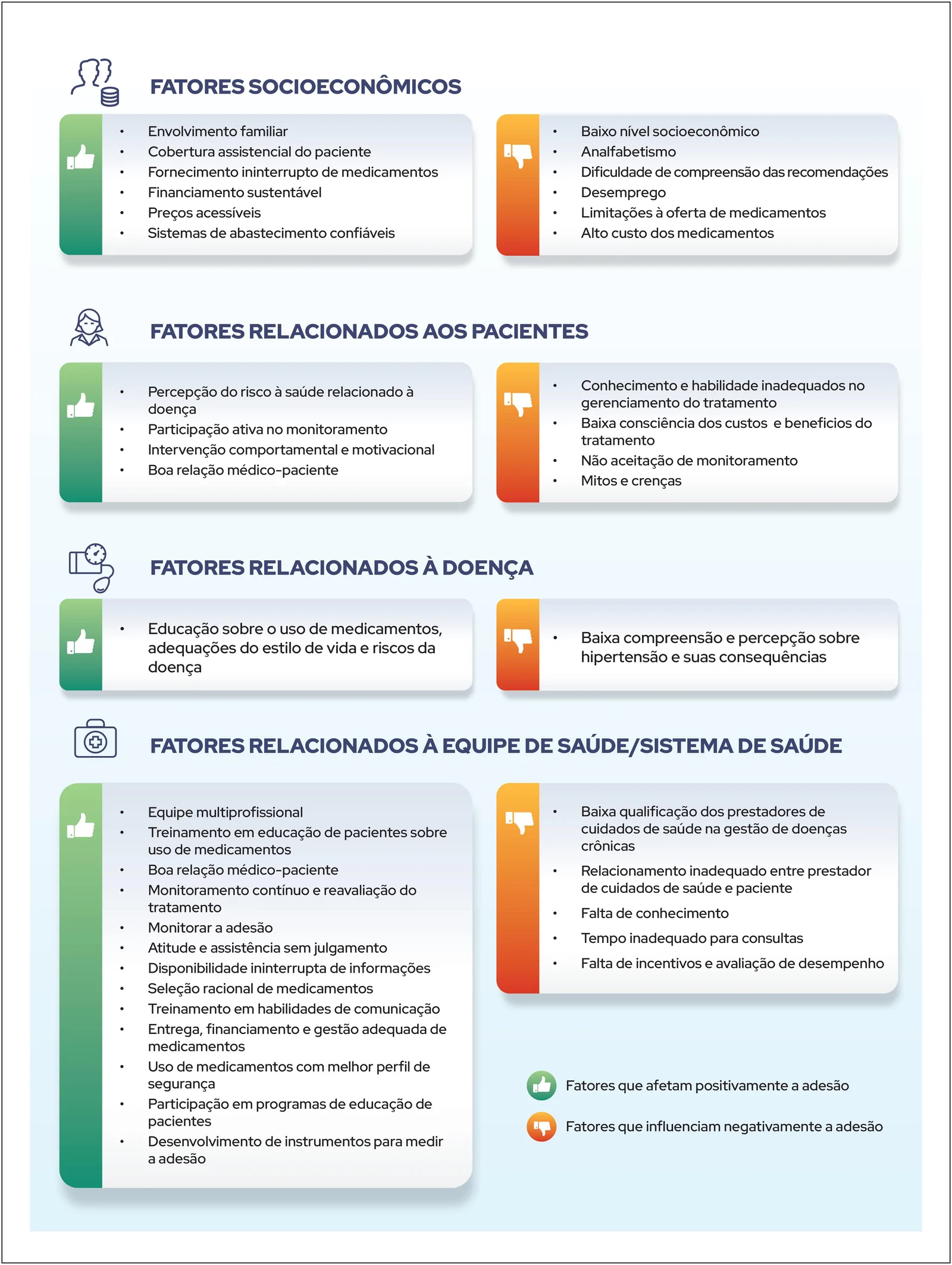
Fatores que influenciam negativamente e positivamente na adesão ao tratamento da hipertensão arterial. Adaptado e modificado da OMS.^43^

**Table:** 

Mensagens principais sobre adesão na hipertensão arterial
O aumento na adesão ao tratamento medicamentoso da HA reduz o risco de desfechos CV, renais e de morte por todas as causas.
Uma melhoria de 20% na adesão aos medicamentos anti-hipertensivos reduz o RR de AVC em 17% e de morte por todas as causas em 12%.

**Table:** 

Recomendação para adesão tratamento anti-hipertensivo	Força da recomendação	Certeza da evidência
Recomendam-se estratégias envolvendo a atuação de equipe multiprofissional, subsídios, recursos de comunicação/educação e aplicativos para promover melhorias na adesão ao tratamento anti-hipertensivo.	FRACA	BAIXA

## 14. Melhores Práticas no Cuidado às Pessoas Com Hipertensão Arterial na Atenção Primária à Saúde do Sistema Único de Saúde

### 14.1. Sistema Único de Saúde e sua Importância no Controle da Hipertensão Arterial

As Unidades Básicas de Saúde (UBSs) são parte integrante do SUS, regulamentadas por leis e normativas federais que orientam sua estruturação e funcionamento. São centros de atenção primária à saúde (APS), onde equipes de Saúde da Família (ESF) realizam uma série de ações, oferecendo atendimento, incluindo prevenção, diagnóstico e tratamento. Elas representam a principal porta de entrada para o sistema, atendendo a necessidades individuais e coletivas.^577^

A Lei nº 8.080, de 19/09/1990, dispõe sobre as condições para a promoção, proteção e recuperação da saúde, a organização e o funcionamento dos serviços correspondentes, instituindo o SUS.^577^ Com mais de 30 anos de existência, o SUS, maior sistema público de saúde do mundo, atende a mais de 190 milhões de pessoas por ano, sempre de forma integral e gratuita, com complexidade tal que necessita ser organizado em diferentes níveis de atenção e assistência à saúde para ter um bom funcionamento. Os níveis de atenção e assistência à saúde no Brasil são estabelecidos pela Portaria 4.279 de 30 de dezembro de 2010, que estabelece as diretrizes para a organização da Rede de Atenção à Saúde (RAS) no âmbito do SUS, sendo eles: atenção primária, secundária e terciária.^578^

Eles são usados para organizar os tratamentos e serviços oferecidos pelo SUS a partir de parâmetros determinados pela OMS, com o objetivo de proteger, restaurar e manter a saúde dos cidadãos, com equidade, qualidade e resolutividade. É na APS, porta preferencial de entrada do usuário no SUS, onde a maioria dos problemas de saúde pode ser resolvida ou encaminhada para tratamento na rede de atenção especializada (níveis secundário e terciário), caso necessário.^578^

#### 14.1.1. Atenção Primária à Saúde

As UBSs, estabelecimentos da APS, ainda conhecidos em muitos locais como "postos de saúde", realizam ações e atendimentos voltados à prevenção, promoção da saúde, diagnóstico e tratamento. Nessas unidades, é possível fazer exames, consultas de rotina com equipes multiprofissionais e profissionais especializadas em Saúde da Família, que trabalham para garantir atenção integral à saúde no território.^578^

É nesse nível que os profissionais se articulam para atuar não apenas nas unidades de saúde, como também em espaços públicos da comunidade, escolas e em visitas domiciliares, na oferta de práticas integrativas e complementares.^578^

Mais do que prover assistência clínica, o objetivo é estar próximo às pessoas e promover a saúde e a qualidade de vida da comunidade. Esse trabalho de prevenção, conscientização e educação é importante para, além de garantir qualidade de vida aos usuários, otimizar o uso de recursos, evitando doenças, internações e tratamentos de agravos à saúde, como HA, DM, sedentarismo, obesidade, dislipidemia, DCVs e renais.^578^

A busca pelo controle da HA é um dos programas estratégicos da APS, e o médico generalista/médico de família e comunidade (MFC), juntamente com a equipe de saúde desse nível de complexidade, são os responsáveis pelo paciente. Cabe ao generalista/MFC, quando necessário, fazer os encaminhamentos para os demais níveis de complexidade e recebê-los, após a avaliação do especialista, com as orientações necessárias expressas em relatório de contrarreferência consubstanciado.^578^

Atualmente, existem 48.161 UBSs no Brasil, portanto é impossível traçar estratégias para aumentar a prevenção, o diagnóstico, o tratamento e o controle da HA em nosso país sem contar com essa rede. Na APS, é possível atender a uma média de 564.232 pessoas por dia. As pessoas podem procurar a UBS mais próxima da residência para atendimentos em praticamente todas as situações, exceto naquelas em que há risco de morte, quando se deve procurar atendimento de urgência e emergência em Unidades de Pronto Atendimento (UPAs) 24 h, hospitais gerais ou serviços habilitados em média e alta complexidade.^578^

#### 14.1.2. Atenção Especializada

A atenção especializada é dividida em dois elementos (atenção secundária e terciária), que são, respectivamente, média e alta complexidade (habitualmente ambulatorial e especializada hospitalar). A média complexidade é composta por serviços especializados encontrados em hospitais e ambulatórios e envolve atendimento direcionado para áreas de cardiologia, nefrologia, endocrinologia, neurologia, entre outras especialidades médicas.^578^ As UPAs concentram os atendimentos de complexidade intermediária, com capacidade de atendimento de 150 a 450 pacientes por dia, por unidade.^578^

Além disso, o Serviço de Atendimento Móvel de Urgência (SAMU – 192), que tem como objetivo chegar precocemente à vítima após alguma situação de urgência ou emergência que possa levar a sofrimento, a sequelas ou mesmo à morte. O SAMU cobre 85,89% da população nacional, com 190 centrais de regulação.^578^

Considerando os dados acima, a APS é o local adequado para garantir a prevenção, o diagnóstico, o tratamento e o controle da HA. De acordo com a Pesquisa Nacional de Saúde de 2019,^579^ entre as pessoas de 18 anos ou mais que referiram diagnóstico médico de HA, 72,2% receberam assistência médica nos últimos 12 meses no SUS. Em 46,6% dos casos, o diagnóstico foi feito em UBS, e 45,11% obtiveram pelo menos um medicamento para HA no programa "Aqui Tem Farmácia Popular", devendo ser considerado que, em muitos locais, os pacientes retiram os medicamentos nas UBSs.^579^

**Table:** 

Mensagens principais sobre a atenção primária no SUS e a HA
As UBSs, estabelecimentos da APS, porta de entrada do SUS, realizam ações e atendimentos voltados à prevenção, promoção da saúde, diagnóstico e tratamento.
Mais do que prover assistência clínica, o objetivo é estar próximo às pessoas e promover a saúde e a qualidade de vida da comunidade.
A busca pelo controle da HA é um dos programas estratégicos da APS, e o médico generalista/MFC, juntamente com a equipe de saúde desse nível de complexidade, são os responsáveis pelo paciente.
Atualmente, existem 48.161 UBSs no Brasil, portanto é impossível traçar estratégias para aumentar a prevenção, o diagnóstico, o tratamento e o controle da HA no Brasil sem contar com essa rede.
A APS é o local adequado para que a prevenção, o diagnóstico, o tratamento e o controle da HA sejam otimizados.

APS: atenção primária à saúde; HA: hipertensão arterial; MFC: médico de família e comunidade; SUS: Sistema Único de Saúde; UBS; Unidade Básica de Saúde.

### 14.2. Busca Ativa por Pessoas com Hipertensão Arterial

Levando-se em conta que, na maioria dos casos, a HA é assintomática, a busca ativa por pessoas com a doença é amplamente justificada, pois o diagnóstico precoce e o tratamento efetivo reduzem os riscos de complicações. Assim, recomenda-se que todo indivíduo adulto (≥ 18 anos), sempre que acessar o SUS, deverá ter sua PA aferida por profissional capacitado, preferencialmente com aparelho automático oscilométrico de braço validado.^46^ Os valores da PA devem ser informados à pessoa, de preferência por escrito e de forma correta (por exemplo, 134/76 mmHg e não apenas "13 por 7").

Idealmente, todas as pessoas devem ter sua PA aferida pelo menos anualmente, em especial aquelas com história familiar de HA, idosas e com outra doença, entre elas, DM, obesidade, DCV ou renal. O profissional de saúde que fizer a medida da PA deve estar orientado a realizar pelo menos três medidas da PA e, caso a média das duas últimas medidas seja ≥ 140/90 mmHg, deverá vincular a pessoa à Unidade de Saúde de sua referência para uma consulta agendada.^46^ A confirmação dos valores da PA deve ser feita já na consulta de acolhimento e na consulta médica e em avaliações subsequentes, sempre de acordo com o protocolo de medida da PA descrito abaixo. A frequência das consultas subsequentes deverá ser avaliada individualmente, levando-se em conta o atingimento de metas e o controle de comorbidades.

### 14.3. Protocolo para a Aferição da Pressão Arterial: Cuidados Necessários

A medida correta da PA resulta em maior precisão no diagnóstico e no cuidado do indivíduo com HA, devendo respeitar rigorosamente os passos recomendados a seguir, em relação à pessoa que terá sua PA aferida, ao observador ou profissional de saúde e ao equipamento utilizado.

Os aparelhos automáticos oscilométricos validados de braço, desde que certificados pelo Instituto Nacional de Metrologia, Qualidade e Tecnologia (Inmetro) e instituições reconhecidas, são preferíveis, pois oferecem comprovadamente medidas precisas e confiáveis da PA, são mais fáceis de manipular, dispensam o uso do estetoscópio, necessitam de treinamento menos sofisticado e têm custo comparáveis aos aparelhos auscultatórios, que exigem mais cuidados para a utilização, particularmente a calibração a cada 6 meses, pouco disponível.^46^

Os cuidados para a correta aferição da PA estão resumidos na Figura 14.1. Siga esses passos e seu diagnóstico e condutas serão mais precisos e apropriados.^46^

**Figura 14.1:**
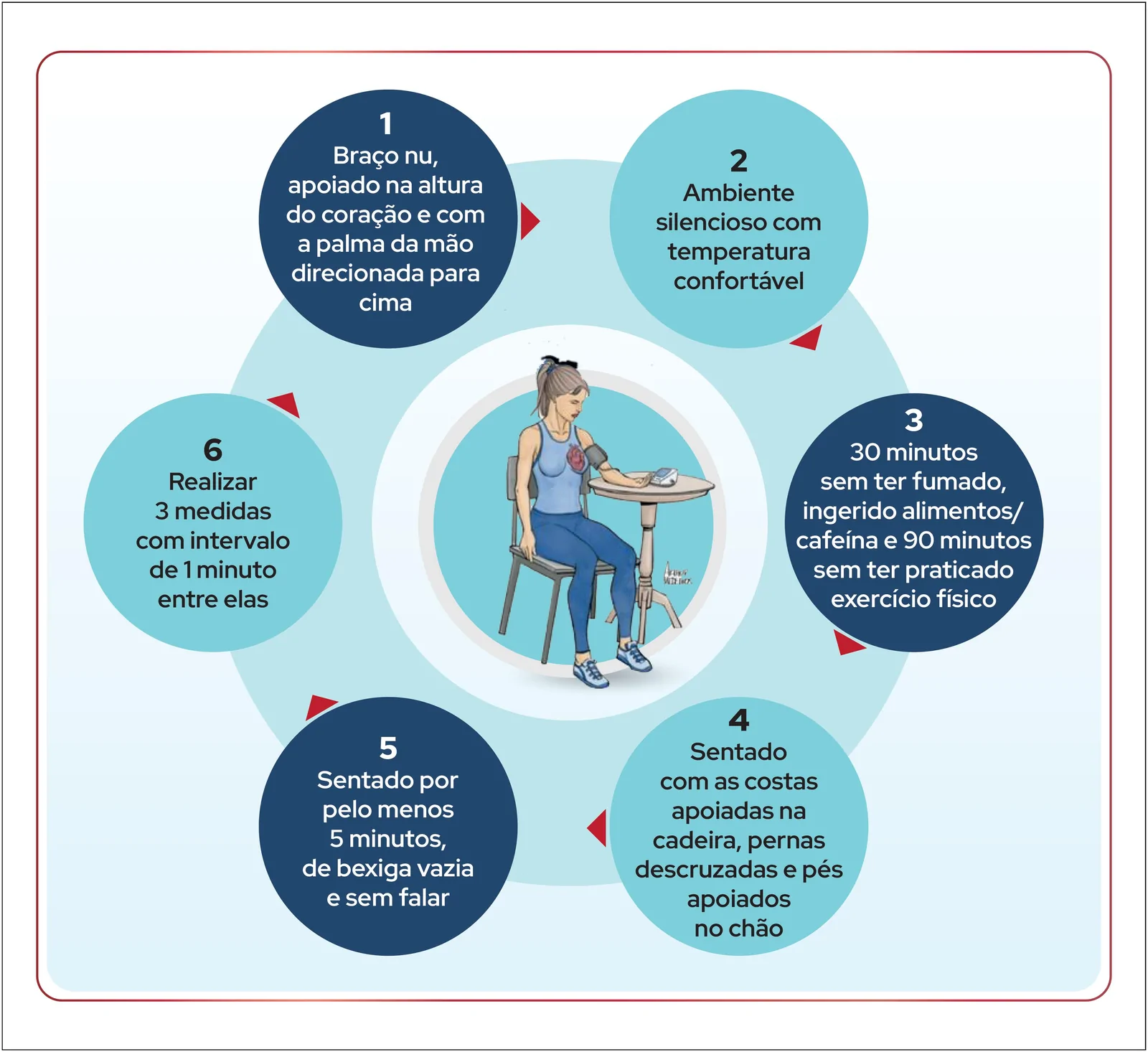
Cuidados indispensáveis para a aferição correta da pressão arterial.

### 14.4. Diagnóstico de Hipertensão Arterial

Considera-se que a pessoa tenha HA se as medidas da PA em consultório estiverem ≥ 140 mmHg (PAS) e/ou ≥ 90 mmHg (PAD), em pelo menos dois momentos diferentes, separados por intervalos de dias ou semanas. Caso a pessoa já use medicamentos para controlar a PA ou tenha LOA, ela deve ser considerada com HA, mesmo com valores pressóricos < 140/90 mmHg. Considera-se como tendo pré-hipertensão as pessoas com PAS entre 120 e 139 mmHg e/ou PAD entre 80 e 89 mmHg. Por outro lado, a PA é normal quando < 120/80 mmHg.

A HA também é classificada em estágios (1 a 3), como apresentado no Quadro 14.1. Como veremos adiante, as pessoas com pré-hipertensão devem receber as orientações necessárias para a prevenção primária da HA (Capítulos 3 e 6).

**Quadro 14.1:** Classificação da pressão arterial de acordo com a medida no consultório a partir de 18 anos de idade

Classificação da PA	PAS (mmHg)		PAD (mmHg)
PA normal	< 120	e	< 80
Pré-hipertensão	120-139	e/ou	80-89
HA Estágio 1	140-159	e/ou	90-99
HA Estágio 2	160-179	e/ou	100-109
HA Estágio 3	≥ 180	e/ou	110

DBHA: Diretriz Brasileira de Hipertensão Arterial; HA: hipertensão arterial; PA: pressão arterial; PAD: pressão arterial diastólica; PAS: pressão arterial sistólica. ^*^A classificação é definida de acordo com a PA no consultório e pelo nível mais elevado de PA, sistólica ou diastólica. ^**^A HA sistólica isolada, caracterizada pela PAS ≥ 140 mmHg e PAD < 90 mmHg, é classificada em Estágio 1, 2 ou 3, de acordo com os valores da PAS nos intervalos indicados. ^***^A HA diastólica isolada, caracterizada pela PAS < 140 mmHg e PAD ≥ 90 mmHg, é classificada em Estágio 1, 2 ou 3, de acordo com os valores da PAD nos intervalos indicados.

**Table:** 

Recomendações para a medida da pressão arterial	Forca da recomendação	Certeza da evidência
Recomenda-se classificar a pré-hipertensão abrangendo valores de PAS entre 120-139 mmHg ou PAD entre 80-89 mmHg no consultório com o objetivo de identificar precocemente indivíduos em risco e incentivar intervenções mais proativas e não medicamentosas para prevenir a progressão para HA.	FORTE	MODERADA
Recomenda-se que o diagnóstico de HA seja feito quando a PA no consultório ≥ 140 e/ou 90 mmHg em duas ocasiões diferentes e classificada em Estágio 1, 2 e 3, de acordo com o maior valor de PAS ou PAD.	FORTE	MODERADA
Recomenda-se realizar a medida da PA utilizando técnica e equipamento adequados.	FORTE	MODERADA
Recomenda-se utilizar equipamento automático de braço com o objetivo de reduzir erros e facilitar a obtenção da medida da PA.	FORTE	MODERADA
Recomenda-se a investigação de hipotensão ortostática em grupos de risco (pacientes idosos, com diabetes, disautonomia e em uso de anti-hipertensivos).	FORTE	ALTA
Recomenda-se usar MAPA ou MRPA para confirmar o diagnóstico de HA e monitorar tratamento.	FORTE	ALTA

HA: hipertensão arterial; MAPA: medida ambulatorial da pressão arterial; MRPA: medida residencial da pressão arterial; PA: pressão arterial; PAD: pressão arterial diastólica; PAS: pressão arterial sistólica.

### 14.5. Avaliação Clínica e Complementar do Indivíduo com Hipertensão Arterial

Uma vez estabelecido o diagnóstico, o indivíduo com HA deve ser avaliado do ponto de vista clínico, laboratorial e por imagem.^1,45^ Essa avaliação tem por finalidade estabelecer: 1) o possível comprometimento sistêmico da doença; 2) os fatores de risco cardiovascular (FRCVs) associados à HA; e 3) uma triagem baseada em dados clínicos e laboratoriais capaz de identificar as principais causas secundárias de HA. O Quadro 14.2 indica os pontos mais importantes da avaliação clínica.

**Quadro 14.2:** Avaliação clínica inicial da pessoa com hipertensão arterial1,^45^

**Anamnese**
• Tempo de evolução, sintomas (raro), presença de fatores de risco para HA primária (idade, sexo biológico, raça/cor da pele, hereditariedade/predisposição familiar; excesso de peso, consumo excessivo de sal, sedentarismo, etilismo e tabagismo). • Ausência de fatores de risco (10%) em pessoas com HA grave ou resistente ao tratamento com três medicamentos em dose plena, sugere hipertensão secundária (doença renal primária, estenose de artéria(s) renal(is), hiperaldosteronismo primário, feocromocitoma, Cushing etc.). Ver Capítulo 4. • O interrogatório sobre os diferentes aparelhos poderá evidenciar o comprometimento de órgãos-alvo da HA (coração, rins, cérebro, retina e artérias periféricas). Investigar a presença de dispneia aos esforços, edema, nictúria, anemia (indicativos de insuficiência cardíaca e/ou injúria renal), de dor precordial aos esforços (isquemia miocárdica) e de claudicação intermitente (insuficiência arterial periférica).
**Exame físico**
• Dados antropométricos: peso, altura, IMC = peso/altura^2^, circunferência abdominal e circunferência braquial, medida criteriosa da PA na posição sentada, deitada e em pé. • Aparelho cardiovascular e respiratório: ictus cordis desviado para a esquerda, presença de 3ª bulha e/ou 4ª bulha e estertores pulmonares; edema de membros inferiores e hepatomegalia; arritmias (extrassistolia e FA), hiperfonese da 2ª bulha nos focos aórtico e pulmonar e sopros valvares que podem ser secundários ao aumento das câmaras cardíacas. • Palpação comparativa dos pulsos carotídeos, radiais, aorta abdominal, pediosos e tibiais posteriores poderá indicar doença arterial obstrutiva periférica. Sopros abdominais ou nos flancos podem ser indicativos de estenose da(s) artéria(s) renal(is).

HA: hipertensão arterial; IMC: índice de massa corporal; PA: pressão arterial; FA: fibrilação atrial.

Além da avaliação clínica, o paciente com HA necessita de avaliação complementar mínima que deve ser repetida anualmente, sem prejuízo de outras indicações (Quadro 14.3). Esses exames estão disponíveis em todas as unidades de saúde da APS e são fundamentais para a pesquisa das causas mais comuns de HA secundária, do comprometimento sistêmico pela HA (LOA) e dos FRCVs associados à HA^1,45^ (Capítulo 4).

**Quadro 14.3:** Avaliação complementar mínima para o paciente com hipertensão arterial

Exames complementares	Indicação
Análise de urina	Detectar doença renal primária (proteinúria > 1,0 g/g creatinina; hematúria glomerular com dismorfismo eritrocitário ou cilindros hemáticos); comprometimento renal (proteinúria ou albuminúria).
Relação albuminúria/creatininúria em amostra isolada de urina	Avaliar o comprometimento renal e sistêmico precoce. Anormal se superior a 30 mg/g de creatinina.
Potássio plasmático	Diminuído: auxiliar no diagnóstico de hiperaldosteronismo primário ou secundário e/ou uso de diurético Aumentado: injúria renal e/ou uso de IECA, BRA, ARM.
Creatinina plasmática	Avaliar a função renal: estimar a taxa de filtração glomerular pela fórmula CKD-EPI; adequar o uso dos diuréticos; acessar o risco CV.
Glicemia em jejum e HbA1c	Diagnosticar diabetes mellitus e acessar o controle glicêmico; acessar o risco CV.
Colesterol total, HDL-colesterol e triglicerídeos plasmáticos*	Diagnosticar e controlar as dislipidemias; acessar o risco CV.
Ácido úrico	Acessar o risco CV, particularmente em homens.
Eletrocardiograma convencional**	Avaliar o comprometimento cardíaco (hipertrofia, infarto antigo) e arritmias cardíacas; acessar o risco CV.

ARM: antagonistas dos receptores mineralocorticoides; BRA: bloqueadores dos receptores da angiotensina II; CKD-EPI: Chronic Kidney Disease-Epidemiology Collaboration (fórmula que estima a taxa de filtração glomerular); CV: cardiovascular; IECA: inibidores da enzima conversora da angiotensina; HbA1c: hemoglobina glicada; HDL: lipoproteínas de alta densidade (high-density lipoprotein).

* O LDL-colesterol (lipoproteínas de baixa densidade – low-density lipoprotein) é calculado pela fórmula: LDLc = colesterol total – (HDLc + triglicerídeos/5) (quando a dosagem de triglicerídeos for abaixo de 400 mg/dL).

** Critério de detecção de hipertrofia ventricular esquerda (HVE) – Sokolow-Lyon: SV1 + RV5,6 > 35 mm; Cornell Voltagem: RaVL + SV3 > 20 mm (mulher), > 28 mm (homem).

### 14.6. Classificação de Risco após a Avaliação Clínica e Complementar

A avaliação clínica e complementar permite combinar as informações e classificar o risco das pessoas com HA em: "sem risco adicional"; "risco baixo"; "risco moderado" e "risco alto" ou "muito alto". Quando disponível, recomenda-se avaliar o risco CV escore de risco PREVENT da AHA,^580^ que pode ser acessada pelo link a seguir: https://professional.heart.org/en/guidelines-and-statements/prevent-calculator. Caso não seja possível fazer essa avaliação, pode-se estratificar o risco CV conforme o Quadro 14.4. Essa classificação de risco é a base para definir as necessidades individuais, o fluxo de atendimento, as prioridades e o rol de cuidados a serem dispensados às pessoas com HA na APS.

**Quadro 14.4:** Classificação dos estágios de HA de acordo com o nível de PA, presença de FRCV, LOA ou DCV estabelecida

FRCV, presença de LOA ou DCV	PA (mmHg)			
	Pré-hipertensão, com PAS 130-139 PAD 80-89	Estágio 1 PAS 140-159 PAD 90-99	Estágio 2 PAS 160-179 PAD 100-109	Estágio 3 PAS ≥ 180 PAD ≥ 110
Sem FRCV	Risco baixo	Risco baixo	Risco moderado	Risco alto
1 ou 2 FRCV	Risco baixo	Risco moderado	Risco alto	Risco alto
≥ 3 FRCV	Risco moderado	Risco alto	Risco alto	Risco alto
LOA, DRC estágio 3 ou DM	Risco alto	Risco alto	Risco alto	Risco muito alto
DCV estabelecida ou DRC estágio ≥ 4	Risco muito alto	Risco muito alto	Risco muito alto	Risco muito alto

FRCV: fator de risco cardiovascular; DCV: doença cardiovascular (infarto do miocárdio, insuficiência cardíaca, acidente vascular cerebral, doença vascular de extremidades); DM: diabetes mellitus; DRC: doença renal crônica; LOA: lesão em órgão-alvo, PA: pressão arterial; PAD: pressão arterial diastólica; PAS: pressão arterial sistólica; São considerados FRCV para essa análise: sexo masculino; idade: > 55 anos no homem e > 65 anos na mulher; DCV prematura em parentes de 1^o^ grau (homens < 55 anos e mulheres < 65 anos); tabagismo; dislipidemia: LDL-colesterol (lipoproteínas de baixa densidade – low-density lipoprotein) ≥ 100 mg/dL e/ou não HDL-colesterol (lipoproteínas de alta densidade – high-density lipoprotein) ≥ 130 mg/dL e/ou HDL-colesterol ≤ 40 mg/dL no homem e ≤ 50 mg/dL na mulher e/ou TG (triglicerídeos) ≥ 150 mg/dL; obesidade (IMC [índice de massa corporal] ≥ 30 kg/m^2^).

**Table:** 

Recomendações para avaliação clínica e complementar, e estratificação de risco no indivíduo com pré-hipertensão e com hipertensão arterial	Forca da recomendação	Certeza da evidência
Recomenda-se realizar anamnese e exame físico com o objetivo de se obter um diagnóstico mais preciso e ajudar na identificação de causas secundárias de HA e LOA.	FORTE	BAIXA
Recomenda-se pesquisar FRCV e LOA idealmente em todos os pacientes no momento do diagnóstico de HA e repetir essa avaliação pelo menos anualmente, sendo a escolha do método decidida conforme recursos disponíveis.	FORTE	BAIXA
Recomenda-se verificar a presença de doença renal e classificá-la de acordo com o KDIGO 2024.	FORTE	MODERADA
Recomenda-se avaliar o risco CV utilizando o escore PREVENT.	FORTE	ALTA
Recomenda-se estratificar o risco CV em indivíduos com pré-hipertensão com o objetivo de orientar o início do tratamento anti-hipertensivo e promover controle mais adequado dos FRCVs.	FORTE	ALTA
Recomenda-se estratificar o risco CV em pacientes com HA com o objetivo de promover uma abordagem mais precisa e personalizada no manejo de medicamentos e direcionamento de metas para controle dos FRCVs.	FORTE	ALTA

CV: cardiovascular; FRCV: fator de risco cardiovascular; HA: hipertensão arterial; KDIGO: Kidney Disease Improving Global Outcomes; LOA: lesão de órgãos-alvo; PREVENT: Predicting Risk of Cardiovascular Disease Events.

### 14.7. Metas do Controle Pressórico

Os estudos mais recentes têm mostrado redução importante do risco CV e renal quando se controla a PA em valores mais baixos. Assim, de uma forma geral e independente do risco CV, recomenda-se para as pessoas com HA que a PA seja mantida em valores < 130/80 mmHg.^366,581^ Algumas pessoas podem não tolerar tais valores de PA. Nesses casos, deve-se titular para os níveis mais baixos toleráveis.

**Table:** 

Recomendações para as metas da pressão arterial	Força da recomendação	Certeza da evidência
Recomenda-se a meta de PA < 130/80 mmHg para pacientes com PA 130-139/80-89 mmHg e risco CV alto.	FORTE	ALTA
Recomenda-se a meta de PA < 130/80 mmHg para os pacientes com HA, independentemente de o risco CV ser baixo, moderado ou alto.	FORTE	ALTA
Recomenda-se que, para pacientes que não tolerem a meta de PA < 130/80 mmHg, deve-se reduzir a PA até o valor mais baixo tolerado.	FORTE	MODERADA
Recomenda-se confirmar o alcance da meta de PA com a medida da PA fora do consultório por meio da MAPA ou MRPA.	FORTE	BAIXA

CV: cardiovascular; HA: hipertensão arterial; MAPA: monitorização ambulatorial da pressão arterial; MRPA: monitorização residencial da pressão arterial; PA: pressão arterial.

### 14.8. Medida da Pressão Arterial fora do Consultório ou do Ambiente de Cuidado à Saúde

Pode ser feita pela MAPA e pela MRPA. A MAPA é realizada com aparelhos automáticos que realizam medidas da PA a intervalos de 15 a 20 minutos durante a vigília e de 20 a 30 minutos durante o sono, e a MRPA com aparelhos automáticos oscilométricos validados, calibrados e que permitam idealmente recuperar os valores registrados na memória do aparelho.^46^ A disponibilidade de aparelhos automáticos oscilométricos a um custo aceitável tem permitido cada vez mais a realização de medidas da PA fora do ambiente de cuidado à saúde, que podem ser muito úteis na definição de diagnósticos e condutas terapêuticas para as pessoas com suspeita ou em tratamento da HA.

O Quadro 14.5 apresenta os valores que são considerados normais e as indicações para esses dois métodos de medida da PA fora do consultório.^46^ Ambas estão aprovadas no âmbito do SUS exclusivamente para fins diagnósticos.^582^ Esta diretriz propõe que os gestores locais de saúde garantam o acesso à MRPA para pessoas com as outras indicações clássicas: a avaliação da eficácia terapêutica anti-hipertensiva; o diagnóstico de HAR (PA fora da meta, apesar da otimização do tratamento anti-hipertensivo com três medicamentos sinérgicos, nas doses máximas toleradas) e a PA controlada com indícios de progressão de LOA, conforme descrito no Capítulo 12 e na Portaria SETICS/MS nº 22 de 10 de maio de 2023.^582^

**Quadro 14.5:** Valores normais da pressão arterial e indicações para a MAPA e MRPA^46^

Valores normais:	MAPA Vigília < 135/85 mmHg Sono < 120/70 mmHg 24 horas < 130/80 mmHg	MRPA 4 a 6 dias em 1 semana Três medidas matinais e três medidas noturnas/dia Média < 130/80 mmHg
Indicações MAPA e MRPA		
Diagnóstico de hipertensão do avental branco	+ + +	+ +
Diagnóstico de hipertensão mascarada	+ + +	+ +
Acompanhamento do tratamento com anti-hipertensivos	+ +	+ + +
Confirmação de hipertensão resistente	+ + +	+ +
Ausência de descenso do sono	+ + +	Não se aplica

MAPA: monitorização ambulatorial da pressão arterial; MRPA: monitorização residencial da pressão arterial. + quanto maior o número de +, mais forte a indicação para o uso da MAPA ou MRPA

**Table:** 

Recomendação para uso da MAPA ou MRPA^66,6^^7^	Força da recomendação	Certeza da evidência
Recomenda-se usar MAPA ou MRPA para confirmar o diagnóstico de HA e monitorar o tratamento.	FORTE	ALTA

HA: hipertensão arterial; MAPA: monitorização ambulatorial da pressão arterial; MRPA: monitorização residencial da pressão arterial.

### 14.9. Bases do Tratamento da Pessoa com Hipertensão Arterial

A HA é uma doença muito prevalente, multifatorial, complexa e, embora assintomática, traz sérios agravos à saúde. Para se obter o controle adequado da doença, é necessário que o paciente e a família compreendam a importância de se manter a PA dentro das metas preconizadas, os princípios do tratamento, a necessidade de MNM, o uso correto e contínuo dos medicamentos prescritos e o acompanhamento regular pela equipe de saúde. Essas metas só serão atendidas se os cuidados da equipe multiprofissional forem complementados pelo autocuidado e adesão do paciente, o que também envolve o apoio familiar.^1^

A equipe multiprofissional facilita e torna mais efetiva essa tarefa, tanto no controle da PA como na redução da morbidade e mortalidade associadas à HA, metas primordiais do tratamento. A abordagem multiprofissional melhora a qualidade da assistência, promove maior adesão ao tratamento medicamentoso e não medicamentoso, coloca o paciente no centro das tomadas de decisão, humaniza a assistência, aumentando para 68% a taxa de pessoas com a PA controlada.^573,573,584^ O modelo de assistência multiprofissional exige integração, agilidade e comunicabilidade de toda a equipe. Cada profissional de saúde deve reconhecer seu papel específico e complementar essa cadeia em busca do melhor cuidado à saúde possível. A equipe multiprofissional poderá ser composta por: a) médico generalista/MFC; b) enfermeiro, c) agente comunitário de saúde; d) nutricionista; e) educador físico e fisioterapeuta; f) farmacêutico; g) psicólogo; h) assistente social; e i) familiares (Figura 14.2).^585^

**Figura 14.2:**
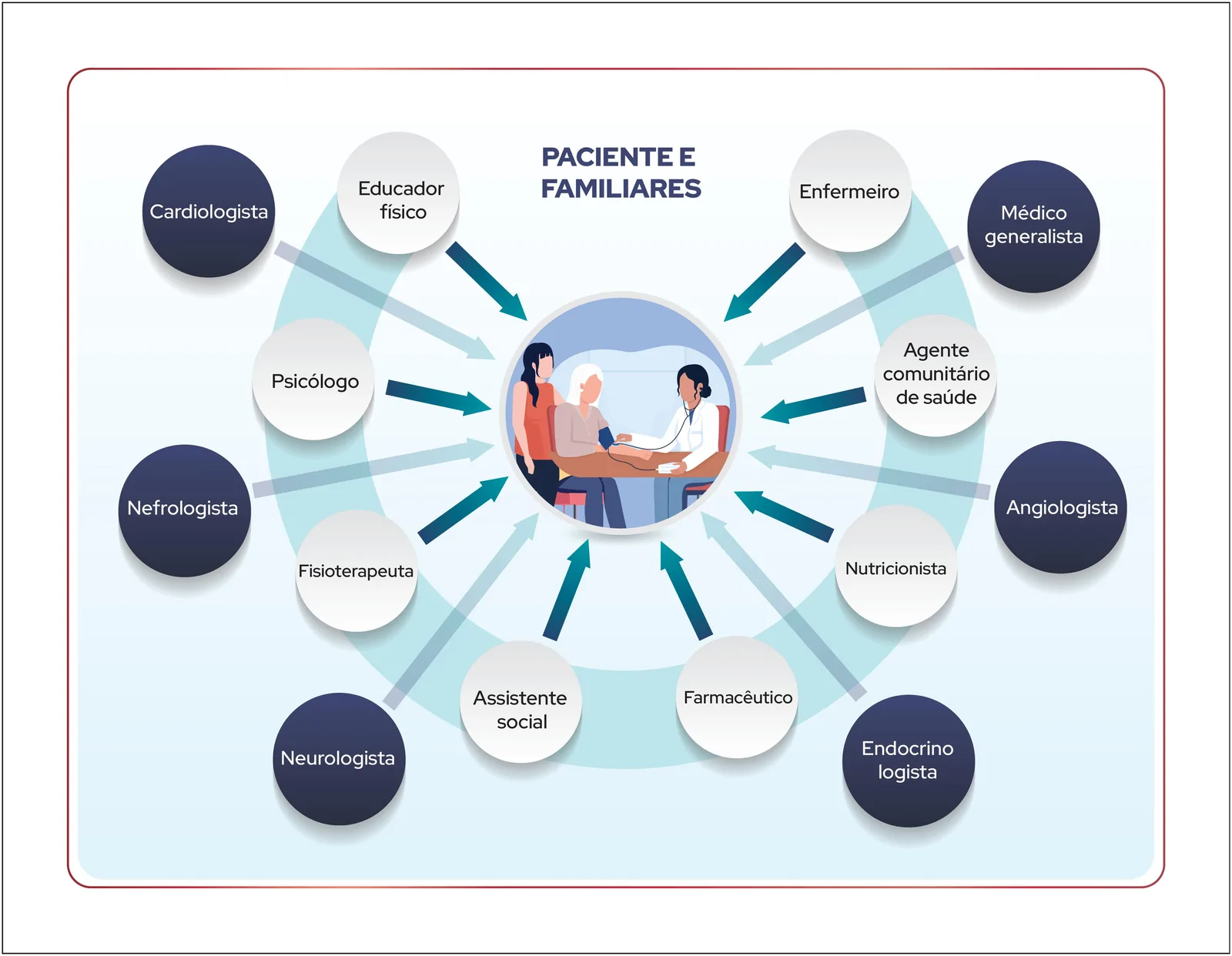
Modelo de atendimento e composição da equipe multiprofissional

Cada município e cada unidade de saúde deve se adequar às necessidades e possibilidades locais para formar a equipe de saúde com a maior diversidade possível de profissionais. As evidências sugerem que uma abordagem multidisciplinar traz benefícios adicionais à efetividade do tratamento e, consequentemente, melhores resultados.^194^ Nesse sentido, as ações educativas respeitando a autonomia e as necessidades individuais dos pacientes e da comunidade são fundamentais para melhorar a adesão ao tratamento medicamentoso e não medicamentoso.^586^

O tratamento não medicamentoso é um dos pilares para o controle da HA, e as principais intervenções, seus efeitos e recomendações são apresentados no Quadro 14.6.

**Quadro 14.6:** Efeito das medidas não medicamentosas para o controle da pressão arterial (Capítulo 6)

Medida não medicamentosa	Redução aproximada da PAS/PAD	Recomendação
Cessação do tabagismo	Resultados são controversos.	Recomendada universalmente. Não usar derivados do tabaco, narguilê e cigarros eletrônicos. Reduzir o tabaco não é suficiente.
Redução do peso	Em média, a redução de 1 kg corporal reduz a PAS em 1,05 mmHg e a PAD em 0,92 mmHg.	Manter IMC < 25 kg/m^2^ até 65 anos; Manter IMC entre 22 e 27 kg/m^2^, após os 65 anos.
Padrão alimentar	Dieta DASH reduz a PAS em 8,7 mmHg e a PAD em 4,5 mmHg.	Dieta rica em verduras e frutas, cereais integrais, laticínios com baixo teor de gordura e carnes brancas (dieta DASH).
Aumento da ingestão de potássio	Reduz a PAS em 4,8 mmHg e a PAD em 3,0 mmHg.	Dieta rica em frutas e verduras (ideal ≥ 3,5 g/dia).
Redução de sal na dieta	A redução de cada 1,15 g de sódio/dia diminui a PAS em 2,8 mmHg e a PAD em 1,4 mmHg.	Restringir a 2 g de sódio/dia (5 g de sal de cozinha/dia). Alimentos naturais já contêm 2 g de sal/dia).
Redução do consumo de álcool	Redução de 50% para os que ingerem seis doses/dia promove diminuição da PAS em 5,5 mmHg e da PAD em 4,0 mmHg.	Limitar o consumo de álcool a uma dose para mulheres e duas doses para homens.
Exercícios físicos	Aeróbicos – redução de 7,6/4,7 mmHg; Combinado (resistido e dinâmico) – redução de 5,3/5,6 mmHg.	Treinamento aeróbico: três a cinco vezes por semana; 30 a 60 minutos por sessão ou 150 minutos por semana.

FC: frequência cardíaca; DASH: Diet Approach to Stop Hypertension; IMC: índice de massa corporal; PAD: pressão arterial diastólica; PAS: pressão arterial sistólica. 1 dose = 10 a 12 g de álcool puro (200 mL de cerveja, 100 mL de vinho, 25 mL de destilados).

Outras intervenções recomendadas no controle da PA, mas com graus de evidência menos robustas são: prática de meditação, respiração lenta e estímulo à espiritualidade ou religiosidade.^1^

**Table:** 

Recomendações para as medidas não medicamentosas para redução da pressão arterial	Força da recomendação	Certeza da evidência
Recomenda-se a cessação do tabagismo para a redução de eventos cardiovasculares e morte.	FORTE	ALTA
Recomenda-se a diminuição do peso corporal para a redução da PA e de mortalidade em pacientes com obesidade.	FORTE	ALTA
Recomenda-se a redução da ingestão de sódio e aumento da ingestão de potássio (exceto para pacientes com DRC) dietético para a redução da PA.	FORTE	ALTA
Recomenda-se limitação do consumo máximo diário de álcool para a redução da PA.	FORTE	MODERADA
Recomendam-se a dieta DASH e a prática regular de AF moderada para a redução da PA e mortalidade.	FORTE	ALTA
Recomenda-se treinamento aeróbico para a redução da PA.	FORTE	ALTA
Recomenda-se meditação para a redução da PA.	FORTE	MODERADA
Recomendação a favor da prática de respiração lenta para a redução de PA.	NEUTRA	BAIXA
Recomenda-se a abordagem de aspectos de espiritualidade e religiosidade no tratamento da HA.	NEUTRA	BAIXA
Recomenda-se a atuação de equipe multiprofissional para se atingir um melhor controle da PA.	FORTE	BAIXA

DASH: Dietary Approaches to Stop Hypertension; DRC: doença renal crônica; HA: hipertensão arterial; PA: pressão arterial; AF: atividade física.

O início do tratamento não medicamentoso e medicamentoso deve ser feito como apontado no Quadro 14.7.

**Quadro 14.7:** Indicações para início do tratamento não medicamentoso e medicamentoso de acordo com o valor da pressão arterial, idade e risco cardiovascular

Tratamento	População-alvo	Início do tratamento
Medidas não medicamentosas	PA ≥ 120/80 mmHg	No diagnóstico
Tratamento medicamentoso	PA ≥ 140/90 mmHg	No diagnóstico
	PA 130-139/80-89 mmHg e alto risco CV	Quando não há controle da PA após 3 meses de MNM

CV: cardiovascular; MNM: medidas não medicamentosas; PA: pressão arterial.

**Table:** 

Recomendações para o início do tratamento com medidas não medicamentosas e medicamentosas	Força da recomendação	Certeza da evidência
Recomendam-se MNM para todos com PA ≥ 120/80 mmHg.	FORTE	ALTA
Recomenda-se tratamento medicamentoso após 3 meses de MNM para indivíduos com PA 130-139/80-89 mmHg e alto risco CV.	FORTE	ALTA
Recomenda-se o início de tratamento medicamentoso para indivíduos com PA ≥ 140/90 mmHg.	FORTE	ALTA

CV: cardiovascular; MNM: medidas não medicamentosas; PA: pressão arterial.

### 14.10. Tratamento com Medicamentos

Quanto ao tratamento medicamentoso, todas as classes de medicamentos anti-hipertensivos estão disponíveis no SUS e podem ser utilizadas a depender das especificidades clínicas de cada pessoa com HA^1^ (Quadro 14.8).

**Quadro 14.8:** Classes e medicações disponíveis no Sistema Único de Saúde, no componente básico e no Programa Farmácia Popular, suas apresentações, doses diárias e frequência

CLASSES E MEDICAÇÕES	Apresentação (mg)	Componente básico	Programa Farmácia Popular	Doses diárias (mg)	Freq./dia	Observações importantes
**DIURÉTICOS VIA ORAL**						
Espironolactona	25	x	x	25-100	1-2	Pode provocar hiperpotassemia, particularmente na IRC e quando associado a IECA ou BRA.
Espironolactona	100	x			1-2	
Furosemida	40	x	x	20-240	1-3	Utilizado em pacientes com edema como ocorre na IRC e ICC.
Hidroclorotiazida	12,5	x		25-50	1	
Hidroclorotiazida	25	x	x	25-50	1	
**BLOQUEADORES DE CANAIS DE CÁLCIO**						
Cloridrato de verapamil	80	x		120-360	1-2	Evite o uso em pacientes com IC com fração de ejeção reduzida. Evite a associação com BBs e em pacientes com bradicardia.
Cloridrato de verapamil	120	x		120-360	1-2	
Anlodipina	5	x		2,5-10	1	Evite o uso em pacientes com IC com fração de ejeção reduzida. Pode provocar edema de membros inferiores relacionado à dose utilizada.
Anlodipina	10	x		2,5-10	1	
Nifedipina	10	x		10-60	1-3	
**INIBIDORES DA ENZIMA CONVERSORA DA ANGIOTENSINA**						
Captopril	25	x	x	25-150	2-3	Evite o uso em mulheres em idade fértil, pois há grande risco de malformações fetais e outras complicações na gestação. Contraindicado em associação com BRA. Risco de hiperpotassemia em pacientes com IC, em uso de ARM, ou que estejam recebendo suplementação de potássio.
Maleato de enalapril	5	x		5-40	1-2	
Maleato de enalapril	10	x	x	5-40	1-2	
Maleato de enalapril	20	x		5-40	1-2	
**BLOQUEADORES DOS RECEPTORES AT 1 DA ANGIOTENSINA II**						
Losartana potássica	50	x	x	50-100	1-2	Mesmas recomendações feitas aos IECAs.
**BETABLOQUEADORES**						
Atenolol	25		x			Deve ser indicado em pacientes com doença arterial coronariana (angina e pós-IM), IC, fibrilação atrial, mulheres que pretendem engravidar, pessoas com enxaqueca. Evitar em pacientes com doença pulmonar obstrutiva crônica, bloqueio AV > 2º grau e doença arterial obstrutiva periférica. A retirada abrupta dos BBs deve ser evitada, pois pode provocar taquicardia reflexa e mal-estar.
Atenolol	50	x		50-100	1-2	
Atenolol	100	x		50-100	1-2	
Cloridrato de propranolol	10	x		80-320	2-3	
Cloridrato de propranolol	40	x	x	80-320	2-3	
Succinato de metoprolol	25	x	x	50-200	1	
Succinato de metoprolol	50	x		50-200	1	
Succinato de metoprolol	100	x		50-200	1	
Tartarato de metoprolol	100	x		50-200	1	
**SIMPATOLÍTICO DE AÇÃO PERIFÉRICA**						
Mesilato de doxazosina	2	x		1-16	1	Iniciar com dose baixa antes de deitar-se, pois pode provocar hipotensão ortostática. Aumentar progressivamente a cada 2 dias.
Mesilato de doxazosina	4	x		1-16	1	
**SIMPATOLÍTICO DE AÇÃO CENTRAL**						
Metildopa	250	x		500-2.000	2	Indicado preferencialmente em grávidas.
**VASODILATADORES DIRETOS**						
Cloridrato de hidralazina	25	x		50-200	2-3	Pode provocar retenção de sódio e água, hipervolemia e taquicardia reflexa. Preferencialmente, deve ser usada em associação com diurético e BB. Síndrome *lupus-like* em dose alta.
Cloridrato de hidralazina	50	x		50-200	2-3	

ARM: antagonistas dos receptores mineralocorticoides; II; AV: atrioventricular; BB: betabloqueador; BCC: bloqueador de canais de cálcio; BRA: bloqueador dos receptores AT1 da angiotensina II; IC: insuficiência cardíaca; ICC: insuficiência cardíaca congestiva; IECA: inibidor da enzima conversora da angiotensina; IM: infarto do miocárdio; IR: injúria renal; IRC: injúria renal crônica.

O aplicativo MedSUS, com informações sobre todos os medicamentos disponíveis na assistência farmacêutica do SUS, já pode ser baixado pela população e profissionais de saúde via plataformas de distribuição digital para IOS ou Android. Uma das principais funcionalidades do aplicativo é fornecer informações sobre os medicamentos disponíveis, alinhados, quando necessário, aos Protocolos Clínicos e Diretrizes Terapêuticas (PCDT) e à Classificação Estatística Internacional de Doenças e Problemas Relacionados com a Saúde (CID). Além disso, o MedSUS fornece informações sobre os locais de dispensação dos medicamentos, como as farmácias das UBS (atenção primária), farmácias de "alto custo" (componente especializado da assistência farmacêutica) e as farmácias credenciadas junto ao Programa Farmácia Popular.^587^

A monoterapia só se justifica para os casos de HA Estágio 1 com risco baixo, os muito idosos (> 80 anos), as pessoas frágeis ou com hipotensão ortostática ou para a pré-hipertensão com risco alto ou muito alto (Quadro 14.4 e Figura 14.3 e Figura 7.3 – Capítulo 7).

**Figura 14.3:**
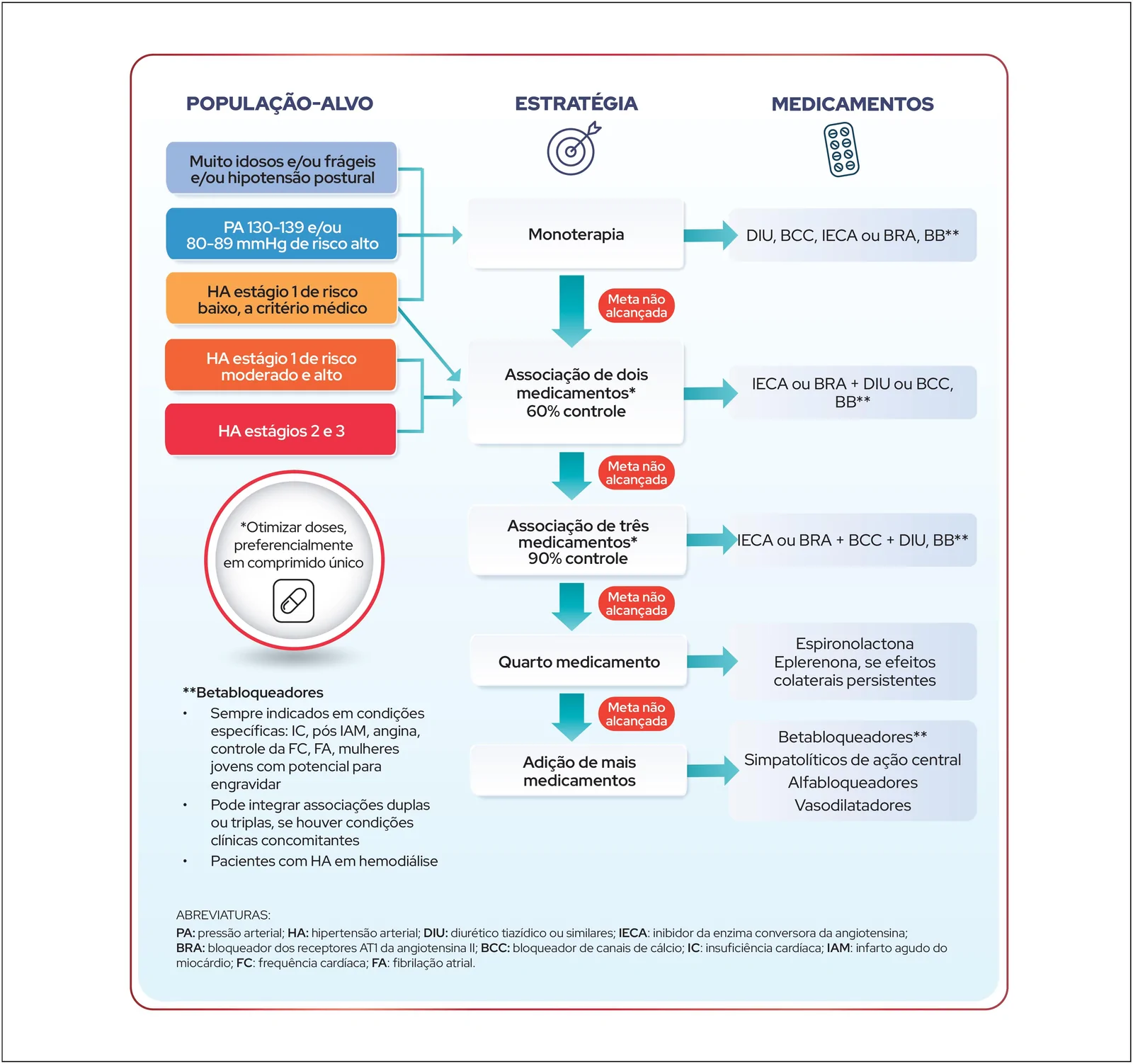
Fluxograma do tratamento medicamentoso da hipertensão arterial.

A combinação de medicamentos é mais efetiva, permite alcançar as metas de PA mais rapidamente, reduz a inércia terapêutica, é segura e recomendada pela maioria das diretrizes.^245,249-251,259^ Recomenda-se iniciar com a combinação de um IECA ou BRA e um DIU tiazídico ou um BCC. Um estudo brasileiro utilizou o esquema de tratamento escalonado combinado de DIU/IECA ou BRA, seguido de anlodipino e, ao final de 12 semanas, possibilitou o controle de 85% de hipertensos Estágio 2 ou 3 atendidos pelo SUS em hospitais universitários de referência.^259^ Deve-se alcançar as doses plenas da combinação de dois medicamentos antes de iniciar o terceiro (Capítulo 7). Os BB podem ser utilizados em casos específicos: pós-IAM; angina; IC; taquiarritmias; mulheres com potencial de engravidar e intolerância aos anti-hipertensivos anteriores. A escolha deve levar em consideração as comorbidades que o paciente apresente.

Caso a PA não seja controlada com a associação de três medicamentos em doses máximas toleradas, incluindo-se, entre eles, preferencialmente, um DIU, caracteriza-se como HAR. Nesse caso, recomenda-se que o quarto medicamento seja, preferencialmente, a espironolactona (25 a 50 mg/dia) e, em seguida, um simpatolítico (metildopa 500 a 1.500 mg/dia ou clonidina 0,200 a 0,600 mg/dia) ou um BB (carvedilol 6,25 a 50 mg/dia, metoprolol 50 a 200 mg/dia ou atenolol 50 a 100 mg/dia), caso não alcance o controle da PA ou se houver eventos adversos com espironolactona (ver Quadro 7.2 – Capítulo 7 e Capítulo 12) (Figura 14.3).

**Table:** 

Recomendação para o tratamento medicamentoso da hipertensão arterial no Sistema Único de Saúde	Força da recomendação	Certeza da evidência
Recomenda-se a associação de medicamentos, preferencialmente em comprimido único e com as classes preferenciais, para atingir metas rigorosas de PA (< 130/80 mmHg) e reduzir eventos CVs e renais.	FORTE	MODERADA
Recomenda-se monoterapia para indivíduos com PA 130-139/80-89 mmHg e alto risco CV; pacientes com HA Estágio 1 de baixo risco (a critério médico, pode-se optar por associação); em indivíduos frágeis; muito idosos (≥ 80 anos) ou com hipotensão ortostática sintomática, especialmente em indivíduos idosos.	FRACA	BAIXA
Recomenda-se, para a maioria dos pacientes, o início do tratamento da HA com associação dupla de medicamentos, em doses baixas, preferencialmente em um comprimido único, se disponível.	FORTE	MODERADA
Recomenda-se o uso de diuréticos tiazídicos, IECA ou BRA e BCC como classes preferenciais para o tratamento da HA e redução de eventos CV e renais importantes.	FORTE	ALTA
Recomenda-se o uso de BB para o tratamento de HA em situações específicas: IC, FA, arritmias, DAC, HA em pacientes em HD e outras (enxaqueca, tremor essencial, mulheres que desejam engravidar, varizes esofágicas).	FORTE	MODERADA
Recomenda-se o uso de espironolactona (ou eplerenona, caso ocorra intolerância à espironolactona) para atingir metas rigorosas de PA (< 130/80 mmHg) e reduzir eventos CVs e renais quando não se tenha atingido apenas com as classes iniciais (HAR e HARf).	FORTE	ALTA

BB: betabloqueador; BCC: bloqueador dos canais de cálcio; BRA: bloqueador dos receptores da angiotensina; CV: cardiovascular; DAC: doença arterial coronariana; FA: fibrilação atrial; HA: hipertensão arterial; HAR: hipertensão arterial resistente; HARf: hipertensão arterial refratária; HD: hemodiálise; IC: insuficiência cardíaca; IECA: inibidor da enzima conversora da angiotensina; PA: pressão arterial.

Recentemente a AHA estabeleceu um conceito de "saúde cardiovascular global" que se aproxima do cuidado integral à saúde, uma das bases do SUS. São oito itens clínicos facilmente verificáveis (dieta saudável, AF, livre de tabagismo, sono saudável, peso adequado, níveis normais de PA, glicemia e lipídeos nas metas de proteção CV e renal) associados ao cuidado com a saúde mental e os determinantes sociais de saúde.^588^ Portanto, são esses 10 itens que devem ser considerados pela equipe de saúde, particularmente nas pessoas com HA.

**Table:** 

Mensagens principais diagnóstico, avaliação clínica e complementar, meta de pressão arterial e tratamento da hipertensão arterial
Todo indivíduo adulto (≥ 18 anos), sempre que acessar o SUS, deverá ter sua PA aferida por profissional capacitado, preferencialmente com aparelho automático oscilométrico de braço validado.
O diagnóstico de HA é definido quando as medidas da PA em consultório estiverem ≥ 140 mmHg (PAS) e/ou ≥ 90 mmHg (PAD), em pelo menos dois momentos diferentes.
Sempre que possível, a medida da PA fora do consultório (MAPA ou MRPA) deve ser utilizada para o diagnóstico e acompanhamento do tratamento.
Todo paciente com HA deve ter uma avaliação clínica e complementar mínima, que deve ser repetida anualmente. Essas informações permitem estimar o risco CV do indivíduo.
A meta de PA recomendada é < 130/80 mmHg.
O tratamento não medicamentoso deve ser enfatizado e inclui: cessação do tabagismo, redução do peso, padrão alimentar saudável, aumento da ingestão de potássio, redução da ingestão de sal e de álcool, e exercícios físicos regulares.
O tratamento medicamentoso deve ser feito com associação de medicamentos para a grande maioria dos pacientes. A monoterapia está indicada apenas para pacientes ≥ 80 anos, frágeis, com HA Estágio 1 de risco baixo, ou com pré-hipertensão de risco alto ou muito alto.
As principais classes de anti-hipertensivos são: diuréticos tiazídicos, bloqueadores dos canais de cálcio, inibidores da ECA e bloqueadores dos receptores da angiotensina II.
A abordagem multiprofissional melhora a qualidade da assistência, promove maior adesão ao tratamento medicamentoso e não medicamentoso, coloca o paciente no centro das tomadas de decisão e humaniza a assistência.

ECA: enzima conversora da angiotensina; HA: hipertensão arterial; MAPA: medida ambulatorial da pressão arterial; MRPA: medida residencial da pressão arterial; PA: pressão arterial.

### 14.11. Condições Clínicas que Justificam o Encaminhamento a Especialista(s)

Para garantir a sustentabilidade do sistema de saúde e o aumento da sua eficiência, é importante que tais encaminhamentos obedeçam a critérios objetivos e visem complementar e melhorar o cuidado prestado na APS. Esse encaminhamento não necessariamente tem caráter definitivo e, na maioria das vezes, deve servir apenas como interconsulta eventual, ou acompanhamento conjunto, mas a contrarreferência deve ser a regra. O prontuário eletrônico único facilita essa interação e deve ser meta de todos os municípios.

O Quadro 14.9 apresenta as condições mais comuns para encaminhamento a especialistas. Alternativas cada vez mais utilizadas em muitos municípios com bons resultados são o matriciamento com especialistas ou a teleconsulta generalista/MFC-especialista, que também têm a função de capacitar os profissionais da APS, melhorando o grau de resolutividade dessa instância de atendimento.

**Quadro 14.9:** Condições clínicas que justificam o encaminhamento à especialista(s) – referência

Condições que justificam encaminhamento a especialista(s)	Especialista
Suspeita de HA secundária para investigação diagnóstica	Nefrologista Endocrinologista Cardiologista
Presença de hipertrofia ventricular esquerda, insuficiência cardíaca, FA, angina, doença coronariana preexistente	Cardiologista
Nefropatia (taxa de filtração glomerular estimada < 30 mL/min/1,73 m^2^ e/ou proteinúria ≥ 300 mg/g creatinina)	Nefrologista
Claudicação intermitente, sopro abdominal ou carotídeo, massa pulsátil sugestiva de aneurisma de aorta	Cirurgião vascular
Hipertensão resistente ao tratamento com três medicamentos em doses máximas toleradas	Cardiologista Nefrologista Endocrinologistas, se houver suspeita de causas endócrinas
Diabetes mellitus com retinopatia, neuropatia, HbA1c > 10%	Endocrinologista Oftalmologista
Avaliação de indicação de cirurgia bariátrica (IMC ≥ 35 kg/m^2^ com comorbidades)	Endocrinologista
Acidente vascular cerebral ou quadro demencial, sem investigação prévia	Neurologista Geriatra, se idoso

HA: hipertensão arterial; HbA1c: hemoglobina glicada; IMC: índice de massa corporal; FA: fibrilação atrial.

### 14.12. Contrarreferência

O protocolo de contrarreferência de pacientes com HA da atenção secundária e terciária para a APS varia conforme as diretrizes de saúde de cada localidade, mas, para que o cuidado ao paciente seja o melhor possível, deve incluir todos os passos listados no Quadro 14.10.^589^

**Quadro 14.10:** Elementos essenciais da contrarreferência

– Após a avaliação clínica e complementar do paciente, a melhor estabilização possível e o estabelecimento do plano terapêutico a longo prazo, o especialista deve reencaminhar o paciente à atenção primária (contrarreferência), local adequado de referência para o seu acompanhamento.
– Enviar um relatório detalhado para a equipe de saúde da atenção primária que contenha todas as informações relevantes do quadro clínico, resultados de exames realizados na atenção secundária ou terciária, medicações prescritas, plano terapêutico personalizado, orientações dietéticas e no estilo de vida e sugestão de periodicidade do acompanhamento na atenção primária e atenção secundária, caso necessário.
– Estabelecer uma forma de comunicação contínua entre os especialistas e a equipe de saúde da atenção primária para discutir casos complexos e ajustar tratamentos, conforme necessário.
– Garantir que o paciente tenha consultas agendadas para acompanhamento na atenção primária e na atenção secundária, se necessária.

### 14.13. Telemedicina na Linha de Cuidado da Hipertensão Arterial no Âmbito do Sistema Único de Saúde

Telemedicina tem como objetivo prestar serviços de saúde à distância utilizando tecnologias de comunicação e informação, como videoconferências, telefonemas e aplicativos de mensagens. A telemedicina surge como uma ferramenta poderosa para apoiar o manejo contínuo e personalizado da HA na APS do SUS, com benefícios para a equipe de saúde e usuários.^590-592^

A incorporação da telemedicina na linha de cuidado da HA no âmbito do SUS representa uma evolução no modelo de atenção, permitindo o acompanhamento mais próximo, contínuo e eficiente dos pacientes. O Quadro 14.11 resume as possíveis indicações para o uso da telemedicina no acompanhamento de pessoas com HA, que permitem ampliar, complementar e agilizar o cuidado oferecido pela equipe de saúde, incentivar o autocuidado e o apoio familiar, observando-se os preceitos éticos.

**Quadro 14.11:** Principais indicações para o uso da telemedicina na atenção primária à saúde do Sistema Único de Saúde

Indicações	Descrição
Identificação e cadastro de pessoas com HA	Possibilita a triagem de pessoas com fatores de risco para HA, a busca ativa, o diagnóstico e a estratificação do risco cardiovascular e renal precoces. Pacientes diagnosticados podem ser cadastrados no sistema de informação de saúde do SUS.
Monitoramento remoto	Acompanhamento regular pelo uso de dispositivos de monitoramento domiciliar da PA; orientações sobre a medida correta e o envio de resultados para a equipe de saúde via aplicativos ou plataformas do SUS. Permite o uso de alertas automáticos para identificar variações significativas na PA com necessidade de intervenção imediata.
Consultas e orientações à distância	Consultas periódicas à distância para avaliar a adesão ao tratamento, ajustar medicações e fornecer orientações sobre medidas não medicamentosas. Educação em saúde sobre a importância do controle da PA, dieta adequada, prática de atividades físicas e acolhimento das necessidades individuais.
Abordagem multidisciplinar	Permite que diferentes profissionais (nutricionistas, psicólogos, assistentes sociais) possam oferecer suporte adicional ao paciente. Os dados coletados devem ser integrados ao prontuário eletrônico do paciente, quando existente, favorecendo a abordagem ampliada do tratamento.
Suporte à decisão clínica e acompanhamento	Discussão e suporte à decisão clínica entre o médico generalista/médico da família e comunidade/equipe de saúde da atenção primária e o especialista.
Acompanhamento longitudinal e continuidade do cuidado	Permite elaborar planos de cuidado personalizados que possam ser revisados e atualizados remotamente, garantindo a continuidade do tratamento ao longo do tempo.

HA: hipertensão arterial; PA: pressão arterial; SUS: Sistema Único de Saúde.

**Table:** 

Mensagens principais sobre referência e contrarreferência de pacientes com hipertensão arterial e uso da telemedicina
Para garantir a sustentabilidade do sistema de saúde e o aumento da sua eficiência, é importante que os encaminhamentos para especialistas obedeçam a critérios objetivos e visem complementar e melhorar o cuidado prestado na atenção primária à saúde.
Após a avaliação clínica e complementar do paciente, a melhor estabilização possível e o estabelecimento do plano terapêutico a longo prazo, o especialista deve contrarreferenciar o paciente à atenção primária, local de referência para o seu acompanhamento.
O médico responsável pelo paciente é o generalista/médico da família e comunidade da atenção primária à saúde.
A incorporação da telemedicina na linha de cuidado da hipertensão arterial no âmbito do SUS representa uma evolução no modelo de atenção, permitindo o acompanhamento mais próximo, contínuo e eficiente dos pacientes.

SUS: Sistema Único de Saúde.

## 15. Perspectivas

### 15.1. Expossoma e Hipertensão Arterial

O termo expossoma foi designado para descrever o impacto total das exposições ambientais sobre a saúde e o bem-estar de um indivíduo.^593^ Diversos fatores que compõem o expossoma, como temperatura ambiental, poluição do ar ambiental, poluição sonora, presença de áreas verdes e caminhabilidade, têm sido implicados no desenvolvimento da HA (Figura 15.1).

**Figura 15.1:**
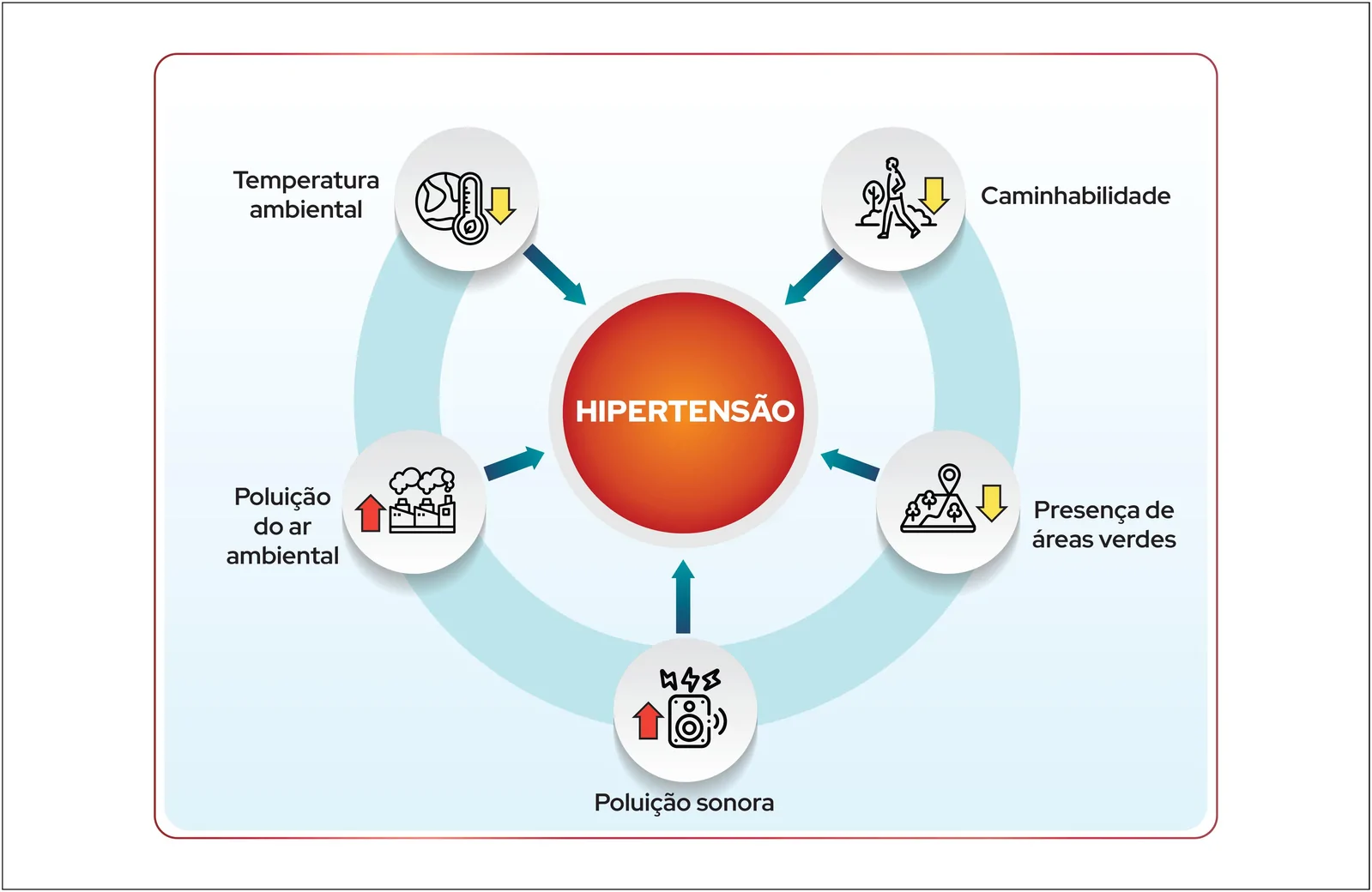
Relação entre componentes do expossoma e hipertensão arterial.

Temperaturas ambientais mais baixas promovem elevações da PA, enquanto temperaturas mais quentes induzem reduções da PA.^41,594^ Esses fatos contribuem para explicar as elevações da PA durante o inverno e as reduções da PA no verão.^41,594^ Além disso, cidades com temperaturas médias mais quentes apresentam maior prevalência de normotensão e HAB, enquanto cidades com temperaturas médias mais frias apresentam maior prevalência de HA sustentada e HM.^41^ Como o Brasil e o mundo estão testemunhando mudanças climáticas acentuadas e uma maior frequência de eventos climáticos extremos, é esperado que a influência da temperatura ambiental seja cada vez mais valorizada no manejo da HA nos próximos anos.

Diversos estudos têm demonstrado que níveis elevados de poluição do ar são acompanhados de maior incidência de HA.^595^ Particularmente, concentrações elevadas no ar de material particulado fino (PM2.5) e de óxidos de nitrogênio (NOx) apresentam forte relação com níveis aumentados de PA e maior incidência de HA.^595^ De maneira similar, locais com maior poluição sonora são acompanhados de maior prevalência de HA.^596^ Em contrapartida, a maior presença de vegetação ou áreas verdes tem sido associada com menor risco de HA.^597^

A caminhabilidade é um termo que engloba os aspectos que influenciam nos deslocamentos a pé no bairro ou na vizinhança. Neste sentido, locais que oferecem maior caminhabilidade estimulam a realização de AF durante os deslocamentos e, por conseguinte, têm sido associados com menor prevalência de HA.^593^

Esse conjunto de dados sugere que estratégias voltadas a reduzir a exposição dos indivíduos à poluição do ar ambiental e sonora e aumentar o espaço de áreas verdes e a caminhabilidade possam ser utilizadas no futuro como medidas de saúde pública adicionais que almejem reduzir a prevalência de HA do ponto de vista populacional.

### 15.2. Wearables e Telemonitoramento em Hipertensão

O avanço das tecnologias digitais na área da saúde tem transformado significativamente a abordagem da HA, permitindo um monitoramento mais contínuo e preciso da PA fora do ambiente de consultório. O telemonitoramento e os dispositivos vestíveis (*wearables*) têm sido progressivamente incorporados às estratégias de controle da HA, com potencial para melhorar a adesão ao tratamento e reduzir complicações CV.^44,598,599^ No entanto, desafios relacionados à validação, precisão e integração clínica dessas tecnologias ainda precisam ser superados para sua implementação mais ampla.^600^

Os *wearables* oferecem a possibilidade de medidas frequentes ou mesmo contínuas da PA, o que facilita a detecção precoce de fenótipos hipertensivos, como HM e variabilidade anormal da PA.^600,601^ Estudos demonstram que o monitoramento remoto da PA, quando associado a estratégias de saúde digital e telemedicina, pode levar a reduções significativas nos níveis de PA.^599^ Além disso, dispositivos que monitoram AF e sono, quando utilizados de forma integrada, podem auxiliar no controle da PA ao incentivar mudanças comportamentais e melhorar a adesão ao tratamento.^602,603^

A confiabilidade dos *wearables* para a medida da PA ainda é uma questão fundamental. Embora avanços tenham sido feitos, muitos dispositivos sem manguito ainda não possuem validação suficiente para uso clínico.^604^ Diretrizes internacionais, como as da Hypertension Canada e da European Society of Hypertension, recomendam cautela na adoção desses dispositivos, enfatizando a necessidade de validação rigorosa antes da aplicação clínica.^605,606^ Além disso, a variabilidade entre dispositivos e a influência de fatores como movimentação corporal e posicionamento dificultam a obtenção de medidas precisas.^73^ Devem ser considerados ainda, os desafios referentes à privacidade de dados e obstáculos regulatórios que precisam ser suplantados.^607^

O telemonitoramento da PA já demonstrou benefícios na prática clínica, especialmente quando combinado a intervenções multimodais que envolvem médicos, enfermeiros e farmacêuticos.^598,608^ A captura automática de dados e sua transmissão para plataformas digitais permitem um acompanhamento mais próximo, facilitando ajustes terapêuticos e a personalização do tratamento.^599^ Além disso, estratégias que utilizam inteligência artificial para a análise de tendências da PA podem contribuir para uma abordagem mais preditiva e preventiva.^609^

Pacientes com HA crônica e idosos parecem se beneficiar mais do uso de *wearables*, especialmente quando associados a programas estruturados de autogestão da HA.^610^ Além disso, o uso dessas tecnologias pode aumentar a AF, um componente fundamental para o controle da PA.^603^ No entanto, para que esses dispositivos sejam amplamente adotados, é essencial que os dados obtidos sejam comparáveis aos métodos tradicionais, garantindo confiabilidade diagnóstica e terapêutica.^73^

Portanto, embora os *wearables* e o telemonitoramento representem uma revolução potencial no cuidado da HA, sua incorporação plena na prática clínica dependerá de validação rigorosa, padronização das medições e integração eficaz com os sistemas de saúde.^605,609^ O futuro do controle da HA provavelmente envolverá uma abordagem híbrida, que combine tecnologia digital com acompanhamento clínico estruturado, transformando a assistência médica de reativa para proativa, permitindo diagnósticos de precisão, um manejo mais personalizado e eficiente da doença, além de estratégias preventivas.^610^

### 15.3. Hipertensão Arterial e Complacência Intracraniana

A complacência cerebral refere-se à capacidade dos vasos sanguíneos cerebrais de se dilatarem e se contraírem em resposta a alterações na PA, desempenhando um papel crucial na regulação do fluxo sanguíneo cerebral.

A associação entre a HA e doenças cerebrovasculares, assim como com o declínio cognitivo, é muito bem conhecida, e as bases fisiopatogênicas dessa associação são complexas e têm sido mais bem compreendidas. A HA promove mudanças estruturais em todo o leito vascular cerebral, e o conceito de pulsatilidade hemodinâmica, que engloba variabilidade pressórica, pressão central e rigidez arterial, foi identificado como um mecanismo que pode impactar a estrutura e a função cerebral.^611-613^

A avaliação do comportamento da complacência e pressão intracraniana era somente possível através de métodos invasivos. Entretanto, hoje existem métodos não invasivos e validados que permitem essa avaliação.^614^ Nesse contexto, um estudo em modelo animal mostrou alterações da complacência intracraniana apenas 3 semanas após a indução de HA.^615^ Mais recentemente, um estudo brasileiro mostrou uma prevalência de 45,6% de alteração da complacência intracraniana em indivíduos com HA acompanhados ambulatorialmente.^616^

Esses resultados servem como um alerta à comunidade científica e enfatizam a necessidade de combinarmos o conhecimento sobre o comportamento da PA periférica e central e o comportamento da complacência e da pressão intracraniana nos pacientes com HA. Além disso, esses dados parecem indicar que a regulação da circulação cerebral e da barreira hematoencefálica possam estar comprometidos em fases muito iniciais da doença hipertensiva.^617^

### 15.4. Combinações de Três ou Quatro Medicamentos em Doses Ultrabaixas

As baixas taxas de controle da PA no Brasil e no mundo^618^ demandam novas estratégias terapêuticas mais simples e efetivas que possam combater a baixa adesão ao tratamento e a inércia terapêutica.

A OMS^619^ e as diretrizes brasileira^1^ e internacionais^45,117^ recomendam o uso de combinações de medicamentos, preferencialmente em comprimido único, como estratégia inicial do tratamento para a grande maioria dos pacientes com HA. No entanto, o uso de monoterapia ainda permanece muito prevalente.^620^

A proposição do uso de combinações de três ou quatro medicamentos em doses ultrabaixas em comprimido único para o início de tratamento na HA não é tão recente. O racional inclui maior eficácia e tolerabilidade, maior adesão ao tratamento e menor inércia terapêutica.

Em 2017, uma RS e metanálise^621^ incluiu 42 ensaios envolvendo 20.284 participantes. Trinta e seis comparações avaliaram ¼ de dose em monoterapia vs. placebo, e a redução da PA observada foi de −4,7/−2,4 mmHg (p < 0,001). Seis comparações foram de terapia dupla com ¼ de dose vs. placebo, e a redução de PA foi de −6,7/−4,4 mmHg (p < 0,001). Não houve ensaios de combinação tripla com ¼ da dose vs. placebo, mas um estudo de terapia quádrupla com ¼ dose observou redução de PA de −22,4/−13,1 mmHg vs. placebo (p < 0,001). Na comparação com a monoterapia em dose padrão, terapias dupla e quádrupla em ¼ da dose mostraram diferenças de PA de +3,7/+2,6 (p < 0,001), +1,3/−0,3 (NS) e −13,1/−7,9 (p < 0,001) mmHg, respectivamente. Em termos de eventos adversos, a terapia de ¼ de dose em monoterapia ou terapia dupla não foi significativamente diferente do placebo, e foram observados menos eventos adversos em comparação com a monoterapia em dose padrão. Esses achados sugeriram que as combinações com ¼ de dose dos seus componentes poderiam fornecer melhores eficácia e tolerabilidade.^621^

Outra metanálise de 2023^622^ confirmou esses achados, incluindo estudos com combinação tripla e quádrupla com ¼ da dose de seus componentes. O desfecho primário foi a redução média da PAS na comparação dessas combinações com monoterapia em dose padrão, com tratamento usual ou com placebo. Foram incluídos sete estudos clínicos com 1.918 pacientes, e as combinações em ultrabaixas doses mostraram maior redução média da PAS do que a monoterapia inicial ou tratamento usual (redução média = 7,4 mmHg; IC 95% 4,3–10,5) ou placebo (redução média = 18,0 mmHg; IC 95% 15,1–20,8) em até 12 semanas de acompanhamento.

O Quadro 15.1 mostra as características e resultados dos principais ECRs sobre essa proposta de tratamento da HA com combinações triplas e quádruplas em doses ultrabaixas.

**Quadro 15.1:** Principais estudos clínicos randomizados em indivíduos com hipertensão arterial tratados com combinações de três ou quatro medicamentos em doses ultrabaixas

Estudo	Desenho do estudo (n)	Intervenção Estratégia terapêutica e doses	Comparador	Desfecho primário	Resultado
TRIUMPH^623^ (2018)	ECR, aberto (n = 700)	T 20 mg A 2,5 mg C 12,5 mg *(múltiplos comprimidos)*	Cuidado usual	Taxa de alcance da meta de PA < 140/90 mmHg ou < 130/80 mmHg no DM e DRC em 6 meses	Aumento da taxa de controle da PA com a combinação tripla vs. cuidado usual em 6 meses (70% vs. 55%, diferença = 12,7% [IC 95% 3,2–22,0%]; p < 0,001).
TRIUMPH^624^ (2020)	ECR, aberto (n = 700)	T 20 mg A 2,5 mg C 12,5 mg *(múltiplos comprimidos)*	Cuidado usual	Inércia terapêutica* em 12 semanas de tratamento	Inércia terapêutica foi mais frequente na combinação tripla do que no cuidado usual em 12 semanas (81 em 90 [90%] vs. 116 em 179 [64,8%]; p < 0,001).
Sung *et al*.^625^ (2022)	ECR duplo cego (n = 176)	T+A+C 10/1,25/3,125 mg – ¼ dose T+A+C 13,3/1,7/4,2 mg – ⅓ dose T+A+C 20/2,5/6,25 mg – ½ dose *(comprimido único)*	A 5 mg A 10 mg T 80 mg Placebo	Diferença na média da PAS sentada após 8 semanas	A média da PAS e da PAD sentadas foram significativamente menores com combinações triplas em ¼ dose e ½ dose, comparadas a placebo, A 5, A 10 e T 80.
QUARTET^626^ (2021)	ECR, duplo cego (n = 591)	Irb 37,5 mg A 1,25 mg I 0,625 mg B, 2,5 mg *(comprimido único)*	Irb 150 mg	Diferença na PAS de consultório sem supervisão em 12 semanas.	A PAS foi menor em 6,9 mmHg no grupo em uso da combinação quádrupla (IC 95% 4,9–8,9; p < 0,0001). As taxas de controle da PA foram maiores no grupo intervenção (76%) vs. grupo-controle (58%; RR 1,30, IC 95% 1,15–1,47; p < 0,0001).
QUARTET^627^ USA (2024)	ECR, duplo cego (n = 62)	Cand 2 mg A 1,25 mg I 0,625 mg B, 2,5 mg *(comprimido único)*	Cand 8 mg	Diferença na PAS de consultório em 12 semanas	Sem diferença significativa na PAS de −4,8 mmHg (IC 95% −10,8 a 1,3; p = 0,123) e maior redução média da PAD de −4,9 mmHg (IC 95% −8,6 a −1,3; p = 0,009) no grupo intervenção em comparação com o grupo-controle, em 12 semanas.
QUARTET^628^ Inércia terapêutica (2024)	ECR, duplo cego (n = 591)	Irb 37,5 mg A 1,25 mg I 0,625 mg B, 2,5 mg *(comprimido único)*	Irb 150 mg	Inércia terapêutica*	Inércia terapêutica ocorreu em número menor de pacientes randomizados para a quadripílula comparados aos em monoterapia.
Ojii *et al*. NIGÉRIA^629^ (2024)	ECR, duplo cego (n = 300)	Combinação tripla – titulação acelerada T+A+I 10/1,25/0,625 mg T+A+I 20/2,5/1,25 mg T+A+I 40/5/2,5 mg *(comprimido único)*	Tratamento padrão A 5 mg A 5 + L 50 mg A 10 + L 100 mg A + L + H	Redução da PA residencial em 6 meses	Combinação tripla mostrou redução da PAS residencial de 31 mmHg (IC 95% 28–33 mmHg) vs. 26 mmHg (IC 95% 22–28 mmHg) no grupo de tratamento padrão (diferença ajustada = −5,8 mmHg [IC 95% −8,0 a −3,6; p < 0,001]), em 6 meses.

A: anlodipina; B: bisoprolol; C: clortalidona; Cand: candesartana; DM: diabetes mellitus; DRC: doença renal crônica; ECR: estudo clínico randomizado; HA: hipertensão arterial; I: indapamida; Irb: irbesartana; L: losartana; PA: pressão arterial; PAD: pressão arterial diastólica; PAS: pressão arterial sistólica; QUARTET: Quadruple Ultra-Low-Dose Treatment for Hypertension; RR: risco relativo; T: telmisartana; TRIUMPH: Treating Resistant Hypertension Using Lifestyle Modification to Promote Health.

* Não intensificar o tratamento em pacientes com PA não controlada

Os resultados dos estudos clínicos apontam para uma maior eficácia anti-hipertensiva e maior tolerabilidade das combinações triplas e quádruplas em doses ultrabaixas comparadas a placebo, monoterapia ou cuidado usual. Mais estudos são necessários, com maior número de pacientes e de mais longo prazo, que permitam avaliar o impacto desta estratégica terapêutica em doses ultrabaixas na proteção de órgãos-alvo e na proteção contra desfechos CV maiores.

### 15.5. Novos Medicamentos para o Tratamento da Hipertensão Arterial

Atualmente, várias classes de medicamentos estão sendo estudadas para o tratamento da HA, visando melhorar o controle da PA e reduzir os riscos CV e renais residuais.

Foram identificadas sete classes terapêuticas diferentes, que são opções emergentes, algumas bastante promissoras, para o tratamento de HAR e HARf. São elas: os antagonistas dos receptores mineralocorticoides não esteroidais, inibidores da aminopeptidase A, antagonistas duplos da endotelina, atenuadores do angiotensinogênio hepático, inibidores duais do receptor de angiotensina II-neprilisina e inibidores da aminopeptidase.^547,630-632^

O Quadro 15.2 resume os principais achados com esses novos medicamentos, que podem estar nas perspectivas de tratamento nos próximos anos e serem incorporados à prescrição medicamentosa do tratamento da HA.

**Quadro 15.2:** Novas estratégias medicamentosas em hipertensão arterial

MEDICAMENTOS	RESULTADOS DOS PRINCIPAIS ESTUDOS
**1. Inibidor de neprilisina + bloqueador dos receptores AT1 da angiotensina II**	
Sacubitril/valsartana^550^	Redução significativa da pressão central, PA de 24 horas e noturna com reversão do descenso noturno. Indicado para ICFEr e HA com IC (prevenção secundária). Não associar com IECA ou BRA.
**2. Antagonistas dos receptores mineralocorticoides não esteroidais**	
Finerenona^236,551^	Reduz eventos renais e CV em pacientes com diabetes quando adicionada à inibição máxima tolerada do SRAA. Menor efeito anti-hipertensivo que a espironolactona.
Apararenona, esaxerenona e ocedurenona^633-635^	Esses três ARMs estão em diferentes fases de desenvolvimento. • A esaxerenona reduziu a PA, diminuiu a albuminúria e mostrou-se bem tolerada e efetiva para o controle da HA noturna, especialmente em idosos. • O estudo BLOCK-CKD demonstrou que a ocedurenona é eficaz na redução da PAS em diversos subgrupos de pacientes, incluindo aqueles com diabetes, albuminúria muito alta e diferentes estágios de DRC. Em estudo de fase II com pacientes com HAR, a ocedurenona (KBP-5074) 0,5 mg reduziu a PA no consultório de −6,9 a −13,1 mmHg, dependendo da dose e do subgrupo com perfil de segurança favorável.^636^ • A apararenona é o ARM não esteroidal que ainda precisa de estudos clínicos para que se determinem sua eficácia, tolerabilidade e segurança.^637^
**3. Inibidores da aldosterona sintase (inibidores seletivos da enzima CYP11B2)** ^638^	
Baxdrostat^552,639^	BrigHTN1: estudo fase II – eficácia e segurança do baxdrostat, em pacientes com HAR. A PAS diminuiu em −20,3, −17,5 e −12,1 mmHg com 2, 1 e 0,5 mg de baxdrostat, respectivamente, vs. −9,4 mmHg no grupo placebo. Boa tolerabilidade e segurança. Aumento dos níveis séricos de potássio dose-dependente. Ensaio clínico de fase III em andamento – dose de 1 e 2 mg vs. placebo em HAR.
Lorundrostat^640-643^	Target-HTN: fase II. Reduziu a PA em um pequeno estudo em pacientes com HAR. Seis participantes tiveram hiperpotassemia > 6,0 mEq/L. O ECR Launch-HTN, com 1.083 pacientes, mostrou queda significante da PAS automatizada de consultório no grupo de tratamento (-16,9 mmHg) vs. −7,9 mmHg no grupo placebo, mas com 50% de eventos adversos leves e moderados.
Dexfadrostat	Foi testado em pequeno grupo de pacientes com hiperaldosteronismo primário com resultados promissores^644^
Vicadrostat	Ainda em estudos de fase 1 em voluntários normais
**4. Antagonista duplo dos receptores de endotelina ETA/ETB**	
Aprocitentano^554,645^	A PAS de consultório reduziu 15,3 mmHg (dose 12,5 mg) e 15,2 mmHg (dose 25 mg) e 11,5 mmHg (placebo) (p = 0,005) no estudo PRECISION. Efeito colateral: edema leve a moderado em 9% (dose 12,5 mg) e 18% (dose 25 mg). Aprovado pela FDA em março de 2024 em combinação com outros anti-hipertensivos.^646^
**5. RNA de interferência: inibe a produção de angiotensinogênio no fígado**	
Zilebesiran^556,647^	Oligonucleotídeos antisense que inibem a tradução de RNA e reduzem a síntese de angiotensinogênio hepático. Administração subcutânea (dose 250 a 500 mg) a cada 3 a 6 meses. • Estudo de fase 1: redução da PA de 24 horas por 24 semanas. Reações leves no local da injeção. • Estudo de fase II (Kardia-1): reduções sustentadas do angiotensinogênio e da PAS de 24 h (-12,84 mmHg) em pacientes com HA leve e moderada por até 6 meses.
IONIS-AGT-LRx^557^	Inibidor antisense administrado por via subcutânea. Os estudos iniciais demonstraram segurança e redução significativa dos níveis de angiotensinogênio em comparação com o placebo, além de tendências na redução da PA.
**6. Inibidores da aminopeptidase A cerebral**	
Firibastat^648,649^	A aminopeptidase A é a enzima responsável por sintetizar a angiotensina III e se constitui um alvo potencial para o tratamento da HA. Mostrou-se seguro e eficaz nos ensaios de fase 2 e está planejado para um ensaio de fase 3.
**7. Inibidores de SGLT2**	
Empaglifozina e dapaglifozina^650,651^	Várias metanálises confirmaram a capacidade dos inibidores de SGLT2 em reduzir a PA de modo discreto em pacientes com DM2. É um efeito de classe. Em uma delas, com 43 ECRs incluindo 22.528 pacientes, o efeito anti-hipertensivo foi de −2,5 mmHg na PAS e −1,5 mmHg da PAD. Outra análise de sete ECRs incluindo 2.381 participantes definiu os efeitos dos inibidores de SGLT2 na MAPA de 24 horas. A mudança foi de −3,6 mmHg na PAS e de −1,7 mmHg na PAD de 24 horas na MAPA, independentemente da dose do inibidor de SGLT2.

ARM: antagonista dos receptores mineralocorticoides; BLOCK-CKD: Blood Pressure in Chronic Kidney Disease; BRA: bloqueador dos receptores da angiotensina; BrigHTN1: Baxdrostat in Resistant Hypertension; CV: cardiovascular; DM2: diabetes mellitus 2; DRC: doença renal crônica; ECR: ensaio clínico randomizado; ETA: receptor de endotelina A (endothelin receptor A); ETB: receptor de endotelina B (endothelin receptor B); FDA: Food and Drug Administration; HA: hipertensão arterial; HAR: hipertensão arterial resistente; IC: insuficiência cardíaca; ICFEr: insuficiência cardíaca com fração de ejeção reduzida; IECA: inibidor da enzima conversora da angiotensina; MAPA: medida ambulatorial da pressão arterial; PA: pressão arterial; PAD: pressão arterial diastólica; PAS: pressão arterial sistólica; RNA: ácido ribonucleico (ribonucleic acid); SGLT2: cotransportador de sódio-glicose 2 (sodium/glucose cotransporter 2); SRAA: sistema renina-angiotensina-aldosterona; Target-HTN: Trial on the Safety and Efficacy of MLS-101 in Patients With Uncontrolled Hypertension.

## Lista de abreviaturas e siglas

A: Anlodipina
AAS: Ácido Acetilsalicílico
ACCORD: Action to Control Cardiovascular Risk in Diabetes
ACS: Agente Comunitário de Saúde
ACTH: Adrenocorticotropina
ADVANCE: Action in Diabetes and Vascular Disease: Preterax and Diamicron MR Controlled Evaluation
AF: Atividade Física
AGA: Avaliação Geriátrica Ampla
AHA: American Heart Association
AHCO: Anticoncepcional Hormonal Combinado Oral
AIT: Ataque Isquêmico Transitório
AMPA: Automedida da Pressão Arterial
AOS: Apneia Obstrutiva do Sono
APP: Aplicativos de Celular
APS: Atenção Primária à Saúde
ARIC: Atherosclerosis Risk in Communities
ARM: Antagonistas dos Receptores Mineralocorticoides
ARTS-DN: Mineralocorticoid Receptor Antagonist Tolerability Study–Diabetic Nephropathy
AV: Atrioventricular
AVC: Acidente Vascular cerebral
AVCH: Acidente Vascular Cerebral Hemorrágico
AVCI: Acidente Vascular Cerebral Isquêmico
B: Bisoprolol
BAV: Bloqueio Atrioventricular
BB: Betabloqueador
BCC: Bloqueador dos Canais de Cálcio
BiPAP: Bilevel Positive Airway Pressure
BLOCK-CKD: Blood Pressure in Chronic Kidney Disease
BNP/NT-proBNP: Peptídeos Natriuréticos Tipo B
BPM: Batimentos por Minuto
BPROAD: Intensive Blood-Pressure Control in Patients with Type 2 Diabetes
BRA: Bloqueador dos Receptores AT1 da Angiotensina II
BrigHTN1: Baxdrostat in Resistant Hypertension
C: Clortalidona
CA: Circunferência Abdominal
Cand: Candesartana
CC: Circunferência da Cintura
CID: Classificação Estatística Internacional de Doenças e Problemas Relacionados com a Saúde
CKD-EPI: Chronic Kidney Disease Epidemiology Collaboration
CPAP: Continuous Positive Airway Pressure
CRIC: The Chronic Renal Insufficiency Cohort Study
CTD: Clortalidona
CV: Cardiovascular
DAC: Doença Arterial Coronariana
DAOP: Doença Arterial Obstrutiva Periférica
DASH: Dietary Approaches to Stop Hypertension
DBHA: Diretriz Brasileira de Hipertensão Arterial
DCV: Doença Cardiovascular
DEXA: Dual-Energy X-Ray Absorptiometry Scanning
DIU: Diurético
DM: Diabetes Mellitus
DMP: Diferença de Média Padronizada
DRC: Doença Renal Crônica
DSR: Denervação Simpática Renal
EAP: Edema Agudo de Pulmão
ECA: Enzima Conversora da Angiotensina
ECG: Eletrocardiograma
ECO: Ecocardiograma
ECR: Ensaio Clínico Randomizado
EF: Exercício Físico
EH: Emergência Hipertensiva
ESC: European Society of Cardiology
ESF: Equipe de Saúde da Família
ESH: European Society of Hypertension
ESO: European Stroke Organisation
ESPRIT: Effects of Intensive Systolic Blood Pressure Lowering Treatment in Reducing Risk of Vascular Events
ETA: receptor de endotelina A
ETB: receptor de endotelina B
EV: Endovenosa
FA: Fibrilação Atrial
FC: Frequência Cardíaca
FDA: Food and Drug Administration
FO: Fundo de Olho
FRCV: Fatorde Risco Cardiovascular
FROM-J: Frontier of Renal Outcome Modifications in Japan
GH: Hormônio do Crescimento
GLP-1: Glucagon-Like Peptide-1
GRADE: Grading of Recommendations Assessment, Development and Evaluation
HA: Hipertensão Arterial
HAA/M: Hipertensão Acelerada-Maligna
HAB: Hipertensão do Avental Branco
HABNC: Hipertensão do Avental Branco não Controlada
HAM: Hipertensão Arterial Maligna
HAR: Hipertensão Arterial Resistente
HARf: Hipertensão Arterial Refratária
HbA1c: Hemoglobina Glicada
HCTZ: Hidroclorotiazida
HD: Hemodiálise
HDLc: High-Density Lipoprotein Cholesterol
HELLP: Hemólise, Elevação de Enzimas Hepáticas e Plaquetopenia
HM: Hipertensão Mascarada
HMNC: Hipertensão Mascarada não Controlada
HMOD: Lesão de Órgão-alvo Mediada pela Hipertensão
HOPE-3: Heart Outcomes Prevention Evaluation 3
HVE: Hipertrofia Ventricular Esquerda
HYVET: Hypertension in the Very Elderly Trial
IAH: Índice de Apneia e Hipopneia
IAM: Infarto Agudo do Miocárdio
IC: Insuficiência Cardíaca
ICC: Insuficiência Cardíaca Congestiva
ICFEp: Insuficiência Cardíaca com Fração de Ejeção Preservada
ICFEr: Insuficiência Cardíaca com Fração de Ejeção Reduzida
IDNT: Irbesartan Diabetic Nephropathy Trial
IECA: Inibidor da Enzima Conversora da Angiotensina
IM: Infarto do Miocárdio
IMC: Índice de Massa Corporal
IMVE: Índice de Massa Ventricular Esquerda
IR: injúria Renal
IRA: Injúria Renal Aguda
IRB: Irbesartana
IRC: Insuficiência Renal Crônica
iSGLT2: Sodium/Glucose Cotransporter 2 Inhibitors
ISH: International Society of Hypertension
ITB: Índice Tornozelo-Braquial
IV: Intravenosa
KDIGO: Kidney Disease Improving Global Outcomes
KWB: Classificação de Keith–Wagener–Barker
L: Losartana
LDLc: Low-Density Lipoprotein Cholesterol
LOA: Lesão de Órgãos-Alvo
LSD: Dietilamida do Ácido Lisérgico
MAPA: Monitoramento Ambulatorial da Pressão Arterial
MDMA: Metilenodioximetanfetamina
MDO: Múltiplos Danos a Órgãos-Alvo
MET: Metabolic Equivalent of Task
MFC: Médico de Família e Comunidade
MNM: Medidas não Medicamentosas
MRPA: Medida Residencial da Pressão Arterial
NHANES: National Health and Nutrition Examination Survey
NIHSS: National Institutes of Health Stroke Scale
NPS: Nitroprussiato de Sódio
O2: Oxigênio
OMS: Organização Mundial da Saúde
ONTARGET: Ongoing Telmisartan Alone and in combination with Ramipril Global Endpoint Trial
P: Percentil
PA: Pressão Arterial
PAD: Pressão Arterial Diastólica
PAM: Pressão Arterial Média
PAS: Pressão Arterial Sistólica
PCDT: Protocolos Clínicos e Diretrizes Terapêuticas
PE: Pré-Eclâmpsia
PLGF: Placental Growth Factor
PP: Pressão de Pulso
PRES: Posterior Reversible Encephalopathy Syndrome
PREVENT: Predicting Risk Of Cardiovascular Disease Events
PTH: Paratormônio
QUARTET: Quadruple Ultra-Low-Dose Treatment for Hypertension
RAC: Relação Albuminúria/Creatininúria
RCV: Risco Cardiovascular
ReHOT: Resistant Hypertension Optimal Treatment
RENAME: Relação Nacional de Medicamentos Essenciais
RESPECT: Randomized Evaluation of Recurrent Stroke Comparing PFO Closure to Established Current Standard of Care Treatment
RM: Repetição Máxima
RNA: Ácido Ribonucleico
RNM: Ressonância Nuclear Magnética
RR: Risco Relativo
RS: Revisão Sistemática
RVP: Resistência Vascular Periférica
SAMU: Serviço de Atendimento Móvel de Urgência
SBC: Sociedade Brasileira de Cardiologia
SBH: Sociedade Brasileira de Hipertensão
SBN: Sociedade Brasileira de Nefrologia
SC: Superfície Corpórea
SCA: Síndrome Coronariana Aguda
SCORE2: Systematic Coronary Risk Evaluation 2
SGLT2: Cotransportador de Sódio-Glicose Tipo 2
SHEP: Systolic Hypertension in the Elderly Program
SNC: Sistema Nervoso Central
SNPS: Sistema Nervoso Parassimpático
SNS: Sistema Nervoso Simpático
SPRINT: Systolic Blood Pressure Intervention Trial
SPRINT-MIND: Systolic Blood Pressure Intervention Trial – Memory and Cognition in Decreased Hypertension
SRAA: Sistema Renina-Angiotensina-Aldosterona
STEP: Semaglutide Treatment Effect in People with Obesity
SUS: Sistema Único de Saúde
SYST-EUR: Systolic Hypertension in Europe
T: Telmisartana
Target-HTN: Trial on the Safety and Efficacy of MLS-101 in Patients With Uncontrolled Hypertension
TC: Tomografia Computadorizada
TFGe: Taxa de Filtração Glomerular Estimada
TNT: Treating to New Targets
TRIDENT: Triple Therapy Prevention of Recurrent Intracerebral Disease Events Trial
TRIUMPH: Treating Resistant Hypertension Using Lifestyle Modification to Promote Health
TSH: Hormônio Tireoestimulante
UBS: Unidade Básica de Saúde
UPA: Unidade de Pronto Atendimento
USG: Ultrassonografia
UTI: Unidade de Terapia Intensiva
VIGITEL: Vigilância de Fatores de Risco e Proteção para Doenças Crônicas por Inquérito Telefônico
VM: Velocidade de Marcha
VOP: Velocidade de Onda de Pulso
