# Supplementary material for: Diretriz Brasileira de Hipertensão Arterial – 2025
Source: Arq Bras Cardiol. 2025 Sep 18;122(9):e20250624. [Article in Portuguese] doi: 10.36660/abc.20250624 (PMC13671149; doi:10.36660/abc.20250624)
Supplement: Translation

# Translation: Brazilian Guidelines of Hypertension – 2025

**DOI:** 10.36660/abc.20250624i

**Authors:** Andréa Araujo Brandão, Cibele Isaac Saad Rodrigues, Luiz Aparecido Bortolotto, Anderson da Costa Armstrong, Rogério Andrade Mulinari, Audes Diógenes de Magalhães Feitosa, Marco Antonio Mota-Gomes, Eduardo Costa Duarte Barbosa, José Andrade Moura, Mario Fritsch Toros Neves, Kunz Sebba Barroso Weimar, Claudia Lucia de Moraes Forjaz, Márcia Regina Simas Torres Klein, Fernando Nobre, Paulo César Brandão Veiga Jardim, Celso Amodeo, Rogério Baumgratz de Paula, Sebastião Rodrigues Ferreira, João Roberto Gemelli, José Fernando Vilela-Martin, Elizabeth Silaid Muxfeldt, Décio Mion, Marcus Vinicius Bolivar Malachias, Carlos Alberto Machado, Fernando Antonio de Almeida, Alexandre Jorge Gomes de Lucena, Alvaro Avezum, Amaury Zatorre Amaral, Ana Flavia Moura, Ana Lúcia Rego Fleury de Camargo, Andrea Pio de Abreu, Andrei C. Sposito, Angela Maria Geraldo Pierin, Annelise Machado Gomes de Paiva, Antonio Gabriele Laurinavicius, Carlos Eduardo Poli-de-Figueiredo, Dilma do Socorro Moraes de Souza, Elizabete Viana de Freitas, Emilton Lima, Erika Maria Gonçalves Campana, Fábio Argenta, Fernanda Marciano Consolim Colombo, Fernanda Salomão Gorayeb Polacchini, Flavio Antonio de Oliveira Borelli, Frida Liane Plavnik, Giovanio Vieira da Silva, Grazia Maria Guerra, Heno Ferreira Lopes, José Augusto Soares Barreto, Juan Carlos Yugar Toledo, Leda Aparecida Daud Lotaif, Lílian Soares da Costa, Lucelia Batista Neves Cunha Magalhães, Luciano Ferreira Drager, Luis Cuadrado Martin, Luiz César Nazário Scala, Maria Eliane Campos Magalhães, Maria Eliete Pinheiro, Maria Emília Figueiredo Teixeira, Nelson Dinamarco, Osni Moreira, Oswaldo Passarelli, Otavio Rizzi Coelho, Renault Mattos Ribeiro, Roberto Dischinger Miranda, Rodrigo Bezerra, Rodrigo Pinto Pedrosa, Rogério Toshiro Passos Okawa, Rui Manuel dos Santos Povoa, Sandra Lia do Amaral Cardoso, Sayuri Inuzuka, Sergio Emanuel Kaiser, Sheyla Cristina Tonheiro Ferro da Silva, Tânia Plens Shecaira, Vera Hermina Kalika Koch, Wilson Nadruz

**Affiliations:** 1 Rio de Janeiro RJ Brazil Universidade do Estado do Rio de Janeiro (UERJ), Rio de Janeiro, RJ – Brazil; 2 Sorocaba SP Brazil Pontifícia Universidade Católica de São Paulo, Faculdade de Ciências Médicas e da Saúde, Sorocaba, SP – Brazil; 3 São Paulo SP Brazil Instituto do Coração (Incor) do Hospital das Clínicas da Faculdade de Medicina da Universidade de São Paulo (HCFMUSP), São Paulo, SP – Brazil; 4 Petrolina PE Brazil Universidade Federal do Vale do São Francisco, Petrolina, PE – Brazil; 5 Curitiba PR Brazil Universidade Federal do Paraná (UFPR),^5^ Curitiba, PR – Brazil; 6 Recife PE Brazil Universidade Federal de Pernambuco, Recife, PE – Brazil; 7 Maceió AL Brazil Centro Universitário CESMAC, Maceió, AL – Brazil; 8 Maceió AL Brazil Hospital do Coração de Alagoas, Maceió, AL – Brazil; 9 Maceió AL Brazil Centro de Pesquisas Clínicas Dr. Marco Mota, Maceió, AL – Brazil; 10 Porto Alegre RS Brazil Universidade FEEVALE, Porto Alegre, RS – Brazil; 11 Salvador BA Brazil Escola Bahiana de Medicina e Saúde Pública (EBMSP), Salvador, BA – Brazil; 12 Goiânia GO Brazil Faculdade de Medicina da Universidade Federal de Goiás (UFG), Goiânia, GO – Brazil; 13 São Paulo SP Brazil Escola de Educação Física e Esporte da Universidade de São Paulo (USP), São Paulo, SP – Brazil; 14 Ribeirão Preto SP Brazil Hospital das Clínicas da Faculdade de Medicina de Ribeirão Preto da Universidade de São Paulo, Ribeirão Preto, SP – Brazil; 15 São Paulo SP Brazil Hcor, Associação Beneficente Síria, São Paulo, SP – Brazil; 16 Juiz de Fora MG Brazil Universidade Federal de Juiz de Fora, Juiz de Fora, MG – Brazil; 17 Uberlândia MG Brazil Universidade Federal de Uberlândia, Uberlândia, MG – Brazil; 18 Porto Velho RO Brasil Governo do Estado de Rondônia, Porto Velho, RO – Brasil; 19 José do Rio Preto SP Brazil Faculdade de Medicina de São José do Rio Preto (FAMERP), São José do Rio Preto, SP – Brazil; 20 Rio de Janeiro RJ Brazil Hospital Universitário Clementino Fraga Filho, Universidade Federal do Rio de Janeiro (UFRJ), Rio de Janeiro, RJ – Brazil; 21 São Paulo SP Brazil Hospital das Clínicas da Faculdade de Medicina da Universidade de São Paulo (HCFMUSP), São Paulo, SP – Brazil; 22 Belo Horizonte MG Brazil Faculdade Ciências Médicas de Minas Gerais, Belo Horizonte, MG – Brazil; 23 Belo Horizonte MG Brazil Fundação Educacional Lucas Machado (FELUMA), Belo Horizonte, MG – Brazil; 24 Belo Horizonte MG Brazil Instituto de Hipertensão de Minas Gerais, Belo Horizonte, MG – Brazil; 25 Brasília DF Brazil Ministério da Saúde, Brasília, DF – Brazil; 26 Sorocaba SP Brazil Fundação São Paulo, Pontifícia Universidade Católica de São Paulo, Sorocaba, SP – Brazil; 27 Recife PE Brazil Hospital Agamenom Magalhães, Recife, PE – Brazil; 28 São Paulo SP Brazil Hospital Alemão Oswaldo Cruz, São Paulo, SP – Brazil; 29 São Paulo SP Brazil Universidade de São Paulo (USP), São Paulo, SP – Brazil; 30 São Paulo SP Brazil Universidade Federal de São Paulo (UNIFESP), São Paulo, SP – Brazil; 31 Ontorio Canada PHRI-McMaster University, Ontorio – Canada; 32 São Paulo SP Brazil Secretaria Municipal de Saúde de São Paulo, São Paulo, SP – Brazil; 33 Campinas SP Brazil Universidade Estadual de Campinas (UNICAMP), Campinas, SP – Brazil; 34 Maceió AL Brazil Centro de Pesquisas Clínicas Dr. Marco Mota, Maceió, AL – Brazil; 35 São Paulo SP Brazil Instituto Dante Pazzanese de Cardiologia, São Paulo, SP – Brazil; 36 Porto Alegre RS Brazil Pontifícia Universidade Católica do Rio Grande do Sul, Porto Alegre, RS – Brazil; 37 Belém PA Brazil Universidade Federal do Pará (UFPA), Belém, PA – Brazil; 38 Belém PA Brazil Fundação Pública Estadual Hospital de Clínicas Gaspar Vianna, Belém, PA – Brazil; 39 Curitiba PR Brazil Universidade Federal do Paraná, Curitiba, PR – Brazil; 40 Cuiabá MT Brazil Instituto Dr. Fábio Argenta, Cuiabá, MT – Brazil; 41 São José do Rio Preto SP Brazil Hospital de Base de São José do Rio Preto, São José do Rio Preto, SP – Brazil; 42 Rio de Janeiro RJ Brazil Rede D’Or São Luiz, Rio de Janeiro, RJ – Brazil; 43 São Paulo SP Brazil Universidade Cruzeiro do Sul (UNCSUL), São Paulo, SP – Brazil; 44 São Paulo SP Brazil Faculdade Beneficência Portuguesa, São Paulo, SP – Brazil; 45 Aracaju SE Brazil Universidade Federal de Sergipe, Aracaju, SE – Brazil; 46 São José do Rio Preto SP Brazil Faculdade de Medicina de São José do Rio Preto (FAMERP), São José do Rio Preto, SP – Brazil; 47 São José do Rio Preto SP Brazil ENDOCOR, São José do Rio Preto, SP – Brazil; 48 Rio de Janeiro RJ Brazil Escola de Ciências da Saúde e Bem-estar, Faculdade Souza Marques, Rio de Janeiro, RJ – Brazil; 49 Rio de Janeiro RJ Brazil Universidade Estácio de Sá, Rio de Janeiro, RJ – Brazil; 50 Rio de Janeiro RJ Brazil Instituto Estadual de Cardiologia Aloysio de Castro – IECAC, Rio de Janeiro, RJ – Brazil; 51 Salvador BA Brazil Faculdade Zarns, Salvador, BA – Brazil; 52 São Paulo SP Brazil Universidade Estadual Paulista (UNESP), São Paulo, SP – Brazil; 53 Cuiabá MT Brazil Universidade Federal de Mato Grosso, Cuiabá, MT – Brazil; 54 Rio de Janeiro RJ Brazil Hospital Universitário Pedro Ernesto, Universidade do Estado do Rio de Janeiro (UERJ), Rio de Janeiro, RJ – Brazil; 55 Maceió AL Brazil Universidade Federal de Alagoas, Maceió, AL – Brazil; 56 Ilhéus BA Brazil Universidade Estadual de Santa Cruz (UESC), Ilhéus, BA – Brazil; 57 Curitiba PR Brazil Clínica Gapski Moreira, Curitiba, PR – Brazil; 58 Brasília DF Brazil Cardios Vita, Brasília, DF – Brazil; 59 Brasília DF Brazil Hospital Santa Lucia,^59^ Brasília, DF – Brazil; 60 Recife PE Brazil Pronto Socorro Cardiológico de Pernambuco (PROCAPE), Recife, PE – Brazil; 61 Maringá PR Brazil Universidade Estadual de Maringá, Maringá, PR – Brazil; 62 São Paulo SP Brazil Universidade Estadual Paulista, Campus de Bauru, São Paulo, SP – Brazil; 63 Goiânia GO Brazil Hospital de Acidentados, Goiânia, GO – Brazil; 64 Aracaju SE Brazil CEMISE Oncoclínicas, Aracaju, SE – Brazil; 65 Aracaju SE Brazil Prefeitura Municipal de Aracaju, Aracaju, SE – Brazil

## Chapters and participations:

**1. INTRODUCTION**
  **Members:** Andréa Araujo Brandão, Cibele Isaac Saad Rodrigues, Luiz Aparecido Bortolotto, Wilson Nadruz
**2. EPIDEMIOLOGY, DEFINITION, AND PRIMARY PREVENTION**
  **Coordenators:** Anderson da Costa Armstrong, Rogério Andrade Mulinari
  **Members:** Alvaro Avezum Junior, Dilma do Socorro Moraes de Souza, Leda Aparecida Daud Lotaif, Lucelia Batista Neves Cunha Magalhães, Luiz César Nazário Scala
**3. BLOOD PRESSURE MEASUREMENT, DIAGNOSIS, AND CLASSIFICATION**
  **Coordenators:** Audes Diógenes de Magalhães Feitosa, Marco Antonio Mota-Gomes
  **Members:** Angela Maria Geraldo Pierin, Annelise Machado Gomes de Paiva, Lílian Soares da Costa, Nelson Dinamarco, Rodrigo Bezerra
**4. CLINICAL AND COMPLEMENTARY ASSESSMENT AND CARDIOVASCULAR RISK STRATIFICATION**
  **Coordenators:** Eduardo Costa Duarte Barbosa, José Andrade Moura-Neto
  **Members:** Andrei C. Sposito, Antonio Gabriele Laurinavicius, Maria Eliane Campos Magalhães, Rodrigo Pinto Pedrosa, Rogério Toshiro Passos Okawa, Rui Manuel dos Santos Povoa, Sayuri Inuzuka
**5. TREATMENT INITIATION AND THERAPEUTIC TARGETS**
  **Coordenators:** Mario Fritsch Toros Neves, Weimar Kuns Sebba Barroso de Souza
  **Members:** Ana Flavia Moura, Frida Liane Plavnik, Luciano Ferreira Drager, Osni Moreira Filho
**6. NONPHARMACOLOGICAL MEASURES**
  **Coordenators:** Claudia Lucia de Moraes Forjaz, Márcia Regina Simas Torres Klein
  **Members:** Grazia Guerra, Maria Eliete Pinheiro, Maria Emília Figueiredo Teixeira, Tânia Plens Shecaira
**7. PHARMACOLOGICAL TREATMENT**
  **Coordenators:** Fernando Nobre, Paulo César Brandão Veiga Jardim
  **Members:** Fernanda Marciano Consolim Colombo, Otavio Rizzi Coelho, Sergio Emanuel Kaiser
**8. HYPERTENSION AND ASSOCIATED CLINICAL CONDITIONS: CORONARY ARTERY DISEASE, CHRONIC KIDNEY DISEASE, DIABETES, OBESITY, COVID-19, POST-STROKE, AND HEART FAILURE**
  **Coordenators:** Celso Amodeo, Rogério Baumgratz de Paula
  **Members:** Andrea Pio de Abreu, Emilton Lima Júnior, Heno Ferreira Lopes
**9. HYPERTENSION IN OLDER ADULTS, CHILDREN, AND ADOLESCENTS**
  **Coordenators:** Sebastião Rodrigues Ferreira-Filho
  **Members:** Alexandre Jorge Gomes de Lucena, Carlos Eduardo Poli de Figueiredo, Elizabete Viana de Freitas, Roberto Dischinger Miranda, Sheyla Cristina Tonheiro Ferro da Silva, Vera Hermina Kalika Koch
**10. HYPERTENSION IN WOMEN**
  **Coordenators:** Sebastião Rodrigues Ferreira-Filho
  **Members:** Alexandre Jorge Gomes de Lucena, Carlos Eduardo Poli-de-Figueiredo, Elizabete Viana de Freitas, Roberto Dischinger Miranda, Sheyla Cristina Tonheiro Ferro da Silva, Vera Hermina Kalika Koch
**11. HYPERTENSIVE CRISIS**
  **Coordenators:** João Roberto Gemelli, José Fernando Vilela-Martin
  **Members:** Fábio Argenta, Fernanda Salomão Gorayeb Polacchini, Luis Cuadrado Martin, Renault Mattos Ribeiro Junior
**12. RESISTANT AND REFRACTORY HYPERTENSION**
  **Coordenators:** Elizabeth Silaid Muxfeldt
  **Members:** Erika Maria Gonçalves Campana, Flavio Antonio de Oliveira Borelli, Juan Carlos Yugar Toledo, Oswaldo Passarelli Junior
**13. ADHERENCE TO ANTIHYPERTENSIVE TREATMENT**
  **Coordenators:** Decio Mion Júnior, Marcus Vinicius Bolivar Malachias
  **Members:** Ana Lúcia Rego Fleury de Camargo, Giovanio Vieira da Silva, Sandra Lia do Amaral Cardoso
**14. BEST PRACTICES IN THE CARE OF PEOPLE WITH HYPERTENSION IN PRIMARY HEALTH CARE SETTINGS WITHIN THE BRAZILIAN UNIFIED HEALTH SYSTEM**
  **Coordenators:** Carlos Alberto Machado, Fernando Antonio de Almeida
  **Members:** Amaury Zatorre Amaral, José Augusto Soares Barreto-Filho
**15. FUTURE PROSPECTS**
  **Members:** Andréa Araujo Brandão, Audes Diógenes de Magalhães Feitosa, Cibele Isaac Saad Rodrigues, Weimar Kuns Sebba Barroso de Souza, Wilson Nadruz

**Table:** 

Brazilian Guidelines of Hypertension – 2025	
The report below lists declarations of interest as reported to the SBC by the experts during the period of the development of these statement, 2024/2025.	
Expert	Type of relationship with industry
Alexandre Jorge Gomes de Lucena	Nothing to be declared
Alvaro Avezum	Other relationships Funding of continuing medical education activities, including travel, accommodation and registration in conferences and courses, from the brazilian or international pharmaceutical, orthosis, prosthesis, equipment and implants industry: - EMS: ESC Congress; Biolab: ESC Congress.
Amaury Zatorre Amaral	Nothing to be declared
Ana Flavia Moura	Financial declaration A - Economically relevant payments of any kind made to (i) you, (ii) your spouse/partner or any other person living with you, (iii) any legal person in which any of these is either a direct or indirect controlling owner, business partner, shareholder or participant; any payments received for lectures, lessons, training instruction, compensation, fees paid for participation in advisory boards, investigative boards or other committees, etc. from the brazilian or international pharmaceutical, orthosis, prosthesis, equipment and implants industry: - AstraZeneca: consultancy.
Ana Lúcia Rego Fleury de Camargo	Nothing to be declared
Anderson da Costa Armstrong	Financial declaration A - Economically relevant payments of any kind made to (i) you, (ii) your spouse/partner or any other person living with you, (iii) any legal person in which any of these is either a direct or indirect controlling owner, business partner, shareholder or participant; any payments received for lectures, lessons, training instruction, compensation, fees paid for participation in advisory boards, investigative boards or other committees, etc. from the brazilian or international pharmaceutical, orthosis, prosthesis, equipment and implants industry: - Alnylam: amvuttra; Novo Nordisk: Rybelsus e wegovy. Other relationships Funding of continuing medical education activities, including travel, accommodation and registration in conferences and courses, from the brazilian or international pharmaceutical, orthosis, prosthesis, equipment and implants industry: - Alnylam: amyloidosis; Novo Nordisk: semaglutide. Any economically relevant equity interest in companies in the healthcare or education industry or in any companies competing with or supplying to SBC: - Medical work clinics: Cardiovasf and Cintilo. Employment relationship with the brazilian or international pharmaceutical, orthosis, prosthesis, equipment and implants industry, as well as any employment relationship with health insurance companies or medical audit companies (including part-time jobs) in the year to which your declaration refers: - Cooperative doctor at Unimed Vale do São Francisco.
Andréa Araujo Brandão	Financial declaration A - Economically relevant payments of any kind made to (i) you, (ii) your spouse/partner or any other person living with you, (iii) any legal person in which any of these is either a direct or indirect controlling owner, business partner, shareholder or participant; any payments received for lectures, lessons, training instruction, compensation, fees paid for participation in advisory boards, investigative boards or other committees, etc. from the brazilian or international pharmaceutical, orthosis, prosthesis, equipment and implants industry: - Biolab: Nebivolol; AstraZeneca: Metoprolol; Servier: Perindopril, Hypera; Mantecorp: Olmesartana e combinações; Torrent: Olmesartana e combinações; Daiichi Sankyo: Olmesartana e combinações. B - Research funding under your direct/personal responsibility (directed to the department or institution) from the brazilian or international pharmaceutical, orthosis, prosthesis, equipment and implants industry: - Daiichi Sankyo: Olmesartan; Servant: Elfie; Brainfarma: Olmesartan. Other relationships Funding of continuing medical education activities, including travel, accommodation and registration in conferences and courses, from the brazilian or international pharmaceutical, orthosis, prosthesis, equipment and implants industry: - Servier: Perindopril; Novo Nordisk: Semaglutide.
Andrea Pio de Abreu	Nothing to be declared
Andrei C. Sposito	Financial declaration A - Economically relevant payments of any kind made to (i) you, (ii) your spouse/partner or any other person living with you, (iii) any legal person in which any of these is either a direct or indirect controlling owner, business partner, shareholder or participant; any payments received for lectures, lessons, training instruction, compensation, fees paid for participation in advisory boards, investigative boards or other committees, etc. from the brazilian or international pharmaceutical, orthosis, prosthesis, equipment and implants industry: - Lilly, Novo Nordisk, Daiichi Sankyo. B - Research funding under your direct/personal responsibility (directed to the department or institution) from the brazilian or international pharmaceutical, orthosis, prosthesis, equipment and implants industry: - AstraZeneca. Other relationships Funding of continuing medical education activities, including travel, accommodation and registration in conferences and courses, from the brazilian or international pharmaceutical, orthosis, prosthesis, equipment and implants industry: - Daiichi Sankyo, Novo Nordisk.
Angela Maria Geraldo Pierin	Nothing to be declared
Annelise Machado Gomes de Paiva	Nothing to be declared
Antonio Gabriele Laurinavicius	Financial declaration A - Economically relevant payments of any kind made to (i) you, (ii) your spouse/partner or any other person living with you, (iii) any legal person in which any of these is either a direct or indirect controlling owner, business partner, shareholder or participant; any payments received for lectures, lessons, training instruction, compensation, fees paid for participation in advisory boards, investigative boards or other committees, etc. from the brazilian or international pharmaceutical, orthosis, prosthesis, equipment and implants industry: - AstraZeneca; EMS; Biolab; Servier; Libbs; Merck. Other relationships Funding of continuing medical education activities, including travel, accommodation and registration in conferences and courses, from the brazilian or international pharmaceutical, orthosis, prosthesis, equipment and implants industry: - Servier.
Audes Diógenes de Magalhães Feitosa	Financial declaration A - Economically relevant payments of any kind made to (i) you, (ii) your spouse/partner or any other person living with you, (iii) any legal person in which any of these is either a direct or indirect controlling owner, business partner, shareholder or participant; any payments received for lectures, lessons, training instruction, compensation, fees paid for participation in advisory boards, investigative boards or other committees, etc. from the brazilian or international pharmaceutical, orthosis, prosthesis, equipment and implants industry: - Omron: hypertension; Servier: hypertension; Biolab: hypertension; Brace: hypertension; Daichi Sankyo: hypertension; FQM: hypertension; Merck: hypertension. B - Research funding under your direct/personal responsibility (directed to the department or institution) from the brazilian or international pharmaceutical, orthosis, prosthesis, equipment and implants industry: - Servier: hypertension; Brace: hypertension; Daiichi Sankyo: hypertension; Angel: Lp(a), Lilly: Lp(a), Janssen: anticoagulant; Bayer: Finerinone. Other relationships Funding of continuing medical education activities, including travel, accommodation and registration in conferences and courses, from the brazilian or international pharmaceutical, orthosis, prosthesis, equipment and implants industry: - Servier: hypertension; FQM: hypertension; Novo Nordisk: diabetes and obesity; Sanofi: vaccines.
Carlos Alberto Machado	Nothing to be declared
Carlos Eduardo Poli-de-Figueiredo	Financial declaration A - Economically relevant payments of any kind made to (i) you, (ii) your spouse/partner or any other person living with you, (iii) any legal person in which any of these is either a direct or indirect controlling owner, business partner, shareholder or participant; any payments received for lectures, lessons, training instruction, compensation, fees paid for participation in advisory boards, investigative boards or other committees, etc. from the brazilian or international pharmaceutical, orthosis, prosthesis, equipment and implants industry: - AstraZeneca: SHUA and Chronic Kidney Disease; Organization of the MAYOPUCRS 2023 event and other Medical Society congresses with corporate support. B - Research funding under your direct/personal responsibility (directed to the department or institution) from the brazilian or international pharmaceutical, orthosis, prosthesis, equipment and implants industry: - Researcher at the PUCRS Clinical Research Center. No studies at this time. Other relationships Funding of continuing medical education activities, including travel, accommodation and registration in conferences and courses, from the brazilian or international pharmaceutical, orthosis, prosthesis, equipment and implants industry: - Medical office (Nefromonitoriza). Participation in studies funded by the Ministry of Health, CNPq and CAPES. Any economically relevant equity interest in companies in the healthcare or education industry or in any companies competing with or supplying to SBC: - Consultório médico (Nefromonitoriza). Participação em órgãos governamentais de regulação, ou de defesa de direitos na área de cardiologia: - FAPERGS Consultant.
Celso Amodeo	Financial declaration A - Economically relevant payments of any kind made to (i) you, (ii) your spouse/partner or any other person living with you, (iii) any legal person in which any of these is either a direct or indirect controlling owner, business partner, shareholder or participant; any payments received for lectures, lessons, training instruction, compensation, fees paid for participation in advisory boards, investigative boards or other committees, etc. from the brazilian or international pharmaceutical, orthosis, prosthesis, equipment and implants industry: - EMS: BRAs. Other relationships Funding of continuing medical education activities, including travel, accommodation and registration in conferences and courses, from the brazilian or international pharmaceutical, orthosis, prosthesis, equipment and implants industry: - EMS.
Cibele Isaac Saad Rodrigues	Nothing to be declared
Claudia Lucia de Moraes Forjaz	Nothing to be declared
Decio Mion Júnior	Nothing to be declared
Dilma do Socorro Moraes de Souza	Financial declaration A - Economically relevant payments of any kind made to (i) you, (ii) your spouse/partner or any other person living with you, (iii) any legal person in which any of these is either a direct or indirect controlling owner, business partner, shareholder or participant; any payments received for lectures, lessons, training instruction, compensation, fees paid for participation in advisory boards, investigative boards or other committees, etc. from the brazilian or international pharmaceutical, orthosis, prosthesis, equipment and implants industry: - Novartis Biociência.
Eduardo Costa Duarte Barbosa	Other relationships Funding of continuing medical education activities, including travel, accommodation and registration in conferences and courses, from the brazilian or international pharmaceutical, orthosis, prosthesis, equipment and implants industry: - Servier; Cardios; Mantecorp; Brace Pharma.
Elizabete Viana de Freitas	Nothing to be declared
Elizabeth Silaid Muxfeldt	Nothing to be declared
Emilton Lima Júnior	Financial declaration A - Economically relevant payments of any kind made to (i) you, (ii) your spouse/partner or any other person living with you, (iii) any legal person in which any of these is either a direct or indirect controlling owner, business partner, shareholder or participant; any payments received for lectures, lessons, training instruction, compensation, fees paid for participation in advisory boards, investigative boards or other committees, etc. from the brazilian or international pharmaceutical, orthosis, prosthesis, equipment and implants industry: - Bayer: kidney disease; Biolab: hypertension and dyslipidemia; Chiesi: COPD; Daiichi Sankyo: dyslipidemia; Novartis: dyslipidemia; Novo Nordisk: cardiometabolism; Servier: hypertension and cardiometabolism. Other relationships Funding of continuing medical education activities, including travel, accommodation and registration in conferences and courses, from the brazilian or international pharmaceutical, orthosis, prosthesis, equipment and implants industry: - Servier: hypertension.
Erika Maria Gonçalves Campana	Financial declaration A - Economically relevant payments of any kind made to (i) you, (ii) your spouse/partner or any other person living with you, (iii) any legal person in which any of these is either a direct or indirect controlling owner, business partner, shareholder or participant; any payments received for lectures, lessons, training instruction, compensation, fees paid for participation in advisory boards, investigative boards or other committees, etc. from the brazilian or international pharmaceutical, orthosis, prosthesis, equipment and implants industry: - Servier, Brace Pharma, Biolab, Momenta. Other relationships Funding of continuing medical education activities, including travel, accommodation and registration in conferences and courses, from the brazilian or international pharmaceutical, orthosis, prosthesis, equipment and implants industry: - Servier, Biolab: hypertension.
Fábio Argenta	Financial declaration A - Economically relevant payments of any kind made to (i) you, (ii) your spouse/partner or any other person living with you, (iii) any legal person in which any of these is either a direct or indirect controlling owner, business partner, shareholder or participant; any payments received for lectures, lessons, training instruction, compensation, fees paid for participation in advisory boards, investigative boards or other committees, etc. from the brazilian or international pharmaceutical, orthosis, prosthesis, equipment and implants industry: - Novartis: Sybrava; Boehringer: Jardiance; AstraZeneca: Forxiga; Servier: Triplixam; Daiichi Sankyo: Benicar; GSK: vacinas. Other relationships Funding of continuing medical education activities, including travel, accommodation and registration in conferences and courses, from the brazilian or international pharmaceutical, orthosis, prosthesis, equipment and implants industry: - Novo Nordisk: Wegovy; Torrent: Rosucor.
Fernanda Marciano Consolim Colombo	Financial declaration A - Economically relevant payments of any kind made to (i) you, (ii) your spouse/partner or any other person living with you, (iii) any legal person in which any of these is either a direct or indirect controlling owner, business partner, shareholder or participant; any payments received for lectures, lessons, training instruction, compensation, fees paid for participation in advisory boards, investigative boards or other committees, etc. from the brazilian or international pharmaceutical, orthosis, prosthesis, equipment and implants industry: - Daiichi Sankyo; Merck; Servier; AstraZeneca. Other relationships Funding of continuing medical education activities, including travel, accommodation and registration in conferences and courses, from the brazilian or international pharmaceutical, orthosis, prosthesis, equipment and implants industry: - Daiichi Sankyo; Servier.
Fernanda Salomão Gorayeb Polacchini	Financial declaration A - Economically relevant payments of any kind made to (i) you, (ii) your spouse/partner or any other person living with you, (iii) any legal person in which any of these is either a direct or indirect controlling owner, business partner, shareholder or participant; any payments received for lectures, lessons, training instruction, compensation, fees paid for participation in advisory boards, investigative boards or other committees, etc. from the brazilian or international pharmaceutical, orthosis, prosthesis, equipment and implants industry: - AstraZeneca: Lokelma e Forxiga.
Fernando Antonio de Almeida	Nothing to be declared
Fernando Nobre	Financial declaration A - Economically relevant payments of any kind made to (i) you, (ii) your spouse/partner or any other person living with you, (iii) any legal person in which any of these is either a direct or indirect controlling owner, business partner, shareholder or participant; any payments received for lectures, lessons, training instruction, compensation, fees paid for participation in advisory boards, investigative boards or other committees, etc. from the brazilian or international pharmaceutical, orthosis, prosthesis, equipment and implants industry: - EMS; Servier; Novartis; Daiichi Sankyo; Biolab. Other relationships Funding of continuing medical education activities, including travel, accommodation and registration in conferences and courses, from the brazilian or international pharmaceutical, orthosis, prosthesis, equipment and implants industry: - AstraZeneca.
Flavio Antonio de Oliveira Borelli	Nothing to be declared
Frida Liane Plavnik	Financial declaration A - Economically relevant payments of any kind made to (i) you, (ii) your spouse/partner or any other person living with you, (iii) any legal person in which any of these is either a direct or indirect controlling owner, business partner, shareholder or participant; any payments received for lectures, lessons, training instruction, compensation, fees paid for participation in advisory boards, investigative boards or other committees, etc. from the brazilian or international pharmaceutical, orthosis, prosthesis, equipment and implants industry: - EMS; Servier; Novartis; Daiichi Sankyo; Biolab. Other relationships Funding of continuing medical education activities, including travel, accommodation and registration in conferences and courses, from the brazilian or international pharmaceutical, orthosis, prosthesis, equipment and implants industry: - Libbs: candesartan.
Giovanio Vieira da Silva	Nothing to be declared
Grazia Maria Guerra	Nothing to be declared
Heno Ferreira Lopes	Nothing to be declared
João Roberto Gemelli	Financial declaration A - Economically relevant payments of any kind made to (i) you, (ii) your spouse/partner or any other person living with you, (iii) any legal person in which any of these is either a direct or indirect controlling owner, business partner, shareholder or participant; any payments received for lectures, lessons, training instruction, compensation, fees paid for participation in advisory boards, investigative boards or other committees, etc. from the brazilian or international pharmaceutical, orthosis, prosthesis, equipment and implants industry: - AstraZeneca: Forxiga, Breztri; Novo Nordisk: Rybelsus; Servier: Trilpixam e Vastarel. Other relationships Funding of continuing medical education activities, including travel, accommodation and registration in conferences and courses, from the brazilian or international pharmaceutical, orthosis, prosthesis, equipment and implants industry: - Astrazeneca: Forxiga, Breztri; Novo Nordisk: Rybelsus; Servier: Trimplixam, Vastarel.
José Andrade Moura-Neto	Financial declaration A - Economically relevant payments of any kind made to (i) you, (ii) your spouse/partner or any other person living with you, (iii) any legal person in which any of these is either a direct or indirect controlling owner, business partner, shareholder or participant; any payments received for lectures, lessons, training instruction, compensation, fees paid for participation in advisory boards, investigative boards or other committees, etc. from the brazilian or international pharmaceutical, orthosis, prosthesis, equipment and implants industry: - AstraZeneca.
José Augusto Soares Barreto-Filho	Financial declaration A - Economically relevant payments of any kind made to (i) you, (ii) your spouse/partner or any other person living with you, (iii) any legal person in which any of these is either a direct or indirect controlling owner, business partner, shareholder or participant; any payments received for lectures, lessons, training instruction, compensation, fees paid for participation in advisory boards, investigative boards or other committees, etc. from the brazilian or international pharmaceutical, orthosis, prosthesis, equipment and implants industry: - Servier: class on cardiovascular risk assessment.
José Fernando Vilela-Martin	Nothing to be declared
Juan Carlos Yugar Toledo	Nothing to be declared
Leda Aparecida Daud Lotaif	Financial declaration A - Economically relevant payments of any kind made to (i) you, (ii) your spouse/partner or any other person living with you, (iii) any legal person in which any of these is either a direct or indirect controlling owner, business partner, shareholder or participant; any payments received for lectures, lessons, training instruction, compensation, fees paid for participation in advisory boards, investigative boards or other committees, etc. from the brazilian or international pharmaceutical, orthosis, prosthesis, equipment and implants industry: - AstraZeneca: Forxiga, Lojelma, Fresenius Kabi: Ketosteril.
Lílian Soares da Costa	Nothing to be declared
Lucelia Batista Neves Cunha Magalhães	Nothing to be declared
Luciano Ferreira Drager	Financial declaration A - Economically relevant payments of any kind made to (i) you, (ii) your spouse/partner or any other person living with you, (iii) any legal person in which any of these is either a direct or indirect controlling owner, business partner, shareholder or participant; any payments received for lectures, lessons, training instruction, compensation, fees paid for participation in advisory boards, investigative boards or other committees, etc. from the brazilian or international pharmaceutical, orthosis, prosthesis, equipment and implants industry: - Aché, AstraZeneca, Biolab, Daiichi Sankyo, Lilly and Novo Nordisk: lectures focused on the diagnosis and treatment of Arterial Hypertension and comorbidities. Other relationships Funding of continuing medical education activities, including travel, accommodation and registration in conferences and courses, from the brazilian or international pharmaceutical, orthosis, prosthesis, equipment and implants industry: - AstraZeneca, Lilly and Novo Nordisk.
Luis Cuadrado Martin	Other relationships Funding of continuing medical education activities, including travel, accommodation and registration in conferences and courses, from the brazilian or international pharmaceutical, orthosis, prosthesis, equipment and implants industry: - Servier: antihypertensives.
Luiz Aparecido Bortolotto	Financial declaration A - Economically relevant payments of any kind made to (i) you, (ii) your spouse/partner or any other person living with you, (iii) any legal person in which any of these is either a direct or indirect controlling owner, business partner, shareholder or participant; any payments received for lectures, lessons, training instruction, compensation, fees paid for participation in advisory boards, investigative boards or other committees, etc. from the brazilian or international pharmaceutical, orthosis, prosthesis, equipment and implants industry: - Servier: Triplixam; GSK: vacina. Other relationships Funding of continuing medical education activities, including travel, accommodation and registration in conferences and courses, from the brazilian or international pharmaceutical, orthosis, prosthesis, equipment and implants industry: - Servier: Triplixam.
Luiz César Nazário Scala	Nothing to be declared
Márcia Regina Simas Torres Klein	Nothing to be declared
Marco Antonio Mota-Gomes	Financial declaration A - Economically relevant payments of any kind made to (i) you, (ii) your spouse/partner or any other person living with you, (iii) any legal person in which any of these is either a direct or indirect controlling owner, business partner, shareholder or participant; any payments received for lectures, lessons, training instruction, compensation, fees paid for participation in advisory boards, investigative boards or other committees, etc. from the brazilian or international pharmaceutical, orthosis, prosthesis, equipment and implants industry: - Omron: Equipamentos; Biolab: Press Plus; Daiichi Sankyo: Benicar Triplo; AstraZeneca: Forxiga; Ache: Edistride. B - Research funding under your direct/personal responsibility (directed to the department or institution) from the brazilian or international pharmaceutical, orthosis, prosthesis, equipment and implants industry: - Novartis: Inclisiran; Libbs: Triple Combination; Servier: Quadruple Combination.
Marcus Vinicius Bolivar Malachias	Financial declaration A - Economically relevant payments of any kind made to (i) you, (ii) your spouse/partner or any other person living with you, (iii) any legal person in which any of these is either a direct or indirect controlling owner, business partner, shareholder or participant; any payments received for lectures, lessons, training instruction, compensation, fees paid for participation in advisory boards, investigative boards or other committees, etc. from the brazilian or international pharmaceutical, orthosis, prosthesis, equipment and implants industry: - Dapagliflozin/AstraZeneca: heart failure, diabetes; Finerenone/Bayer: heart failure, diabetes; Empagliflozin/Boehringer-Ingelheim: heart failure, diabetes; Olmesartan/Daiichi-Sankyo: hypertension; Telmisartan/EMS: hypertension; Arexvy/GSK: vaccines; Candesartan/Libbs: hypertension, heart failure; Levanlodipine/Mantecorp: hypertension; Bisoprolol/Merck: hypertension, heart failure; Inclisiran/Novartis: heart failure, dyslipidemia; Semaglutide/Novo Nordisk: diabetes; Elecsys/Roche: biomarkers, heart failure, diabetes; Eplerenone/Viatris: heart failure, hypertension.
Maria Eliane Campos Magalhães	Financial declaration A - Economically relevant payments of any kind made to (i) you, (ii) your spouse/partner or any other person living with you, (iii) any legal person in which any of these is either a direct or indirect controlling owner, business partner, shareholder or participant; any payments received for lectures, lessons, training instruction, compensation, fees paid for participation in advisory boards, investigative boards or other committees, etc. from the brazilian or international pharmaceutical, orthosis, prosthesis, equipment and implants industry: - AstraZeneca: Forxiga, Breztri, Lokelma.
Maria Eliete Pinheiro	Nothing to be declared
Maria Emília Figueiredo Teixeira	Nothing to be declared
Mario Fritsch Toros Neves	Nothing to be declared
Nelson Dinamarco	Nothing to be declared
Osni Moreira Filho	Financial declaration A - Economically relevant payments of any kind made to (i) you, (ii) your spouse/partner or any other person living with you, (iii) any legal person in which any of these is either a direct or indirect controlling owner, business partner, shareholder or participant; any payments received for lectures, lessons, training instruction, compensation, fees paid for participation in advisory boards, investigative boards or other committees, etc. from the brazilian or international pharmaceutical, orthosis, prosthesis, equipment and implants industry: - Servier: arterial hypertension. Other relationships Funding of continuing medical education activities, including travel, accommodation and registration in conferences and courses, from the brazilian or international pharmaceutical, orthosis, prosthesis, equipment and implants industry: - Torrent: arterial hypertension.
Oswaldo Passarelli Junior	Nothing to be declared
Otavio Rizzi Coelho	Financial declaration A - Economically relevant payments of any kind made to (i) you, (ii) your spouse/partner or any other person living with you, (iii) any legal person in which any of these is either a direct or indirect controlling owner, business partner, shareholder or participant; any payments received for lectures, lessons, training instruction, compensation, fees paid for participation in advisory boards, investigative boards or other committees, etc. from the brazilian or international pharmaceutical, orthosis, prosthesis, equipment and implants industry: - Bayer: Finerinone; EMS: Rivaroxabana; AstraZeneca: Dapaglifozina; Libbs: Ticagrelor. Other relationships Funding of continuing medical education activities, including travel, accommodation and registration in conferences and courses, from the brazilian or international pharmaceutical, orthosis, prosthesis, equipment and implants industry: - Bayer; Novo Nordisk; AstraZeneca.
Paulo César Brandão Veiga Jardim	Nothing to be declared
Renault Mattos Ribeiro Júnior	Other relationships Funding of continuing medical education activities, including travel, accommodation and registration in conferences and courses, from the brazilian or international pharmaceutical, orthosis, prosthesis, equipment and implants industry: - Novo Nordisk, GSK.
Roberto Dischinger Miranda	Financial declaration A - Economically relevant payments of any kind made to (i) you, (ii) your spouse/partner or any other person living with you, (iii) any legal person in which any of these is either a direct or indirect controlling owner, business partner, shareholder or participant; any payments received for lectures, lessons, training instruction, compensation, fees paid for participation in advisory boards, investigative boards or other committees, etc. from the brazilian or international pharmaceutical, orthosis, prosthesis, equipment and implants industry: - Aché: Enoxaparin; Daiichi: Benicar; Nutricia: Nutridrink; EMS: Xaquilis; Novo Nordisk: Wegovy; Pfizer: Prevenar and Arexvy vaccines. Any economically relevant equity interest in companies in the healthcare or education industry or in any companies competing with or supplying to SBC: - Founding partner of the Longevitá Institute (health sector).
Rodrigo Bezerra	Nothing to be declared
Rodrigo Pinto Pedrosa	Nothing to be declared
Rogério Andrade Mulinari	Financial declaration B - Research funding under your direct/personal responsibility (directed to the department or institution) from the brazilian or international pharmaceutical, orthosis, prosthesis, equipment and implants industry: - Tricida, Inc: TRC101 in chronic kidney disease.
Rogério Baumgratz de Paula	Nothing to be declared
Rogério Toshiro Passos Okawa	Financial declaration A - Economically relevant payments of any kind made to (i) you, (ii) your spouse/partner or any other person living with you, (iii) any legal person in which any of these is either a direct or indirect controlling owner, business partner, shareholder or participant; any payments received for lectures, lessons, training instruction, compensation, fees paid for participation in advisory boards, investigative boards or other committees, etc. from the brazilian or international pharmaceutical, orthosis, prosthesis, equipment and implants industry: - EMS: hypertension. Other relationships Funding of continuing medical education activities, including travel, accommodation and registration in conferences and courses, from the brazilian or international pharmaceutical, orthosis, prosthesis, equipment and implants industry: - Novo Nordisk: obesity.
Rui Manuel dos Santos Povoa	Nothing to be declared
Sandra Lia do Amaral Cardoso	Nothing to be declared
Sayuri Inuzuka	Other relationships Funding of continuing medical education activities, including travel, accommodation and registration in conferences and courses, from the brazilian or international pharmaceutical, orthosis, prosthesis, equipment and implants industry: - Servier.
Sebastião Rodrigues Ferreira-Filho	Nothing to be declared
Sergio Emanuel Kaiser	Financial declaration A - Economically relevant payments of any kind made to (i) you, (ii) your spouse/partner or any other person living with you, (iii) any legal person in which any of these is either a direct or indirect controlling owner, business partner, shareholder or participant; any payments received for lectures, lessons, training instruction, compensation, fees paid for participation in advisory boards, investigative boards or other committees, etc. from the brazilian or international pharmaceutical, orthosis, prosthesis, equipment and implants industry: - Bayer: Firialta; Novartis: Sybrava; Libbs: Naprix; Daiichi Sankyo: Nustendi and Benicar; Biolab: Repatha and Livalo; Astrazeneca: Selozok and Forxiga; Novo Nordisk: Ozempic and Wegovy: Boehringer Ingelheim: Jardiance. Other relationships Funding of continuing medical education activities, including travel, accommodation and registration in conferences and courses, from the brazilian or international pharmaceutical, orthosis, prosthesis, equipment and implants industry: - Bayer: Vericiguat; Novo Nordisk: Wegovy; Daiichi Sankyo: Nustendi; Benicar: Lixiana; Novartis: Sybrava.
Sheyla Cristina Tonheiro Ferro da Silva	Other relationships Funding of continuing medical education activities, including travel, accommodation and registration in conferences and courses, from the brazilian or international pharmaceutical, orthosis, prosthesis, equipment and implants industry: - AstraZeneca: Forxiga, Lokelma; Novartis: Entresto, Sybrava; Lilly: Jardiance, Mounjaro; Novo Nordisk: Rybelsus, Wegovy.
Tânia Plens Shecaira	Nothing to be declared
Vera Hermina Kalika Koch	Nothing to be declared
Weimar Kunz Sebba Barroso	Financial declaration A - Economically relevant payments of any kind made to (i) you, (ii) your spouse/partner or any other person living with you, (iii) any legal person in which any of these is either a direct or indirect controlling owner, business partner, shareholder or participant; any payments received for lectures, lessons, training instruction, compensation, fees paid for participation in advisory boards, investigative boards or other committees, etc. from the brazilian or international pharmaceutical, orthosis, prosthesis, equipment and implants industry: - EMS; Brace Pharma; Servier; Biolab; Omron; Cardios. B - Financiamento de pesquisas sob sua responsabilidade direta/pessoal (direcionado ao departamento ou instituição) provenientes da indústria farmacêutica, de órteses, próteses, equipamentos e implantes, brasileiras ou estrangeiras: - Ministry of Health; PROADI SUS; Novartis: Entresto. C - Personal research funding paid by the brazilian or international pharmaceutical, orthosis, prosthesis, equipment and implants industry: - EMS: RITMO Study; Brace Pharma: BRACE 23 Study. Other relationships Funding of continuing medical education activities, including travel, accommodation and registration in conferences and courses, from the brazilian or international pharmaceutical, orthosis, prosthesis, equipment and implants industry: - Servier; European Congress on Hypertension.
Wilson Nadruz	Nothing to be declared

## Table of contents

**1. Introduction** 22
  **1.1. Key Highlights** 23
**2. Epidemiology, Definition, and Primary Prevention** 28
  **2.1. Definition, Epidemiology, and Primary Prevention** 28
    **2.1.1. Definition of Hypertension** 28
    **2.1.2. Epidemiological Data and Impact of Hypertension** 28
  **2.3. Risk Factors for Hypertension** 28
    **2.3.1. Genetics** 28
    **2.3.2. Age and Sex** 28
    **2.3.3. Ethnicity, Urbanization, and Socioeconomic Factors** 28
    **2.3.4. Spirituality and Psychosocial Factors** 29
    **2.3.5. Overweight/Obesity and Physical Inactivity** 29
    **2.3.6. Sodium and Potassium Intake** 29
    **2.3.7. Alcohol** 30
    **2.3.8. COVID-19** 30
    **2.3.9. Medications, Illicit Drugs, and Anabolic Steroid Use** 30
    **2.3.10. Sleep Disorders** 30
    **2.3.11. Smoking** 30
    **2.3.12. Environmental Factors** 30
  **2.4. Primary Prevention** 30
**3. Blood Pressure Measurement, Diagnosis, and Classification** 32
  **3.1. Introduction** 32
  **3.2. Office Blood Pressure Measurement** 32
    **3.2.1. Blood Pressure Measurement Techniques** 32
    **3.2.2. Special Populations** 32
    **3.2.3. Classification** 32
  **3.3. Out-of-Office Blood Pressure Measurement** 34
    **3.3.1. Indications for Ambulatory or Home Blood Pressure Monitoring** 34
  **3.4. White-Coat Effect and Masked Effect** 34
  **3.5. White Coat Hypertension and Masked Hypertension** 34
  **3.6. Uncontrolled Masked Hypertension and Uncontrolled White-Coat Hypertension** 35
  **3.7. Recommendations for Diagnosis and Follow-Up** 35
    **3.7.1. Self-Measured Blood Pressure** 36
    **3.7.2. Exercise Stress Test** 36
  **3.8. Central Blood Pressure, Pulse Wave Velocity, and Augmentation Index** 36
    **3.9. New Technologies for Blood Pressure Measurement** 37
**4. Clinical and Complementary Assessment and Cardiovascular Risk Stratification** 38
  **4.1. Estratificação de Risco Adicional (Condições Associadas)** 39
  **4.2. Clinical and Complementary Assessment** 39
    **4.2.1. Medical History** 39
    **4.2.2. Physical Examination** 39
    **4.2.3. Additional Tests** 39
  **4.4. Hypertension-Mediated Organ Damage** 39
  **4.5. Chronic Kidney Disease Assessment** 39
  **4.6. Assessment of Secondary Causes** 41
  **4.7. Cardiovascular Risk Calculation** 41
  **4.8. Additional Considerations and Challenges in Cardiovascular Risk Assessment in Hypertension** 43
**5. Treatment Initiation and Therapeutic Targets** 46
  **5.1. Treatment Initiation** 46
  **5.2. Blood Pressure Targets** 47
  **5.3. Blood Pressure Targets in Ambulatory and Home Blood Pressure Monitoring** 47
  **5.4. The J-Curve of Blood Pressure** 47
  **5.5. Intensive Blood Pressure Reductions and Risk of Adverse Events** 47
  **5.6. Considerations in Isolated Systolic Hypertension** 49
**6. Nonpharmacological Measures** 50
  **6.1. Smoking Cessation** 50
  **6.2. Weight Loss** 50
  **6.3. Dietary Modifications** 50
    **6.3.1. Restricted Sodium Intake** 50
    **6.3.2. Increased Dietary Potassium Intake** 51
    **6.3.3. Reduced Alcohol Consumption** 51
    **6.3.4. Adopting a Healthy Eating Pattern** 51
    **6.3.5. Other Dietary Interventions** 52
  **6.4. Physical Activity and Exercise** 52
  **6.5. Approach to Psycho-Emotional Aspects** 52
    **6.5.1. Stress Management Techniques** 52
    **6.5.2. Slow Breathing** 52
    **6.5.3. Spirituality and Religiosity** 53
  **6.6. Multidisciplinary Team in the Adoption of Nonpharmacological Measures** 53
  **6.7. Final Considerations on Nonpharmacological Measures** 53
**7. Pharmacological Treatment** 55
  **7.1. Treatment Targets and Rationale for Medication Use** 55
    **7.1.1. Heart Failure** 55
    **7.1.2. Stroke** 55
    **7.1.3. Coronary Artery Disease** 56
    **7.1.4. Chronic Kidney Disease** 56
    **7.1.5. Cardiovascular Death** 56
  **7.2. Pharmacological Treatment** 56
  **7.3. General Principles of Pharmacological Treatment** 57
  **7.4. Classes of Medications Used in the Treatment of Hypertensiont** 57
  **7.5. Medications Indicated for other Conditions that Affect Blood Pressure and Cardiovascular Events but are Not yet Established in Routine Hypertension Treatment** 57
    **7.5.1. Sacubitril/Valsartan** 57
    **7.5.2. Sodium-Glucose Cotransporter 2 Inhibitors** 58
    **7.5.3. Glucagon-Like Peptide-1 Receptor Agonists and New Selective Nonsteroidal Mineralocorticoid Receptor Antagonists** 61
  **7.6. Treatment Strategies** 61
    **7.6.1. Monotherapy** 61
    **7.6.2. Combination Therapy** 62
**8. Hypertension and Associated Clinical Conditions: Coronary Artery Disease, Chronic Kidney Disease, Diabetes, Obesity, COVID-19, Post-Stroke, and Heart Failure** 65
  **8.1. Hypertension and Coronary Artery Disease** 65
    **8.1.1. Blood Pressure Targets in Coronary Artery Disease** 65
    **8.1.2. Treatment of Hypertension and Coronary Artery Disease** 63
  **8.2. Hypertension in Chronic Kidney Disease in Patients on Conservative Treatment and Dialysis** 66
    **8.2.1. Blood Pressure Target for Chronic Kidney Disease Under Conservative Treatment** 66
    **8.2.2. Blood Pressure Target for Patients with Chronic Kidney Disease on Dialysis** 67
    **8.2.3. Treatment of Hypertension in Chronic Kidney Disease on Conservative Management** 67
    **8.2.4. Treatment of Hypertension in Patients with Chronic Kidney Disease on Dialysis** 67
  **8.3. Hypertension and Diabetes** 68
    **8.3.1. Blood Pressure Targets in Patients with Diabetes** 69
    **8.3.2. Treatment of Hypertension in Patients with Diabetes** 69
  **8.4. Hypertension and Obesity** 70
    **8.4.1. Blood Pressure Target in Patients with Obesity** 70
    **8.4.2. Treatment of Hypertension in Obesity** 70
  **8.5. Hypertension and COVID-19** 71
  **8.6. Hypertension, Stroke, and Cognitive Impairment** 72
    **8.6.1. Blood Pressure Target in Chronic Phase Post-stroke Hypertension** 72
    **8.6.2. Treatment of Hypertension in the Chronic Phase after Stroke** 72
  **8.7. Hypertension and Heart Failure with Preserved and Reduced Ejection Fraction** 73
**9. Hypertension in Older Adults, Children, and Adolescents** 74
  **9.1. Hypertension in Older Adults** 74
    **9.1.1. Introduction** 74
    **9.1.2. Pathophysiological Mechanisms** 75
    **9.1.3. Diagnosis and Therapeutic Decision-Making** 75
      **9.1.3.1. Diagnosis** 75
      **9.1.3.2. Assessment of Functional Status and Frailty** 75
      **9.1.3.3. Assessment of Cognitive Function** 75
      **9.1.3.4. Therapeutic Decision-Making** 76
    **9.1.4. Blood Pressure Targets and Treatment** 76
      **9.1.4.1. Blood Pressure Targets in Older Adults** 77
      **9.1.4.2. Non-Pharmacological Treatment** 77
      **9.1.4.3. Pharmacological Treatment** 77
      **9.1.4.4. Polypharmacy and Adherence** 77
      **9.1.4.5. Deintensification and Deprescribing** 77
  **9.2. Hypertension in Children and Adolescents** 79
**10. Hypertension in Women** 88
  **10.1. Hypertension in Young Women and During Menopause** 88
    **10.1.1. Prevalence** 88
    **10.1.2. Factors Associated with Hypertension in Women** 88
      **10.1.2.1. Young Women and Oral Contraceptives** 88
      **10.1.2.2. Hypertension, Sexual Function, and Fertility in Women** 88
      **10.1.2.3. Menopause** 88
      **10.1.2.4. Polycystic Ovary Syndrome** 89
    **10.1.3. Target-Organ Damage and Comorbidities in Women** 89
    **10.1.4. Treatment of Hypertension in Women** 89
  **10.2. Hypertension in Pregnancy** 90
    **10.2.1. Classification** 90
    **10.2.2. Prediction and Prophylaxis of Preeclampsia** 90
    **10.2.3. Therapeutic approach to hypertension in pregnancy** 90
    **10.2.4. Postpartum Hypertension** 92
    **10.2.5. Future Cardiovascular Risk** 92
**11. Hypertensive Crisis** 94
  **11.1. Definition and Classification** 94
  **11.2. Classification** 94
  **11.3. Epidemiological and prognostic aspects** 94
    **11.3.1. Epidemiology** 94
    **11.3.2. Prognosis** 95
  **11.4. Complementary Clinical and Laboratory Investigation** 95
  **11.5. Overall management of hypertensive crisis** 95
  **11.6. Hypertensive Emergencies in Special Situations** 98
    **11.6.1. Hypertensive Encephalopathy** 96
    **11.6.2. Stroke** 99
      **11.6.2.1. Ischemic Stroke** 99
      **11.6.2.2. Hemorrhagic Stroke** 99
    **11.6.3. Acute Coronary Syndromes** 99
    **11.6.4. Acute Pulmonary Edema** 99
    **11.6.5. Acute Aortic Dissection** 99
    **11.6.6. Adrenergic Crisis** 99
    **11.6.7. Accelerated-Malignant Hypertension** 99
    **11.6.8. Hypertension with Multi-Organ Damage** 100
    **11.6.9. Hypertension in Pregnancy: Preeclampsia and Eclampsia** 100
**12. Resistant and Refractory Hypertension** 101
  **12.1. Definition and Classification** 101
  **12.2. Epidemiology of Resistant and Refractory Hypertension** 102
  **12.3. Pathophysiology** 102
  **12.4. Diagnostic Evaluation** 102
    **12.4.1. Inadequate Blood Pressure Measurement Technique** 102
    **12.4.2. White-Coat Effect** 104
    **12.4.3. Therapeutic Inertia** 104
    **12.4.4. Medication Nonadherence** 104
  **12.5. Clinical Approach** 104
  **12.6. Treatment** 104
    **12.6.1. Non-Pharmacological Treatment** 104
    **12.6.2. Pharmacological Treatment** 104
    **12.6.3. Perspectives** 105
    **12.6.4. Renal Denervation** 107
**13. Adherence to Antihypertensive Treatment** 108
  **13.1. Measuring Adherence** 108
  **13.2. Evidence** 108
  **13.3. Strategies** 109
  **13.4. Recommendations** 109
**14. Best Practices in the Care of People With Hypertension in Primary Health Care Settings Within the Brazilian Unified Health System** 109
  **14.1. The Brazilian Unified Health System and its Importance to Hypertension Control** 109
    **14.1.1. Primary Health Care** 109
    **14.1.2. Specialist Care** 112
  **14.2. Active Case-Finding of Hypertension** 112
  **14.3. Blood Pressure Measurement Protocol: Necessary Precautions** 113
  **14.4. Diagnosis of Hypertension** 113
  **14.5. Clinical and Complementary Assessment of the Patient with Hypertension** 114
  **14.6. Risk Stratification after Clinical Assessment and Workup** 114
  **14.7. Blood Pressure Targets** 116
  **14.8. Out-of-Office/Non-Healthcare Setting Blood Pressure Measurement** 117
  **14.9. Cornerstones of Management for Individuals with Hypertension** 117
  **14.10. Pharmacotherapy** 118
  **14.11. Clinical Conditions that Justify Specialist Referral** 123
  **14.12. Counter-Referral** 124
  **14.13. Telemedicine in the Brazilian Unified Health System Care Pathway for Hypertension** 124
**15. Future Prospects** 125
  **15.1. The Exposome and Hypertension** 125
  **15.2. Wearables and Telemonitoring in Hypertension** 126
  **15.3. Hypertension and Intracranial Compliance** 127
  **15.4. Ultra-low-Dose Triple and Quadruple Antihypertensive Drug Combinations** 127
  **15.5. Novel Drugs for the Treatment of Hypertension** 127
**Acknowledgments** 127
**References** 130

## 1. Introduction

The Brazilian Guidelines of Hypertension – 2025 represent a collaborative effort by the Brazilian Society of Cardiology (SBC), the Brazilian Society of Nephrology (SBN), and the Brazilian Society of Hypertension (SBH).

This document compiles the most up-to-date, evidence-based recommendations for the management of hypertension. It addresses all aspects of care, including diagnosis, monitoring, and treatment across different patient subgroups, associated clinical conditions, and complex scenarios such as hypertensive emergencies (HEs) and cases of resistant or refractory hypertension (Central Illustration).

A major challenge in developing these guidelines was ensuring strict adherence to the Standards for Developing Guidelines, Recommendations, and Position Statements set forth by the SBC. These standards require the use of the Grading of Recommendations Assessment, Development and Evaluation (GRADE) methodology to classify the strength of recommendation and certainty of evidence. We are especially grateful to methodologist and cardiologist Dr. Leonardo Luna for his essential insights, support, and guidance throughout this process.

A noteworthy innovation in the 2025 edition is the inclusion of a dedicated chapter on hypertension management within the Brazilian Unified Health System (SUS). This is a crucial addition, as approximately 75% of Brazilian patients with hypertension receive treatment through the SUS, particularly within the scope of primary health care services.

The development of a document as comprehensive as the Brazilian Guidelines of Hypertension – 2025, at 150 pages, was only possible thanks to the active participation of numerous physicians from various specialties and dedicated health care professionals. Their expertise and unwavering commitment to the care of patients with hypertension were instrumental, and we extend our deepest gratitude for their valuable contributions.

In this publication, we will highlight approximately 30% of the recommendations from the Brazilian Guidelines of Hypertension – 2025, those deemed most relevant or representing significant updates compared to the previous edition.

We hope this content proves valuable and encourage you to share it widely. Together, we can continue to improve the quality of care for patients with hypertension across Brazil.

**Andréa Araujo Brandão**

General Coordinator

**Wilson Nadruz**

Brazilian Society of Cardiology

**Cibele Isaac Saad Rodrigues**

Brazilian Society of Nephrology

**Luiz Aparecido Bortolotto**

Brazilian Society of Hypertension

### 1.1. Key Highlights

**Chapter 3:** Blood pressure measurement, diagnosis, and classification

Recommendations for Blood Pressure Measurement, Diagnosis, and Classification	Strength of recommendation	Certainty of evidence
Prehypertension should be classified as office SBP between 120-139 mm Hg or office DBP between 80-89 mm Hg to identify individuals at-risk early and encourage proactive, non-pharmacological measures to prevent progression to hypertension.	STRONG	MODERATE
Hypertension should be diagnosed when office BP is ≥ 140 and/or 90 mm Hg on two separate occasions, and classified into stages 1, 2, or 3 according to the highest SBP or DBP value.	STRONG	MODERATE
The use of automated upper-arm devices is recommended to reduce measurement errors and facilitate BP monitoring.	STRONG	MODERATE
The use of ABPM or HBPM is recommended to confirm the diagnosis of hypertension and monitor treatment.	STRONG	HIGH

ABPM: ambulatory blood pressure monitoring; BP: blood pressure; DBP: diastolic blood pressure; HBPM: home blood pressure monitoring; SBP: systolic blood pressure.

**Chapter 4:** Clinical and complementary assessment and cardiovascular risk stratification

Recommendations for clinical and complementary assessment, and cardiovascular risk stratification	Strength of recommendation	Certainty of evidence
CV risk factors and HMOD should ideally be assessed in all patients at the time of hypertension diagnosis and re-evaluated at least annually, with the choice of methods depending on available resources.	STRONG	LOW
It is recommended to assess CV risk using the PREVENT score.	STRONG	HIGH
Stratification of CV risk in individuals with prehypertension is recommended to guide the initiation of antihypertensive therapy and optimize control of CV risk factors.	STRONG	HIGH
The stratification of CV risk in patients with hypertension is recommended to allow for a more accurate and individualized approach to pharmacological therapy and to set tailored goals for CV risk factor control.	STRONG	HIGH

CV: cardiovascular; PREVENT: Predicting Risk of Cardiovascular Disease Events; HMOD: hypertension-mediated organ damage.

**Chapter 5:** Treatment initiation and therapeutic targets

Recommendations for initiation of antihypertensive treatment	Strength of recommendation	Certainty of evidence
NPMs are recommended for all individuals with BP ≥ 120/80 mm Hg.	STRONG	HIGH
Pharmacological treatment is recommended after 3 months of NPMs in individuals with BP 130-139/80-89 mm Hg and high CV risk.	STRONG	HIGH
Initiation of pharmacological therapy is recommended for individuals with BP ≥ 140/90 mm Hg.	STRONG	HIGH

BP: blood pressure; CV: cardiovascular; NPMs: non-pharmacological measures.

**Table:** 

Recommendations for therapeutic targets	Strength of recommendation	Certainty of evidence
A BP target of < 130/80 mm Hg is recommended for patients with BP 130-139/80-89 mm Hg and high CV risk.	STRONG	HIGH
A BP target of < 130/80 mm Hg is recommended for patients with hypertension, regardless of low, moderate, or high CV risk.	STRONG	HIGH
For patients who do not tolerate the < 130/80 mm Hg BP target, BP should be reduced to the lowest level tolerated.	STRONG	MODERATE
BP target achievement should be confirmed using out-of-office measurements (ABPM or HBPM).	STRONG	LOW

ABPM: ambulatory blood pressure monitoring; BP: blood pressure; CV: cardiovascular; HBPM: home blood pressure monitoring.

**Chapter 6:** Nonpharmacological measures

Recommendations for non-pharmacological measures	Strength of recommendation	Certainty of evidence
Reducing body weight is recommended for BP and mortality reduction in patients with obesity.	STRONG	HIGH
Reducing sodium intake and increasing dietary potassium intake (except for patients with CKD) is recommended for BP reduction.	STRONG	HIGH
DASH diet and moderate physical activity are recommended for BP reduction.	STRONG	HIGH

BP: blood pressure; CKD: chronic kidney disease; DASH: *Dietary Approaches to Stop Hypertension.*

**Chapter 7:** Pharmacological treatment

Recommendations for pharmacological treatment	Strength of recommendation	Certainty of evidence
The combination of antihypertensive drugs, preferably in a single-pill formulation and using preferred classes, is recommended to achieve strict BP targets (< 130/80 mm Hg) and reduce CV and renal events.	STRONG	MODERATE
Monotherapy is recommended for individuals with BP 130-139/80-89 mm Hg and high CV risk; patients with stage 1 hypertension and low risk (combination therapy may be considered at the physician's discretion); frail individuals; oldest-old adults (≥ 80 years); or those with symptomatic orthostatic hypotension, particularly in older adults.	WEAK	LOW
For most patients, initiation of treatment for hypertension with a two-drug regimen, preferably in a single pill combination, is recommended.	STRONG	MODERATE
Thiazide or thiazide-like diuretics, ACE inhibitors or ARBs, and CCBs are recommended as preferred classes for hypertension treatment and for reducing major CV and renal events.	STRONG	HIGH
BBs are recommended for the treatment of hypertension in specific situations: HF, AF, arrhythmias, CAD, hypertension in patients on hemodialysis, and other conditions (eg, migraine, essential tremor, women planning pregnancy, esophageal varices).	STRONG	MODERATE
Clonidine is recommended to achieve strict BP targets (< 130/80 mm Hg) and reduce CV and renal events in cases where BP targets are not met with initial classes alone (RH and RfH), and in those intolerant to spironolactone/eplerenone or still uncontrolled after their use.	STRONG	HIGH

ACE: angiotensin-converting enzyme; AF: atrial fibrillation; ARBs: angiotensin receptor blockers; BP: blood pressure; CAD: coronary artery disease; CCBs: calcium channel blockers; CV: cardiovascular; HF: heart failure; RfH: refractory hypertension; RH: resistant hypertension.

**Chapter 8:** Hypertension and associated clinical conditions: coronary artery disease, chronic kidney disease, diabetes, obesity, COVID-19, post-stroke, and heart failure

Recommendations for hypertension management in patients with coronary artery disease	Strength of recommendation	Certainty of evidence
BP targets < 130/80 mm Hg are recommended for individuals with hypertension and CAD. Lowering BP to 120/70 mm Hg does not increase CV risk, and asymptomatic patients with BP < 120/70 mm Hg do not need to discontinue or reduce their antihypertensive medication.	STRONG	MODERATE

BP: blood pressure; CAD: coronary artery disease; CV: cardiovascular.

**Table:** 

Recommendations for hypertension management in patients with chronic kidney disease on conservative treatment	Strength of recommendation	Certainty of evidence
In adults with hypertension and CKD, a BP target of < 130/80 mm Hg is recommended to reduce CV events and kidney failure.	STRONG	HIGH
The choice of antihypertensive agents in CKD-related hypertension should, unless contraindicated, include ACE inhibitors or ARBs, and favor the initiation and maintenance of drugs with modest antihypertensive effects but with proven nephroprotective and cardioprotective properties, such as finerenone, GLP-1 receptor agonists in patients with diabetes, and SGLT2 inhibitors, due to their established benefits in cardiorenal protection.	STRONG	HIGH

ACE: angiotensin-converting enzyme; ARB: angiotensin receptor blocker; BP: blood pressure; CKD: chronic kidney disease; GLP-1: glucagon-like peptide-1; SGLT2: sodium-glucose cotransporter 2.

**Table:** 

Recommendations for hypertension management in patients with diabetes	Strength of recommendation	Certainty of evidence
Pharmacological treatment should be initiated immediately upon diagnosis with two drugs: an ACE inhibitor or, in cases of intolerance to ACE inhibitors, an ARB, combined with either a CCB or a thiazide-like diuretic.	STRONG	HIGH

ACE: angiotensin-converting enzyme; ARB: angiotensin receptor blocker; CCB: calcium channel blocker.

**Table:** 

Recommendations for hypertension management in patients with obesity	Strength of recommendation	Certainty of evidence
A BP target of < 130/80 mm Hg is recommended for patients with obesity.	STRONG	MODERATE
Weight-loss medications such as GLP-1 receptor agonists (eg, liraglutide, semaglutide, dulaglutide) are recommended, as they have shown good efficacy in reducing weight and CV risk, although their effects on BP reduction are modest.	STRONG	HIGH

BB: beta-blocker; BP: blood pressure; CV: cardiovascular; GLP-1 RA: glucagon-like peptide-1 receptor agonist.

**Table:** 

Recommendations for hypertension management in post-stroke patients with cognitive impairment	Strength of recommendation	Certainty of evidence
A BP target of < 130/80 mm Hg is recommended for patients with prior stroke or TIA to reduce major CV events (MI, stroke recurrence, death).	STRONG	HIGH

BP: blood pressure; CV: cardiovascular; MI: myocardial infarction; TIA: transient ischemic attack.

**Table:** 

Recommendations for hypertension management in patients with heart failure	Strength of recommendation	Certainty of evidence
A BP target of < 130/80 mm Hg is recommended for patients with hypertension and HFpEF or HFrEF.	WEAK	LOW
The use of multiple antihypertensive drugs that improve prognosis – BBs, ACE inhibitors (or ARBs), and MRAs – is recommended in patients with HFrEF or HFpEF and hypertension.	STRONG	HIGH

ARB: angiotensin receptor blocker; BP: blood pressure; EF: ejection fraction; HF: heart failure; HFpEF: HF with preserved EF; HFrEF: HF with reduced EF.

**Chapter 9:** Hypertension in older adults, children, and adolescents

Recommendations for older patients	Strength of recommendation	Certainty of evidence
A BP target < 130/80 mm Hg is recommended for most older patients.	STRONG	HIGH
It is recommended that, for frail older adults, the oldest-old, or those with conditions that compromise life expectancy, BP be lowered to the lowest tolerated level.	STRONG	HIGH
In older patients on polypharmacy, each medication should be periodically reviewed, adverse events should be assessed, and antihypertensive therapy should involve the smallest possible number of daily pills. This can be achieved through the use of once-daily, fixed-dose combination antihypertensive medications, along with the promotion of NPMs.	STRONG	MODERATE

BP: blood pressure; NPMs: non-pharmacological measures.

**Table:** 

Recommendations for children and adolescents	Strength of recommendation	Certainty of evidence
In children and adolescents with symptomatic hypertension, HMOD, stage 2 hypertension without an identifiable modifiable cause, or persistent hypertension unresponsive to NPMs when BP is ≥ 95th percentile or ≥ 130/80 mm Hg in adolescents ≥ 13 years, it is recommended to initiate pharmacological treatment with an ACE inhibitor, ARB, long-acting CCB, or thiazide diuretic.	STRONG	LOW

ACE: angiotensin-converting enzyme; ARB: angiotensin receptor blocker; BP: blood pressure; CCB: calcium channel blocker; NPMs: non-pharmacological measures; HMOD: hypertension-mediated organ damage.

**Chapter 10:** Hypertension in women

Recommendations for pregnant and non-pregnant women	Strength of recommendation	Certainty of evidence
BP should be monitored before OCP prescription in young women. After initiation, regular monitoring (every 6 months) is recommended, including home BP monitoring and management of other CV risk factors.	STRONG	MODERATE
For the pharmacological treatment of hypertension in women during and after menopause, it is recommended to start with the combination of a RAS blocker with a CCB or a thiazide diuretic.	STRONG	MODERATE
BP targets should be the same for women and men, and the same classes of antihypertensive medications may be used for both sexes.	STRONG	MODERATE
In pregnant women with hypertension, it is recommended to initiate treatment with methyldopa or dihydropyridine CCBs (nifedipine retard or amlodipine).	STRONG	LOW
Long-term follow-up is recommended for all women with a history of hypertension in pregnancy to ensure effective prevention of CV disease.	STRONG	LOW

BP: blood pressure; CCB: calcium channel blocker; CV: cardiovascular; DBP: diastolic blood pressure; OCP: oral contraceptive pill; SBP: systolic blood pressure.

**Chapter 11:** Hypertensive crisis

Recommendations for the management of hypertensive crisis	Strength of recommendation	Certainty of evidence
In patients with severe BP elevation without acute HMOD (previously known as hypertensive urgency), outpatient reassessment should be performed within 1 to 7 days, with a target SBP < 160 and DBP < 100 mm Hg.	WEAK	LOW
Patients with HE should be admitted to the ICU and receive IV antihypertensives, with BP monitoring and observation of HMOD progression.	STRONG	MODERATE

BP: blood pressure; DBP: diastolic blood pressure; HE: hypertensive emergency; HMOD: hypertension-mediated organ damage; ICU: intensive care unit; IV: intravenous; SBP: systolic blood pressure.

**Chapter 12:** Resistant and refractory hypertension

Recommendations for the management of resistant and refractory hypertension	Strength of recommendation	Certainty of evidence
ABPM is preferred to confirm the diagnosis of RH and RfH, if available; otherwise, HBPM should be used.	STRONG	MODERATE
A BP target of < 130/80 mm Hg is recommended for patients with RH or RfH.	WEAK	LOW

ABPM: ambulatory blood pressure monitoring; BP: blood pressure; HBPM: home blood pressure monitoring; RfH: refractory hypertension; RH: resistant hypertension.

**Chapter 13:** Adherence to antihypertensive treatment

Recommendations for Adherence to Antihypertensive Treatment	Strength of recommendation	Certainty of evidence
Strategies involving the work of a multidisciplinary team, subsidies, communication/education resources, and applications are recommended to improve adherence to antihypertensive treatment.	WEAK	LOW

## 2. Epidemiology, Definition, and Primary Prevention

### 2.1. Definition, Epidemiology, and Primary Prevention

#### 2.1.1. Definition of Hypertension

Hypertension is a chronic noncommunicable disease defined as a persistent elevation of systolic blood pressure (SBP) greater than or equal to 140 mm Hg and/or diastolic BP (DBP) greater than or equal to 90 mm Hg, measured using the correct technique on at least two separate occasions, in the absence of antihypertensive medication. It is a multifactorial condition, dependent on genetic/epigenetic, environmental, and psychosocial factors. While the definition of hypertension has evolved over the years, it remains consistent with the terms outlined in the Brazilian Guidelines of Hypertension – 2020.^1^

#### 2.1.2. Epidemiological Data and Impact of Hypertension

The main epidemiological data on hypertension in Brazil are derived from the Brazilian Telephone Survey for Surveillance of Risk and Protective Factors for Chronic Diseases (VIGITEL) and the National Health Survey, both conducted by the Brazilian Ministry of Health. Vigitel, which started in 2006, is conducted annually through telephone interviews with individuals aged 18 years and older residing in the capitals of Brazil's 26 states and the Federal District.^2^ The National Health Survey is a nationwide, household-based, questionnaire-driven health survey that was conducted in 2013 and 2019.^3^ A major limitation of these surveys, particularly the 2019 National Health Survey and Vigitel, is that hypertension diagnoses are self-reported, which can significantly underestimate its prevalence. However, these surveys provide a comprehensive and evolving perspective on hypertension in the country.

Analysis of Vigitel data from 2006 to 2019 showed a mean prevalence of hypertension in adults of 24.5%, remaining stable during this period.^4^ After the COVID-19 pandemic, the 2023 Vigitel data indicated an upward trend in the prevalence of hypertension, reaching 27.9% (95% CI, 26.6%-29.2%). The state capital with the lowest prevalence was São Luís (19.2%; 95% CI, 15.7%-22.7%), while Rio de Janeiro showed the highest prevalence (34.4%; 95% CI, 29.8%-39.0%).^4^

In 2023, the World Health Organization (WHO) published global projections for hypertension, using much more alarming Brazilian data from 2019, estimating a global prevalence of 45% for Brazilians aged 30 to 70 years – 42% for women and 48% for men. According to this estimate, 67% of individuals with hypertension in Brazil were diagnosed, 62% were treated, and only 33% had controlled hypertension.^5^ However, the WHO publication does not specify the sample of the Brazilian population evaluated. Conversely, analyses of urban Brazilian population samples, in which the diagnosis of hypertension included blood pressure (BP) measurement, have shown a mean prevalence of hypertension of approximately 36%.^6^

The National Health Survey's longitudinal assessment indicates an improvement in self-care with BP measurement among Brazilian adults. The percentage of individuals who reported never having measured their BP decreased from 3% (95% CI, 2.7%-3.2%) in 2013 to 2% (95% CI, 1.8%-2.2%) in 2019. However, the diagnosis of hypertension showed an upward trend, rising from 21.4% (95% CI, 20.8%-22.0%) in 2013 to 23.9% (95% CI, 23.4%-24.4%) in 2019. Despite this, there are favorable data on the impact of hypertension on health, with a downward trend for limitations of daily activities due to the disease, decreasing from 12.1% (95% CI, 11.2%-13.1%) in 2013 to 9.8% (95% CI, 9.1%-10.5%) in 2019.^3^

Cardiovascular disease (CVD) remains the leading cause of death globally and in Brazil, with hypertension identified as the primary risk factor.^7,8^ In this context, data from the Global Burden of Cardiovascular Diseases and Risks for Brazil in 2019 indicate that hypertension was the main risk factor responsible for all-cause mortality in the country (104.8 per 100,000 population).^7^ Also in 2019, the WHO estimated that 54% of deaths from cardiovascular (CV) causes in Brazil were attributed to hypertension.^5^ The number of CVD deaths associated with hypertension has shown a concerning upward trend in the country (Figure 2.1).

**Figure 2.1:** Absolute number of deaths from cardiovascular diseases between 1996 and 2022 (Information Technology Department of the Brazilian Unified Health System [DATASUS]).^9^

### 2.3. Risk Factors for Hypertension

#### 2.3.1. Genetics

While rare single-gene mutations can be associated with hypertension, the genetic inheritance associated with primary hypertension is typically polygenic, involving thousands of genetic risk variants, each exerting a small-magnitude effect on BP. The probability of developing primary hypertension increases with the number of risk alleles and is modulated by environmental factors.^10^

#### 2.3.2. Age and Sex

The prevalence of hypertension increases steadily with age, reaching 65.1% in individuals over 65 years of age in 2023.^2^ In general, from late adolescence, men have a substantially higher prevalence of hypertension than women. However, women tend to experience a more pronounced increase in BP as they age, leading to a similar or even higher prevalence of hypertension than men after the age of 60 years.^11^

#### 2.3.3. Ethnicity, Urbanization, and Socioeconomic Factors

These three factors are closely connected and, in addition to influencing several relevant aspects (lifestyle, worldview, diet, treatment adherence, social vulnerability, physical activity, access to the health system, and level of education), are determinants of hypertension risk.

In addition to these factors, a residual risk related to race/ethnicity persists even after adjusting for socioeconomic, behavioral, and health indicators. In Brazil, a study showed an overall incidence of hypertension of 43.4 per 1000 person-years, being lower among White women (30.5/1000 person-years). Using the incidence rate in White women as a reference, hypertension was higher in Mixed-race women (1.47; 95% CI, 1.31-1.67), Black women (1.85; 95% CI, 1.50-2.30), White men (1.76; 95% CI, 1.49-2.08), Mixed-race men (1.89; 95% CI, 1.59-2.25), and Black men (2.25; 95% CI, 1.65-3.08).^12^ Furthermore, the contribution of hypertension to CV events is usually greater in the Black population than in the White population.^13,14^

Indigenous Brazilian peoples are traditionally socially vulnerable, and many groups are currently undergoing rapid urbanization. A meta-analysis including at least 33 Indigenous Brazilian ethnicities showed the highest prevalence of hypertension in Indigenous peoples living in urban areas of the South region of Brazil (30%; 95% CI, 10%-50%), while the lowest prevalence was observed in those living in less urbanized areas of the North region (1%; 95% CI, 1%-2%).^15^ Compared to non-Indigenous participants in the same study, Indigenous participants living in urban areas tended to have a similar prevalence of hypertension but greater odds of having uncontrolled PB than their non-Indigenous counterparts.^16^

#### 2.3.4. Spirituality and Psychosocial Factors

The SBC published a Position Statement on Hypertension and Spirituality in 2021. This Statement, based on extensive literature review, recognized that spirituality and religiosity can act through mechanisms such as stress reduction, improved self-care, and increased adherence to treatment, potentially influencing BP control.^17^

Psychosocial factors such as occupational stress, low socioeconomic status, social isolation, racial discrimination, depression, and anxiety have been identified as potential contributors to the development of hypertension and its complications. A meta-analysis showed that psychosocial stress was associated with a 2.4 (95% CI, 1.6-3.5) times higher risk of hypertension, and patients with hypertension had a 2.7 (95% CI, 2.3-3.1) times higher incidence of psychosocial stress than normotensive patients.^18^

#### 2.3.5. Overweight/Obesity and Physical Inactivity

The relationship between body mass index (BMI) and SBP or DBP is direct and continuous.^19^ Major consequences of overweight or obesity include a higher prevalence of hypertension and a cascade of associated metabolic disorders.^20^

Physical inactivity is a factor associated not only with hypertension but also with coronary artery disease (CAD), heart failure (HF), insulin resistance, type 2 diabetes mellitus (DM), dyslipidemia, stroke, dementia, and other chronic diseases.^21^

#### 2.3.6. Sodium and Potassium Intake

High sodium intake is a well-established risk factor for elevated BP and increased prevalence of hypertension in children and adults.^22,23^ Furthermore, reductions in BP can be achieved by using potassium-enriched salt substitutes.^24^

#### 2.3.7. Alcohol

Data from the 2023 Vigitel survey indicate a rise in alcohol abuse in Brazil, defined as four drinks/day for women and five drinks/day for men in at least 1 of the previous 30 days. This prevalence increased from 15.7% in 2006 to 20.8% in 2023, being particularly notable among women (7.8% to 15.2%).^2^ There is a strong, continuous, and nonlinear positive association between alcohol intake and BP.^25^ In fact, an intake exceeding 15 g/day for women and 30 g/day for men (equivalent to 7 and 14 drinks per week, respectively) has been identified as a risk factor for developing hypertension.^26^

#### 2.3.8. COVID-19

COVID-19 has not been conclusively demonstrated as a risk factor for the development of hypertension. A large Brazilian survey conducted from January 2019 to December 2020 indicated no significant effects of the pandemic on hypertension profiles for in-office or out-of-office BP measurements. In this study, treated patients (n = 27,699), compared with untreated patients (n = 24,227), showed a modest and transient improvement in in-office BP, which was restricted to the early months following the COVID-19 pandemic outbreak.^27^ However, hypertension appears to be a risk factor for a worse prognosis in patients with COVID-19. During the pandemic, Brazil experienced an increase in the CVD mortality rate, with hypertension being identified as one of the risk factors for fatal complications in individuals who developed COVID-19.^28,29^

#### 2.3.9. Medications, Illicit Drugs, and Anabolic Steroid Use

Some medications have the potential to elevate BP or hinder BP control, such as monoamine oxidase inhibitors, sympathomimetics, tricyclic antidepressants, thyroid hormones, oral contraceptives, nonsteroidal anti-inflammatory drugs, glucocorticoids, calcineurin inhibitors, carbenoxolone, licorice, erythropoietin, and testosterone. Illicit drugs can also lead to increased BP, such as cocaine, cannabis sativa, amphetamine, and 3,4-methylenedioxymethamphetamine (MDMA).^1^

There is an increasing use of testosterone among transgender individuals, with the prevalence of hypertension in transgender men ranging widely from 1.5% to 25.2%. The estimated prevalence of hypertension in transgender women has also varied widely, ranging from 1.5% to 29.2%. However, it remains unclear whether transgender individuals are at greater risk of developing hypertension.^30^ Conversely, the use of anabolic steroids for aesthetic and performance purposes is associated with hypertension as well as other CV and kidney diseases.^31^

#### 2.3.10. Sleep Disorders

An increased risk of hypertension has been demonstrated in relation to sleep deprivation, duration, and quality. Sleep disorders, such as obstructive sleep apnea, can affect regulatory mechanisms such as sympathetic nervous system activation, increased cortisol release, and chronic inflammation.^32-34^

#### 2.3.11. Smoking

Data from the 2023 Vigitel survey indicate a progressive reduction in smoking prevalence in Brazil from 15.7% in 2006 to 9.3% in 2018, remaining stable until 2023.^2^ While smoking is unquestionably related to increased CV morbidity and mortality, its direct role in inducing hypertension remains controversial.^35,36^ More recently, the use of electronic cigarettes, despite their prohibition in Brazil,^37^ has been a cause for concern, especially among adolescents. Substances found in these devices have been associated with CVD^38^ and have shown acute effects in elevating BP, but their long-term effects are still not fully understood.^39^

#### 2.3.12. Environmental Factors

The mechanisms linking environmental factors to an increased hypertension risk are complex and involve epigenetics, social factors, and gut microbiota composition.^40-42^ Noise pollution, for example, appears to be related to elevated BP mediated by increased psychosocial stress, while air pollution affects BP through physical and chemical pollutants that lead to endothelial dysfunction and increased oxidative stress. Furthermore, a lack of urban green spaces has been associated with physical inactivity and reduced social interaction, which are determinants of hypertension.^40^

The impact of climate on hypertension risk is also a complex but increasingly well-understood area. In temperate countries, increases in SBP are observed in winter temperatures (where norepinephrine levels rise) compared to summer temperatures (where nitric oxide and physical activity levels rise). Furthermore, extreme temperature variations during summer or winter are associated with an increase in CV events.^40^ In Brazil, regions with higher temperatures have a lower prevalence of hypertension, while regions with lower temperatures have a higher prevalence.^41^

Our understanding of the role of the gut microbiome in elevated BP has advanced in recent years. However, further studies are still needed to adequately assess its clinical relevance in the development of hypertension and its complications.^42^

Figure 2.2 summarizes the main risk factors for hypertension.

**Figure 2.2:** Main risk factors for hypertension.

### 2.4. Primary Prevention

Primary prevention of hypertension should be attempted through a patient-centered approach in order to promote healthier lifestyles (Figure 2.3).^5^ Chapter 6 of this Guideline provides detailed recommendations for non-pharmacological measures, which play an important role in both the prevention and management of hypertension.

**Figure 2.3:** FMeasures to prevent hypertension.

In general, a hypertension prevention plan should include weight management, healthy eating habits, regular physical activity, sufficient good quality sleep, stress management, control of alcohol consumption, and strengthening of positive social connections. Regarding smoking cessation, health professionals should consistently recommend it as a primary CV prevention measure, regardless of its potential benefit in reducing BP.^36,38^

The implementation of these measures requires individuals to actively engage and take responsibility for their health, fostering sustainable lifestyle changes. Furthermore, it is crucial to ensure public policies that support a culture of hypertension prevention and self-care.^43^

**Table:** 

Key messages
There is a high prevalence of hypertension in the Brazilian adult population with low rates of BP control.
Hypertension is the main determinant of CV mortality both in Brazil and worldwide.
Hypertension is multifactorial, and its risk factors include innate genetic determinants, aging, obesity, physical inactivity, psychosocial stress, inadequate diet, use of licit and illicit drugs, sleep disorders, alcohol abuse, and complex environmental factors, with different behavior according to sex, ethnic differences, and socioeconomic factors.
Hypertension prevention should be attempted through a patient-centered approach to promote healthier lifestyles that include weight management, healthy eating, regular physical activity, sufficient good quality sleep, stress management, control of alcohol consumption, and strengthening of positive social connections, reinforcing a sustainable multidisciplinary approach and encouraging public health promotion policies.

BP: blood pressure; CV: cardiovascular.

## 3. Blood Pressure Measurement, Diagnosis, and Classification

### 3.1. Introduction

BP measurement is essential for the initial evaluation of individuals with or without hypertension and should be performed at every medical appointment, by any health care professional, using appropriate techniques and validated, calibrated equipment. BP should be measured both in the office and outside of it, using either ambulatory blood pressure monitoring (ABPM) and/or home blood pressure monitoring (HBPM), to ensure an accurate diagnosis of hypertension and monitor control.^44-46^ The diagnostic assessment of hypertension includes confirmation of the condition, identification of secondary causes, and evaluation of CV risk. It is essential to investigate the possibility of hypertension-mediated organ damage (HMOD) and associated diseases. This process should preferably involve multiple BP measurements on different occasions, a complete medical history, a detailed physical examination, and clinical and laboratory investigation.

### 3.2. Office Blood Pressure Measurement

BP should be measured at every evaluation by any trained health care professional. However, the diagnosis of hypertension and its phenotypes, as well as clinical management, are the sole responsibility of physicians.^46^

#### 3.2.1. Blood Pressure Measurement Techniques

BP should be measured using the correct technique and adequate equipment.^46-48^ Automated or semi-automated sphygmomanometers using the oscillometric technique offer advantages over the auscultatory method with aneroid sphygmomanometers. These advantages include a reduction in observer-related errors and greater ease of execution.^44-46^ Another benefit is the possibility of performing unattended BP measurement in the office, in which the patient takes the measurement alone in a private room without the presence of a health care professional. This approach improves measurement reproducibility and reduces the white-coat effect (WCE).^46,49^ Nevertheless, it is essential that devices be validated according to standardized protocols and their calibration be checked at least annually.^46^

BP should initially be measured in both arms, and subsequent measurements should be taken in the arm with the higher reading. An SBP difference greater than 15 mm Hg between arms indicates increased CV risk and may suggest the presence of obstructive CAD.^50^ This difference is also associated with adverse left ventricular remodeling.^51^

#### 3.2.2. Special Populations

In older adults and in individuals with DM, autonomic dysfunction, or those using antihypertensive agents, BP should also be measured in the supine position and again 1 and 3 minutes after standing, with the arm supported at heart level by the examiner. This is important for identifying orthostatic hypotension (OH), which is defined as a decrease in SBP ≥ 20 mm Hg and/or DBP ≥ 10 mm Hg.^45,46,52,53^

It is important to use cuffs that are appropriate for the size and shape of the arm of the patient to avoid underestimation or overestimation of BP. When arm measurement is challenging, particularly in individuals with obesity, wrist measurement using the radial artery with validated devices may be considered.^46^ If upper limb BP measurement is not feasible, measurement in the lower limbs, especially at the calves, using oscillometric devices has been suggested.^54^

Inaccurate BP measurements can lead to incorrect diagnoses and inadequate treatment. Therefore, it is crucial to follow strict protocols to ensure accuracy and effectiveness in the management of hypertension. Figure 3.1 and Chart 3.1 illustrate the steps for BP measurement. For more detailed information on the recommended techniques, refer to the Brazilian Guidelines for In-office and Out-of-office Blood Pressure Measurement.^46^ Adherence to these guidelines ensures that BP measurements are performed with the highest precision, minimizing errors and improving the management of patients with hypertension.

**Figure 3.1:** Steps for office blood pressure measurement.

**Chart 3.1:** Procedure for office blood pressure measurement^46^

Steps for BP measurement
Wrap the cuff snugly around the arm, 2 to 3 cm above the antecubital fossa, positioning the center of the cuff bladder directly over the brachial artery.
Estimate systolic BP level.*
Place the bell or diaphragm of the stethoscope over the brachial artery in the antecubital fossa without applying too much pressure.*
Inflate the cuff rapidly until the estimated SBP level is exceeded by 20-30 mm Hg.**
Proceed to deflate the cuff slowly (approximately 2 mm Hg/s).**
Determine SBP by auscultation of the first Korotkoff sound.*
Determine DBP when the sounds disappear.*
Take 3 measurements at 1-minute intervals and use the average of the last 2 readings. If the difference between readings is greater than 10 mm Hg, take additional measurements.

BP: blood pressure; DBP: diastolic blood pressure; SBP: systolic blood pressure.

* Applied to the auscultatory technique.

#### 3.2.3. Classification

Based on office BP measurements, individuals are considered hypertensive if SBP ≥ 140 mm Hg and/or DBP ≥ 90 mm Hg. The classification of BP is detailed in Chart 3.2.^46^ The new BP classification proposed by this Guideline introduces several important changes compared to the 2020 Brazilian Guidelines on Hypertension. The main changes include:

**Chart 3.2:** Classification of blood pressure from office measurements, ages 18 and more

Classification of BP	SBP (mm Hg)		DBP (mm Hg)
**Normal BP**	< 120	and	< 80
**Prehypertension**	120-139	and/or	80-89
Stage 1 hypertension	140-159	and/or	90-99
Stage 2 hypertension	160-179	and/or	100-109
Stage 3 hypertension	≥ 180	and/or	110

BHG: Brazilian Hypertension Guidelines; BP: blood pressure; DBP: diastolic blood pressure; SBP: systolic blood pressure.

* Classification follows office BP and the highest BP level, either systolic or diastolic.

** Isolated systolic hypertension, characterized by SBP ≥ 140 mm Hg and DBP < 90 mm Hg, is classified into stage 1, 2, or 3 according to SBP values at the intervals indicated.

*** Isolated diastolic hypertension, characterized by SBP < 140 mm Hg and DBP ≥ 90 mm Hg, is classified into stage 1, 2, or 3 according to SBP values at the intervals indicated.

Optimum BP: The "optimum BP" category has been removed. This BP range, previously considered optimal, is now included under the new term "Normal BP", defined as SBP < 120 mm Hg and DBP < 80 mm Hg.

Prehypertension: The new "Prehypertension" category now includes values that were previously classified as "Normal BP" and "Prehypertension" in the 2020 Guidelines. This means SBP values between 120-129 mm Hg and/or DBP values between 80-84 mm Hg, which were previously considered normal, are now reclassified as prehypertensive. As such, the prehypertension category includes SBP from 120-139 mm Hg and/or DBP from 80-89 mm Hg. These changes aim to identify at-risk individuals early and to encourage more proactive interventions to prevent progression to hypertension.

To validate the diagnosis of hypertension, repeated BP measurements are required during two or more medical examinations, spaced days or weeks apart. Alternatively, ABPM or HBPM may be used for a more accurate assessment. However, in patients with HMOD or established CVD, the presence of these findings is sufficient to confirm the diagnosis of hypertension, eliminating the need for out-of-office BP measurement. Hypertension classification is based on the highest BP value, either systolic or diastolic, measured in the office.^44-46^

Individuals with SBP ≥ 140 mm Hg and/or DBP < 90 mm Hg are classified as having isolated systolic hypertension, whereas those with SBP < 140 mm Hg and DBP ≥ 90 mm Hg are classified as having isolated diastolic hypertension.^46^

### 3.3. Out-of-Office Blood Pressure Measurement

Out-of-office BP may be measured using ABPM or HBPM. These BP measurement modalities are essential to confirm the diagnosis of hypertension and monitor BP control, particularly in patients with suspected white-coat hypertension (WCH) or masked hypertension (MH). Chart 3.3 summarizes the advantages and disadvantages of these methods.

**Chart 3.3:** Main advantages and disadvantages of out-of-office blood pressure measurement^46^

Advantages	ABPM	HBPM
Allows out-of-office BP measurement without the presence of a physician	X	X
Is correlated with better prognosis than office BP measurement	X	X
Potentially reduces health care costs	X	X
Is the current gold standard for BP assessment	X	
Assesses BP control over 24 hours, including during sleep and activities of daily living	X	
Allows assessment of morning BP surge	X	
Allows long-term monitoring of treatment efficacy and increases treatment adherence		X
Improves BP control and treatment adherence		X
Is less expensive than ABPM		X
**Disadvantages**		
Potentially limited availability	X	X
Some patients are reluctant to use and repeat the test	X	X
It may be uncomfortable, especially during sleep	X	
Nighttime BP is often not calculated according to the patient's sleep schedule	X	
BP measurement only at rest during daytime		X
Guidance and training are required; potential measurement errors: measurements at inadequate times, excessive number of measurements, induction of anxiety, change of medication by the patient, patient underreports BP values		X

BP: blood pressure; ABPM: ambulatory blood pressure monitoring; HBPM: home blood pressure monitoring.

#### 3.3.1. Indications for Ambulatory or Home Blood Pressure Monitoring

Both ABPM and HBPM are indicated in several important clinical scenarios. These include suspected WCH, MH, assessment of antihypertensive treatment efficacy, and the diagnosis and follow-up of hypertension in patients with significant BP variability. HBPM can be integrated into telemedicine, increasing treatment adherence and BP control, especially when combined with medical guidance. ABPM is particularly useful for evaluating BP behavior during sleep. According to a recent study, it can also be combined with polysomnography without affecting total sleep time, sleep efficiency, or the number of awakenings.^55^ Abnormal BP values are summarized in Chart 3.4, and the main indications for these methods are described in Chart 3.5.^46,56-60^

**Chart 3.4:** Definition of hypertension according to office, ambulatory, and home blood pressure monitoring

Category		SBP (mm Hg)		DBP (mm Hg)
Office BP		≥ 140	and/or	≥ 90
24-hour ABPM		≥ 130	and/or	≥ 80
	Daytime	≥ 135	and/or	≥ 85
	Sleep	≥ 120	and/or	≥ 70
HBPM		≥ 130	and/or	≥ 80

BP: blood pressure; SBP: systolic blood pressure; DBP: diastolic blood pressure; ABPM: ambulatory blood pressure monitoring; HBPM: home blood pressure monitoring.

**Chart 3.5:** Indications for ambulatory or home blood pressure monitoring^46^

Indications	ABPM	HBPM
Suspected WCH – white-coat effect	X	X
Stage 1 hypertension (140-159 and/or 90-99 mm Hg) in the office	X	X
BP ≥ 140/90 mm Hg in the office without HMOD and low CV risk	X	X
Isolated systolic or isolated diastolic hypertension in the office	X	X
Suspected MH – masking effect	X	X
BP in the prehypertension range (130-139 and/or 85-89 mmHg)	X	X
BP < 140/90 mmHg in the office with HMOD and high CV risk	X	X
Suspected white-coat effect	X	X
Elevated office BP or suspected preeclampsia in pregnant women	X	X
Investigation of UH, RH, and excessive decline in BP	X	X
Adjustment of antihypertensive medication	X	X
Ensuring adequate BP control	X	X
Evaluation of BP control over 24 hours, including during sleep and activities of daily living	X	
Investigation of postural and postprandial hypotension, as well as during siesta	X	
Evaluation of BP variability in dysautonomia	X	
Evaluation of symptoms, especially hypotension	X	
Monitoring long-term treatment efficacy and improving adherence		X

ABPM: ambulatory blood pressure monitoring; BP: blood pressure; CV: cardiovascular; HBPM: home blood pressure monitoring; RH: resistant hypertension; HMOD: hypertension-mediated organ damage; UH: uncontrolled hypertension; WCH: white-coat hypertension; MH: masked hypertension.

### 3.4. White-Coat Effect and Masked Effect

The difference in BP between measurements taken in and out of the physician's office is known as the WCE or ME. The WCE is considered significant when office SBP is ≥ 20 mm Hg (compared to 24-hour ABPM average) or ≥ 15 mm Hg (compared to HBPM), or when DBP is ≥ 15 mm Hg (ABPM) or ≥ 9 mm Hg (HBPM), in relation to out-of-office measurements.^61,62^ Conversely, the ME is considered significant when office SBP is ≤ 2 mm Hg (ABPM) or ≤ –1 mm Hg (HBPM), or DBP is ≤ 2 mm Hg (ABPM) or ≤ –1 mm Hg (HBPM), compared to out-of-office values.^61,62^ These variations do not change the diagnosis of hypertension, but they are useful in identifying which patients should undergo out-of-office BP monitoring more frequently. This contributes to more effective therapeutic management.

### 3.5. White Coat Hypertension and Masked Hypertension

WCH occurs when BP is elevated in the medical office (≥ 140 mm Hg and/or 90 mm Hg) but normal outside of it (< 130/80 mm Hg on HBPM or 24-hour ABPM), whereas MH is characterized by normal office BP but elevated out-of-office BP.

The prevalence of WCH ranges from 7% to 15% and is more common in patients with stage 1 hypertension and in those with isolated systolic or diastolic hypertension.^46,56,57^ The prevalence of MH ranges from 8-22%, being more frequent in individuals with additional risk factors such as DM and obesity, and may reach up to 62% in individuals with prehypertension (Figure 3.2).^46^ However, in individuals with DM, MH appears to be better identified through ABPM than HBPM.^46,63^ Both WCH and MH are associated with an increased risk of developing DM, HMOD, and long-term CV events. Conversely, individuals with normal BP both in and out of the office are classified as having true normotension, while those with elevated BP both in and out of the office are considered to have sustained hypertension. For a more comprehensive review, refer to the *Brazilian Guidelines for In-office and Out-of-office Blood Pressure Measurement*.^46^

**Figure 3.2:** Prevalence of blood pressure phenotypes in patients treated or not with antihypertensive medications.

### 3.6. Uncontrolled Masked Hypertension and Uncontrolled White-Coat Hypertension

Patients on antihypertensive medications may also show either concordance or discrepancy between office and out-of-office BP measurements. The corresponding terms for true normotension, WCH, MH, and sustained hypertension in treated individuals are controlled hypertension, uncontrolled WCH (uWCH), uncontrolled MH (uMH), and uncontrolled sustained hypertension. In patients on antihypertensive medication, this Guideline considers BP values to be elevated in and out of the office (HBPM or 24-hour ABPM) when they are ≥ 130 mm Hg and/or 80 mm Hg. The prevalence of each BP phenotype is shown in Figure 3.2.^64,65^ For a more comprehensive review, refer to the Brazilian Guidelines for In-office and Out-of-office Blood Pressure Measurement.^46^

### 3.7. Recommendations for Diagnosis and Follow-Up

Hypertension is often asymptomatic; therefore, it should be investigated in every medical visit and in population screening programs. BP measurements should be performed regularly, with the frequency depending on the BP classification. Figure 3.3 summarizes the recommendations for the diagnosis and follow-up of hypertension, considering BP measurements both in and out of the office.^46^

**Figure 3.3:** Screening and diagnosis of hypertension.

Due to the high variability of BP, the diagnosis of hypertension should not be based on a single office measurement, unless BP is substantially elevated (stage 3 hypertension) or there is an established diagnosis of HMOD or CVD. It is recommended that elevated BP be confirmed with repeated measurements during subsequent medical visits. Patients with stage 2 or 3 hypertension require more frequent visits at shorter intervals, while those with stage 1 hypertension may be followed at longer intervals, provided there is no HMOD and CV risk is low.^46^

Conversely, when logistically and economically feasible, ABPM and HBPM should be performed to confirm the diagnosis of hypertension and monitor treatment, as these methods are superior to office BP measurement in predicting CV risk.^46,66,67^ This approach is also essential for identifying WCH and MH, providing valuable clinical information.^46^

#### 3.7.1. Self-Measured Blood Pressure

Self-measured BP refers to BP readings taken by the patient or a family member at home, without following any predefined protocol, and using the patient's own automatic device.^46^ Currently, there is no conclusive evidence to support the establishment of normative values for this method, nor the adoption of specific protocols (eg, number of measurements, timing, or days of monitoring) for its evaluation.^46^ Therefore, this Guideline recommends that self-measured BP be used solely as a screening tool, with confirmatory methods – such as ABPM or HBPM – being requested when indicated.

#### 3.7.2. Exercise Stress Test

The exercise stress test is not recommended for the diagnostic evaluation of hypertension due to the lack of standardization and consensus on the normal BP response during exercise testing.^68^

### 3.8. Central Blood Pressure, Pulse Wave Velocity, and Augmentation Index

Non-invasive estimation of central BP (CBP) can be conducted using specific algorithms based on brachial BP measurements.^69^ Studies suggest that CBP may be a better predictor of CV events than brachial BP, although further evidence is needed to confirm its incremental prognostic value.^46^

Spurious hypertension, commonly observed in young adults – especially male athletes – is the main indication for CBP measurement.^46,70^ Associated parameters, such as pulse wave velocity (PWV) and the augmentation index, have well-established prognostic value and may be useful for more accurate CV risk stratification. For a more comprehensive discussion on this topic, refer to the *Brazilian Guidelines for In-office and Out-of-office Blood Pressure Measurement*.^46^

### 3.9. New Technologies for Blood Pressure Measurement

Although the traditional cuff-based method is widely used, it has limitations, such as offering only intermittent and static measurements and being prone to errors related to cuff size or positioning. To address these issues, new technologies have emerged that allow for continuous and more comfortable BP monitoring without the use of a cuff. These innovations offer several advantages, including the ability to take measurements without disrupting sleep or daily activities, as well as facilitating self-monitoring and improving treatment adherence.^71^

These new devices work by analyzing pulse wave characteristics, pulse arrival time, and pulse transit time. However, despite their promise, concerns remain regarding the accuracy of these methods. Challenges such as calibration, applicability across diverse populations, and the lack of transparency in the algorithms used raise important concerns.^72,73^ Therefore, clinical use of these technologies should be approached with caution until more robust evidence is available regarding their accuracy and performance.

**Table:** 

Recommendations for blood pressure measurement, diagnosis, and classification	Strength of recommendation	Certainty of evidence
Prehypertension should be classified as office SBP between 120-139 mm Hg or office DBP between 80-89 mm Hg to identify individuals at-risk early and encourage proactive, non-pharmacological measures to prevent progression to hypertension.	STRONG	MODERATE
Hypertension should be diagnosed when office BP is ≥ 140 and/or 90 mm Hg on two separate occasions, and classified into stages 1, 2, or 3 according to the highest SBP or DBP value.	STRONG	MODERATE
BP should be measured using adequate techniques and equipment.	STRONG	MODERATE
The use of automated upper-arm devices is recommended to reduce measurement errors and facilitate BP monitoring.	STRONG	MODERATE
It is recommended to assess for orthostatic hypotension in at-risk groups (older adults, patients with diabetes, autonomic dysfunction, or using antihypertensive medications).	STRONG	HIGH
The use of ABPM or HBPM is recommended to confirm the diagnosis of hypertension and monitor treatment.	STRONG	HIGH
The primary indication for central BP assessment is the evaluation of spurious systolic hypertension in young adults.	WEAK	LOW

ABPM: ambulatory blood pressure monitoring; BP: blood pressure; DBP: diastolic blood pressure; HBPM: home blood pressure monitoring; SBP: systolic blood pressure.

## 4. Clinical and Complementary Assessment and Cardiovascular Risk Stratification

The clinical and complementary assessment of individuals with hypertension aims not only to establish the presence of hypertension, its underlying determinants, and potential secondary causes, but also to identify associated CV risk factors and HMOD.^45,74-76^

CV risk stratification is a systematic process designed to categorize patients according to their individual risk of developing major CV and renal outcomes. This process involves the integration of multiple CV risk factors and HMOD markers through equations or algorithms that estimate risk using multivariable regression models derived from population-based studies.^74^

### 4.1. Additional Risk Stratification (Associated Conditions)

Additional risk stratification in patients with hypertension involves identifying and managing associated conditions that, on their own, may increase cardiovascular risk, regardless of BP levels. Among these conditions are coexisting risk factors such as DM, smoking, dyslipidemia, and obesity, as well as HMOD, including left ventricular hypertrophy (LVH), retinopathy, arterial stiffness, peripheral artery disease (PAD), and chronic kidney disease (CKD).^45,74^ Patients with multiple risk factors and/or HMOD have a significantly higher risk of developing major CVDs such as acute myocardial infarction (MI), stroke, HF, and death.^45,74^ The linear correlation between age and CV risk, with a marked increase in men over 55 and women over 65,^76^ highlights the importance of early interventions in high-risk populations.

Among metabolic risk factors, dyslipidemia – characterized by elevated levels of low-density lipoprotein cholesterol and triglycerides, along with reduced high-density lipoprotein cholesterol – is one of the major contributors to the progression of atherosclerosis.^77^ Similarly, DM, diagnosed according to established criteria such as fasting plasma glucose ≥ 126 mg/dL and glycated hemoglobin (HbA1c) ≥ 6.5%, is strongly associated with increased CV mortality in patients with hypertension, underscoring the importance of strict glycemic control in this population. In addition, obesity, assessed via BMI and waist circumference, is a well-established risk factor in hypertension, contributing to the development of insulin resistance and endothelial dysfunction.^78^

### 4.2. Clinical and Complementary Assessment

The clinical and complementary assessment of patients with hypertension should follow the traditional approach, consisting of medical history, physical examination, and complementary tests.

#### 4.2.1. Medical History

Although hypertension is typically asymptomatic, changes resulting from HMOD and CV, renal, or neurological complications may lead to a variety of symptoms. Investigating the determinants of hypertension, as described in Chapter 2 – including dietary habits, weight gain, salt intake, alcohol consumption, sleep patterns, physical activity level, as well as socioeconomic, biopsychosocial, cultural, and environmental factors – is essential. Family history should also be obtained to support the diagnosis of primary hypertension.^79^ A detailed record of disease duration, previous or current medication use, and corresponding dosages is also critical for adequate assessment and management. In addition, secondary causes or contributing factors, such as medications or substances that may affect BP, should also be investigated when taking the patient's medical history.

Chart 4.1 summarizes the main objectives to be achieved when taking medical history.

**Chart 4.1:** Main objectives of medical history

Investigate symptoms that may be related to hypertension
Ask about family history of hypertension
Understand dietary habits (salt intake and type of diet) and addictions, such as history of smoking, alcohol use, and illicit drug use (duration and quantity)
Evaluate adherence to nonpharmacological measures, antihypertensive therapy, and other medications, especially those that may interfere with blood pressure control
Inquire about pregnancy history, particularly prior preeclampsia or eclampsia, in women
Identify associated cardiovascular and renal risk factors and apply a risk score
Screen for hypertension-mediated organ damage
Investigate the presence of other associated diseases
Ask about physical inactivity and stress level
Ask about sleep history, including snoring and sleep apnea
Investigate history of erectile dysfunction
Screen for signs suggestive of secondary hypertension

#### 4.2.2. Physical Examination

Physical examination can help assess the severity and progression of hypertension, detect secondary forms, and identify signs of HMOD and associated risk factors. While one of the main objectives of physical examination is to accurately measure BP, as detailed in Chapter 3, the assessment of all organ systems is important, particularly the CV system.^80^ The ankle-brachial index (ABI), calculated as the ratio of brachial SBP to ankle SBP, is also part of the physical examination and improves the accuracy of CV risk prediction.^81^ Anthropometric assessment, including measurement of weight, height, and waist circumference, allows for the calculation of BMI, the diagnosis of obesity, and the estimation of abdominal obesity.^82^

The key components of a physical examination in patients with hypertension are presented in Figure 4.1 and Chart 4.2.

**Figure 4.1:** Key components of physical examination in patients with hypertension.

**Chart 4.2:** Anthropometric and ankle-brachial index parameters. Note: WC should be measured at the midpoint between the lowest rib and the top of the iliac crest, laterally.^83,84^

BMI (kg/m^2^)	WC (cm)	ABI
Normal: < 24,9 Overweight 25-29,9 Obesidade: ≥ 30	Normal WC: Women: < 88 cm Men: < 102 cm	Normal: > 0.90 Mild obstruction: 0.71-0.90 Moderate obstruction: 0.41-0.70 Severe obstruction: ≤0.40

#### 4.2.3. Additional Tests

Chart 4.3 lists routine laboratory tests that should be requested for patients with hypertension at the first medical visit and at least annually, provided the results remain within reference ranges. If abnormalities are detected, follow-up should occur at appropriate intervals to monitor the effects of the treatment implemented.

**Chart 4.3:** Routine laboratory tests

Urinalysis
Plasma potassium
Plasma creatinine
eGFR using the CKD-EPI equation*
Protein-to-creatinine or albumin-to-creatinine ratio
Fasting blood glucose and glycated hemoglobin (for patients at risk of diabetes or metabolic syndrome)
Serum total cholesterol, LDL-c, HDL-c, and triglycerides**
Plasma uric acid
Eletrocardiograma convencional

CKD-EPI: Chronic Kidney Disease Epidemiology Collaboration; eGFR: estimated glomerular filtration rate; HDL-c: high-density lipoprotein cholesterol; LDL-c: low-density lipoprotein cholesterol.

* The CKD-EPI equation estimates eGFR based on sex, age, and plasma creatinine levels.^85^

** LDL-c can be calculated using the following formula: LDL-c = total cholesterol – (HDL-c + triglycerides/5) (when triglyceride levels are <400 mg/dL).^86^ LDL-c is also directly measured in some laboratories as part of routine testing.

### 4.4. Hypertension-Mediated Organ Damage

The assessment of HMOD should be performed in all patients at the time of hypertension diagnosis. The choice of method(s) may vary depending on the availability of resources. Electrocardiogram (ECG), echocardiogram, estimated glomerular filtration rate (eGFR), and albuminuria are widely available tests with proven added value for risk stratification and for defining therapeutic goals in patients with hypertension.^87^ It is recommended that HMOD assessment be repeated over time to monitor its evolution (whether progression, stabilization, or regression) and to guide timely therapeutic interventions.^88,89^ The criteria used for HMOD detection are described in Chart 4.4.^81,90-96^

**Chart 4.4:** Criteria for diagnosing hypertension-mediated organ damage

Organ	Method	Criteria
Heart	ECG	Sokolow-Lyon index (S wave in V1 + R wave in V5 or V6) > 35 mm; R wave in aVL ≥ 11 mm; Cornell voltage index (S wave in V3 + R wave in aVL) > 28 mm in men and > 20 mm in women.^90,91^
	Echocardiogram	LVMI ≥ 116 g/m² in men or ≥ 96 g/m² in women (indexed by body surface area), or ≥ 45 g/m² in men or ≥ 49 g/m² in women (indexed by height^2,7^).^92^
Large vessels	ABI	ABI < 0.9.^81^
	Carotid ultrasound	Presence of plaques.^93^
	PWV	Carotid-femoral PWV ≥ 10 m/s.^94^
Kidney	eGFR	Chronic kidney disease stage 3a or higher (eGFR < 60 mL/min/1.73 m²).^95^
	Albuminuria	Albuminuria ≥ 30 mg/24 h or albumin-to-creatinine ratio ≥ 30 mg/g.^95^
Retina	Fundoscopy	Hypertensive retinopathy (KWB).^96^

ABI: ankle-brachial index; ECG: electrocardiogram; eGFR: estimated glomerular filtration rate; LVMI: left ventricular mass index; PWV: pulse wave velocity; KWB: Keith–Wagener–Barker.

The time required to detect significant changes in HMOD after treatment adjustments varies according to the target organ and the method used. Albuminuria and PWV are the parameters that tend to respond most quickly to antihypertensive control (within weeks to a few months).^97^ Although there are no clinical trials specifically designed to determine the optimal frequency for monitoring HMOD, patients with uncontrolled BP and/or evidence of HMOD at baseline should be reassessed more frequently (every 6 months to 1 year) until BP goals are achieved and HMOD is stabilized or reversed.^88,98^

### 4.5. Chronic Kidney Disease Assessment

CKD is defined as abnormalities of kidney structure or function, present for a minimum of 3 months, with implications for health.^95^ CKD is classified into five stages based on eGFR, as shown in Figure 4.2, in accordance with the Kidney Disease: Improving Global Outcomes (KDIGO) recommendations.^95^ At any stage – especially from stage G3a onward (eGFR < 60 mL/min/1.73 m²) – there is an increased risk of CVD, specific renal outcomes, and both CV and all-cause mortality.^95,99^ It is worth noting that an albumin-to-creatinine ratio (ACR) ≥ 30 mg/g is associated with higher mortality and greater rates of CV events, even in patients with preserved renal function (Figure 4.2).^95^ To adequately assess CV risk in patients with CKD, it is essential to use risk calculators that include kidney function, as calculators that omit eGFR may underestimate CV risk in this population.^95^

**Figure 4.2:** Assessment and prognosis of chronic kidney disease Adapted from KDIGO 2024 Clinical Practice Guideline for the Evaluation and Management of Chronic Kidney Disease.^95^

### 4.6. Assessment of Secondary Causes

Secondary hypertension (SH) refers to a form of hypertension caused by an identifiable underlying condition, which can be treated with a specific intervention, potentially resulting in a cure or improved BP control. The true prevalence of SH is unknown but is estimated to be between 5% and 10%.^100^ It should be investigated when there are clinical indicators (from medical history, physical examination, or routine tests) that suggest a potential underlying cause (Chart 4.5). Patients should also be asked about the use of hormones, medications, and other substances that may increase BP or hinder its control, as discussed in Chapter 2. Individuals with SH typically face higher CV and renal risk and exhibit more pronounced HMOD, primarily due to more severe and sustained elevations in BP. Further information on the diagnosis and treatment of SH can be found in the 2020 Brazilian Hypertension Guidelines.^1^

**Chart 4.5:** Main causes of secondary hypertension: nonendocrine and endocrine, suggestive signs, and diagnostic screening

Diagnostic suspicion	Clinical findings	Additional findings
**Non-endocrine causes**		
Renal parenchymal disease	Edema, anemia, anorexia, fatigue, elevated creatinine and urea levels, and abnormal urinary sediment or imaging findings	Serum creatinine with eGFR, renal ultrasound, urinalysis for proteinuria and/or dysmorphic hematuria. Protein-to-creatinine or albumin-to-creatinine ratio
Renal artery stenosis	Sudden-onset or worsening hypertension without apparent cause before age 30 or after age 55, resistant or refractory hypertension, malignant hypertension, abdominal bruit, sudden pulmonary edema, unexplained deterioration of renal function or due to RAAS-blocking agents, asymmetric renal size > 1.5 cm	Renal Doppler ultrasound with measurement of flow velocity and resistive index (screening, observer-dependent), MR angiography or spiral CT angiography. Gold standard: conventional renal angiography
OSA	More common in men or postmenopausal women; snoring most nights, sleep fragmentation with choking or gasping, excessive daytime sleepiness, nonrestorative sleep, fatigue, nocturia, morning headache, metabolic syndrome	Screening questionnaires have low accuracy. Gold standard: polysomnography or polygraphy (for patients with high pretest probability). AHI < 5 events/h: no OSA; AHI 5-14.9: mild OSA; AHI 15-29.9: moderate OSA; AHI ≥ 30: severe OSA
Coarctation of the aorta	Lower limb weakness, reduced or absent pulses in the lower limbs, systolic BP in upper limbs > 10 mm Hg higher than in lower limbs, interscapular systolic murmur or chest murmur	Chest radiography, echocardiography (screening), CT angiography of the aorta or, preferably, magnetic resonance angiography (gold standard). Invasive angiography only when additional information is necessary.
**Endocrine causes**		
Primary aldosteronism (hyperplasia or adenoma)	Resistant or refractory hypertension and/or hypokalemia (not mandatory) and/or adrenal nodule	Ratio of plasma aldosterone concentration to plasma renin activity, followed by confirmatory tests (saline infusion test, captopril challenge, fludrocortisone suppression test, IV furosemide test). Imaging: thin-slice CT or MR imaging. Selective adrenal vein sampling for aldosterone and cortisol to determine subtype, if indicated
Pheochromocytoma and paraganglioma	Paroxysmal hypertension with the classic triad of headache, sweating, and palpitations	Plasma free metanephrines and urinary fractionated metanephrines (screening), MR imaging and scintigraphy when indicated
Hypothyroidism	Fatigue, weight gain, hair loss, diastolic hypertension, muscle weakness, somnolence	Screening: TSH and free T4
Hyperthyroidism	Heat intolerance, weight loss, tachycardia/palpitations, exophthalmos, hyperthermia, hyperreflexia, tremors, goiter	Screening: TSH and free T4
Hyperparathyroidism (hyperplasia or adenoma)	Nephrolithiasis, osteoporosis, depression, lethargy, muscle weakness or spasms, thirst, polyuria, polydipsia, constipation	Total and/or ionized calcium, phosphate, PTH, 24-hour urine calcium, and vitamin D levels
Cushing's syndrome (hyperplasia, adenoma, or ACTH overproduction)	Weight gain, decreased libido, fatigue, hirsutism, amenorrhea, moon facies, dorsal hump, purple striae, truncal obesity, hypokalemia	Baseline cortisol, midnight salivary cortisol, 24-hour urinary free cortisol, dexamethasone or betamethasone suppression test: take 1 mg dexamethasone at 11:00-12:00 PM and measure morning cortisol at 7:00-8:00 AM. Imaging: CT, MR imaging
Class I obesity: BMI 30 to 34.9 kg/m^2^; class 2: BMI 35 to < 40 kg/m^2^; class 3: BMI ≥ 40 kg/m^2^	Increased central or generalized fat	BMI (weight in kg/height in m²) and abdominal circumference (>102 cm in men, >88 cm in women). Imaging tests: DXA (gold standard), CT, MR imaging (clinical research)
Acromegaly	Hypertension in up to 30% of cases, along with diabetes, LVH, and OSA. Other symptoms: visual field defects, cranial nerve palsies, headache, mandibular overgrowth, enlargement of hands/feet, soft tissue hypertrophy, macroglossia, musculoskeletal complications	IGF-1, serum GH, and GH levels after oral glucose tolerance test. Localization: MR imaging (preferred) or CT of the sella turcica

ACTH: adrenocorticotropic hormone; AHI: apnea-hypopnea index; BMI: Body mass index; CT: computed tomography; DXA: dual-energy X-ray absorptiometry; eGFR: estimated glomerular filtration rate; GH: growth hormone; IGF-1: insulin-like growth factor 1; LVH: left ventricular hypertrophy; MR: magnetic resonance; OSA: obstructive sleep apnea; PTH: parathyroid hormone; RAAS: renin-angiotensin-aldosterone system; TSH: thyroid-stimulating hormone.

### 4.7. Cardiovascular Risk Calculation

Among the most widely used scores for CV risk stratification are SCORE2, developed by the European Society of Cardiology in collaboration with the European Society of Hypertension (ESH),^101^ and PREVENT, recently validated by the American Heart Association (AHA).^74^ These tools estimate individual CV risk and are easily accessible through digital calculators.

SCORE2, an evolution of the original SCORE risk model, incorporates variables such as age, sex, total cholesterol, SBP, smoking, and the presence of DM. It is adjusted according to the baseline CV risk of different European populations, accounting for regional disparities in CVD epidemiology.^101^ The PREVENT score, in addition to including traditional variables (age, SBP, cholesterol levels, smoking, and DM), incorporates metabolic and renal markers such as eGFR, ACR, and HbA1c. This reflects a more advanced approach to early identification of subclinical organ damage and elevated CV risk.^74^ The PREVENT calculator was developed using data from over 6 million individuals across diverse ethnicities, geographic regions, and socioeconomic backgrounds.^102^

This Guideline recommends the use of the PREVENT score, proposed by the AHA, as it is the only model to include criteria related to cardiorenal and metabolic syndromes. In addition, the PREVENT calculator estimates the risk of developing CVD over 10 and 30 years, for individuals aged 30 to 79 years, allowing for more accurate, long-term risk prediction – particularly important for younger patients.

The PREVENT calculator is available on the American Heart Association (AHA) website: https://professional.heart.org/en/guidelines-and-statements/prevent-calculator. Risk stratification using the PREVENT calculator to estimate 10-year atherosclerotic cardiovascular disease (ASCVD) risk is based on the following percentage cut-off points: low risk: <5.0%; borderline risk: 5.0% to <7.5%; intermediate risk: 7.5% to <20%; high risk: ≥20%.

Although the PREVENT score is based on data from a more diverse and mixed population compared to SCORE2, it is important to highlight the lack of Brazilian population data in these models, which may limit their accuracy in assessing CV risk in Brazil. In other words, internationally derived scores may underestimate risk by failing to account for the most prevalent or relevant risk factors in the local population.

Furthermore, access to the PREVENT calculator may not be universally feasible in some clinical settings. In such cases, risk analysis may be conducted using the criteria suggested in Chart 4.6, although the accuracy of this approach is limited and has not been validated.

**Quadro 4.6:** Classificação dos estágios de hipertensão arterial de acordo com o nível de pressão arterial, presença de fatores de risco cardiovascular, lesão de órgão-alvo ou doença cardiovascular estabelecida

	BP (mm Hg)			
CV risk factors, presence of HMOD or CVD	SBP 130-139 DBP 80-89	Stage 1 SBP 140-159 DBP 90-99	Stage 2 SBP 160-179 DBP 100-109	Stage 3 SBP ≥ 180 DBP ≥ 110
No CV risk factors	LOW RISK	LOW RISK	MODERATE RISK	HIGH RISK
1 or 2 CV risk factors	LOW RISK	MODERATE RISK	HIGH RISK	HIGH RISK
≥ 3 CV risk factors	MODERATE RISK	HIGH RISK	HIGH RISK	HIGH RISK
HMOD, stage 3 CKD, or DM	HIGH RISK	HIGH RISK	HIGH RISK	VERY HIGH RISK
Established CVD or stage ≥ 4 CKD	VERY HIGH RISK	VERY HIGH RISK	VERY HIGH RISK	VERY HIGH RISK

BP: blood pressure; CVD: cardiovascular disease; CKD: chronic kidney disease; DBP: diastolic blood pressure; DM: diabetes mellitus; SBP: systolic blood pressure; HMOD: hypertension-mediated organ damage. CV risk factors considered in this analysis: male sex; age: > 55 years for men and > 65 years for women; premature CVD in first-degree relatives (men < 55 years and women < 65); smoking; dyslipidemia: LDL-cholesterol ≥ 100 mg/dL and/or non-HDL cholesterol ≥ 130 mg/dL and/or HDL-cholesterol ≤ 40 mg/dL in men or ≤ 50 mg/dL in women and/or triglycerides > 150 mg/dL; obesity: body mass index ≥ 30 kg/m².

### 4.8. Additional Considerations and Challenges in Cardiovascular Risk Assessment in Hypertension

Several clinical conditions can influence CV risk stratification in patients with hypertension, with age being a particularly relevant factor. In the short term, older adults have a higher absolute risk, whereas younger individuals present a lower absolute risk – even when they have an unfavorable risk profile.^45^ However, in the long term or over the lifetime, this relationship inverts: younger individuals who develop risk factors early in life experience greater loss of longevity compared to those who develop them later.

A key limitation in CV risk assessment is the lack of consideration for the duration of exposure to hypertension and other risk factors. The length of time the patient has had hypertension, the duration of antihypertensive treatment, and the historical levels of BP over time can significantly influence risk estimation.^103^ Moreover, scores that use binary answers (yes or no) for clinical conditions such as DM and smoking to assess CV risk do not take into consideration the duration of these conditions. Patients with longer exposure are at higher CV risk than those with lower exposure to the same conditions.^45^ Therefore, incorporating new tools that account for relative risk vs lifetime risk, as well as periods of increased exposure, is important, particularly for younger individuals who may have low absolute risk but high relative risk for CVD.^102,104^ The concepts of "vascular age" (calculated by PWV) and "cardiometabolic age" may help improve this assessment.^105,106^

Additionally, out-of-office BP measurements obtained by ABPM and HBPM provide superior prognostic value compared to office BP readings.^107^ However, current risk calculators still do not incorporate ABPM or HBPM values, treatment duration, or longitudinal BP trends, which could improve risk prediction. Conversely, BP phenotypes derived from office and out-of-office measurements, such as MH, uMH, WCH, and uWCH, may have prognostic value and are useful in risk assessment and patient management.^108-110^

Compared to normotension, MH is associated with a worse CV prognosis, similar to that of sustained hypertension. Meanwhile, uMH has a worse prognosis than controlled hypertension, tending to resemble that of uncontrolled sustained hypertension.^108^ Therefore, it is recommended that patients with MH and uMH undergo routine CV risk stratification in the same manner as those with sustained or uncontrolled sustained hypertension, including the assessment of HMOD.

As for WCH, evidence suggests that this phenotype is associated with an intermediate CV risk (between normotension and sustained hypertension); however, the mechanisms underlying this increased risk remain unclear.^109^ Therefore, it is suggested that patients with WCH be regularly assessed for HMOD and CV risk. Conversely, uWCH does not appear to carry a higher risk of CV events compared to controlled hypertension, although some evidence suggests a greater prevalence of HMOD.^109,110^

The incorporation of novel markers into CV risk stratification may also support a more accurate and individualized approach in managing patients with hypertension. In this context, biomarkers such as lipoprotein(a),^111^ high-sensitivity troponin, and natriuretic peptides (NT-proBNP and BNP),^112^ along with advanced imaging techniques, including coronary artery calcium score,^113^ are emerging as additional tools for CV risk assessment.

Finally, it is important to emphasize that risk stratification in individuals with prehypertension is essential to guide the initiation of antihypertensive therapy and to better control CV risk factors. CV risk assessment in individuals with established hypertension allows for a more accurate and individualized approach to pharmacological therapy and goal setting for CV risk factor control.

Flowcharts for CV risk stratification in individuals with prehypertension and hypertension are presented in Figure 4.3.

**Figure 4.3:** Cardiovascular risk stratification in individuals with prehypertension and hypertension.

**Table:** 

Recommendations for clinical and complementary assessment and cardiovascular risk stratification	Strength of recommendation	Certainty of evidence
A thorough medical history and physical examination are recommended to improve diagnostic accuracy and help in the identification of secondary causes of hypertension and HMOD.	STRONG	LOW
CV risk factors and HMOD should ideally be assessed in all patients at the time of hypertension diagnosis and re-evaluated at least annually, with the choice of methods depending on available resources.	STRONG	LOW
It is recommended to assess for kidney disease and classify it according to the KDIGO 2024 guidelines.	STRONG	MODERATE
It is recommended to assess CV risk using the PREVENT score.	STRONG	HIGH
Stratification of CV risk in individuals with prehypertension is recommended to guide the initiation of antihypertensive therapy and optimize control of CV risk factors.	STRONG	HIGH
The stratification of CV risk in patients with hypertension is recommended to allow for a more accurate and individualized approach to pharmacological therapy and to set tailored goals for CV risk factor control.	STRONG	HIGH

CV: cardiovascular; KDIGO: Kidney Disease Improving Global Outcomes; PREVENT: Predicting Risk of Cardiovascular Disease Events; HMOD: hypertension-mediated organ damage.

## 5. Treatment Initiation and Therapeutic Targets

### 5.1. Treatment Initiation

It remains a matter of debate whether the decision to initiate treatment should be based solely on BP levels or on the associated CV risk as well. A meta-analysis published in 2021 by the Blood Pressure Lowering Treatment Trialists’ Collaboration^114^ suggests that a fixed degree of BP reduction is similarly effective for primary and secondary prevention of major CV events, even at BP levels considered "normal" and typically not targeted for pharmacological treatment. The higher the individual risk, the greater the benefit in terms of outcome reduction.

In addition, a 2018 meta-analysis by Karmali et al.^115^ compared a BP-lowering treatment strategy based on predicted CV risk with one based on SBP level. They found that the treatment strategy based on CV risk was more effective. However, even though this meta-analysis is of high quality and implies a high certainty of evidence, it is recommended that the decision to initiate treatment should incorporate both approaches.

Individuals with BP ≥ 120/80 mm Hg should adopt nonpharmacological measures (NPMs) to maintain BP < 130/80 mm Hg and reduce CV risk (see Chapter 6). For patients with BP ranging from 130-139 and/or 80-89 mm Hg, CV risk estimation is an important factor in guiding treatment initiation. A meta-analysis of 24 randomized controlled trials (RCTs) including 47,991 individuals investigated the impact of BP reduction on CV outcomes in individuals with BP in the prehypertensive range. It demonstrated that a BP reduction of 10/5 mm Hg significantly reduced the risk of stroke by 60% in patients at high and very high CV risk.^116^ However, the meta-analysis by Karmali et al. questions these protective effects.^115^

In patients with hypertension, CV risk stratification should not interfere with the decision to initiate treatment. The benefits of treating hypertensive patients without other associated risk factors and who present with persistently elevated BP levels are well established and it has been consistently recommended by national and international guidelines.^1,44,45,117^

Therefore, this Guideline draws on meta-analyses using individual participant data from RCTs involving individuals with stage 1 hypertension and no prior CVD to define the most appropriate management strategy.^118,119^ One of these studies showed that treating low-risk hypertensive patients did not reduce CAD outcomes, CV events, or CV mortality over a follow-up period of 4 to 5 years.^118^ However, there was a trend toward reduction of stroke and all-cause mortality, suggesting these benefits might become significant with longer follow-up and/or larger sample sizes.

The other study showed that treatment led to reductions in CVD and mortality when initial BP was ≥ 140/90 mm Hg.^119^ In patients with stage 1 hypertension, there was a significant reduction in mortality, although the decrease in major CV events was less pronounced. These findings were supported by results from a subgroup analysis of the Heart Outcomes Prevention Evaluation (HOPE)-3 trial.^114^ In that study, antihypertensive treatment in patients classified as stage 1 with intermediate CV risk led to a mean reduction of 6 mm Hg in SBP and a 27% reduction in the rate of combined CV events among those with office SBP greater than 143 mm Hg.^114^ Based on these data, pharmacological treatment should be initiated in conjunction with NPMs when office BP is ≥ 140/90 mm Hg.

Indications for initiating nonpharmacological and pharmacological treatment are presented in Table 5.1 and Figure 5.1.^114,116,118-120^

**Table 5.1:** Indications for nonpharmacological and pharmacological treatment according to BP and age

Treatment	Target population	Treatment initiation
NPMs	BP ≥ 120/80 mm Hg	At diagnosis
Pharmacological treatment	BP ≥ 140/90 mm Hg	At diagnosis
	BP 130-139/80-89 mm Hg and high CV risk	When BP remains uncontrolled after 3 months of NPMs
	Older adults ≥ 60 years	When SBP ≥ 140 mm Hg
	Older adults ≥ 80 years	When SBP ≥ 140 mm Hg*

BP: blood pressure; CV: cardiovascular NPMs: nonpharmacological measures; SBP: systolic blood pressure.

* Use caution and individualize the treatment strategy in patients with a history of orthostatic hypotension, moderate to severe frailty, or life expectancy under 3 years.^114,116,118-120^

**Figure 5.1:** Treatment initiation and BP targets.

### 5.2. Blood Pressure Targets

Regarding BP targets in patients with hypertension and low CV risk, both a meta-analysis and data from a large observational study suggest that achieving BP < 140/90 mm Hg is associated with significant reductions in the risk of mortality, stroke, and end-stage CKD.^121,122^ According to a systematic review and meta-analysis published by the SBC, this Guideline recommends a BP target of < 130/80 mm Hg, even in patients at low or intermediate risk (Figure 5.1).^123^

Regardless of BP level (whether within the prehypertensive range or at any stage of hypertension), the presence of CVD, DM or CKD, familial hypercholesterolemia, HMOD, or a 10-year risk estimate ≥ 20% according to the PREVENT risk score are all indicators of high or very high CV risk.^124^

The SPRINT trial, which involved patients without diabetes and with high CV risk, demonstrated that the mean SBP was 134.6 mm Hg in the standard treatment group and 121.5 mm Hg in the intensive treatment group over a follow-up period of 3.26 years. This resulted in lower rates of both fatal and non-fatal CV events and all-cause mortality.^125^ Some adverse events were more frequently observed in the intensive treatment group. Therefore, in general, the BP target for patients at high CV risk can be standardized to <130/80 mm Hg.^123^

The combined analysis of the SPRINT and ACCORD trials showed that the longer the duration spent within the controlled BP range, the lower the risk of adverse kidney and CV events,^46^ highlighting the importance of adherence to and persistence with treatment.

### 5.3. Blood Pressure Targets in Ambulatory and Home Blood Pressure Monitoring

It is important to consider that, ideally, BP should be measured both in the medical office and at home using ABPM or HBPM. Among the many advantages of office and out-of-office BP measurements, one of the most significant is the ability to identify different BP behavior phenotypes, which has implications for risk stratification and individualized treatment strategies, as well as for the use of ABPM- or HBPM-guided targets in some cases.^126^

For the WCH and MH phenotypes, it is always recommended to implement NPMs and, when indicated, pharmacological therapy. In these conditions, BP control targets should be guided by both office and out-of-office measurements, according to the reference ranges shown in Table 5.2.

**Table 5.2:** Blood pressure targets in ABPM (24-hour, daytime, and nighttime) and HBPM

	SBP (mm Hg)	DBP (mm Hg)
24-hour ABPM	< 130	< 80
Daytime ABPM	< 135	< 85
Nighttime ABPM	< 120	< 70
HBPM	< 130	< 80

ABPM: ambulatory blood pressure monitoring; DBP: diastolic blood pressure; HBPM: home blood pressure monitoring; SBP: systolic blood pressure.^126^

### 5.4. The J-Curve of Blood Pressure

In the treatment of hypertension, excessive BP reduction may be associated with an increased risk of CV events. This phenomenon suggests a J- or U-shaped relationship, where both very high and very low BP levels are linked to increased risks. In other words, there may be a threshold below which further BP reduction could actually increase the risk of adverse events.^127,128^

The J-curve is more frequently observed in relation to DBP, especially in patients with established CAD. The main hypothesis is that very low DBP levels may lead to reduced coronary perfusion. However, observational data do not consistently demonstrate the presence of a J-curve relationship between BP and CV risk.^127,128^

More recent observational studies suggest that the BP J-curve may not be a causal phenomenon and that several confounding factors could be involved.^128-130^ A nonlinear Mendelian randomization study found no evidence that lowering DBP increases the risk of CV events. In fact, it indicated that the risk of MI continues to decline with lower DBP values.^114^ In summary, there is no evidence that low BP is the cause of a high CV risk state; rather, it may simply be a marker of such risk, particularly among older adults and those with comorbidities.

Recent systematic reviews and meta-analyses that examined populations with pre-existing CVD and SBP reductions greater than 120 mm Hg found robust evidence suggesting additional benefits in reducing hard outcomes.^131^

### 5.5. Intensive Blood Pressure Reductions and Risk of Adverse Events

More intensive reductions in BP often raise concerns about the potential increase in serious adverse events, particularly among older adults and/or frail patients. A recent meta-analysis evaluating 20,895 older adults treated to achieve more stringent BP targets found a 29% reduction in major CV events, without an increase in serious adverse outcomes.^132^

These and other findings support the recommendation of more intensive BP reduction targets in individuals with hypertension, regardless of age. However, special attention should be given to frail patients, oldest-old adults (≥ 80 years), those with reduced life expectancy, or individuals with characteristics that may increase the risk of adverse events.^131,133^ If the < 130/80 mm Hg target is not tolerated, BP should be reduced to the lowest level tolerated by the patient.^117^

An important question explored in recent clinical trials is whether BP reductions below 120/70 mm Hg, particularly in patients with high CV risk, offer additional CV protection. A meta-analysis published in 2021 showed that for any initial BP range (even among individuals with baseline SBP below 120 mm Hg), a 5 mm Hg reduction was associated with an 11% decrease in CV outcomes, without a significant increase in the incidence of adverse events.^131^

### 5.6. Considerations in Isolated Systolic Hypertension

In patients with a wide pulse pressure (PP), SBP ≥ 140 mm Hg, and DBP < 90 mm Hg, evidence suggests that antihypertensive therapy should be initiated and intensified (as long as it is well tolerated) to reduce CV risk.^134,135^ In isolated systolic hypertension, the BP control target naturally focuses solely on SBP. In clinical practice, achieving this target may be limited by the concomitant and progressive reduction of DBP, which is already below 90 mm Hg and may fall below 60 mm Hg during treatment. However, there is no definitive evidence supporting a lower limit for DBP in this context, especially considering that these individuals are typically older and present with different comorbidities. As previously noted, a causal relationship between low DBP and increased CV risk has not been confirmed.^128^ Therefore, BP targets should always be pursued on an individualized basis, with closer monitoring, particularly in older adults with comorbidities.^134^

Some patients who achieve a more intensive BP target, with values below 120/70 mmHg, may be at increased risk of symptomatic hypotension, syncope, acute kidney injury, and hyperkalemia. Despite substantial heterogeneity in the defining metrics of these adverse effects, stricter BP control should be maintained in those with good tolerance to antihypertensive treatment.^136^

Current recommendations emphasize the need for individualized hypertension management, considering factors such as age, frailty, polypharmacy, and the presence of other health conditions.^136^

**Table:** 

Key messages on treatment initiation
The adoption of NPMs aims to reduce CV risk. All individuals with BP ≥ 120/80 mm Hg should initiate and maintain NPMs, even after starting pharmacological treatment when indicated.
CV risk estimation is important for individuals with BP 130-139 and/or 80-89 mm Hg; pharmacological treatment is indicated for those with high CV risk.
In patients with hypertension, the initiation of pharmacological treatment does not depend on CV risk stratification.

BP: blood pressure; CV: cardiovascular; NPMs: nonpharmacological measures.

**Table:** 

Recommendations for treatment initiation	Strength of recommendation	Certainty of evidence
NPMs are recommended for all individuals with BP ≥ 120/80 mm Hg.	STRONG	HIGH
Pharmacological treatment is recommended after 3 months of NPMs for individuals with BP 130-139/80-89 mm Hg and high CV risk.	STRONG	HIGH
Initiation of pharmacological treatment is recommended for individuals with BP ≥140/90 mm Hg.	STRONG	HIGH

BP: blood pressure; CV: cardiovascular; NPMs: non-pharmacological measures. * Use caution and individualize the treatment strategy in patients with a history of orthostatic hypotension, moderate to severe frailty, or life expectancy under 3 years.^114,116,118-121^

**Table:** 

Key messages on blood pressure targets
There is no consistent evidence supporting the existence of a J-curve relationship between BP and CV risk.
Patient tolerability to intensive BP reduction should be carefully considered and monitored, particularly in frail individuals, those aged ≥ 80 years, and patients with symptomatic orthostatic hypotension or reduced life expectancy.
If the < 130/80 mm Hg BP target is not tolerated, BP should be reduced to the lowest level tolerated.
In WCH and MH, assess BP control using ABPM or HBPM.
In isolated systolic hypertension, an SBP target < 130 mm Hg should be achieved, and DBP reduction should be monitored for potential adverse events.

ABPM: ambulatory blood pressure monitoring; BP: blood pressure; CV: cardiovascular; DBP: diastolic blood pressure; HBPM: home blood pressure monitoring; MH: masked hypertension; SBP: systolic blood pressure; WCH: white-coat hypertension.

**Table:** 

Recommendations for blood pressure targets	Strength of recommendation	Certainty of evidence
A BP target of < 130/80 mm Hg is recommended for patients with BP 130-139/80-89 mm Hg and high CV risk.	STRONG	HIGH
A BP target of < 130/80 mm Hg is recommended for patients with hypertension, regardless of low, moderate, or high CV risk.	STRONG	HIGH
For patients who do not tolerate the < 130/80 mm Hg BP target, BP should be reduced to the lowest level tolerated.	STRONG	MODERATE
BP target achievement should be confirmed with out-of-office BP measurement (ABPM or HBPM).	STRONG	LOW

ABPM: ambulatory blood pressure monitoring; BP: blood pressure; CV: cardiovascular; HBPM: home blood pressure monitoring.

## 6. Nonpharmacological Measures

This chapter explores several nonpharmacological interventions for health promotion, prevention, and BP control. It presents the latest evidence and grade of recommendation for each evidence in hypertension management. Given the broad scope and diversity of NPMs, studies on their effects on outcomes such as mortality and CV events are limited to a few approaches. Therefore, this chapter and its recommendations focus on the effects of NPMs on BP, with the effects on mortality being discussed only when specific evidence is available. The chapter's objective is to provide practical information, including effect size results for each NPM and recommendations for their implementation. Furthermore, new approaches with promising evidence of benefit are highlighted within each intervention. Finally, the chapter underscores the crucial role of a multidisciplinary team in the successful application of NPMs for hypertension management.

### 6.1. Smoking Cessation

Smoking, which includes the use of tobacco-derived products, hookah, and electronic cigarettes, is a major CV risk factor, so that quitting smoking has been associated with a reduction in overall and CV mortality.^137,138^ The relationship between smoking and BP remains controversial, with some studies showing no association between smoking levels and BP^139^ and others indicating an increase in BP with smoking onset^26^ and a reduction in the prevalence of hypertension in long-term former smokers.^140^ Given the reduction in overall CV risk, all health care professionals should recommend smoking cessation for their patients. Also, various strategies and medications are available to assist patients in adopting this behavior and should be used under expert guidance.^141^

### 6.2. Weight Loss

Hypertension is strongly related to overweight and obesity, and 60% to 77% of individuals with obesity have hypertension.^142,143^ Even without achieving the desired body weight, weight loss can reduce BP and CV risk proportionally to the magnitude and duration of weight loss.^143,144^ A meta-analysis showed that every 1 kg of weight loss resulted in a reduction of 1.05 mm Hg in SBP and 0.92 mm Hg in DBP.^145^ In patients with obesity, weight loss is associated with a 15% reduction in mortality.^143,146^ Therefore, for patients with hypertension who are overweight or obese, weight loss is an essential recommendation. In adults, the goal is to achieve and maintain a healthy body weight, represented by a BMI < 25 kg/m^2^,^1,1392^ and in older adults, a BMI between 22 and < 27 kg/m^2^.^1,139^

Beyond BMI, assessments should include central adiposity parameters such as WC, which should ideally be < 102 cm in men and < 88 cm in women.^1^ Maintaining a healthy weight involves adopting healthy habits, which can be complemented by medication or surgical procedures when necessary. Evidence suggests that bariatric surgery, combined with NPMs, is particularly effective in reducing hypertension and atherosclerotic CVD in severe obesity.^143,144^

### 6.3. Dietary Modifications

Consistent evidence indicates that some dietary modifications can lower BP and should be recommended for patients with hypertension (Table 6.1). These recommendations and the supporting evidence are presented below.

**Table 6.1:** Dietary measures for the prevention and treatment of hypertension

Modification	Goal
Restricted sodium intake	< 2 g/day = 5 g of salt/day = 1 teaspoon/day^11^
Increased dietary potassium intake	≥ 3.5 g/day^18^*
Reduced alcohol consumption	Limit alcohol consumption to: Men – 2 drinks**/day Women – 1 drink**/day^14^ Binge drinking should be avoided^14^
Adopting a healthy eating pattern	Preferably adopt the DASH diet^14,23^

DASH: Dietary Approaches to Stop Hypertension.

* Except for patients with chronic kidney disease or hyperkalemia.

** There are different definitions for the amount of alcohol present in 1 drink,^17^ ranging from a mean of 10 to 15 g. Recent recommendations for hypertension define 1 drink as containing 10 to 12 g of alcohol,^14^ corresponding to approximately 200 mL of beer (5% alcohol), 100 mL of wine (12% alcohol), and 25 mL of liquors, such as whiskey, vodka, and brandy (40% alcohol).

#### 6.3.1 Restricted Sodium Intake

High sodium intake is associated with increased BP and may contribute to approximately 30% of the prevalence of hypertension.^147^ Reducing sodium intake leads to a proportional decrease in BP, which is more pronounced in individuals with hypertension, Black people, and older adults.^148^ A daily reduction of 1.15 g of sodium can lower SBP by 2.8 mm Hg and DBP by 1.4 mm Hg in patients with hypertension.^148^ Therefore, it is recommended to limit sodium intake to a maximum of 2 g per day, which is equivalent to 5 g of salt or 1 teaspoon/day.^149^

However, current global sodium intake, including Brazil, is typically double this recommended amount.^149^ Fresh foods contribute a very small portion to our sodium intake. In high-income countries, 70% to 80% of consumed sodium comes from processed and ultra-processed foods or fast-food restaurants. In low- and middle-income countries, the primary sources of sodium are salt added during cooking or at the table, as well as the use of high-sodium condiments. The expansion of the food industry has increased the exposure to processed foods in these countries.^147,149^ Strategies for reducing sodium intake include increasing public awareness, reformulating processed foods, incorporating herbs and spices as seasonings, and using salt substitutes in which some of the sodium is replaced by potassium.^149,150^ These salt substitutes can lower SBP by 5.9 mm Hg and DBP by 2.2 mm Hg in patients with hypertension and may decrease the risk of CV events, as well as CV and all-cause mortality.^24^ However, potassium-enriched salt substitutes may elevate serum potassium levels in individuals at risk for hyperkalemia, such as patients with CKD.^151^ Low-sodium or ‘light’ salt is an option of salt substitute, offering at least a 50% reduction in sodium compared to regular table salt.^152^ Another option of low-sodium salt substitute is herbal salt, which combines table salt with dried herbs such as rosemary, basil, oregano, and parsley. However, other types of salt, such as Himalayan pink salt and sea salt, contain the same sodium content as table salt.^153,154^

#### 6.3.2. Increased Dietary Potassium Intake

Increased potassium intake can lower SBP by 4.8 mm Hg and DBP by 3.0 mm Hg in patients with hypertension, and this effect is more pronounced when sodium intake is high.^150,155^ Although most studies have used potassium chloride capsules, dietary modifications can achieve a similar BP response.

Since high-potassium diets reflect healthy eating patterns, they are the recommended approach to increase this mineral,^156^ aiming at an intake of at least 3.5 g/day.^156^ Good sources of potassium include fruits, vegetables, greens, nuts, legumes, and low-fat dairy products.^151^ Four to five servings of fruit (1 serving equals 1 medium fruit or 1/2 cup of fresh cut fruit) and vegetables (1 serving equals 1 cup of raw leafy greens or 1/2 cup of cooked vegetables) typically provide 1.5 to 3.0 g of potassium.^45,157-159^

#### 6.3.3. Reduced Alcohol Consumption

Alcohol consumption is directly associated with BP and the incidence of hypertension, especially when intake exceeds 2 drinks per day (10 to 12 g/day).^150,151,160^ While reducing alcohol intake may not lower BP in individuals who consume up to 2 drinks per day, it does reduce BP in those who consume 3 or more drinks daily. This effect is even more pronounced in those who consume 6 or more drinks per day and reduce their intake by approximately 50%, leading to a reduction of 5.5 mm Hg in SBP and 4.0 mm Hg in DBP.^161^

Although light-to-moderate alcohol consumption has previously been associated with a cardioprotective effect, recent evidence indicates that this association may be due to confounding factors. In fact, alcohol intake is related to a higher risk of CVD, with an exponential increase as consumption levels rise.^25^ This means that even light alcohol intake can be harmful to health, with the greatest risks associated with heavy consumption, whether chronic or episodic.^162^ Therefore, for individuals who consume alcoholic beverages, reducing or moderating their intake is recommended, along with total avoidance for a few days per week to help control BP.^45,150,161^

#### 6.3.4. Adopting a Healthy Eating Pattern

The assessment of dietary patterns, defined as the combination of all foods typically consumed, allows for the joint study of nutrients and foods, as well as their synergistic effects. These patterns include the Dietary Approaches to Stop Hypertension (DASH) diet, which emphasizes the consumption of fruits, vegetables, low-fat dairy products, whole grains, lean meats, and nuts, while limiting the intake of saturated fats, fatty meats, refined grains, and added sugars. This diet is rich in potassium, calcium, magnesium, and fiber, with reduced amounts of sodium, cholesterol, total fat, and saturated fat.^163^ The DASH diet can reduce SBP/DBP by 8.7/4.5 mm Hg in patients with hypertension,^164^ with even greater reductions when combined with additional sodium restriction.^163^ Another dietary pattern that has an effect on BP is the Mediterranean diet, which is rich in fruits, vegetables, and whole grains and low in red meat. It is characterized by the consumption of fish, nuts, generous amounts of olive oil, and moderate amounts of red wine.^165^ This diet promotes a modest reduction in SBP/DBP of 1.5/0.9 mm Hg.^165^ Therefore, adopting healthy eating patterns, such as the DASH or Mediterranean diet, is recommended in hypertension. The beneficial effects of the DASH diet and other healthy diets depend on long-term adherence, which is often challenging.^151,166,167^ Barriers to adherence include lack of motivation and food costs.

#### 6.3.5. Other Dietary Interventions

Other dietary components may also affect BP, but current evidence is neither conclusive nor robust. Some of these components are discussed below.

- Coffee and caffeinated beverages: Recent studies suggest that habitual moderate coffee consumption does not raise BP or increase the risk of hypertension.^168^ Therefore, moderate coffee consumption is recommended, limiting intake to 3-4 cups per day.^151^ Higher amounts should be avoided due to the high caffeine content, which averages 80 mg/200 mL of filtered coffee. Caffeine can cause an acute increase in BP, especially in those not accustomed to it–intake of up to 400 mg/day is considered safe for healthy adults.^169^ Therefore, consumption of other caffeine-rich beverages, such as energy drinks, should also be monitored.
- Probiotics: Evidence has recently emerged suggesting that probiotic supplementation may produce modest BP reductions in patients with hypertension.^170^
- Vitamin D supplementation: While there is no consensus, vitamin D supplementation appears to reduce BP, particularly in patients with hypertension experiencing hypovitaminosis D.^171^

### 6.4. Physical Activity and Exercise

Physical activity (PA) encompasses any bodily movement that increases energy expenditure above rest, such as locomotion and occupational, household, and leisure activities. Exercise, in turn, refers to structured PA performed with specific goals, such as improving health and/or physical fitness. Sedentary behavior refers to time spent in low-energy expenditure activities (≤ 1.5 metabolic equivalent of task [MET]), such as sitting, reclining, or lying down (eg, watching TV, using a computer, playing video games).^172^ Every hour spent in sedentary behavior is estimated to increase CV risk by 5%. Therefore, it is recommended to interrupt periods of sedentary behavior with light PA.^173^

Regular PA can reduce SBP by 2 to 5 mm Hg and DBP by 1 to 4 mm Hg, and these reductions are more pronounced in individuals with higher initial BP levels.^174^ Leisure-time PA, in particular, can decrease the incidence of hypertension by up to 19% and, in patients with hypertension, it can reduce the risk of mortality by approximately 30%.^174,175^ Therefore, regular PA is recommended for both the prevention and treatment of hypertension.^173,174^

In patients with hypertension, structured exercise offers the most substantial benefits. Aerobic training, dynamic resistance training, isometric resistance training, and combined aerobic and dynamic resistance training can lower in-office BP (Table 6.2).^176^ However, only aerobic training can reduce ambulatory BP.^177^ Therefore, aerobic training is recommended for patients with hypertension, and it can be combined with dynamic resistance training to achieve additional health benefits.

**Table 6.2:** Reduction in systolic and diastolic blood pressure with different exercise training types

Training	In-office SBP/DBP (mm Hg)^33^	24-hour SBP/DBP (mm Hg)^34^
Aerobic	-7.6/-4.7	-5.5/-3.8
Dynamic resistance	-2.6/-2.1	---
Isometric resistance	-4.3/-5.0	---
Combined	-5.3/-5.6	---

---: no proven effect; SBP: systolic blood pressure; DBP: diastolic blood pressure.

While other exercise modalities, such as water-based exercise, alternative neuromotor practices (eg, yoga, tai chi, Pilates), and high-intensity interval training, can reduce in-office BP in patients with hypertension, current research supporting these effects is scarce and of limited quality, with no evidence of ambulatory BP reduction or assessment of potential risks.^174^ Therefore, these practices are not recommended as treatments for hypertension, but they can be used in specific situations.

Table 6.3 shows PA and exercise recommendations for patients with hypertension. Some precautions should be taken before and during PA and exercise.^172,178^ Prescribing light-to-moderate PA for individuals without pre-existing heart, kidney, or cerebrovascular disease does not require a prior medical evaluation. If any symptoms arise during exercise, the activity should be discontinued and a physician consulted. Individuals with hypertension who have comorbidities, CV symptoms (eg, angina, fatigue, dyspnea, dizziness, edema), multiple risk factors, or who intend to engage in high-intensity or competitive activities should undergo prior medical evaluation. Exercise stress testing is recommended to assess physical fitness and prescribe training, as it allows for the assessment of BP response to exertion and suspected CAD in symptomatic individuals or those with multiple risk factors. A training session should not be started if BP is above 160/105 mm Hg. If BP rises above 180/105 mm Hg during aerobic exercise, the intensity of the activity should be reduced.^172,179-182^

**Table 6.3:** Guidelines for prescribing physical activity and exercise

**Interrupting sedentary behavior**		
- Stand up and move around for 5 min for every 30 min sitting down.		
**Physical activity**		
- Aim for at least 150 min/week of moderate aerobic PA or 75 min/week of vigorous aerobic PA, or an equivalent combination of both moderate and vigorous physical activity. - For maximum benefit, include muscle-strengthening exercises twice/week.		
**Exercise training**		
- Aerobic complemented by resistance		
**Aerobic training – mandatory**		
- Modalities: walking, running, dancing, and swimming, among others.		
- Volume: accumulate at least 150 min/week. - Frequency: 3 to 5 times per week (more often – increased effect). - Duration: 30 to 60 min per session (longer – increased effect).		
- Moderate intensity defined as:		
	1) Highest intensity while maintaining the ability to carry on a conversation (without getting out of breath)	
	2) Feeling "slightly tired" (a rating of 12-13 on the 20-point Borg scale)	
	3) Maintaining training heart rate (HR) within the range calculated as follows:	
		Training HR = (maximal HR – resting HR) × % + resting HR
Where: Maximal HR: obtained from a maximal exercise stress test performed while taking regular medications or by calculating age-predicted maximal HR (ie, 220 – age). This formula is not suitable for patients with hypertension who have heart disease or are taking beta-blockers or non-dihydropyridine calcium channel blockers. Resting HR: measured after 5 min of lying down. %: use 40% as the lower limit and 60% as the upper limit.		
**Resistance training – complementary**		
- Frequency: 2 to 3 times per week.		
- 8 to 10 exercises for the main muscle groups, prioritizing unilateral exercises when possible.		
- 1 to 3 sets.		
- 10 to 15 repetitions to moderate fatigue (where movement speed is reduced) – approximately 60% of 1-repetition maximum (1RM).		
- Long passive rest periods – 90 to 120 s.		

PA: physical activity. Adapted from references.^1,172,173,178^

### 6.5. Approach to Psycho-Emotional Aspects

#### 6.5.1. Stress Management Techniques

Mental stress is associated with an increased risk of hypertension and is more common in hypertensive patients than in normotensive individuals.^18^ While usual stress reduction interventions can lower SBP and DBP, they have a lower certainty of evidence than other lifestyle modification interventions.^164^ In general, behavioral therapies for stress management have not shown robust evidence of efficacy in reducing BP, except for meditation.^164^ A meta-analysis showed that mindfulness-based stress reduction programs, consisting of at least 8 intensive meditation sessions, led to a reduction of 6.6 mm Hg in SBP and 2.5 mm Hg in DBP.^183^ Furthermore, transcendental meditation reduced SBP by 5.6 mm Hg and DBP by 2.9 mm Hg, while other meditation techniques showed reductions of 5.1 mm Hg in SBP and 2.6 mm Hg in DBP.^184-186^

#### 6.5.2. Slow Breathing

Slow or guided breathing, which involves reducing the respiratory rate to 6-10 breaths/minutes for 15-20 minutes daily, has been shown to promote a 6.4 mm Hg reduction in both SBP and DBP compared to natural breathing.^187^ In patients with hypertension and CVD, voluntary slow breathing exercises also reduce SBP and DBP.^187^ Regarding device-guided breathing (Resperate device®), while a meta-analysis initially indicated a reduction in BP, this effect disappeared when studies sponsored by the device manufacturer were excluded.^188^ Therefore, slow breathing is recommended as a low-risk adjunctive therapy for hypertension.

#### 6.5.3. Spirituality and Religiosity

Spirituality is defined as a set of moral, mental, and emotional values that guide an individual's thoughts, behaviors, and attitudes in both intrapersonal and interpersonal relationships. Spirituality can be measured and is distinct from religiosity, which refers to the extent to which an individual believes, follows, and practices a particular religion, which, in turn, is defined as a multidimensional construct that includes beliefs, behaviors, dogmas, rituals, and ceremonies that can be performed or practiced in private or public contexts.^189^

Cultivating spiritual well-being and uplifting feelings has been shown to positively impact CV health, leading to reduced CV risk and a lower prevalence of hypertension.^190^ Recently, the FEEL trial demonstrated a 7.6 mm Hg reduction in SBP through the encouragement of feelings such as forgiveness, gratitude, optimism, and purpose in life.^191^ Therefore, it is recommended that health care professionals be trained in addressing aspects of spirituality and religiosity with respect, without interfering with patients’ personal beliefs.^192^

### 6.6. Multidisciplinary Team in the Adoption of Nonpharmacological Measures

Studies have demonstrated that a multidisciplinary approach can improve BP control, leading to better management of CV risk factors and reduction in CV morbidity and mortality.^193^ Some descriptive studies indicate that team-based care and shared decision-making, with active patient participation, are associated with reduced costs and improved outcomes in hypertension management.^194^ A multidisciplinary approach was successfully employed in primary, secondary, and tertiary care settings, resulting in a hypertension control rate of 68%.^195^ Therefore, comprehensive multidisciplinary care is recommended for both the prevention and treatment of hypertension. The team should include professionals from diverse backgrounds (primary care physicians, cardiologists, endocrinologists, nephrologists, geriatricians, nurses, pharmacists, dietitians, physical education professionals, psychologists, social workers, and community health workers, among others), as they complement each other, integrating their skills, abilities, and knowledge while sharing responsibilities and decision-making.^196^ While patients remain central to this process, each actor should be empowered to play a leading role in order to develop a field of research in services and promote professional development. Educational and therapeutic actions should be extended to all patients, their families, and the community, always considering sociocultural, local, and regional particularities. Modern strategies, such as social media, distance learning, and digital educational resources, can facilitate the adoption of and adherence to NPMs for hypertension management.^197^

### 6.7. Final Considerations on Nonpharmacological Measures

NPMs encompass several approaches supported by different levels of evidence and recommendations for the prevention and treatment of hypertension. Figure 6.1 summarizes the key recommendations that, in addition to their effect on hypertension, offer general health benefits. Therefore, unless otherwise specified in the tables, all health professionals should recommend them to all adults with BP ≥ 120/80 mm Hg, especially those with hypertension.

**Figure 6.1:** Nonpharmacological measures for the prevention and treatment of hypertension.

**Table:** 

Recommendations for nonpharmacological measures in blood pressure management	Strength of recommendation	Certainty of evidence
Smoking cessation is recommended to reduce cardiovascular events and mortality.	STRONG	HIGH
Reducing body weight is recommended for BP and mortality reduction in patients with obesity.	STRONG	HIGH
Reducing sodium intake and increasing dietary potassium intake (except for patients with CKD) is recommended for BP reduction.	STRONG	HIGH
Limiting daily maximum alcohol consumption is recommended for BP reduction.	STRONG	MODERATE
The DASH diet and regular moderate physical activity are recommended for BP and mortality reduction.	STRONG	HIGH
Aerobic training is recommended for BP reduction.	STRONG	HIGH
Meditation is recommended for BP reduction.	STRONG	MODERATE
Slow breathing is recommended for BP reduction.	NEUTRAL	LOW
Addressing aspects of spirituality and religiosity is recommended for hypertension management.	NEUTRAL	LOW
A multidisciplinary approach is recommended for improved BP control.	STRONG	LOW

DASH: Dietary Approaches to Stop Hypertension; CKD: chronic kidney disease; BP: blood pressure.

## 7. Pharmacological Treatment

### 7.1. Treatment Targets and Rationale for Medication Use

Reducing morbidity and mortality is the primary goal of antihypertensive treatment. BP reduction is the initial target, with the broader goal of decreasing CV and renal outcomes, as well as hypertension-associated mortality, regardless of patient age.^114,198-200^

A high-quality meta-analysis from the Blood Pressure Lowering Treatment Trialists’ Collaboration analyzed individual data from approximately 350,000 participants from 48 RCTs to assess the effects of BP-lowering treatment on CV outcomes. Over an average 4 years of follow-up, a 5 mm Hg reduction in SBP lowered the relative risk of major CV events by 10%. Specifically, the risks of stroke, HF, CAD, and death from CV disease were reduced by 13%, 13%, 8%, and 5%, respectively. The benefits were greater among individuals with higher CV risk and those with greater BP reductions.^114^

#### 7.1.1. Heart Failure

With hypertension treatment, the risk of developing HF is reduced by approximately 30% in younger adults, 50% in older adults, and nearly 80% in older adults with a history of MI. In addition, BP control reduces the risk of recurrent HF by approximately 50%.^2021,202^

#### 7.1.2. Stroke

Antihypertensive therapy significantly reduces the risk of stroke in both primary and secondary prevention. A meta-analysis demonstrated that a 10 mm Hg reduction in SBP is associated with a 33% reduction in the risk of stroke.^203^ In addition, findings from the HOPE-3 study showed that combining antihypertensive therapy with statins significantly reduced the risk of ischemic stroke by 59% in individuals with intermediate CV risk.^204^ Similar benefits are seen in secondary prevention. A meta-analysis of RCTs found that antihypertensive treatment reduced the risk of recurrent stroke by 27%.^205^

#### 7.1.3. Coronary Artery Disease

A 10 mm Hg reduction in SBP is associated with a 17% decrease in the risk of CAD.^120^ Achieving BP targets in patients with hypertension can lead to a 40% to 50% reduction in the risk of death from CAD among middle-aged individuals. However, it is important to note that treatment efficacy may vary depending on factors such as the presence of comorbidities and the classes of antihypertensive medications used.^120,206^

#### 7.1.4. Chronic Kidney Disease

According to the FROM-J study, achieving an SBP level of < 130 mm Hg is associated with a 42% reduction in the risk of renal outcomes in patients with baseline SBP ≥ 130 mm Hg.^207^ Furthermore, the ARIC study suggests that even modest population-wide reductions in SBP may prevent new cases of CKD, with an estimated reduction of 11.7 to 13.4 CKD events per 100,000 person-years for every 1 mm Hg decrease in SBP.^208^

#### 7.1.5. Cardiovascular Death

The meta-analysis by Ettehad et al. showed that a 10 mm Hg reduction in SBP results in a 13% reduction in all-cause mortality, which includes CV death.^120^

A summary of the benefits of pharmacological treatment of hypertension is illustrated in Figure 7.1.^114,120,198-208^

**Figure 7.1:** Benefits of pharmacological treatment of hypertension.^114,120,198-208^

### 7.2. Pharmacological Treatment

The pharmacological treatment of hypertension is guided by recommendations based on specific classes of medications, primarily supported by robust evidence from RCTs demonstrating their effectiveness in lowering BP and reducing the risk of CV and renal events.^1,117,209^

The most commonly recommended classes include thiazide or thiazide-like diuretics (DIUs), angiotensin-converting enzyme inhibitors (ACEIs), angiotensin II receptor blockers (ARBs), and calcium channel blockers (CCBs). There is ongoing debate about whether beta-blockers (BBs) should be included among the preferred first-line agents.^45,117,150,210^ For this reason, a systematic review with meta-analysis was specifically conducted for this Guideline to provide an evidence-based answer, which will be discussed later in this chapter. Conversely, the use of BBs is well established in clinical conditions such as coronary syndromes (post-MI and angina),^211^ atrial fibrillation (AF), HF with reduced ejection fraction (HFrEF), heart rate (HR) control, in women who are pregnant or planning pregnancy,^212^ and in patients with CKD on hemodialysis.^213^

Despite the recognized benefits of antihypertensive treatment in reducing CV and renal morbidity and mortality, significant global disparities remain between high- and low-income countries regarding diagnosis, treatment, and BP control.^214^ There is still much to plan and implement in order to achieve better health outcomes.

### 7.3. General Principles of Pharmacological Treatment

Most patients with hypertension will require pharmacological treatment in addition to NPMs to achieve the recommended BP targets (see Chapter 5).

Chart 7.1 shows the five main classes of antihypertensive medications that have demonstrated BP-lowering effects compared to placebo, along with substantial benefits in reducing fatal and nonfatal CV and renal outcomes. These benefits are primarily attributed to BP reduction.^215-219^

**Chart 7.1:** Main classes of antihypertensive medications used in the treatment of hypertension^215-219^

Diuretics
Calcium channel blockers
Angiotensin-converting enzyme inhibitors
Angiotensin II receptor blockers
Beta-blockers, with specific indications

Specific information about each class of medication is detailed in Chart 7.2 to 7.6.

**Chart 7.2:** Diuretics: usual daily doses (mg/day), frequency (doses/day), and additional information

DIURETICS
Diuretics inhibit sodium reabsorption at different sites in the nephron, leading to natriuresis. In the long term, they reduce peripheral resistance. They may be combined synergistically with other antihypertensive agents.

Class and medication	Usual daily dose (mg)	Frequency (doses/day)	Additional information
**THIAZIDE AND THIAZIDE-LIKE DIURETICS**			
Hydrochlorothiazide	25-50	1	- Acts on the distal convoluted tubule - Higher doses increase diuretic effect but also metabolic side effects, without additional antihypertensive benefit - Contraindicated in active gout
Chlorthalidone	12.5-25	1	
Indapamide (slow release)	1.5-3.0	1	
**LOOP DIURETICS**			
Furosemide	20-240	1 to 3	- Acts on the thick ascending limb of the loop of Henle - Used in CKD, HF, and edematous states (sodium and fluid retention)
**POTASSIUM-SPARING DIURETICS**			
Spironolactone	25-100	1 to 2	- Non-selective steroidal mineralocorticoid receptor antagonist - Used in HF, hyperaldosteronism, resistant/refractory hypertension (4th-line agent), hypertension with comorbidities, reduces proteinuria - May cause hyperkalemia, particularly in CKD and when combined with ACEI or ARB - Side effects: gynecomastia, decreased libido, impotence
Eplerenone	25-100	1	- Selective non-steroidal mineralocorticoid receptor antagonist - More selective for aldosterone receptors (↓ adverse effects) - Similar hyperkalemia risk to spironolactone - Same indications as spironolactone
Amiloride	2.5-5	1	- Acts on sodium channels in the distal and collecting tubules - Available in combination with thiazide or thiazide-like diuretics

CKD: chronic kidney disease; HF: heart failure; ACEI: angiotensin-converting enzyme inhibitor; ARB: angiotensin II receptor blocker.

**Chart 7.3:** Calcium channel blockers: usual daily doses (mg/day), frequency (doses/day), and additional information

CCBs			
These agents block slow calcium channels, reducing calcium influx into vascular smooth cells and vascular resistance. All CCBs have demonstrated similar BP-lowering effects at equipotent doses. They may be combined synergistically with other antihypertensive agents.			

Class and medication	Usual daily dose (mg)	Frequency (doses/day)	Additional information
**Dihydropyridine CCBs**			
Amlodipine	2,5 – 10	1	- Avoid in patients with HFrEF and tachyarrhythmias - May cause headache, facial flushing, and peripheral edema - Edema is dose-dependent and improves with RAAS blockers but does not respond well to diuretics
Felodipine	2,5 – 10	1	
Nifedipine	10 – 60	1 – 3	
Nitrendipine	10 – 30	1	
Manidipine	10 – 20	1	
Lacidipine	2 – 6	1	
Lercanidipine	10 – 20	1	
Levamlodipine	2,5 – 5	1	
**Non-dihydropyridine CCBs** Currently rarely used in hypertension treatment			
Phenylalkylamine (verapamil)	120 – 360	1 a 2	- Avoid in patients with HFrEF - Avoid combining with BBs or using in patients with bradycardia (HR <50 bpm)
Benzothiazepine (diltiazem)	80 – 240	1 a 2	- Same precautions regarding bradycardia and combination with BBs

BB: beta-blocker; CCB: calcium channel blocker; HFrEF: heart failure with reduced ejection fraction; RAAS: renin-angiotensin-aldosterone system.

**Chart 7.4:** Angiotensin-converting enzyme inhibitors: usual daily doses (mg/day), frequency (doses/day), and additional information

Class and medication	Usual daily dose (mg)	Frequency (doses/day)	Additional information and recommendations
**ACEIs**			
These agents block ACE formation, reducing the production of angiotensin II and the degradation of bradykinin. They have anti-atherosclerotic and anti-inflammatory properties. ACEIs are effective in HFrEF and post-myocardial infarction, and help slow the progression of CKD, reduce proteinuria, and left ventricular hypertrophy. They are synergistic with other antihypertensive agents.			
Captopril	25 – 150	2 to 3	- Usually well tolerated - Avoid in women of childbearing age and contraindicated in pregnancy - Contraindicated in combination with ARBs - Can be used with spironolactone in HF and CKD with proteinuria - Contraindicated in bilateral renal artery stenosis or solitary kidney - Risk of hyperkalemia (especially in advanced CKD or with potassium supplements/spironolactone) - May worsen renal function initially, with later stabilization - Cough is a common side effect (due to bradykinin accumulation)
Enalapril	5 – 40	1 to 2	
Benazepril	10 – 40	1 to 2	
Lisinopril	10 – 40	1	
Fosinopril	10 – 40	1	
Ramipril	2,5 – 20	1 to 2	
Perindopril	4 – 16	1	

ACE: angiotensin-converting enzyme; ARB: angiotensin II receptor blocker; CKD: chronic kidney disease; HF: heart failure; HFrEF: heart failure with reduced ejection fraction; LVH: left ventricular hypertrophy; MI: myocardial infarction; RAAS: renin-angiotensin-aldosterone system.

**Chart 7.5:** Angiotensin II receptor blockers: usual daily doses (mg/day), frequency (doses/day), and additional information

**ARBs** These agents block the action of angiotensin II by antagonizing the angiotensin type 1 receptor. They have anti-atherosclerotic and anti-inflammatory properties and are effective in HFrEF, post-myocardial infarction, and in slowing the progression of CKD, proteinuria, and left ventricular hypertrophy. They are synergistic with other antihypertensive agents.			
**Medication**	**Usual daily dose (mg)**	**Frequency (doses/day)**	**Additional Information and recommendations**
Losartan	50 – 100	1 to 2	Same recommendations and side effects as ACEIs, except for cough (less common)
Valsartan	80 – 320	1	
Irbesartan	150 – 300	1	
Candesartan	8 – 32	1	
Olmesartan	20 – 40	1	
Telmisartan	20 – 80	1	

ACEI: angiotensin-converting enzyme inhibitor; CKD: chronic kidney disease; HFrEF: heart failure with reduced ejection fraction; LVH: left ventricular hypertrophy; MI: myocardial infarction.

**Chart 7.6:** Beta-blockers: usual daily doses (mg/day), frequency (doses/day), and additional information

Class and medication	Usual daily dose (mg)	Frequency (doses/day)	Additional Information and recommendations
**BBs**			
Indications: HFrEF, acute coronary syndromes (post-myocardial infarction and in patients with angina), atrial fibrillation. Also used in hypertension associated with arrhythmias, essential tremor, mitral valve prolapse, migraine, anxiety, portal hypertension, hyperkinetic syndromes (eg, thyrotoxicosis), and obstructive sleep apnea. Contraindications: severe or decompensated bronchial disease (if essential, use cardioselective or vasodilating BBs with caution); sinoatrial or atrioventricular block; bradycardia (HR <50 bpm); peripheral artery disease (except for vasodilating BBs).			
**1^st^ GENERATION, NON-CARDIOSELECTIVE**			
Propranolol	80-320	2 to 3	- Block both β1 (mainly in myocardium) and β2 (smooth muscle, lungs, blood vessels) receptors - More pronounced peripheral effects such as increased peripheral vascular resistance and bronchoconstriction - Pindolol has intrinsic sympathomimetic activity, resulting in less bradycardia
Nadolol	40-160	1	
Pindolol	10-60	1	
**2^nd^ GENERATION, CARDIOSELECTIVE**			
Atenolol	50-100	1 to 2	- Selectively block β1-adrenergic receptors, mostly in the heart, nervous system, and kidneys, avoiding undesirable peripheral blocking effects - High doses may affect β2 receptors
Metoprolol	50-200	1	
Bisoprolol	5-20	1	
**3^rd^ GENERATION**			
Nebivolol	2.5–10	1	- Cardioselective - Vasodilating action via increased nitric oxide availability
Carvedilol	6.25-50	1 to 2	- Non-cardioselective - Combined alpha- and beta-blocker effects, causes less bradycardia, and increases vasodilation - Indicated in HFrEF

BB: beta-blocker; HFrEF: heart failure with reduced ejection fraction; HR: heart rate; MI: myocardial infarction.

Other classes – such as non-steroidal mineralocorticoid receptor antagonists (MRAs), alpha blockers, central and peripheral sympatholytic agents, and direct-acting vasodilators – have not been widely studied in RCTs and are associated with higher rates of adverse events. These agents should be reserved for cases where BP remains uncontrolled despite the use of combinations from the main recommended classes (Chart 7.1).

Desirable characteristics of an antihypertensive medication include the ability to reduce CV and renal morbidity and mortality; oral efficacy and good tolerability; long-acting properties allowing for once-daily dosing; pharmacologic synergy with other classes, allowing combination therapy; and quality-controlled manufacturing – compounded formulations should not be used.^1^

In addition to considering the characteristics of medications, it is also important to:

- Adjust treatment, usually after 4 weeks of use, except in specific situations.
- Educate patients and caregivers that adherence is critical to successful BP control.
- Be prepared to adjust dosages, switch medications, or add agents as needed.
- Inform patients about possible adverse effects and how to manage them.

Antihypertensive medications, whether in monotherapy or combination, can be administered in the morning or at night, with similar efficacy.^220^

### 7.4. Classes of Medications Used in the Treatment of Hypertension

Figure 7.2 summarizes the "octet" of hypertension treatment, with the preferred classes of medications forming the base of the pyramid and the less commonly used medications (those with limited supporting evidence) positioned at the top.

**Figure 7.2:** Hypertension treatment octet Adapted from Feitosa et al.^221^

The inclusion of BBs at the base of the hypertension treatment octagon, with specific indications, was based on a systematic review with meta-analysis developed by the SBC, aimed at resolving ongoing controversies among international guidelines.^117,219,221^

Seven RCTs meeting the inclusion criteria were analyzed to assess the efficacy and safety of atenolol for the treatment of primary hypertension in comparison with other well-established, effective, and well-tolerated antihypertensive medications.^211^

The PICO question focused specifically on atenolol, as it is the only BB with eligible RCT data, and thus the recommendation cannot be extrapolated to other BBs not evaluated in the analysis.^211^

When compared with amlodipine and losartan, atenolol was associated with a slightly higher incidence of CV events, with the certainty of evidence rated as low and moderate, respectively.^211^

In the same analysis, the hydrochlorothiazide (HCTZ) + amiloride combination showed the greatest reduction in CV events, although the certainty of this evidence was very low. However, BP reduction was similar across all treatment comparisons, with small differences in CV outcome reduction slightly favoring thiazide DIUs, amlodipine, and losartan. It is important to note that this is a weak recommendation, with low or very low certainty of evidence, except for the atenolol vs. losartan comparison, which had moderate certainty (for outcomes with effect differences).^211^

Based on these findings, atenolol may be considered an option for treating primary hypertension in combination with first-line agents (ie, low-dose thiazide/thiazide-like DIUs, RAAS blockers, and CCBs). This choice should be individualized based on best clinical indications, comorbidities, patient characteristics, and preferences.^211^

Charts 7.2 to 7.7 present a summary of all antihypertensive medications available in Brazil, including their pharmacological characteristics, dosing and frequency, main indications, and additional information such as major side effects.

**Chart 7.7:** Other medications used in the treatment of hypertension: sympatholytic agents and direct vasodilators – usual daily doses (mg/day), frequency (doses/day), and additional Information

Class and medication	Usual daily dose (mg)	Frequency (doses/day)	Additional Information and recommendations
**CENTRAL SYMPATHOLYTIC AGENTS**			
Methyldopa	500-2000	2	- Medication of choice for hypertension during pregnancy; virtually its only indication
Clonidine	0.2-0.9	2 to 3	- Abrupt withdrawal can cause rebound hypertension (hypertensive crisis) due to catecholamine release at the synaptic terminal - May be used in acute BP elevation and in resistant or refractory hypertension, especially when spironolactone or eplerenone causes intolerable side effects
**PERIPHERAL SYMPATHOLYTIC AGENTS**			
Prazosine	1-20	2 to 3	- Start with a low dose at bedtime due to risk of orthostatic hypotension; increase dose gradually every 2 days - May induce tachyphylaxis and first-dose phenomenon
Doxazosin	1-16	1	- Other alpha-blockers used for benign prostatic hyperplasia, with occasional use in hypertension: tamsulosin, alfuzosin, silodosin
Terazosin	1-20	1	
**DIRECT VASODILATORS**			
Hydralazine	50-200	2 to 3	- May cause sodium and fluid retention, hypervolemia, and reflex tachycardia - Often requires combination with loop diuretics and/or beta-blockers - Should not be used as monotherapy - May be used during pregnancy
Minoxidil	5-40	1	- Indicated in resistant or refractory hypertension, malignant hypertension, or during withdrawal of intravenous vasodilators - Same effects (adverse or otherwise) as hydralazine, but contraindicated in pregnancy and may cause hirsutism

BP: blood pressure; CKD: chronic kidney disease.

### 7.5. Medications Indicated for other Conditions that Affect Blood Pressure and Cardiovascular Events but are Not yet Established in Routine Hypertension Treatment

#### 7.5.1. Sacubitril/Valsartan

Originally developed for the treatment of hypertension, sacubitril/valsartan has demonstrated benefits in reducing morbidity and mortality in patients with HFrEF. However, there is insufficient data to recommend its use solely for the treatment of hypertension.^45,114,199,211,222^

A meta-analysis showed that sacubitril/valsartan was more effective in lowering BP compared to olmesartan.^223,224^ Another network meta-analysis on its use in primary hypertension concluded that the most effective dose, in terms of antihypertensive efficacy assessed by ABPM, was 200 mg/day.^225,226^ Nevertheless, additional RCTs are needed to determine the incremental value of sacubitril/valsartan as an antihypertensive agent in comparison to currently recommended classes of medications, as no systematic reviews or meta-analyses have yet evaluated hard outcomes or adverse effects, such as hypotension. Cough and angioedema are possible side effects. It is important to note that concomitant use of ACEIs or ARBs with sacubitril/valsartan is not recommended.

#### 7.5.2. Sodium-Glucose Cotransporter 2 Inhibitors

Sodium–glucose cotransporter 2 (SGLT2) inhibitors have shown beneficial effects on CV outcomes and renal function in patients with diabetes, as well as in those with CKD from other causes and in HF. However, their effect on BP reduction is modest.^213-215^

The antihypertensive mechanisms of SGLT2 inhibitors involve natriuresis and osmotic diuresis, leading to reduced intravascular volume and peripheral vascular resistance. In addition, activation of tubuloglomerular feedback and the weight loss associated with these agents may further contribute to BP reduction. The presence of SGLT2 receptors in the central nervous system (CNS) may also play a role in their modest BP-lowering effect.^227^

A meta-analysis demonstrated that SGLT2 inhibitors significantly reduced SBP and DBP, as measured by ABPM, in patients with type 2 DM and hypertension. The mean reduction observed was –5.08 mm Hg for SBP and –2.73 mm Hg for DBP.^228^

There is consistent evidence in the literature that SGLT2 inhibitors promote a modest but consistent reduction in BP in patients with type 2 DM, with smaller effects observed in other populations. These effects are clinically relevant, especially when considering their favorable safety profile and additional cardiorenal benefits.^228-231^

#### 7.5.3. Glucagon-Like Peptide-1 Receptor Agonists^216^ and New Selective Nonsteroidal Mineralocorticoid Receptor Antagonists^218,232^

Finerenone, a selective nonsteroidal MRA available in Brazil, and glucagon-like peptide-1 (GLP-1) receptor agonists generally appear to have only modest effects on BP. However, they still require more scientific evidence to be recommended specifically as antihypertensive agents, although they should be considered in the clinical conditions for which they have demonstrated nephroprotective and cardioprotective effects.

A meta-analysis showed that GLP-1 receptor agonists reduced SBP by a mean of –3.37 mm Hg and DBP by –1.05 mm Hg compared with placebo.^233^ Another study confirmed these findings, reporting modest SBP reductions with different GLP-1 receptor agonists, such as semaglutide, liraglutide, and dulaglutide.^233^

A network meta-analysis comparing different GLP-1 receptor agonists showed that subcutaneous semaglutide is among the most effective in reducing SBP and DBP within this class of medication.^234^

Regarding finerenone, ABPM analysis from the ARTS-DN trial (Mineralocorticoid Receptor Antagonist Tolerability Study–Diabetic Nephropathy) showed that this MRA reduced 24-hour, daytime, and nighttime SBP, with a placebo-adjusted reduction of up to –11.2 mm Hg at a 15 mg dose.^235^

Most meta-analyses and systematic reviews involving finerenone focus on renal and CV outcomes in patients with CKD associated with type 2 DM, with BP reduction generally reported as a secondary or exploratory endpoint.^236-238^

### 7.6. Treatment Strategies

Treatment may be initiated with monotherapy in selected cases (Figure 7.3) or with combination therapy, noting that most patients will require two medications from the outset, preferably in a single-pill combination.

**Figure 7.3:** Patients with initial indication for monotherapy.

The pharmacological treatment flow chart is depicted in Figure 7.4.

**Figure 7.4:** Flowchart for pharmacological treatment of hypertension.

#### 7.6.1. Monotherapy

Monotherapy is indicated as an initial antihypertensive strategy exceptionally for the groups of patients described in Figure 7.3.

In these groups, the desired BP reduction is small and/or gradual to avoid serious adverse events.

Treatment should be individualized, and the choice of initial medication should be based on the properties of the antihypertensive agents, the specific characteristics of each patient, the presence of comorbidities and HMOD, as well as socioeconomic factors and the availability of medications in the public health system.^239-245^

BBs may be considered as the initial medication in specific situations, as previously described; however, they are more frequently used in combination with other agents.^211,213,246,247^

#### 7.6.2. Combination Therapy

For most patients, it is recommended to start hypertension treatment with a two-drug regimen, preferably in a single-pill combination when available.^239-243^ At the physician's discretion, patients with stage 1 hypertension and low CV risk, as well as those in other situations described in Figure 7.3, or drug combination.

Preferred combinations should include an RAAS blocker (ACEI or ARB) with either a CCB or a thiazide/thiazide-like DIU. These strategies are supported by evidence showing that combination therapy (preferably in a single-pill combination) achieves greater BP reductions than monotherapy, causes fewer side effects than the individual components, improves treatment adherence and long-term persistence, allows earlier BP control,^114,198-200,225-233,248^ and reduces therapeutic inertia as well as the risk of all-cause mortality and CV hospitalizations.^245,248,250-252^

In the Brazilian public health system, Ministry of Health medication distribution programs do not offer single-pill combinations. Therefore, patients treated in the public sector should receive the available medications in multiple-pill combinations, adjusting the individually tolerated doses of the medications listed in the National List of Essential Medicines, as described in Chapter 14, until BP targets are achieved. If the target BP is not reached with two agents, doses should be titrated to the maximum recommended and tolerated levels according to the patient's characteristics. Subsequently, other classes of antihypertensive medications should be added until BP control is achieved, following the treatment flow chart in Figure 7.4. Whenever possible, patients requiring three antihypertensive agents should receive them in a single-pill combination.

Importantly, combining an ARB with an ACEI is contraindicated because it increases adverse effects without improving outcomes.^235^ This recommendation is based mainly on unfavorable results from the ONTARGET trial,^253^ although two systematic reviews^254,255^ have raised some controversy, resulting in a weak recommendation with low certainty of evidence.^256-259^

**Table:** 

Key messages on hypertension treatment
Pharmacological treatment should always be combined with lifestyle changes (see Chapter 6).
For most patients with hypertension, the recommended initial strategy is a combination of two low-dose antihypertensive medications, whether in a single-pill formulation or not, taken in the morning or evening. Dual therapy allows BP control in over 60% of cases.
If BP control is not achieved with a two-drug regimen, even after titrating individual doses to the maximum tolerated levels, the next step should be a three-drug regimen, preferably in a single-pill formulation when available. Triple therapy allows BP control in approximately 90% of treated patients.
Spironolactone is recommended as a fourth-line treatment. If persistent adverse effects occur, spironolactone should be replaced by eplerenone. Additional antihypertensive drug classes should be introduced progressively, following the recommendations of this Guideline and the treatment flowchart, to achieve BP targets.
If BP targets are still not met, the patient is considered to have RH or RfH and should be referred to a specialized center for further assessment (see Chapter 12).

BP: blood pressure; RH: resistant hypertension; RfH: refractory hypertension.

**Table:** 

Recommendations for pharmacological treatment	Strength of recommendation	Certainty of evidence
The combination of antihypertensive medications, preferably in a single-pill formulation and using the preferred classes, is recommended to achieve strict BP targets (< 130/80 mm Hg) and reduce CV and renal events.	STRONG	MODERATE
Monotherapy is recommended for individuals with BP 130-139/80-89 mm Hg and high CV risk; patients with stage 1 hypertension and low risk (combination therapy may be considered at the physician's discretion); frail individuals; oldest-old adults (≥ 80 years); or those with symptomatic orthostatic hypotension, particularly in older adults.	WEAK	LOW
For most patients, initiation of treatment for hypertension with a two-drug regimen, preferably in a single pill combination, is recommended.	STRONG	MODERATE
Thiazide or thiazide-like diuretics, ACE inhibitors or ARBs, and CCBs are recommended as preferred classes for the treatment of hypertension and for reducing major CV and renal events.	STRONG	HIGH
BBs are recommended for the treatment of hypertension in specific situations: HF, AF, arrhythmias, CAD, hypertension in patients on hemodialysis, and other conditions (eg, migraine, essential tremor, women planning pregnancy, esophageal varices).	STRONG	MODERATE
Spironolactone (or eplerenone in case of intolerance to spironolactone) is recommended to achieve strict BP targets (<130/80 mmHg) and reduce CV and renal events when these have not been achieved with the initial three classes alone (RH and RfH).	STRONG	HIGH
Clonidine is recommended to achieve strict BP targets (<130/80 mm Hg) and reduce CV events in cases where BP targets are not met with initial classes alone (RH and RfH), and in those intolerant to spironolactone/eplerenone or still uncontrolled after their use.	WEAK	LOW
Concomitant use of ACE inhibitors and ARBs is not recommended.	STRONG	HIGH

ACE: angiotensin-converting enzyme; AF: atrial fibrillation; ARBs: angiotensin receptor blockers; BP: blood pressure; CAD: coronary artery disease; CCBs: calcium channel blockers; CV: cardiovascular; HF: heart failure; RfH: refractory hypertension; RH: resistant hypertension.

## 8. Hypertension and Associated Clinical Conditions: Coronary Artery Disease, Chronic Kidney Disease, Diabetes, Obesity, COVID-19, Post-Stroke, and Heart Failure

### 8.1. Hypertension and Coronary Artery Disease

Patients with hypertension and CAD are considered at high or very high CV risk.^260^ Aging plays an important role in influencing the relationship between BP parameters and CAD risk. In patients under 50 years of age, DBP is a stronger risk predictor than SBP or PP, suggesting that increased peripheral vascular resistance (PVR) and altered pulse wave amplification are dominant in determining risk. Between the ages of 50 and 59, all three BP parameters show similar predictive value, suggesting a balance between small vessel resistance and large artery stiffness. From the age of 60, there is a shift in favor of PP and SBP as predictors of CAD risk, indicating that large artery stiffness with early pulse wave reflection becomes the dominant hemodynamic determinant. Although DBP is more relevant than SBP in younger adults, the highest burden of CVD occurs in older individuals with isolated systolic hypertension and wide PP.^261^

Lowering BP is associated with reduced CV risk in patients with CAD. However, early studies suggested that excessively low DBP might impair coronary perfusion. This is particularly relevant in CAD patients, in whom excessive lowering of DBP may trigger myocardial ischemia.^262^ The TNT (Treating to New Targets) trial showed that very low SBP levels – below 110-120/60-70 mm Hg – were associated with increased CV event risk, except for stroke, in which lower pressures were beneficial.^262^

There is ongoing discussion regarding the possible existence of a J-curve phenomenon, already addressed in Chapter 5. In a retrospective cohort of Korean patients with CAD undergoing percutaneous coronary intervention, a J-curve was observed for major complications, with a nadir at 119 mm Hg SBP and 74 mm Hg DBP.^263^ However, a post hoc analysis of a Japanese population–based study on the effects of antihypertensive therapy in CAD patients found no evidence of a J-curve for systolic BP, nor in Japanese CAD patients undergoing percutaneous coronary intervention.^264^

Evidence of a J-curve in BP comes from observational studies, with low certainty of evidence and a high probability of not reflecting a causal relationship between BP reduction and CV outcomes. Such findings are more likely related to residual confounding and/or reverse causality.^128,130,265^

#### 8.1.1. Blood Pressure Targets in Coronary Artery Disease

This guideline recommends BP targets of < 130/80 mm Hg for individuals with hypertension and CAD. Clearly, there is a threshold beyond which BP reduction may cause CV harm; however, there is no evidence to support what this exact value is or how it may vary depending on the presence of comorbidities. Lowering BP to 120/70 mm Hg does not increase CV risk, and asymptomatic patients with BP < 120/70 mm Hg do not need to stop or reduce their antihypertensive medication. BP reduction should be gradual and cautious, according to patient tolerance, particularly in those with evidence of myocardial ischemia, in individuals with diabetes, and in the very older individuals.^266,267^ Attention should be paid to adverse events such as orthostatic hypotension, syncope, electrolyte imbalances, and AKI.^266,267^ In the presence of increased PP, BP reduction should also be gradual and careful due to the possible onset or worsening of myocardial ischemia.^206^

#### 8.1.2. Treatment of Hypertension and Coronary Artery Disease

BBs and both dihydropyridine (DHP) and non-DHP (CCBs) are preferred for treating hypertension in symptomatic patients with CAD. In cases of recent MI, BBs also improve prognosis and should be prescribed unless contraindicated.^268-270^ The duration of the benefits associated with BB use remains uncertain. However, in the absence of specific issues, there is no reason to discontinue BB therapy. Increased HR is linearly correlated with CV events, and the benefit of HR reduction as a treatment target in CAD patients has been demonstrated with various medications, including BBs.^271^ ACEIs have been shown to reduce CV outcomes in patients at high risk. ARBs may be used as alternatives in patients with hypertension and CAD who are intolerant to ACE inhibitors.^272^

**Table:** 

Key messages on hypertension in patients with coronary artery disease
BP control reduces the risk of coronary events.
There is controversy regarding the existence of a J-curve in CAD, as the supporting evidence is of low quality.

BP: blood pressure; CAD: coronary artery disease.

**Table:** 

Recommendations for hypertension management in patients with coronary artery disease	Strength of Recommendation	Certainty of Evidence
BP targets < 130/80 mm Hg are recommended for individuals with hypertension and CAD. Lowering BP to 120/70 mm Hg does not increase CV risk, and asymptomatic patients with BP < 120/70 mm Hg do not need to discontinue or reduce their antihypertensive medication.	STRONG	MODERATE
BP reduction in hypertensive patients with CAD should be performed gradually and cautiously, according to tolerance, especially in those with evidence of myocardial ischemia, diabetes, very older individuals, and those with increased PP. Attention should be paid to adverse events such as orthostatic hypotension, syncope, electrolyte imbalances, and AKI.	WEAK	LOW
BBs are recommended for the treatment of hypertension in symptomatic patients with CAD to reduce major CV events and angina symptoms.	STRONG	HIGH
ACE inhibitors are recommended to reduce CV outcomes in patients with CAD. ARBs may replace ACE inhibitors in patients who are intolerant to this drug class.	STRONG	HIGH
CCBs are recommended for the treatment of hypertension and concomitant CAD, especially in patients with angina, to reduce major CV events and angina symptoms.	WEAK	MODERATE

ACE: angiotensin-converting enzyme; AKI: acute kidney injury; ARB: angiotensin receptor blocker; BB: beta-blocker; BP: blood pressure; CAD: coronary artery disease; CCB: calcium channel blocker; CV: cardiovascular; PP: pulse pressure.

### 8.2. Hypertension in Chronic Kidney Disease in Patients on Conservative Treatment and Dialysis

#### 8.2.1. Blood Pressure Target for Chronic Kidney Disease Under Conservative Treatment

Hypertension is the main risk factor for CKD in Brazil and must therefore be treated effectively and early, avoiding therapeutic inertia.^273^ This is a complex population that often presents with resistant or refractory hypertension, accompanied by other comorbidities and a high risk of progression to kidney failure and CV mortality when not properly controlled.^274^ The optimal BP target for these patients remains controversial in literature.^275,276^

In hypertensive patients with CKD under conservative treatment, the SPRINT (Systolic Blood Pressure Intervention Trial) is used as a reference, proposing an SBP target of < 120 mm Hg compared with < 140 mm Hg,^256^ as a strategy to reduce cardiovascular risk and mortality in adults without diabetes at high cardiovascular risk.^277^ Although this trial underpinned the 2024 update of KDIGO (Kidney Disease: Improving Global Outcomes),^95^ the leading international guideline for CKD management and its complications, achieving this target remains challenging in real-world clinical practice, particularly outside specialized care settings.^276^

SPRINT has characteristics that limit the generalizability of its findings to all patients with hypertension and CKD. A key point is its exclusion criteria: age under 50 years, eGFR < 20 mL/min/1.73 m^2^, proteinuria > 1 g/day, patients with diabetes, polycystic kidney disease, or prior stroke – all of which include a significant portion of individuals diagnosed with CKD in Brazil.^278,279^ Additionally, BP was measured using professional automated sphygmomanometers (Omron® model 907XL), not commercially available in Brazil, and often in an unattended setting, which tends to yield lower values than office-based measurements, making it unsuitable for extrapolation to nonstandardized settings.^49^

Importantly, renal outcomes were not the primary endpoint of the SPRINT trial.^280^ Thus, it is not possible to affirm from its results that intensive BP control slows CKD progression – or even that it does not accelerate it – since it may significantly increase the risk of hypotension, renal hypoperfusion with AKI, electrolyte abnormalities, syncope, and other adverse effects.^256^

According to a 2024 Cochrane systematic review including 6 RCTs with a total of 7,348 participants, lower BP targets (≤ 130/80 mm Hg) compared to standard targets (140-160/90-100 mm Hg) did not show significant differences in all-cause mortality, total CV events, or progression to dialysis-dependent CKD. However, participants in the lower target group achieved lower SBP and DBP values and required a higher number of antihypertensive drugs.^281^

Specifically in patients with diabetes, BP control is associated with reduced albuminuria, improved retinopathy, and reduced stroke risk when more intensive targets are used – though without effects on other CV outcomes.^282^ In the ACCORD trial, no reduction in CV events was observed with SBP < 120 mm Hg,^283^ likely due to the limited statistical power of the study's factorial design. On the other hand, a long-term post hoc analysis of the IDNT trial showed that lowering SBP to 120 mm Hg improved both renal and patient survival.^95^

Thus, the current guideline recommends a BP target of < 130/80 mm Hg for patients with CKD on conservative treatment. A target of SBP < 120 mm Hg may be considered in selected cases, particularly in younger patients with fewer comorbidities, without frailty, using automated and preferably unattended BP measurements, and under close monitoring.

The debate around the optimal BP target for preventing CV and renal outcomes must be standardized to ensure a unified message across all hypertension and diabetes guidelines. However, while further RCTs with global applicability – especially in resource-limited settings – are still lacking, caution is warranted due to ongoing disagreement, even among highly reputable institutions such as KDIGO and Cochrane.^117,245,276,281,284-289^

#### 8.2.2. Blood Pressure Target for Patients with Chronic Kidney Disease on Dialysis

There are no long-term RCTs specifically designed to determine ideal BP targets in hypertensive patients on dialysis. Several observational studies have demonstrated a U-shaped (or J-shaped) relationship between BP control and mortality in these patients.^290-292^

The CRIC (Chronic Renal Insufficiency Cohort) study also identified a U-shaped relationship between all-cause mortality and SBP measured in dialysis centers among patients on hemodialysis (HD) (n = 326). However, when SBP was measured outside the dialysis center, the study found a linear association with mortality. These results suggest that BP assessment in dialysis patients should ideally be performed during the interdialytic period, preferably using 44-hour ABPM, or HBPM if ABPM is unavailable.^293^

Because of the lack of robust evidence from more than one RCT and considering the higher risks of hypotension, poor treatment adherence, and potential worsening of renal injury in patients under intensive BP control, a target BP < 130/80 mm Hg is considered reasonable for patients on conservative treatment or with kidney transplants.^281^ For patients on HD or hemofiltration, a pre-dialysis BP target of < 140/90 mm Hg and a post-dialysis BP target of < 130/80 mm Hg is recommended. Post-dialysis BP should be measured after catheter sealing or arteriovenous fistula hemostasis.^294^ Finally, for peritoneal dialysis (PD), a target BP < 140/90 mm Hg is suggested, preferably measured by 24-hour ABPM.^295^

#### 8.2.3. Treatment of Hypertension in Chronic Kidney Disease on Conservative Management

Alongside pharmacological treatment, the implementation of NPMs is essential^95^ (Chapter 6). ACE inhibitors or ARBs are first-line agents for patients with hypertension and CKD, with or without albuminuria, due to their effects on reducing renal and CV events; however, their combined use is not recommended. Thiazide or thiazide-like diuretics, loop diuretics, and CCBs are effective and appropriate for combination therapy. BBs are indicated in CAD and HF. MRAs (spironolactone and eplerenone) have cardioprotective effects and are effective in RfH, though the risk of hyperkalemia is high, particularly in patients with CKD stages 3b–5. This does not contraindicate their use but requires close monitoring.^95^

Recently, drugs with modest antihypertensive effects but proven renal and CV protection have been incorporated into CKD treatment, especially in diabetic patients. SGLT2 inhibitors stand out by reducing renal and CV outcomes in patients with and without diabetes and with eGFR > 20 mL/min.^231,296^ Finerenone, a non-steroidal MRA, also offers renal and CV protection, as shown in patients with diabetes and CKD.^297,298^ Additionally, semaglutide, a GLP-1 receptor agonist, has been shown to reduce serious renal and CV events in patients with CKD and type 2 DM.^276^

**Table:** 

Key messages on hypertension in patients with chronic kidney disease on conservative treatment
Hypertension is the leading cause of CKD in Brazil and must therefore be treated effectively and early.
Hypertension in CKD is often resistant or refractory to treatment.
The BP target in CKD patients under conservative treatment is controversial, with systematic reviews and meta-analyses presenting divergent conclusions, even among highly credible institutions such as Cochrane and KDIGO.

BP: blood pressure; CKD: chronic kidney disease.

#### 8.2.4. Treatment of Hypertension in Patients with Chronic Kidney Disease on Dialysis

Given the specificity of this topic for nephrologists, we recommend following the guidance of the I Brazilian Guideline on Hypertension in Dialysis of the Brazilian Society of Nephrology for both NPMs and pharmacological treatment, which are summarized below.^295^

The following is recommended for patients on dialysis:

Loop diuretics for all patients on dialysis with residual diuresis;

ACE inhibitors or ARBs in hypertensive dialysis patients, especially due to their pleiotropic effects: reduction in left ventricular hypertrophy, HF, peritoneal fibrosis in PD, and CV mortality;

CCBs are recommended in patients on HD or PD, due to their antihypertensive and CV protective effects;

MRAs are recommended in HD or PD patients due to their antihypertensive effects, mortality reduction, and cardioprotective effects, if tolerated;

MRAs are recommended in HD or PD patients for their antihypertensive effects, CV protection, and mortality reduction – provided they are tolerated.

**Table:** 

Recommendations for hypertension management in patients with chronic kidney disease on conservative treatment ^231,299,300^	Strength of Recommendation	Certainty of Evidence
In adults with hypertension and CKD, a BP target of < 130/80 mm Hg is recommended to reduce CV events and kidney failure.	STRONG	HIGH
For patients under conservative CKD treatment, a SBP target of < 120 mm Hg is recommended in selected cases – particularly in younger patients with fewer comorbidities, no frailty, with BP measured using automatic, preferably unattended methods under supervision.	STRONG	MODERATE
The following targets are recommended for dialysis patients: HD: pre-session BP < 140/90 mm Hg or post-HD BP < 130/80 mm Hg Hemodiafiltration: < 130/80 mm Hg PD: < 140/90 mm Hg.	WEAK	VERY LOW
For patients on HD or PD, BP monitoring is recommended to be performed preferably using 44-hour or 24-hour ABPM, respectively. HBPM is an alternative if ABPM is unavailable.	WEAK	LOW
The choice of antihypertensive agents in CKD-related hypertension should, unless contraindicated, include ACE inhibitors or ARBs, and favor the initiation and maintenance of drugs with modest antihypertensive effects but with proven nephroprotective and cardioprotective properties, such as finerenone and GLP-1 receptor agonists in patients with diabetes mellitus, as well as SGLT2 inhibitors, due to their established cardiorenal protective benefits.	WEAK	HIGH
Potassium levels and renal function should be monitored during hypertension treatment in patients with CKD under conservative management.	STRONG	HIGH

ACE: angiotensin-converting enzyme; ARB: angiotensin receptor blocker; BP: blood pressure; CKD: chronic kidney disease; GLP-1: glucagon-like peptide-1; HD: hemodialysis; PD: peritoneal dialysis; SGLT2: sodium-glucose cotransporter 2.

### 8.3. Hypertension and Diabetes

The association between hypertension and diabetes is common and appears to be bidirectional – hypertension may contribute to the development and worsening of diabetes, and the reverse is also true. Approximately 80%-90% of patients with diabetes will develop hypertension, complicating disease management and increasing the risk of both microvascular and macrovascular complications, such as kidney disease, retinopathy, PAD, and CVD. Strict BP control is therefore essential.^278^ Clinical and complementary evaluations should always follow the guidance provided in Chapter 4.

Assessment and classification of albuminuria using a spot urine sample, preferably the ACR, are recommended for evaluating renal involvement, and the use of the terms microalbuminuria and macroalbuminuria is not recommended.^289^ (Chapter 4). Albuminuria should be confirmed in two or three urine samples, as factors such as exercise, fever, or high-protein meals may interfere with results and do not necessarily indicate kidney damage.

BP monitoring outside the office is recommended. ABPM and HBPM should be used to identify MH and WCH, which are common in patients with diabetes.^301^ These methods support both diagnosis and treatment evaluation.

Patients with well-controlled diabetes of less than 10 years’ duration, without evidence of HMOD and no additional CV risk factors, are classified as having moderate risk. However, those at higher risk – such as individuals with a history of CV events or evidence of organ damage – are considered high-risk.^255,302-304^

#### 8.3.1. Blood Pressure Targets in Patients with Diabetes

The association between hypertension and diabetes increases the risk of microvascular and macrovascular complications, making rigorous BP control essential. On the other hand, no data are available for patients with newly diagnosed diabetes, without complications and thus at relatively low CV risk. In general, BP control is more difficult in individuals with diabetes.

The ACCORD (Action to Control Cardiovascular Risk in Diabetes) trial^283^ did not confirm that targeting SBP < 120 mm Hg reduces CV event rates in individuals with diabetes. However, stroke incidence was reduced by 41% with intensive treatment. The ADVANCE (Action in Diabetes and Vascular Disease: Preterax and Diamicron MR Controlled Evaluation) trial^305^ showed that lowering SBP to 135 mm Hg significantly reduced CV events compared to 140 mm Hg.

A recent study, BPROAD (Intensive Blood-Pressure Control in Patients with Type 2 Diabetes), was designed specifically to evaluate the benefits of intensive BP control in patients with type 2 DM.^306^ The study included 12,821 diabetic patients aged over 50 with elevated SBP and high CV risk. Over an average follow-up of 4.2 years, patients in the intensive-treatment group (target SBP < 120 mm Hg) experienced a 21% reduction in major CV events compared to the standard-treatment group (target SBP < 140 mm Hg), indicating a benefit in reducing events such as stroke, MI, and HF. Albuminuria occurred less frequently in the intensive-treatment group. Although the incidence of serious adverse events was similar between the groups, symptomatic hypotension and hyperkalemia were more frequent in the intensive-treatment group. The inclusion of only Chinese participants may limit the generalizability of the findings to other populations.^306^

One of the largest meta-analyses on BP reduction in type 2 DM, including over 70,000 participants, found that tighter BP control significantly reduced stroke risk by 31%, but did not reduce MI risk.^282^

Based on current evidence, this guideline recommends that individuals with both diabetes and hypertension be treated to achieve BP targets <130/80 mmHg.

#### 8.3.2. Treatment of Hypertension in Patients with Diabetes

Antihypertensive treatment in patients with diabetes must always be individualized. Potential adverse effects of therapy should also be considered. Older individuals, those with CKD, and those who are frail are at greater risk of adverse effects from intensive BP control. Additionally, patients with orthostatic hypotension, significant comorbidities, functional limitations, or polypharmacy may be at high risk for adverse effects, and in such cases, less aggressive BP targets may be preferred to preserve quality of life.^307^

The treatment of hypertension is based on NPMs,^308^ already described in Chapter 6, and pharmacological therapy. Since these patients are at high CV risk, treatment should begin with two different classes of antihypertensive drugs, preferably combined in a single pill. A number of RCTs recommend agents that block the renin–angiotensin-aldosterone system (RAAS) as first-line therapy – using an ACE inhibitor or an ARB in patients intolerant to ACE inhibitors.^67^ This recommendation is supported by robust evidence of the effectiveness of ACE inhibitors in reducing both microvascular and macrovascular outcomes in patients with diabetes.^304,309^

Other antihypertensive agents such as CCBs and diuretics^310^ are also fundamental in initial therapy, considering the individual's clinical profile and needs. When choosing a diuretic as a second agent, one must consider its metabolic effects.^311^ Thiazide-like diuretics (eg, chlorthalidone and indapamide) have advantages over hydrochlorothiazide (HCTZ). Among them, indapamide has the best evidence of fewer adverse metabolic effects when compared to chlorthalidone.^312^

When planning strategies for the management of hypertension in diabetes, the impact of initiating or discontinuing SGLT2 inhibitors or GLP-1 receptor agonists should be considered. These two drug classes produce modest BP reductions, but significantly reduce CV events.^296,299,313^

In patients with diabetic kidney disease, finerenone – a selective nonsteroidal MRA – has shown both cardiac and renal protection, along with slight reductions in BP.^297^

Finally, patient education in self-management techniques, including HBPM and the use of technology-based tools, helps improve treatment adherence and the adoption of healthy lifestyles – both of which are essential for effective, sustained disease control.^298,308^

**Table:** 

Key messages on hypertension in patients with diabetes
The association between hypertension and diabetes is common. Hypertension may contribute to the development and worsening of diabetes, and the reverse may also be true.
Approximately 80%-90% of patients with diabetes will develop hypertension, increasing the risk of microvascular and macrovascular complications – such as kidney disease, retinopathy, PAD, and CVD – which makes strict BP control essential.
NPMs should be prescribed for all patients with both hypertension and diabetes.

BP: blood pressure; CVD: cardiovascular disease; PAD: peripheral artery disease; NPM: nonpharmacological measures.

**Table:** 

Recommendations for hypertension management in patients with diabetes	Strength of Recommendation	Certainty of Evidence
For the assessment of BP, the use of out-of-office BP measurement, using ABPM and/or HBPM, is recommended.	STRONG	MODERATE
Urinary albumin measurement is recommended using two or three samples at diagnosis and subsequently, as clinically indicated	STRONG	HIGH
A BP target of < 130/80 mm Hg is recommended for patients with hypertension and diabetes	STRONG	HIGH
Pharmacological treatment should be initiated immediately upon diagnosis with two drugs: an ACE inhibitor or, in cases of intolerance to ACE inhibitors, an ARB, combined with either a CCB or a thiazide-like diuretic	STRONG	HIGH

ABPM: ambulatory blood pressure monitoring; ACE: angiotensin-converting enzyme; ARB: angiotensin receptor blocker; BP: blood pressure; CCB: calcium channel blocker; HBPM: home blood pressure monitoring.

### 8.4. Hypertension and Obesity

Obesity is closely linked to hypertension through various pathophysiological mechanisms. Epidemiological studies, such as the Framingham Heart Study^314^ and the Nurses’ Health Study,^315^ have demonstrated a direct, continuous, and almost linear relationship between BMI and BP. Obesity, especially abdominal obesity, interferes with both the endocrine and immune systems, increasing the risk of insulin resistance, diabetes, hypertension, and CVD.^316^

Different patterns of body fat distribution play an important role in the association between hypertension and obesity. Body fat may be classified as central or peripheral. Central or abdominal fat is referred to as visceral fat. However, individuals with the same waist circumference (WC) may have a predominance of subcutaneous or intra-abdominal (visceral) fat. Visceral fat differs anatomically, functionally, and genetically from subcutaneous fat.^317^ Recent studies have demonstrated a strong association between visceral fat and hypertension.^318^

Visceral obesity increases the risk of primary hypertension by 65%-75%.^319-321^ The pathophysiological mechanisms behind this association are complex and not yet fully understood. Notable among them are: (a) sympathetic-vagal imbalance, with dominance of sympathetic activity over parasympathetic; (b) paracrine production of angiotensinogen, angiotensin II, and aldosterone by adipocytes; and (c) renal compression due to increased intra-abdominal and retroperitoneal fat, leading to reduced sodium excretion and activation of the RAAS.^319^ In addition to these mechanisms, reduced levels of natriuretic peptides in obesity and the production of various adipocytokines appear to play important roles in the pathogenesis of obesity-related hypertension.^322^

#### 8.4.1. Blood Pressure Target in Patients with Obesity

The recommended BP target for patients with obesity is < 130/80 mm Hg, similar to other high CV and renal risk conditions.

#### 8.4.2. Treatment of Hypertension in Obesity

The main focus of hypertension treatment in individuals with obesity is weight reduction, aiming for a stable BMI between 20 and < 25 kg/m², through NPMs, pharmacological therapy, and obesity surgery, when indicated.^144^

NPMs should aim to achieve at least a 5–10% reduction in body weight through the measures already described in Chapter 6. These measures may result in weight loss and better control of BP and metabolic parameters.^144^

A wide range of anti-obesity medications with different mechanisms of action has been studied.^323^ Most of these agents have shown modest effects on weight and BP reduction. Recently, GLP-1 receptor agonists (GLP-1 RAs), such as liraglutide, semaglutide, and dulaglutide, and tirzepatide, a dual GLP-1 and glucose-dependent insulinotropic polypeptide (GIP) agonist, have been used with favorable results in reducing weight and CV risk,^324^ have shown promising results in terms of weight reduction and CV risk reduction. However, their impact on BP reduction remains modest.^305^

Bariatric surgery has shown favorable results in terms of weight and BP control in hypertensive patients. Nonetheless, the indication for these procedures should follow thorough clinical evaluation and well-established protocols.^282,325-329^

Regarding the preferred antihypertensive drugs in obese patients, several drug classes with different mechanisms of action are effective for BP reduction. However, they may have favorable or unfavorable metabolic effects^330^ (Chart 8.1).

**Chart 8.1:** Preferred antihypertensive drug classes in patients with obesity

Drug class	Main action	Effects on insulin
Diuretics	Natriuresis, ↓PVR	↓Insulin sensitivity
ACE inhibitors or ARBs	Natriuresis, ↓aldosterone, ↓PVR	↑Insulin resistance
CCBs	↓PVR	↓Plasma insulin
BBs	↓Production of renin, ↓PVR	↓Insulin sensitivity

ACE: angiotensin-converting enzyme; ARBs: angiotensin receptor blockers; BB: betabloqueadores; CCB: calcium channel blocker; PVR: peripheral vascular resistance.

**Table:** 

Key messages for patients with hypertension and obesity
Obesity is closely related to hypertension through various pathophysiological mechanisms.
There is a strong association between visceral fat and hypertension, with visceral obesity increasing the risk of primary hypertension by 65%-75% due to complex pathophysiological mechanisms.

**Table:** 

Recommendations for hypertension management in patients with obesity	Strength of Recommendation	Certainty of Evidence
A BP target of < 130/80 mm Hg is recommended for patients with obesity.	STRONG	MODERATE
In addition to NPMs, treatment of individuals with obesity and hypertension should include diuretics, RAAS blockers, CCBs, or BBs (in specific conditions), noting that each of these drug classes may interfere with metabolic parameters.	STRONG	LOW
Weight-loss medications such as GLP-1 receptor agonists (eg, liraglutide, semaglutide, and dulaglutide) are recommended, as they have shown favorable results in reducing weight and CV risk, although their effects on BP reduction are modest.	STRONG	HIGH

BB: beta-blocker; BP: blood pressure; CCB: calcium channel blocker; CV: cardiovascular; GLP-1 RA: glucagon-like peptide-1 receptor agonist; RAAS: renin–angiotensin–aldosterone system.

### 8.5. Hypertension and COVID-19

Since the beginning of the COVID-19 pandemic in 2019, noncommunicable diseases have been associated with increased disease severity and mortality,^331^ and among them, hypertension was one of the most prevalent comorbidities, consistently reported as a marker of poor prognosis^304,309^ due to its association with a higher risk of severe COVID-19.^96,97,332-334^

Studies have shown that hypertension remained a risk factor for greater COVID-19 severity even after adjusting for age.^333,335^ In treated hypertensive patients, increased SBP demonstrated a dose–response relationship with severe COVID-19, even when adjusted for age and CV comorbidities.^335^

One possible explanation for this association with increased severity is that hypertension often causes subclinical damage to vital organs,^87^ potentially weakening the body's defenses against severe infections. Another hypothesis is the immune dysregulation present in hypertension.^336^ Studies have shown an association between severe COVID-19 and the presence of shorter telomeres in chromosomes,^337^ which in turn are also linked to various comorbidities, including hypertension.^338^

Hypertension is also significantly present in long COVID, which occurs three months after the onset of the disease, with symptoms lasting for at least two months. Evidence suggests that the clinical course of hypertension-related disorders, such as CVD and kidney disease, is affected by long COVID. However, further investigation is needed.^339^

Regarding SARS-CoV-2 vaccination, it is recommended for patients with hypertension. No evidence of increased BP or consistent alterations emerged from RCTs assessing the safety and efficacy of the vaccines.^340^

The SARS-CoV-2 coronavirus uses the ACE2 receptor to enter cells. Some experimental studies have suggested that treatment with ACE inhibitors or ARBs could increase ACE2 expression, raising concerns that such treatment might enhance viral infectivity and severity. However, a systematic review showed that upregulation of ACE2 by RAAS blockers was a rare event.^341^ Observational studies and meta-analyses showed that treatment with RAAS inhibitors did not affect the risk of COVID-19 infection, severe disease, or mortality.^342,343^

**Table:** 

Key messages for patients with hypertension and COVID-19
Patients with hypertension are at increased risk of COVID-19 morbidity and mortality.
SARS-CoV-2 vaccination is indicated for patients with hypertension.
Hypertension is notably present in long COVID-19, which occurs three months after disease onset, with symptoms lasting at least 2 months. The clinical course of hypertension-related disorders, such as CVD and kidney disease, is affected by long COVID.

CVD: Cardiovascular disease.

**Table:** 

Recommendations for patients with hypertension and COVID-19	Strength of Recommendation	Certainty of Evidence
Patients treated with RAAS blockers for hypertension or other clinical conditions should continue treatment in the presence of COVID-19.	STRONG	HIGH

RAAS: renin–angiotensin–aldosterone system.

### 8.6. Hypertension, Stroke, and Cognitive Impairment

Hypertension is one of the most important risk factors for stroke and cognitive impairment. Hypertension contributes to approximately 60% of the risk of chronic cerebrovascular disease across populations since it directly affects brain structure and microvasculature.^344^ The goal of the treatment of hypertension in patients who have experienced a stroke or transient ischemic attack (TIA) is to reduce the risk of recurrence and other CV complications as well as to prevent the onset of dementia or slow the progression of cognitive impairment.^345,346^

In the INTERSTROKE study,^347^ individuals with hypertension were twice as likely to suffer a stroke in a primary prevention context. Therefore, BP control after a stroke is critical for secondary prevention, reducing the risk of recurrent cerebrovascular events and cognitive decline.

#### 8.6.1. Blood Pressure Target in Chronic Phase Post-stroke Hypertension

BP reduction during the chronic phase of stroke is highly effective in preventing recurrent strokes, protecting against CV events, preserving cognitive function, and reducing overall mortality. In the RESPECT trial,^348^ authors concluded that the reductions in recurrence rates in the intensive-treatment group (SBP < 120 mm Hg) compared to the standard-treatment group (SBP < 140 mm Hg) were not statistically significant. However, a meta-analysis combining these results with three previous trials showed a 22% reduction in recurrent cerebrovascular events with more intensive BP lowering.^348^

The ESPRIT (Effects of Intensive Systolic Blood Pressure Lowering Treatment in Reducing Risk of Vascular Events) study included patients with diabetes and those with prior stroke and confirmed that an intensive treatment strategy (SBP <120 mmHg) can prevent CV events. Syncope, as a serious adverse event, occurred more frequently in the intensive-treatment group.^132^

Based on clinical trial findings, the BP target recommended by the current guideline is < 130/80 mm Hg. This same target is also recommended by the AHA/American Stroke Association^349,350^ and the European Stroke Organisation (ESO) Guidelines^351^ for patients with stroke or TIA for secondary stroke prevention.

Similar findings were reported in a standard meta-analysis and a meta-regression of 10 RCTs involving 40,710 patients which assessed the association between the magnitude of differential BP reduction and stroke recurrence in patients with stroke or TIA.

After 2.8 years, pooled results showed that more intensive treatment compared to less intensive treatment was associated with a reduced risk of recurrent stroke (absolute risk [AR]: 8.4% vs. 10.1%; relative risk [RR], 0.83; 95% CI, 0.78-0.88). The meta-regression demonstrated a log-linear association between the magnitude of SBP and DBP reduction and a lower risk of recurrent stroke in patients with stroke or TIA. Similar associations were observed between BP reduction and major CV events.^352^

Acute ischemic or hemorrhagic stroke cases are discussed in Chapter 11.

#### 8.6.2. Treatment of Hypertension in the Chronic Phase after Stroke

Regarding treatment, a systematic review with meta-analysis of 8 studies including 33,774 patients with stroke or TIA followed for 25 months showed that a subsequent stroke occurred in 7.9% (7.4% ischemic stroke and 0.6% hemorrhagic stroke) of patients taking any type of drug to lower BP, compared to 9.7% in those taking placebo (odds ratio [OR], 0.79 [95% CI, 0.66-0.94]; AR reduction [ARR], −1.9% [95% CI, −3.1% to −0.5%]).

Moderate-quality evidence indicated that mortality rates were similar between patients receiving any antihypertensive treatment and those receiving placebo, with ARs of 7.3% and 7.9%, respectively (OR, 1.01 [95% CI, 0.92-1.10]; ARR 0.1% [95% CI, −0.6% to 0.7%]).^353^

Evidence is still limited to support specific drug recommendations for treating patients with prior stroke or TIA. Treatment is usually performed with thiazide diuretics or similar drugs and RAAS blockers (ACE inhibitors or ARBs) targeting secondary prevention.^349,354,355^ CCBs appear to be effective but increase the risk of HF. A Cochrane review also found these two drug classes to be the most frequently used, based on 11 RCTs involving 38,742 patients.^356^ However, additional studies are needed to determine the best drug combinations, particularly for secondary stroke prevention. A combination of three drugs is currently being tested after intracerebral hemorrhage in the TRIDENT trial (Triple Therapy Prevention of Recurrent Intracerebral Disease Events Trial; NCT02699645), though results are still pending.^357^

Therefore, our recommendation is to use thiazide diuretics or similar agents, such as indapamide (as used in the PROGRESS trial), in combination with a RAAS blocker.^358^ If the BP target is not reached, the next step would be the addition of a DHP CCB.

The treatment of hypertension is essential for reducing the global burden of dementia or cognitive impairment at the population level. Recent meta-analyses convincingly support the effectiveness of BP reduction in lowering the risk of dementia or cognitive impairment.^345,346^ The SPRINT MIND study concluded that intensive BP control does not have a harmful effect on cognitive function in patients with low DBP.^359^ Although the SYST-EUR (Systolic Hypertension in Europe)^360^ study suggested that long-acting CCBs may be superior, no specific antihypertensive class has been definitively established as preferable for preventing dementia and cognitive impairment.^361-363^

The role of competing risk mechanisms – including orthostatic hypotension^364^ and BP variability^363^ – may be important in treatment decisions for individuals with frailty, multimorbidity, and/or chronic cerebrovascular disease.

**Table:** 

Key messages for patients with hypertension, stroke, and cognitive impairment
Hypertension is the most important risk factor for stroke and cognitive impairment (60%).
Evidence is limited to support recommendations for specific antihypertensive drug classes in patients with prior stroke or TIA.
No specific antihypertensive drug class has been established as preferable for preventing dementia or cognitive impairment.

TIA: transient ischemic attack.

**Table:** 

Recommendations for hypertension management in post-stroke patients with cognitive impairment	Strength of Recommendation	Certainty of Evidence
A BP target of < 130/80 mm Hg is recommended for patients with prior stroke or TIA to reduce major CV events (MI, stroke recurrence, death).	STRONG	HIGH
A BP target of < 130/80 mm Hg is recommended for the prevention of cognitive impairment and dementia.	WEAK	MODERATE

BP: blood pressure; CV: cardiovascular; MI: myocardial infarction; TIA: transient ischemic attack.

### 8.7. Hypertension and Heart Failure with Preserved and Reduced Ejection Fraction

More than 90% of patients who develop HF have hypertension, and the most common comorbidities are type 2 DM, obesity, AF, and CAD.^365^ Hypertension is considered a risk factor for both HF phenotypes – HFrEF and HF with preserved EF (HFpEF). Proper treatment of hypertension reduces the incidence of HF and should be effectively implemented in all patients with BP ≥ 140/90 mm Hg.^1,117,366^However, no long-term, controlled RCTs have been conducted in patients with HF comparing different treatment targets.^367,368^

Thus, BP target recommendations for patients with HFpEF and HFrEF are extrapolated from studies including populations at high risk and should be < 130/80 mm Hg.^367,368^

Many patients with HFrEF tend to be hypotensive but require multiple drugs with antihypertensive action that also improve prognosis – such as BBs, ACE inhibitors or ARBs, MRAs, diuretics, sacubitril/valsartan, SGLT2 inhibitors, and direct vasodilators (hydralazine combined with nitrate). From this perspective, low BP values should not limit the therapeutic optimization of these medications in patients with HFrEF, as long as they are tolerated without adverse events.^368,369^

In patients with HFpEF, the ideal BP range remains unclear. An observational study of 3,417 patients with HFpEF compared CV event and mortality rates in intensive vs. standard DBP control, and found that intensive DBP control increased the risk of adverse events.^370^ A post hoc analysis of an RCT assessing mortality and adverse outcomes in 3,915 hospitalized patients with HFpEF showed that SBP < 120 mm Hg was significantly associated with worse outcomes.^371^ However, due to study design limitations, it is unclear whether these findings were caused by antihypertensive treatment per se, or if patients with lower BP values had worse clinical and cardiac function–and therefore worse prognosis. Thus, there is still insufficient evidence to determine a minimum BP target in HFpEF patients.

Regarding treatment of HFpEF, drug therapy appears to be the same regardless of EF and should be guided by predominant symptoms (related to sodium and water retention), signs (tachycardia), severity (functional class), and comorbidities. All patients with HF should receive the same drug classes recommended for HFrEF, if well tolerated (Figure 8.1).^372^

**Figure 8.1:** Single blood pressure target for hypertension in special situations of high cardiovascular and renal risk.

**Table:** 

Key messages on hypertension in patients with heart failure
Over 90% of patients who develop HF have hypertension, with the most frequent comorbidities being DM, obesity, AF, and CAD.
Hypertension is a risk factor for both HF phenotypes (HFrEF and HFpEF).
Proper management of hypertension reduces the incidence of HFrEF or HFpEF and should be implemented in all patients with BP ≥ 140/90 mm Hg.
Low BP levels should not prevent therapeutic dose optimization of these medications (BBs, ACE inhibitors [or ARBs], MRAs, diuretics, sacubitril/valsartan, SGLT2 inhibitors, and direct-acting vasodilators [hydralazine in combination with nitrate]) in patients with HFrEF or HFpEF, provided they are tolerated without adverse effects.
Antihypertensive treatment in HF appears to be the same regardless of EF and should be guided by predominant symptoms.

AF: atrial fibrillation; BP: blood pressure; CAD: coronary artery disease; EF: ejection fraction; HF: heart failure; HFpEF: HF with preserved EF; HFrEF: HF with reduced EF; DM: diabetes mellitus.

**Table:** 

Recommendations for hypertension management in patients with heart failure	Strength of Recommendation	Certainty of Evidence
A BP target of < 130/80 mm Hg is recommended for patients with hypertension and HFpEF or HFrEF.	WEAK	LOW
The use of antihypertensive drugs that improve prognosis – BBs, ACE inhibitors (or ARBs), and MRAs – is recommended in patients with HFrEF and hypertension.	STRONG	HIGH
The use of diuretics is recommended for treating patients with hypertension and HF, when signs of hypervolemia are present.	STRONG	HIGH
The use of sacubitril/valsartan is recommended for patients with HFrEF or HFpEF and hypertension.	STRONG	MODERATE
The use of SGLT2 inhibitors is recommended for patients with HFrEF or HFpEF and hypertension to reduce hospitalization and CV mortality.	WEAK	HIGH

ARB: angiotensin receptor blocker; BP: blood pressure; CV: cardiovascular; EF: ejection fraction; HF: heart failure; HFpEF: HF with preserved EF; HFrEF: HF with reduced EF; SGLT2: sodium-glucose cotransporter 2.

## 9. Hypertension in Older Adults, Children, and Adolescents

### 9.1. Hypertension in Older Adults

#### 9.1.1. Introduction

Brazil is undergoing a rapid population aging process, with the oldest-old (≥80 years) representing the fastest-growing age group.^373^ More than 60% of older adults have hypertension, with a high prevalence of isolated systolic hypertension and low control rates.^374^ Hypertension is one of the main modifiable risk factors for CV morbidity and mortality, cognitive decline, dementia, and loss of functional capacity in older adults.^375,376^

Functional status is far more important than chronological age per se; therefore, no treatment should be denied or withdrawn solely on the basis of age.^377,378^

#### 9.1.2. Pathophysiological Mechanisms

Aortic stiffening caused by vascular aging accelerates PWV toward peripheral circulation (centrifugal) and reflected waves returning to the heart (centripetal). The superimposition of these two waves during the protomesosystolic phase leads to the increase in SBP and widening of pulse pressure (PP = SBP – DBP), a useful hemodynamic marker of arterial stiffness, seen in older adults.^379^ Arterial stiffness is a useful prognostic marker for CV outcomes.^380^

#### 9.1.3. Diagnosis and Therapeutic Decision-Making

##### 9.1.3.1. Diagnosis

The investigation of hypertension in older adults may be more challenging due to the presence of multiple comorbidities and polypharmacy. The recommendations from Chapters 3 and 4 of these Guidelines regarding BP measurement, physical examination, and laboratory evaluation should also be followed for this age group. However, certain characteristics specific to older adults must be considered during their clinical and laboratory assessment.

The evaluation of older patients, particularly the oldest-old, differs from a traditional medical evaluation. First, the physician should consider that these consultations will be more time-consuming because of several factors, such as the complexity of multiple comorbidities; the patient's physical and cognitive slowness; the presence of family members and caregivers with whom the physician must discuss aspects of the patient's clinical condition and proposed therapeutic strategies; and, in the case of very frail patients, the potential need for additional consultations because the patient may become exhausted.^381^

Given the greater variability in BP and certain peculiarities of this population, BP measurements may yield inaccurate values. The main factors that interfere with BP measurement in older patients include auscultatory gap, pseudohypertension, and postural and postprandial variations^382^ (see Chapter 3).

Due to increased arterial stiffness, changes in intravascular volume have a significant impact on BP control. Older adults have decreased baroreceptor sensitivity and, therefore, are more prone to OH and postprandial hypotension (PPH). These conditions are also associated with the higher prevalence of neurodegenerative disorders in this population. Approximately 20% of older adults present with OH, and about 30% experience hypotension after meals. Therefore, older adults should be carefully monitored for both OH and PPH. In these patients, ABPM is particularly useful for characterizing BP patterns as well as the often atypical associated symptoms.^383,384^

Poorly controlled hypertension and certain antihypertensive medications, such as alpha-blockers, can trigger or worsen OH. The best approach for managing OH involves NPMs, including adequate hydration, increased salt intake, slow positional changes, elevating the head of the bed, and using compression stockings.

For PPH, older adults should avoid large meals, excessive carbohydrate and alcohol intake, and practicing physical activity immediately after eating.^385^

Out-of-office BP monitoring, whether using ABPM or HBPM, is increasingly valued and recommended for diagnosing hypertension in older patients. Self-measurement of BP, despite its limitations, should also be considered (see Chapter 3).^46^

When ordering additional tests, the investigation of secondary causes of hypertension should be approached cautiously, weighing the risks and benefits of each procedure (see Chapter 4).^386^

The evaluation of older patients should also include the assessment of functional status and frailty, as well as of cognitive function – parameters that are essential for therapeutic decision-making and choosing medications.

##### 9.1.3.2. Assessment of Functional Status and Frailty

In older adults, and especially in the oldest-old, particular attention should be given to functional status and frailty. The Comprehensive Geriatric Assessment, conducted using standardized scales and tests, allows accurate characterization of the overall condition of the patient and helps design strategies for therapeutic management.^387,388^ While this is the optimal method of evaluation, it may require the involvement of a geriatrician or gerontologist.

Routine use of functional tests is recommended, such as gait speed (GS), which can be easily implemented in clinical practice and was shown to be a reliable predictor of survival.^389^ Individuals are considered frail or at risk for frailty if they present with GS < 0.8 m/s (or are unable to walk 6 meters in less than 8 seconds), warranting further investigation.^390^ Additionally, we suggest the use of the Clinical Frailty Scale, which has already been translated and validated for use in Brazil (Figure 9.1),^391^ adapted from the Canadian Clinical Frailty Scale. This tool has been extensively tested and applied due to its simplicity, reliability, comprehensive view of the patient, and ability to determine prognosis.^392^

**Figure 9.1:** Clinical frailty scale.^391^

Frailty is associated with a higher risk of hypertension, subclinical disease, CV events, and mortality.^393-395^ Adequate hypertension control may potentially influence the course of frailty. Conversely, advanced degrees of frailty are associated with lower BP values, lower BMI, reduced muscle mass, poorer cognition, and higher mortality.^395^

##### 9.1.3.3. Assessment of Cognitive Function

In addition to being well established as the main cause of stroke, hypertension is also implicated as a pathogenic factor in cognitive impairment, both of vascular origin and in Alzheimer's disease – which are the leading causes of dementia in older adults, with a more pronounced effect over the long term.^396^

A few RCTs have shown that lowering BP with antihypertensive medication reduces white matter lesion volume, cognitive decline, and – to a lesser extent – dementia, with intensive treatment proving even more effective.^346,397,398^ However, these studies have some limitations in demonstrating a reduction in dementia cases, such as cognition not being the primary endpoint, inconsistency in dementia definitions and cognitive assessment tools, and short study durations.

##### 9.1.3.4. Therapeutic Decision-Making

Despite some divergence among different guidelines, all – including the present Guideline – consider individualized management to be essential, taking into account functional status, cognition, degree of frailty, patient expectations, comorbidities, HMOD, overall CV risk, polypharmacy, and treatment tolerability.^45,117,399^

When initiating treatment, frailty should be considered: for healthy older adults, the BP thresholds for treatment initiation are similar to those in younger adults (≥ 140/≥ 90 mm Hg), whereas in frail older patients, treatment should be individualized according to patient tolerability.^1,45^

#### 9.1.4. Blood Pressure Targets and Treatment

The therapeutic strategy in the elderly, particularly those over 80 years of age, cannot be uniform. Therefore, more important than chronological age when planning treatment are factors such as comorbidities, autonomy, functional status, and degree of frailty.^377,378^ No therapeutic intervention should be withheld or discontinued solely on the basis of age.

##### 9.1.4.1. Blood Pressure Targets in Older Adults

The SHEP trial,^40^ including older adults with isolated systolic hypertension, and the HYVE trial,^401,402^ including oldest-old patients (≥ 80 years), clearly demonstrated the benefits of BP reduction, with a significant decrease in CV outcomes and mortality, even among those with frailty.

The SPRINT Senior trial evaluated a cohort of 2,636 hypertensive patients aged 75 years or older and found that in the intensive SBP target group (< 120 mm Hg), there was a 34% reduction in the composite primary endpoint.^403^ Furthermore, in the SPRINT MIND substudy, patients assigned to the intensive treatment group (< 120 mm Hg) showed a smaller increase in cerebral white matter lesion volume and a lower incidence of cognitive decline.^404^

Finally, the STEP (Semaglutide Treatment Effect in People with Obesity) trial evaluated older hypertensive patients in China with SBP targets of 130–150 mmHg versus 110–130 mmHg. After 3.3 years of follow-up, the trial was stopped early owing to the clear benefit of intensive SBP treatment in reducing CV events.^133^ Recent meta-analyses have also shown that more intensive BP control in older hypertensive patients reduces the incidence of CV events without increasing the rate of adverse effects.^131,405^

Based on the evidence presented, these Guidelines recommend a target BP of < 130/80 mm Hg for the vast majority of older hypertensive patients. For frail older individuals, the oldest-old, or those with conditions that reduce life expectancy, BP should be lowered to the maximum level tolerated by the patient.

##### 9.1.4.2. Non-Pharmacological Treatment

All NPMs that apply to younger individuals (see Chapter 4) are also valid for older adults; however, greater caution is required, taking into account both the actual benefit and the potential risk of each.

Older people are more sensitive to salt, making sodium restriction more effective in this age group.^148^ Nevertheless, excessive salt reduction may lead to hyponatremia and loss of appetite, potentially resulting in malnutrition.

Potassium-rich diets should be encouraged,^406^ but greater caution is warranted due to the risk of hyperkalemia, given the frequent presence of CKD and the use of medications that reduce potassium excretion.

Aerobic and resistance physical exercise is essential for older adults and should be prescribed.^407,408^ In older adults, particularly those who are frail or sarcopenic, weight reduction without concomitant physical activity and adequate protein intake may result in loss of muscle mass and worsening of functional status.

Follow-up by a multidisciplinary team (see Chapters 6 and 13), health education, and the involvement of family members and/or caregivers are even more crucial for older adults.

##### 9.1.4.3. Pharmacological Treatment

When choosing antihypertensive medication(s) for older adults, consideration should be given to the high prevalence of comorbidities, specific contraindications, potential drug interactions, cost, as well as medication availability and clinical experience. It is prudent to initiate treatment with monotherapy or a low-dose combination and, if necessary, gradually increase the dose or add other antihypertensives, allowing a minimum interval of 2 weeks between adjustments.^409^

Details on when to prefer or avoid specific antihypertensive agents, as well as guidance on medication combinations, are provided in Chapter 7. Here, we highlight a few considerations specific to the older population.

Antihypertensive classes such as centrally acting agents, aldosterone antagonists, and direct vasodilators, as well as other invasive approaches targeting the sympathetic nervous system, should be regarded as exceptions rather than standard therapy for older patients.

The risk of falls in older adults may increase during the first weeks of treatment with thiazide DIUs and on the first day of treatment with other classes of medications.^410^

RCTs have shown that hypertension control reduces CV events without increasing the risk of OH or falls resulting in injuries.^257,411^ The benefits of antihypertensive therapy were similar even among those who presented with OH at baseline.^384^

##### 9.1.4.4. Polypharmacy and Adherence

Polypharmacy, defined as the regular use of five or more medications, becomes more frequent with advanced age.^411^ It is associated with a higher likelihood of adverse events, drug interactions, and reduced treatment adherence.^412^

Accordingly, it is recommended that, particularly in older patients on polypharmacy, each medication be periodically reviewed and potential adverse effects assessed,^413^ and that antihypertensive therapy involve the smallest possible number of daily pills. This can be achieved through the use of once-daily, fixed-dose combination antihypertensive medications, along with the promotion of NPMs.

Special attention should be given to medications that may contribute to OH or PPH, such as DIUs, sympatholytic agents, nitrates, and tricyclic antidepressants.

##### 9.1.4.5. Deintensification and Deprescribing

In certain clinical situations, it may be necessary to gradually reduce the dose or even discontinue antihypertensive therapy, such as: symptomatic hypotension, adverse reactions, persistent SBP well below target values detected both in and out of the medical office, adjustment of BP targets to less stringent levels (noting also that BP tends to decrease in very advanced age as a result of the progressive reduction in organ reserve and greater frailty), and palliative care at the end of life.^414,415^

A key point in the management of hypertension in older adults, and particularly in the oldest-old, is the careful monitoring of adverse events and treatment tolerability, with attention to atypical signs and symptoms. Short-term discontinuation of antihypertensive agents appears to be safe; however, no benefits have been demonstrated in terms of cognition or functional ability in activities of daily living.^414,41^^5^

**Table:** 

Key messages on hypertension in older patients
More than 60% of older patients have hypertension and multiple chronic diseases, with polypharmacy being common, increasing both cost and the risk of adverse events and drug interactions.^373,374^
Polypharmacy is associated with higher mortality and hospitalization rates in older patients.^416,417^
Most older adults present with isolated or predominantly elevated systolic blood pressure.
The diagnosis of hypertension in older adults requires awareness of some specific features, such as the auscultatory gap and pseudohypertension, with regular assessment for orthostatic hypotension and postprandial hypotension being essential.^379,380^
NPMs can be effective but require greater caution.^388^
Pharmacological treatment is similar to that in younger adults, but greater attention should be paid to tolerability, comorbidities, orthostatic and postprandial hypertension, and polypharmacy, all of which are more common in the older population.^389^
Clinical evaluation should involve a multidisciplinary team as well as the patient's caregivers and/or family members, which helps improve adherence rates and the likelihood of treatment success.
The loss of organ reserve in advanced age is associated with a gradual decline in BP and may warrant deintensification or even deprescription of one or more medications,^390^ which does not increase mortality and can reduce costs.^418^
In older patients receiving palliative care for advanced disease without a prognosis or with severe frailty, the main treatment goal is symptom control. Deprescription of antihypertensive agents should be considered.^391^

BP: blood pressure; NPMs: nonpharmacological measure.

**Table:** 

Recommendations for older patients	Strength of recommendation	Certainty of Evidence
Out-of-office BP monitoring (ABPM/HBPM) should be performed in older patients whenever possible at diagnosis, annually for treatment assessment, or when there is a change in therapeutic strategy.	WEAK	LOW
Functional, cognitive, nutritional, and social status should be considered when defining intrinsic capacity and/or the degree of frailty in older patients.	WEAK	LOW
A BP target < 130/80 mm Hg is recommended for most older patients.	STRONG	HIGH
It is recommended that, for frail older adults, the oldest-old, or those with conditions that compromise life expectancy, BP be lowered to the lowest tolerated level.	STRONG	HIGH
Beta-blockers are not recommended as initial monotherapy in older patients, except in the presence of specific comorbidities, in which they may even have a mandatory indication, such as HF or coronary syndromes.	STRONG	MODERATE
When choosing antihypertensive medication(s) for older adults, consideration should be given to the high prevalence of comorbidities, specific contraindications, potential drug interactions, cost, as well as medication availability and clinical experience.	STRONG	MODERATE
In older patients on polypharmacy, each medication should be periodically reviewed, adverse events should be assessed, and antihypertensive therapy should involve the smallest possible number of daily pills. This can be achieved through the use of once-daily, fixed-dose combination antihypertensive medications, along with the promotion of non-pharmacological measures.	STRONG	MODERATE

ABPM: ambulatory blood pressure monitoring; BP: blood pressure; HBPM: home blood pressure monitoring; HF: heart failure.

### 9.2. Hypertension in Children and Adolescents

Recent data indicate a global pooled prevalence of hypertension of 4.0% in individuals aged ≤ 19 years, with 4.0% classified as stage 1, 0.95% as stage 2, and 9.67% in the group previously categorized as elevated BP. This latter category, which corresponds to the prehypertension designation used in adults, will henceforth also be referred to as prehypertension in children and adolescents.^419^

In 2017, the staging of BP in children and adolescents based on BP percentiles (on the basis of sex, age, and height percentiles) was revised. These percentiles continue to apply to individuals under 13 years of age, while adult thresholds have been adopted starting at age 13.^44,420^ These changes streamlined the initial management of patients, increased the prevalence of diagnosed prehypertension and hypertension, improved sensitivity for early detection of HMOD in the pediatric age group,^421^ facilitated the transition from adolescent to adult care, and introduced ABPM for diagnostic confirmation and therapeutic monitoring of pediatric hypertension.^420^ Updates to BP staging based on ABPM in the pediatric population, following the same conceptual framework, were introduced in 2022.^422^ Conditions that warrant routine BP measurement in children under 3 years of age include prematurity, very low birth weight, intrauterine growth restriction, neonatal intensive care unit admission or umbilical artery catheterization, congenital heart disease (whether operated or not), CKD, recurrent urinary tract infections, hematuria or proteinuria, renal disease or urologic malformations, solid-organ transplant, malignancy or bone marrow transplant, DM, chronic use of medications known to raise BP, systemic diseases associated with hypertension (eg, neurofibromatosis, tuberous sclerosis, sickle cell disease), and evidence of intracranial hypertension.^420^

BP measurement should preferably be performed using the auscultatory method.^420^ However, oscillometric devices validated for pediatric use are useful in newborns and young children until they are able to cooperate with auscultatory BP and may also be used as a screening tool in older children and adolescents. Oscillometric BP measurement follows the same technical rules as the auscultatory method.^420^ A list of oscillometric devices validated for pediatric use is available at the following websites: https://www.stridebp.org/children-pdf/ (BHS); https://www.validatebp.org (US/AMA); https://hypertension.ca/bpdevices (Canada); https://www.stridebp.org/bp-monitors (STRIDE BP), a joint initiative of the European Society of Hypertension, International Society of Hypertension, and the World Hypertension League.

Tables 9.1, 9.2, 9.3, and 9.4 present, respectively, the updated definitions of normal BP, prehypertension, and stages 1 and 2 hypertension in children and adolescents, based on age, sex, and height percentiles; BP ranges for boys and girls by age and height percentiles;^420^ and updated definitions of normal BP, WCH, MH, and hypertension based on office and ABPM readings.^422,423^

**Table 9.1:** Updated definition of blood pressure by age group

Children aged 1 to 12 years	Children aged ≥ 13 years
Normal BP: < 90th percentile for age, sex, and height	Normal BP: < 120/< 80 mm Hg
Elevated BP (prehypertension): ≥ 90th to < 95th percentile for age, sex, and height or BP ≥ 120/80 mm Hg to < 95th percentile (whichever is lower)	Prehypertension: SBP 120 to 129/< 80 mm Hg
Stage 1 hypertension: ≥ 95th percentile to < 95th percentile for age, sex, and height + 12 mm Hg; or BP 130/80 to 139/89 mm Hg (whichever is lower)	Stage 1 hypertension: BP 130/80 up to 139/89 mm Hg
Stage 2 hypertension: ≥ 95th percentile + 12 mm Hg for age, sex, and height; or BP ≥ 140/90 mm Hg (whichever is lower)	Stage 2 hypertension: BP ≥ 140/90 mm Hg

BP: blood pressure. Adapted from Flynn et al.^420^

**Table 9.2:** Blood pressure ranges for boys by age and height percentiles

Age (years)	BP percentiles	Systolic blood pressure (mm Hg) Height percentile or measured height (cm)							Diastolic blood pressure (mm Hg) Height percentile or measured height (cm)						
		5%	10%	25%	50%	75%	90%	95%	5%	10%	25%	50%	75%	90%	95%
1	Height (cm)	77,2	78,3	80,2	82,4	84,6	86,7	87,9	77,2	78.3	80,2	82,4	84,6	86,7	87,9
	50P	85	85	86	86	87	88	88	40	40	40	41	41	42	42
	90P	98	99	99	100	100	101	101	52	52	53	53	54	54	54
	95P	102	102	103	103	104	105	105	54	54	55	55	56	57	57
	95P + 12 mm Hg	114	114	115	115	116	117	117	66	66	67	67	68	69	69
2	Height (cm)	86,1	87,4	89,6	92,1	94,7	97,1	98,5	86,1	87,4	89,6	92,1	94,7	97,1	98,5
	50P	87	87	88	89	89	90	91	43	43	44	44	45	46	46
	90P	100	100	101	102	103	103	104	55	55	56	56	57	58	58
	95P	104	105	105	106	107	107	108	57	58	58	59	60	61	61
	95P + 12 mm Hg	116	117	117	118	119	119	120	69	70	70	71	72	73	73
3	Height (cm)	92,5	93,9	96,3	89	101,6	104,3	105,8	92,5	93,9	96,3	99	101,8	104,3	105,8
	50P	88	89	89	90	91	92	92	45	46	46	47	48	49	49
	90P	101	102	102	103	104	105	105	58	58	59	59	60	61	61
	95P	106	106	107	107	108	109	109	60	61	61	62	63	64	64
	95P + 12 mm Hg	118	118	119	119	120	121	121	72	73	73	74	75	76	76
4	Height (cm)	98,5	100,2	102,9	105,9	108,9	111,5	113,2	98,5	100,2	102,9	105,9	108,9	111,5	113,2
	50P	90	90	91	92	93	94	94	48	49	49	50	51	52	52
	90P	102	103	104	105	105	106	107	60	61	62	62	63	64	64
	95P	107	107	108	108	109	110	110	63	64	65	66	67	67	68
	95P + 12 mm Hg	119	119	120	120	121	122	122	75	76	77	78	79	79	80
5	Height (cm)	104,4	106,2	109,1	112,4	115,7	118,6	120,3	104,4	106,2	109,1	112,4	115,7	118,6	120,3
	50P	91	92	93	94	95	96	96	51	51	52	53	54	55	55
	90P	103	104	105	106	107	108	108	63	64	65	65	66	67	67
	95P	107	108	109	109	110	111	112	66	67	68	69	70	70	71
	95P + 12 mm Hg	119	120	121	121	122	123	124	78	79	80	81	82	82	83
6	Height (cm)	110,3	112,2	115,3	118,9	122,4	125,6	127,5	110,3	112,2	115,3	118,9	122,4	125,6	127,5
	50P	93	93	94	95	96	97	98	54	54	55	56	57	57	58
	90P	105	105	106	107	109	110	110	66	66	67	68	68	69	69
	95P	108	109	110	111	112	113	114	69	70	70	71	72	72	73
	95P + 12 mm Hg	120	121	122	123	124	125	126	81	82	82	83	84	84	85
7	Height (cm)	116,1	118	121,4	125,1	128,9	132,4	134,5	116,1	118	121,4	125,1	128,9	132,4	134,5
	50P	94	94	95	97	98	98	99	56	56	57	58	58	59	59
	90P	106	107	108	109	110	111	111	68	68	69	70	70	71	71
	95P	110	110	111	112	114	115	116	71	71	72	73	73	74	74
	95P + 12 mm Hg	122	122	123	124	126	127	128	83	83	84	85	85	86	86
8	Height (cm)	121,4	123,5	127	131	135,1	138,8	141	121,4	123,5	127	131	135,1	138,8	141
	50P	95	96	97	98	99	99	100	57	57	58	59	59	60	60
	90P	107	108	109	110	111	112	112	67	70	70	71	72	72	73
	95P	111	112	112	114	115	116	117	72	73	73	74	75	75	75
	95P + 12 mm Hg	123	124	124	126	127	128	129	84	85	85	86	87	87	87
9	Height (cm)	126	128,3	132,1	136,3	140,7	144,7	147,1	126	128,3	132,1	136,3	140,7	144,7	147,1
	50P	96	97	98	99	100	101	101	57	58	59	60	61	62	62
	90P	107	108	109	110	112	113	114	70	71	72	73	74	74	74
	95P	112	112	113	115	116	118	119	74	74	75	76	76	77	77
	95P + 12 mm Hg	124	124	125	127	128	130	131	86	86	87	88	88	89	89
10	Height (cm)	130,2	132,7	136,7	141,3	145,9	150,1	152,7	130,2	132,7	136,7	141,3	145,9	150,1	152,7
	50P	97	98	99	100	100	102	103	59	60	61	62	63	63	64
	90P	108	109	11¹	112	113	115	116	72	73	74	74	75	75	76
	95P	112	113	114	116	118	120	121	76	76	77	77	78	78	78
	95P + 12 mm Hg	124	125	126	128	130	132	133	88	88	89	89	90	90	90
11	Height (cm)	134,7	137,3	141,5	146,4	151,3	155,8	158,6	134,7	137,3	141,5	146,4	151,3	155,8	158,6
	50P	99	99	101	102	103	104	106	61	61	62	63	63	63	63
	90P	110	111	112	114	116	117	118	74	74	75	75	75	76	76
	95P	114	114	116	118	120	123	124	77	78	78	78	78	78	78
	95P + 12 mm Hg	126	126	128	130	132	135	136	89	90	90	90	90	90	90
12	Height (cm)	140,3	143	147,5	152,7	157,9	162,6	165,5	140,3	143	147,5	152,7	157,9	162,6	165,5
	50P	101	101	102	104	106	108	109	61	62	62	62	62	63	63
	90P	113	114	115	117	119	121	122	75	75	75	75	75	76	76
	95P	116	117	118	121	124	126	128	78	78	78	78	78	79	79
	95P + 12 mm Hg	128	129	130	133	136	138	140	90	90	90	90	90	91	91
13	Height (cm)	147	150	154,9	160,3	165,7	170,5	173,4	147	150	154,9	160,3	165,7	170,5	173,4
	50P	103	104	105	108	110	111	112	61	60	61	62	63	64	65
	90P	115	116	118	121	124	126	126	74	74	74	75	76	77	77
	95P	119	120	122	125	128	130	131	78	78	78	78	80	81	81
	95P + 12 mm Hg	131	132	134	137	140	142	143	90	90	90	90	92	93	93
14	Height (cm)	153,8	156,9	162	167,5	172,7	177,4	180,1	153,8	156,9	162	167,5	172,7	177,4	180,1
	50P	105	106	109	111	112	113	113	60	60	62	64	65	66	67
	90P	119	120	123	126	127	128	129	74	74	75	77	78	79	80
	95P	123	125	127	130	132	133	134	77	78	79	81	82	83	84
	95P + 12 mm Hg	135	137	139	142	144	145	146	89	89	91	93	94	95	96
15	Height (cm)	159	162	166,9	172,2	177,2	181,6	184,2	159	162	166,9	172,2	177,2	181,6	184,2
	50P	108	110	112	113	114	114	114	61	62	64	65	66	67	68
	90P	123	124	126	128	129	130	130	75	76	78	79	80	81	81
	95P	127	129	131	132	134	135	135	78	79	81	83	84	85	85
	95P + 12 mm Hg	139	141	143	144	146	147	147	90	91	93	95	96	97	97
16	Height (cm)	162,1	165	169,6	174,6	179,5	183,8	186,4	162,1	165	169,6	174,6	179,5	183,8	186,4
	50P	111	112	114	115	115	116	116	63	64	66	67	68	69	69
	90P	126	127	128	129	131	131	132	77	78	79	80	81	82	82
	95P	130	131	133	134	135	136	137	80	81	83	84	85	86	86
	95P + 12 mm Hg	142	143	145	146	147	148	149	92	93	95	96	97	98	98
17	Height (cm)	163,8	166,5	170,9	175,8	180,7	184,9	187,5	163,8	166,5	170,9	175,8	180,7	184,9	187,5
	50P	114	115	116	117	117	118	118	65	66	67	68	69	70	70
	90P	128	129	130	131	132	133	134	78	79	80	81	82	82	83
	95P	132	133	134	135	137	138	138	81	82	84	85	86	86	87
	95P + 12 mm Hg	144	145	146	147	149	150	150	93	94	96	97	98	98	99

Adapted from Flynn et al.^420^

**Table 9.3:** Blood pressure ranges for girls by age and height percentiles

Age (years)	BP percentiles	Systolic blood pressure (mm Hg) Height percentile or measured height (cm)							Diastolic blood pressure (mm Hg) Height percentile or measured height (cm)						
		5%	10%	25%	50%	75%	90%	95%	5%	10%	25%	50%	75%	90%	95%
1	Height (cm)	75,4	76,6	78,6	80,8	83	84,9	86,1	75,4	76,6	78,6	80,8	83	84,9	86,1
	50P	84	85	86	86	87	88	88	41	42	42	43	44	45	46
	90P	98	99	99	100	101	102	102	54	55	56	56	57	58	58
	95P	101	102	102	103	104	105	105	59	59	60	60	61	62	62
	95P + 12 mm Hg	113	114	114	115	116	117	117	71	71	72	72	73	74	74
2	Height (cm)	84,9	86,3	88,6	91,1	93,7	96	97,4	84,3	86,3	88,6	91,1	93,7	96	97,4
	50P	84	87	88	89	90	91	91	45	46	47	48	49	50	51
	90P	101	101	102	103	104	105	106	58	58	59	60	61	62	62
	95P	104	105	106	106	107	108	109	62	63	63	64	65	66	66
	95P + 12 mm Hg	116	117	118	118	119	120	121	75	75	75	76	77	78	78
3	Height (cm)	91	92,4	94,9	97,6	100,5	103,1	104,6	91	92,4	94,9	97,6	100,5	103,1	104,6
	50P	88	89	89	90	91	92	93	48	48	49	50	51	53	53
	90P	102	103	104	104	105	106	107	60	61	61	62	63	64	65
	95P	106	106	107	108	109	110	110	64	65	65	66	67	68	69
	95P + 12 mm Hg	118	118	119	120	121	122	122	76	77	77	78	79	80	81
4	Height (cm)	97,2	98,8	101,4	104,5	107,6	110,5	112,2	97,2	98,8	101,4	104,5	107,6	110,5	112,2
	50P	89	90	91	92	93	94	94	50	51	51	53	54	55	55
	90P	103	104	105	106	107	108	108	62	63	64	65	66	67	67
	95P	107	108	109	109	110	111	112	66	67	68	69	70	70	71
	95P + 12 mm Hg	119	120	121	121	122	123	124	78	79	80	81	82	82	83
5	Height (cm)	103,6	105,3	108,2	111,5	114,9	118,1	120	103,6	105,3	108,2	111,5	114,9	118,1	120
	50P	90	91	92	93	94	95	96	52	52	53	55	56	57	57
	90P	104	105	106	107	108	109	110	64	65	66	67	68	69	70
	95P	108	109	109	110	111	112	113	68	69	70	71	72	73	73
	95P + 12 mm Hg	120	121	121	122	123	124	125	80	81	82	83	84	85	85
6	Height (cm)	110	111,8	114	118,4	122,1	125,6	127,7	110	111,8	114,9	118,4	122,1	125,6	127,7
	50P	92	92	93	94	96	97	97	54	54	55	56	57	58	59
	90P	105	106	107	108	109	110	111	67	67	68	69	70	71	71
	95P	109	109	110	111	112	113	114	70	71	72	72	73	74	74
	95P + 12 mm Hg	121	121	122	123	124	125	126	82	83	84	84	85	86	86
7	Height (cm)	115,9	117,8	121,1	124,9	128,8	132,5	134,7	115,9	117,8	121,1	124,9	128,8	132,5	134,7
	50P	92	93	94	95	97	98	99	55	54	56	57	58	59	60
	90P	106	106	107	109	110	111	112	68	68	69	70	71	72	72
	95P	109	110	111	112	113	114	115	72	72	73	73	74	74	75
	95P + 12 mm Hg	121	122	123	124	125	126	127	84	84	85	85	86	86	87
8	Height (cm)	121	123	126,5	130,6	134,7	138,8	140,9	121	123	126,5	130,6	134,7	138,5	140,9
	50P	93	94	95	97	98	99	100	56	56	57	59	60	61	61
	90P	107	107	108	110	111	112	113	69	70	71	72	72	73	73
	95P	110	111	112	113	115	116	117	72	73	74	74	75	75	75
	95P + 12 mm Hg	122	123	124	125	127	128	129	84	85	86	86	87	87	87
9	Height (cm)	125,3	127,6	131,3	135,6	140,1	144,1	146,6	125,3	127,6	131,3	135,6	140,1	144,1	146,6
	50P	95	95	97	98	99	100	101	57	58	59	60	60	61	61
	90P	108	108	109	111	112	113	114	71	71	72	73	73	73	73
	95P	112	112	113	114	116	117	118	74	74	75	75	75	75	75
	95P + 12 mm Hg	124	124	125	126	128	129	130	86	86	87	87	87	87	87
10	Height (cm)	129,7	132,2	136,3	141	145,8	150,2	152,8	129,7	132,2	136,3	141	145,8	150,2	152,8
	50P	96	97	98	99	101	102	103	58	59	59	60	61	61	61
	90P	109	110	111	112	113	115	116	72	73	73	73	73	73	73
	95P	113	114	114	116	117	119	120	75	75	76	76	76	76	76
	95P + 12 mm Hg	125	126	126	128	129	131	132	87	87	88	88	88	88	88
11	Height (cm)	135,6	138,3	142,8	147,8	152,8	157,3	160	135,6	138,3	142,8	147,8	152,8	157,3	160
	50P	98	99	101	102	104	105	106	60	60	60	61	62	63	64
	90P	111	112	113	114	116	118	120	74	74	74	74	74	75	75
	95P	115	116	117	118	120	123	124	76	77	77	77	77	77	77
	95P + 12 mm Hg	127	128	129	130	132	135	136	88	89	89	89	89	89	89
12	Height (cm)	142,8	145,5	149,9	154,8	159,6	163,8	166,4	142,8	145,5	149,9	154,8	159,6	163,8	166,4
	50P	102	102	104	105	107	108	108	61	61	61	62	64	65	65
	90P	114	115	116	118	120	122	122	75	75	75	75	76	76	76
	95P	118	119	120	122	124	125	126	78	78	78	78	79	79	79
	95P + 12 mm Hg	130	131	132	134	136	137	138	90	90	90	90	91	91	91
13	Height (cm)	148,1	150,6	154,7	159,2	163,7	167,8	170,2	148,1	150,6	154,7	159,2	163,7	167,8	170,2
	50P	104	105	106	107	108	108	109	62	62	63	64	65	65	65
	90P	116	117	119	121	122	123	123	75	75	75	76	76	76	76
	95P	121	122	123	124	126	126	127	79	79	79	79	80	80	81
	95P + 12 mm Hg	133	134	135	136	138	138	139	91	91	91	91	92	92	93
14	Height (cm)	150,6	153	156,9	161,3	165,7	169,7	172,1	150,6	153	156,9	161,3	165,7	169,7	172,1
	50P	105	106	107	108	109	109	109	63	63	64	65	66	66	66
	90P	118	118	120	122	123	123	123	76	76	76	76	77	77	77
	95P	123	123	124	125	126	127	127	80	80	80	80	81	81	82
	95P + 12 mm Hg	135	135	136	137	139	139	139	92	92	92	92	93	93	94
15	Height (cm)	151,7	154	157,9	162,3	166,7	170,6	173	151,7	154	157,9	162,3	166,7	170,6	173
	50P	105	106	107	108	109	109	109	64	64	64	65	66	67	67
	90P	118	119	121	122	123	123	124	76	76	76	77	77	78	78
	95P	124	124	125	126	127	127	128	80	80	80	81	82	82	82
	95P + 12 mm Hg	136	136	137	138	139	139	140	92	92	92	93	94	94	94
16	Height (cm)	152,1	154,5	158,4	162,8	167,1	171,1	173,4	152,1	154,5	158,4	162,8	167,1	171,1	173,4
	50P	106	107	108	109	109	110	110	64	64	65	66	66	67	67
	90P	119	120	122	123	124	124	124	76	76	76	77	78	78	78
	95P	124	125	125	127	127	128	128	80	80	80	81	82	82	82
	95P + 12 mm Hg	136	137	137	139	139	140	140	92	92	92	93	94	94	94
17	Height (cm)	152,4	154,7	158,7	163	167,4	171,3	173,7	152,4	154,7	158,7	163	167,4	171,3	173,7
	50P	107	108	109	110	110	110	111	64	64	65	66	66	66	67
	90P	120	121	123	124	124	125	125	76	76	77	77	78	78	78
	95P	125	125	126	127	128	128	128	80	80	80	81	82	82	82
	95P + 12 mm Hg	137	137	138	139	140	140	140	92	92	92	93	94	94	94

Adapted from Flynn et al.^420^

**Table 9.4:** Office BP and ABPM ranges for hypertension phenotypes in children and adolescent^422^

	Office systolic or diastolic BP		ABPM	
	< 13 years	≥ 13 years	< 13 years	≥ 13 years
Normal BP	< 95th percentile	< 130/80	< 95th percentile OR adolescent cut points*	< 125/75 mm Hg 24-h AND< 130/80 mm Hg wake E<110/65 mm Hg sleep
White-coat hypertension	≥ 95th percentile	≥ 130/80		
Masked hypertension	< 95th percentile	< 130/80	≥ 95th percentile OR adolescent cut points*	≥ 125/75 mm Hg 24-h OR ≥ 130/80 mm Hg wake OR≥ 110/65 mm Hg sleep
Ambulatory hypertension	≥ 95th percentile	≥ 130/80		

ABPM: ambulatory blood pressure monitoring; BP: blood pressure.

** Including 24 h, wake, and sleep BP.

In neonates, BP increases with birth weight, gestational age, and postnatal age. Small observational studies using different methods have led to inconsistent proposals for BP standardization in neonates.^424^ It is suggested to use the estimated BP values after 2 weeks of life in neonates with postconceptional ages ranging from 26 to 44 weeks, as shown in Table 9.5,^425^ and the BP values for the first year of life, as presented in Tables 9.2, 9.3.^426^

**Tables 9.5:** Estimated systolic, diastolic, and mean blood pressure (mm Hg) values after 2 weeks of age in infants from 26 to 44 weeks postconceptional age^425^

Postconceptional age	50^th^ percentile	95^th^ percentile	99^th^ percentile
**44 weeks**			
SBP	88	105	110
DBP	50	68	73
MAP	63	80	85
**42 weeks**			
SBP	85	98	102
DBP	50	65	70
MAP	62	76	81
**40 weeks**			
SBP	80	95	100
DBP	50	65	70
MAP	60	75	80
**38 weeks**			
SBP	77	92	97
DBP	50	65	70
MAP	59	74	79
**36 weeks**			
SBP	72	87	92
DBP	50	65	70
MAP	57	72	71
**34 weeks**			
SBP	70	85	90
DBP	40	55	60
MAP	50	65	70
**32 weeks**			
SBP	68	83	88
DBP	40	55	60
MAP	50	65	70
**30 weeks**			
SBP	65	80	85
DBP	40	55	60
MAP	48	65	68
**28 weeks**			
SBP	60	75	80
DBP	38	50	54
MAP	45	58	63
**26 weeks**			
SBP	55	72	77
DBP	30	50	56
MAP	38	57	63

SBP: systolic blood pressure; DBP: diastolic blood pressure; MAP: mean arterial pressure. Adapted from Dionne et al.^425^

The diagnosis of pediatric hypertension is based on the confirmation of BP values ≥ the 95th percentile on three separate occasions, using the auscultatory method.^398^

A detailed history and physical examination, covering the perinatal period and the biopsychosocial environment, are essential for guiding further investigations and determining the etiology, which may be primary or secondary. SH is more likely in younger children and in those with markedly elevated BP values, whereas primary hypertension is more prevalent among adolescents and in individuals with overweight or obesity.^420^

A detailed investigation of SH is generally unnecessary in children aged ≥ 6 years with overweight/obesity, a positive family history of hypertension, and no clinical findings suggestive of secondary causes on history and physical examination.^420^ However, screening for proteinuria is mandatory in cases of CKD and hypertension.^420^

ABPM is indicated for confirmation of hypertension in children and adolescents with office BP values consistent with prehypertension for at least 1 year or consistent with stage 1 hypertension in three outpatient visits.^420^ The procedure should follow standardized techniques, using devices validated for pediatric use and based on normative pediatric values.^420,422^

Even in asymptomatic cases, uncontrolled pediatric hypertension may lead to increased carotid intima-media thickness, reduced arterial distensibility, retinal arteriolar narrowing,^427^ and LVH, which is present in up to 40% of pediatric hypertension cases at the time of diagnosis.^428^ Prospective long-term cohort studies have shown that CV risk factors present during childhood are associated with HMOD in adulthood. Evidence suggests that the reduction of body weight and alcohol intake, increased consumption of vegetables, regular physical activity, and reduced sedentary behavior from childhood into adulthood can help resolve hypertension during the transition from childhood to adult life and minimize adverse cardiometabolic outcomes in nonobese adults.^429-432^ These findings support the notion that pediatric hypertension should be considered an early manifestation of CVD.

The goals of pediatric hypertension treatment include achieving BP control, minimizing the risk of HMOD, and reducing the likelihood of hypertension and CV complications in adulthood. Treatment planning depends on the etiology of hypertension, CV risk, presence of comorbidities, and evidence of HMOD.^420^ The therapeutic target is to lower SBP and DBP below the 90th percentile in children, or below 130/80 mm Hg in adolescents aged 13 years or older. Children or adolescents with CKD should be treated to lower 24-h mean arterial pressure (MAP) to < 50th percentile by ABPM.^420^

NPMs are indicated for all patients with BP values ≥ 90th percentile, or ≥130/80 mm Hg in adolescents aged ≥ 13 years old.^420^ Some measures include weight loss, regular physical exercise, dietary modifications, and stress management. The combination of these four measures has been shown to produce synergistic benefits.^433^ The DASH diet is particularly recommended in obesity-associated hypertension.^420,434^ Stress-reduction strategies should also be part of the management plan.^420^

Pharmacological treatment should be initiated with an ACEI, ARB, long-acting CCB, or thiazide diuretic in adolescents ≥13 years of age with symptomatic hypertension, presence of HMOD, stage 2 hypertension without an identifiable modifiable cause, or persistent hypertension unresponsive to NPMs when BP is ≥95th percentile or ≥130/80 mm Hg.^420^ In cases of SH due to CKD or DM, and/or in the presence of proteinuria, treatment with an ACEI or ARB is recommended.

Echocardiography, using the criteria defined for each age group,^398^ is suggested prior to the initiation of pharmacological therapy and may be repeated after 6 to 12 months, as necessary.^420^

Antihypertensive therapy should begin with a single agent at a low dose, with titration every 2 to 4 weeks until the therapeutic target is achieved. In cases of SH, the choice of antihypertensive medication should be guided by the underlying pathophysiological mechanism and the patient's comorbidities.^420^ The frequency of clinical follow-up depends on the specific needs of each patient. Follow-up ABPM is indicated when hypertension remains uncontrolled or in cases with a risk of MH, such as in the late postoperative period after repair of aortic coarctation. In patients with CKD, annual ABPM is mandatory to rule out MH. HBPM may also be used as an adjunct tool for assessing BP control.^420^

**Table:** 

Key messages on hypertension in children and adolescents
The global pooled prevalence of hypertension in individuals aged ≤ 19 years is 4.0%.
Secondary hypertension is more likely in younger children and in those with markedly elevated BP values. Primary hypertension is more prevalent among adolescents and in individuals with overweight or obesity.
Reduction of body weight and alcohol intake, increased consumption of vegetables, regular physical activity, and reduced sedentary behavior from childhood into adulthood can help resolve hypertension during the transition from childhood to adult life and minimize adverse cardiometabolic outcomes in non-obese adults.

BP: blood pressure.

**Table:** 

Recommendations for children and adolescents	Strength of recommendation	Certainty of evidence
BP should be measured annually in children starting at 3 years of age.	STRONG	LOW
It is recommended to investigate suspected WCH and secondary hypertension in children born prematurely and those with CKD, DM, OSA, obesity, solid-organ transplant, renovascular hypertension, and after aortic coarctation repair.	STRONG	MODERATE
The diagnosis of hypertension in children or adolescents up to 13 years of age should be based on the confirmation of BP values ≥95th percentile according to height percentiles; for adolescents ≥ 13 years of age, it should be based on the confirmation of BP values ≥ 130/80 mm Hg on three different visits using the auscultatory method.	STRONG	LOW
ABPM is recommended for confirmation of hypertension in children and adolescents with office BP values consistent with prehypertension for at least 1 year or consistent with stage 1 hypertension in three outpatient visits.	STRONG	MODERATE
In children with CKD, ABPM is recommended annually to rule out MH.	WEAK	LOW
Screening for proteinuria is recommended in children with CKD and hypertension.	STRONG	MODERATE
The therapeutic target is BP < 90th percentile in children and < 130/80 mm Hg in adolescents aged ≥ 13 years.	WEAK	LOW
The DASH diet is recommended for children and adolescents, especially in cases of obesity-associated hypertension, along with stress management strategies.	NEUTRAL	LOW
In children with BP ≥ 95th percentile or in adolescents ≥ 13 years with BP ≥ 130/80 mm Hg and symptomatic hypertension, HMOD, stage 2 hypertension without an identifiable modifiable cause, or persistent hypertension unresponsive to NPMs, it is recommended to initiate pharmacological treatment with an ACE inhibitor, ARB, long-acting CCB, or thiazide diuretic.	STRONG	LOW
In cases of secondary hypertension due to CKD or DM, and/or presence of proteinuria, it is recommended to initiate treatment an ACE inhibitor or ARB.	STRONG	MODERATE

ABPM: ambulatory blood pressure monitoring; ACE: angiotensin-converting enzyme; ARB: angiotensin receptor blocker; BP: blood pressure; CCB: calcium channel blocker; CKD: chronic kidney disease; DASH: Dietary Approaches to Stop Hypertension; DM: diabetes mellitus; MH: masked hypertension; NPMs: non-pharmacological measures; OSA: obstructive sleep apnea; HMOD: hypertension-mediated organ damage; WCH: white-coat hypertension.

## 10. Hypertension in Women

### 10.1. Hypertension in Young Women and During Menopause

#### 10.1.1. Prevalence

Although the prevalence is similar,^7,45^ the life trajectory of SBP and DBP differ between men and women. In women, the age-related increase in BP progresses more rapidly and begins in young adulthood.^435^ These sex-based differences in BP development over the life course and the age-related hypertension may be explained by different BP regulatory mechanisms, likely influenced by sex- and gender-specific hormonal factors.^45^ After the age of 60, women tend to have higher BP levels and a greater prevalence of hypertension than men.^1^ The frequency of hypertension increases with age, reaching 61.5% in men and 68.0% in women aged 65 years or older.^45^

#### 10.1.2. Factors Associated with Hypertension in Women

Women present with specific risk factors associated with hypertension, including those related to social determinants and biological factors.^436^ Notable among these are menopause, polycystic ovary syndrome (PCOS), the use of oral contraception pills (OCPs), and hypertensive disorders of pregnancy. Traditional risk factors also affect women differently, such as aging, obesity, and physical inactivity, as well as emerging factors such as autoimmune diseases, chronic stress, and environmental and psychosocial influences. In young women, certain secondary causes of hypertension, such as renal artery obstruction due to fibromuscular dysplasia, are more prevalent than in men.^437^

##### 10.1.2.1. Young Women and Oral Contraceptives

The association between the use of combined oral contraceptives (COCs) and hypertension has been studied, and a meta-analysis including 24 studies with a total of 270,284 participants reported a relative risk of 1.47 (95% CI, 1.25–1.73) for hypertension among users with longer vs. shorter duration of COC use, with a linear dose-response relationship showing a 13% increased risk (relative risk 1.13; 95% CI, 1.03–1.25) for every 5-year increment in COC use.^438^

OCPs containing both estrogen and progestin are more likely to induce hypertension, whereas progestin-only contraceptives and formulations with lower estrogen doses are associated with smaller BP increases.^436,439,440^ The use of drospirenone – a progestin with MRA properties – combined with ethinylestradiol has been shown to reduce SBP by 1-4 mm Hg, although it is associated with an increased risk of venous thromboembolism.^436^

It is recommended to monitor BP in young women before prescribing OCPs and, after initiation, to emphasize the importance of regular BP measurements (every 6 months), including home BP monitoring and the management of other CV risk factors.^436^ In cases of persistently elevated SBP (≥ 160 mm Hg) and/or DBP (≥ 100 mm Hg), alternative contraceptive methods or discontinuation of OCPs should be considered.^436^

The investigation of secondary causes of hypertension in young women is essential. Among the main causes are obesity, PCOS, OSA, coarctation of the aorta, autoimmune diseases, endocrine disorders, and primary nephropathies.^437^

For the treatment of hypertension, ACE inhibitors and ARBs should not be prescribed to women with childbearing potential due to their teratogenic risks in case of pregnancy, except in mandatory indications (eg, HF) and when reliable contraceptive measures are in place.^436^ Preferably, treatment at this stage should be initiated with CCBs or BBs, particularly those with associated vasodilatory properties.^1^

##### 10.1.2.2. Hypertension, Sexual Function, and Fertility in Women

Hypertension can negatively impact women's reproductive capacity, although the mechanisms by which it affects reproductive health and function remain to be fully elucidated. Hypertension may impair vascular health in the uterus and ovaries, compromising fertility.^441^

In addition, women with hypertension may experience sexual dysfunction due to alterations in clitoral and vaginal vasculature, reduced blood flow in the pelvic region, and thinning of the vaginal wall and clitoral smooth muscle cells, resulting in vaginal dryness.^441^ Other reported complications include dyspareunia, anorgasmia, adverse effects of the arousal phase, and sexual reluctance.^441^ Hypertension may also lead to fibrosis of the clitoris and the vaginal wall due to lower nitric oxide levels and reduced blood flow in the pelvic region.^441^

##### 10.1.2.3. Menopause

Menopause is associated with a decline in estradiol levels and an altered androgen/estrogen ratio.^439^ These hormonal changes can lead to weight gain, which in turn increases sympathetic nervous system activity and activation of the RAAS. This cascade results in elevated oxidative stress, endothelial dysfunction, reduced nitric oxide availability, and increased endothelin levels.^435,439,442^ Collectively, these mechanisms increase sensitivity to salt and promote renal vasoconstriction and the development of hypertension.^442^ In addition, postmenopausal women experience increased aortic stiffness due to proliferation of smooth muscle cells, collagen accumulation, and elevated levels of vasoconstrictor molecules in the arterial wall as a result of the lack of estrogen's protective effects, which may contribute to isolated systolic hypertension.^439^

Postmenopausal women with hypertension undergo a cardiometabolic transition characterized by a higher incidence of LVH and a greater risk of developing diastolic dysfunction compared to younger adult women.^439^

For pharmacological treatment during and after menopause, it is recommended to initiate therapy with a combination of an RAS inhibitor and a CCB or a thiazide DIU. The latter may offer additional benefits by helping prevent osteoporosis in women.^436^

When menopausal hormone therapy (MHT) is indicated, BP should be monitored from the outset, and if adequate control is not achieved, the therapy should be discontinued. Women with controlled hypertension and moderate to severe vasomotor symptoms may use MHT via any route. However, in the presence of obesity, dyslipidemia, DM, or metabolic syndrome, transdermal estrogen therapy (via gel or patches) is preferred.^439^

##### 10.1.2.4. Polycystic Ovary Syndrome

PCOS, a prevalent endocrine and metabolic condition among women of reproductive age, is a heterogeneous disorder that is associated with increased CV risk.^437,443^ Previous studies have shown that nearly all CV risk factors, including hypertension, obesity, insulin resistance, DM, atherogenic dyslipidemia, and metabolic syndrome, are more prevalent in patients with PCOS.^443^ These risk factors contribute to the development of early CV events in young women with PCOS. Androgen excess in PCOS is clearly associated with an increased prevalence of cardiometabolic disorders.^437^ Although studies assessing the association between PCOS and hypertension have yielded conflicting results, recent meta-analyses have demonstrated a strong association between PCOS and elevated BP in young women.^443,444^

A systematic review including data from three retrospective and two cross-sectional studies found that women with PCOS had a higher risk of developing metabolic disturbances, including hypertension, obesity, dyslipidemia, insulin resistance, and DM.^444^ Hyperandrogenemia was associated with elevated BP independently of age, insulin resistance, obesity, and dyslipidemia. Another meta-analysis reported a relative risk of 1.7 for hypertension in young women with PCOS, while in postmenopausal women, the presence of PCOS did not increase the risk of hypertension.^443^

#### 10.1.3. Target-Organ Damage and Comorbidities in Women

Women have smaller aortic root dimensions than men, even after adjusting for body size. Greater arterial stiffness has also been documented in women, particularly in the ascending aorta. Arterial stiffness is less modifiable by antihypertensive therapy in women than in men.^442^ Regarding the heart, hypertensive LVH is more prevalent and less modifiable by antihypertensive treatment in women than in men. Women with hypertension more frequently develop AF and HFpEF.^442^ In terms of renal outcomes, the overall prevalence of CKD is higher in women (51.7%) than in men, according to the NHANES study, with a prevalence 20 times higher among women over the age of 65.^445^ However, a recent meta-analysis involving more than 2 million individuals found that the incidence of hypertension-related CKD was lower in women than in men.^446^

It is recommended that women with hypertension undergo annual monitoring of renal function, including albuminuria, when possible.^436^ In addition, hypertensive women using hormone replacement therapy show a significant increase in albuminuria.

Women with both CKD and hypertension are more vulnerable to the effects of fluid overload on the left ventricle; therefore, strict maintenance of dry weight goal is essential.^436^

Hypertension is the most important risk factor for MI, HFpEF, stroke, cognitive decline, and PAD in women. CVD risk in women begins at brachial SBP levels approximately 10 mm Hg lower than in men.^447^

#### 10.1.4. Treatment of Hypertension in Women

NPMs are strongly recommended as a first-line strategy for BP control in women.^442^ Postmenopausal women with hypertension tend to be more sensitive to salt; therefore, reduction of sodium intake may provide significant BP-lowering benefits.

In the DASH study, dietary sodium restriction led to significant BP reductions in women only.^448,449^ Regarding physical activity, a meta-analysis of 93 RCTs investigating the impact of aerobic exercise on BP found that exercise produced greater BP reductions in men compared women.^450^

As for pharmacological treatment, the absorption, distribution, metabolism, and excretion of antihypertensive medications differ between women and men, probably due to the influence of sex hormones on absorption transporters (such as P-glycoprotein), distribution volume, cytochrome P450 activity, and renal clearance.^440^ However, these differences do not warrant sex-specific dosing of antihypertensive medications, but they may help explain the higher frequency of adverse effects observed in women, particularly during menopause.^439^ Common adverse effects include ACE inhibitor-induced cough, ankle edema with CCBs, and hypokalemia and hyponatremia with DIUs. These side effects may contribute to lower adherence to hypertension treatment among postmenopausal women.^439,451^

Moreover, women tend to achieve poorer BP control than men. In a study conducted in Sweden, women with hypertension had higher BP levels, received less antihypertensive treatment, and exhibited poorer control compared to men. Female sex was identified as a predictor of less intensive antihypertensive therapy compared to male sex. ^452^

Women and gender-specific aspects of hypertension are understudied, and the existing evidence is poorly translated into clinical guidelines.^453^ Some meta-analyses have shown that BP reductions achieved with treatment are as important (in terms of benefits) in women as in men, and reductions in events occurred similarly in men and women.^454,455^ A meta-analysis of 31 RCTs including 103,268 men and 87,349 women found that reductions in BP and incidence of CV events were comparable for men and women when treated with regimens based on ACE inhibitors, ARBs, CCBs, DIUs, or BBs.^454^ Similarly, another meta-analysis of 40 RCTs involving 152,379 patients found that no class of medications (ACEIs, ARBs, CCBs, or BBs) was significantly superior to thiazide DIUs as a first-line therapy for any outcome (all-cause mortality, CV mortality, HF, or stroke) in either women or men when analyzed separately.^455^

The recommended BP targets are the same for women and men, and all classes of antihypertensive medications may be used equally in the treatment of hypertension in both sexes.^453^

### 10.2. Hypertension in Pregnancy

Hypertension in pregnancy is one of the leading causes of maternal and perinatal morbidity and mortality worldwide, with a higher burden observed in low-income countries in Latin America and the Caribbean, including Brazil.^456-458^ Hypertension in pregnancy is defined as SBP ≥ 140 mm Hg and/or DBP ≥ 90 mm Hg, based on the average of at least two measurements taken 4 hours apart or during two separate outpatient visits.^456^ It is important to emphasize that BP measurement during pregnancy should be performed with the woman in a seated position, and the fifth Korotkoff sound should be used to determine DBP when using the auscultatory method.

#### 10.2.1. Classification

The definitions and classification of hypertensive disorders of pregnancy are described in Chart 10.1. Compared to the 2020 Brazilian Hypertension Guidelines, the concepts of WCH, MH, and posterior reversible encephalopathy syndrome (PRES) syndrome have been added.^456,459,460^

**Chart 10.1:** Definitions and classification of hypertensive disorders of pregnancy

DEFINITIONS	
White-coat hypertension	SBP ≥ 140 and/or DBP ≥ 90 mmHg when measured in the office or clinic, and BP < 135/85 mmHg when measured by daytime ABPM or HBPM
Masked hypertension	SBP < 140 and/or DBP < 90 mmHg when measured in the office or clinic, but BP ≥ 135/85 mmHg when measured by daytime ABPM or HBPM
Hypertension in pregnancy	SBP ≥ 140 mm Hg and/or DBP ≥ 90 mm Hg, measured on two different occasions at least 4 hours apart.
Severe hypertension in pregnancy	SBP ≥ 160 mm Hg and/or DBP ≥110 mm Hg, measured on two different occasions at least 4 hours apart.
Proteinuria	≥ 300 mg urinary protein in a 24-hour urine collection, or a urine protein to creatinine ratio of ≥ 0.3 g/g in a spot urine sample, or a ++ result on urine dipstick testing (ideally confirmed with quantitative methods).
**CLASSIFICATION**	
Preeclampsia (with or without severe features)	SBP ≥ 140 mm Hg and/or DBP ≥ 90 mm Hg, typically after 20 weeks of gestation, often accompanied by proteinuria. In the absence of proteinuria, preeclampsia can be diagnosed in the presence of any of the following severe features: thrombocytopenia (< 100,000 × 10^9^/L), serum creatinine > 1.1 mg/dL or double the baseline, liver transaminases elevated to twice the normal value, pulmonary edema, and visual disturbances or persistent headache not accounted for by other diagnoses.
Chronic hypertension	Hypertension diagnosed or developed before pregnancy or before 20 weeks of gestation; or hypertension first diagnosed during pregnancy that does not resolve postpartum.
Chronic hypertension with superimposed preeclampsia	Development of preeclampsia in a woman with pre-existing hypertension prior to pregnancy or before 20 weeks of gestation.
Gestational hypertension	SBP ≥ 140 mm Hg and/or DBP ≥ 90 mm Hg in a previously normotensive woman, occurring after 20 weeks of gestation, measured on two different occasions at least 4 hours apart, without proteinuria or severe features, and returning to normal postpartum.
**OTHER DIAGNOSTIC DEFINITIONS**	
Eclampsia	Tonic-clonic seizures in the absence of other causative conditions in a patient with preeclampsia.
HELLP syndrome	Hemolysis, elevated liver enzymes, and low platelet count, with or without hypertension.
PRES syndrome	Neurological symptoms such as headache, seizures, confusion, vomiting, and visual disturbances. MRI demonstrates vasogenic edema, predominantly in the parieto-occipital regions.

BP: blood pressure; DBP: diastolic blood pressure; HELLP: hemolysis, elevated liver enzymes and low platelets; PRES: posterior reversible encephalopathy syndrome; SBP: systolic blood pressure.

#### 10.2.2. Prediction and Prophylaxis of Preeclampsia

Preeclampsia (PE) prediction should ideally be performed in the first trimester, to allow timely initiation of prophylactic interventions. The Fetal Medicine Foundation (FMF) first trimester prediction model is available at: https://fetalmedicine.org/research/assess/preeclampsia/first-trimester. This model combines maternal risk factors (Chart 10.2) with MAP, uterine artery pulsatility index (uterine artery Doppler between 11 and 14 weeks of gestation), and serum placental growth factor (PIGF). Validated for use in the first trimester, this model achieves detection rates of 90% for early PE (< 34 weeks) and 75% for preterm PE (< 37 weeks). It outperforms the use of maternal risk factors alone in identifying high-risk pregnancies.^461^

**Chart 10.2:** Risk factors for preeclampsia

	High-risk factor (presence of 1 criterion)	MODERATE-risk factors (presence of 2 or more criteria)
Previous pregnancy history	Previous PE	Prior placental abruption Previous intrauterine growth restriction Previous stillbirth
Demographic data	BMI > 30 kg/m^2^ before gestation	Maternal age > 40 years
Clinical History	Chronic hypertension Pregestational diabetes mellitus Chronic kidney disease Systemic lupus erythematosus/antiphospholipid syndrome	
Current gestation	Assisted reproduction	Nulliparity Multifetal pregnancy

Source: Adapted from Magee et al.^456^

For PE prevention, all women should be encouraged to engage in physical activity, preferably starting before conception.^462^ The use of low-dose aspirin (100-150 mg at bedtime) from 16 weeks until 36 weeks’ gestation reduces the risk of PE – particularly early PE – in high-risk patients.^463,464^

Calcium supplementation in doses above 500 mg, especially in women with low dietary intake (< 900 mg/day), initiated before pregnancy or by 20 weeks of gestation, may also reduce the risk of PE.^456,465,466^

#### 10.2.3. Therapeutic approach to hypertension in pregnancy

There is no difference in outcomes between strict bed rest and some rest in hospital for pregnant women with hypertension and proteinuria,^467^ whereas some rest in hospital reduces the risk of severe hypertension compared with routine activity at home.^468^ However, bed rest should not be recommended routinely for the management of hypertension during pregnancy.^441^ Clinical outcomes for mothers and newborns are similar between antenatal day care units and hospital admission, but women prefer outpatient day care.^469^

There are no specific recommendations for inpatient care, but continuous monitoring of both maternal and fetal conditions is essential. BP should be measured at least four times daily, with daily assessment of weight and urine output, along with patient guidance on warning signs. Laboratory tests (complete blood count with platelet count, liver enzymes, uric acid, creatinine, and proteinuria) should be performed once or twice a week. Fetal development can be monitored through assessments of fetal growth and movements, evaluation of fetal well-being and biophysical profile, and US examinations.^469^

The initiation of antihypertensive therapy in women with nonsevere hypertension (ie, below 160/110 mm Hg) remains controversial, except in those with HMOD. A Cochrane systematic review found that treating mild to moderate hypertension with more intensive BP targets did not significantly reduce maternal, fetal, or neonatal morbidity.^470^ In cases of persistent severe hypertension (≥ 15 minutes), antihypertensive medication is indicated in addition to NPMs to prevent irreversible neurological damage. To prevent maternal death, SBP > 150-160 mm Hg should be considered an indication for urgent treatment. In line with other Brazilian and international guidelines, a threshold of 160 mm Hg is recommended.^45,456,459^

Aggressive treatment (targeting DBP up to 85 mm Hg) vs less aggressive treatment (DBP up to 100 mm Hg) results in a reduction in severe hypertension and adverse fetal outcomes. BP control target should be SBP > 120 and < 160 mm Hg and DBP > 80 and < 110 mm Hg, as both hypertension and treatment-induced hypotension can impair placental perfusion and consequently lead to fetal growth restriction.^470^ The goal of treatment is to prevent the progression of HMOD, cardiac and cerebrovascular complications, as well as obstetric and fetal complications.^471^

Recommendations for pharmacological therapy in women with hypertension during pregnancy are based on observational studies, given the challenges of conducting RCTs in this population; therefore, the certainty of evidence is considered low. Pharmacological therapy should be initiated as monotherapy with first-line agents: methyldopa, long-acting nifedipine or amlodipine, or beta-blockers (except atenolo).^472^ If BP control is not achieved, another first-line agent or a second-line medication (thiazide DIUs, clonidine, or hydralazine) may be added. ACE inhibitors and ARBs are contraindicated during pregnancy due to the risk of fetal malformations, which may result in intrauterine renal failure,^471,473^ and atenolol is contraindicated because of the high risk of fetal growth restriction.^471,473^ The use of MRAs is also contraindicated in pregnancy due to hormonal blockade and the lack of safety data; these agents are classified as FDA pregnancy category C. DIUs should also be avoided in patients with PE, as they may exacerbate intravascular volume depletion.^471^ A study comparing the efficacy of labetalol, nifedipine retard, and methyldopa for the management of severe hypertension in pregnancy suggested that all three medications are viable options; however, nifedipine retard was more effective than labetalol and methyldopa.^474^

For severe hypertension in pregnancy, two recent systematic reviews and meta-analyses evaluated the best therapeutic strategies for these conditions. One, including 41 studies in the systematic review and 46 in the meta-analysis, found similar efficacy for nifedipine, hydralazine, and labetalol.^475,476^ The other, including 19 RCTs, found both labetalol and hydralazine to be effective for hypertensive disorders of pregnancy, but labetalol also reduced the incidence of maternal hypotension.^476^ International guidelines recommend intravenous labetalol for the treatment of hypertensive emergencies in eclampsia or severe PE; however, this medication is not available in Brazil.^117,477^ Thus, in Brazil, hypertensive emergencies in pregnant women can be managed with intravenous hydralazine at a dose of 5 mg every 20-30 minutes, up to a maximum total dose of 15 mg. If hydralazine is unavailable, oral nifedipine 10 mg may be administered, with repeat doses of 10-20 mg every 20-30 minutes as needed.^477^ Sublingual nifedipine should not be used in any situation of acute BP elevation.^1^

In exceptional cases, such as acute pulmonary edema (APE) and severe and refractory hypertension, intravenous nitroglycerin^448^ or sodium nitroprusside^478^ are preferred for urgent BP control, with nitroprusside limited to a maximum of 4 hours due to the risk of fetal cyanide toxicity.

The use of magnesium sulfate is recommended for the prevention of eclampsia in pregnant women with symptomatic severe hypertension and for the treatment of eclampsia.^456,479^

#### 10.2.4. Postpartum Hypertension

In women without chronic hypertension, BP levels usually return to normal within the first week postpartum (typically by days 5-6). However, during this period, there remains a risk of hypertension-related complications such as eclampsia, stroke, APE, and AKI.^480^

A Brazilian RCT demonstrated that methyldopa and captopril were equally effective in controlling BP in the immediate postpartum period.^481^

Postpartum women may receive any antihypertensive medication, including ACE inhibitors, with the choice guided by breastfeeding considerations. Preference should be given to medications that are minimally excreted in breast milk.

Chart 10.3 shows the main antihypertensive medications available in Brazil and their excretion in breast milk.^1^

**Chart 10.3:** Antihypertensive medications and breastfeeding

Medication	Excretion in breast milk	Breastfeeding recommendation
Nifedipine	Minimal	Allowed
Amlodipine	Insufficient data	Uncertain (apparently safe)
Diltiazem, Verapamil	Insufficient data	Uncertain (use alternative)
Clonidine	Increased	Avoid
Enalapril, captopril	Minimal	Allowed without restriction
Lisinopril, ramipril	Insufficient data	Uncertain (apparently safe)
Losartan, valsartan, candesartan, olmesartan, telmisartan	Insufficient data	Uncertain (use alternative)
Hydrochlorothiazide	Minimal	Use low dose (< 50 mg/day)
Chlorthalidone	Minimal	Slow elimination in newborns – avoid
Furosemide	Insufficient data	May decrease milk production
Spironolactone	Minimal	Allowed
Atenolol	Increased	Avoid
Metoprolol	Minimal	Allowed
Carvedilol	Insufficient data	Unclear
Propranolol	Minimal	Allowed
Bisoprolol	Insufficient data	Uncertain (apparently safe)
Hydralazine	Minimal	Allowed
Methyldopa	Minimal	Allowed

#### 10.2.5. Future Cardiovascular Risk

Hypertensive disorders of pregnancy are markers of increased future CV risk. Women with such a history should receive more comprehensive and proactive follow-up to ensure effective prevention of CVD and renal disease.^1,45^

**Table:** 

Key messages on the management of hypertension in pregnant and non-pregnant women
From the age of 60, women tend to have higher BP levels and a greater prevalence of hypertension.
Differences in hypertension development in women are linked to gender-specific regulatory mechanisms.
The investigation of secondary causes of hypertension in young women is essential; the main conditions include obesity, polycystic ovary syndrome, obstructive sleep apnea, aortic coarctation, autoimmune diseases, endocrine disorders, and primary nephropathies.
Women have specific hypertension-related risk factors, including those associated with social determinants and biological factors. Some of these include menopause, polycystic ovary syndrome, use of oral contraceptives, and hypertensive disorders of pregnancy.
The association between hypertension and the use of oral contraceptive pills shows a 13% increased risk of new cases, with a linear dose-response relationship for every 5-year increment in oral contraceptive use.
Adverse effects of antihypertensive medications are reported more frequently in women, particularly after menopause, and may contribute to lower treatment adherence.
Evidence indicates that female sex is a predictor of less intensive antihypertensive therapy compared with male sex.
Hypertension in pregnancy should be classified as preeclampsia, chronic hypertension, chronic hypertension with superimposed preeclampsia, or gestational hypertension. Additional diagnostic considerations include eclampsia, PRES syndrome, HELLP syndrome, white coat hypertension, and MH.

MH: masked hypertension; HELLP: hemolysis, elevated liver enzymes, and low platelets.

**Table:** 

Recommendations for pregnant and non-pregnant women	Strength of recommendation	Certainty of evidence
BP should be monitored before OCP prescription in young women. After initiation, regular monitoring (every 6 months) is recommended, including home BP monitoring and management of other CV risk factors.	STRONG	MODERATE
Alternative contraceptive methods or OCP discontinuation should be considered in women with persistent SBP ≥ 160 mm Hg and/or DBP ≥ 100 mm Hg while on OCPs.	STRONG	LOW
In menopausal and postmenopausal women, it is recommended to initiate pharmacological treatment with a combination of a RAS inhibitor and a CCB or a thiazide/thiazide-like diuretic.	STRONG	MODERATE
BP targets should be the same for women and men, and the same classes of antihypertensive medications may be used for both sexes.	STRONG	MODERATE
The risk of preeclampsia should be assessed between 11 and 14 weeks of gestation using at least maternal risk factors, preferably combined with uterine artery Doppler pulsatility index and measurement of placental growth factor.	STRONG	LOW
All pregnant women, unless contraindicated, should be encouraged to engage in physical activity to reduce the likelihood of gestational hypertension and preeclampsia.	STRONG	MODERATE
Calcium should be supplemented in high-risk pregnant women with low dietary intake (< 900 mg/day) to reduce the risk of preeclampsia.	STRONG	MODERATE
The use of low-dose aspirin, initiated before 16 weeks of gestation in high-risk pregnant women, is recommended to reduce the incidence of preeclampsia before 34 weeks.	STRONG	MODERATE
Non-pharmacological treatment alone is not recommended in cases with persistent SBP >160 mm Hg for more than 15 minutes.	STRONG	LOW
Some rest in hospital, with monitoring, is recommended for pregnant women with preeclampsia; hospitalization should be indicated for those with severe hypertension.	STRONG	MODERATE
Urgent antihypertensive treatment is recommended in pregnant women in cases of severe hypertension and when clinical signs of emergency are present.	STRONG	MODERATE
Antihypertensive treatment should be initiated in pregnant women when BP is above 150–160/100–110 mm Hg, with a BP target of 120–160/80–100 mm Hg.	WEAK	LOW
In pregnant women with hypertension, it is recommended to initiate treatment with methyldopa or dihydropyridine calcium channel blockers (long-acting nifedipine or amlodipine).	STRONG	LOW
ACE inhibitors or ARBs are not recommended during pregnancy due to the risk of fetal malformations.	STRONG	MODERATE
Atenolol, prazosin, and MRAs are not recommended during pregnancy due to potential associated risks.	STRONG	LOW
The use of magnesium sulfate is recommended for the prevention of eclampsia in pregnant women with symptomatic severe hypertension and for the treatment of eclampsia.	STRONG	MODERATE
Long-term follow-up is recommended for all women with a history of hypertension in pregnancy to ensure effective prevention of CV disease.	STRONG	LOW

ACE: angiotensin-converting enzyme; ARBs: angiotensin receptor blockers; BP: blood pressure; CCB: calcium channel blocker; CV: cardiovascular; DBP: diastolic blood pressure; MRAs: mineralocorticoid receptor antagonists; OCP: oral contraceptive pill; RAS: renin-angiotensin system; SBP: systolic blood pressure.

## 11. Hypertensive Crisis

### 11.1. Definition and Classification

Hypertensive crisis is characterized by an acute and severe elevation of BP, accompanied or not by acute HMOD.^1,44,45,482,483^ HEs are symptomatic clinical situations in which there is a marked increase in BP (arbitrarily defined as SBP ≥ 180 and/or DBP ≥ 110 mm Hg or, depending on the situation, even with lower values) along with acute and progressive HMOD.^1,44,45,482,483-5^ The term "hypertensive urgency" is currently under discussion. It is now recommended that the appropriate term for situations previously referred to as hypertensive urgency be "severe elevation of BP without progressive damage to target organs."^1,44,45,482,483^ Chart 11.1 shows the main differences between HE and severe BP elevation without acute HMOD (formerly hypertensive urgency).

**Chart 11.1:** Diagnosis, prognosis, and management of hypertensive crises

Severe BP elevation without acute HMOD	Emergency
Markedly high BP values	Markedly high BP values
Without acute and progressive HMOD	With acute and progressive HMOD
Oral combination therapy	Parenteral medications
Without imminent risk of death	With imminent risk of death
Early outpatient follow-up (1 to 7 days)	ICU admission

BP: blood pressure; HMOD: hypertension-mediated organ damage; ICU: intensive care unit.

A common condition encountered in emergency departments (EDs), which is part of the differential diagnosis of hypertensive crisis, is hypertensive pseudocrisis. It generally occurs in patients with uncontrolled hypertension, presenting with very high BP values but who are oligosymptomatic or asymptomatic. Elevated BP triggered by emotional, painful, or uncomfortable events–such as migraine, rotational vertigo, musculoskeletal headache, and panic disorder–is also classified as a hypertensive pseudocrisis.^1,482^

### 11.2. Classification

HE is defined not only by elevated BP, but primarily by the patient's clinical status. It can manifest as a CV, cerebrovascular, or renal event, or as PE or eclampsia during pregnancy. Chart 11.2 shows the classification of HEs.

**Chart 11.2:** Classification of hypertensive emergencies

HYPERTENSIVE EMERGENCIES
**Cerebrovascular** - Hypertensive encephalopathy - Intracerebral hemorrhage - Subarachnoid hemorrhage - Ischemic stroke **Cardiocirculatory** - Acute aortic dissection - Acute pulmonary edema with left ventricular failure - Acute myocardial infarction - Unstable angina **Systemic** - Accelerated/malignant hypertension - Hypertension-MOD **Severe adrenergic crisis** - Pheochromocytoma crisis - Illicit drug overdose (cocaine, crack cocaine, LSD) **Hypertension in pregnancy** - Eclampsia - Severe preeclampsia - HELLP syndrome - Severe hypertension in late pregnancy

HELLP: hemolysis, elevated liver enzymes, and low platelets; MOD: multi-organ damage.

### 11.3. Epidemiological and prognostic aspects

#### 11.3.1. Epidemiology

Hypertensive crises account for 0.45% to 0.59% of all ED visits, with HEs making up 25% of all hypertensive crisis cases. Ischemic stroke and APE are the most common conditions associated with HEs.^1,482^

#### 11.3.2. Prognosis

The mortality rate associated with untreated HE can reach approximately 80% within 1 year, depending on the type of HE, and antihypertensive treatment substantially improves the prognosis.^1,482^ Individuals with hypertensive urgency tend to have a higher 5-year survival rate than those with hypertensive emergency.^148,2^

### 11.4. Complementary Clinical and Laboratory Investigation

A thorough medical history and physical examination are essential for the correct diagnosis of a hypertensive crisis. If a life-threatening condition is identified, appropriate treatment should be initiated concurrently with the investigation.^1,44,45,482,483^ The medical history should include information about the patient's usual BP and any situations that might have triggered the hypertensive crisis, such as anxiety, stress, pain, or excessive salt intake. The use of antihypertensive medications (dose and adherence), their discontinuation (adrenergic inhibitors), or the use of substances that can increase BP should be considered, including illicit drugs.^1,44,45,482,483^ It is important to inquire about the most common symptoms, such as dyspnea, palpitations, headache, chest pain, and neurological symptoms.^1,44,45,482-484^ BP should be measured in both upper limbs, preferably in a quiet environment. Systematic clinical, laboratory, and imaging investigations can assist in the diagnosis of acute HMOD (Chart 11.3).^1,44,45,482-485^

**Chart 11.3:** Complementary clinical and laboratory investigation according to hypertension-mediated organ damage in hypertensive emergencies^1,44,45,482,483^

Target organ	Hypertensive emergency	Symptoms	Physical examination abnormalities	Complementary assessment
Heart and arteries	HF, APE Coronary syndrome Acute aortic dissection	Chest, abdominal, upper back, or low back pain Dyspnea, orthopnea Palpitations Edema	HR, gallop rhythm Jugular venous distention, pulmonary, abdominal, and peripheral congestion Heart and vascular murmurs Asymmetric or absent peripheral pulses Difference in BP between limbs	ECG, chest radiograph Echocardiogram O2 saturation Markers of myocardial necrosis BNP and NT-proBNP Chest CTA and MRI
Brain	Stroke Hypertensive encephalopathy	Vertigo, headache Visual, hearing, or speech impairments Paresis or paresthesia Confusion, agitation, drowsiness	Altered level of consciousness Seizure, focal deficits, neck stiffness	Brain CT or MRI Lumbar puncture for CSF collection
Kidney	AKI Thrombotic microangiopathy	Altered urine output and frequency Urine color change Edema Anorexia, weight loss, nausea, vomiting, fatigue	Edema Hematuria or foamy urine Abdominal masses and murmurs Pale skin and mucosa	Urine sediment examination with dysmorphic hematuria and/or proteinuria, ACR, complete blood count, creatinine, urea, sodium, potassium, chloride, venous blood gas analysis, lactate dehydrogenase Renal and urinary tract US Renal artery Doppler US Abdominal CT or MRI
Retina	Retinopathy KWB grade 3 or 4	Blurred vision Phosphenes, scotomas Amaurosis	Spasms, pathological arteriovenous shunts Papilledema, retinal hemorrhages, cotton-wool spots	
Suspected HE	ECG, chest radiograph, complete blood count, troponin, creatinine, type 1 urine, and potassium; Exclude pregnancy in women of childbearing age.			

ACR: albumin-to-creatinine ratio; AKI: acute kidney injury; APE: acute pulmonary edema; BNP and NT-proBNP: B-type natriuretic peptide; BP: blood pressure; CSF: cerebrospinal fluid; CT: computed tomography; CTA: computed tomography angiography; ECG: electrocardiogram; HE: hypertensive emergency; HELLP: hemolysis, elevated liver enzymes, and low platelets; HF: heart failure; HR: heart rate; KWB: Keith- Wagener-Barker classification; MRI: magnetic resonance imaging; O2 : oxygen; PE: preeclampsia; US: ultrasound.

### 11.5. Overall management of hypertensive crisis

The presence and type of acute HMOD should guide the treatment of hypertensive crisis (Figure 11.1 and Chart 11.4). It is important to consider the risks associated with rapid or excessive BP reduction, with can lead to irreversible target organ ischemia due to loss of BP autoregulation, and to evaluate the benefits of treatment to minimize the progression of identified HMOD.^486^ There is no evidence supporting ED treatment for patients with severe BP elevation without acute HMOD; therefore, outpatient management is recommended, similar to the management of patients with uncontrolled and asymptomatic hypertension.^1,482,486^ The first measure for patients with severe BP elevation and symptoms unrelated to acute HMOD is clinical observation for 30 minutes in a quiet environment, assessing for symptom resolution and BP reduction.^1,482^ Acute/abrupt BP reductions > 25% to 30% within the first 2 to 4 hours should be avoided due to the risk of tissue ischemia–this can occur with the use of immediate-release nifedipine, which is not recommended.^1,482,487^

**Figure 11.1:** Flowchart for hypertensive crisis management.^1,44^

**Chart 11.4:** Intravenous medications used for the treatment of hypertensive emergencies

Medication	Method of administration and dosage	Onset of action	Duration	Indications	Adverse events and precautions
**Sodium nitroprusside** (arterial/venous vasodilator stimulates cGMP formation)	Continuous IV infusion 0.25-10 mg/kg/min	Immediate	1-2 min	Most hypertensive emergencies	Cyanide poisoning, severe hypotension, nausea, vomiting. Caution in AKI and liver disease. Protect from light.
**Nitroglycerin** (arterial/venous vasodilator)	Continuous IV infusion 5-15 mg/h	2-5 min	3-5 min	Coronary insufficiency, APE	Headache, reflex tachycardia, tachyphylaxis, flushing, methemoglobinemia
**Metoprolol** (selective BB)	5 mg IV (repeat every 10 min, if necessary up to 20 mg)	5-10 min	3-4 h	Coronary insufficiency, acute aortic dissection	Bradycardia, advanced AV block, HF, bronchospasm
**Esmolol** (ultra-short-acting selective β-adrenergic blocker)	Loading dose: 500 μg/kg Intermittent IV infusion 25-50 μg/kg/min **↑** 25 μg/kg/min 10-20 min. Maximum 300 μg/kg/min	1-2 min	1-20 min	Acute aortic dissection (in combination with SNP), severe postoperative hypertension	Nausea, vomiting, first-degree AV block, bronchospasm, hypotension
*Phentolamine (α-adrenergic blocker)	Continuous IV infusion: 1-5 mg Maximum 15 mg	1-2 min	3-5 min	Catecholamine excess	Reflex tachycardia, flushing, dizziness, nausea, vomiting
*Trimethaphan (ganglionic blocker of the SNS and PSNS)	Continuous IV infusion: 0.5-1.0 mg/min. **↑** 0.5 mg/min up to a maximum of 15 mg/min	1-5 min	10 min	Catecholamine excess, acute aortic dissection	Tachyphylaxis
**Hydralazine** (direct-acting vasodilator)	10-20 mg IV or 10-40 mg IM every 6 h	10-30 min	3-12 h	Eclampsia	Tachycardia, headache, vomiting. Worsening angina and AMI. Monitor intracranial pressure
*Diazoxide (vasodilator)	IV infusion 10-15 min 1-3 mg/kg. Maximum 150 mg	1-10 min	3-18 h	Hypertensive encephalopathy	Sodium and water retention, hyperglycemia, and hyperuricemia
*Fenoldopam (dopamine agonist)	Continuous IV infusion 0.1-1.6 μg/kg/min	5-10 min	10-15 min	AKI	Headache, nausea, flushing
*Nicardipine (CCB)	Continuous IV infusion 5-15 mg/h	5-10 min	1-4 h	Stroke, hypertensive encephalopathy, APE	Reflex tachycardia, avoid in patients with HF or myocardial ischemia
*Labetalol (α/β-adrenergic blocker)	Loading dose: 20-80 mg every 10 min Continuous IV infusion 2 mg/min (maximum 300 mg/24 h)	5-10 min	2-6 h	Stroke, acute aortic dissection (in combination with SNP)	Nausea, vomiting, AV block, bronchospasm, orthostatic hypotension
*Enalaprilat (ACE inhibitor)	Intermittent IV infusion 1.25-5.0 mg every 6 h	15 min	4-6 h	Left ventricular failure with APE	Hypotension, AKI
**Furosemide** (loop diuretic)	20-60 mg (repeat after 30 min)	2-5 min	30-90 min	APE, hypervolemia	Hypokalemia

ACE: angiotensin-converting enzyme; AKI: acute kidney injury; AMI: acute myocardial infarction; APE: acute pulmonary edema; AV: atrioventricular; BB: beta-blocker; CCB: calcium channel blocker; cGMP: cyclic guanosine monophosphate; HF: heart failure; IM: intramuscular; IV: intravenous; PSNS: parasympathetic nervous system; SNP: sodium nitroprusside; SNS: sympathetic nervous system.

* Not available in Brazil.

A retrospective cohort study showed that recurrence of ED visits and mortality rates were similar for patients with markedly elevated BP but without acute HMOD, regardless of whether they received acute antihypertensive therapy or not. If there is no BP reduction or symptom resolution after clinical observation, consider using oral short-acting antihypertensives with a rapid onset of action (typically clonidine or captopril), adjusting long-term antihypertensives, or initiating antihypertensive therapy (see Chapter 7 on pharmacological treatment).^1,482,488^

### 11.6. Hypertensive emergencies in special situations

#### 11.6.1. Hypertensive Encephalopathy^1,482,485^

Hypertensive encephalopathy is a neurological HMOD characterized by cerebral edema secondary to acute BP elevation. Generally reversible, it can affect individuals with chronic hypertension who develop malignant hypertension (detailed later in this chapter) or normotensive individuals who experience acute, excessive BP elevations. Failure in cerebral perfusion autoregulation results in increased intracranial pressure. The onset is insidious and may progress to holocranial headache, nausea and/or vomiting, visual disturbances, confusion, coma, generalized seizures, hyperreflexia, and signs of intracranial hypertension. In the presence of a localized deficit, a focal neurological lesion should be considered, and brain magnetic resonance imaging (MRI) or computed tomography (CT) should be performed.^489^

The goal of treatment is to restore cerebral autoregulation by gradually reducing BP (initially reducing BP by 20% to 25% within 1 hour), as rapid reduction can cause cerebral hypoperfusion due to loss of BP autoregulation. Intravenous medications can be used, such as sodium nitroprusside (SNP), nitroglycerin (NTG), or esmolol (available in Brazil), although caution is advised with NTG or SNP due to reduced cerebral blood flow observed with their use in normotensive individuals.^490^ A systematic review comparing intravenous treatments of neurological HEs, including hypertensive encephalopathy, was unable to determine the best medication for these situations because of the very low quality of evidence.^491^ Oral antihypertensives should also be initiated for better BP control (BP target of 160-100 mm Hg within 48 hours).

#### 11.6.2. Stroke

Hypertension is the main risk factor for stroke, particularly hemorrhagic stroke. Diagnosis is based on a comprehensive neurological examination, and the National Institutes of Health Stroke Scale (NIHSS) should be used to assess the severity of the condition. Brain CT and MRI scans allow us to determine the type of stroke (85% ischemic and 15% hemorrhagic) and identify the affected brain region.^1,482,483,485^ MRI is more sensitive than CT for incipient infarction.^492^ Prehospital treatment with rapid and substantial BP reduction has not shown benefits in ischemic or hemorrhagic stroke, except for a reduction in motor deficit in patients with hemorrhagic stroke.^493^

##### 11.6.2.1. Ischemic Stroke

Elevated BP is a common occurrence in ischemic stroke, affecting approximately 80% of patients, especially those with a history of hypertension. BP often decreases spontaneously within 90 to 120 minutes during the acute phase of stroke.^494-497^

The main recommendations for the management of hypertensive emergency with ischemic stroke are summarized in the table at the end of the chapter.

##### 11.6.2.2. Hemorrhagic Stroke

Hemorrhagic stroke accounts for 15% of all stroke cases, with a recent Swiss study reporting an annual incidence of 13.2 per 100,000 individuals.^498^ Elevated BP increases the risk of hematoma expansion, raises the risk of death, and worsens the prognosis. Cerebral edema occurs in 30% of cases, typically within the first 24 hours, often requiring decompressive craniectomy and transfer to specialized centers.^1,482,492,499^

The main recommendations for the management of hypertensive crisis with hemorrhagic stroke are summarized in the table at the end of the chapter.

#### 11.6.3. Acute Coronary Syndromes

Acute coronary syndromes (ACSs) may be accompanied by elevated BP as a reflection of myocardial ischemia. Increased PVR raises myocardial oxygen demand by increasing left ventricular parietal tension.^500-503^

For older and frail individuals, the target BP may be less restrictive. Hydralazine, nifedipine, and SNP are not indicated.^1,482,485^ Intravenous nitrates reduce PVR, improve coronary perfusion, and have a systemic venodilator effect, reducing preload and myocardial oxygen consumption.

#### 11.6.4. Acute Pulmonary Edema

Approximately 40% of patients admitted with APE and HE have preserved left ventricular function.^504^ Myocardial ischemia may also be involved in the pathophysiology of APE associated with HE.^1,482,485^ APE should be treated in the ICU with intravenous medication, continuous monitoring, and gradual BP reduction. NTG and SNP reduce both preload and afterload. Loop DIUs also reduce volume overload and, consequently, BP. In some cases, noninvasive ventilation, such as continuous positive airway pressure (CPAP) and bilevel positive airway pressure (BiPAP), may be indicated to reduce pulmonary edema and venous return.^1,482,485^

#### 11.6.5. Acute Aortic Dissection

Pain control should be concomitantly provided with morphine. The antihypertensives of choice are short-acting, titratable intravenous BBs (metoprolol or esmolol), which reduce HR and left ventricular ejection velocity. In patients with asthma, non-dihydropyridine CCBs may be used. SNP should be initiated only after β-blockade to prevent an increase in HR and aortic ejection velocity.^1,485,485,505^

#### 11.6.6. Adrenergic Crisis

Adrenergic crisis can be caused by the sudden discontinuation of sympatholytic agents and BBs, pheochromocytomas, or illicit substance use. In these situations, SBP should be reduced to < 140 mm Hg within the first hour.^1,482,483^

In cases of abrupt discontinuation of medications that act on the sympathetic nervous system, their immediate reintroduction is recommended to prevent serious complications.^1,482,483^ Recurrent paroxysmal hypertensive crises with the triad of palpitations, sweating, and headache, without a report of illicit drug use, should prompt investigation for pheochromocytomas.^1,482,506^

Treatment with doxazosin is recommended,^507^ as phentolamine is not available in Brazil. β-blockade can only be initiated after α-blockade because of the paradoxical elevation of BP with blockade of β-adrenergic vasodilation.^507^

Amphetamines, ecstasy, and cocaine can raise BP through sympathomimetic action.^508,509^ Amphetamines cause increased BP, tachycardia, palpitations, sweating, and arrhythmias.^508^ Ecstasy intoxication can lead to serotonin syndrome (rhabdomyolysis and AKI).^508^ The acute increase in BP induced by cocaine is more pronounced in hypertensive patients than in normotensive individuals and is potentiated by caffeine, nicotine, and particularly alcohol.^1,482,509^ In this condition, vasoconstriction, thrombus formation, increased myocardial oxygen demand, conduction defects, and arrhythmias can occur.^1,482^

#### 11.6.7. Accelerated-Malignant Hypertension

Malignant hypertension is an HE with an excessive BP elevation and accelerated disease progression, characterized by acute microvascular injury, systemic endothelial damage, and impaired autoregulatory mechanisms, affecting the retina, brain, heart, kidneys, and vascular system. Typical findings include Keith-Wagener-Barker grade 4 retinopathy, with or without AKI, and fibrinoid necrosis of renal arterioles, which reflect systemic endothelial damage. Even in the absence of these findings, the condition can be considered if multiple severe target organ injuries are present concomitantly.^510,511^ This condition can have a rapidly progressive and fatal course, with an 80% mortality rate within 2 years if left untreated.^511^ It usually, but not necessarily, presents with stage 3 hypertension. Elevated BP in the presence of retinal hemorrhages and spots in the fundus, but without papilledema, is referred to as accelerated hypertension. The terms "malignant" and "accelerated" are considered interchangeable, leading to the used of the combined term "accelerated-malignant hypertension."^36^ If papilledema is observed, neuroimaging will be required to rule out other neurological causes.^1,482,485^

Although treatment for accelerated-malignant hypertension cannot completely reverse the high renal and cardiac risks, it drastically reduces mortality.^1,482^ Patients require hospitalization and intensive care to control BP with vasodilators with an immediate onset of action (SNP).^1,482,483,485,513^ Subsequently, oral antihypertensives should be initiated, including ACEIs or ARBs, along with any other classes of medications that are deemed necessary.^1,482,483,485,513^

#### 11.6.8. Hypertension with Multi-Organ Damage

Hypertension with multi-organ damage (hypertension-MOD) is characterized by the involvement of at least 3 of the following 4 systems:^514^ renal – rapid deterioration of kidney function or proteinuria; cardiac – marked LVH, systolic dysfunction, ventricular repolarization abnormalities, or increased troponin; neurological – stroke or hypertensive encephalopathy; and hematological – microangiopathic hemolysis.^515^

#### 11.6.9. Hypertension in Pregnancy: Preeclampsia and Eclampsia

The main aspects of diagnosis, BP targets, and treatment are discussed in Chapter 10.

**Table:** 

Key messages on hypertensive crisis management
Important elevated BP (≥ 180/110 mm Hg) in pregnant women, without acute HMOD and without imminent risk of death, allows BP reduction over a longer period with oral antihypertensives, and early outpatient reassessment should occur within 1 to 7 days.
Hypertensive emergency: elevated BP (≥ 180/110 mm Hg) with acute HMOD and immediate risk of death. It requires rapid and gradual reduction of BP within minutes to hours, depending on the specific HMOD present. Intravenous medications are typically used.
The severity of the clinical condition is not determined by the absolute BP level, but rather by the magnitude of BP elevation. While the numerical definition serves as a parameter, it should not be used as an absolute criterion.
The BP reduction target varies depending on the type of HE presentation. BP should be reduced gradually and carefully, by 10% to 15% within the first hour of treatment and by 25% within 2 hours of starting treatment in most clinical situations. Some BP reduction targets are specific to the HMOD involved: Catecholaminergic crises and APE: SBP < 140 mm Hg within 1 hour. Acute aortic dissection: SBP < 120 mm Hg within 1 hour.
Antihypertensive treatment varies depending on the diagnosis of HE. The most commonly used medications include sodium nitroprusside, nitroglycerin, beta-blockers, calcium channel blockers, and loop diuretics.

APE: acute pulmonary edema; BP: blood pressure; HE: hypertensive emergency; HMOD: hypertension-mediated organ damage.

Main recommendations for hypertensive emergencies and marked blood pressure elevation without acute target-organ damage

**Table:** 

Main Recommendations for hypertensive emergencies and for severe blood pressure elevation without acute target-organ damage	Strength of recommendation	Certainty of evidence
In patients with severe BP elevation without acute HMOD (previously known as hypertensive urgency), outpatient reassessment should be performed within 1 to 7 days, with a target SBP < 160 and DBP < 100 mm Hg.	WEAK	LOW
Patients with HE should be admitted to the ICU and receive IV antihypertensives, with BP monitoring and observation of HMOD progression.	STRONG	MODERATE
In patients with HE, BP should be reduced by up to 25% within 1 h; if stable, to 160/100 mm Hg within the next 2 to 6 h; and to normal levels within the next 24 to 48 h, except for specific HMODs (stroke, acute aortic dissection, PE, eclampsia, and PHEO).	WEAK	MODERATE
In patients with ischemic stroke and indication for thrombolysis, BP should be reduced to < 185/110 mm Hg and maintained at < 180/105 mm Hg within the first 24 h after thrombolysis, and do not perform thrombolytic infusion or thrombectomy if BP remains ≥ 185/110 mm Hg.	STRONG	MODERATE
An initial 15% BP reduction is recommended in patients with ischemic stroke and BP ≥ 220/120 mm Hg, associated with comorbidities (aortic dissection, acute coronary events, eclampsia, post-thrombolysis, and/or APE).	WEAK	LOW
In patients with ischemic stroke and BP ≥ 220/120 mm Hg who have not received thrombolytic agents and have no comorbidities, BP should be reduced by 15% within the first 24 h after the onset of ischemic stroke.	WEAK	LOW
It is recommended to safely initiate or restart antihypertensives in neurologically stable individuals with ischemic stroke and BP > 140/90 mm Hg.	STRONG	MODERATE
In patients with hypertension and ischemic stroke, SBP should be 120-130 mm Hg and DBP 70-79 mm Hg within 7 to 10 days after ischemic stroke.	STRONG	HIGH
In patients with hemorrhagic stroke and SBP > 220 mm Hg, reduction and frequent monitoring of BP with continuous IV infusion is recommended, with an SBP target < 180 mm Hg.	STRONG	MODERATE
In patients with mild-to-moderate hemorrhagic stroke and SBP 150-220 mm Hg, BP should be reduced to < 140 mm Hg with IV medications (esmolol and CCBs – nicardipine and clevidipine).	WEAK	MODERATE
In patients with subarachnoid hemorrhage and SBP > 180 mm Hg, SBP should be reduced slowly and gradually over a period of 24 to 72 h.	WEAK	MODERATE
In patients with APE due to HE, the use of IV NTG is recommended within the first 48 h, provided there is no hypotension, right ventricular infarction, or use of phosphodiesterase type 5 inhibitors (sildenafil, tadalafil) in the previous 48 h, and it can be used in combination with BBs or ACE inhibitors that reduce mortality (provided there are no contraindications).	WEAK	MODERATE
In patients with ACS due to HE, the recommended target is SBP < 140 mm Hg (avoid < 120 mm Hg) and DBP 70-80 mm Hg using esmolol, metoprolol, or NTG over a period of 24 to 72 h.	STRONG	HIGH
In patients with adrenergic crisis, the use of benzodiazepines and sublingual nitrate is recommended in milder cases; for other cases, the agents of choice are NTG and SNP.	WEAK	LOW
It is not recommended to use BB alone in adrenergic crisis due to PHEO, as stimulation of the α-adrenergic receptor, with blocked β-adrenergic vasodilation, can worsen coronary spasm, which can be relieved with CCBs.	WEAK	LOW
In patients with acute aortic dissection due to HE, it is recommended to achieve the following targets: HR < 60 bpm and SBP 90-120 mm Hg, which should be achieved within 20 min.	STRONG	LOW

ACE: angiotensin-converting enzyme; ACS: acute coronary syndrome; APE: acute pulmonary edema; BB: beta-blocker; BP: blood pressure; CCB: calcium-channel blocker; DBP: diastolic blood pressure; HE: hypertensive emergency; HMOD: hypertension-mediated organ damage; HR: heart rate; ICU: intensive care unit; IV: intravenous; NTG: nitroglycerin; PE: preeclampsia; PHEO: pheochromocytoma; SBP: systolic blood pressure; SNP: sodium nitroprusside.

## 12. Resistant and Refractory Hypertension

### 12.1. Definition and Classification

Resistant hypertension (RH) is defined as office BP that remains above target values despite the use of three or more classes of antihypertensive agents with synergistic effects, prescribed at the maximum recommended or tolerated doses. These agents should preferably include a thiazide or thiazide-like diuretic, a RAAS inhibitor (either an ACE inhibitor or an ARB), and a long-acting CCB, except under special conditions, for at least 30 to 60 days, after ruling out causes of pseudoresistance. Patients requiring four or more antihypertensive agents to achieve BP control are also considered to have RH (Figure 12.1).^516,517^

**Figure 12.1:** Classification of resistant and refractory hypertension based on the number of medications and blood pressure control.

Refractory resistant hypertension (RHr) is defined as a subgroup of patients with true RH who remain uncontrolled (above their individualized BP targets) despite treatment with five or more antihypertensive agents, preferably including a mineralocorticoid receptor antagonist (MRA)–spironolactone, or eplerenone in cases of hormonal side effects–and a long-acting thiazide-like diuretic (chlorthalidone or indapamide) (Figure 12.1).^517-519^

### 12.2. Epidemiology of Resistant and Refractory Hypertension

The prevalence of RH varies due to several factors: (1) the clinical setting (population-based study, tertiary referral center, etc.), (2) optimization of prescribed antihypertensive drug classes and dosages, (3) prior assessment of treatment adherence, (4) accurate BP measurement, and (5) therapeutic targets defined by different guidelines. In population-based studies, the prevalence of RH is estimated to range from 6% to 18% among individuals with hypertension.^1,517,520^ A large meta-analysis reported a prevalence of 10.3% (95% CI, 7.6-13.2%) for true RH and 10.3% (95% CI, 6.0-15.5%) for pseudoresistant hypertension.^520^ In Brazil, the multicenter ReHOT study found a prevalence of 11.7% for RH.^259^ The prevalence of RfH among patients with RH ranges from 3.6% to 17.6% across different studies, given that its definition has been developed over the past 12 years.^518,521-523^

### 12.3. Pathophysiology

Accumulated evidence in recent years suggests that the development of RH is multifactorial, involving complex interactions among the CNS, kidneys, adrenal glands, heart, and baroreceptors. There is a sophisticated interplay between sympathetic overactivity and the RAAS, renal sodium handling, blood volume, arterial remodeling, endothelial dysfunction, the adrenocortical system, as well as genetic, environmental, psychosocial and factors and ancestry. However, a comprehensive understanding of these pathophysiological mechanisms remains incomplete.^516,517,524,525^

RH is primarily characterized by RAAS hyperactivity, resulting in sodium and fluid retention due to a hyperaldosteronism state. This condition is reflected by elevated aldosterone levels and suppressed renin activity, more pronounced in individuals of African descent, those with obesity, and those with higher salt intake.^516,526-528^ In contrast, RfH is less volume-dependent, as these patients, by definition, are on dual, potent DIU therapy.^525^ Thus, RfH is more strongly associated with sympathetic overactivity, evidenced by elevated HR, 24-hour urinary norepinephrine excretion, and systemic vascular resistance (SVR), as illustrated in Figure 12.2.^516^

**Figure 12.2:** Pathophysiology of resistant and refractory hypertension and its relationship with the proposed pharmacological therapy.

### 12.4. Diagnostic Evaluation

The diagnosis of RH requires the exclusion of conditions associated with pseudoresistance, which is estimated to occur in approximately 50% of patients initially diagnosed with RH (Figure 12.3).^516,517,529,530^

**Figure 12.3:** Causes of pseudoresistance.

#### 12.4.1. Inadequate Blood Pressure Measurement Technique

It is essential to use a cuff size that fits the patient's arm circumference, especially in individuals with obesity, and to exercise caution when measuring BP in older adults with significant arterial stiffness, as this can lead to overestimation of BP values.^516,517,530,531^

#### 12.4.2. White-Coat Effect

The WCE can lead to a false diagnosis of RH. Out-of-office BP monitoring, either using HBPM or ABPM, is mandatory for both the diagnosis and follow-up of patients with RH. ABPM is preferred over HBPM because it provides nighttime BP measurements, which are crucial for diagnosis and stricter BP control, in addition to being a strong prognostic marker.^530-534^

#### 12.4.3. Therapeutic Inertia

Therapeutic inertia refers to delayed initiation of treatment, underdosing, use of inadequate therapeutic regimens, and delayed reassessment of treatment targets, all of which result in failure to adequately adjust the prescription.

#### 12.4.4. Medication Nonadherence

The pharmacological treatment of RH necessarily involves polypharmacy, which reduces adherence, as adherence is inversely proportional to the number of doses prescribed. This is the main cause of pseudoresistance. Therefore, fixed-dose combinations are recommended whenever possible to improve treatment adherence.^516,517,535^

### 12.5. Clinical Approach

RH is associated with increased risk of CV events and mortality.^532,536^ Once true RH is confirmed, with an adequate antihypertensive regimen maintained for at least 30 days, the following diagnostic workup is recommended (Figure 12.4:^1,517,530-532,537^

**Figure 12.4:** Diagnostic approach to resistant hypertension.

- Investigation of risk factors: sex and age, family history of premature CVD, obesity, central obesity, smoking, glycemic and lipid profile abnormalities, social determinants of health, and emotional disorders;
- Assessment of subclinical HMOD: ABI, aortic stiffness (central pressure and PWV), LVH, albuminuria, stage 3 CKD, and carotid disease (carotid US);
- Evaluation for established CVD and CKD: CAD, cerebrovascular disease, HF, PAD; hypertensive retinopathy; stages 4 or 5 CKD;
- Investigation of SH: hyperaldosteronism, renovascular disease, obstructive sleep apnea (OSA). Investigation for OSA is recommended in patients with RH due to its high prevalence in this population, reaching up to 80%.^538,539^

Verification of a healthy lifestyle must be part of the clinical approach, as its implementation reduces the need for pharmacological treatment and lowers CV risk. Special emphasis should be placed on sodium and alcohol intake, as well as on addressing obesity, sedentary behavior, smoking, and illicit drug use.^517,532^

The clinical management of patients with RH is dynamic and requires constant vigilance, with individualized care and frequent assessment–intervention–reassessment cycles involving medication adherence, lifestyle, HMOD, and pharmacological optimization.^519,536^ A key part of screening and identifying these patients is confirming the two defining elements of RH: uncontrolled BP and adequate treatment. These patients should be followed in specialized hypertension centers that offer a multidisciplinary approach with trained professionals and access to the necessary diagnostic tools.^531,540^

### 12.6. Treatment

#### 12.6.1. Non-Pharmacological Treatment

As with all patients with hypertension, NPMs must be implemented. Particular emphasis should be placed on reducing sodium intake,^541^ achieving and maintaining a healthy body weight, smoking cessation, engaging in regular physical activity, and managing stress.^1,532^

#### 12.6.2. Pharmacological Treatment

The use of at least three different classes of antihypertensive agents with complementary pharmacological mechanisms is recommended. Typically, the preferred combination includes an RAAS blocker – either an ACE inhibitor or an ARB – combined with a long-acting dihydropyridine CCB and a thiazide DIU, preferably in fixed-dose combinations.^45^ Replacing a short-acting thiazide diuretic (such as HCTZ) with a long-acting thiazide-like diuretic, such as chlorthalidone or indapamide, may be considered, as RCTs have shown greater antihypertensive efficacy and a lower incidence of adverse effects on lipid profile, serum uric acid, and potassium, as observed in retrospective studies.^1,542^ However, some controversy remains following a head-to-head comparison between HCTZ and chlorthalidone.^543^

After 30 to 60 days of regular use of this therapeutic regimen, the patient should undergo ABPM (preferably) or HBPM.^533,534^ Uncontrolled BP on ABPM or HBPM confirms the diagnosis of true RH, at which point a fourth antihypertensive agent is indicated–preferably spironolactone at 25 to 50 mg/day (considering the hyperaldosteronism status).^517,259,544^ The most common adverse effect of spironolactone is gynecomastia. To minimize this, the dose may be reduced to 12.5 mg/day or the medication may be replaced with eplerenone, which lacks hormonal side effects but has a lower antihypertensive potency.^545^ Other adverse effects include hyperkalemia and deterioration of renal function, especially in patients with CKD who are on RAAS blockers.

Spironolactone should be used with caution in individuals with eGFR < 30 mL/min/1.73 m² and plasma potassium concentrations > 5.5 mEq/L, and may need to be replaced by another antihypertensive agent in cases of treatment-refractory hyperkalemia. Plasma potassium and eGFR should be monitored at intervals of at least 3 to 6 months.^45,517^ When spironolactone or other MRAs are not tolerated or are contraindicated, alternatives include sympatholytic agents^258,544^ or amiloride at doses of 10 to 20 mg/day, which has been shown to be as effective as spironolactone.^1,516,517^ The RCT by Lee et al.^546^ demonstrated that amiloride (up to 10 mg/day) was non-inferior to spironolactone (up to 25 mg/day) in reducing home SBP in patients with RH already on standard triple-drug therapy. BP control rates (< 130 mm Hg) were similar between groups, both in home and office measurements. However, in Brazil, amiloride is only available in 2.5 mg and 5 mg doses, in fixed combinations with hydrochlorothiazide or chlorthalidone.

Based pathophysiology, patients taking four classes of medications – including dual blockade with a thiazide and an MRA – theoretically no longer have hypervolemia or hyperaldosteronism as causal factors for uncontrolled BP. If BP remains above target levels, the next step is to reduce sympathetic overactivity through the use of sympatholytic agents such as central alpha agonists (eg, clonidine), alpha-blockers (eg, doxazosin), or BBs, preferably bisoprolol, carvedilol, or nebivolol. Bisoprolol and nebivolol are highly selective for β1-receptors. Carvedilol and nebivolol have vasodilatory effects and do not adversely affect the metabolic profile^1,259,517,544,545^ (Figure 12.5).

**Figure 12.5:** Treatment flowchart.

In certain individualized cases, modifications to the treatment regimen should be considered:^547^

- Consider replacing the thiazide DIU with a loop DIU in patients with eGFR < 30 mL/min/1.73 m². Chlorthalidone may also used when eGFR is between 15 and 30 mL/min/1.73 m².^548^
- Carefully evaluate specific clinical scenarios such as AF, HF, or CKD, in which the initial triple therapy may require different drug classes. In some cases, there may be an indication to include a BB in the initial triple-drug therapy (see Chapter 7).^1,45,517^
- For patients intolerant to calcium channel blockers (CCBs) due to side effects such as lower-limb edema–a common cause of treatment discontinuation and poor BP control–alternative dihydropyridine CCBs such as manidipine, lercanidipine, or levamlodipine may be prescribed. In carefully selected cases, a non-dihydropyridine CCB such as diltiazem or verapamil may be considered.^1,45,517^
- When CCBs cannot be used, a BB – preferably one with vasodilatory action and no negative metabolic effects, such as nebivolol or carvedilol – may be considered.^1,45,517^
- Low doses of furosemide in combination with chlorthalidone and spironolactone (sequential nephron blockade) in patients with eGFR > 30 mL/min/1.73 m² have shown promising results in randomized trials.^549^

#### 12.6.3. Perspectives

New medications are currently being researched and developed and may play a role in the treatment of RH and RfH. These include endothelin receptor antagonists, aldosterone synthase inhibitors, novel MRAs, and agents that inhibit hepatic production of angiotensinogen (RNA interference therapies), as well as aminopeptidase A inhibitors and atrial natriuretic peptides, as detailed in Chart 15.2 of Chapter 15.^236,530,550-557^Nonetheless, several gaps remain to be addressed in the management of RH and RfH.

#### 12.6.4. Renal Denervation

Renal sympathetic denervation (RSD) is a procedure that uses radiofrequency or US to ablate the renal sympathetic nerves. This results in increased renal blood flow, inhibition of the RAAS, and suppression of sympathetic activity in the heart and vessels through brain signals.^558^ However, results have been highly variable, largely due to the lack of standardized and well-defined procedural protocols. Two major trials have investigated RSD in patients with RH: the Radiance-HTN Trio (US ablation) and Simplicity HTN-3 (radiofrequency ablation). The Simplicity HTN-3 trial, which included 535 participants (171 in the sham control group), did not demonstrate a significant reduction in 24-hour ambulatory SBP compared to the control group (−2.0 mm Hg; 95% CI, −5.0 to 1.1; p = 0.98).^559^ In contrast, the Radiance-HTN Trio trial showed a significant reduction of −6.3 mm Hg (95% CI, −9.3 to −3.2; p < 0.0001).^560^ A recent meta-analysis evaluating patients with uncontrolled hypertension (not exclusively those with RH) included 15 clinical trials with a total of 2,581 patients (1,723 undergoing RSD and 858 in the sham group). It found a reduction of −2.23 mm Hg (95% CI, −3.56 to −0.90; p = 0.001) in 24-hour ambulatory SBP, −6.39 mm Hg (95% CI, −11.49 to −1.30; p = 0.01) in office BP, and −6.08 mm Hg (95% CI, −11.54 to −0.61; p = 0.03) in home BP among those on antihypertensive treatment.^561^ Therefore, RSD may be considered as an adjunctive intervention to achieve BP targets in selected patients with RH and RfH, in an individualized and shared decision-making approach with the patient.^45^

**Table:** 

Key messages on resistant and refractory hypertension
RH and RfH are associated with an increased risk of CV events, renal complications, and mortality.
The diagnosis of RH and RfH requires accurate office BP measurements using adequate technique and equipment, along with the mandatory exclusion of pseudoresistance through ABPM (preferably) or HBPM.
Management of RH and RfH involves the use of three or more classes of antihypertensive medications with synergistic effects at the maximum tolerated doses.
RH is primarily characterized by RAAS hyperactivity and a hyperaldosteronism state. In contrast, RfH is mainly driven by sympathetic overactivity as its key pathophysiological component. Understanding the underlying pathophysiology leads to more effective therapeutic choices.
New medications are being studied with promising results (see Chapter 15), and the use of RSD to achieve BP targets remains controversial.

RH: resistant hypertension; RfH: refractory hypertension; CV: cardiovascular; HBPM: home blood pressure monitoring; RAAS: Renin-Angiotensin-Aldosterone System; RSD: Renal sympathetic denervation; BP: blood pressure.

**Table:** 

Recommendations for the management of resistant and refractory hypertension	Strength of recommendation	Certainty of evidence
It is recommended that patients with RH and RfH be followed in specialized hypertension centers capable of providing a multidisciplinary approach with trained professionals and appropriate diagnostic resources.	WEAK	LOW
It is recommended to investigate CV risk factors, HMOD, and secondary causes of hypertension in patients with RH and RfH.	STRONG	HIGH
ABPM is preferred to confirm the diagnosis of RH and RfH, if available; otherwise, HBPM should be used.	STRONG	MODERATE
A BP target of < 130/80 mm Hg is recommended for patients with RH or RfH.	WEAK	LOW
It is recommended to combine a RAAS blocker (ACE inhibitor or ARB) with a CCB and a thiazide diuretic, preferably in a single-pill combination.	STRONG	HIGH
It is recommended to replace short-acting thiazide diuretics (eg, hydrochlorothiazide) with long-acting thiazide-like diuretics such as chlorthalidone or indapamide.	WEAK	LOW
If hormonal side effects occur with spironolactone, replacement with eplerenone is recommended.	WEAK	HIGH
If BP remains above target levels despite the use of four medications, the fifth medication should target sympathetic overactivity: sympatholytic agents (central alpha agonists such as clonidine; alpha-blockers such as doxazosin; or beta-blockers, preferably bisoprolol, carvedilol, or nebivolol).	WEAK	LOW
Careful evaluation is recommended in specific clinical scenarios such as AF, HF, or CKD, in which the initial triple therapy may require different drug classes, including indications for BB use in the initial regimen.	STRONG	MODERATE
RSD may be considered as an adjunctive procedure to achieve BP targets in patients with RH and RfH, in an individualized and shared decision-making approach.	WEAK	LOW

ABPM: ambulatory blood pressure monitoring; BP: blood pressure; HBPM: home blood pressure monitoring; RfH: refractory hypertension; RH: resistant hypertension.

## 13. Adherence to Antihypertensive Treatment

Adherence to treatment and persistence with treatment recommendations are critical issues in the management of hypertension, affecting clinical outcomes and healthcare costs.^8^ Adherence to antihypertensive treatment includes the patient's compliance with the prescriptions and recommendations of doctors and other healthcare providers regarding pharmacotherapy and NPMs.^8,562^ In this context, treatment persistence, defined as the time from start of therapy to discontinuation of therapy, should also be considered.^563^

Studies indicate that a substantial proportion of patients with chronic CV do not adhere to prescribed treatments, pharmacotherapeutic or otherwise. A WHO publication found that approximately 50% of patients with chronic diseases do not take their medications as prescribed, leading to increased rates of hospitalization, morbidity, and mortality.^43^ One study that specifically evaluated patients in treatment for CVD revealed that low adherence could affect 40%–60% of them, particularly those prescribed polypharmacy regimens.^564^

### 13.1. Measuring Adherence

Measuring adherence to both pharmacological and nonpharmacological treatment recommendations is complex, involving both direct and indirect approaches. Direct methods primarily consist of quantifying drug levels or their metabolites in blood or urine, providing concrete evidence of intake, but they are often impractical for routine monitoring.^565^ Indirect methods are most commonly used and include patient self-reports, pill counts, pharmacy refill data, and electronic monitoring systems, the latter of which can record the date and time a medication container is opened.^566^

Each method has its advantages and limitations. For instance, self-reporting is simple and inexpensive, but can be biased due to patients’ desire to demonstrate adherence. Pill counts are also easy to implement, but can be manipulated by patients. Pharmacy refill records provide an objective measure of the continuity of medication supply, but do not confirm actual intake. Electronic monitoring is considered the gold standard among indirect methods due to its greater accuracy in capturing dosage patterns over time, but it is more costly, subject to bias if the container is opened but the medication it not actually taken, and can also be interpreted as intrusive. Combining these methods is often recommended to provide a more comprehensive assessment of adherence in both clinical and research settings.^566^

Adherence to pharmacotherapy is often quantified using various cutoff points and categorized into distinct levels, which facilitates understanding of patient behavior and treatment effectiveness. Most commonly, adherence to recommendations is defined as "high" if 80% or greater, "average" if 50–79%, and "low" when less than 50%.^566^

The WHO suggests that adherence levels above 80% are generally necessary to achieve optimal therapeutic outcomes in most chronic conditions.^43^ However, the great individual and phenotypic variability of patients with hypertension may determine different needs for minimum treatment adherence for the successful prevention of adverse outcomes.

### 13.2. Evidence

In addition to robust evidence that controlling hypertension promotes significant reductions in deaths and CV and renal events, both in primary and secondary prevention, there are also studies demonstrating the relationship between adherence to antihypertensive treatment and reduction in relevant clinical outcomes.^120,567-570^

Liu et al. evaluated the association between adherence to CV medications and the risk of CV events, stroke, and all-cause mortality, both in primary and secondary prevention.^571^ More than 4 million patients across 46 observational studies were included in the analysis, with a mean Newcastle–Ottawa quality assessment score of 7.9 (out of 9) and a mean follow-up period of 4.6 years.^569^ Regarding antihypertensive medication, dose-response analysis indicated that a 20% increase in adherence was associated with a 17% [0.83 (0.78-0.89)] reduction in the RR of stroke and a 12% reduction in the RR of death [0.88 (0.82-0.94)]. Based on the generally satisfactory quality of the primary studies, and as the only publication to evaluate the dose-dependent relationship between adherence and reduction of CV events, this review provided moderate certainty of evidence according to the GRADE system.^571^

Lee et al., in another systematic review, estimated the overall prevalence and consequences of low adherence to antihypertensive medications among adult patients with hypertension. The analysis included several methods of measuring treatment adherence and involved 161 observational studies. The results indicated that low adherence to antihypertensive medication was associated with an increased odds ratio (OR) of death [1.38 (95% CI, 1.35-1.41)]. Improved adherence to antihypertensive medication was associated with a 25% reduction [0.75 (0.73-0.76)] in the RR of death. However, these results were based on only two studies enrolling 1,653,763 patients with a mean follow-up of 4.5 years.^572^

### 13.3. Strategies

An overview of systematic reviews evaluated the evidence of effectiveness of the various strategies employed to increase adherence to antihypertensive treatment (Table 13.1). Among the strategies analyzed, the study revealed significant positive effects of interventions related to pharmaceutical care, self-monitoring, and the use of mobile applications and SMS-based alerts, which can be used in primary health care settings to improve treatment adherence in adults with hypertension. A Cochrane review reported low-certainty evidence regarding the effects of mobile phone-delivered interventions to improve adherence to medications prescribed for the primary prevention of CVD, and moderate-certainty evidence that these interventions do not result in harm.^573^

**Table 13.1:** Results achieved in terms of adherence to pharmacotherapy for hypertension through strategies involving pharmaceutical professionals, other healthcare professionals, mobile applications, and subsidies

Intervention	Outcome
Pharmacist-based	
Clinical pharmacist on team	Adherence increased by 45.4%
Pharmaceutical care services	Improved continuity and adherence
Telephone counseling and reminders	Increased adherence
Bundle including medication management, education, visits/follow-up contact	Adherence increased by 67%
Other professionals	
Group relaxation; educational booklets; contracts setting behavioral goals and rewards	Better adherence; less treatment discontinuation
Intensive interventions/counseling by CHWs	Adherence increased by 26% and 17%, respectively
Group-based training, education, telephone calls	• Adherence increased by 16% with training, and 26.4% with educational actions • 3% reduction in medication discontinuation with telephone calls
Blister packaging, case management, face-to-face education, education plus behavior support	Benefit from intervention and collaborative care
Self-monitoring, mobile apps, text messages	
Automonitoramento da pressão arterial	• Effect on adherence (SMD = 0.21; 95% CI 0.08 to 0.34; I2=43%) • No significant effect on patient adherence as determined by pharmacy refill data
eHealth, apps, telephone calls	Improved adherence • *eHealth* (RR = 0.79; 95% CI, 0.48 to 1.01; I^2^=91.3%) • Application (RR = 0.55; 95% CI, 0.33 to 0.93; P = 0.004) • Telephone calls (RR = 0.44; 95% CI, 0.09 to 2.13; I^2^= 90.7) • Blood pressure telemonitoring (RR = 0.99; 95% CI, 0.92 to 1.05; I^2^= 44.2) • E-mails (RR = 0.26; 95% CI, 0.11 to 0.61) • Website had no effect on adherence (RR = 1.01; 95% CI, 0.84 to 1.22; I^2^ = 0)
mHealth	Improved adherence, although not statistically significant
App	Improved adherence
App	Improved blood pressure and medication adherence
Home blood pressure monitoring	Similar adherence between groups at 8 weeks
Weekly text messages with reminders, self-report monitoring, appointment scheduling	Did not show benefit
Subsidized prescription medications	
Subsidies	Offering full coverage of antihypertensive medications was associated with a 9% absolute increase in adherence (P = 0.0340)

aapp: mobile application; CHW: community health worker; SMD: standardized mean difference; RR: relative risk. Adapted from: da Silva et al.^576^

In another review, reducing the number of daily doses appeared to be effective in increasing adherence to blood pressure-lowering medication and should be tried as a first-line strategy, although there is less evidence of a blood pressure-lowering effect.^574^ The work of multidisciplinary teams is essential to improving adherence to antihypertensive treatment.^575^ These interventions can be implemented alone or in combination, depending on the local context.

Barriers to implementing these interventions identified as significant included low digital literacy, limited access to technology, the cost of medications, and the health status of patients; on the provider side, they included lack of integration into workflow, insufficient human resources, incipient training, and difficult-to-use electronic systems. Significant facilitators of adherence were related to socioeconomic factors, a good relationship between patients and the health service/providers, and provider-side factors. On the health system side, the main facilitator was improved access to healthcare services.^576^

#### 13.4. Recommendations

WHO lists five groups of factors that impact adherence to treatment of hypertension: patient-related, disease-related, therapy-related, health systems-related, and socioeconomic factors.^43^ Recommendations of strategies to improve adherence to treatment of hypertension, as well as challenges in their implementation, are summarized in Figure 13.1 (adapted and modified from WHO).

**Figure 13.1:** Factors that negatively and positively influence adherence to treatment of hypertension.^43^

**Table:** 

Key messages on adherence to hypertension treatment
Increased adherence to antihypertensive pharmacotherapy reduces the risk of CV outcomes, renal outcomes, and all-cause mortality.
A 20% improvement in adherence to antihypertensive pharmacotherapy reduces the relative risk of stroke by 17% and of death from all causes by 12%.

**Table:** 

Recommendation for adherence to antihypertensive treatment	Strength of Recommendation	Certainty of Evidence
Strategies involving the work of a multidisciplinary team, subsidies, communication/education resources, and mobile applications are recommended to improve adherence to antihypertensive treatment.	WEAK	LOW

## 14. Best Practices in the Care of People With Hypertension in Primary Health Care Settings Within the Brazilian Unified Health System

### 14.1. The Brazilian Unified Health System and its Importance to Hypertension Control

Primary health care centers (*Unidades Básicas de Saúde,* UBS) are an integral part of the SUS. Based at these centers, Family Health Teams (*Equipes de Saúde da Família,* ESF) carry out a series of care activities, including prevention, diagnosis, and treatment. Primary health care (PHC) facilities represent the main gateway or point of entry to the system, meeting individual and collective needs.^577^

Federal Law nº 8,080 of September 19, 1990,^577^ establishes the conditions for the promotion, protection, and recovery of health, as well as the organization and functioning of the corresponding services, instituting the SUS. Over 30 years since its creation, the SUS, the largest publicly funded health system in the world, serves more than 190 million people a year, comprehensively and always free at the point of care, providing care of such complexity that it needs to be organized into different levels of health care and assistance to function properly. The levels of health care and assistance in Brazil are established by Ordinance 4,279 of December 30, 2010, which defines the guidelines for the organization of the Health Care Network (RAS) within the SUS: primary, secondary, and tertiary care.^578^

These levels are used to organize the treatments and other services offered by the SUS based on parameters determined by the WHO, with the aim of protecting, restoring, and preserving the health of citizens with equity, quality, and resolutive capacity. As the preferred point of entry to the SUS for users, PHC provides a setting in which the majority of health problems can be addressed, or referred for specialized (secondary and tertiary) care as needed otherwise.^578^

#### 14.1.1. Primary Health Care

PHC facilities carry out activities and provide services focused on prevention, health promotion, diagnosis, and treatment. These include tests, routine visits with multidisciplinary teams, and access to specialized family health practitioners, who work to ensure the provision of comprehensive health care in their catchment area.^578^

At the primary care level, professionals come together to work not only in healthcare facilities, but also in public spaces in the community, schools, and make house calls, as well as offer integrative and complementary practices.^578^

Beyond providing clinical care, the goal is to remain close to people in the community and promote health and quality of life within the community. Such prevention, awareness, and education work is important not only to improve quality of life, but also to optimize the utilization of healthcare resources, preventing diseases and conditions that might require hospitalization and treatment, such as hypertension, DM, sedentary lifestyle, obesity, dyslipidemia, CVD, and CKD.^578^

The pursuit of hypertension control is one of the strategic purposes of PHC. Within the SUS framework, the general practitioner or family physician, working alongside the multidisciplinary team at the corresponding level of complexity, are in charge of the care of patients with hypertension. It is up to the general practitioner/family physician to refer patients to higher levels of complexity and provide follow-up care after specialist assessment (counter-referral), with the necessary guidance provided by the specialist in a specific report.^578^

There are currently 48,161 primary health care centers in Brazil, and it is impossible to outline strategies to improve the prevention, diagnosis, treatment, and control of hypertension in the country without relying on this network. The Brazilian PHC network can provide care to an average of 564,232 patients a day. People are encouraged to seek care at the primary care facility closest to their home for essentially all of their health needs, except in life-threatening emergencies, when care should be sought at 24-hour freestanding emergency departments, general hospitals, or other intermediate- and high-complexity services designated for this purpose.^578^

#### 14.1.2. Specialist Care

Within the SUS framework, specialist care is divided into two levels: secondary and tertiary, which correspond, respectively to intermediate-complexity (usually outpatient) and high-complexity (usually inpatient) care. Intermediate-complexity care is provided by specialist services based in hospitals and outpatient clinics and involves such specialties as cardiology, nephrology, endocrinology, and neurology, among others.^578^ Such care is currently concentrated at freestanding emergency departments, which have capacity to serve 150 to 450 patients per day per facility.^578^

Another component of specialist care is the nationwide emergency medical service (EMS), SAMU 192, which aims to provide timely prehospital care to victims of urgent or emergency conditions that could lead to suffering, life-altering, or even life-ending consequences. SAMU 192 covers 85.89% of the Brazilian population, with 190 dispatch centers across the country.^578^

Within this context, PHC is the most appropriate setting for prevention, diagnosis, treatment, and control of hypertension. According to the 2019 Brazilian National Health Survey (PNS),^579^ among people aged 18 or older who reported a medical diagnosis of hypertension, 72.2% had received medical care in the last 12 months through the SUS. In 46.6% of cases, the diagnosis was made at a primary health center, and 45.11% obtained at least one medication for hypertension management via the "Aqui Tem Farmácia Popular" subsidized prescription drug program. Furthermore, in many places, patients collect their medications directly from their local primary health center.^579^

**Table:** 

Key messages about primary health care within the Brazilian Unified Health System and hypertension
PHC facilities serve as the point of entry to the Unified Health System (SUS) and provide services focused on prevention, health promotion, diagnosis, and treatment.
Beyond providing clinical care, the goal is to remain close to people in the community and promote health and quality of life within the community.
The pursuit of hypertension control is one of the strategic purposes of PHC. Within the SUS framework, the general practitioner or family physician, working alongside the multiprofessional team at the corresponding level of complexity, are in charge of the care of patients with hypertension.
There are currently 48,161 primary health care centers in Brazil, and it is impossible to outline strategies to improve the prevention, diagnosis, treatment, and control of hypertension in the country without relying on this network.
PHC is the most appropriate setting to optimize the prevention, diagnosis, treatment, and control of hypertension.

PHC: primary health care.

### 14.2. Active Case-Finding of Hypertension

Considering that hypertension is usually asymptomatic, active case-finding is widely justified, as early diagnosis and effective treatment reduce the risk of complications. Therefore, it is recommended that every adult individual (age ≥ 18 years), at every interaction with the health system, should always have their BP measured by a trained professional, preferably with a validated, automated, oscillometric arm sphygmomanometer.^46^ The BP measurement should be communicated to the individual, preferably in writing and properly expressed (eg, 134/76 mm Hg, not just "13 over 7"). Ideally, everyone should have their BP measured at least once annually, especially those with a family history of hypertension, older adults, and those with comorbidities, including DM, obesity, CVD, or CKD. The healthcare provider who obtains the BP measurement must be instructed to take at least three measurements. If the average of the last two measurements is ≥140/90 mm Hg, the person must be referred to their local primary care center for an appointment.^46^ BP measurements must be confirmed at first intake, during the actual appointment and at subsequent visits, always following the BP measurement protocol described below. The frequency of subsequent visits should be assessed individually, taking into account whether targets are being achieved and any comorbidities are under control.

### 14.3. Blood Pressure Measurement Protocol: Necessary Precautions

Proper BP measurement results in greater accuracy in the diagnosis and care of individuals with hypertension. The steps recommended below must be strictly followed with respect to the person whose BP is being measured, the person obtaining the measurement, and the equipment used to obtain it. Validated automated oscillometric arm BP monitors (as long as they are certified by INMETRO or other recognized institutions) are preferred, as they are proven to offer accurate and reliable BP measurements, are easier to handle, obviate the need for a stethoscope, require less sophisticated training, and are comparable in cost to auscultatory sphygmomanometers, the use of which requires additional precautions, particularly calibration every 6 months, which is not widely available.^46^

The necessary precautions for proper BP measurement are summarized in Figure 14.1. Following these steps ensures that diagnosis and subsequent management of hypertension will be more accurate and appropriate.^46^

**Figure 14.1:** Essential precautions for proper blood pressure measurement.

### 14.4. Diagnosis of hypertension

Individuals are considered hypertensive if office BP measurements obtained on at least two occasions, days or weeks apart, yield an SBP ≥ 140 mm Hg and/or DBP ≥ 90 mm Hg. Anyone who is already taking antihypertensive medication or has HMOD should be considered to have AH even if their BP values are <140/90 mm Hg. Individuals with an SBP 120–139 mm Hg and/or a DBP 80–89 mm Hg are considered to have prehypertension. BP is normal when <120/80 mm Hg. Hypertension is also classified into stages (1 to 3), as shown in Chart 14.1. Individuals with prehypertension must receive appropriate guidance for the primary prevention of hypertension, as described in Chapters 3 and 6.

**Chart 14.1:** Classification of BP from in-office measurement, ages 18 and older

Classification of BP	SBP (mm Hg)		DBP (mm Hg)
**Normal BP**	< 120	and	< 80
**Prehypertension**	120-139	and/or	80-89
Stage 1 hypertension	140-159	and/or	90-99
Stage 2 hypertension	160-179	and/or	100-109
Stage 3 hypertension	≥ 180	and/or	110

BP: blood pressure; SBP: systolic blood pressure; DBP: diastolic blood pressure.

* Classification follows office BP and the highest BP level, either systolic or diastolic.

** Isolated systolic hypertension, characterized by SBP ≥ 140 mm Hg and DBP < 90 mm Hg, is classified into stage 1, 2, or 3 according to SBP values at the intervals indicated.

*** Isolated diastolic hypertension, characterized by SBP < 140 mm Hg and DBP ≥ 90 mm Hg, is classified into stage 1, 2, or 3 according to SBP values at the intervals indicated.

**Table:** 

Recommendations for blood pressure measurement	Strength of recommendation	Certainty of evidence
It is recommended that prehypertension be classified as SBP between 120-139 mm Hg or DBP between 80-89 mm Hg in the office, with the aim of early identification of at-risk individuals and to encourage proactive, non-pharmacological interventions to prevent progression to hypertension.	STRONG	MODERATE
It is recommended that the diagnosis of hypertension be made when office BP is ≥ 140 and/or 90 mm Hg on two separate occasions, and classified into stages 1, 2, or 3 according to the highest SBP or DBP value.	STRONG	MODERATE
BP measurements must be obtained using proper technique and equipment.	STRONG	MODERATE
The use of automated devices reduces errors and makes it easier to obtain BP measurements.	STRONG	MODERATE
It is recommended to assess for orthostatic hypotension in at-risk groups (older adults, patients with diabetes, autonomic dysfunction, or using antihypertensive medications).	STRONG	HIGH
It is recommended to use ABPM or HBPM to confirm the diagnosis of hypertension and monitor treatment	STRONG	HIGH

ABPM: ambulatory blood pressure monitoring; BP: blood pressure; DBP: diastolic BP; HBPM: home blood pressure monitoring; NPM: nonpharmacological measures; SBP: systolic BP.

### 14.5. Clinical and Complementary Assessment of the Patient with Hypertension

Once the diagnosis of hypertension has been established, the patient must undergo a clinical, laboratory, and imaging workup.^4,45^ This assessment aims to: 1. Identify potential systemic complications of hypertension; 2. Identify the CVRFs associated with hypertension; and 3. Screen for the main secondary causes of hypertension, on the basis of clinical and laboratory findings. Chart 14.2 describes the key points of clinical assessment.

**Chart 14.2:** Initial clinical assessment of the patient with hypertension^1,45^

History:
• Disease duration, symptoms (rare), presence of risk factors for primary hypertension (age, biological sex, race/skin color, heredity/family predisposition, overweight, excess salt intake, sedentary lifestyle, alcohol use, tobacco smoking). • Absence of risk factors (10%) in individuals with severe hypertension or hypertension that is resistant to treatment with three antihypertensive agents at maximum dose suggests secondary causes (primary kidney disease, renal artery stenosis, primary hyperaldosteronism, pheochromocytoma, Cushing syndrome, etc.). See Chapter 4. • The review of systems may reveal hypertension-mediated organ damage to the heart, kidneys, brain, retina, and peripheral arteries (the "target organs" of hypertension). Patients should be screened for dyspnea on exertion, edema, nocturia, anemia (indicative of heart failure and/or kidney failure), chest pain on exertion (indicative of myocardial ischemia), and intermittent claudication (indicative of peripheral arterial insufficiency).
Physical Examination:
• Anthropometric measurements: weight, height, BMI (weight/height^2^), waist circumference, mid-upper arm circumference, and careful measurement of BP in the sitting, lying, and standing positions. • Cardiovascular and respiratory system: apex beat displaced to the left, presence of a 3rd and/or 4th heart sound, pulmonary crackles, lower-extremity edema, hepatomegaly, arrhythmias (extrasystole and atrial fibrillation), loud second heart sound (A2 and/or P2) and valve murmurs, all of which may be secondary to enlargement of the chambers of the heart. • Comparative palpation of the carotid, radial, abdominal aortic, dorsalis pedis, and posterior tibial pulses may demonstrate evidence of peripheral artery disease. Abdominal or flank bruits may be indicative of renal artery stenosis.

BP: blood pressure; BMI: body mass index.

In addition to clinical examination, patients with hypertension require a minimum laboratory workup evaluation that must be repeated annually (additional tests may be ordered as indicated) (Chart 14.3). These tests are available at all primary health centers and are essential for detecting the most common causes of secondary hypertension, HMOD, and CVRFs associated with hypertension^1,45^ (Chapter 4).

**Chart 14.3:** Minimum workup for the patient with hypertension

Complementary tests	Indication
Urinalysis	Detect primary renal disease (urinary protein-to-creatinine ratio > 1.0 g/g; glomerular hematuria with red cell dysmorphism or red blood cell casts) or renal impairment (proteinuria or albuminuria)
Albumin-to-creatinine ratio in spot urine sample	Assess early renal impairment and HMOD Abnormal if > 30 mg/g
Plasma potassium	Decreased: assist in the diagnosis of primary or secondary hyperaldosteronism and/or diuretic use Increased: kidney failure and/or ACEI, ARB, MRA use
Plasma creatinine	Assess renal function: estimate the glomerular filtration rate using the CKD-EPI formula; adjust diuretic therapy; assess CV risk
Fasting blood glucose and HbA1c	Diagnose diabetes mellitus and assess glycemic control; access CV risk
Serum total cholesterol, HDL-c, and triglycerides*	Diagnose and manage dyslipidemias; assess CV risk
Uric acid	Assess CV risk, particularly in men
Standard electrocardiogram**	Assess cardiac involvement (hypertrophy, old myocardial infarction) and cardiac arrhythmias; assess CV risk

ACEI: angiotensin-converting enzyme inhibitors; ARB: angiotensin II receptor blockers; CKD-EPI: Chronic Kidney Disease-Epidemiology Collaboration; CV: cardiovascular; HMOD: hypertension-mediated organ damage; MRA: mineralocorticoid receptor antagonists.

* LDL cholesterol is calculated using the formula: LDL-c = total cholesterol – (HDL-c + triglycerides/5) (if the triglyceride level is below 400 mg/dL).

** Criteria for diagnosis of left ventricular hypertrophy (LVH): Sokolow-Lyon: SV1 + RV5,6 > 35 mm; Cornell voltage criteria: RaVL + SV3 > 20 mm (female), > 28 mm (male).

### 14.6. Risk Stratification after Clinical Assessment and Workup

Integration of data from clinical assessment and complementary tests allows stratification of individuals with hypertension into risk categories: "no additional risk"; "low risk"; "moderate risk" and "high risk" or "very high risk". Whenever possible, it is recommended that CV risk be assessed with the AHA PREVENT formula,^580^ which is available online via the following link: https://professional.heart.org/en/guidelines-and-statements/prevent-calculator. If this is unavailable, CV risk may be stratified as described in Chart 14.4. This risk stratification is the basis for defining individual needs, subsequent care pathways, priorities, and the range of services to be provided to people with hypertension within PHC.

**Quadro 14.4:** Classificação dos estágios de HA de acordo com o nível de PA, presença de FRCV, LOA ou DCV estabelecida

FRCV, presença de LOA ou DCV	BP (mm Hg)			
	Prehypertension SBP 130-139 DBP 85-89	Stage 1 SBP 140-159 DBP 90-99	Stage 2 SBP 160-179 DBP 100-109	Stage 3 SBP ≥ 180 DBP ≥ 110
No CV risk factors	Low risk	Low risk	Moderate risk	High risk
1 or 2 CV risk factors	Low risk	Moderate risk	High risk	High risk
≥ 3 CV risk factors	Moderate risk	High risk	High risk	High risk
HMOD, stage 3 CKD, or DM	High risk	High risk	High risk	Very high risk
Established CVD or stage ≥ 4 CKD	Very high risk	Very high risk	Very high risk	Very high risk

BP: blood pressure; CKD: chronic kidney disease; CV: cardiovascular; CVD: cardiovascular disease (myocardial infarction, heart failure, stroke, peripheral vascular disease); DBP: diastolic blood pressure; DM: diabetes mellitus; SBP: systolic blood pressure; HMOD: hypertension-mediated organ damage. CV risk factors considered in this analysis: Male sex; age: > 55 years for men and > 65 years for women; premature CVD in first-degree relatives (men < 55 years and women < 65); smoking; dyslipidemia: LDL-cholesterol ≥ 100 mg/dL and/or non-HDL cholesterol ≥ 130 mg/dL and/or HDL-cholesterol ≤ 40 mg/dL in men or ≤ 50 mg/dL in women and/or triglycerides ≥ 150 mg/dL; obesity: body mass index ≥ 30 kg/m².

**Table:** 

Recommendations for clinical and complementary assessment and subsequent risk stratification of individuals with prehypertension and hypertension	Strength of recommendation	Certainty of evidence
It is recommended to obtain the patient's medical history and perform a physical examination to obtain a more accurate diagnosis and help identify secondary causes of hypertension and HMOD.	STRONG	LOW
It is recommended to assess CV risk factors and HMOD ideally in all patients at the time of hypertension diagnosis and repeat this assessment at least annually, with the choice of method depending on available resources.	STRONG	LOW
It is recommended to assess for kidney disease and classify it according to KDIGO 2024 guidelines.	STRONG	MODERATE
It is recommended to assess CV risk using the PREVENT score (Predicting Risk of CVD Events).	STRONG	HIGH
It is recommended to stratify CV risk in individuals with prehypertension to guide the initiation of antihypertensive treatment and improve CV risk factor control.	STRONG	HIGH
It is recommended to stratify CV risk in patients with hypertension to allow for a more accurate and personalized approach to pharmacological therapy and goal setting for CV risk factor control.	STRONG	HIGH

CV: cardiovascular; KDIGO: Kidney Disease Improving Global Outcomes; HMOD: hypertension-mediated organ damage; PREVENT: Predicting Risk of Cardiovascular Disease Events.

### 14.7. Blood Pressure Targets

The most recent studies have shown significant reductions in CV and CKD risk when BP is controlled to lower levels. Therefore, overall and regardless of CV risk, this Guideline recommends a BP target of < 130/80 mm Hg for individuals with hypertension.^366,581^ Some individuals may not tolerate such low BP values. In these cases, titration to the lowest tolerable levels should be pursued.

**Table:** 

Recommendations for blood pressure targets	Strength of recommendation	Certainty of evidence
For patients with BP 130-139/80-89 mm Hg and high CV risk, a BP target of < 130/80 mm Hg is recommended.	STRONG	HIGH
For patients with any hypertension and low or moderate CV risk, a BP target of < 130/80 mm Hg is recommended.	STRONG	HIGH
For patients who do not tolerate the < 130/80 mm Hg BP target, BP should be reduced to the lowest level tolerated	STRONG	MODERATE
It is recommended to confirm the achievement of BP targets with out-of-office BP measurement using ABPM or HBPM.	STRONG	LOW

BP: blood pressure; CV: cardiovascular.

### 14.8. Out-of-Office/Non-Healthcare Setting Blood Pressure Measurement

It consists of ABPM and HBPM. ABPM is performed with devices that take automatic BP measurements at intervals of 15–20 minutes when the individual is awake and 20–30 minutes during sleep, while HBPM is measured with validated, calibrated, automated oscillometric devices that allow retrieval of the BP values recorded in the device's memory.^46^ The availability of automated oscillometric monitors at an acceptable cost has increasingly allowed BP measurements to be obtained in non-healthcare settings, which can be very useful in the diagnostic approach to people with suspected hypertension and in the management of those already in treatment for hypertension.

Chart 14.5 lists the normal values and indications for these out-of-office BP measurement modalities.^46^ Both are approved for use within the SUS, although exclusively for diagnostic purposes.^582^ This Guideline proposes that local health managers ensure that HBPM is also made available to individuals with the other classical indications for this methos: assessment of antihypertensive drug treatment efficacy; diagnosis of HR (off-target BP despite optimized therapy with 3 classes of antihypertensive medications having synergistic effects at the maximum tolerated doses); and evidence of progression of HMOD despite well-controlled BP, as described in Chapter 12 of this Guideline and the Brazilian Ministry of Health Ordinance which incorporated HBPM into the SUS.^582^

**Chart 14.5:** Normal BP values and indications for ABPM and HBPM^46^

Normal values:	ABPM < 135/85 mm Hg (awake) < 120/70 mm Hg (asleep) < 130/80 mm Hg (24-hour)	HBPM 4–6 days in 1 week 3 morning measurements and 3 nighttime measurements per day < 130/80 mm Hg (average)
Indications for ABPM and HBPM		
Diagnosis of white-coat hypertension	+ + +	+ +
Diagnosis of masked hypertension	+ + +	+ +
Monitoring of antihypertensive pharmacotherapy	+ +	+ + +
Diagnostic confirmation of resistant hypertension	+ + +	+ +
Absence of nighttime BP dipping	+ + +	Not applicable

ABPM: ambulatory blood pressure monitoring; HBPM: home blood pressure monitoring. The greater the number of crosses, the stronger the indication for ABPM or HBPM in that setting.

**Table:** 

Recommendations for ABPM or HBPM^66,67^	Strength of recommendation	Certainty of evidence
ABPM or HBPM are recommended to confirm the diagnosis of hypertension and to monitor treatment.	STRONG	HIGH

ABPM: ambulatory blood pressure monitoring; HBPM: home blood pressure monitoring.

### 14.9. Cornerstones of Management for Individuals with Hypertension

Hypertension is a highly prevalent, multifactorial, complex disease which, although asymptomatic, leads to serious health consequences. To achieve adequate disease control, patients and their families must understand the importance of keeping BP within recommended targets, the principles of treatment, the need for NPMs, the proper and uninterrupted intake of prescribed medications, and regular monitoring by the care team. These goals will only be met if the care provided by the multidisciplinary team is supplemented by patient self-management and adherence, which also involves family support.^1^

Multidisciplinary teams facilitate and enhance the effectiveness of this endeavor, both in controlling BP and in reducing the morbidity and mortality associated with hypertension, which are the primary goals of treatment. The multidisciplinary approach improves the quality of care, promotes greater adherence to pharmacotherapy and NPMs, places the patient at the center of decision-making, and humanizes care, increasing the rate of patients with controlled BP to 68%.^573,583,584^ The multidisciplinary care model requires integration, agility, and an openness to communication across the entire team. Each healthcare provider must recognize their specific, complementary role in this chain in pursuit of the best possible care. The multidisciplinary team may be composed of: a) general practitioner/family physician; b) nurse, c) community health worker; d) dietitian; e) physical educator/physical therapist; f) pharmacist; g) psychologist; h) social worker; and i) relatives/family members (Figure 14.2).^585^

**Figure 14.2:** The SUS care model and composition of the multidisciplinary team.

Each municipality and each health facility must adapt to local needs and possibilities and bring together a team which is as diverse as possible. Evidence suggests that a multidisciplinary approach adds benefit in terms of treatment effectiveness and, consequently, leads to better outcomes.^194^ In this sense, educational actions that empower patients and respect their individual needs and those of the community are fundamental to improving adherence to pharmacological and nonpharmacological treatment measures.^586^

NPMs are one of the cornerstones of hypertension management. Key interventions, their effects, and recommendations are presented in Chart 14.6.

**Chart 14.6:** Effect of nonpharmacological measures for blood pressure management (Chapter 6)

Nonpharmacological Measure	Approximate Reduction in SBP/DBP	Recommendation
Smoking cessation	Results are controversial	Universally recommended Refrain from use of tobacco products, including hookah/shisha and e-cigarettes Cutting down is not enough
Weight loss	On average, every 1-kg reduction in body weight reduces SBP by 1.05 mm Hg and DBP by 0.92 mm Hg	Maintain a BMI < 25 kg/m^2^ up to age 65 years Maintain a BMI between 22 and 27 kg/m^2^ after 65 years of age
Dietary pattern	DASH diet reduces SBP by 8.7 mm Hg and DBP by 4.5 mm Hg	Diet rich in vegetables and fruits, whole grains, low-fat dairy products, and white meats (DASH diet)
Increased potassium intake	Reduces SBP by 4.8 mm Hg and DBP by 3.0 mm Hg	Diet rich in fruits and vegetables (ideally > 3.5 g/day)
Reduction of dietary salt intake	Every 1.15 g/day reduction in sodium intake decreases SBP by 2.8 mm Hg and DBP by 1.4 mm Hg	Limit to 2 g sodium/day (5 g table salt/day). Natural foods already provide 2 g salt/day)
Reduction of alcohol intake	A 50% reduction for those who consume 6 units/day results in a 5.5 mm Hg decrease in SBP and a 4.0 mm Hg decrease in DBP	Limit alcohol intake to 1 standard drink for women and 2 standard drinks for men
Physical exercise	Aerobic: 7.6/4.7 mm Hg reduction Combined (resistance/dynamic): 5.3/5.6 mm Hg reduction	Aerobic training: 3 to 5 times a week 30 to 60 minutes per session or 150 minutes a week

BMI: body mass index; DASH: Dietary Approaches to Stop Hypertension; DBP: diastolic blood pressure; HR: heart rate; SBP: systolic blood pressure. 1 unit or standard drink = 10 to 12 g of pure alcohol (200 mL beer, 100 mL wine, 25 mL spirits).

Other recommended interventions for BP management, but with less robust levels of evidence, include meditation practice, slow breathing, and encouraging spirituality or religiosity.^1^

**Table:** 

Recommendations of nonpharmacological measures for blood pressure reduction	Strength of Recommendation	Certainty of Evidence
Smoking cessation is recommended to reduce CV events and mortality	STRONG	HIGH
Reducing body weight is recommended for BP and mortality reduction in patients with obesity	STRONG	HIGH
Reducing sodium intake and increasing dietary potassium intake (except for patients with CKD) is recommended for BP reduction	STRONG	HIGH
Limiting daily maximum alcohol consumption is recommended for BP reduction	STRONG	MODERATE
The DASH diet and regular moderate physical activity are recommended for BP and mortality reduction.	STRONG	HIGH
Regular moderate physical activity is recommended for BP and mortality reduction	STRONG	HIGH
Aerobic training is recommended for BP reduction	STRONG	HIGH
Meditation is recommended for BP reduction	STRONG	MODERATE
Slow breathing is recommended for BP reduction	NEUTRAL	LOW
Addressing aspects of spirituality and religiosity is recommended for hypertension management	NEUTRAL	LOW
A multidisciplinary team approach is recommended for improved BP control	STRONG	LOW

BP: blood pressure; CKD: chronic kidney disease; DASH: Dietary Approaches to Stop Hypertension; CV: cardiovascular.

Nonpharmacological and pharmacological treatment should be initiated as described in Chart 14.7.

**Chart 14.7:** Indications for initiation of nonpharmacological and pharmacological treatment according to BP level, age, and CV risk

Treatment	Target population	Treatment initiation
Nonpharmacological measures	BP ≥ 120/80 mm Hg	At diagnosis
Drug therapy	BP ≥ 140/90 mm Hg	At diagnosis
	BP 130-139/80-89 mm Hg and high CV risk	When BP is not controlled after 3 months of non-pharmacological measures

BP: blood pressure; CV: cardiovascular.

**Table:** 

Recommendations for initiation of treatment with nonpharmacological measures and pharmacotherapy	Strength of recommendation	Certainty of evidence
NPMs are recommended for all individuals with blood pressure (BP) ≥ 120/80 mm Hg.	STRONG	HIGH
Pharmacological treatment is recommended after 3 months of NPMs for individuals with BP 130-139/80-89 mm Hg and high CV risk.	STRONG	HIGH
Initiation of pharmacological treatment is recommended for individuals with BP ≥ 140/90 mm Hg.	STRONG	HIGH

BP: blood pressure; CV: cardiovascular; NPM: nonpharmacological measure.

### 14.10. Pharmacotherapy

All classes of antihypertensive drugs are available in the SUS, and can be used depending on the clinical particulars of each individual with hypertension^1^ (Chart 14.8).

**Chart 14.8:** Antihypertensive drug classes and agents available in the Unified Health System core pharmaceutical care component and "Farmácia Popular" Program with dosage strengths, daily doses, and frequency of administration

CLASSES AND AGENTS	Dosage strength (mg)	Core Component	Farmácia Popular Program	Dosage, daily (mg)	Freq /Day	Important notes
**ORAL DIURETICS**						
Spironolactone	25	x	x	25-100	1 to 2	May cause hyperkalemia, particularly in CKD and when combined with ACEI or ARB
Spironolactone	100	x			1 to 2	
Furosemide	40	x	x	20-240	1 to 3	Used in edematous states, eg, in CKD and HF
Hydrochlorothiazide	12.5	x		25-50	1	
Hydrochlorothiazide	25	x	x	25-50	1	
**CALCIUM CHANNEL BLOCKERS (CCB)**						
Verapamil hydrochloride	80	x		120-360	1 to 2	Avoid in patients with HFrEF. Avoid in combination with BB and in patients with bradycardia.
Verapamil hydrochloride	120	x		120-360	1 to 2	
Amlodipine	5	x		2.5 a 10	1	Avoid in patients with HFrEF. May cause dose-dependent lower-extremity edema.
Amlodipine	10	x		2.5 a 10	1	
Nifedipine	10	x		10 to 60	1 to 3	
**ANGIOTENSIN-CONVERTING ENZYME INHIBITORS (ACEI)**						
Captopril	25	x	x	25-150	2 to 3	Avoid in women of childbearing age, as there is a high risk of fetal malformations and other complications during pregnancy. Contraindicated in combination with ARBs. Risk of hyperkalemia in patients with HF or when taken with MRAs or potassium supplements.
Enalapril maleate	5	x		5-40	1 to 2	
Enalapril maleate	10	x	x	5-40	1 to 2	
Enalapril maleate	20	x		5-40	1 to 2	
**ANGIOTENSIN II RECEPTOR BLOCKERS (ARB)**						
Losartan potassium	50	x	x	50-100	1 to 2	Same recommendations as for ACEIs.
**BETA BLOCKERS (BB)**						
Atenolol	25		x			Indicated in patients with coronary artery disease (angina and status post MI), HF, atrial fibrillation, migraine, women planning pregnancy. Avoid in patients with COPD, > 2nd degree heart block, and peripheral artery disease. Abrupt withdrawal of BBs should be avoided, as it may cause reflex tachycardia and malaise.
Atenolol	50	x		50-100	1 to 2	
Atenolol	100	x		50-100	1 to 2	
Propranolol hydrochloride	10	x		80-320	2 to 3	
Propranolol hydrochloride	40	x	x	80-320	2 to 3	
Metoprolol succinate	25	x	x	50-200	1	
Metoprolol succinate	50	x		50-200	1	
Metoprolol succinate	100	x		50-200	1	
Metoprolol tartrate	100	x		50-200	1	
**PERIPHERAL SYMPATHOLYTIC AGENTS**						
Doxazosin mesylate	2	x		1-16	1	Start with a low dose at bedtime due to risk of orthostatic hypotension Increase every 2 days.
Doxazosin mesylate	4	x		1-16	1	
**CENTRAL SYMPATHOLYTIC AGENTS**						
Methyldopa	250	x		500-2000	2	Indicated preferably in pregnant women.
**DIRECT VASODILATORS**						
Hydralazine hydrochloride	25	x		50 to 200	2 to 3	May cause sodium and fluid retention, hypervolemia, and reflex tachycardia. Preferably used in combination with diuretics and/or beta-blockers. High doses associated with lupus-like syndrome.
Hydralazine hydrochloride	50	x		50 to 200	2 to 3	

ACEI: angiotensin-converting enzyme inhibitors; ARB: angiotensin II receptor blockers; BB: beta blockers; CCB: calcium channel blockers; CKD: chronic kidney disease; HF: heart failure; MI: myocardial infarction; MRA: mineralocorticoid receptor antagonists.

The MedSUS mobile application, which contains information on all medications available through the pharmaceutical care component of the SUS, is available for iOS and Android devices and can be downloaded by the general population and healthcare professionals. One of the main features of the application is to provide information on available medications aligned, as necessary, with SUS Clinical Practice Guidelines and the International Statistical Classification of Diseases and Related Health Problems (ICD). In addition, MedSUS provides information on where medicines are dispensed, such as dispensaries at primary health care units, High-Cost Pharmacies (part of the SUS's Specialized Pharmaceutical Care Component), and retail drugstores accredited by the Farmácia Popular Program.^587^

Monotherapy is only justified in cases of low-risk stage 1 hypertension, the oldest old (age > 80 years), frail individuals, those with orthostatic hypotension, or those with high or very high risk prehypertension (Chart 14.4, Figure 14.3 and Figure 7.3 – Chapter 7).

**Figure 14.3:** Flowchart for pharmacological treatment of hypertension.

Combination therapy is more effective, allows BP targets to be achieved more quickly, reduces therapeutic inertia, and is safe and recommended by most guidelines.^245,249-251,259^ It is recommended that treatment begin with a combination of an ACEI or ARB and a thiazide diuretic or CCB. A Brazilian study of SUS patients with stage 2 or 3 hypertension patients treated at university hospitals used a combined escalated treatment regimen consisting of a diuretic and an ACEI or ARB followed by amlodipine; at the end of 12 weeks, 85% of patients had achieved BP control.^259^ Full doses of the two-drug combination should be attempted before starting a third (Chapter 7). Beta blockers can be used in specific cases (status post MI, angina, HF, tachyarrhythmias, women of childbearing potential, and intolerance to previous antihypertensive therapies). The choice of drug should take the patient's comorbidities into account.

If BP remains uncontrolled despite triple-drug therapy (preferably including a diuretic) at maximum tolerated doses, the patient is considered to have RH. In this case, it is recommended that the fourth drug preferably be spironolactone (25 to 50 mg/day), followed by a sympatholytic (methyldopa 500 to 1500 mg/day or clonidine 0.200 to 0.600 mg/day) or BB (carvedilol 6.25 to 50 mg/day, metoprolol 50 to 200 mg/day, or atenolol 50 to 100 mg/day), if BP control is not achieved or if there are adverse reactions to spironolactone (see Chart 7.2 and Chapter 7 and Chapter 12) (Figure 14.3).

**Table:** 

Recommendations for drug therapy of hypertension in the Brazilian Unified Health System	Strength of Recommendation	Certainty of Evidence
The combination of antihypertensive drugs, preferably in a single-pill formulation and using preferred classes, is recommended to achieve strict BP targets (< 130/80 mm Hg) and reduce CV and renal events.	STRONG	MODERATE
Monotherapy is recommended for individuals with BP 130-139/80-89 mm Hg and high CV risk; patients with stage 1 hypertension and low risk (combination therapy may be considered at the physician's discretion); frail individuals; oldest-old adults (≥ 80 years); or those with symptomatic orthostatic hypotension, particularly in older adults.	WEAK	LOW
For most patients, initiation of treatment for hypertension with a two-drug regimen, preferably in a single pill combination, is recommended.	STRONG	MODERATE
Thiazide diuretics, ACEIs or ARBs, and CCBs are recommended as preferred classes for the treatment of hypertension and for reducing major CV and renal events.	STRONG	HIGH
BBs are recommended for the treatment of hypertension in specific situations: HF, AF, arrhythmias, CAD, hypertension in patients on hemodialysis, and other conditions (eg, migraine, essential tremor, women planning pregnancy, esophageal varices).	STRONG	MODERATE
Spironolactone (or eplerenone in cases of intolerance) is recommended to achieve strict BP targets (<130/80 mm Hg) and reduce CV and renal events when these are not reached with initial classes alone (RH and RfH).	STRONG	HIGH

ACEI: angiotensin-converting enzyme inhibitor; AF: atrial fibrillation; ARB: angiotensin II type 1 receptor blocker; BP: blood pressure; CAD: coronary artery disease; CCB: calcium channel blocker; CV: cardiovascular; HF: heart failure; RfH: refractory hypertension; RH: resistant hypertension.

The AHA recently developed the construct of *overall cardiovascular health*, which resembles comprehensive health care, one of the pillars of the SUS. It consists of eight readily verifiable clinical items known as "Life's Essential 8" (healthy diet, physical activity, avoidance of nicotine, healthy sleep, healthy weight, and healthy levels of BP, blood glucose, and blood lipids) as CV and renal protection goals, plus mental health and well-being and the social determinants of health.^588^ These 10 components should be considered by the care team, particularly in people with hypertension.

**Table:** 

Key messages: diagnosis, clinical assessment and complementary workup, blood pressure targets, and treatment of hypertension
Every adult (aged ≥ 18 years), at every interaction with the health system, should have their BP measured by a trained professional, preferably using a validated, automated, oscillometric arm sphygmomanometer.
The diagnosis of hypertension is defined by an SBP ≥ 140 mm Hg and/or DBP ≥ 90 mm Hg on office BP measurements obtained on at least two occasions.
Out-of-office BP measurement (ABPM or HBPM) should be used for diagnosis and treatment monitoring whenever possible.
Every patient with hypertension must undergo a minimum clinical assessment and workup; this assessment should be repeated annually. This information allows estimation of individual CV risk.
The recommended BP target is < 130/80 mm Hg.
Emphasis should be placed on non-pharmacological treatment measures, which include smoking cessation, weight loss, healthy eating, increased potassium intake, reduced salt and alcohol intake, and regular exercise.
For the vast majority of patients, drug treatment should consist of combination therapy. Monotherapy is only indicated for patients aged ≥ 80 years, frail older adults, those with low-risk stage 1 hypertension, or those with high- or very high-risk prehypertension.
The main classes of antihypertensive agents are thiazide diuretics, calcium channel blockers, ACE inhibitors, and angiotensin II receptor blockers.
A multidisciplinary approach improves the quality of care, promotes greater adherence to pharmacotherapy and non-pharmacological measures, places the patient at the center of decision-making, and humanizes care.

ABPM: ambulatory blood pressure monitoring; ACE: angiotensin-converting enzyme; BP: blood pressure; CV: cardiovascular; DBP: diastolic blood pressure; HBPM: home blood pressure monitoring; SBP: systolic blood pressure.

### 14.11. Clinical Conditions that Justify Specialist Referral

To ensure the sustainability of the health system and increase its efficiency, specialist referrals must follow objective criteria and should be intended to supplement and enhance the care provided in PHC settings. Specialist referrals are not necessarily definitive and, in most cases, should only occur for an occasional consult or joint patient follow-up; counter-referral back to primary care should be the rule. Having a single electronic medical record facilitates this interaction and should be a goal for all municipalities.

Chart 14.9 lists the most common conditions that justify referral of the patient with hypertension for specialist care. Alternatives increasingly used in many municipalities, with good results, are matrix support with specialists or teleconsultations between the general practitioner/family physician and specialists. These alternatives also play a role in further training of PHC providers, thus improving the resolution capacity of the first level of care.

**Chart 14.9:** Clinical conditions that justify referral for specialist care

Conditions that justify referral for specialist care	Specialist(s)
Diagnostic workup of suspected secondary hypertension	Nephrologist Endocrinologist Cardiologist
Left ventricular hypertrophy, heart failure, atrial fibrillation, angina, pre-existing coronary artery disease	Cardiologist
Kidney disease (estimated glomerular filtration rate < 30 mL/min/1.73 m^2^ and/or urinary protein-to-creatinine ratio ≥ 300 mg/g)	Nephrologist
Intermittent claudication, abdominal or carotid bruit, pulsatile mass suggestive of aortic aneurysm	Vascular surgeon
Resistant hypertension (uncontrolled despite triple-drug therapy at maximum tolerated doses)	Cardiologist Nephrologist Endocrinologist (if endocrine causes are suspected)
Diabetes mellitus with retinopathy, neuropathy, or HbA1C > 10%	Endocrinologist Ophthalmologist
Assessment of indication for bariatric surgery (BMI ≥ 35 with comorbidities)	Endocrinologist
Stroke or dementia not previously investigated	Neurologist Geriatrician (for older adults)

BMI: body mass index; HbA1C: glycated hemoglobin.

### 14.12. Counter-Referral

Counter-referral protocols for patients with hypertension from secondary and tertiary care back to PHC vary according to local health guidelines, but to ensure optimal patient care, must include all the steps listed in Chart 14.10.^589^

**Chart 14.10:** Essential elements of counter-referral

- After clinical assessment and complementary testing, stabilization to the greatest extent possible and definition of a long-term treatment plan, the specialist must refer the patient back to primary care (counter-referral) as the most appropriate setting for their subsequent follow-up.
- The specialist must send a detailed report to the primary care team containing all relevant information on the patient's clinical picture, the results of any tests performed in secondary or tertiary care, medications prescribed, a personalized treatment plan, dietary and lifestyle guidelines, and the suggested frequency of follow-up in primary and, if necessary, secondary care.
- Establish an ongoing communication channel between specialists and the primary care team to discuss complex cases and adjust treatment as needed.
- Ensure that the patient has scheduled follow-up appointments in primary and, if necessary, secondary care.

### 14.13. Telemedicine in the Brazilian Unified Health System Care Pathway for Hypertension

Telemedicine is designed to provide remote healthcare services through the use of information and communication technologies, such as videoconferencing, telephone calls, and messaging applications. It has emerged as a powerful tool to support ongoing, personalized management of hypertension in primary care within the SUS, with benefits for the care team and users alike.^590-592^

The incorporation of telemedicine into the SUS hypertension care pathway represents an evolution of the care model, allowing for closer, ongoing, efficient follow-up of these patients. Chart 14.11 summarizes potential indications for the use of telemedicine in monitoring patients with hypertension, which include expanding, complementing, and streamlining the care already provided by the multidisciplinary team, encouraging self-management, and family support, all while respecting ethical aspects of care.

**Chart 14.11:** Key indications for the use of telemedicine in primary care within the Brazilian Unified Health System

Indications	Description
Identification and registration of patients with hypertension	Enables screening of people with risk factors for hypertension, active case-finding, diagnosis, and early stratification of cardiovascular and renal risk. Diagnosed patients can be registered in the SUS health information system.
Remote monitoring	Regular monitoring using home BP monitoring devices; guidance on how to measure BP properly and send results to the care team via SUS applications or platforms. Allows the setting of automated alerts to identify significant variations in BP requiring immediate intervention.
Teleconsultation and remote guidance	Periodic teleconsultations to assess treatment adherence, adjust medications, and provide guidance on non-pharmacological measures. Health education on the importance of controlling BP, following a proper diet, engaging in physical activity, and meeting individual needs.
Multidisciplinary approach	Allows different providers (dietitians, psychologists, social workers) to offer additional support to the patient. The collected data must be integrated into the patient's electronic medical record if one is available, to facilitate an expanded approach to treatment.
Clinical decision support and follow-up	Discussion and clinical decision support between the general practitioner/family physician/primary care team and specialists.
Longitudinal follow-up and continuity of care	Allows development of personalized care plans that can be reviewed and updated remotely, ensuring continuity of care over time.

BP: blood pressure; SUS: Brazilian Unified Health System.

**Table:** 

Key messages on referral and counter-referral of patients with hypertension and the use of telemedicine
To ensure the sustainability of the health system and increase its efficiency, referrals to specialist care must follow objective criteria and seek to complement and enhance the services provided by primary care.
After clinical assessment and complementary testing, stabilization to the greatest extent possible, and definition of a long-term treatment plan, the specialist must refer the patient back to primary care (counter-referral), which is the most appropriate setting for their subsequent follow-up.
The general practitioner/family physician working in primary care remains responsible for the patient.
The incorporation of telemedicine into the SUS hypertension care pathway represents an evolution of its care model, allowing for closer, ongoing, efficient follow-up of these patients.

SUS: Brazilian Unified Health System.

## 15. Future Prospects

### 15.1. The Exposome and Hypertension

The term exposome was coined to describe the overall impact of environmental exposures on an individual's health and well-being.^593^ Several factors that make up the exposome, such as ambient temperature, ambient air pollution, noise pollution, availability of green spaces, and walkability, have been implicated in the development of hypertension (Figure 15.1).

**Figure 15.1:** Relationship between components of the exposome and hypertension.

Lower ambient temperatures promote BP elevation, while warmer temperatures induce BP reductions.^41,594^ These phenomena help explain the elevations in BP observed during winter and reductions in BP observed in the summer.^41,594^ Furthermore, cities with warmer average temperatures have a higher prevalence of normotension and WCH, whereas cities with colder average temperatures have a higher prevalence of sustained hypertension and MH.^41^ As Brazil and the world undergo striking climate change and a growing frequency of extreme weather events, it is expected that the influence of ambient temperature will play an increasing role in the management of hypertension in the coming years.

Several studies have shown that high levels of air pollution are followed by a higher incidence of hypertension.^595^ Specifically, high airborne concentrations of fine particulate matter (PM2.5) and nitrogen oxides (NOx) are strongly associated with increased BP levels and a higher incidence of hypertension.^595^ Likewise, a higher prevalence of hypertension is seen in areas with higher noise pollution.^596^ In contrast, greater presence of vegetation or green spaces has been associated with a lower risk of hypertension.^597^

Walkability is a term that describes the aspects that influence walking in one's neighborhood. Walkable neighborhoods encourage physical activity as a form of travel and, therefore, have been associated with a lower prevalence of hypertension.^593^

This dataset suggests that strategies aimed at reducing individuals’ exposure to ambient air pollution and noise pollution while increasing availability of green spaces and walkability could be used in the future as additional public health measures to reduce the prevalence of hypertension from a population-wide perspective.

### 15.2. Wearables and Telemonitoring in Hypertension

The advancement of digital technologies in healthcare has significantly transformed the approach to hypertension management, allowing near-continuous and accurate out-of-office BP monitoring. Telemonitoring and wearable devices have been progressively incorporated into hypertension management strategies, with the potential to improve treatment adherence and reduce CV complications.^44,598,599^ However, challenges related to the validation, accuracy, and clinical integration of these technologies still need to be overcome for their wider implementation.^600^

Wearables offer the possibility of frequent or even continuous BP measurement, which facilitates the early detection of hypertensive phenotypes such as MH and abnormal BP variability.^600,601^ Studies have shown that remote BP monitoring, when combined with broader digital health and telemedicine strategies, can lead to significant reductions in BP levels.^599^ Furthermore, devices that monitor physical activity and sleep quality, when used in an integrated manner, can help manage BP by encouraging behavioral changes and improving treatment adherence.^602,603^

The reliability of wearables for BP measurement remains a key issue. Although advances have been made, many cuffless devices still lack sufficient validation for clinical use.^604^ International guidelines, such as those from Hypertension Canada and the European Society of Hypertension, recommend caution in adopting these devices and emphasize the need for rigorous validation before clinical application.^605,606^ Furthermore, variability between devices and the influence of factors such as movement and body position can make it difficult to obtain accurate measurements.^73^ Issues of data privacy and regulatory hindrances that still need to be overcome must also be considered.^607^

BP telemonitoring has already demonstrated benefit in clinical practice, especially when combined with multimodal interventions involving physicians, nurses, and pharmacists.^598,608^ Automatic data capture and transmission to digital platforms allows for closer patient monitoring, facilitating adjustment of therapy and personalized treatment.^599^ Furthermore, strategies that use artificial intelligence to analyze BP trends may be able to contribute to a more predictive and preventive approach^609^

Patients with chronic hypertension and older adults appear to benefit most from the use of wearable devices, especially when combined with structured hypertension self-management programs.^610^ Furthermore, the use of these technologies may increase physical activity, a fundamental component of BP management.^603^ However, for these devices to be widely adopted, it is essential that the data they provide are comparable to those obtained using conventional methods, ensuring diagnostic and therapeutic reliability.^73^

Therefore, although wearables and telemonitoring represent a potential revolution in hypertension management, their full incorporation into clinical practice will depend on rigorous validation, standardization of measurements, and effective integration with health systems.^605,609^ The future of hypertension management will likely involve a hybrid approach, combining digital technology with structured clinical monitoring, transforming healthcare from reactive to proactive, enabling precision diagnosis, more personalized and efficient disease management, and preventive strategies.^610^

### 15.3. Hypertension and Intracranial Compliance

Cerebral compliance refers to the ability of cerebral blood vessels to dilate and constrict in response to changes in BP, a phenomenon which plays a crucial role in regulating cerebral blood flow.

The association between hypertension and cerebrovascular diseases (as well as cognitive decline) is well established, and the complex pathophysiological bases of this association are increasingly better understood. Hypertension promotes structural changes throughout the cerebral vasculature, and the concept of hemodynamic pulsatility, which encompasses pressure variability, central pressure, and arterial stiffness, has been identified as a mechanism that can impact brain structure and function.^611-613^

Assessment of intracranial compliance and pressure was long only possible through invasive methods. However, there are now validated noninvasive methods for assessment of these phenomena.^614^ In this context, one study in an animal model showed changes in intracranial compliance just three weeks after induction of hypertension.^615^ More recently, a Brazilian study showed a 45.6% prevalence of changes in intracranial compliance among individuals with hypertension receiving outpatient follow-up.^616^

These findings should serve as a warning to the scientific community and highlight the need to integrate knowledge about the behavior of peripheral and central BP and the behavior of intracranial pressure and compliance in patients with hypertension. Furthermore, these data appear to indicate that regulation of the cerebral circulation and the blood–brain barrier may be compromised from the very earliest stages of hypertensive disease.^617^

### 15.4. Ultra-low-Dose Triple and Quadruple Antihypertensive Drug Combinations

Low BP control rates in Brazil and worldwide^618^ require new, streamlined, more effective strategies to address low treatment adherence and therapeutic inertia.

The WHO^619^ and Brazilia^31^ and international^45,117^ guidelines all recommend the use of fixed-dose combinations, preferably in single-pill formulations, as the first-line treatment strategy of choice for the vast majority of patients with hypertension. However, monotherapy still remains highly prevalent.^620^

The proposal to use ultra-low-dose combinations of three or four antihypertensive drugs in a single pill as first-line treatment for hypertension is not as recent as it may appear. The rationale includes greater efficacy and tolerability, greater treatment adherence, and less therapeutic inertia.

A 2017 systematic review and meta-analysis^621^ included 42 trials involving 20,284 participants. Thirty-six comparisons evaluated quarter-dose monotherapy vs. placebo and found a BP reduction of −4.7/−2.4 mm Hg (p < 0.001). Six comparisons were of dual quarter-dose combinations vs. placebo, which found a BP reduction of −6.7/−4.4 mm Hg (p < 0.001). There were no trials of triple quarter-dose combinations vs. placebo, but a quadruple quarter-dose combination trial reported a BP reduction of −22.4/−13.1 mm Hg vs. placebo (p < 0.001). Compared with standard-dose monotherapy, double and quadruple quarter-dose combinations were associated with BP differences of 3.7/2.6 (p < 0.001), 1.3/−0.3 (NS), and −13.1/−7.9 (p < 0.001) mm Hg, respectively. In terms of adverse events, quarter-dose monotherapy or dual therapy were not significantly different from placebo, and fewer adverse events were observed compared with standard-dose monotherapy. These findings suggested that quarter-dose combinations could provide improved efficacy and tolerability.^621^

A 2023 meta-analysis^622^ corroborated these findings, including trials of triple and quadruple quarter-dose combinations versus their individual components. The primary outcome was the mean reduction in SBP when comparing these combinations with standard-dose monotherapy, usual care, or placebo. Seven clinical trials enrolling 1918 patients were included. The ultra-low-dose combinations were associated with a greater mean reduction in SBP than initial monotherapy or usual care (mean reduction = 7.4 mm Hg; 95% CI, 4.3-10.5) or placebo (mean reduction = 18.0 mm Hg; 95% CI, 15.1-20.8) at up to 12 weeks of follow-up.

Chart 15.1 shows the characteristics and results of key RCTs of ultra-low-dose triple and quadruple drug combinations.

**Chart 15.1:** Major randomized clinical trials of ultra-low-dose triple and quadruple drug combinations for treatment of hypertension

Study	Study design (n)	Intervention Therapeutic strategy and doses	Comparator regimen	Primary outcome	Result
TRIUMPH^623^ (2018)	RCT, open-label (n=700)	T 20 mg A 2.5 mg C 12.5 mg (multiple pills)	Usual care	Rate of achievement of BP target < 140/90 mm Hg (or < 130/80 mm Hg in DM/CKD) at 6 months	Increased BP control rate with the triple combination vs. usual care at 6 months (70% vs. 55%, difference=12.7% [95% CI, 3.2%-22.0%]; p < 0.001).
TRIUMPH^624^ (2020)	RCT, open-label (n=700)	T 20 mg A 2.5 mg C 12.5 mg (multiple pills)	Usual care	Therapeutic inertia* at 12 weeks of treatment	Therapeutic inertia was more frequent with the triple combination than with usual care at 12 weeks (81 of 90 [90%] vs. 116 of 179 [64.8%]; p < 0.001).
Sung et al.^625^ (2022)	Double-blind RCT (n=176)	T+A+C 10/1.25/3.125 mg (quarter-dose) T+A+C 13.3/1.7/4.2 mg (one-third dose) T+A+C 20/2.5/6.25 mg (half dose) (single pill)	A 5 mg A 10 mg T 80 mg Placebo	Difference in mean sitting SBP at 8 weeks	Mean sitting SBP and DBP were significantly lower with quarter-dose and half-dose triple combinations vs. placebo, A5, A10, and T80
QUARTET^626^ (2021)	Double-blind RCT (n=591)	Irb 37.5 mg A 1.25 mg I 0.625 mg B 2.5 mg (single pill)	Irb 150 mg	Difference in unsupervised office SBP at 12 weeks.	SBP was 6.9 mm Hg lower in the quadruple combination group (95% CI, 4.9–8.9; p < 0.0001) BP control rates were higher in the intervention group (76%) vs. the control group (58%; relative risk [RR] 1.30; 95% CI, 1.15–1.47; p < 0.0001)
QUARTET^627^ USA (2024)	Double-blind RCT (n=62)	Cand 2 mg A 1.25 mg I 0.625 mg B 2.5 mg (single pill)	Cand 8 mg	Difference in office SBP at 12 weeks	No significant difference in SBP [**−**4.8 mm Hg (95% CI, **−**10.8 to 1.3; p = 0.123)], but greater mean reduction in DBP [**−**4.9 mm Hg (95% CI, **−**8.6 to **−**1.3); p = 0.009] in intervention group vs. control group at 12 weeks
QUARTET^628^ Therapeutic inertia (2024)	Double-blind RCT (n=591)	Irb 37.5 mg A 1.25 mg I 0.625 mg B 2.5 mg (single pill)	Irb 150 mg	Therapeutic inertia*	Therapeutic inertia occurred in a smaller number of patients randomized to the quadripill compared to those on monotherapy
Ojji et al. NIGERIA^629^ (2024)	Double-blind RCT (n=300)	Triple combination – accelerated titration T+A+I 10/1.25/0.625 mg T+A+I 20/2.5/1.25 mg T+A+I 40/5/2.5 mg (single pill)	Standard care A5 mg A5 + L50 mg A10 + L100 mg A + L + H	Reduction in home BP at 6 months	Triple combination was associated with a 31 mm Hg reduction in home SBP (95% CI, 28–33 mm Hg) vs. 26 mm Hg (95% CI, 22–28 mm Hg) in the standard-care group (adjusted difference = **−**5.8 mm Hg [95% CI, **−**8.0 to **−**3.6]; p < 0.001) at 6 months

BP: blood pressure; DM: diabetes mellitus; CKD: chronic kidney disease; RCT: randomized clinical trial. A: amlodipine; B: bisoprolol; C: chlorthalidone; Cand: candesartan; H: hydrochlorothiazide; I: indapamide; T: telmisartan; SBP: systolic blood pressure; DBP: diastolic blood pressure; QUARTET: Quadruple Ultra-Low-Dose Treatment for Hypertension; RR: risco relativo; TRIUMPH: Treating Resistant Hypertension Using Lifestyle Modification to Promote Health.

* Failure to intensify treatment in patients with uncontrolled BP

The results of these clinical trials suggest greater antihypertensive efficacy and improved tolerability of triple and quadruple combinations at ultra-low doses compared to placebo, monotherapy, or usual care. Further studies with larger sample sizes and longer follow-up periods are needed to assess the impact of the ultra-low-dose therapeutic strategy on the protection of target organs and protection against major adverse CV outcomes.

### 15.5. Novel Drugs for the Treatment of Hypertension

Currently, several classes of drugs are being studied for the treatment of hypertension, aiming to improve BP control and reduce residual CV and renal risks.

Seven different pharmacological classes have been identified as emerging options – some of them highly promising – for the treatment of RH and RfH, namely: nonsteroidal mineralocorticoid receptor antagonists; aminopeptidase A inhibitors; dual endothelin antagonists; hepatic angiotensinogen attenuators; dual angiotensin II receptor-neprilysin inhibitors; and aminopeptidase inhibitors.^547,630-632^

Chart 15.2 summarizes key findings of trials of these novel drugs which may be incorporated into the pharmacological armamentarium for treatment of hypertension in the coming years.

**Chart 15.2:** Novel drug strategies for treatment of hypertension

DRUG/CLASS	RESULTS OF PIVOTAL TRIALS
**1. Neprilysin inhibitor + angiotensin II receptor blocker**	
Sacubitril/Valsartan^550^	Significant reduction in central pressure, 24-hour BP, and nocturnal BP, with reversal of nighttime BP dipping. Indicated for HFrEF and hypertension with HF (secondary prevention). Not to be combined with ACEIs or ARBs
**2. Nonsteroidal mineralocorticoid receptor antagonists**	
Finerenone^236,551^	Reduces renal and CV events in diabetics when added to maximum tolerated RAAS blockade. Less of an antihypertensive effect than spironolactone
Apararenone, Esaxerenone, and Ocedurenone^633-635^	These 3 MRAs are at different stages of development. • Esaxerenone reduced BP, decreased albuminuria, and was well tolerated and effective in controlling nocturnal hypertension, especially in older adults. • The BLOCK-CKD trial demonstrated that ocedurenone is effective in reducing SBP in several patient subgroups, including those with diabetes, very high albuminuria, and different stages of CKD. In a phase II study of patients with RH, ocedurenone (KBP-5074) 0.5 mg reduced office BP from −6.9 to −13.1 mm Hg, depending on the dose and subgroup, with a favorable safety profile.^636^ • Apararenone is a nonsteroidal MRA that still requires further clinical trials to determine its efficacy, tolerability, and safety.^637^
**3. Aldosterone synthase inhibitors (selective CYP11B2 inhibitors)**638	
Baxdrostat^552,639^	BrigHTN1: phase II trial - efficacy and safety of baxdrostat in patients with resistant hypertension. SBP decreased by −20.3, −17.5, and −12.1 mm Hg respectively with 2 mg, 1 mg, and 0.5 mg baxdrostat vs. −9.4 mm Hg in the placebo group. Good tolerability and safety. Dose-dependent increase in serum potassium levels. Ongoing phase III clinical trial – 1 mg and 2 mg vs. placebo in RH
Lorundrostat^640-643^	Target-HTN: phase II. Reduced BP in a small study of patients with RH. Six participants had hyperkalemia > 6.0 mEq/L. The Launch-HTN RCT with 1083 patients showed a significant decrease in automated office SBP in the treatment group (-16.9 mm Hg vs. −7.9 mm Hg in the placebo group), but with 50% mild and moderate adverse events
Dexfadrostat	Tested in a small group of patients with primary hyperaldosteronism with promising results^644^
Vicadrostat	Still in phase 1 trials in healthy volunteers
**4. Dual endothelin A/endothelin B (ETA/ETB) receptor antagonist**	
Aprocitentan^554,645^	Office SBP reduced –15.3 mm Hg (12.5 mg dose) and –15.2 mm Hg (25 mg dose) vs. –11.5 mm Hg with placebo (p = 0.005) in the PRECISION trial Adverse effects: mild to moderate edema in 9% (12.5 mg dose) and 18% (25 mg dose). FDA-approved in March 2024, in combination with other antihypertensives^646^
**5. Small interfering RNAs targeting hepatic angiotensinogen**	
Zilebesiran^556,547^	Antisense oligonucleotides that inhibit RNA translation and reduce hepatic angiotensinogen synthesis Subcutaneous administration (250 to 500 mg) every 3 to 6 months • Phase I trial: Reduction in 24-hour BP for 24 weeks. Mild injection site reactions • Phase II trial (Kardia-1): sustained reductions in angiotensinogen and 24-h SBP (–12.84 mm Hg) in patients with mild and moderate hypertension for up to 6 months
IONIS-AGT-LRx^557^	Antisense inhibitor administered subcutaneously. Initial studies demonstrated safety, significant reduction in angiotensinogen levels compared to placebo, and trends in BP reduction
Brain aminopeptidase A inhibitors	
Firibastat^648,649^	Aminopeptidase A is the enzyme responsible for catalyzing angiotensin III synthesis and is a potential druggable target for the treatment of hypertension. Safe and effective in phase II trials; a phase III trial is planned
**6. SGLT2 inhibitors**	
Empagliflozin and Dapagliflozin^650,651^	Several meta-analyses have confirmed the ability of SGLT2 inhibitors to modestly reduce BP in patients with T2DM. It is a class effect. In one meta-analysis of 43 RCTs including 22, 528 patients, the antihypertensive effect was −2.5 mm Hg in SBP and −1.5 mm Hg in DBP. Another analysis of 7 RCTs including 2381 participants assessed the effects of SGLT2 inhibitors on 24-hour ABPM. The reduction was −3.6 mm Hg in SBP and −1.7 mm Hg for 24-hour DBP on ABPM, regardless of SGLT2 inhibitor dose.

ABPM: ambulatory blood pressure monitoring; ACEI: angiotensin-converting enzyme inhibitor; ARB: angiotensin ii receptor blocker; BLOCK-CKD: Blood Pressure in Chronic Kidney Disease; BP: blood pressure; BrigHTN1: Baxdrostat in Resistant Hypertension; CKD: chronic kidney disease; CV: cardiovascular; DBP: diastolic blood pressure; ETA: endothelin receptor A; ETB: endothelin receptor B; FDA: Food and Drug Administration; HF: heart failure; HFrEF: heart failure with reduced ejection fraction; MRA: mineralocorticoid receptor antagonist; RAAS: renin-angiotensin-aldosterone system; RCT: randomized controlled trial; RH: resistant hypertension; SBP: systolic blood pressure; SGLT2: sodium–glucose cotransporter 2 inhibitors; Target-HTN: Trial on the Safety and Efficacy of MLS-101 in Patients With Uncontrolled Hypertension.

## List of abbreviations

ABI: Ankle-Brachial Index
ABPM: Ambulatory Blood Pressure Monitoring
ACCORD: Action to Control Cardiovascular Risk in Diabetes
ACE: Angiotensin-Converting Enzyme
ACEI: Angiotensin-Converting Enzyme Inhibitor
ACR: Albumin-To-Creatinine Ratio
ACS: Acute Coronary Syndrome
ACTH: adrenocorticotropic hormone
ADVANCE: Action in Diabetes and Vascular Disease: Preterax and Diamicron MR Controlled Evaluation
AF: Atrial Fibrillation
AHI: Apnea-hypopnea index
AKI: Acute Kidney Injury
APE: Acute Pulmonary Edema
ARB: Angiotensin II Receptor Blocker
ARIC: Atherosclerosis Risk in Communities
ARTS-DN: Mineralocorticoid Receptor Antagonist Tolerability Study–Diabetic Nephropathy
AV: Atrioventricular
BB: Beta-Blocker
BHG: Brazilian Hypertension Guidelines
BiPAP: Bilevel Positive Airway Pressure
BLOCK-CKD: Blood Pressure in Chronic Kidney Disease
BMI: Body Mass Index
BP: Blood Pressure
BPROAD: Intensive Blood-Pressure Control in Patients with Type 2 Diabetes
BrigHTN1: Baxdrostat in Resistant Hypertension
CAD: Coronary Artery Disease
CBP: Central Blood Pressure
CCB: Calcium Channel Blocker
cGMP: cyclic guanosine monophosphate
CKD: Chronic Kidney Disease
CNS: Central Nervous System
COC: Combined Oral contraceptive
CPAP: Continuous Positive Airway Pressure
CRIC: The Chronic Renal Insufficiency Cohort Study
CSF: cerebrospinal fluid
CT: Computed Tomography
CTA: computed tomography angiography
CTD: Clortalidona
CV: Cardiovascular
CVD: Cardiovascular Disease
CVRF: Cardiovascular Risk Factors
DASH: Dietary Approaches to Stop Hypertension
DATASUS: Information Technology Department of the Brazilian Unified Health System
DBP: Diastolic Blood Pressure
DHP: Dihydropyridine
DIU: Diuretics
DM: Diabetes Mellitus
DXA: Dual-Energy X-Ray Absorptiometry
ECG: Electrocardiogram
ED: Emergency Department
EF: Ejection Fraction
eGFR: Estimated Glomerular Filtration Rate
EMS: Emergency Medical Service
ESF: Family Health Teams
ESO: European Stroke Organisation
ESPRIT: Effects of Intensive Systolic Blood Pressure Lowering Treatment in Reducing Risk of Vascular Events
ETA: Endothelin Receptor A
ETB: Endothelin Receptor B
FDA: Food and Drug Administration
FMF: Fetal Medicine Foundation
FROM-J: Frontier of Renal Outcome Modifications in Japan
GH: Growth Hormone
GLP-1: Glucagon-Like Peptide-1
GLP-1 RA: Glucagon-Like Peptide-1 Receptor Agonist
GRADE: Grading of Recommendations Assessment, Development and Evaluation
GS: Gait Speed
HbA1C: Glycated Hemoglobin
HBPM: Home Blood Pressure Monitoring
HCTZ: Hydrochlorothiazide
HE: Hypertensive Emergency
HELLP: Hemolysis, Elevated Liver Enzymes, and Low Platelets
HF: Heart Failure
HFpEF: Heart Failure with Preserved Ejection Fraction
HFrEF: Heart Failure with Reduced Ejection Fraction
HMOD: Hypertension-Mediated Organ Damage
HOPE-3: Heart Outcomes Prevention Evaluation 3
HR: Heart Rate
HTN: Hypertension
HYVET: Hypertension in the Very Elderly Trial
ICU: Intensive Care Unit
IDNT: Irbesartan Diabetic Nephropathy Trial
IGF-1: Insulin-Like Growth Factor 1
IM: Intramuscular
IV: Intravenous
KDIGO: Kidney Disease: Improving Global Outcomes
KWB: Keith-Wagener-Barker Classification
LVH: Left Ventricular Hypertrophy
MDMA: Methylenedioxymethamphetamine
MET: Metabolic Equivalent of Task
MH: Masked Hypertension
MHT: Menopausal Hormone Therapy
MI: Myocardial Infarction
MRA: Mineralocorticoid Receptor Antagonist
MRI: Magnetic Resonance Imaging
NHANES: National Health and Nutrition Examination Survey
NIHSS: National Institutes of Health Stroke Scale
NPM: Nonpharmacological Measure
NTG: Nitroglycerin
O2: Oxygen
OCP: Oral Contraceptive Pill
OH: Orthostatic Hypotension
ONTARGET: Ongoing Telmisartan Alone and in combination with Ramipril Global Endpoint Trial
OSA: Obstructive Sleep Apnea
PA: Physical Activity
PAD: Peripheral Artery Disease
PCOS: Polycystic Ovary Syndrome
PD: Peritoneal Dialysis
PE: Preeclampsia
PHC: Primary Health Care
PIGF: Serum Placental Growth Factor
PP: Pulse Pressure
PPH: Postprandial Hypotension
PRES: Posterior Reversible Encephalopathy Syndrome
PREVENT: Predicting Risk of Cardiovascular Disease Events
PSNS: Parasympathetic Nervous System
PTH: Parathyroid Hormone
PVR: Peripheral Vascular Resistance
PWV: Pulse Wave Velocity
RAAS: Renin-Angiotensin-Aldosterone System
RAS: Health Care Network
RCT: Randomized Controlled Trial
RESPECT: Randomized Evaluation of Recurrent Stroke Comparing PFO Closure to Established Current Standard of Care Treatment
RfH: Refractory Hypertension
RH: Resistant Hypertension
RHr: Refractory Resistant Hypertension
SBC: Brazilian Society of Cardiology
SBH: Brazilian Society of Hypertension
SBN: Brazilian Society of Nephrology
SBP: Systolic Blood Pressure
SCORE2: Systematic Coronary Risk Evaluation 2
SGLT2: Sodium-Glucose Cotransporter 2
SGLT2 inhibitors: Sodium–Glucose Cotransporter 2 Inhibitors
SH: Secondary Hypertension
SHEP: Systolic Hypertension in the Elderly Program
SNP: Sodium Nitroprusside
SNS: Sympathetic Nervous System
SPRINT: Systolic Blood Pressure Intervention Trial
SPRINT-MIND: Systolic Blood Pressure Intervention Trial – Memory and Cognition in Decreased Hypertension
STEP: Semaglutide Treatment Effect in People with Obesity
SUS: Brazilian Unified Health System
SYST-EUR: Systolic Hypertension in Europe
Target-HTN: Trial on the Safety and Efficacy of MLS-101 in Patients With Uncontrolled Hypertension
TIA: Transient Ischemic Attack
TNT: Treating to New Targets
TRIDENT: Triple Therapy Prevention of Recurrent Intracerebral Disease Events Trial
TSH: Thyroid-Stimulating Hormone
UBS: Primary Health Care Centers
UH: Uncontrolled Hypertension
uMH: Uncontrolled Masked Hypertension
US: Ultrasound
uWCH: Uncontrolled White-Coat Hypertension
VIGITEL: Brazilian Telephone Survey for Surveillance of Risk and Protective Factors for Chronic Diseases
WC: Waist Circumference
WCE: white-Coat Effect
WCH: White-Coat Hypertension
WHO: World Health Organization
